# Supplementary material for: Ligand-promoted cobalt-catalyzed radical hydroamination of alkenes
Source: Nat Commun. 2020 Feb 7;11:783. doi: 10.1038/s41467-020-14459-x (PMC7005876; doi:10.1038/s41467-020-14459-x)
Supplement: Supplementary file 2 — Supplementary Information [file 41467_2020_14459_MOESM2_ESM.pdf]

Ligand-Promoted Cobalt-Catalyzed Radical Hydroamination of Alkenes

Supplementary Information

*Xuzhong Shen, Xu Chen, Jieping Chen, Yufeng Sun, Zhaoyang Cheng, and Zhan Lu**

Department of Chemistry, Zhejiang University, Hangzhou 310058, China

Correspondence and requests for materials should be addressed to Z.L. (email: luzhan@zju.edu.cn)

Supplementary Figures

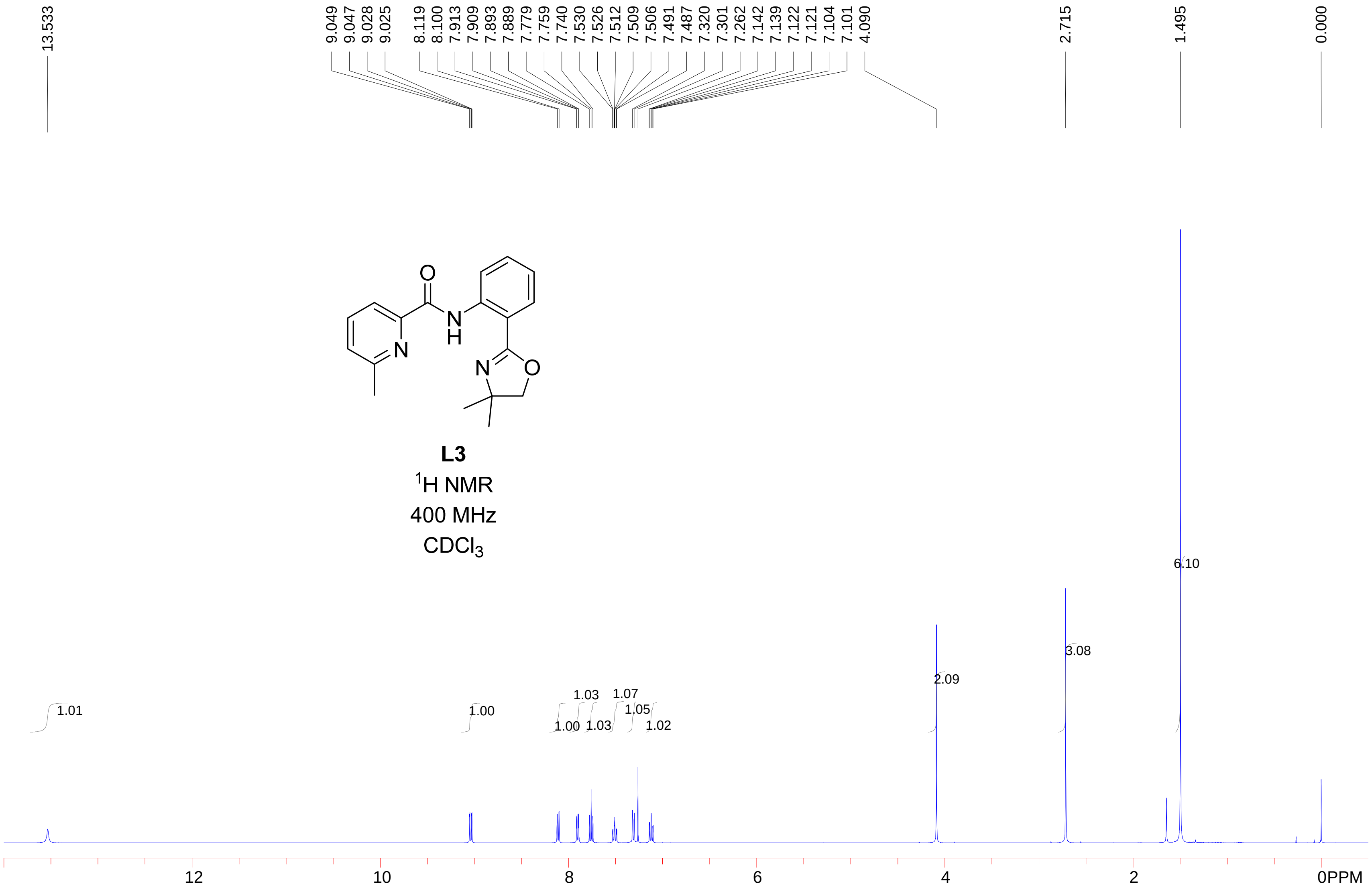

Supplementary Figure 1. ^{1}H NMR spectrum for **L3**

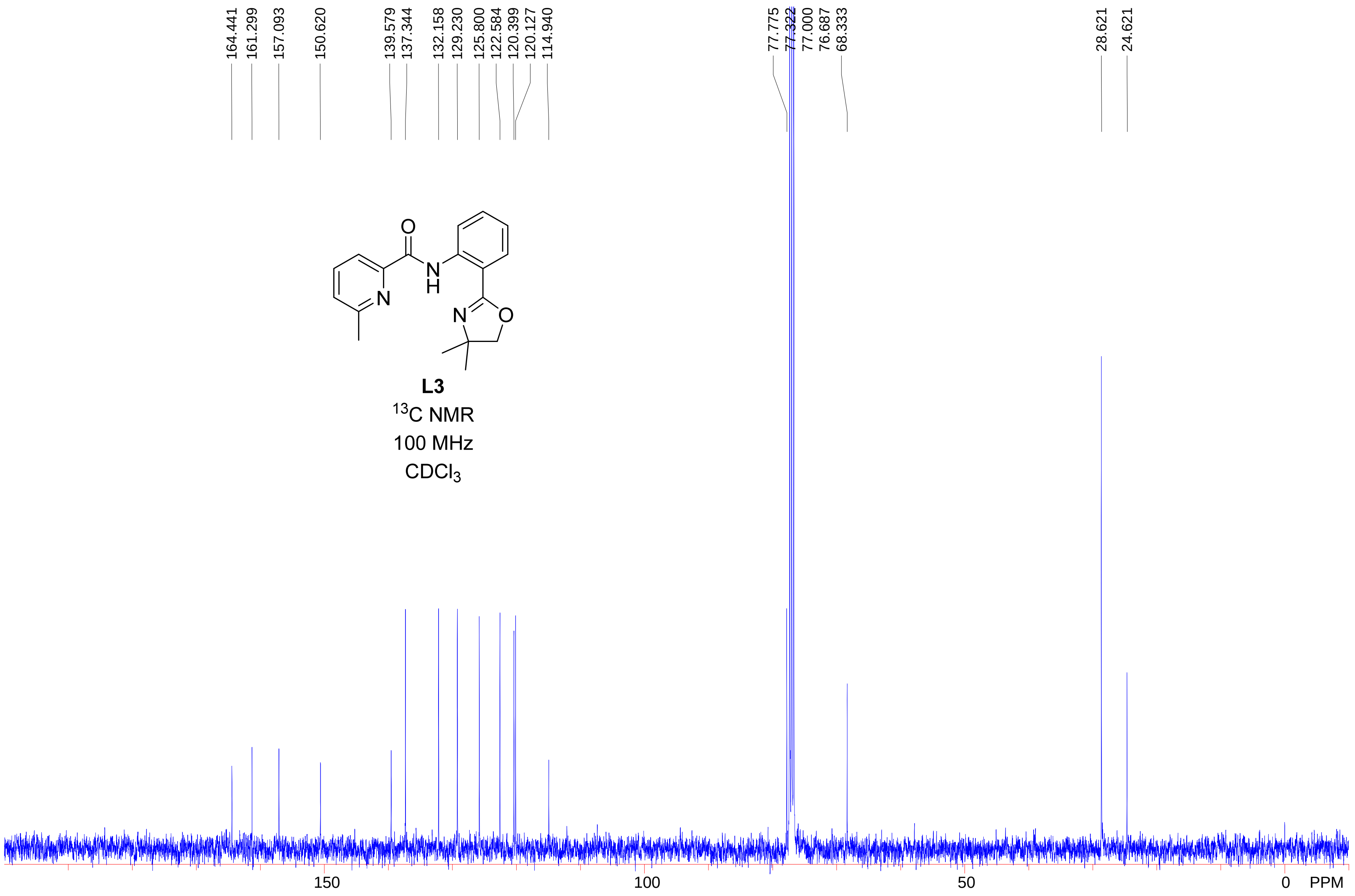

Supplementary Figure 2. ^{13}C NMR spectrum for **L3**

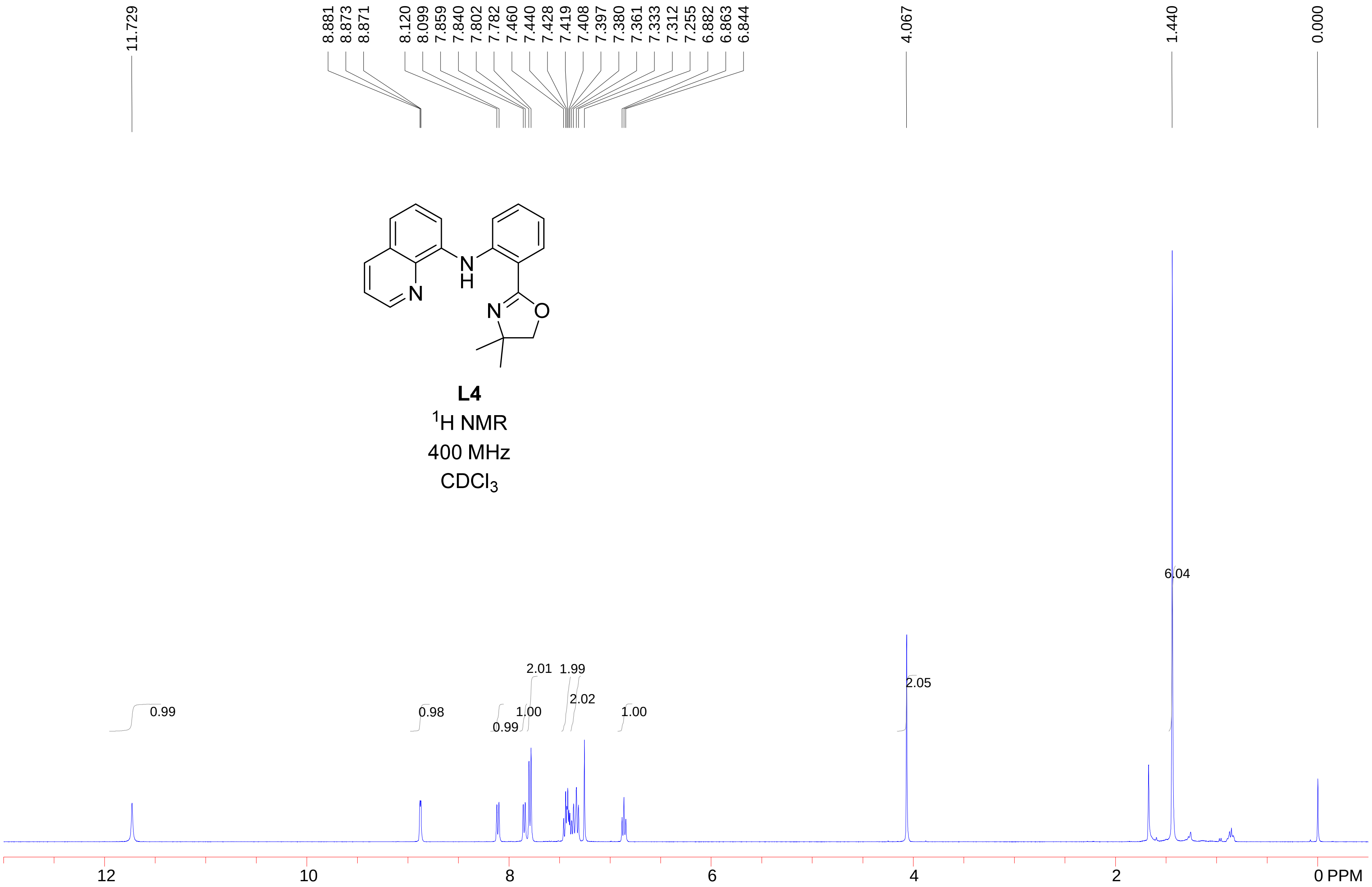

Supplementary Figure 3. ^{1}H NMR spectrum for **L4**

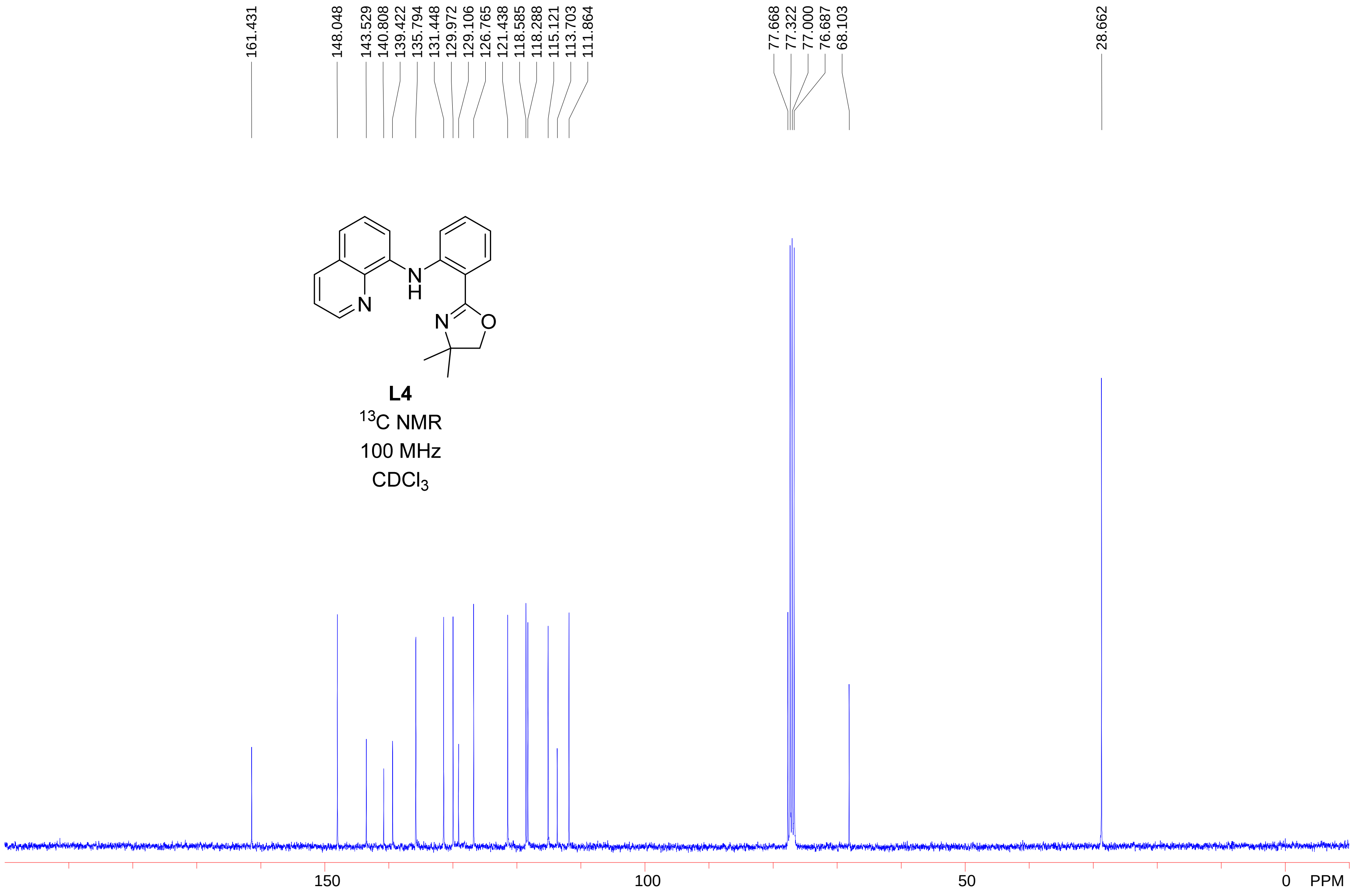

Supplementary Figure 4. ^{13}C NMR spectrum for **L4**

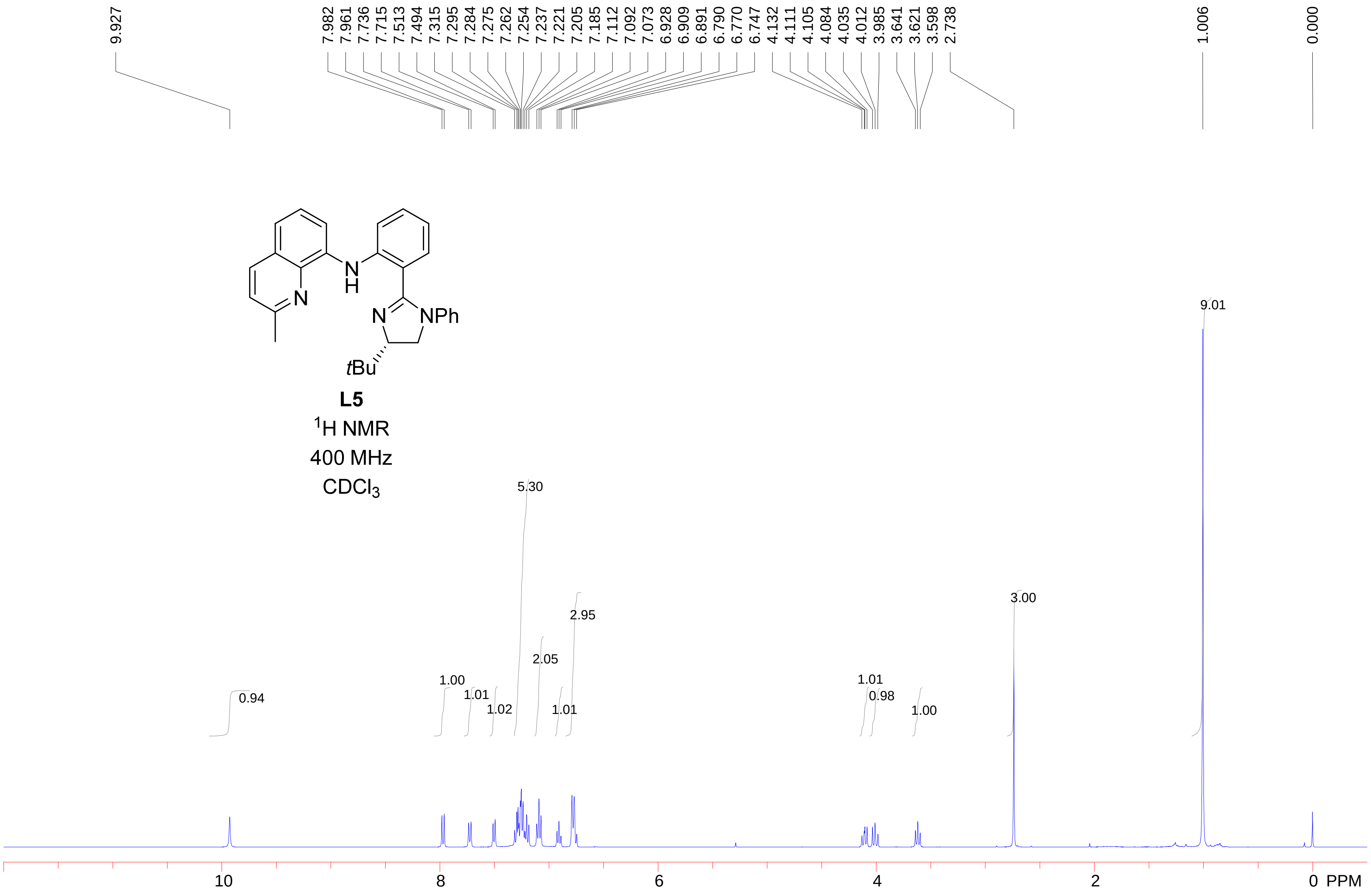

Supplementary Figure 5. ^{1}H NMR spectrum for **L5**

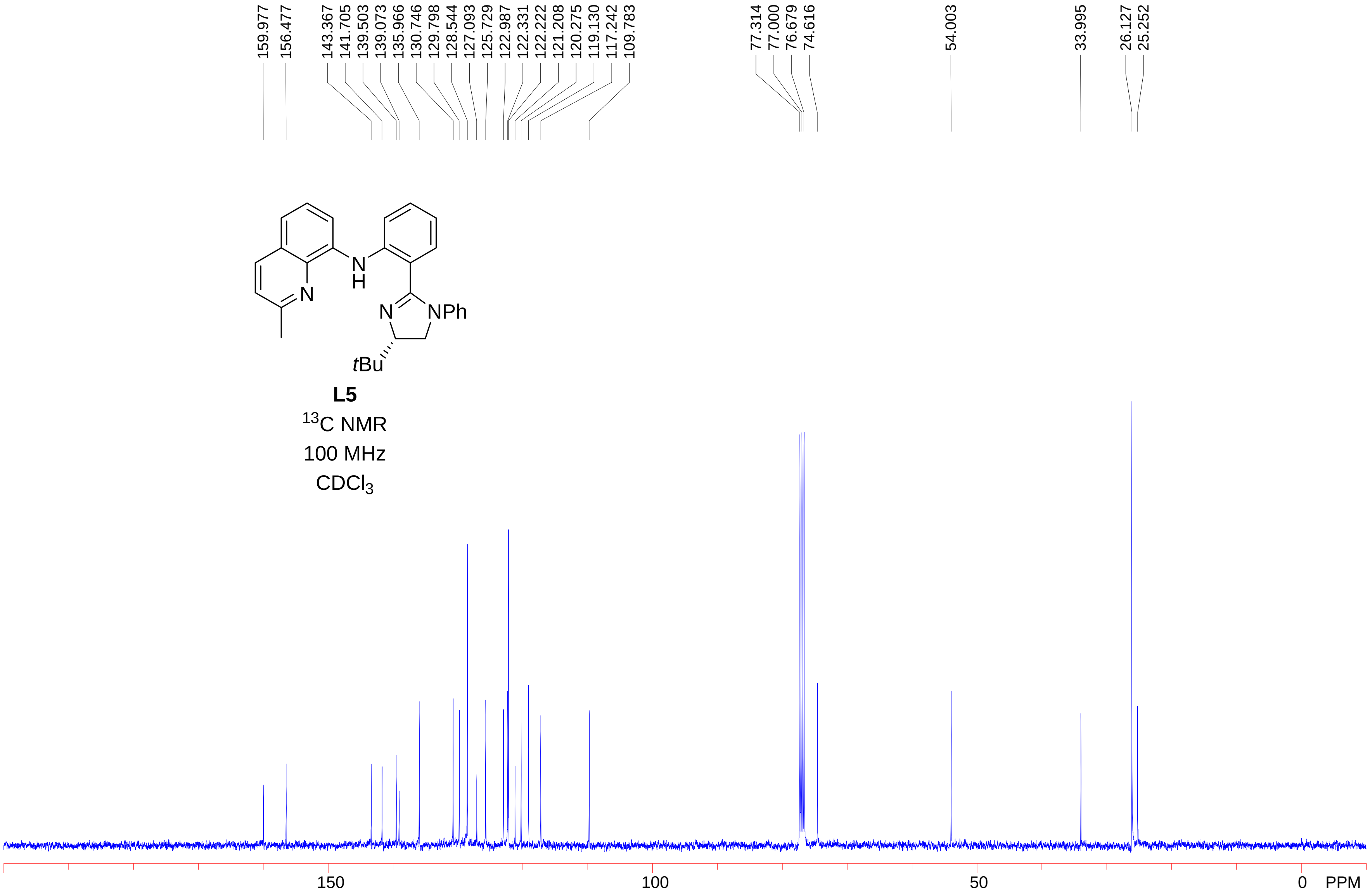

Supplementary Figure 6. ^{13}C NMR spectrum for **L5**

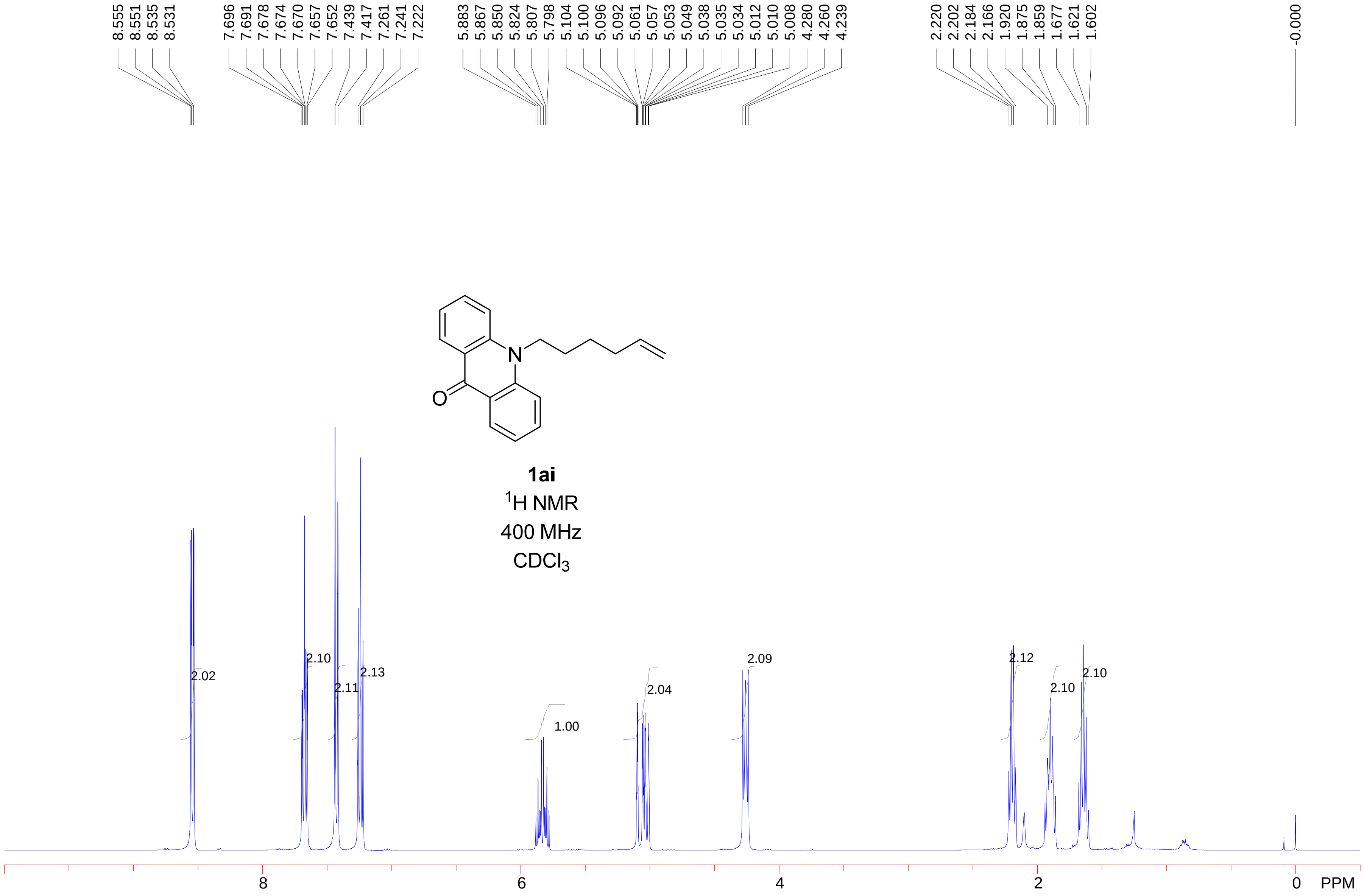

Supplementary Figure 7. ^{1}H NMR spectrum for **1ai**

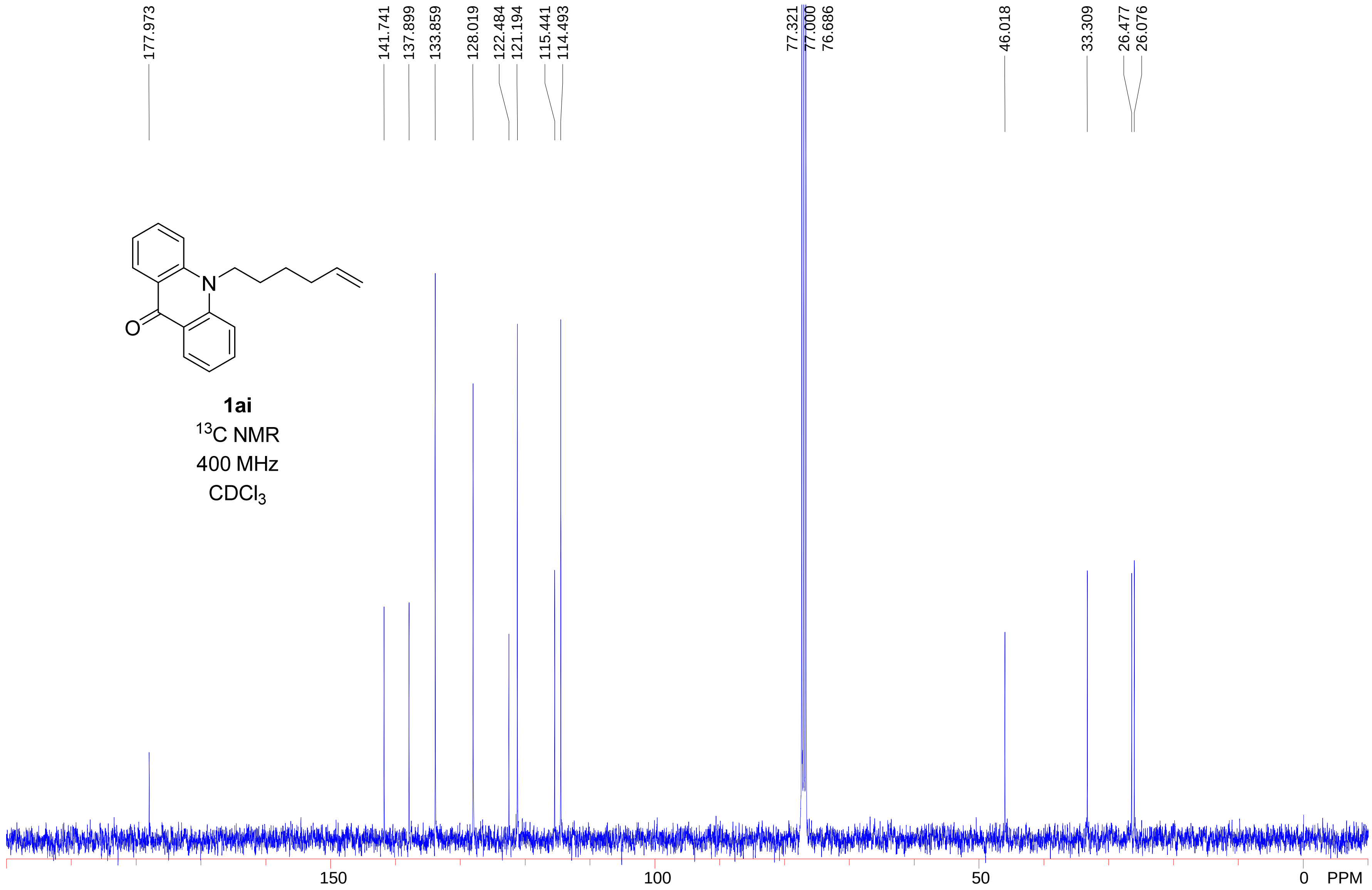

Supplementary Figure 8. ^{13}C NMR spectrum for **1ai**

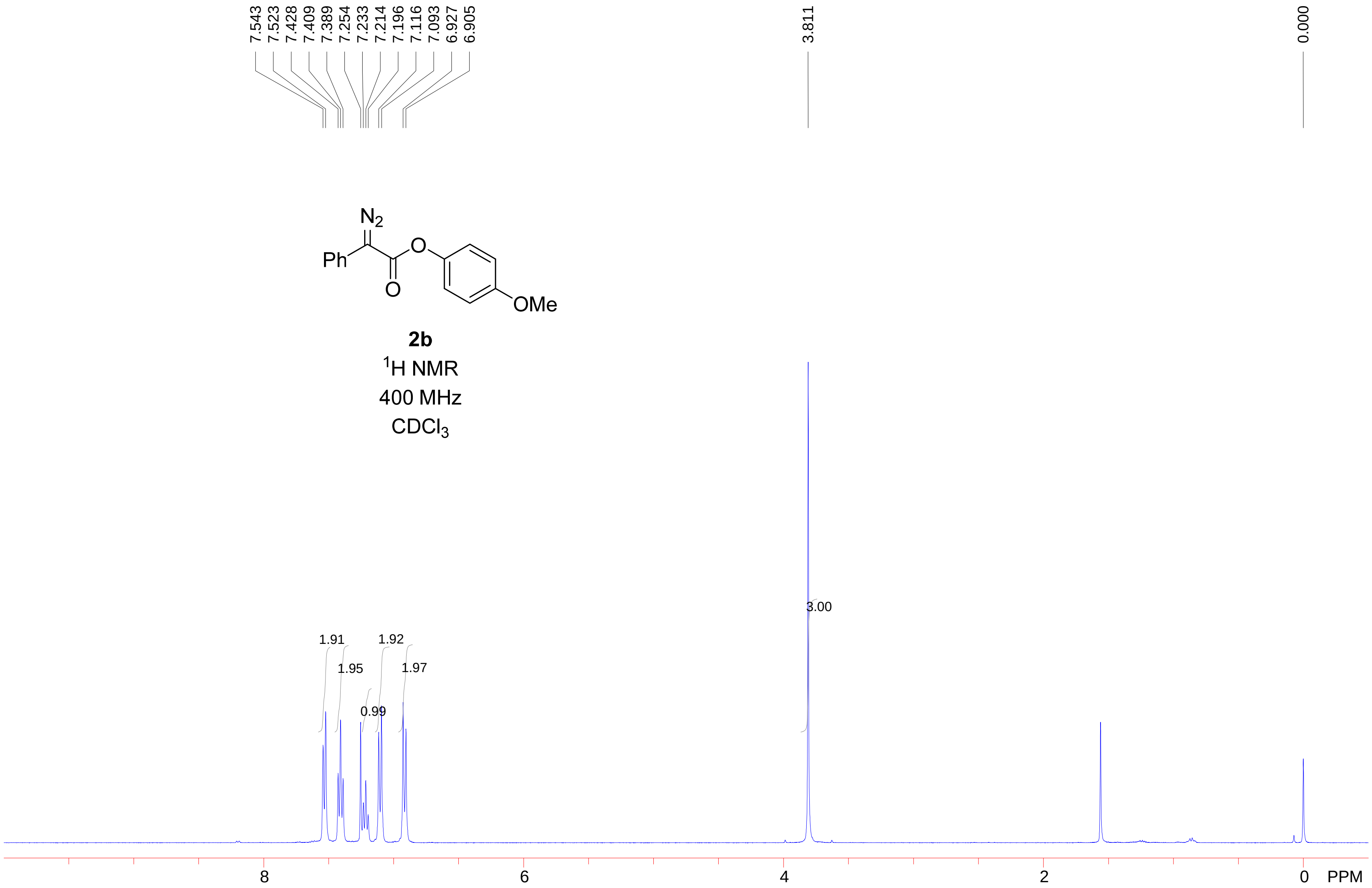

Supplementary Figure 9. ^{1}H NMR spectrum for **2b**

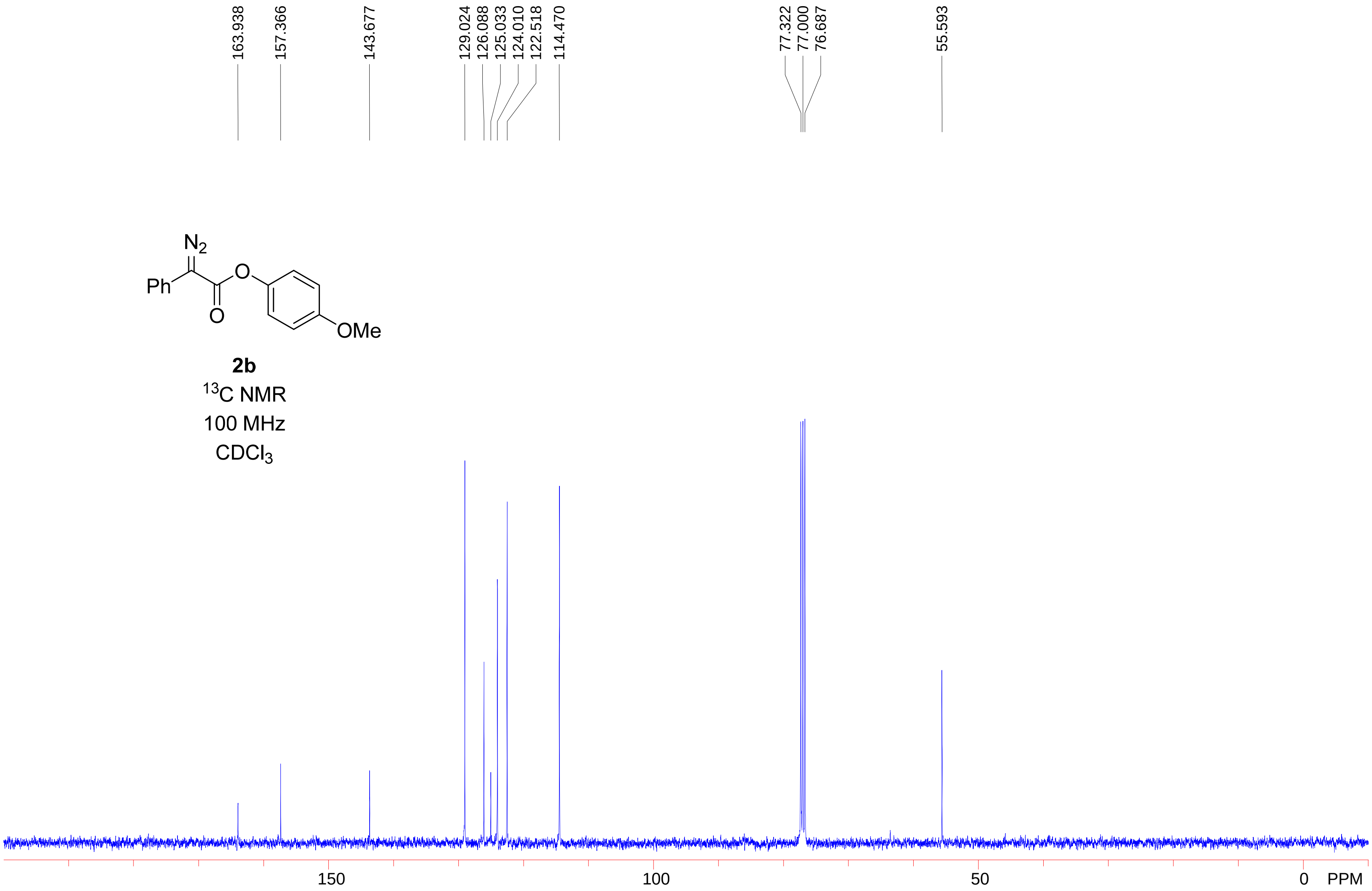

Supplementary Figure 10. ^{13}C NMR spectrum for **2b**

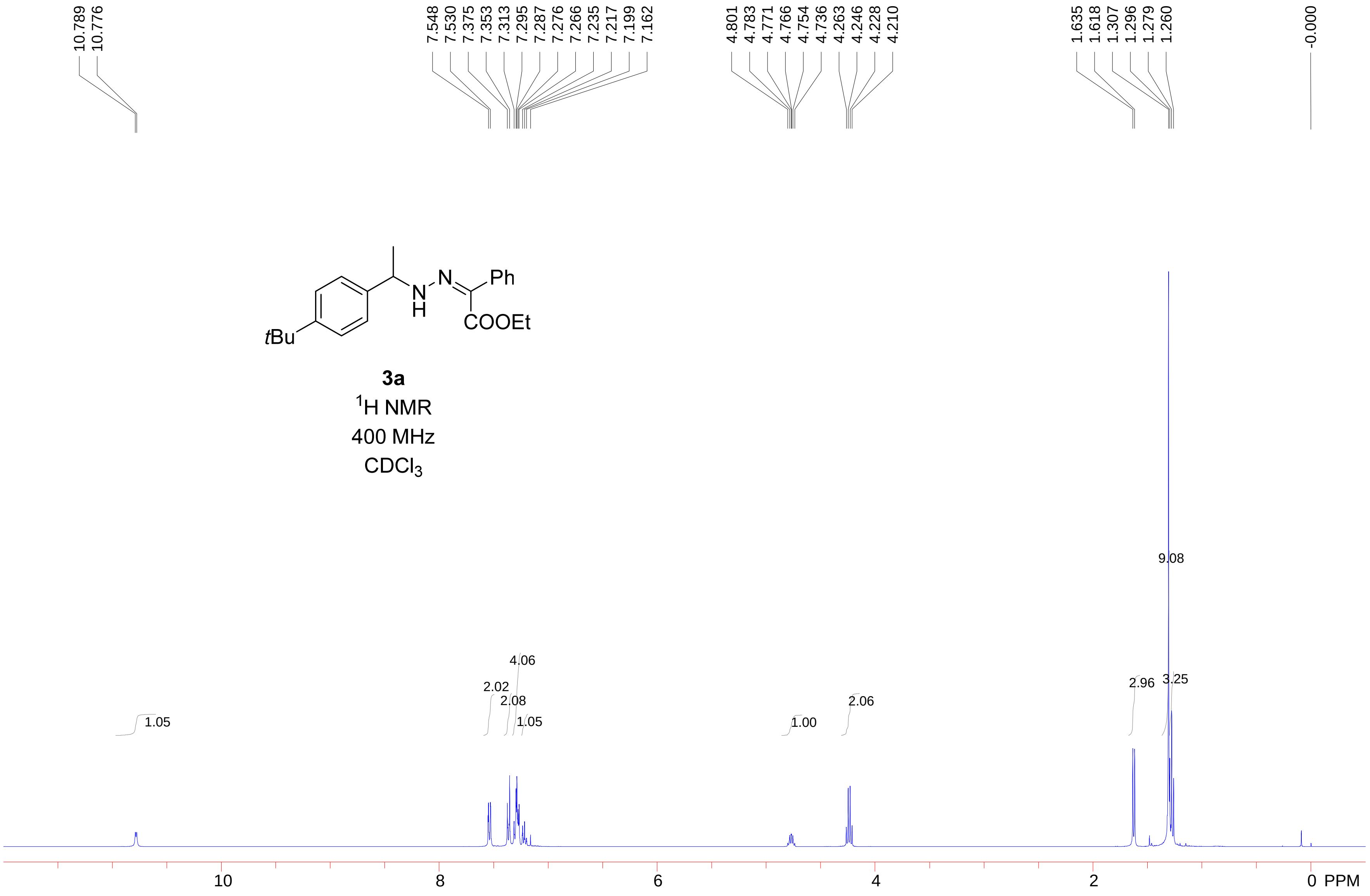

Supplementary Figure 11. ^{1}H NMR spectrum for **3a**

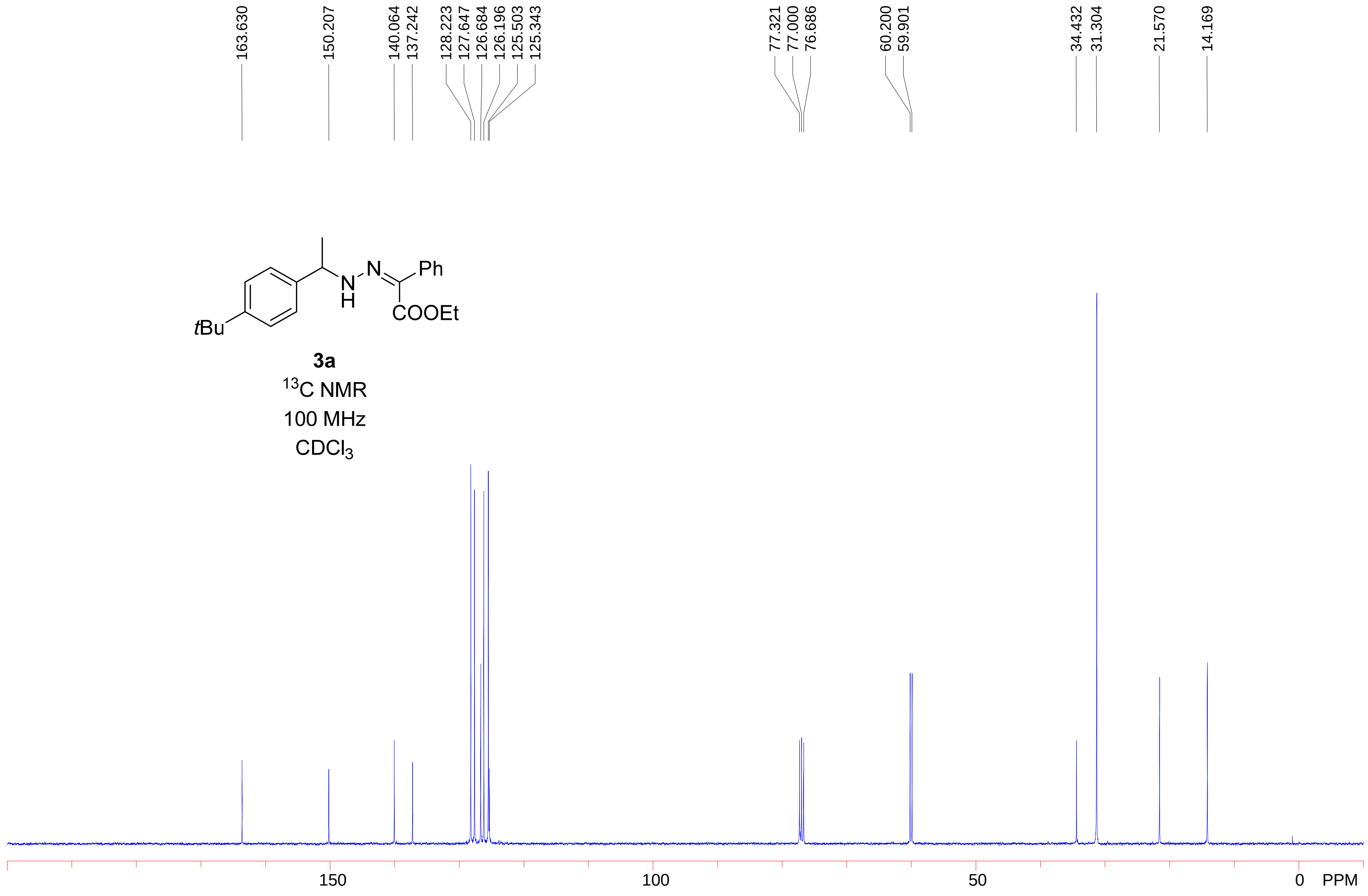

Supplementary Figure 12. ^{13}C NMR spectrum for **3a**

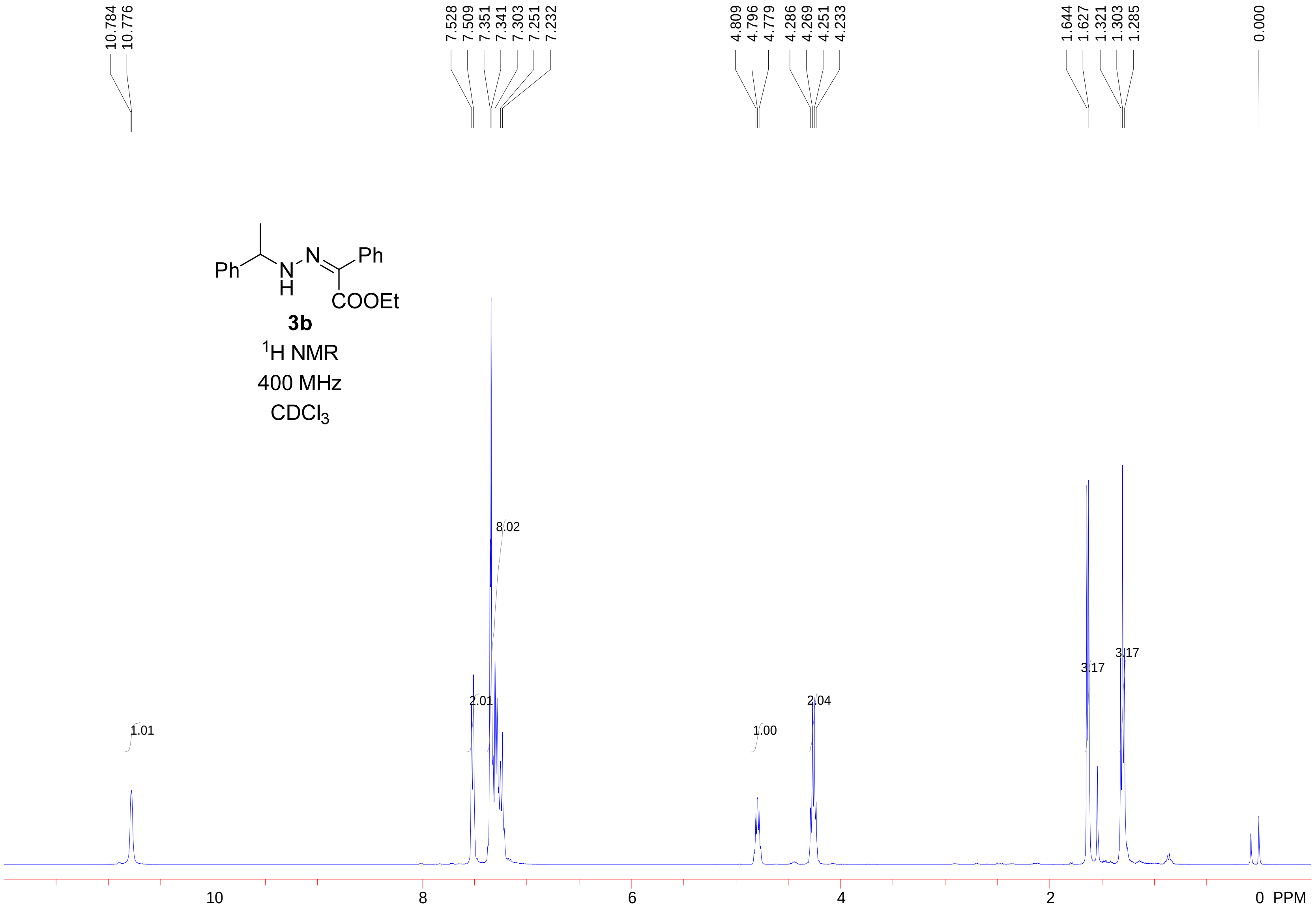

Supplementary Figure 13. ^{1}H NMR spectrum for **3b**

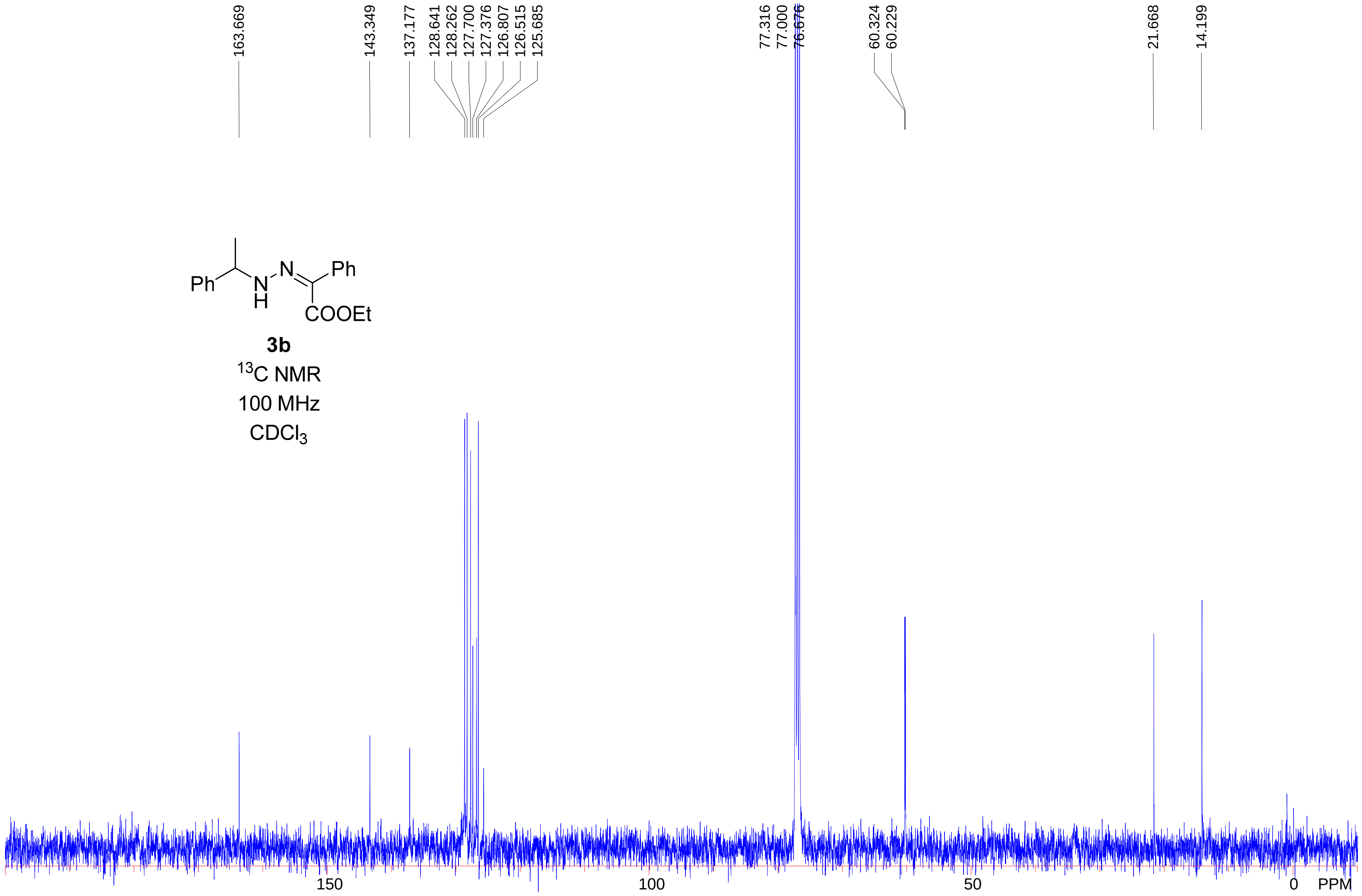

Supplementary Figure 14. ^{13}C NMR spectrum for **3b**

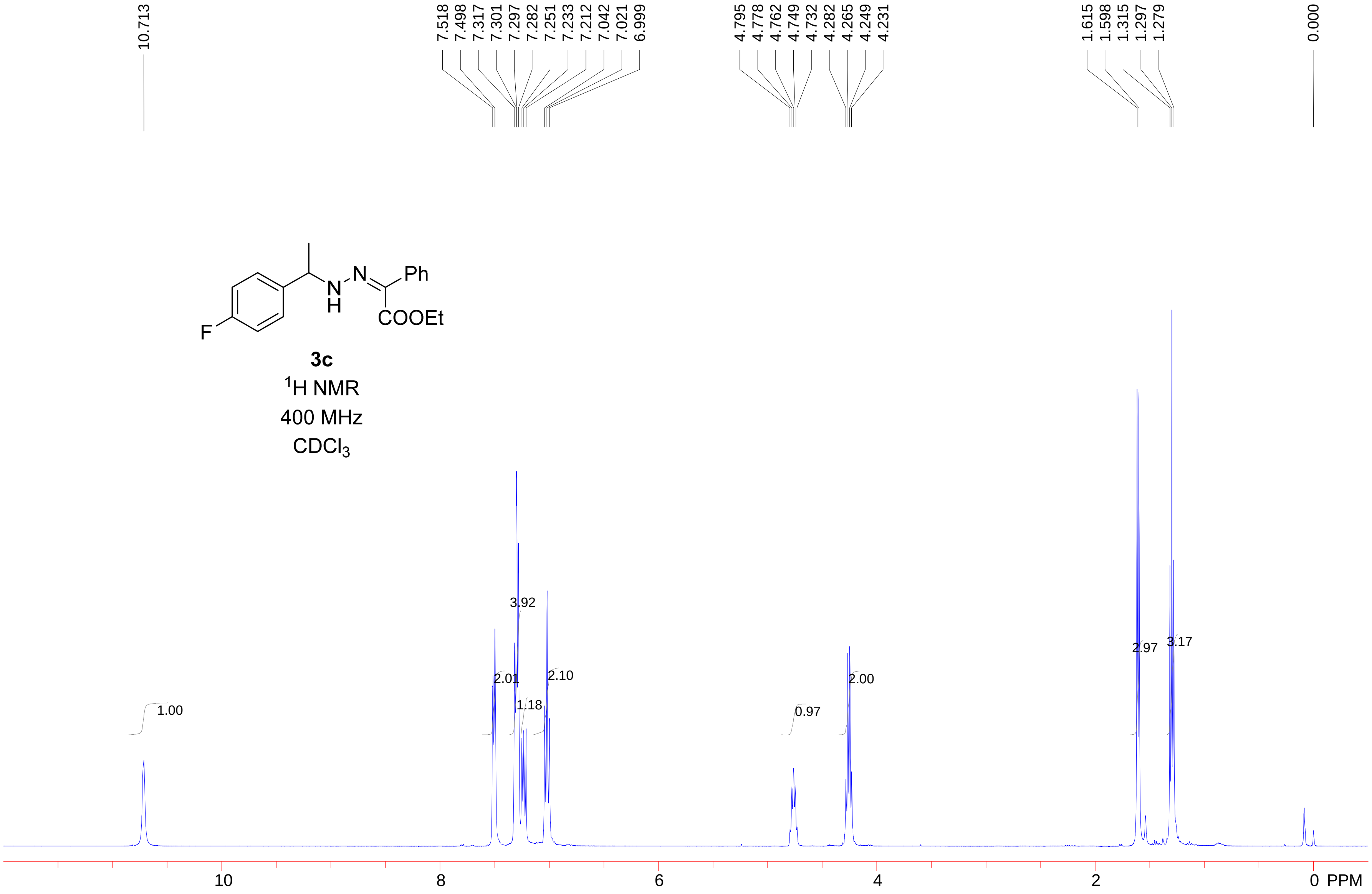

Supplementary Figure 15. ^{1}H NMR spectrum for **3c**

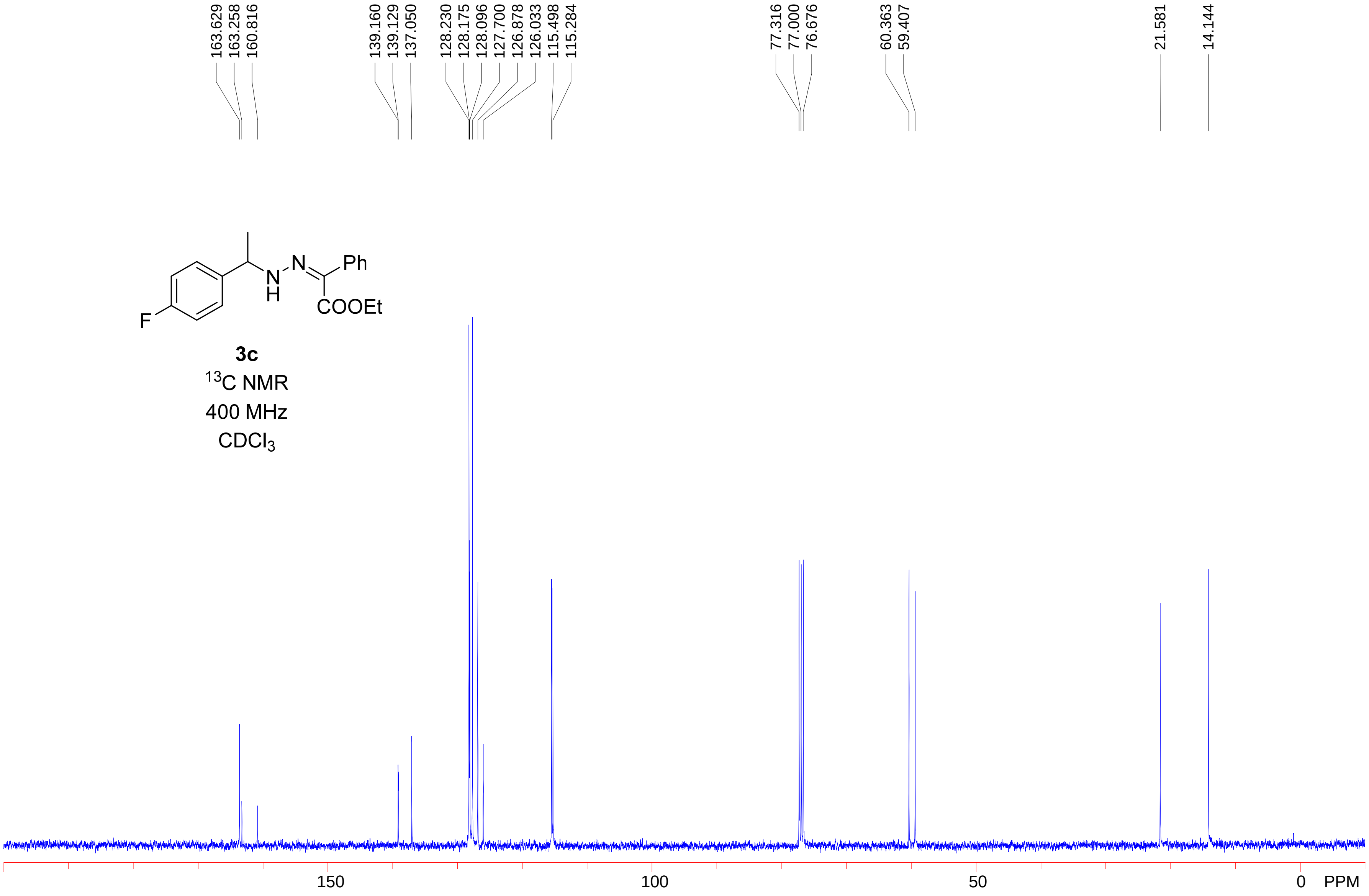

Supplementary Figure 16. ^{13}C NMR spectrum for **3c**

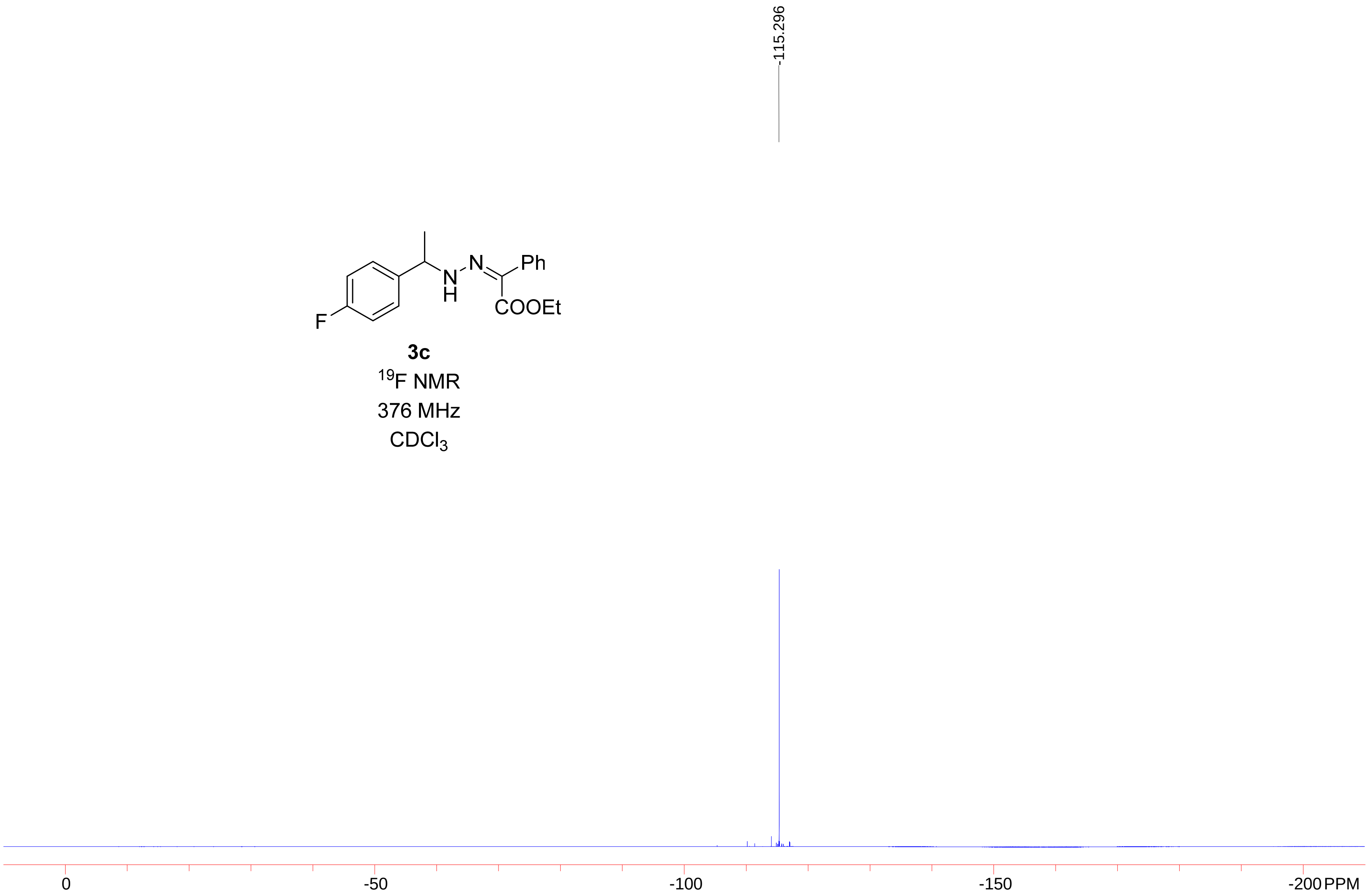

Supplementary Figure 17. ^{19}F NMR spectrum for **3c**

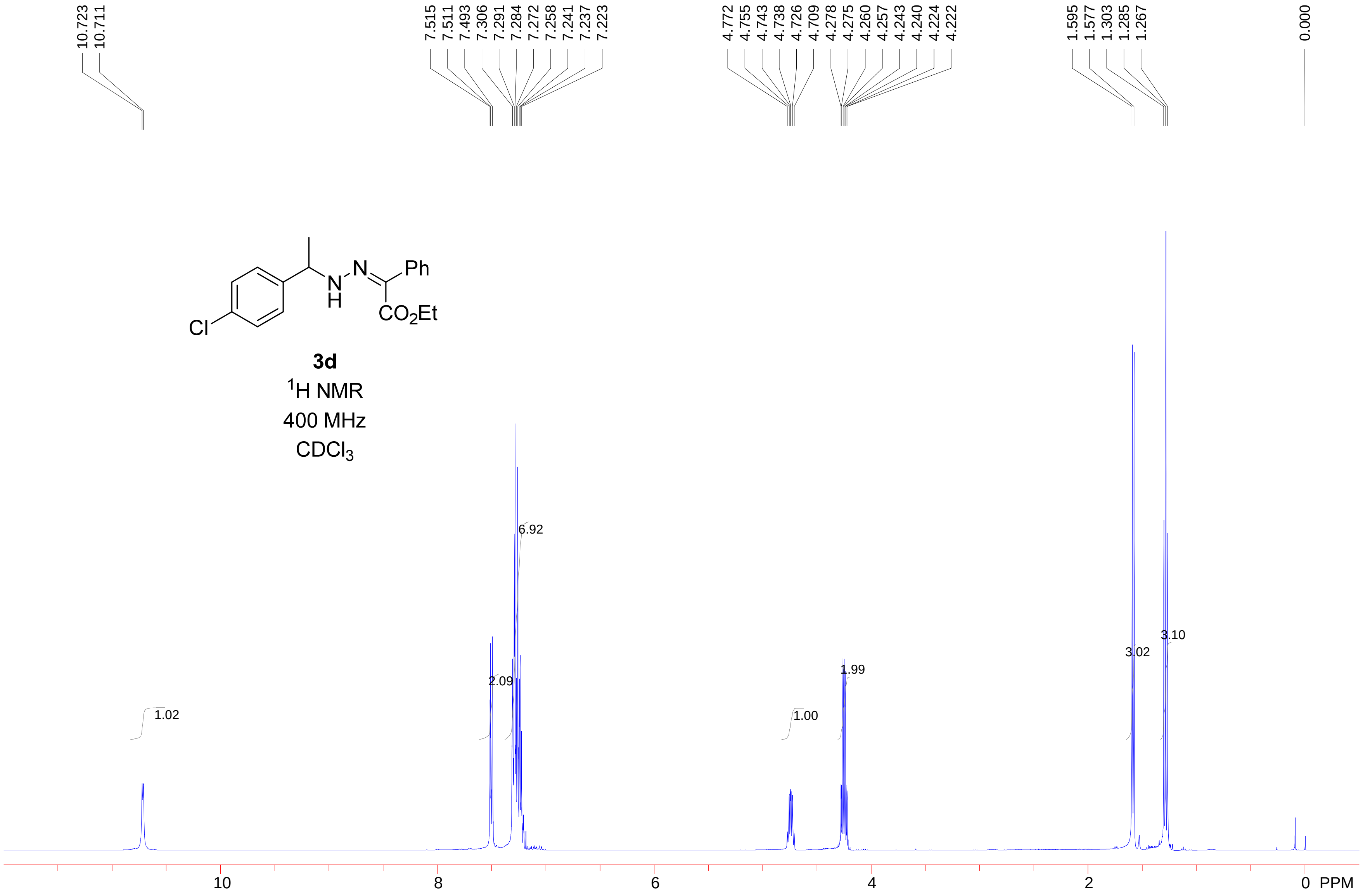

Supplementary Figure 18. ^{1}H NMR spectrum for **3d**

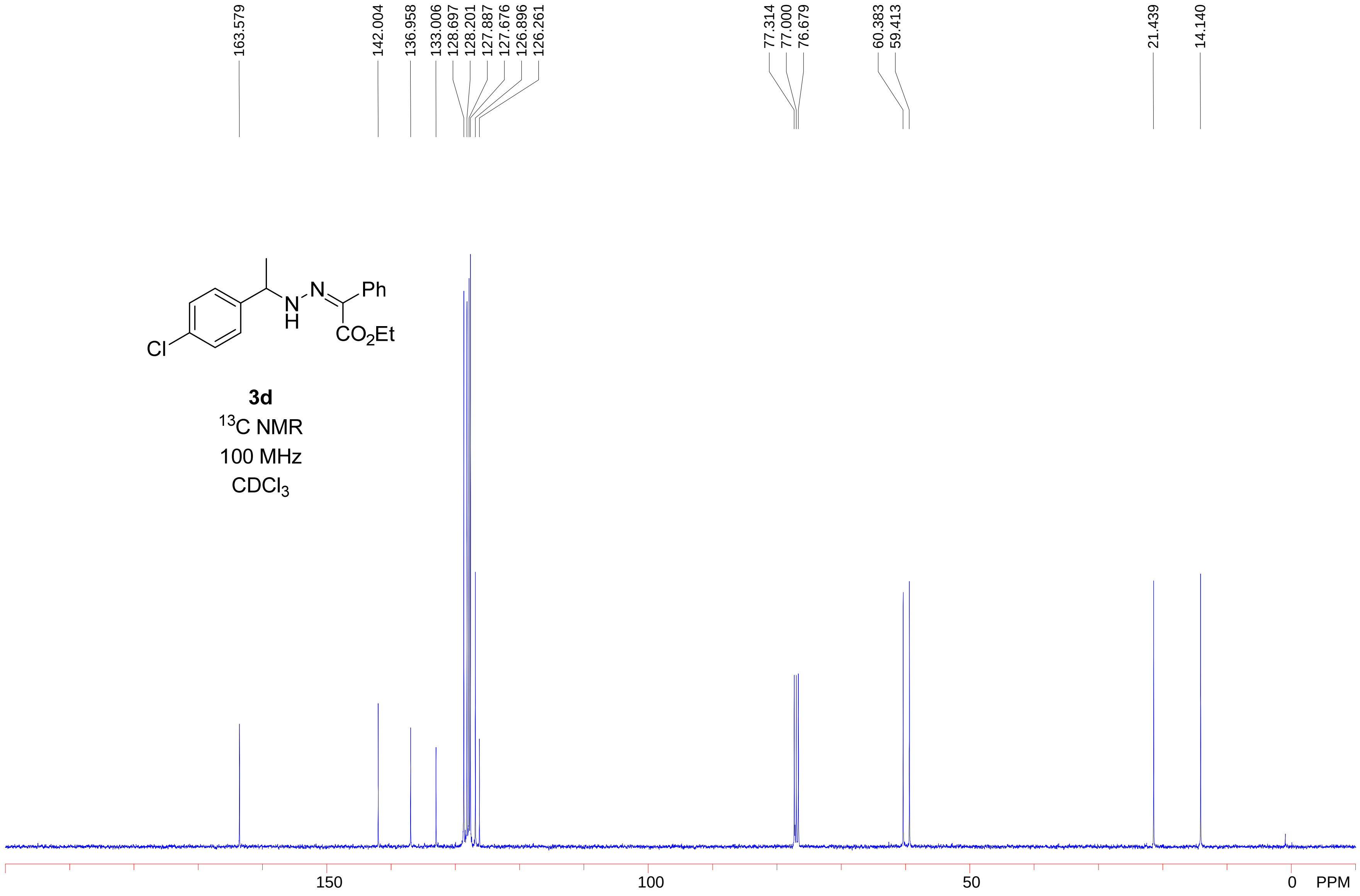

Supplementary Figure 19. ^{13}C NMR spectrum for **3d**

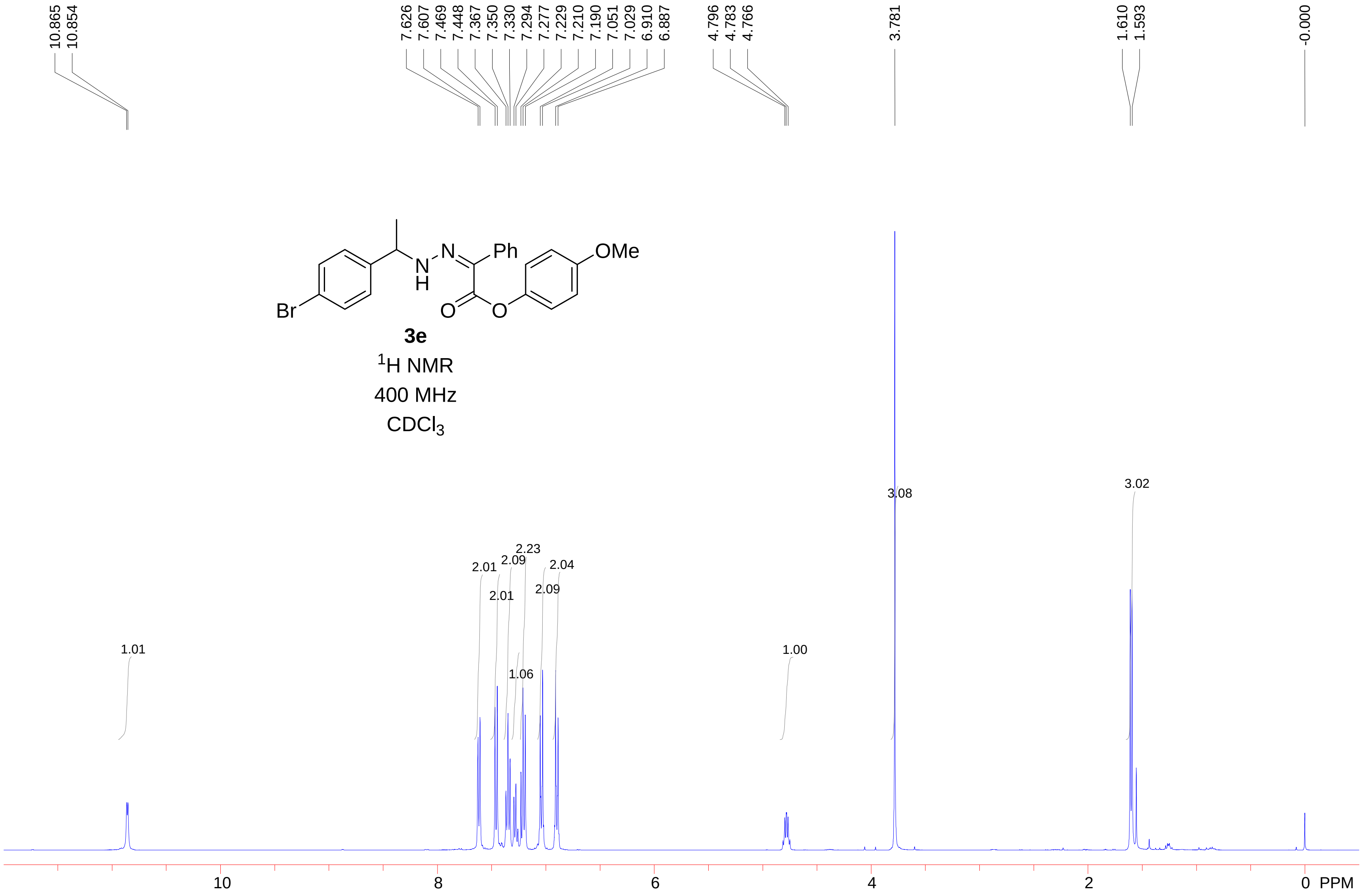

Supplementary Figure 20. ^{1}H NMR spectrum for **3e**

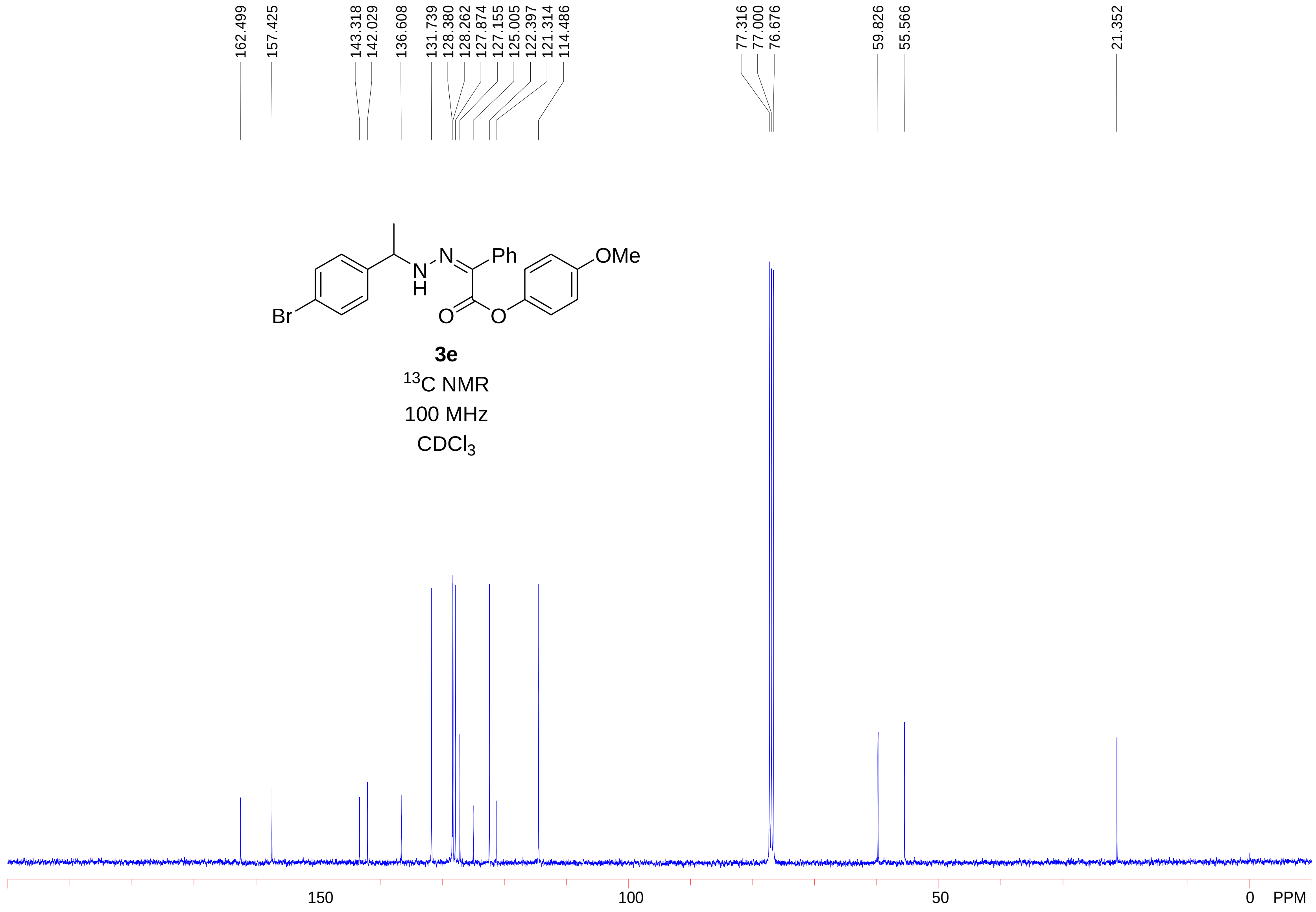

Supplementary Figure 21. ^{13}C NMR spectrum for **3e**

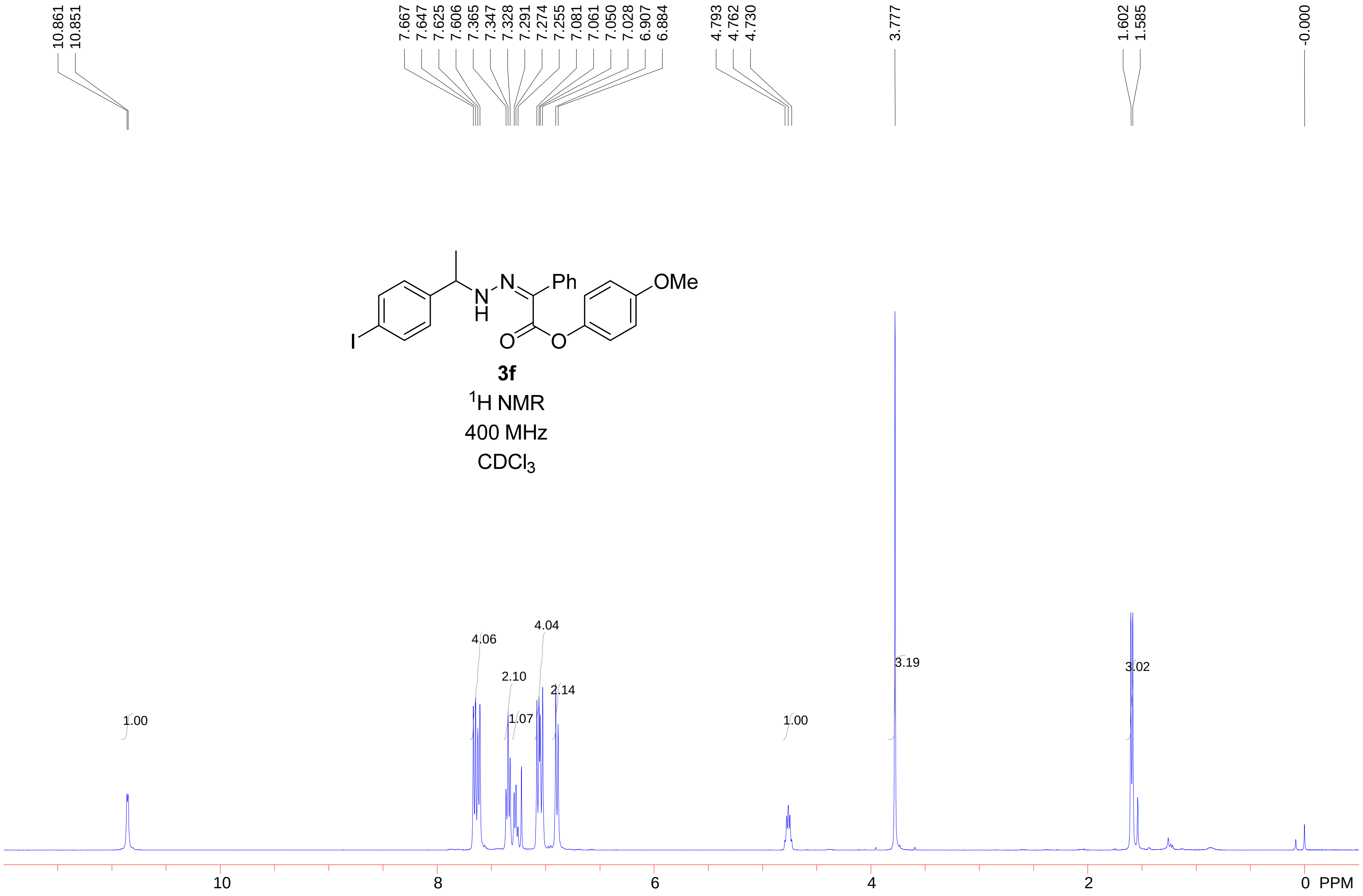

Supplementary Figure 22. ^{1}H NMR spectrum for **3f**

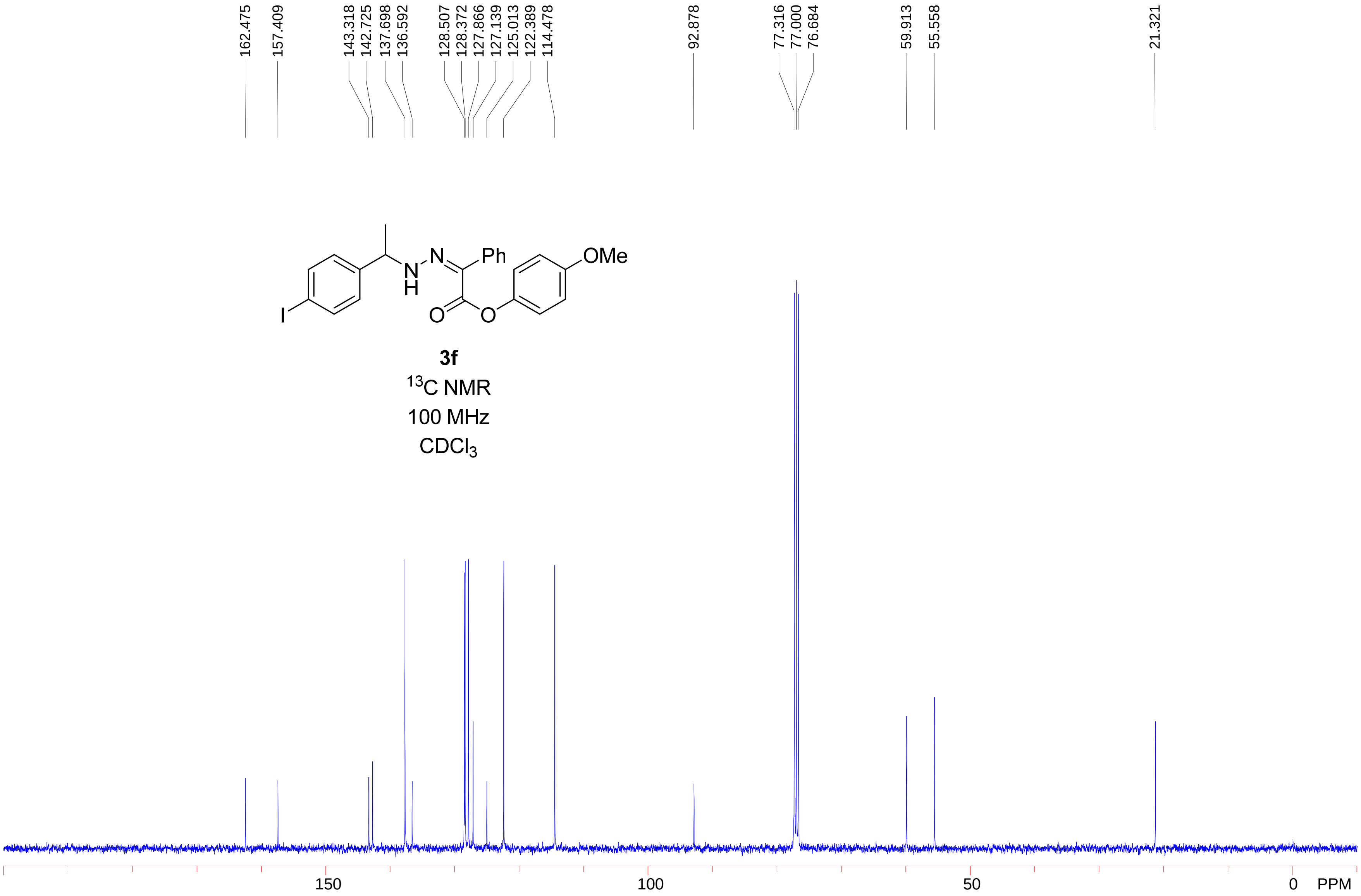

Supplementary Figure 23. ^{13}C NMR spectrum for **3f**

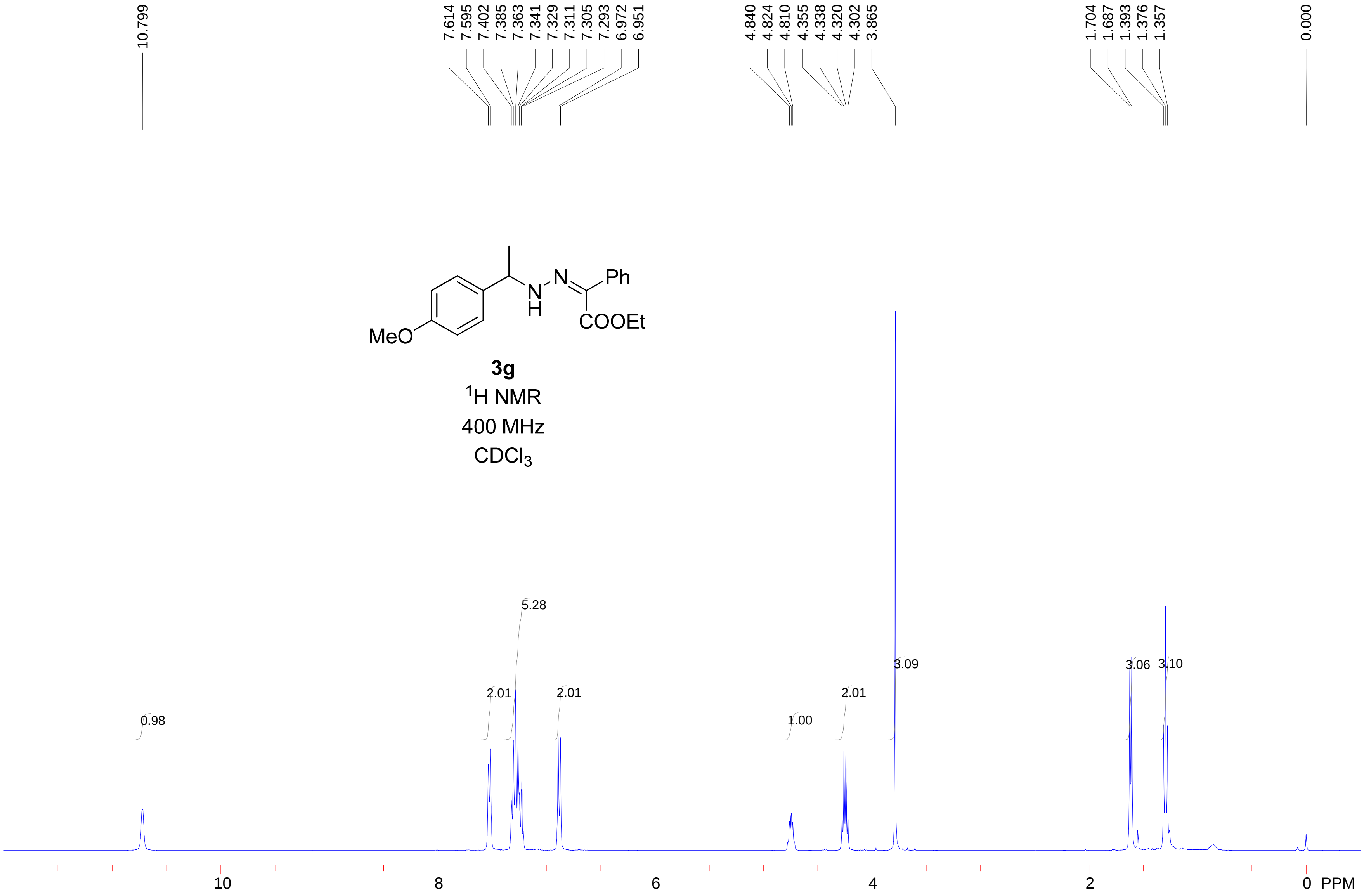

Supplementary Figure 24. ^{1}H NMR spectrum for **3g**

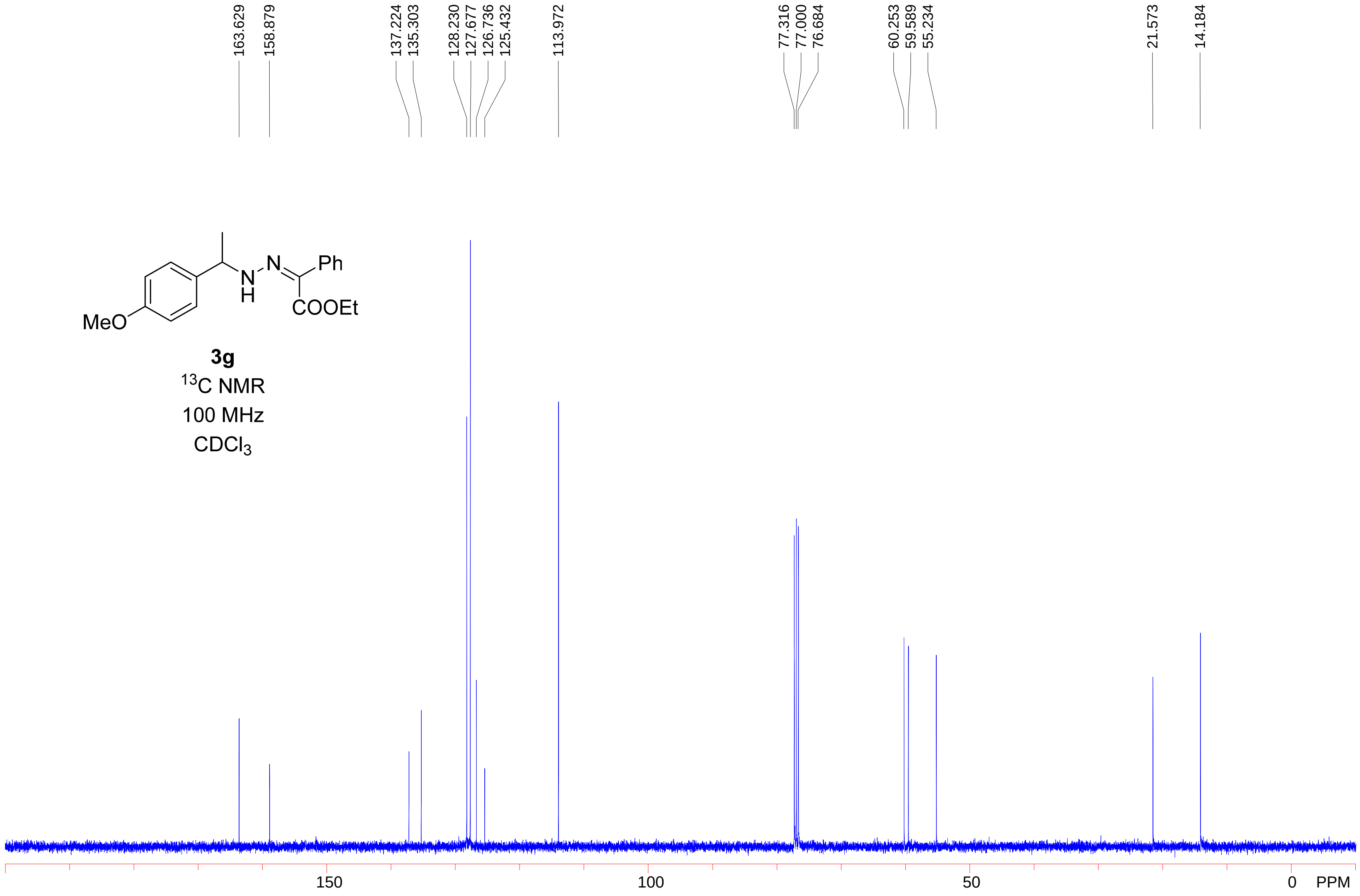

Supplementary Figure 25. ^{13}C NMR spectrum for **3g**

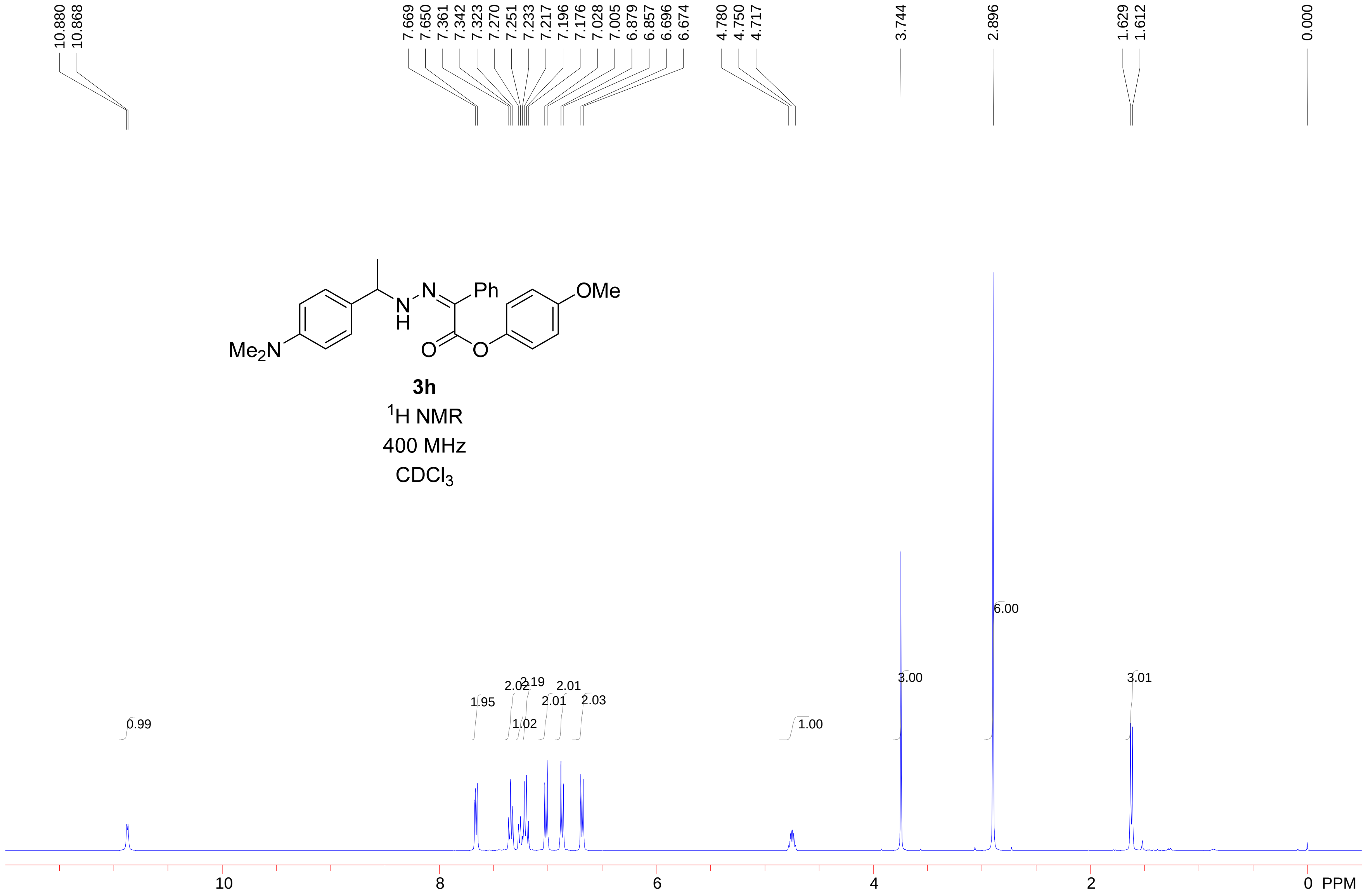

Supplementary Figure 26. ^{1}H NMR spectrum for **3h**

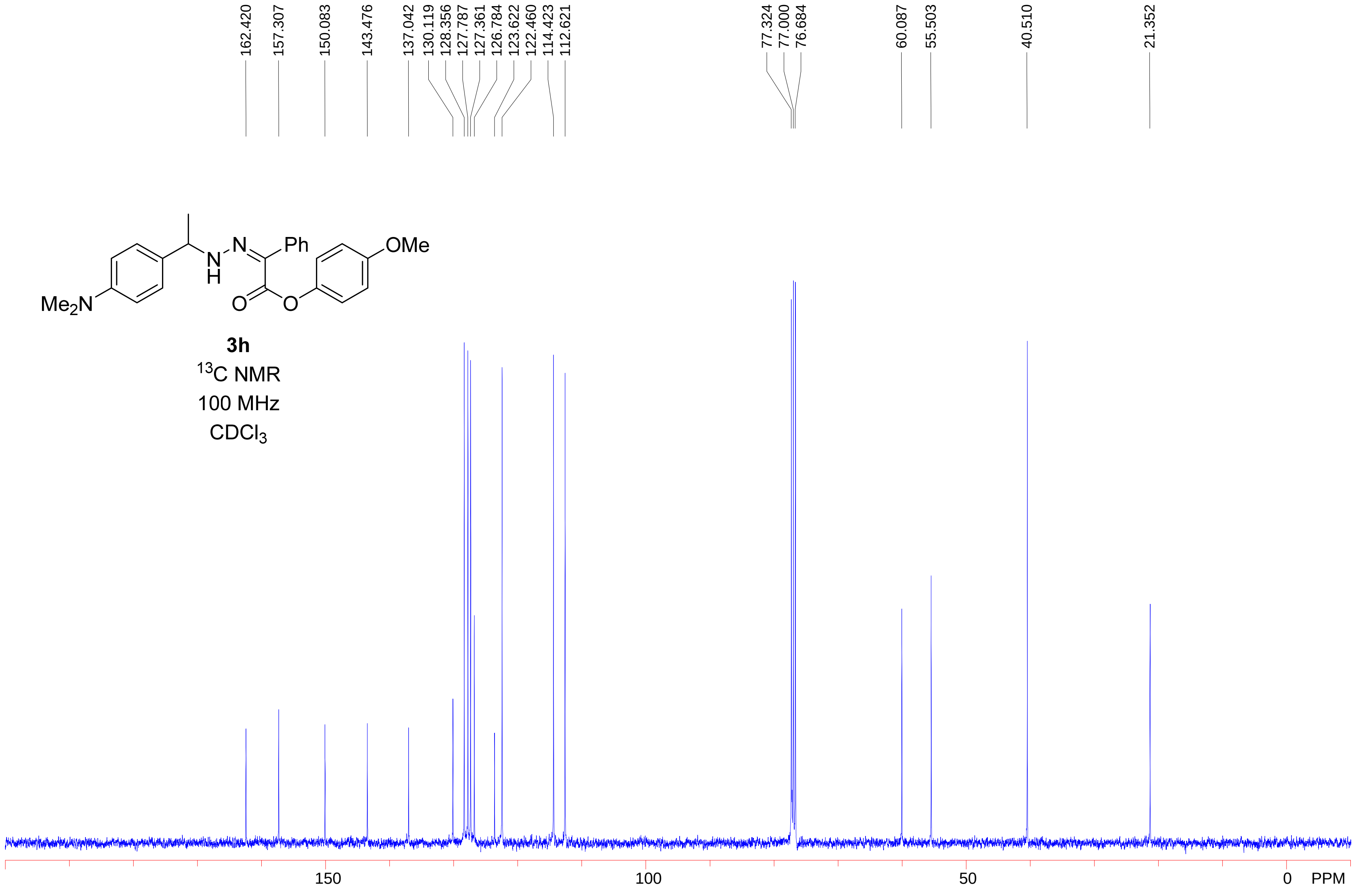

Supplementary Figure 27. ^{13}C NMR spectrum for **3h**

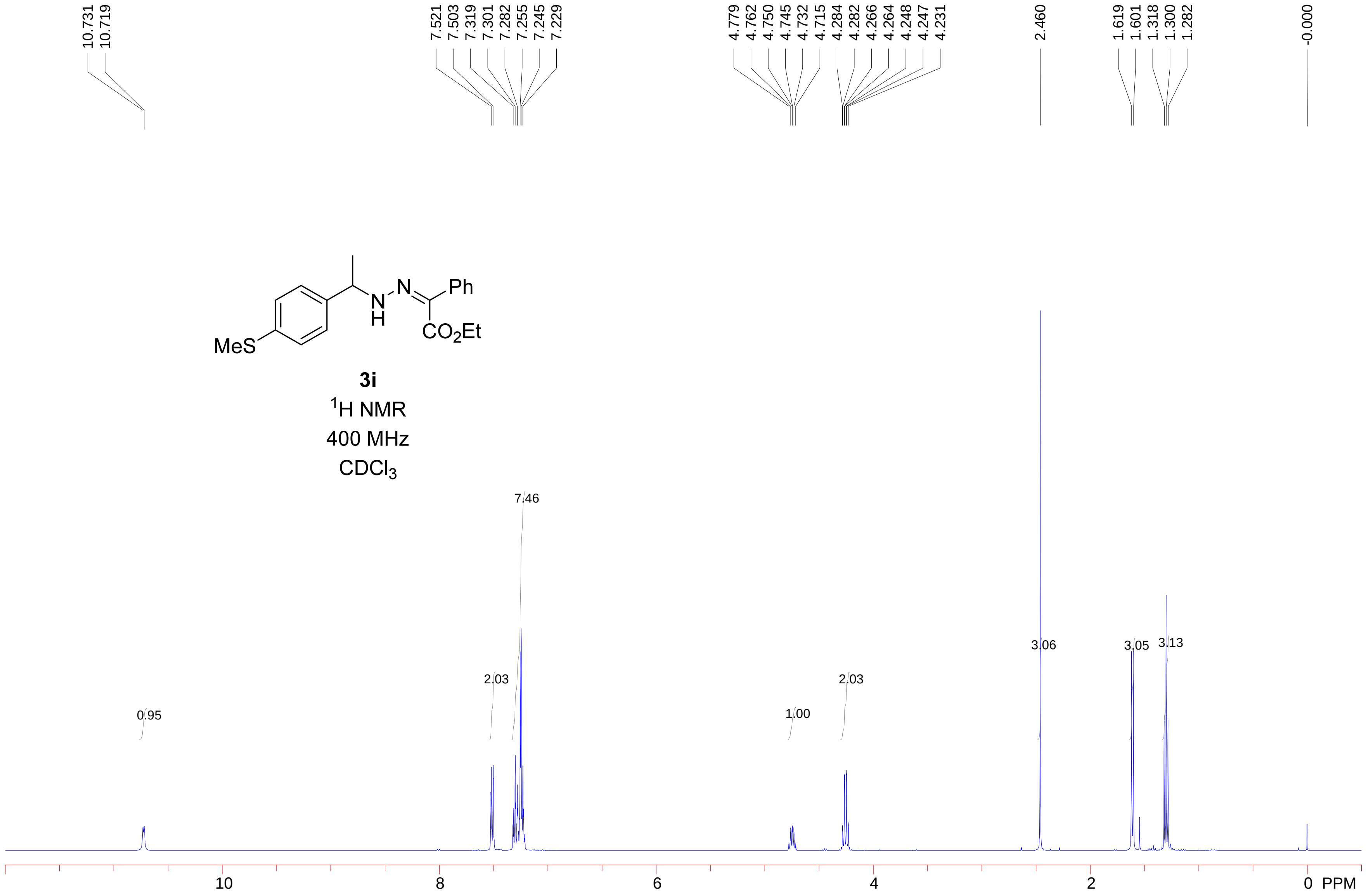

Supplementary Figure 28. ^{1}H NMR spectrum for **3i**

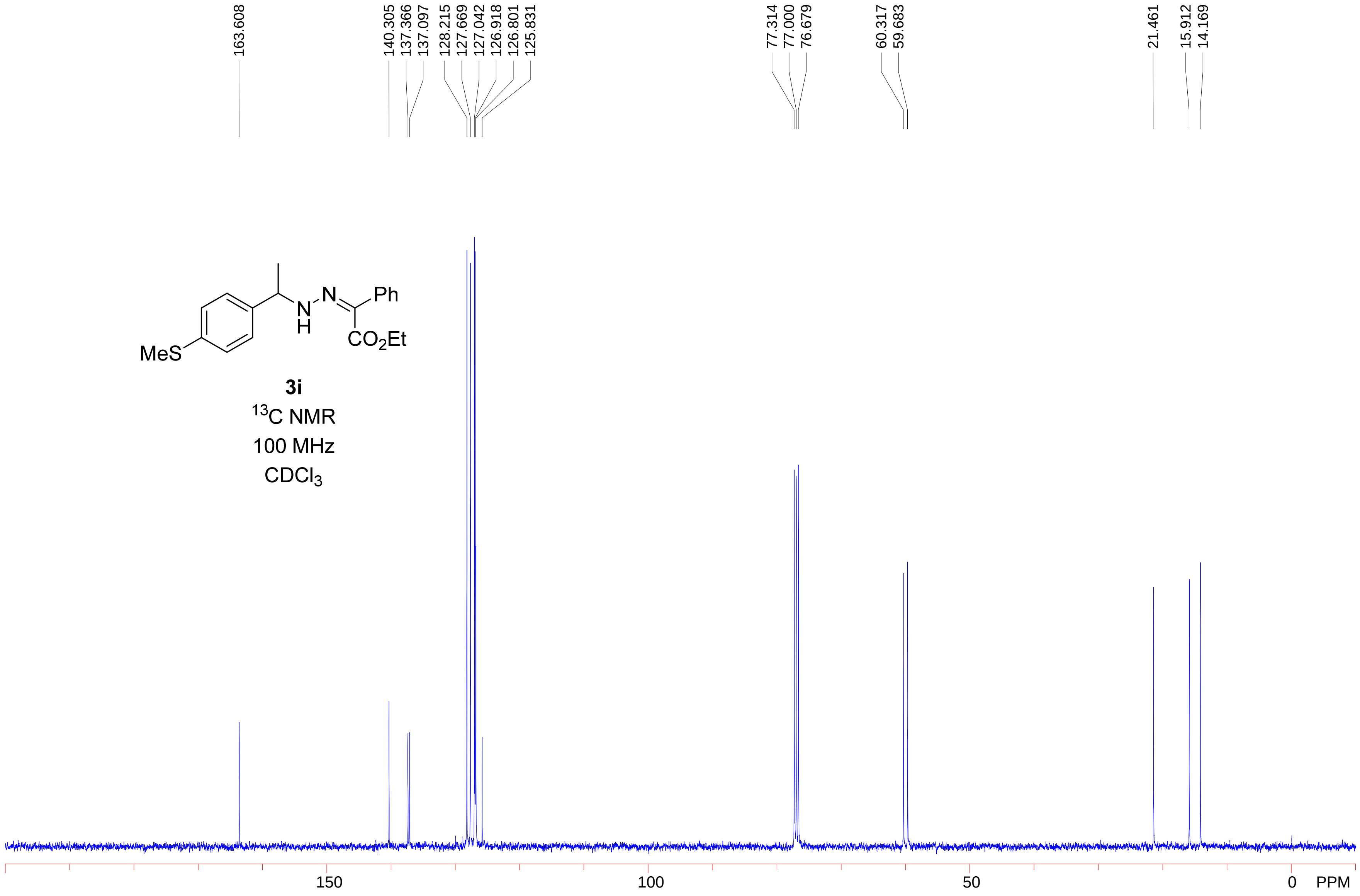

Supplementary Figure 29. ^{13}C NMR spectrum for **3i**

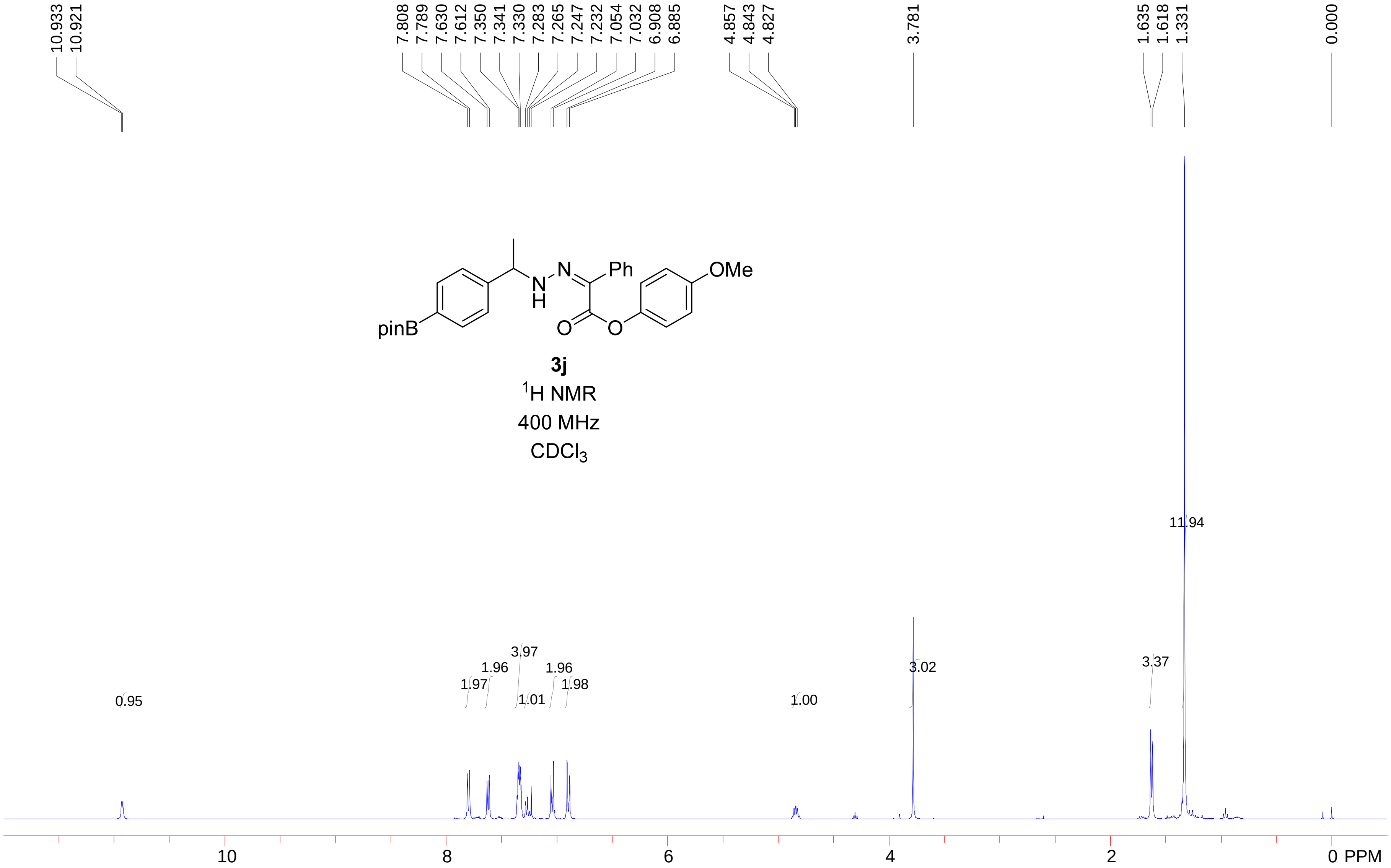

Supplementary Figure 30. ^{1}H NMR spectrum for **3j**

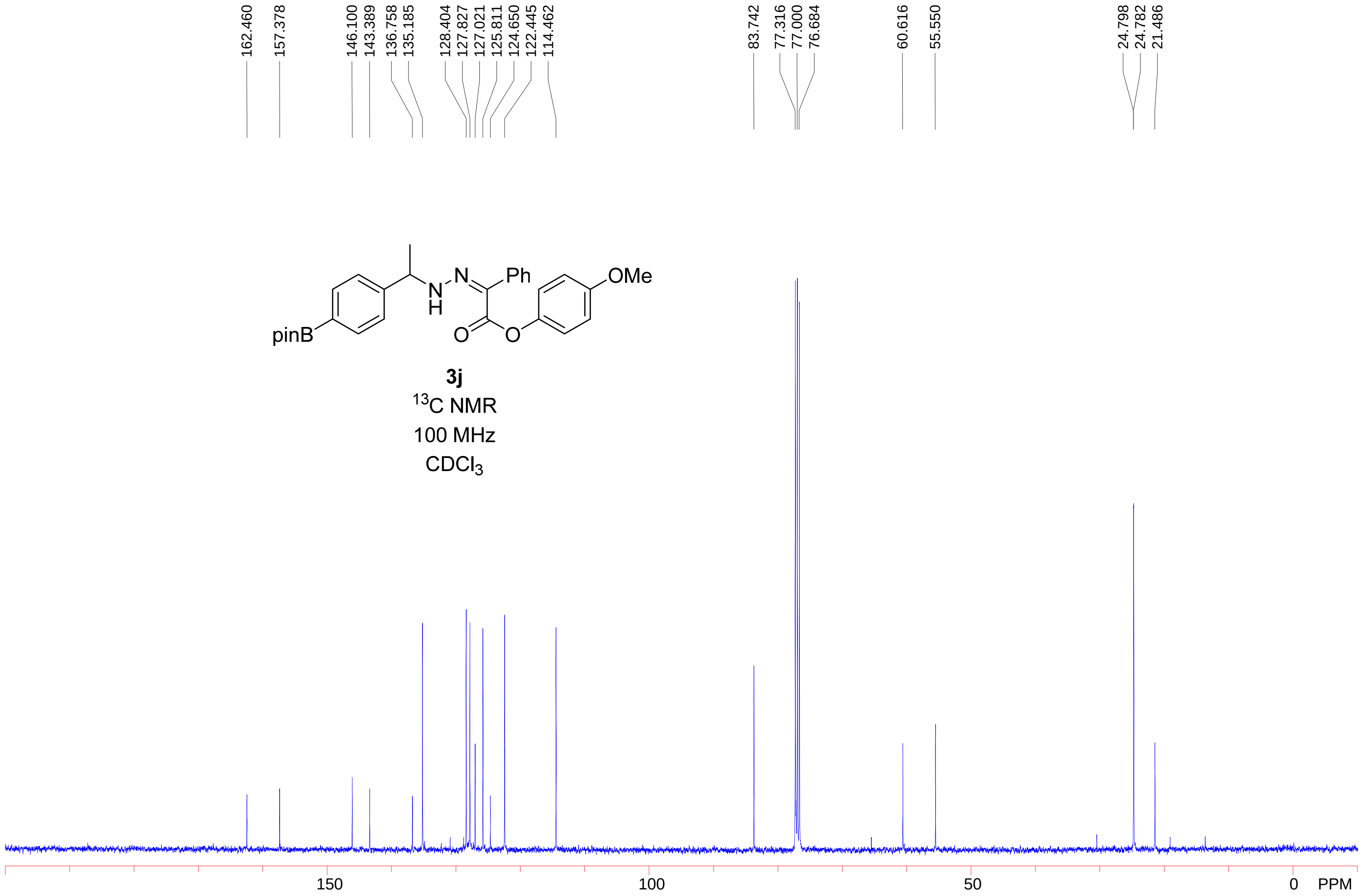

Supplementary Figure 31. ^{13}C NMR spectrum for **3j**

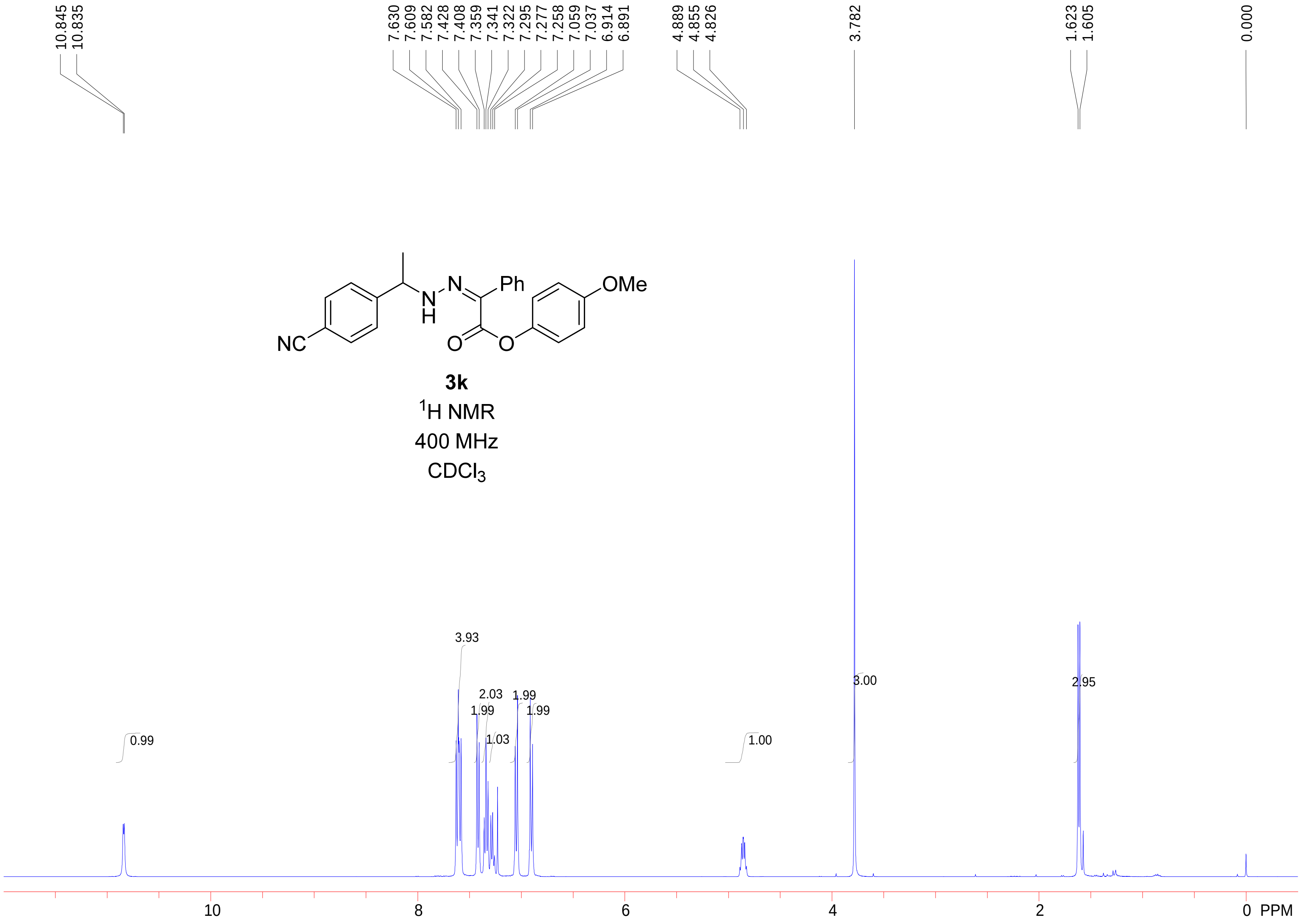

Supplementary Figure 32. ^{1}H NMR spectrum for **3k**

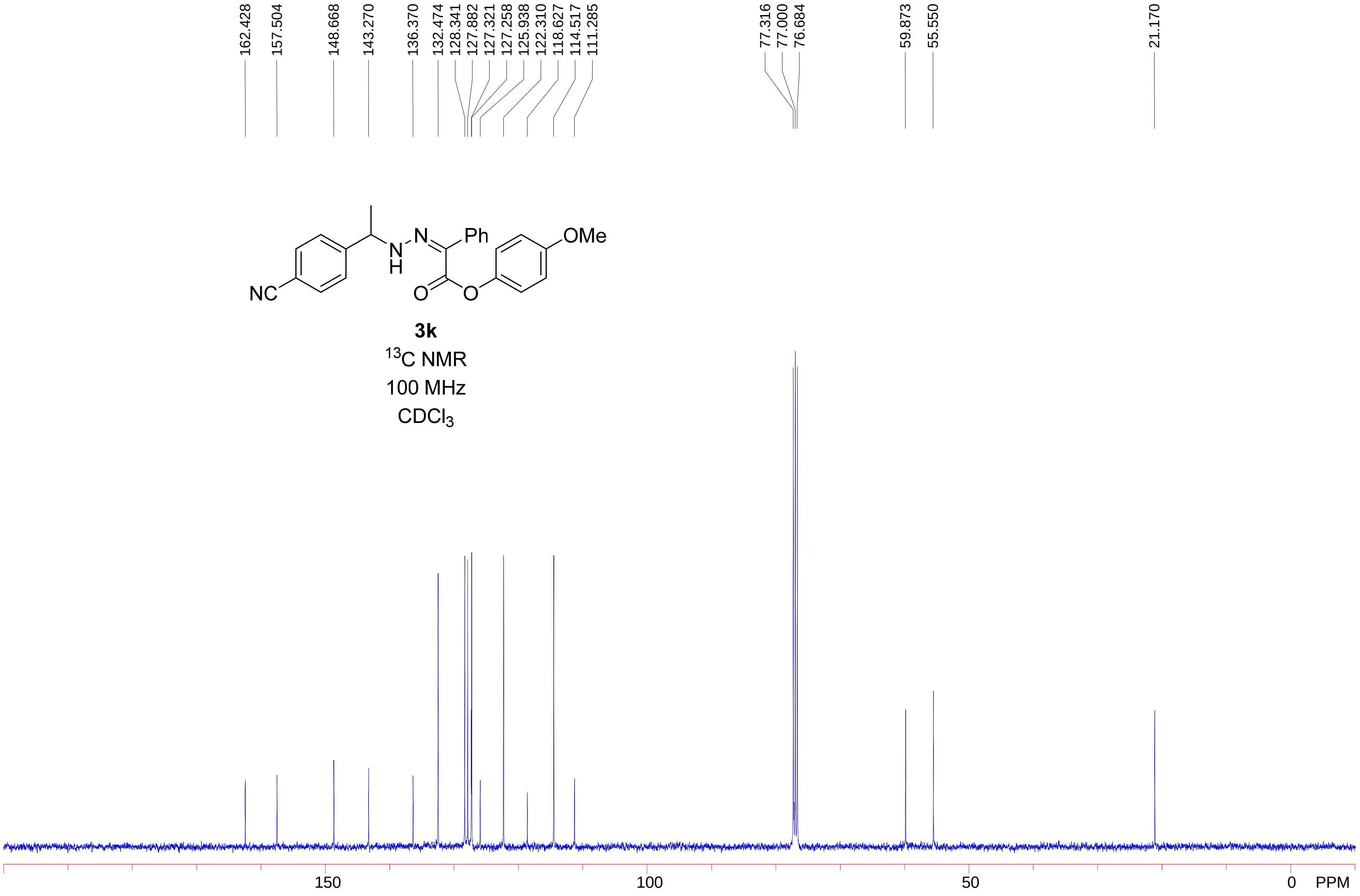

Supplementary Figure 33. ^{13}C NMR spectrum for **3k**

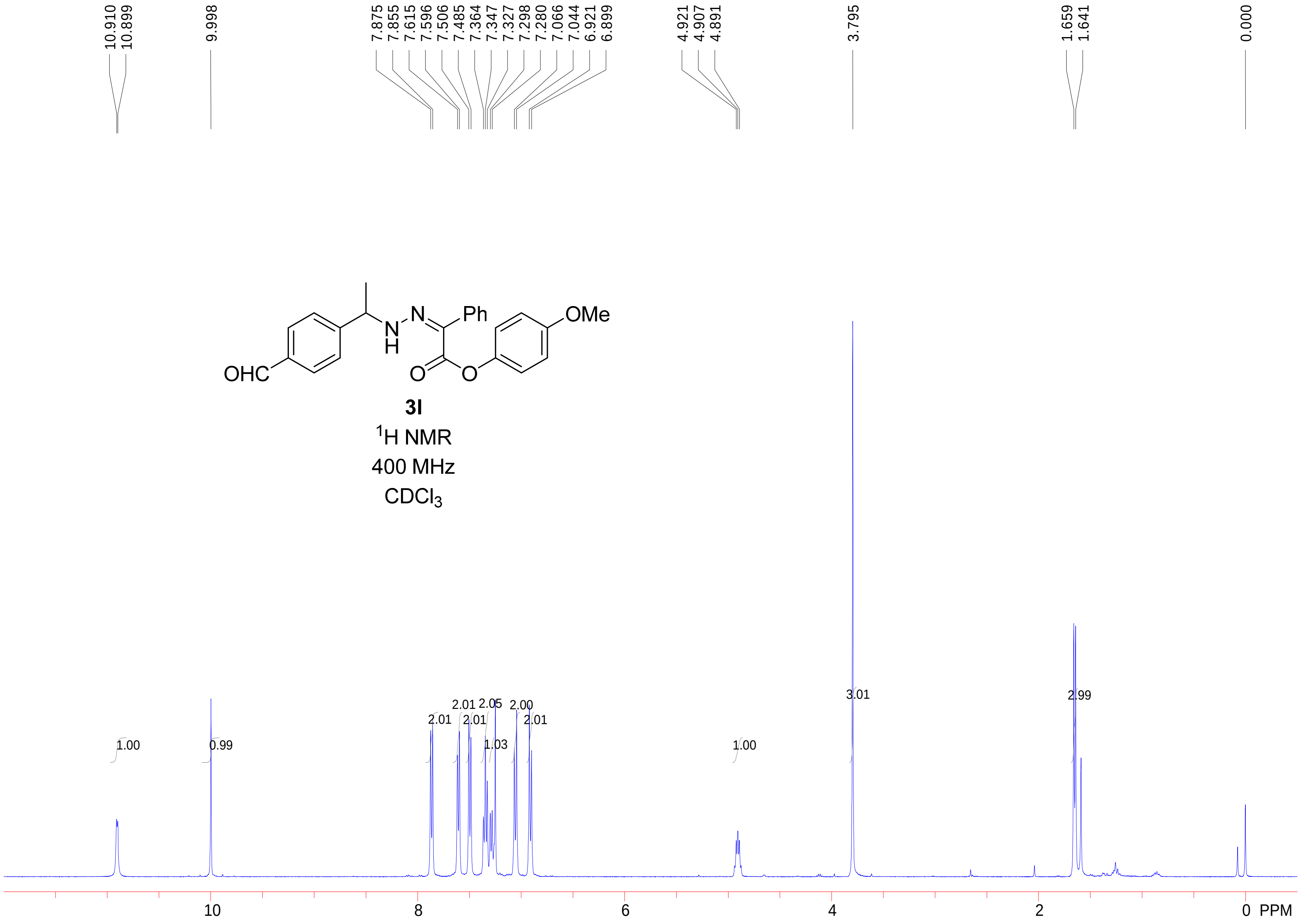

Supplementary Figure 34. ^{1}H NMR spectrum for **3l**

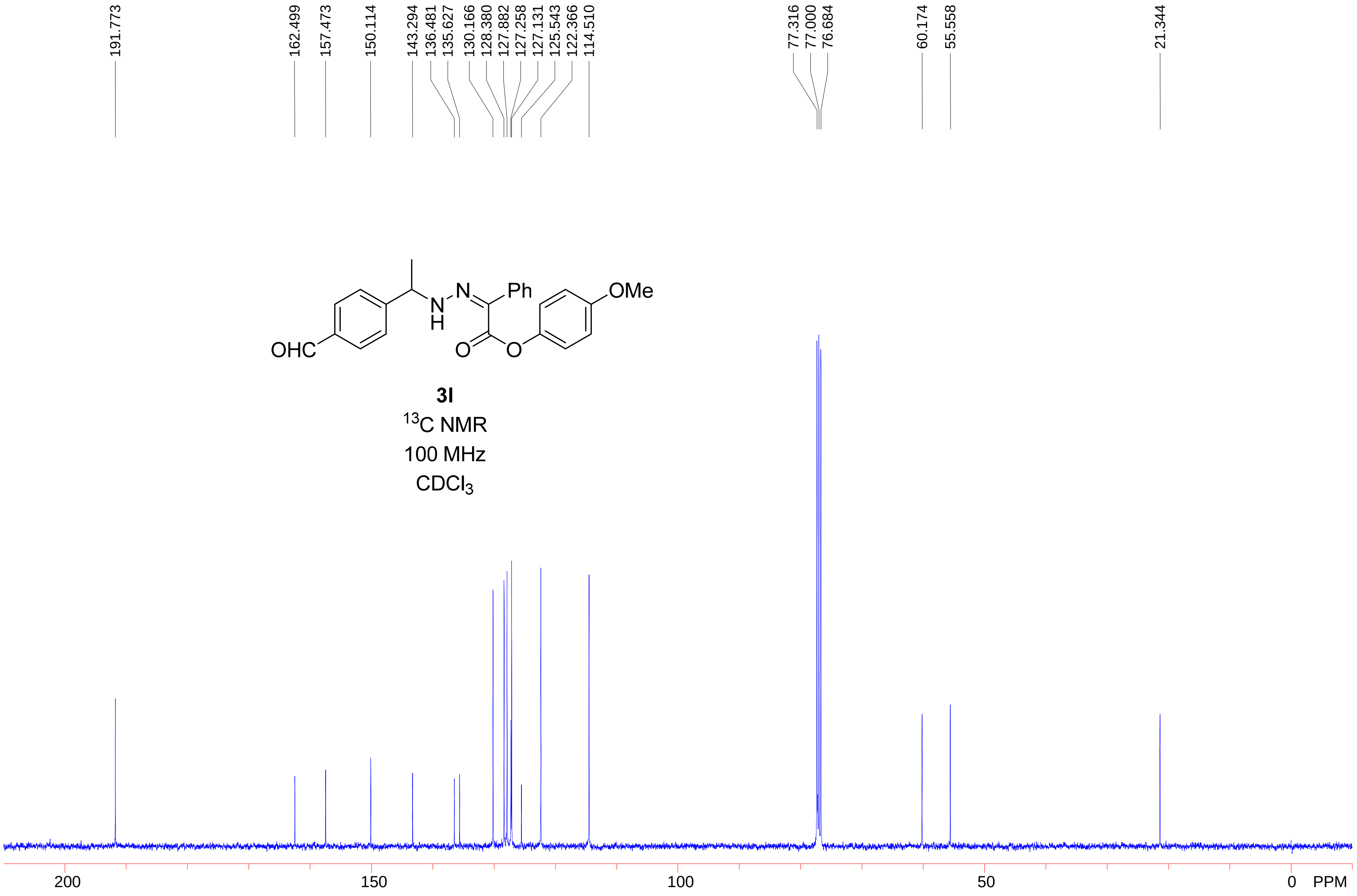

Supplementary Figure 35. ^{13}C NMR spectrum for **3l**

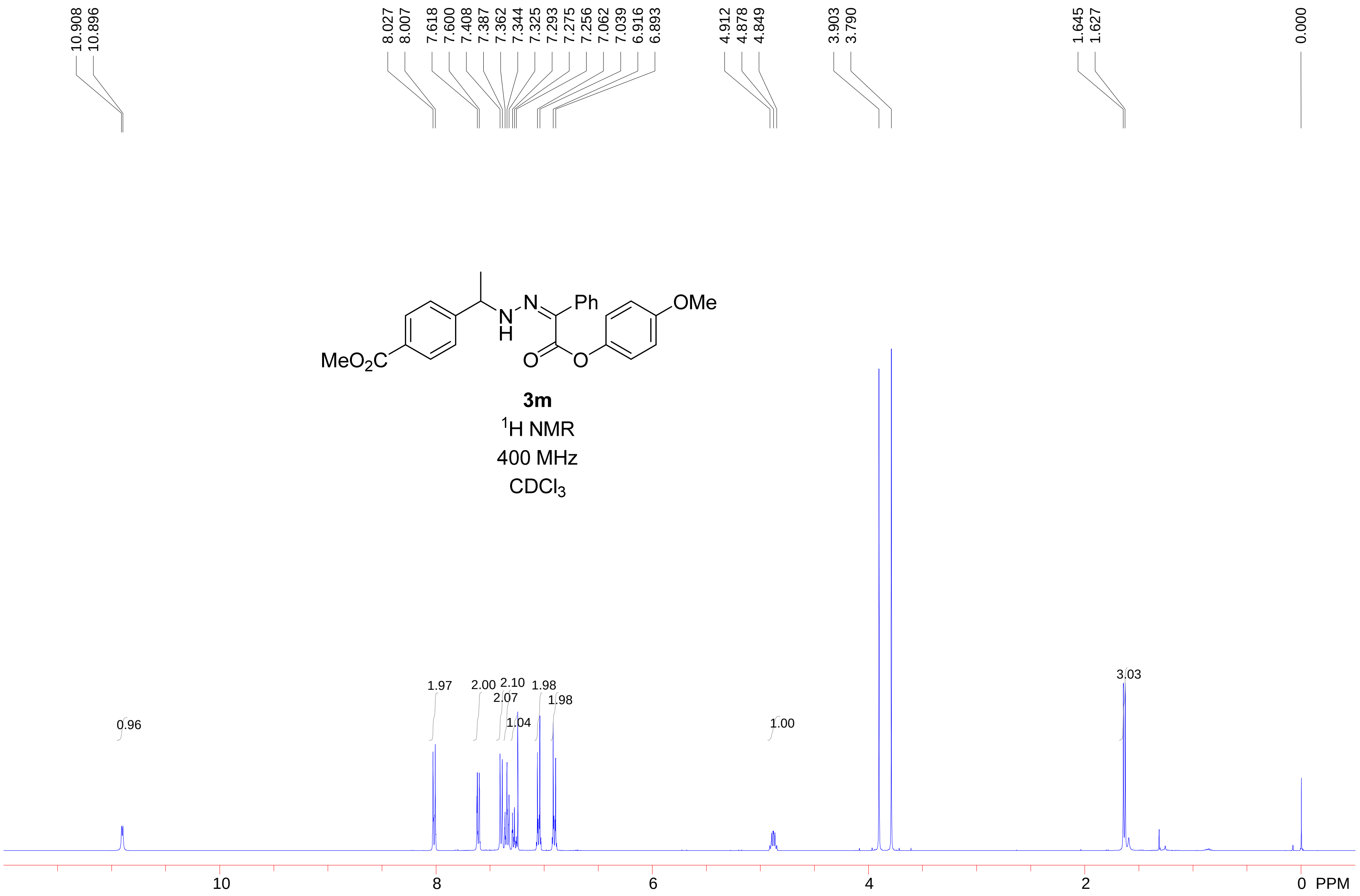

Supplementary Figure 36. ^{1}H NMR spectrum for **3m**

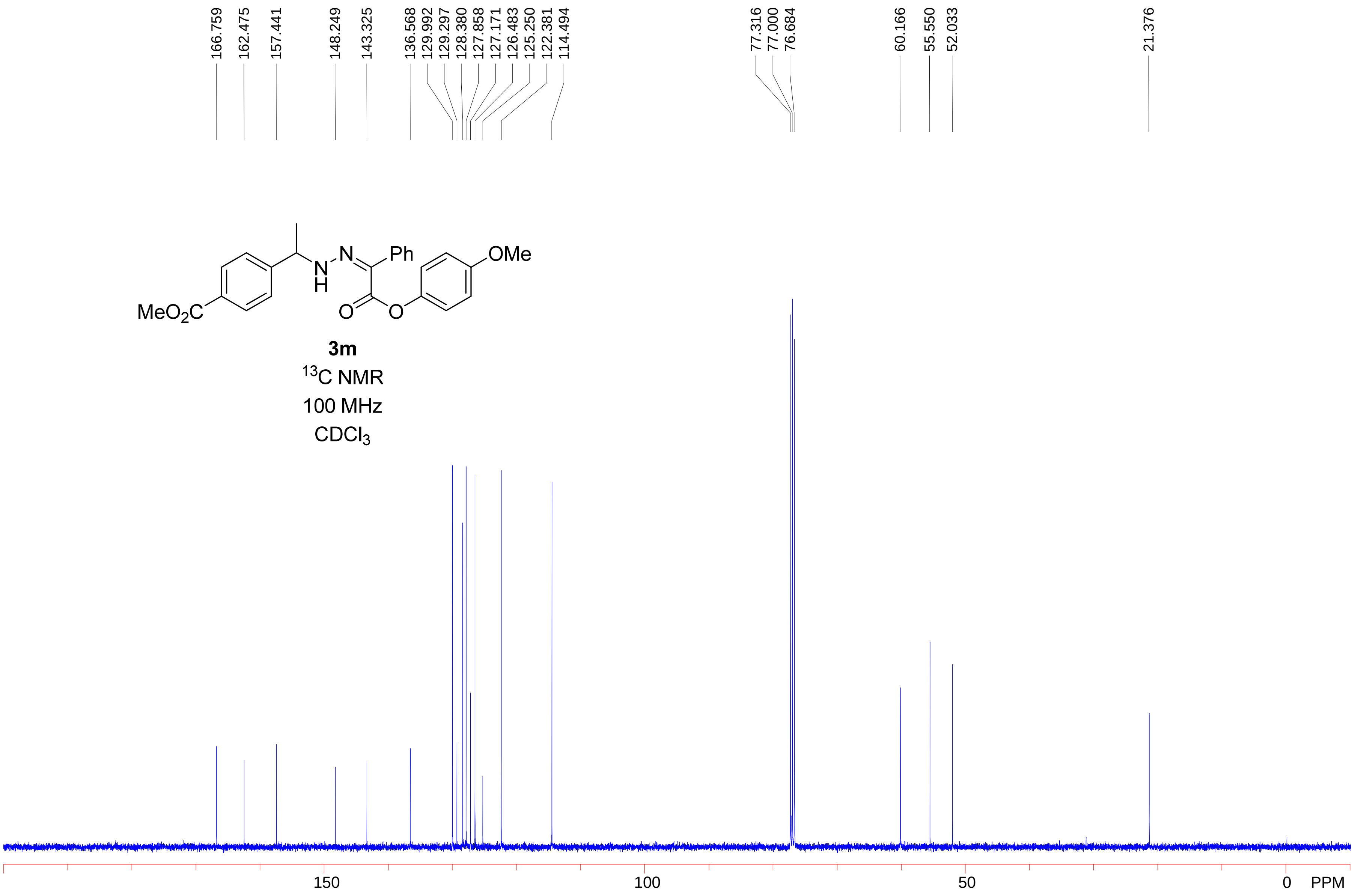

Supplementary Figure 37. ^{13}C NMR spectrum for **3m**

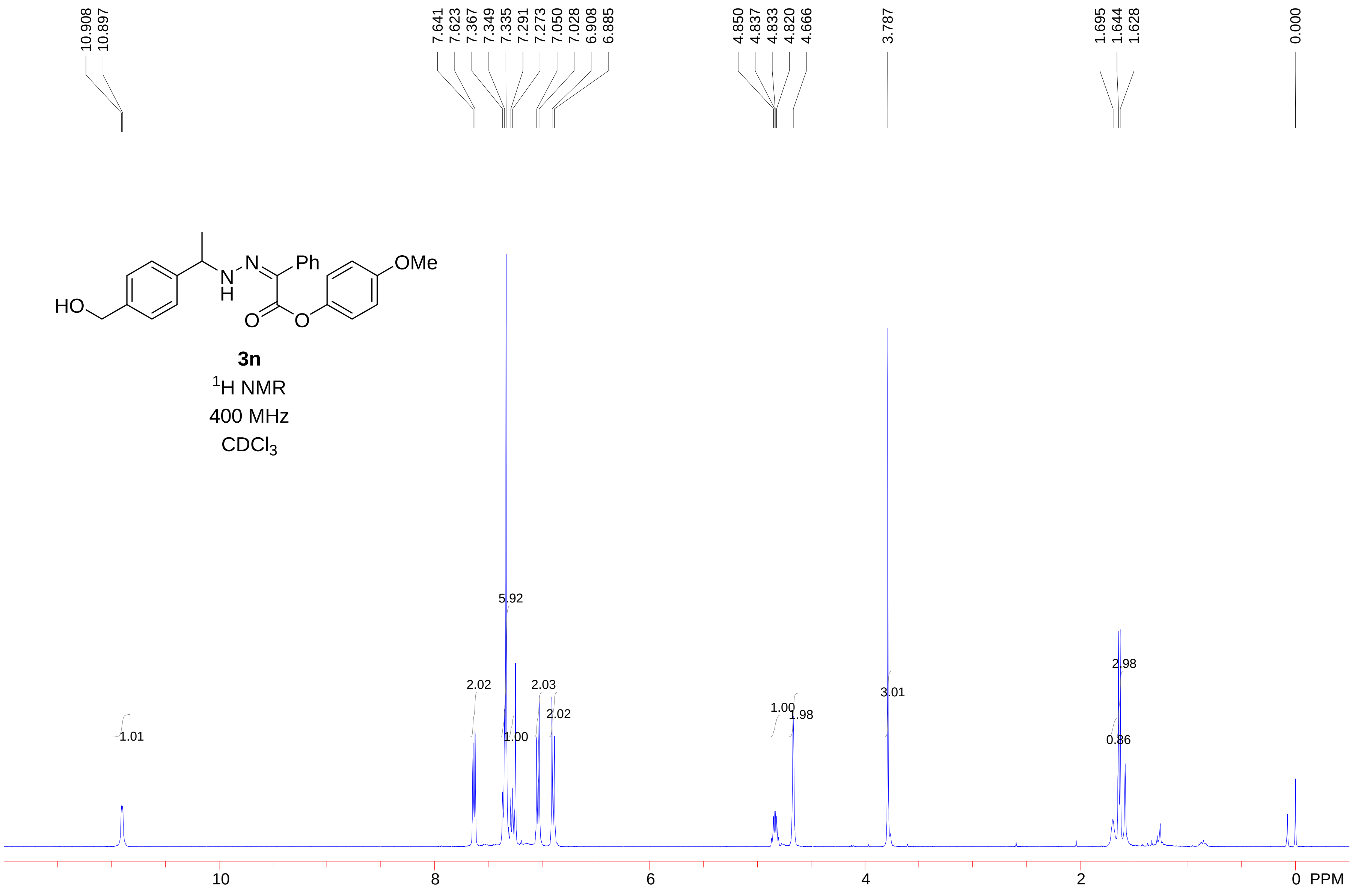

Supplementary Figure 38. ^{1}H NMR spectrum for **3n**

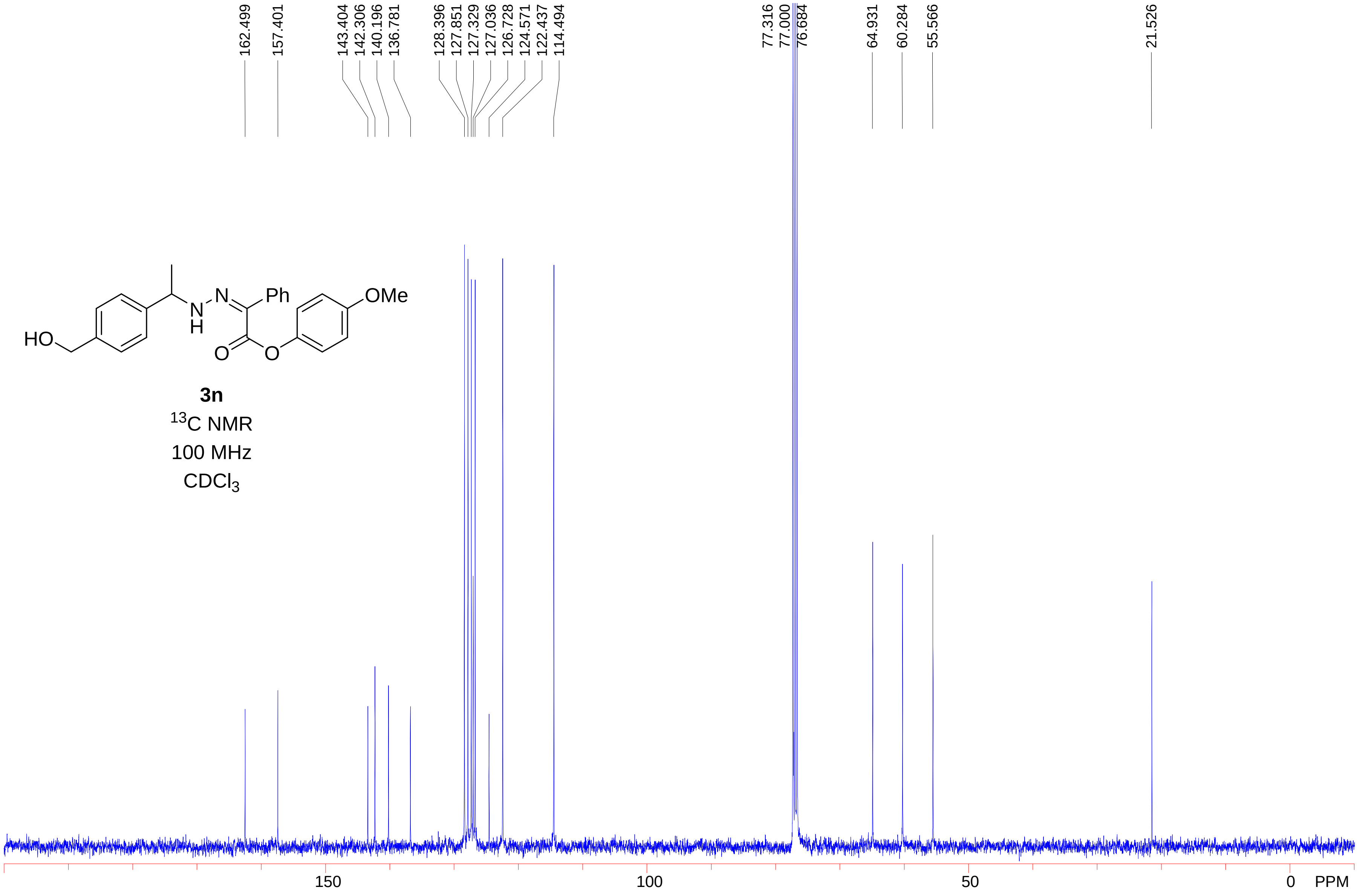

Supplementary Figure 39. ^{13}C NMR spectrum for 3n

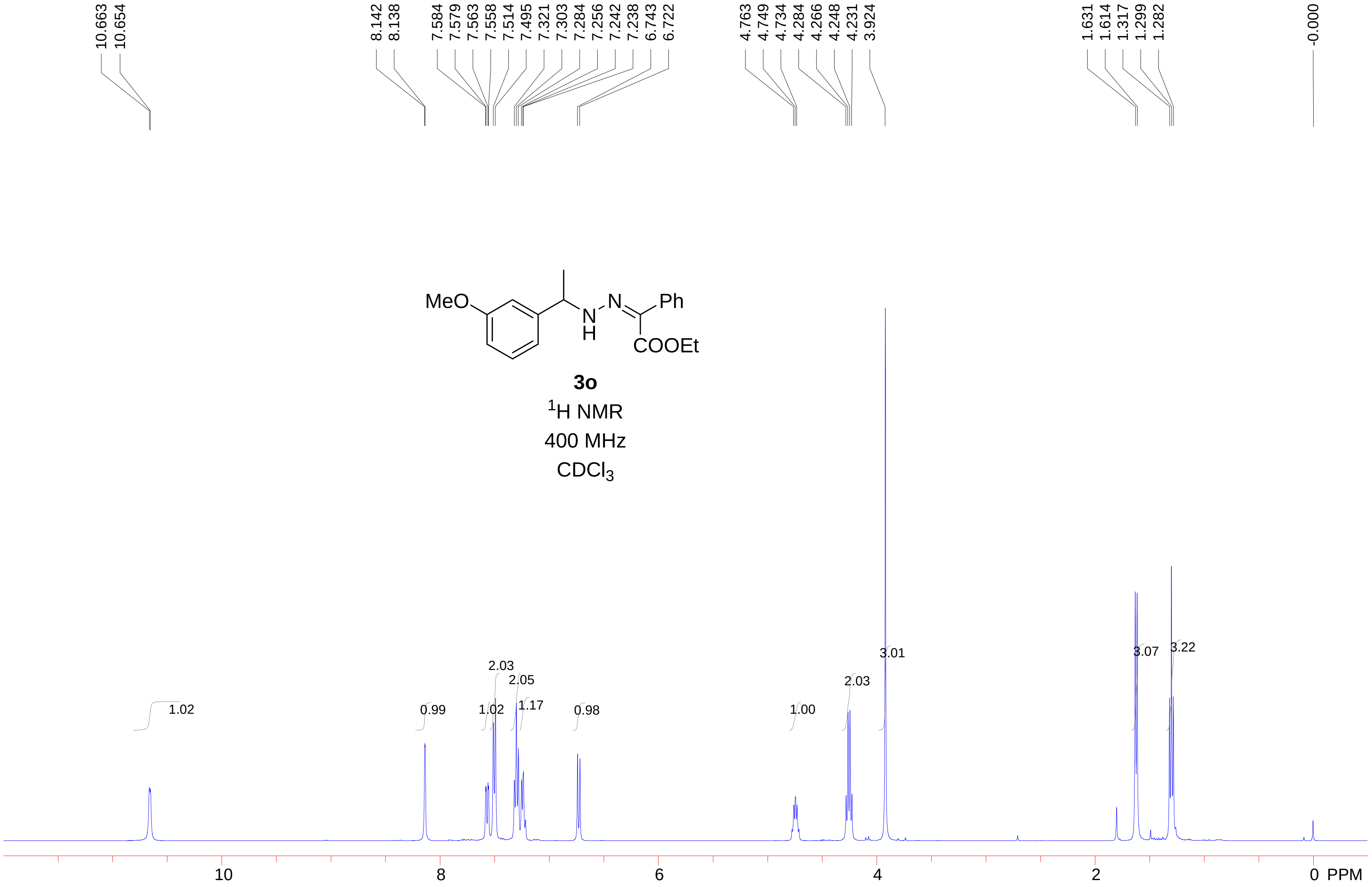

Supplementary Figure 40. ^{1}H NMR spectrum for **3o**

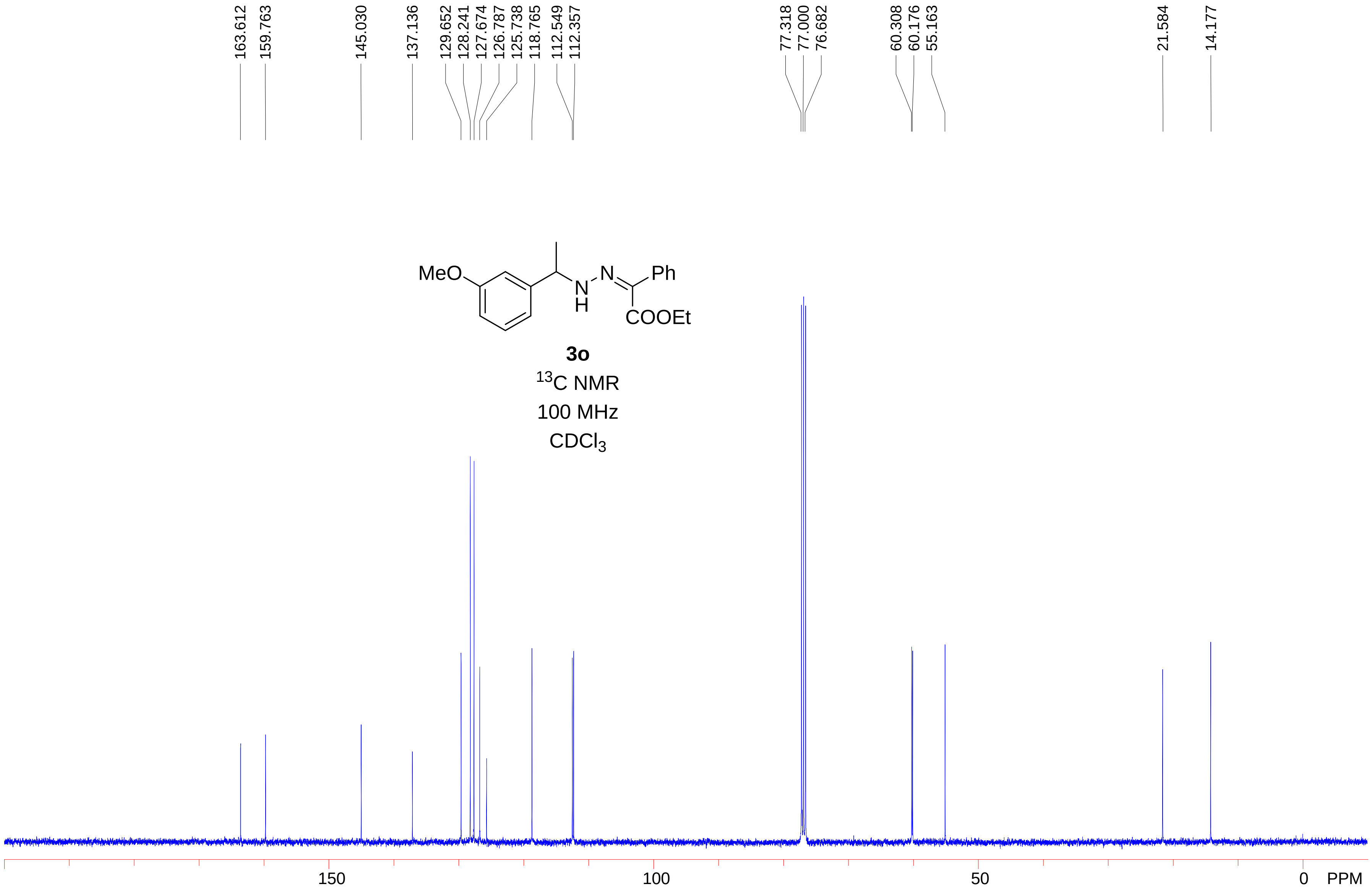

Supplementary Figure 41. ^{13}C NMR spectrum for **3o**

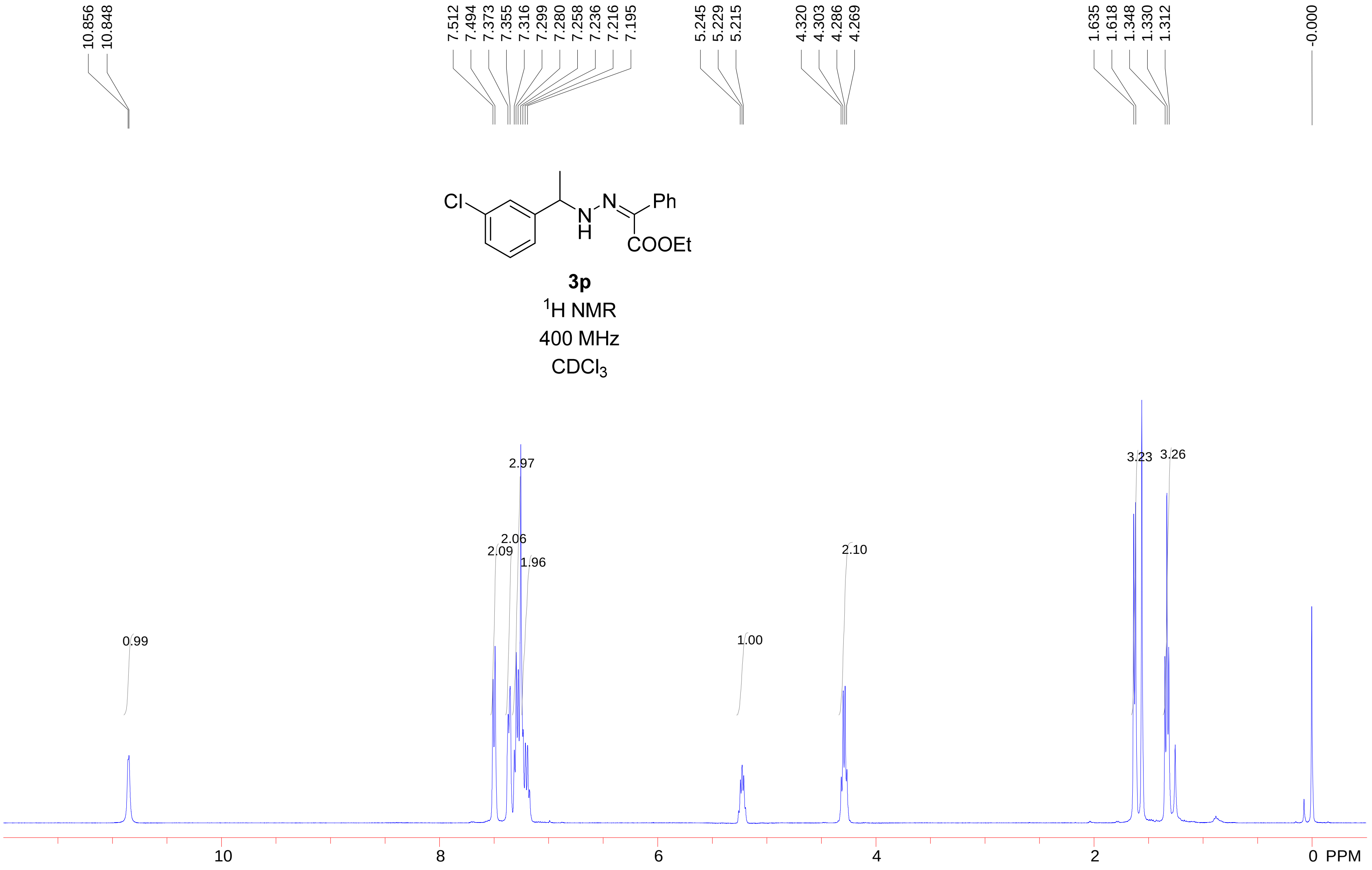

Supplementary Figure 42. ^{1}H NMR spectrum for **3p**

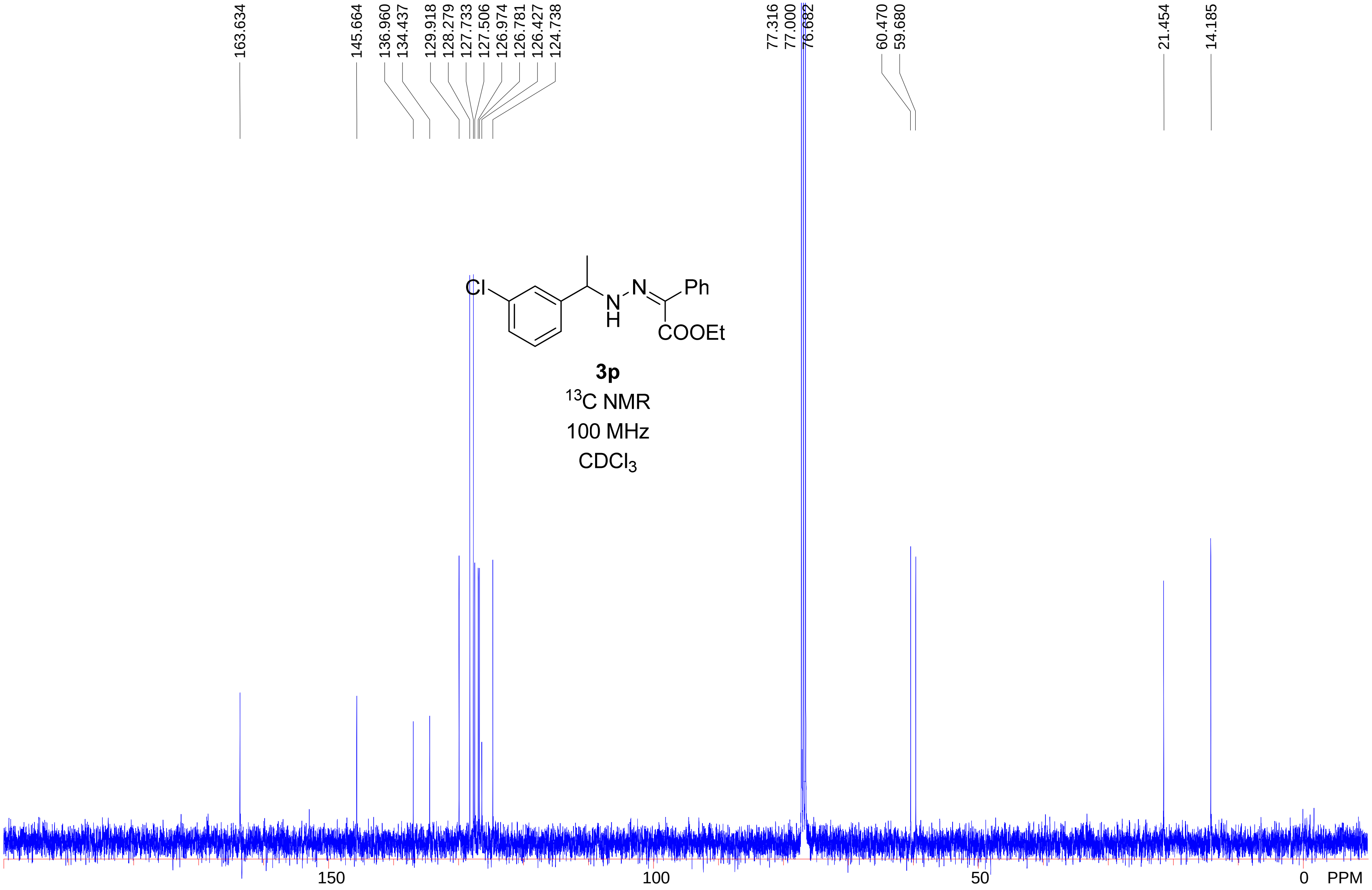

Supplementary Figure 43. ^{13}C NMR spectrum for **3p**

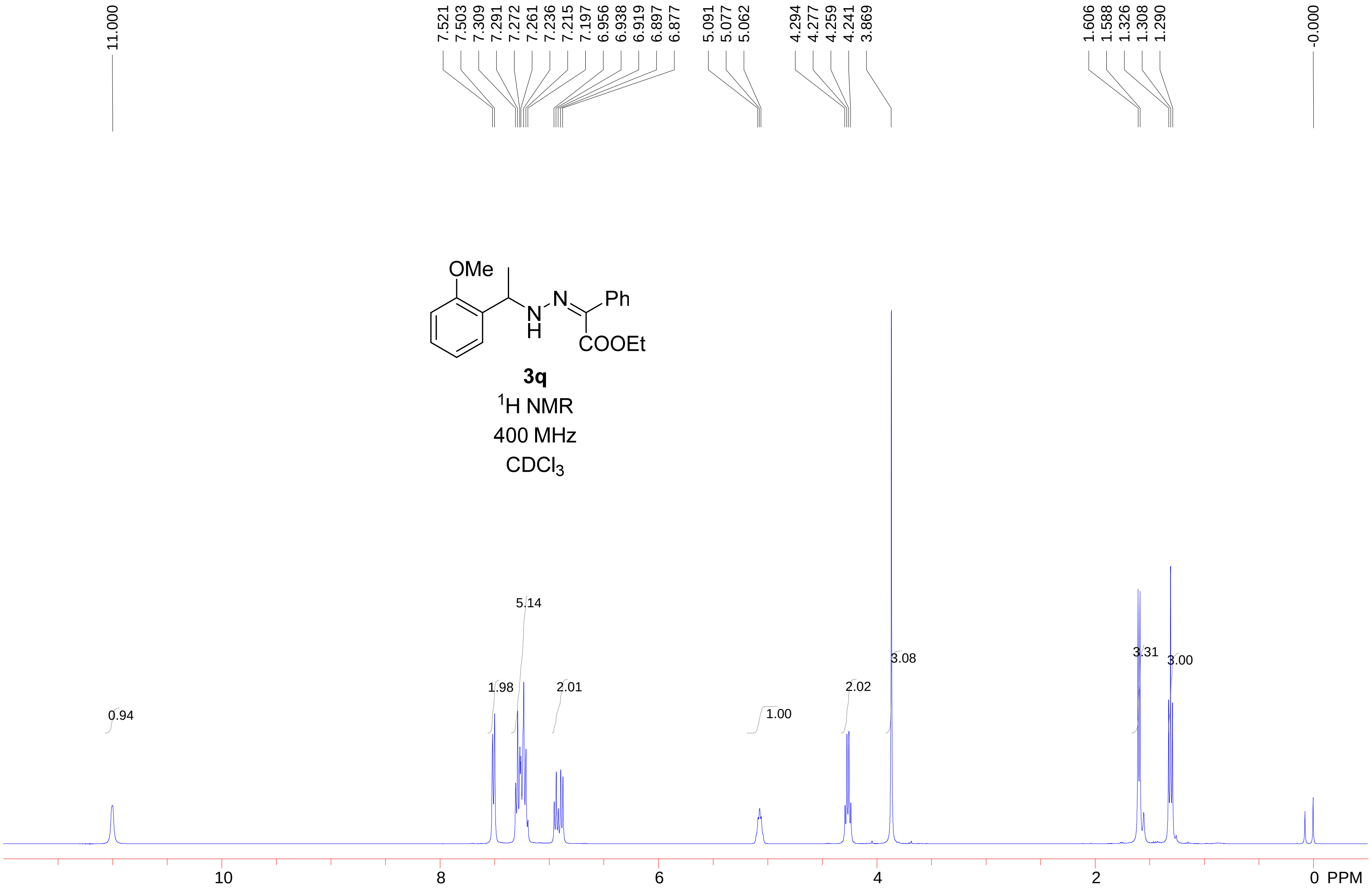

Supplementary Figure 44. ^{1}H NMR spectrum for **3q**

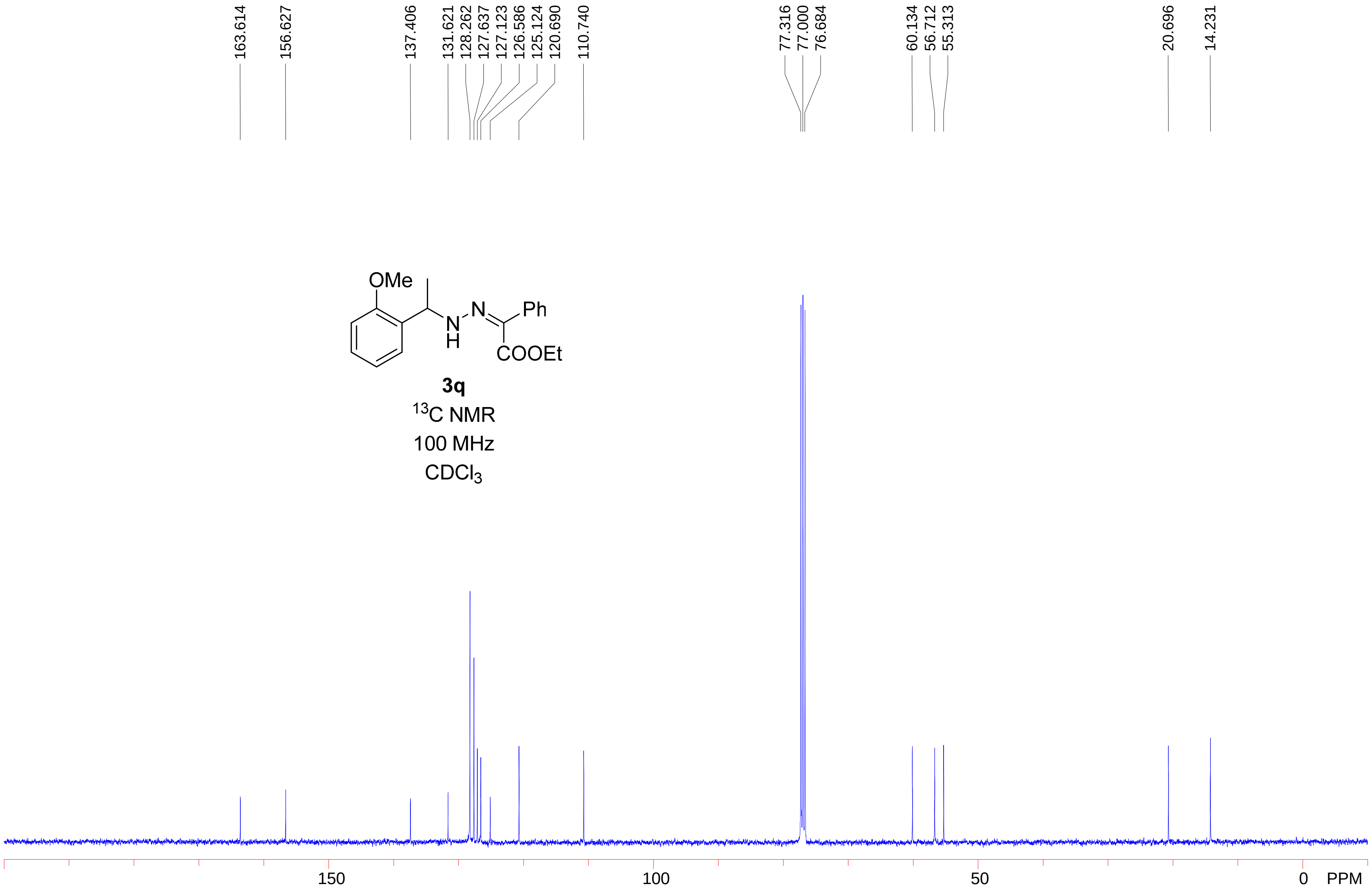

Supplementary Figure 45. ^{13}C NMR spectrum for **3q**

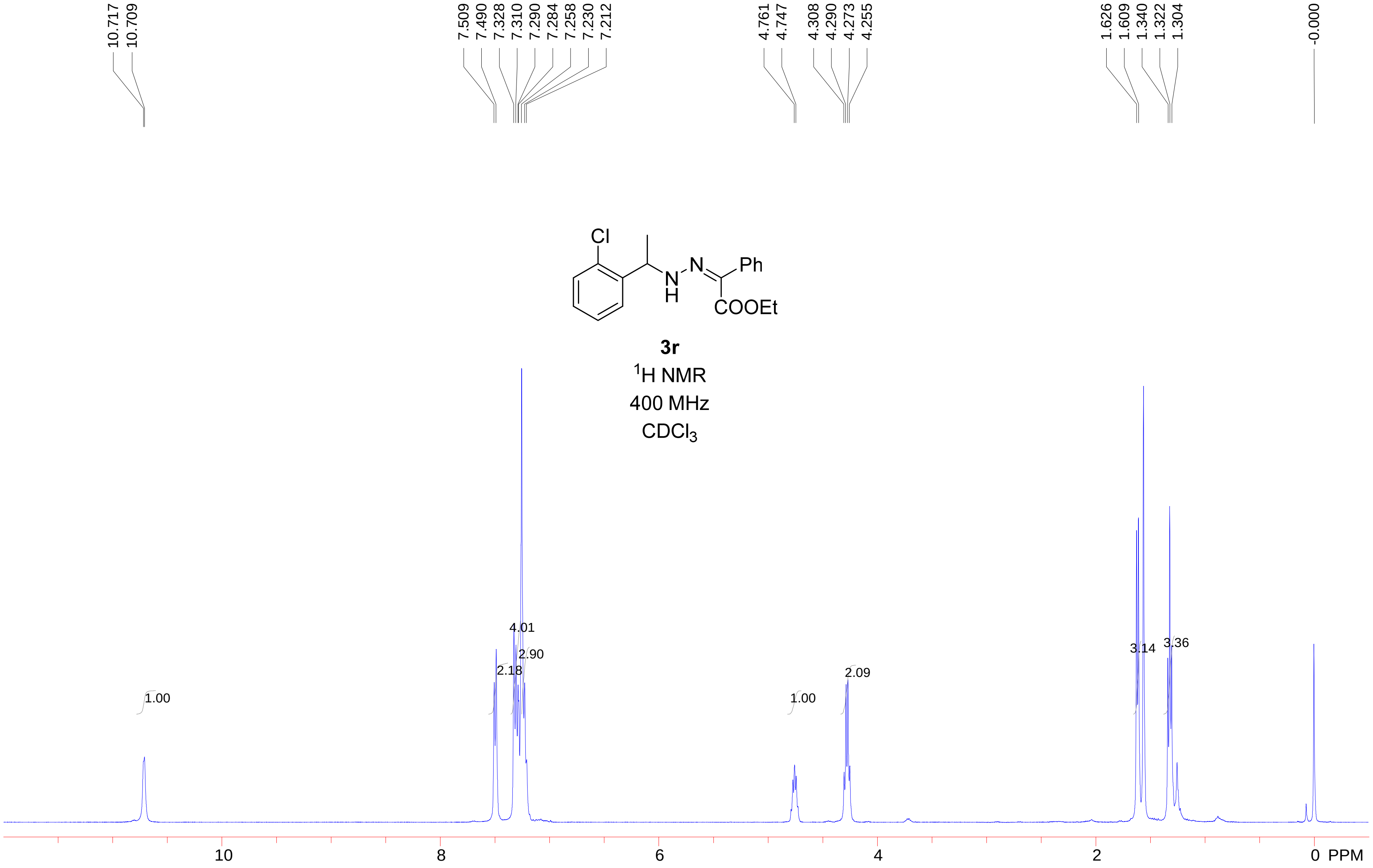

Supplementary Figure 46. ^{1}H NMR spectrum for **3r**

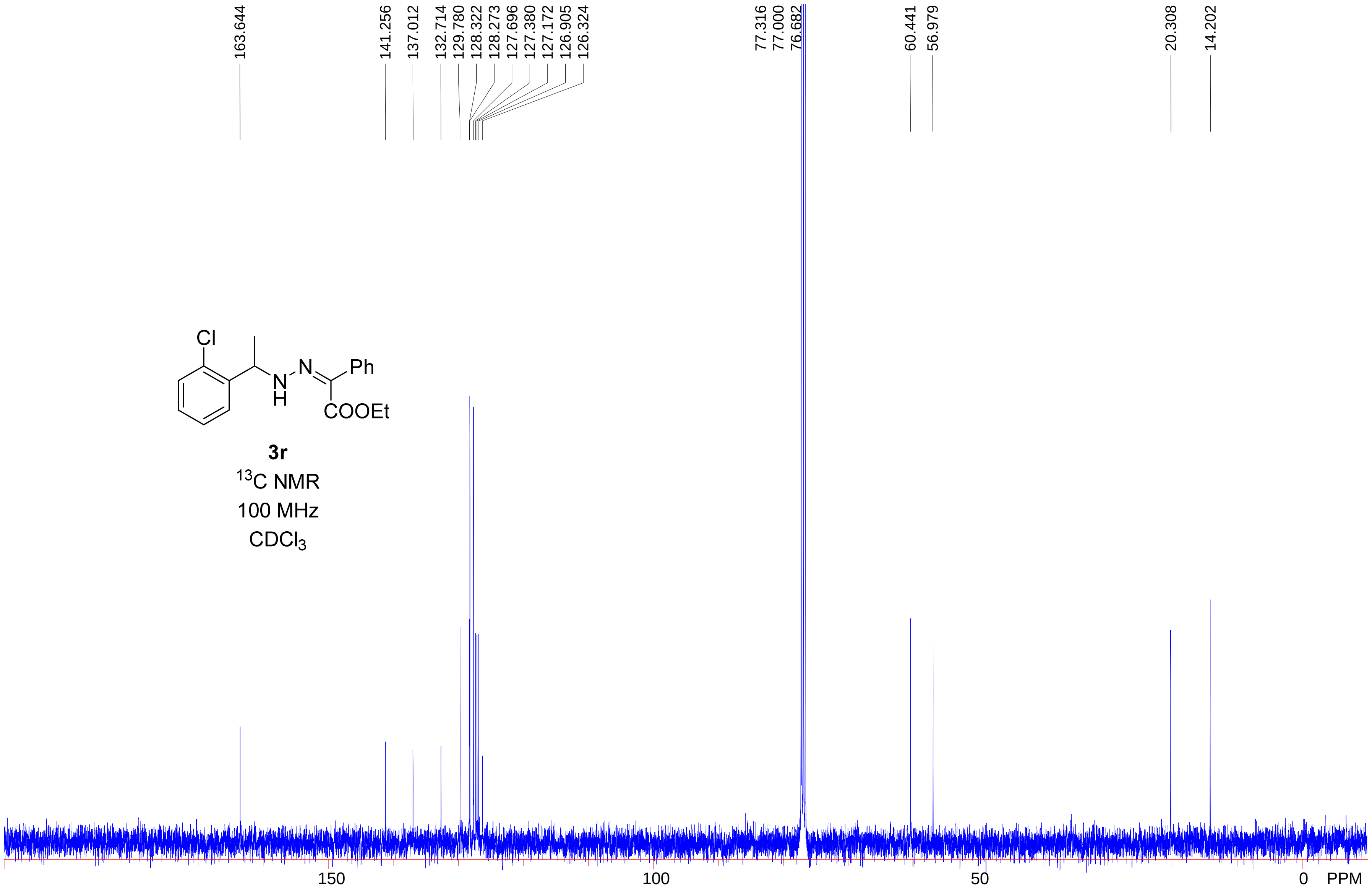

Supplementary Figure 47. ^{13}C NMR spectrum for **3r**

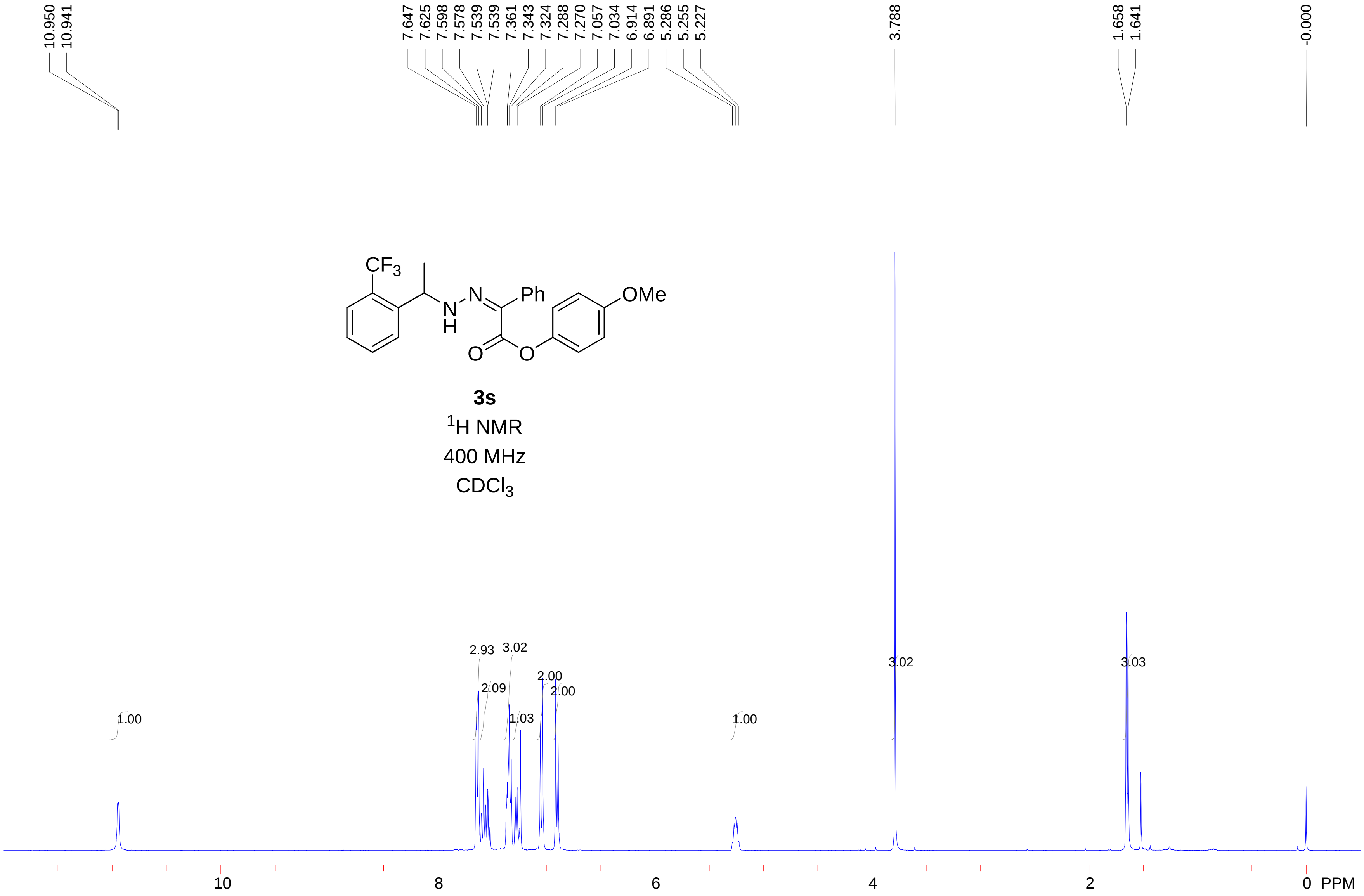

Supplementary Figure 48. ^{1}H NMR spectrum for **3s**

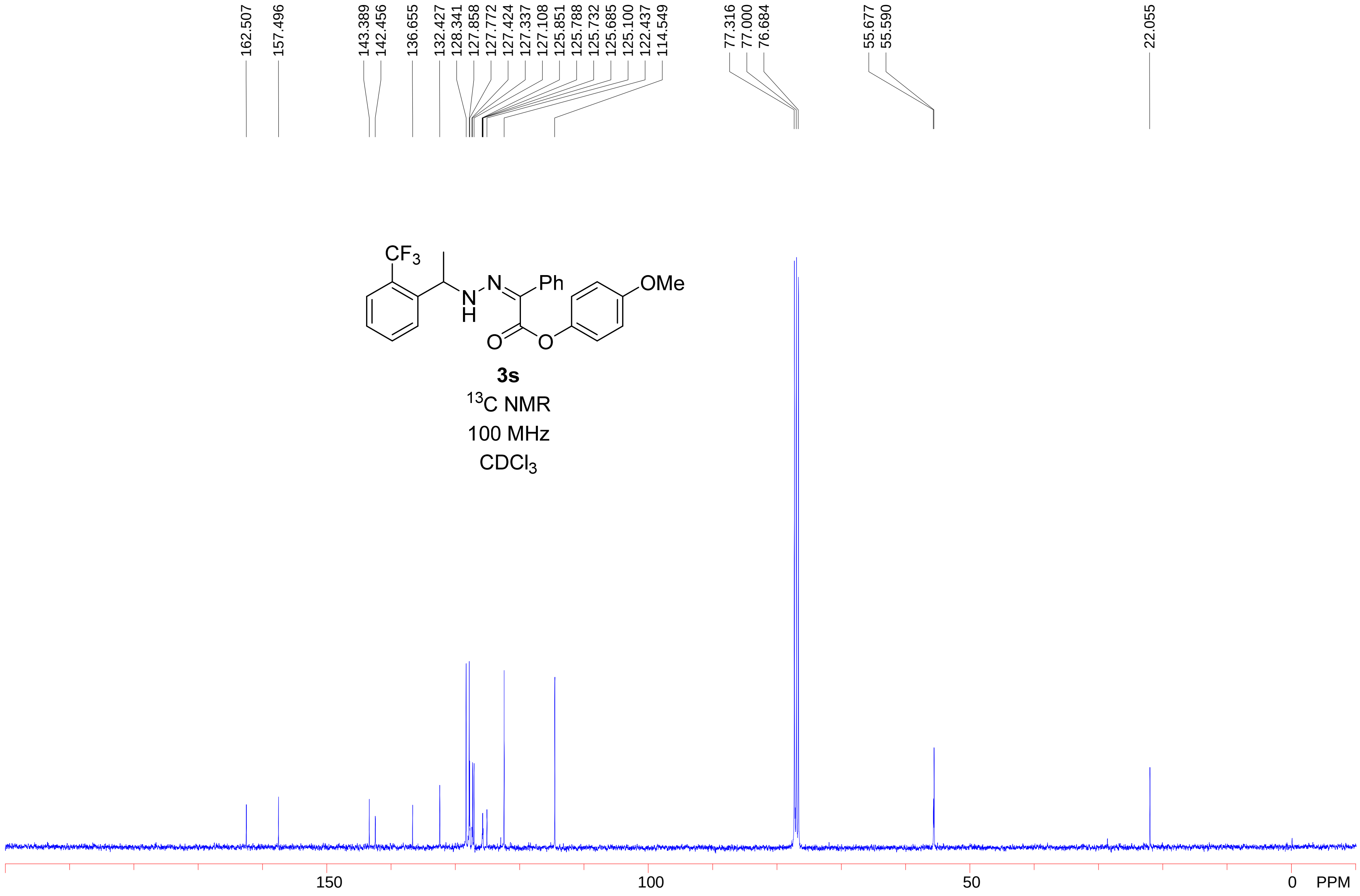

Supplementary Figure 49. ^{13}C NMR spectrum for **3s**

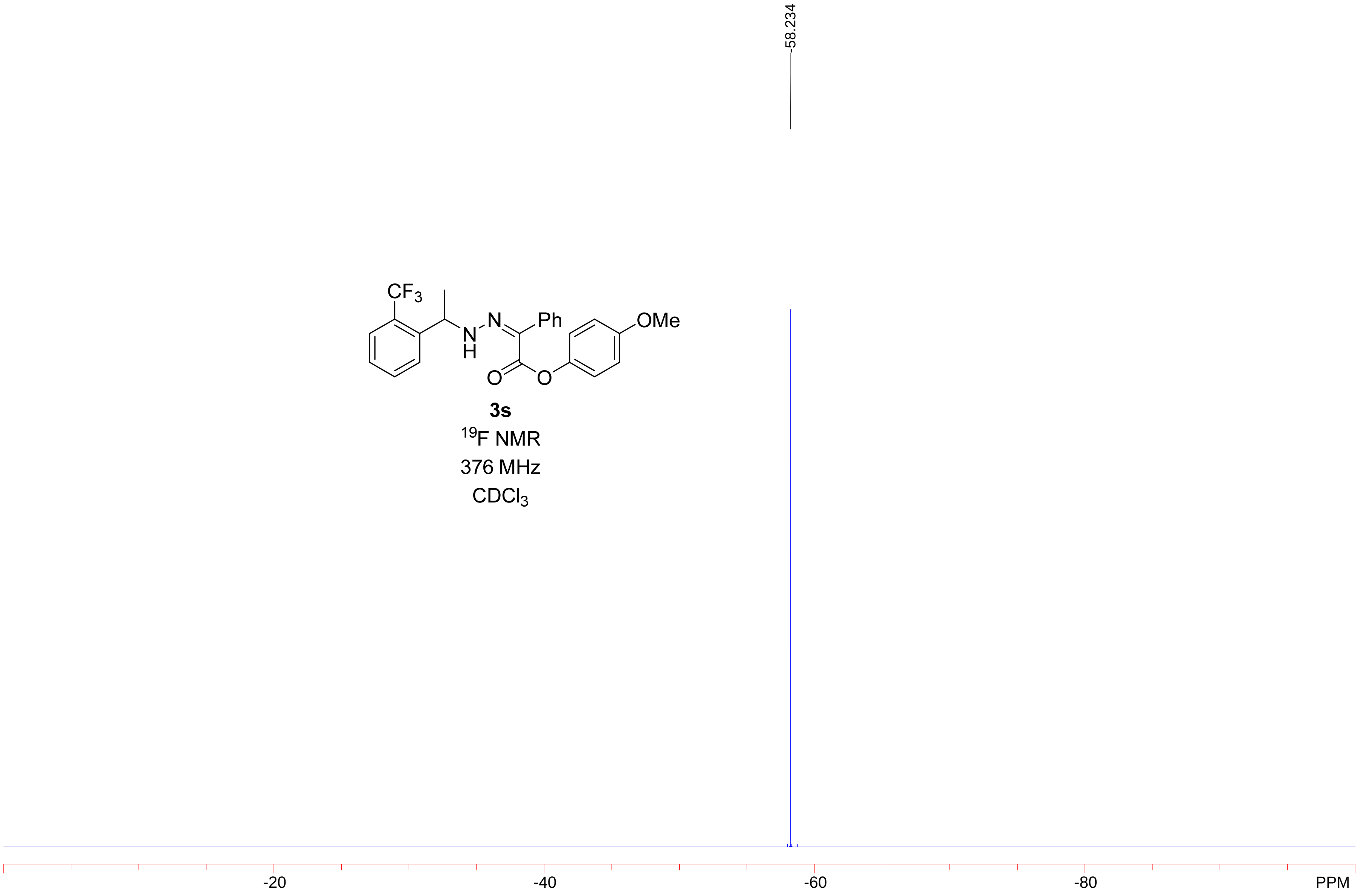

Supplementary Figure 50. ^{19}F NMR spectrum for **3s**

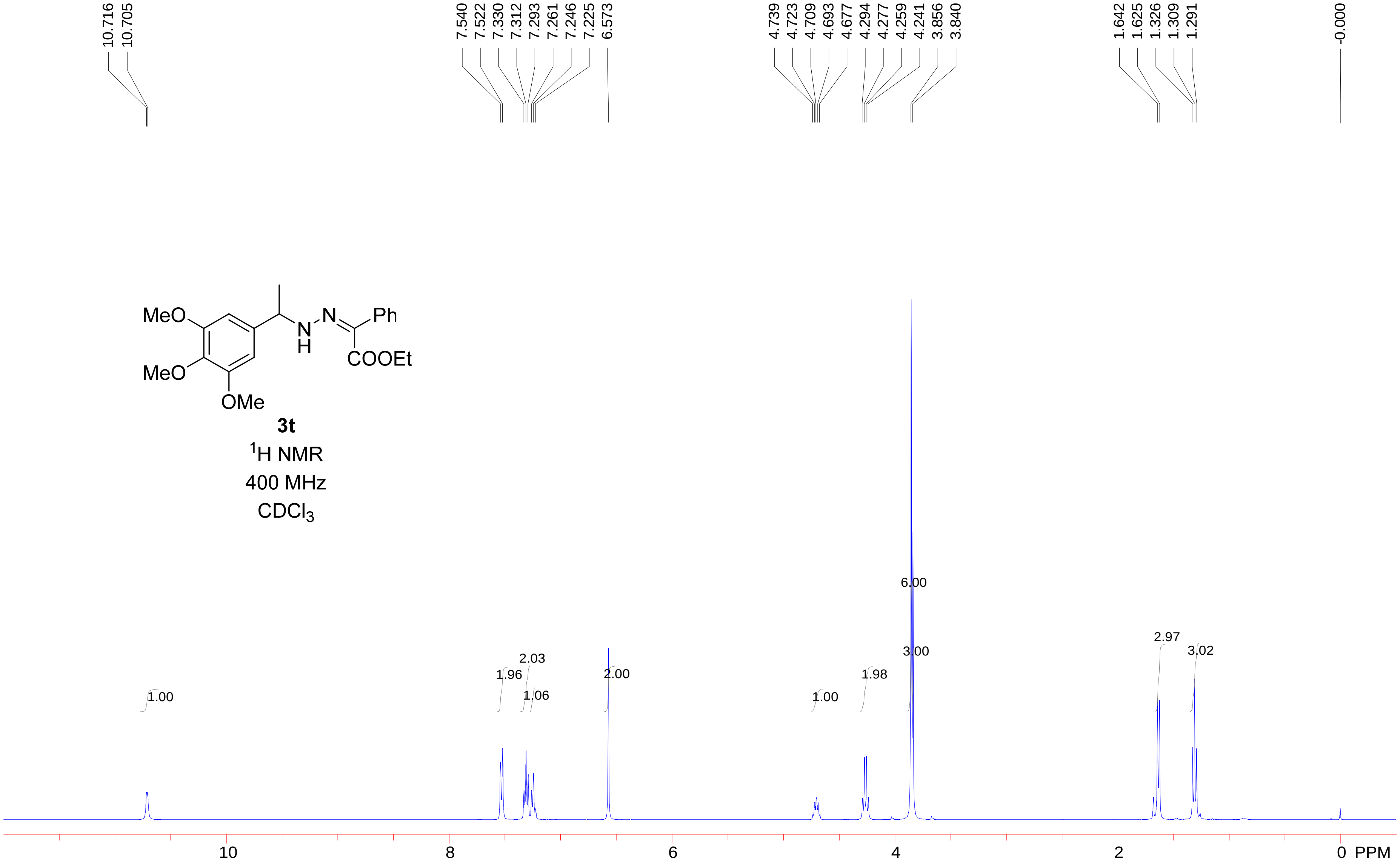

Supplementary Figure 51. ¹H NMR spectrum for **3t**

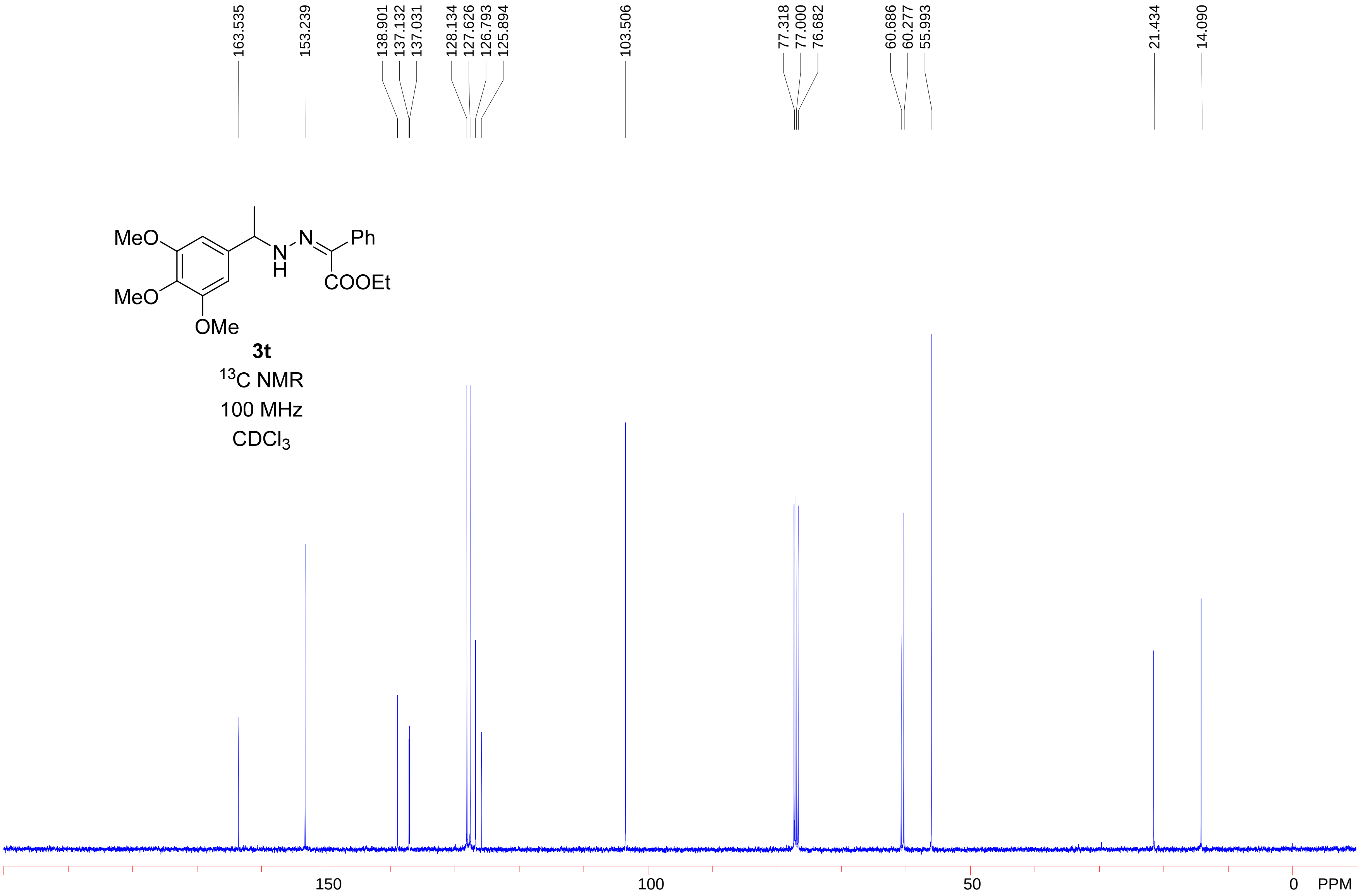

Supplementary Figure 52. ^{13}C NMR spectrum for **3t**

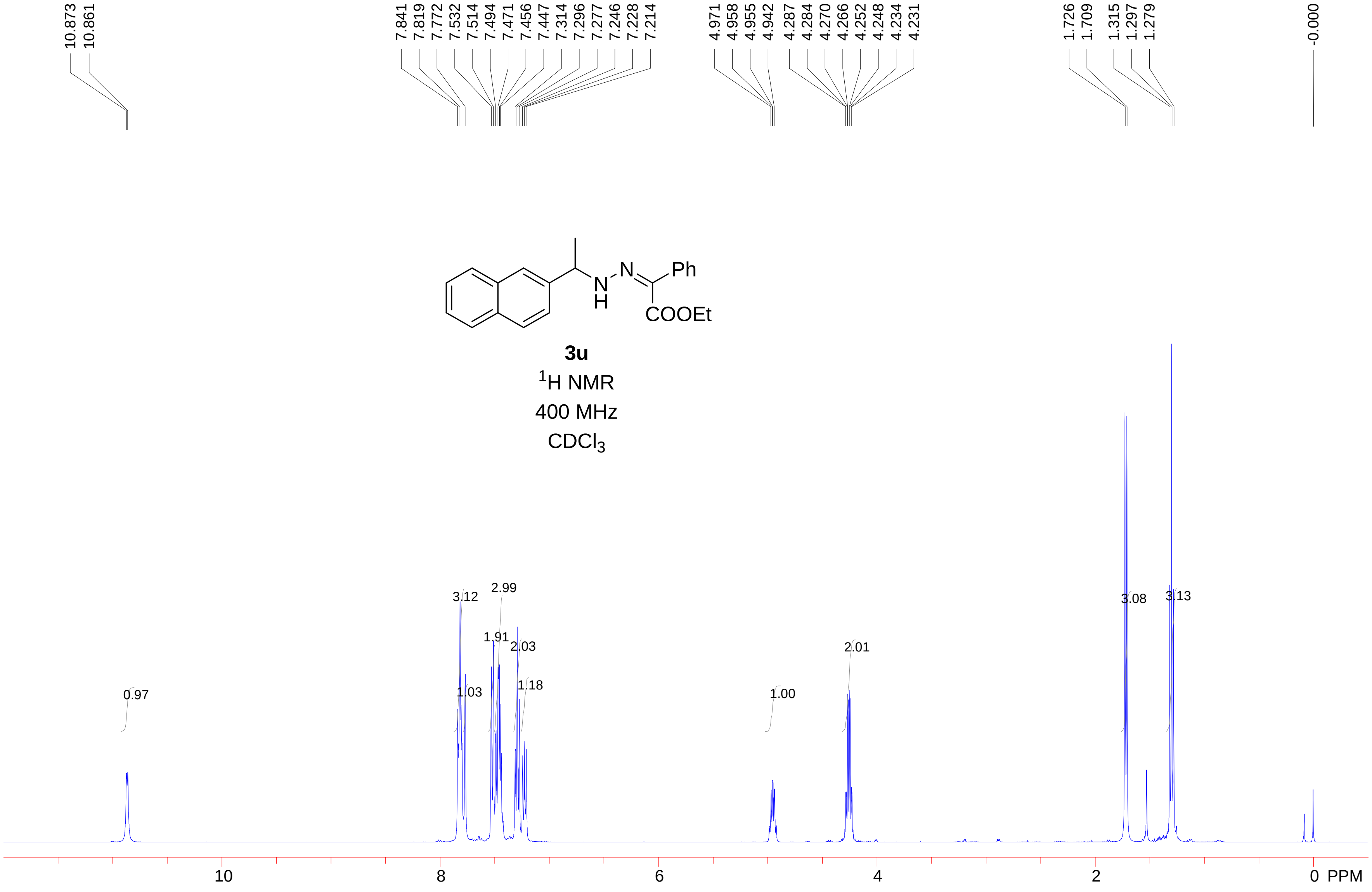

Supplementary Figure 53. ^{1}H NMR spectrum for **3u**

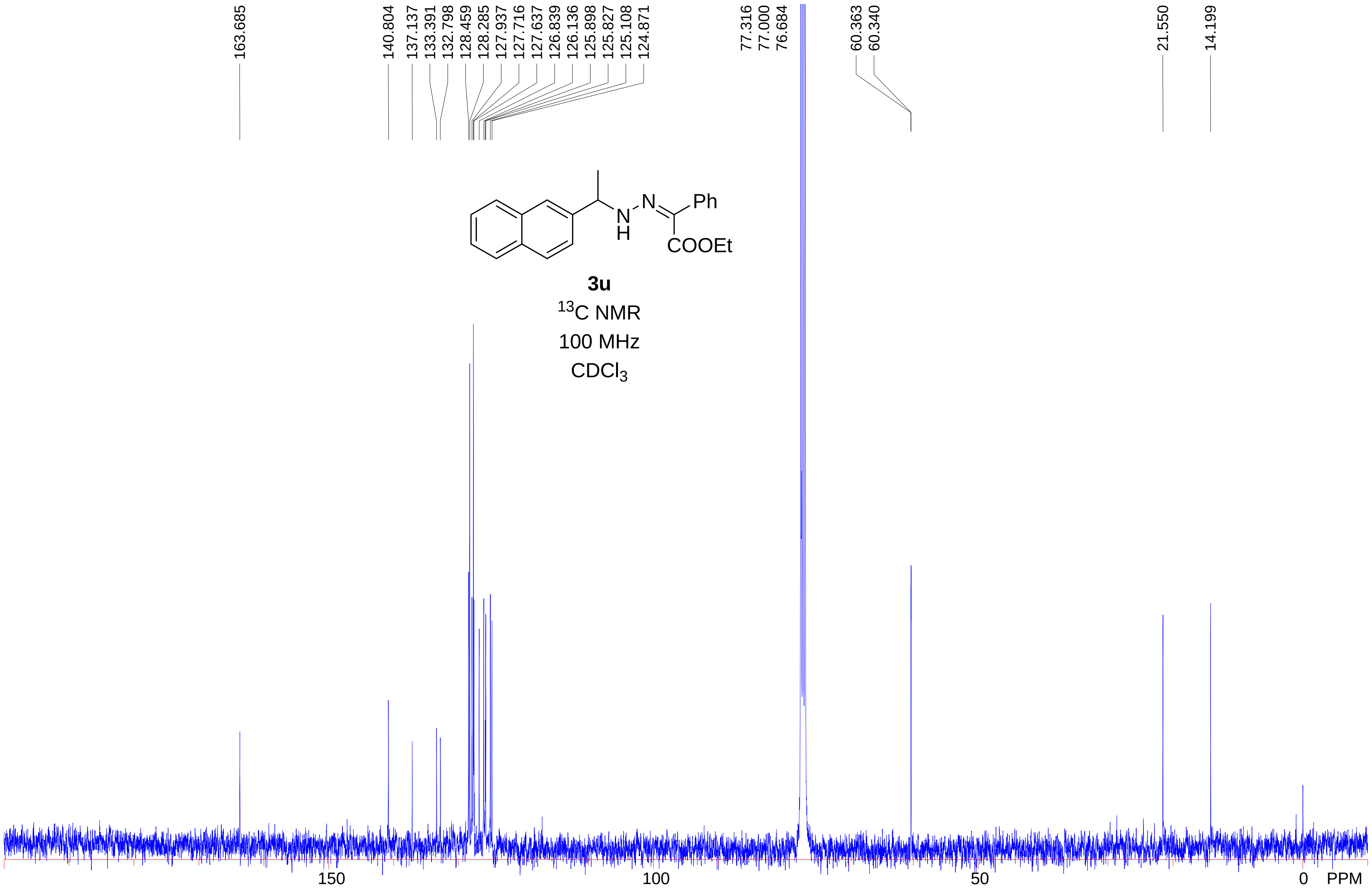

Supplementary Figure 54. ^{13}C NMR spectrum for **3u**

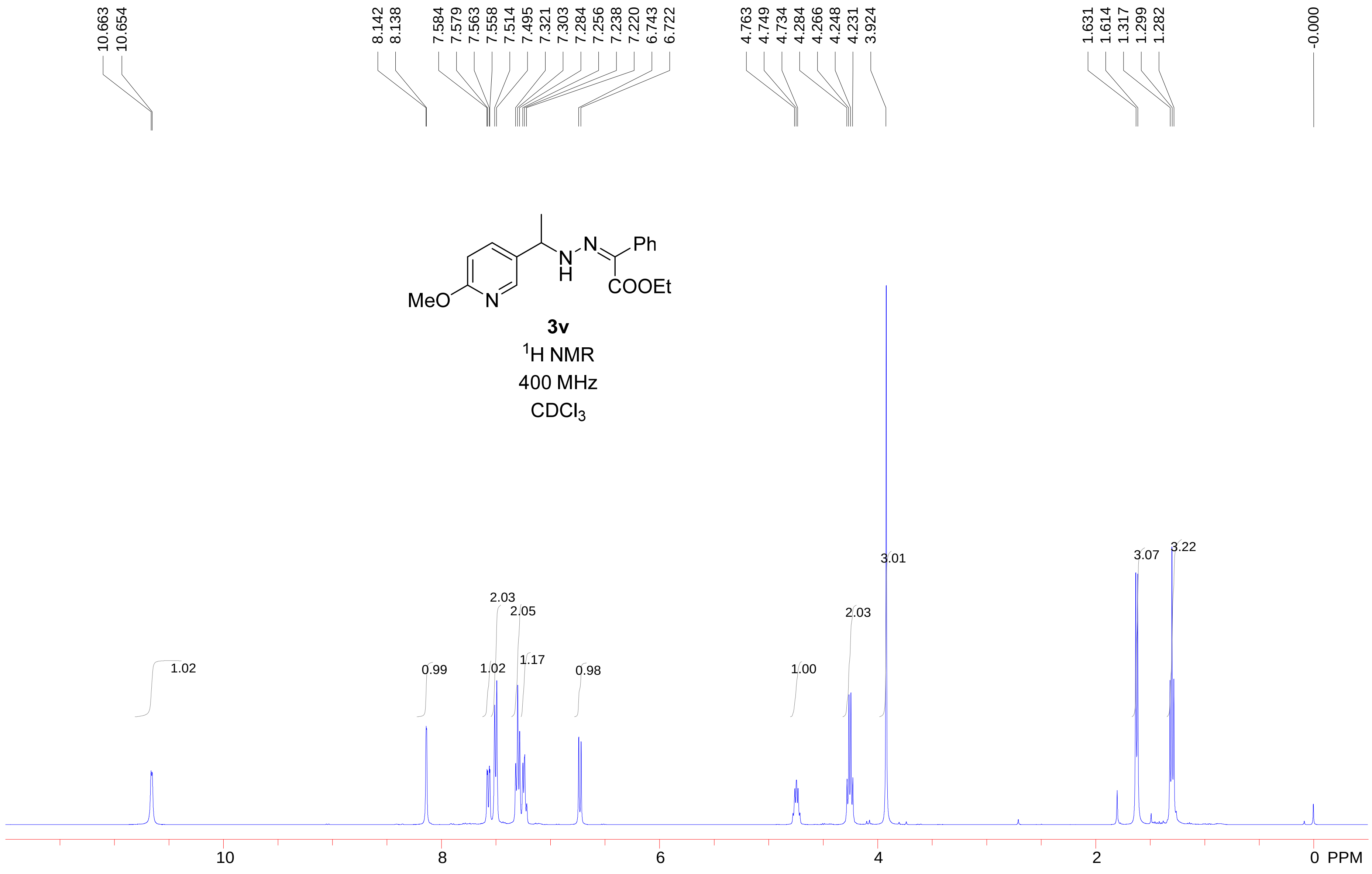

Supplementary Figure 55. ^{1}H NMR spectrum for **3v**

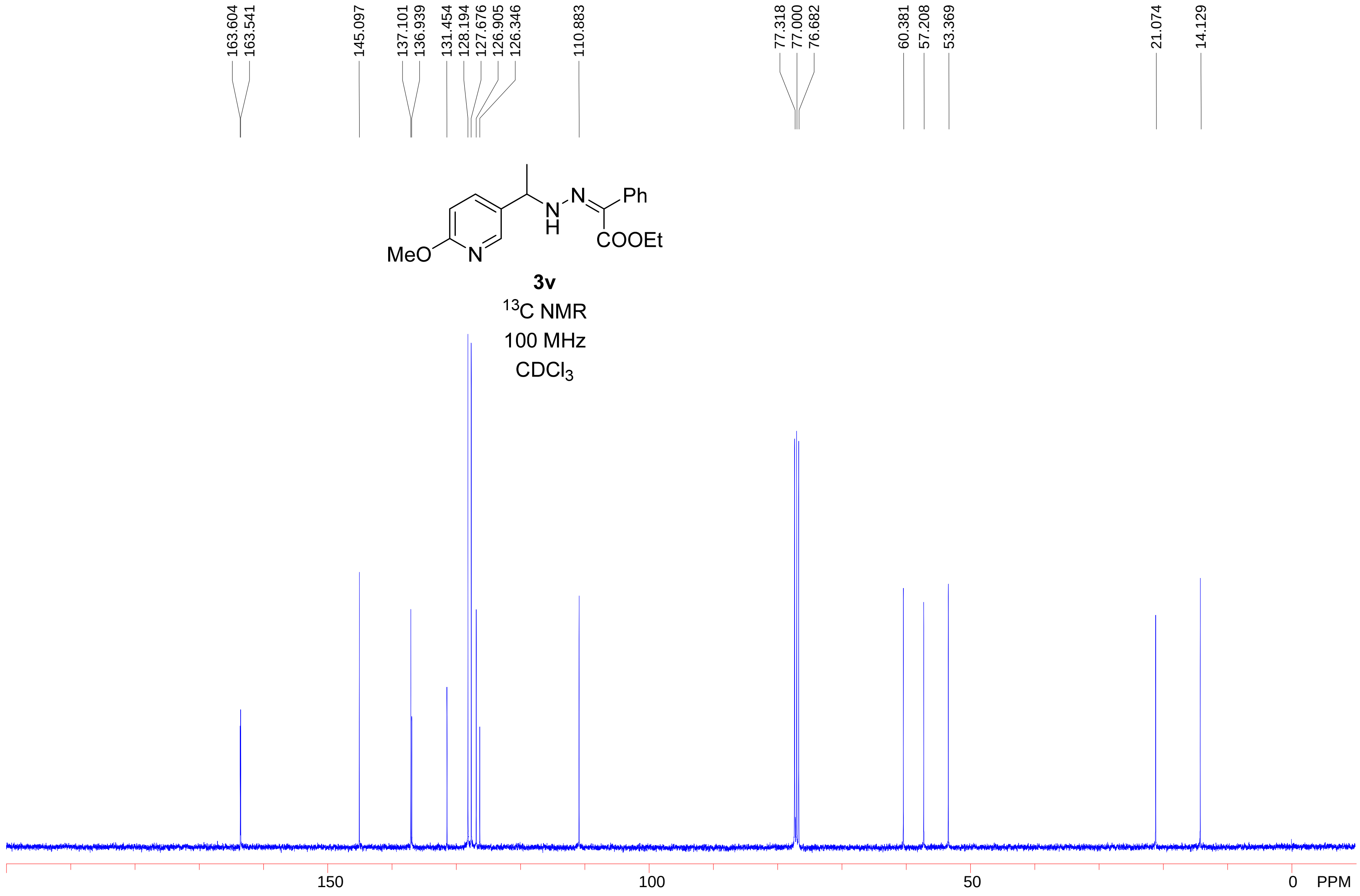

Supplementary Figure 56. ^{13}C NMR spectrum for **3v**

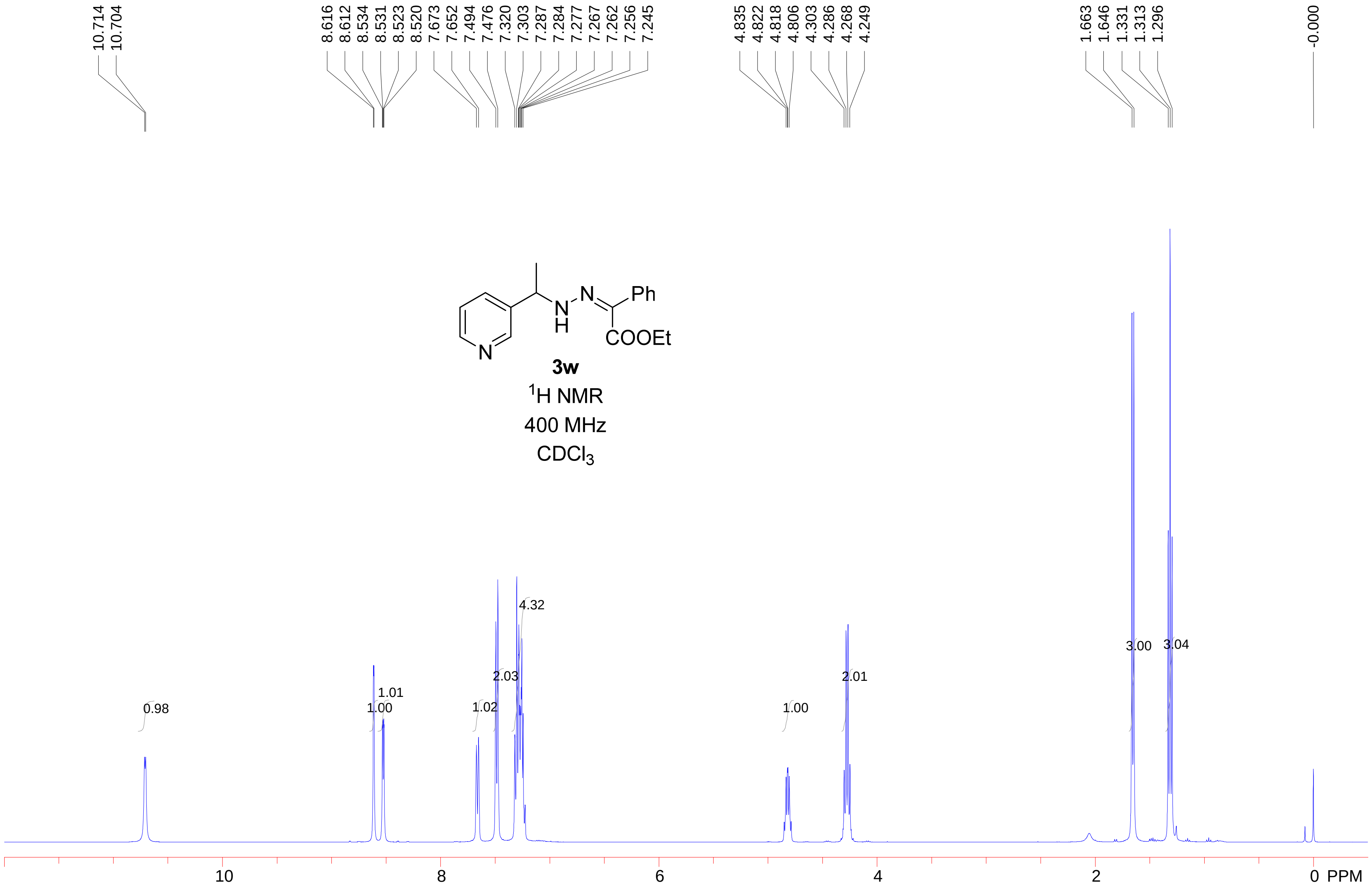

Supplementary Figure 57. ^{1}H NMR spectrum for **3w**

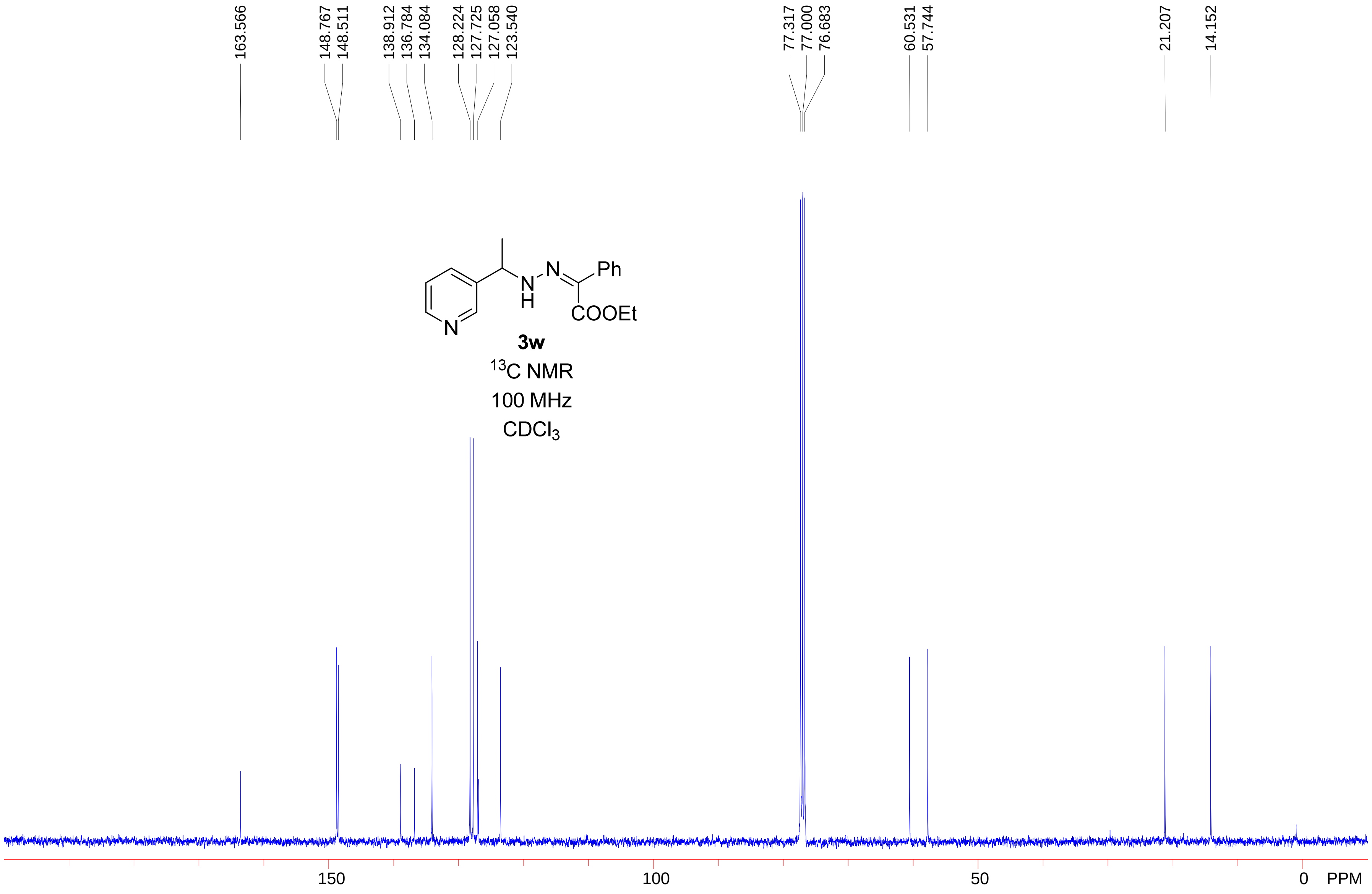

Supplementary Figure 58. ^{13}C NMR spectrum for **3w**

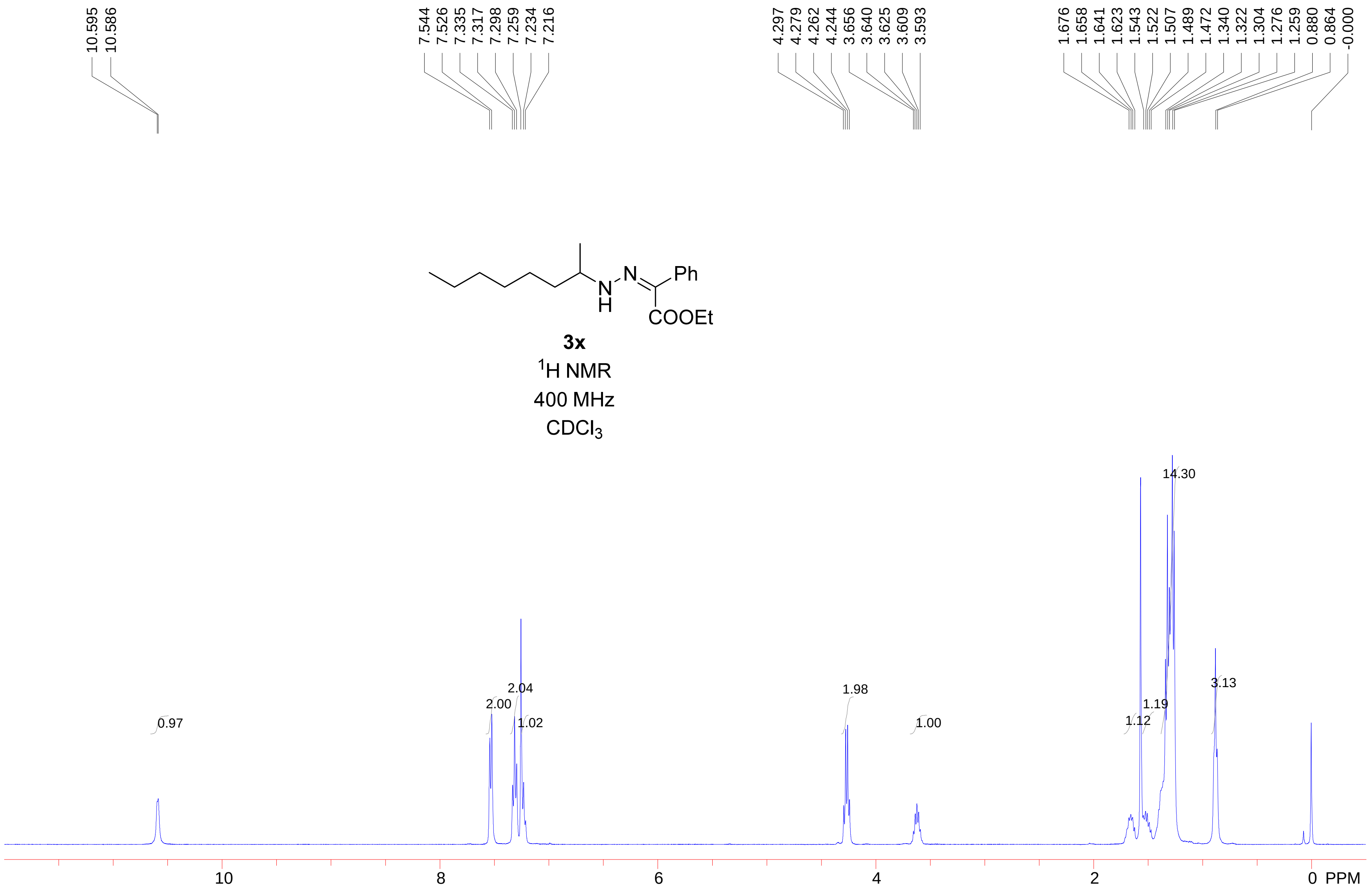

Supplementary Figure 59. ^{1}H NMR spectrum for **3x**

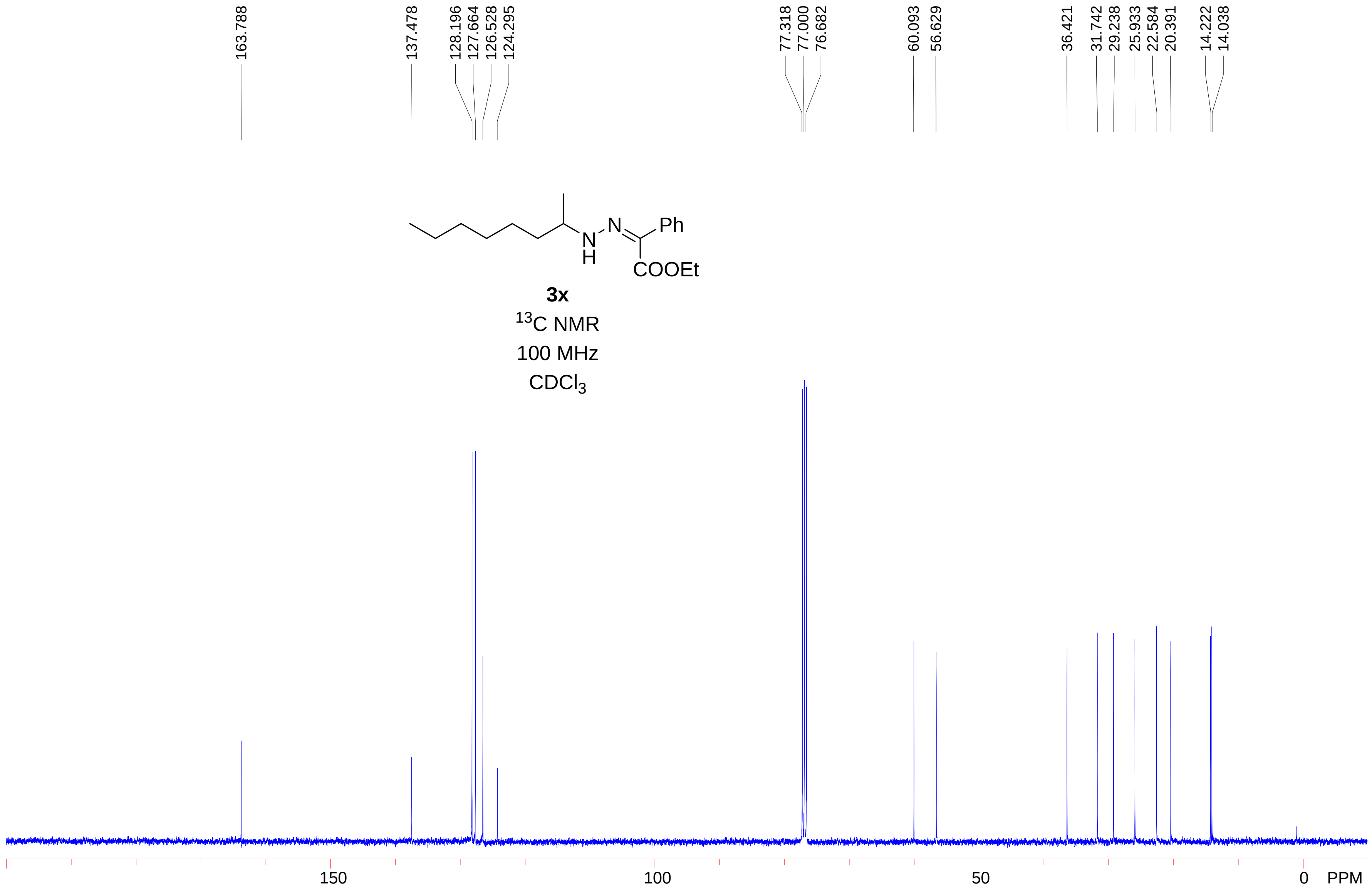

Supplementary Figure 60. ^{13}C NMR spectrum for **3x**

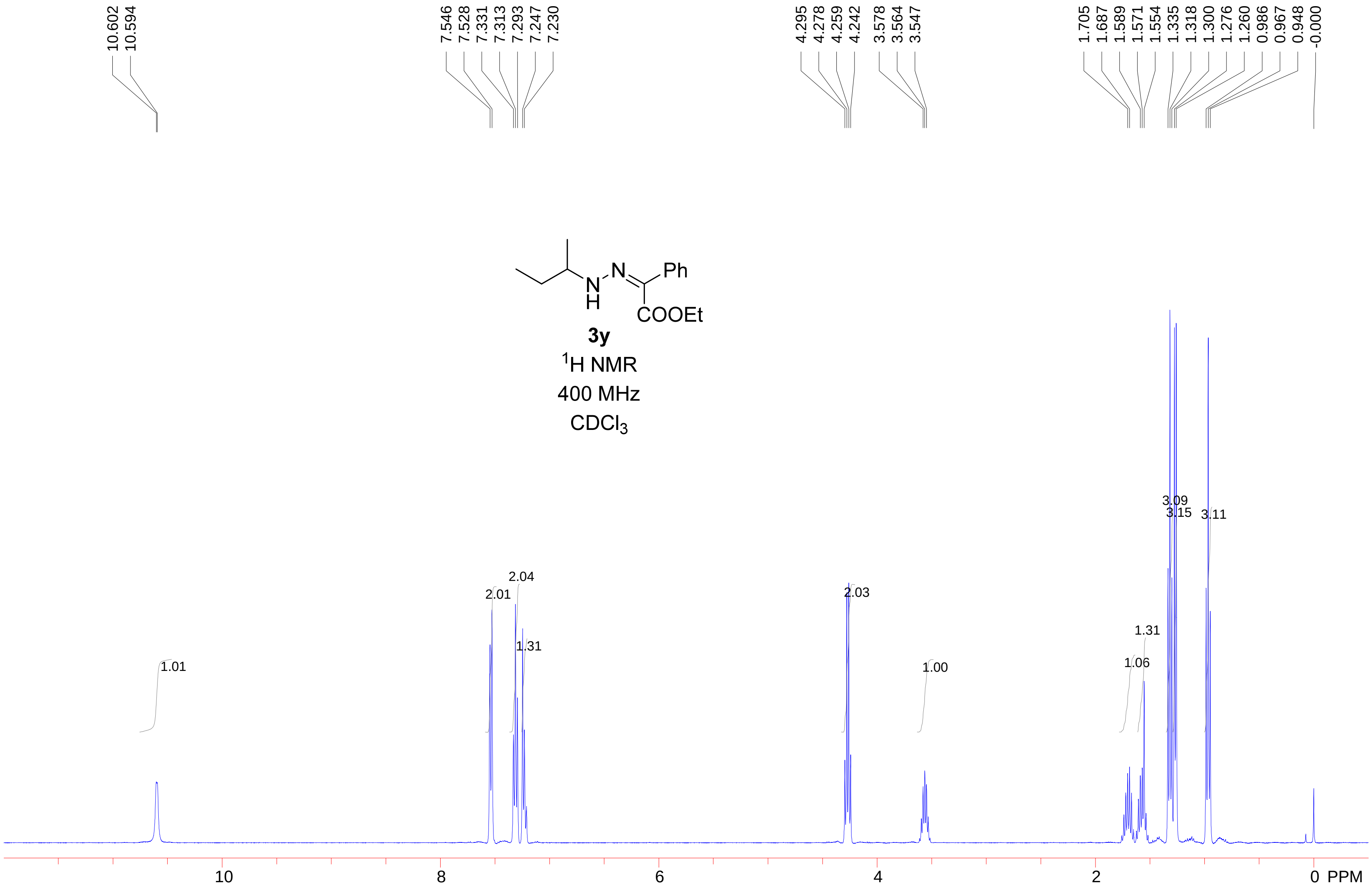

Supplementary Figure 61. ^{1}H NMR spectrum for **3y**

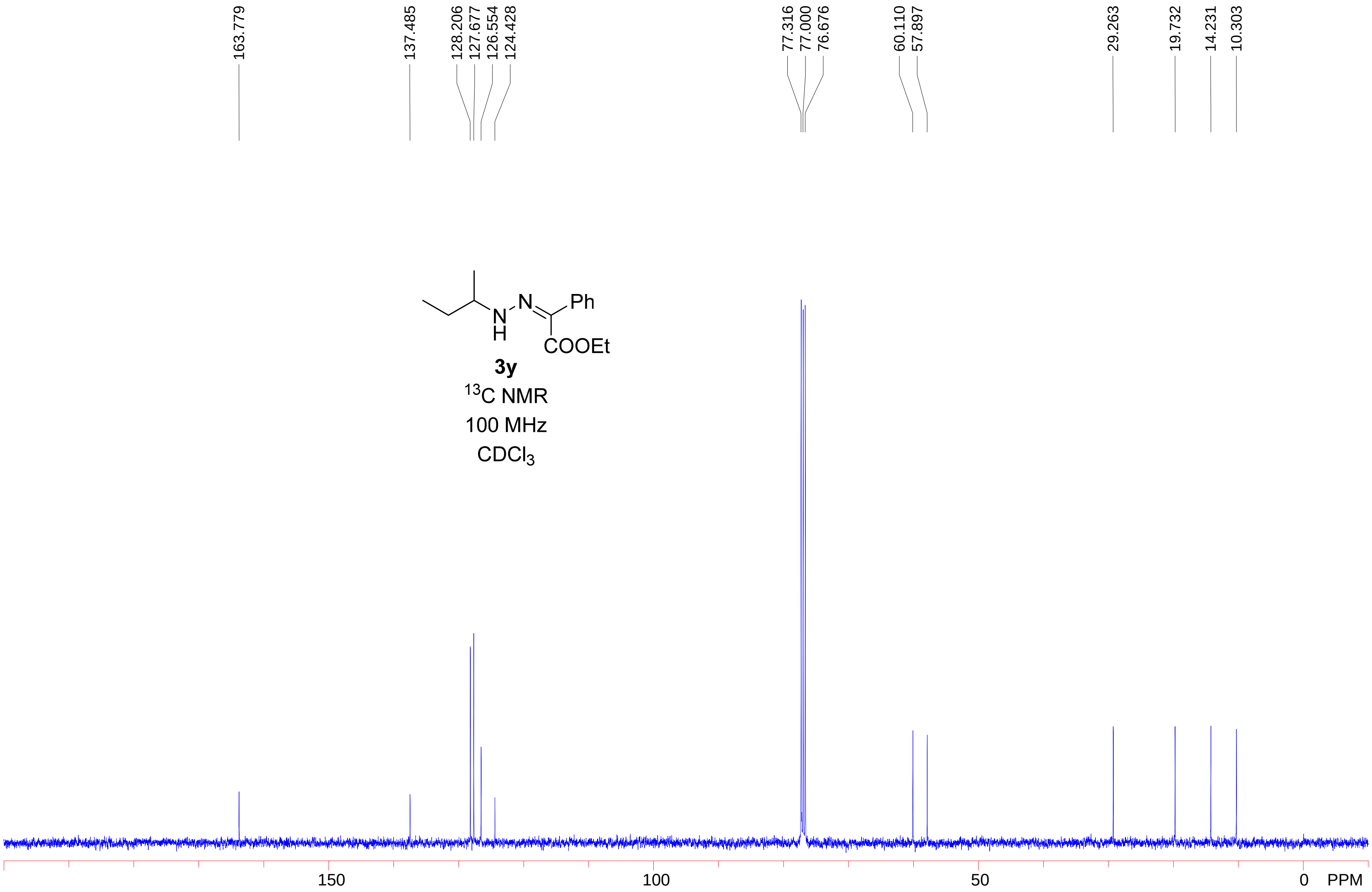

Supplementary Figure 62. ^{13}C NMR spectrum for **3y**

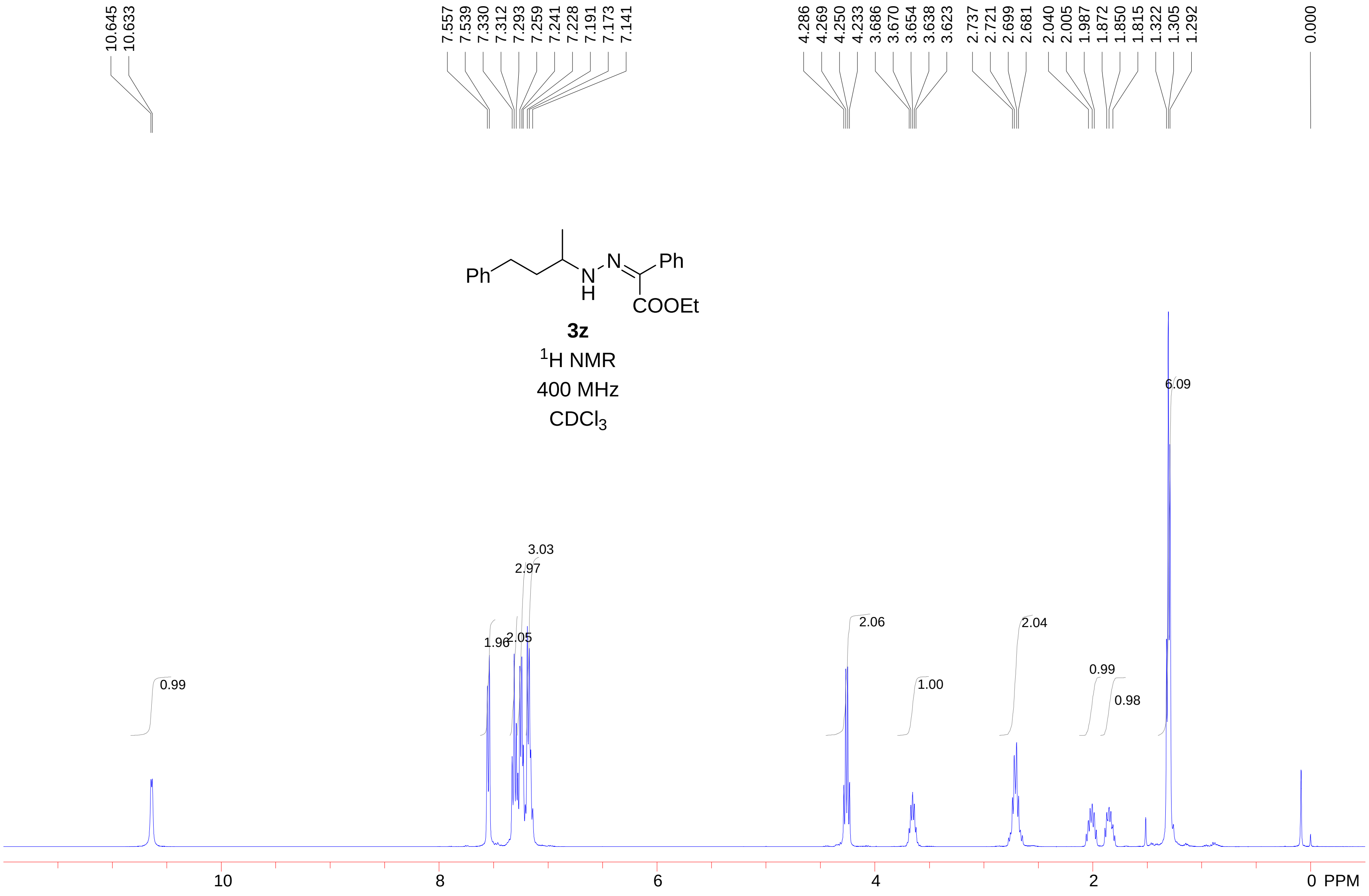

Supplementary Figure 63. ^{1}H NMR spectrum for **3z**

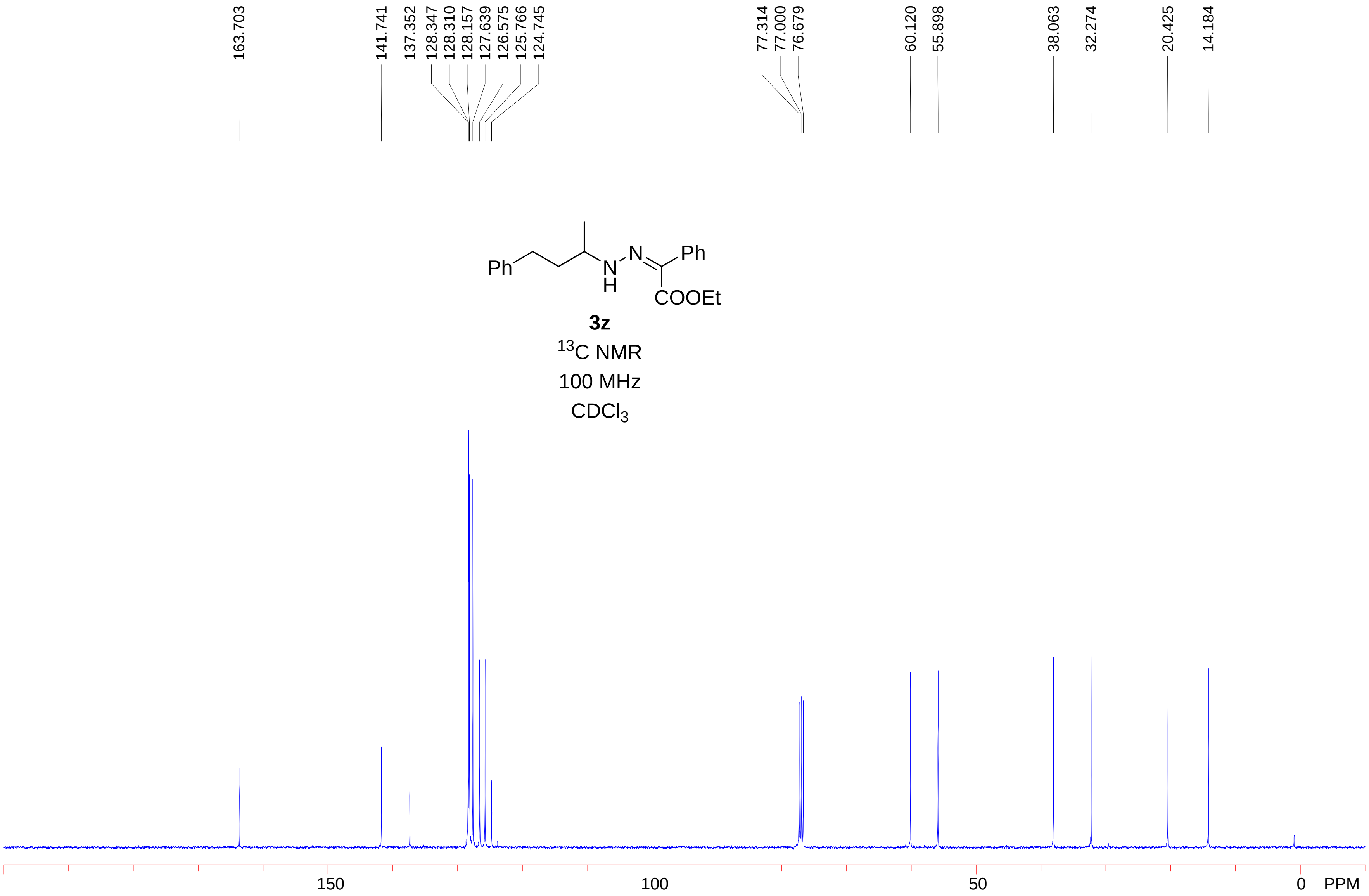

Supplementary Figure 64. ^{13}C NMR spectrum for **3z**

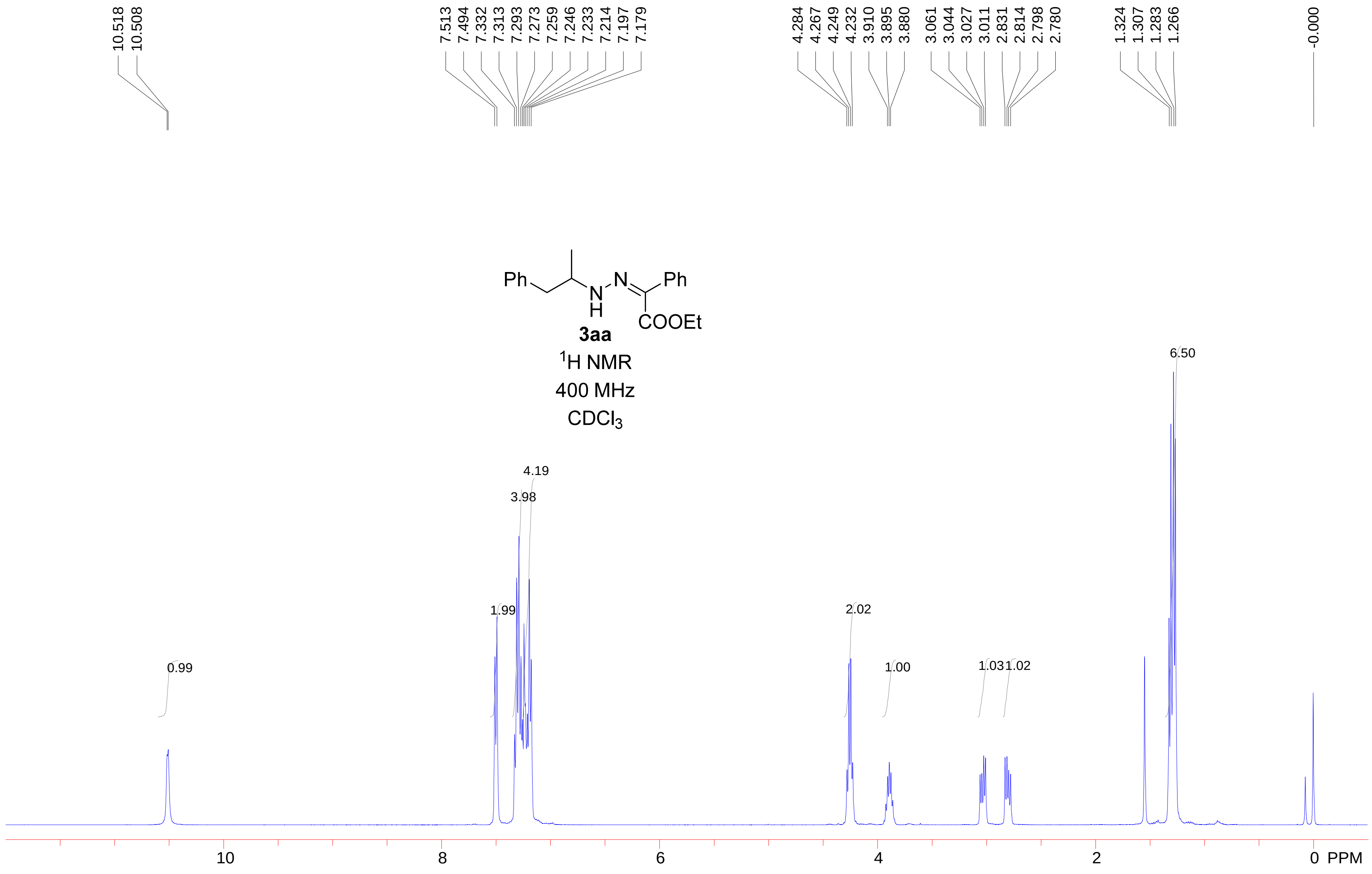

Supplementary Figure 65. ^{1}H NMR spectrum for **3aa**

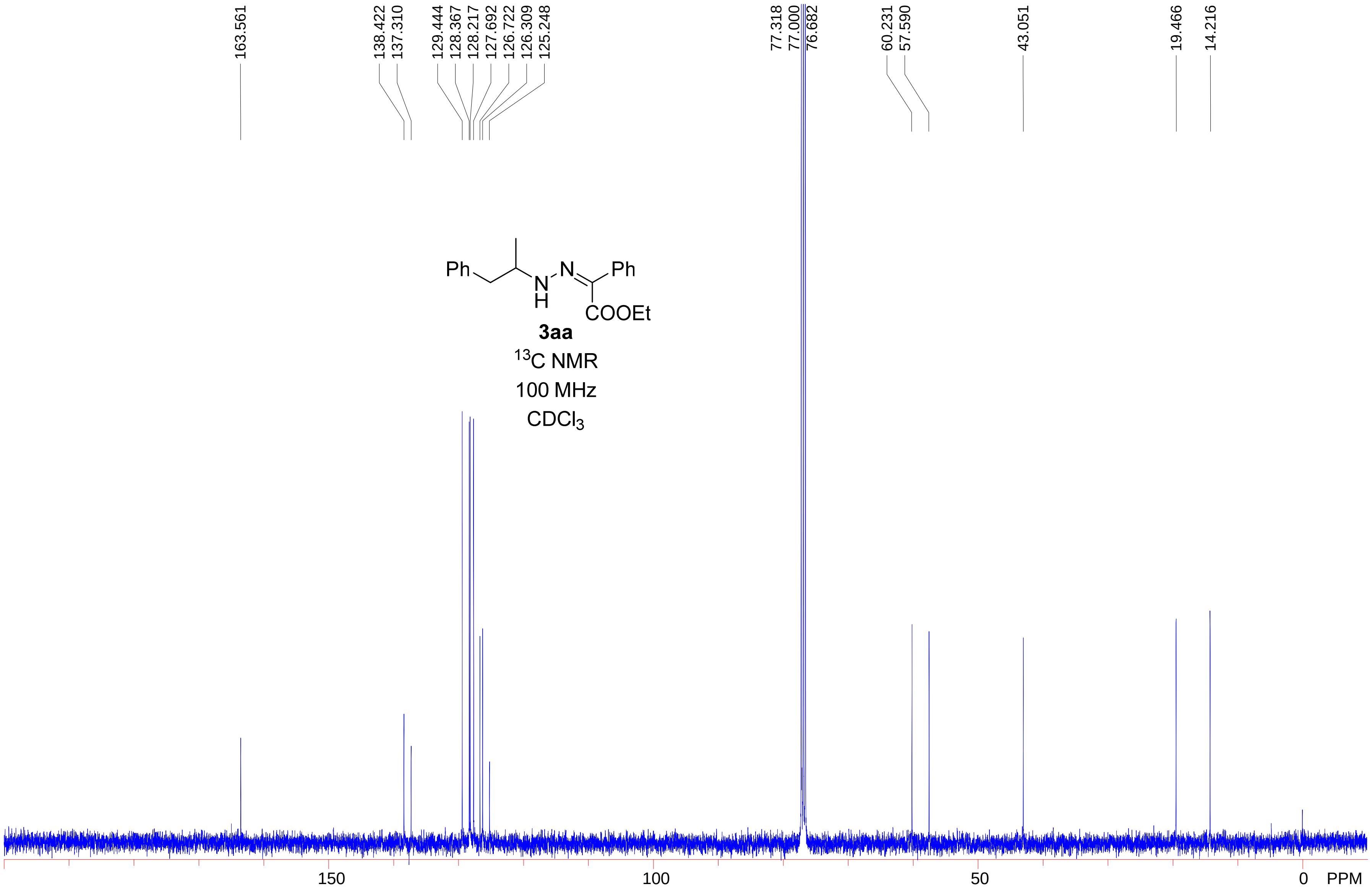

Supplementary Figure 66. ^{13}C NMR spectrum for **3aa**

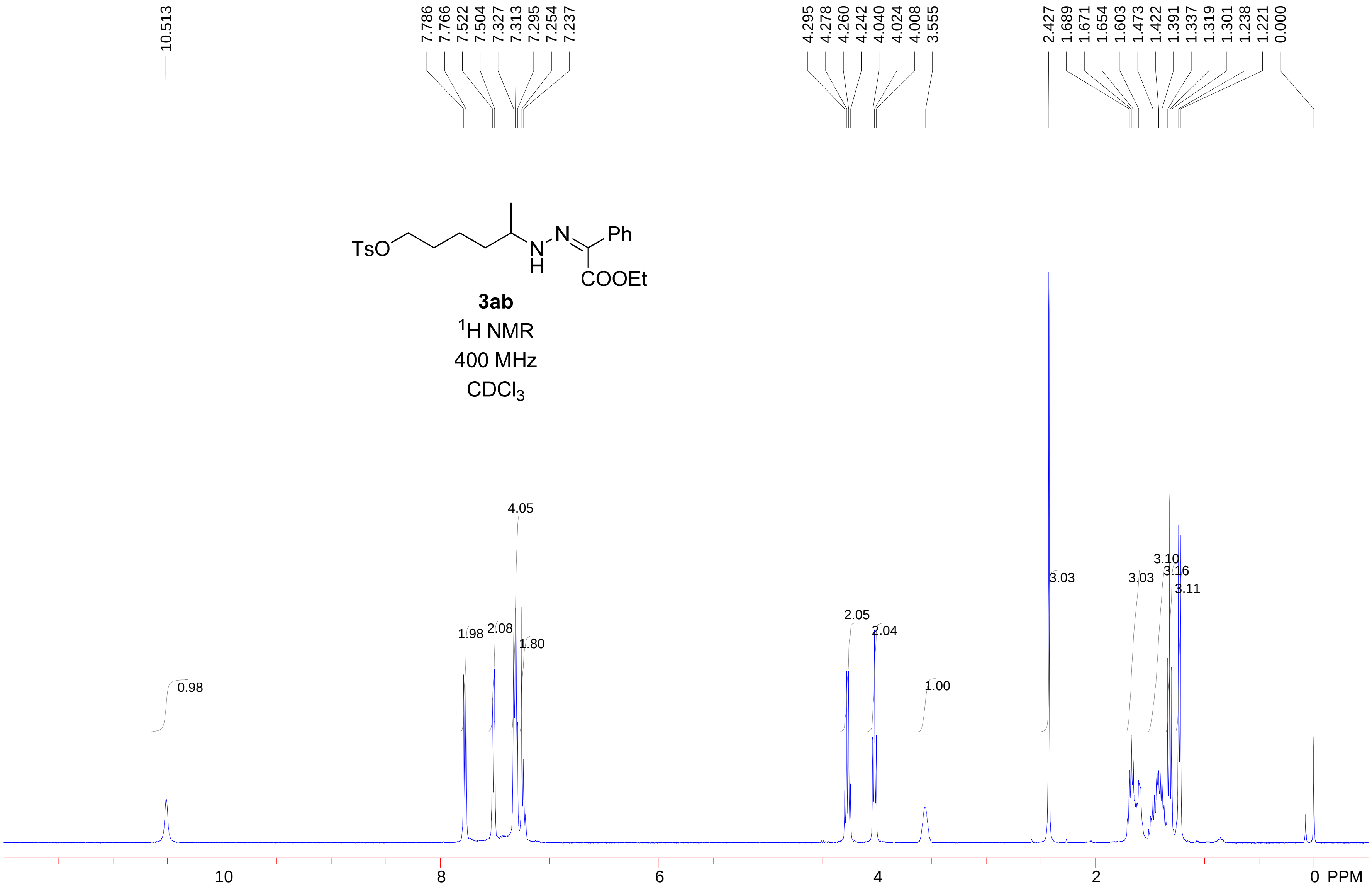

Supplementary Figure 67. ^{1}H NMR spectrum for **3ab**

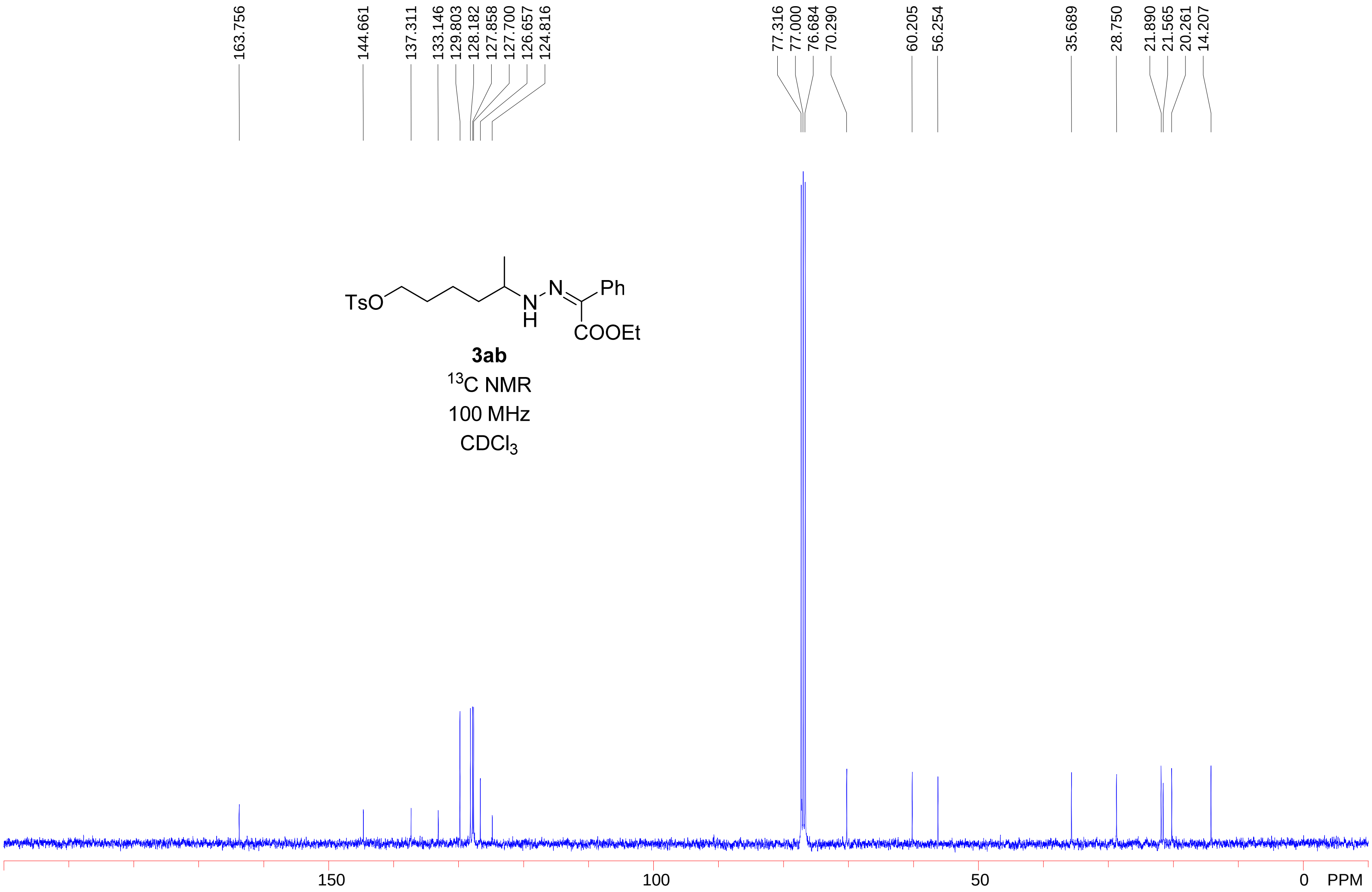

Supplementary Figure 68. ^{13}C NMR spectrum for **3ab**

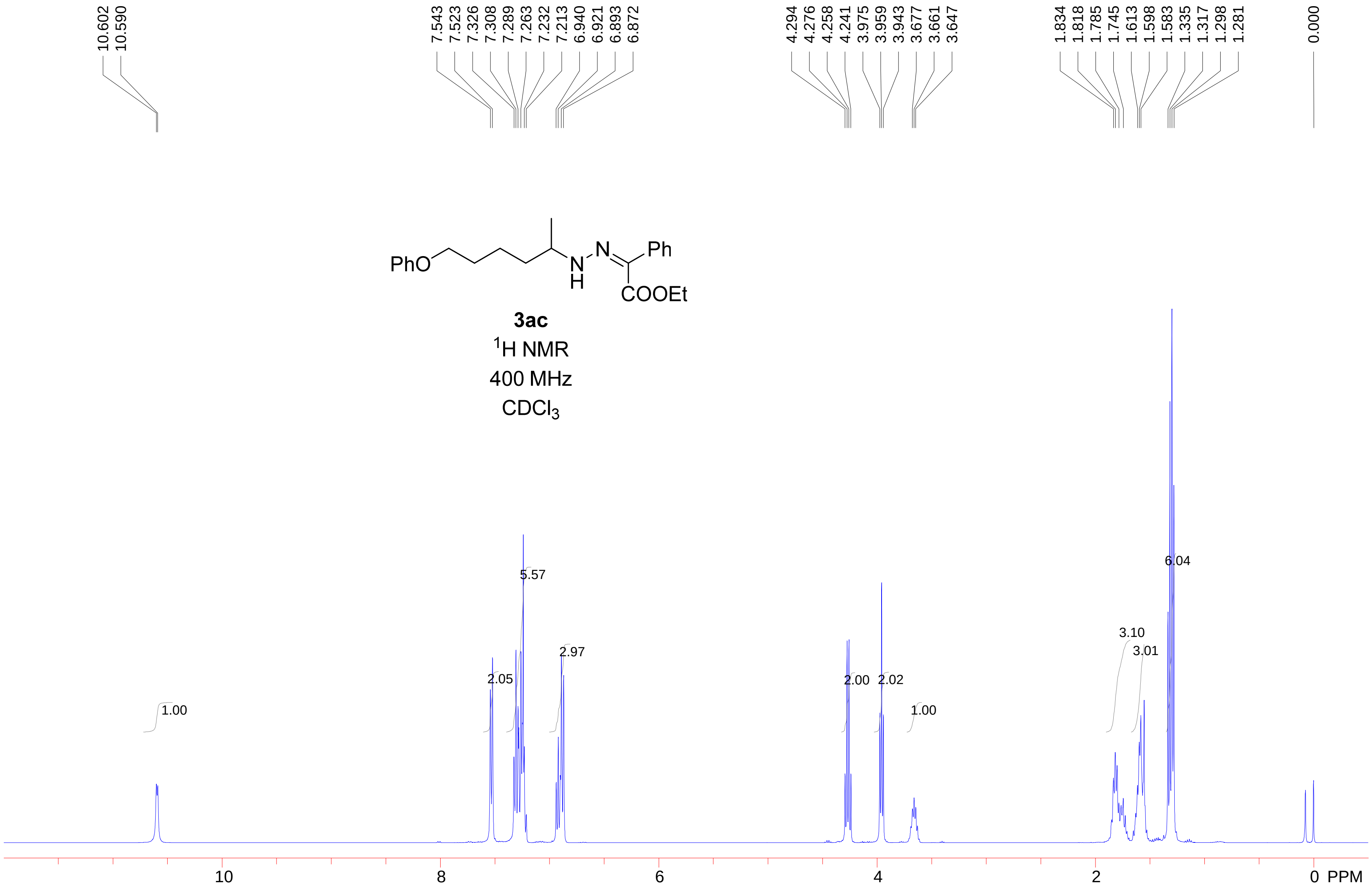

Supplementary Figure 69. ^{1}H NMR spectrum for **3ac**

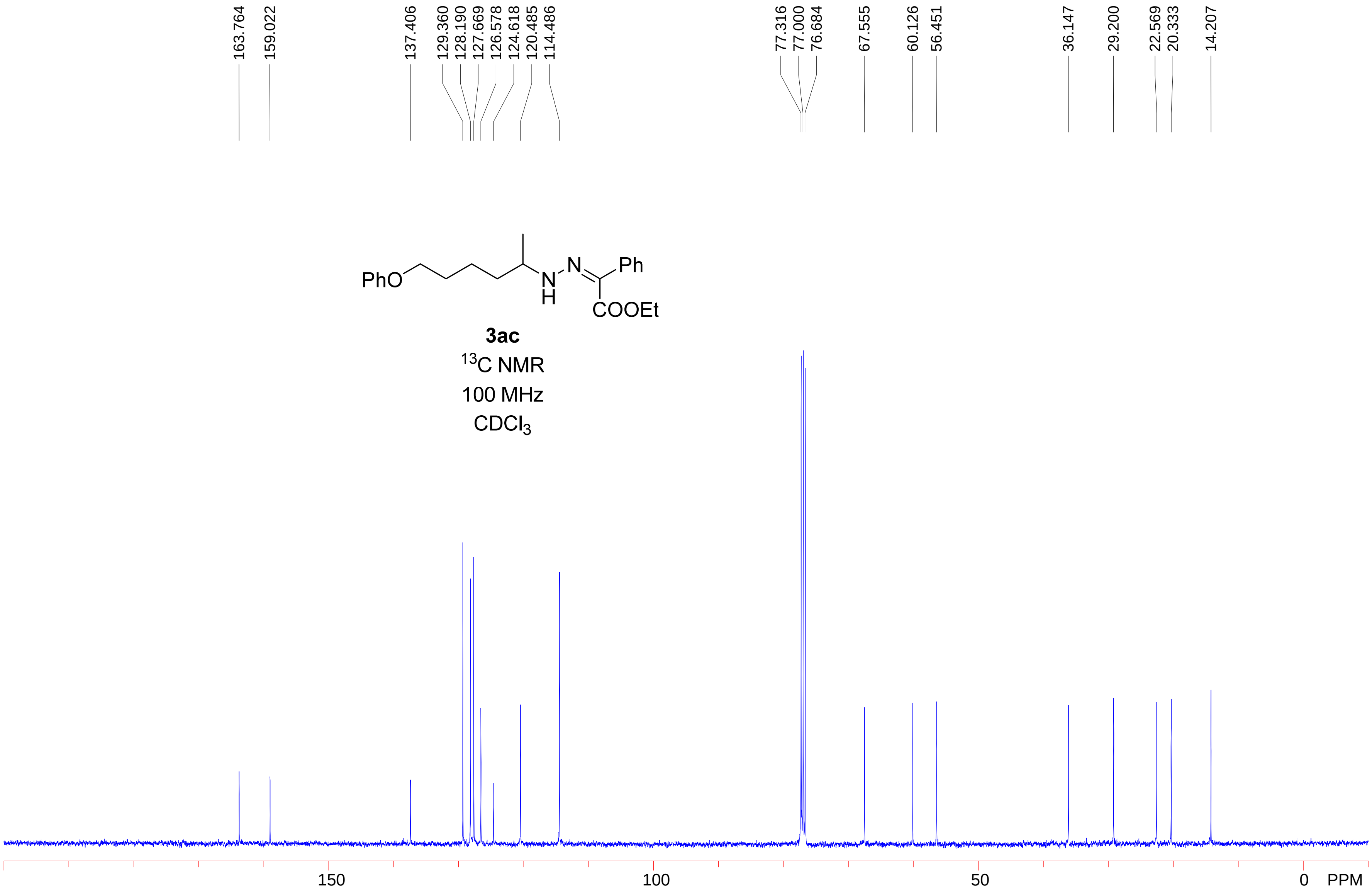

Supplementary Figure 70. ^{13}C NMR spectrum for **3ac**

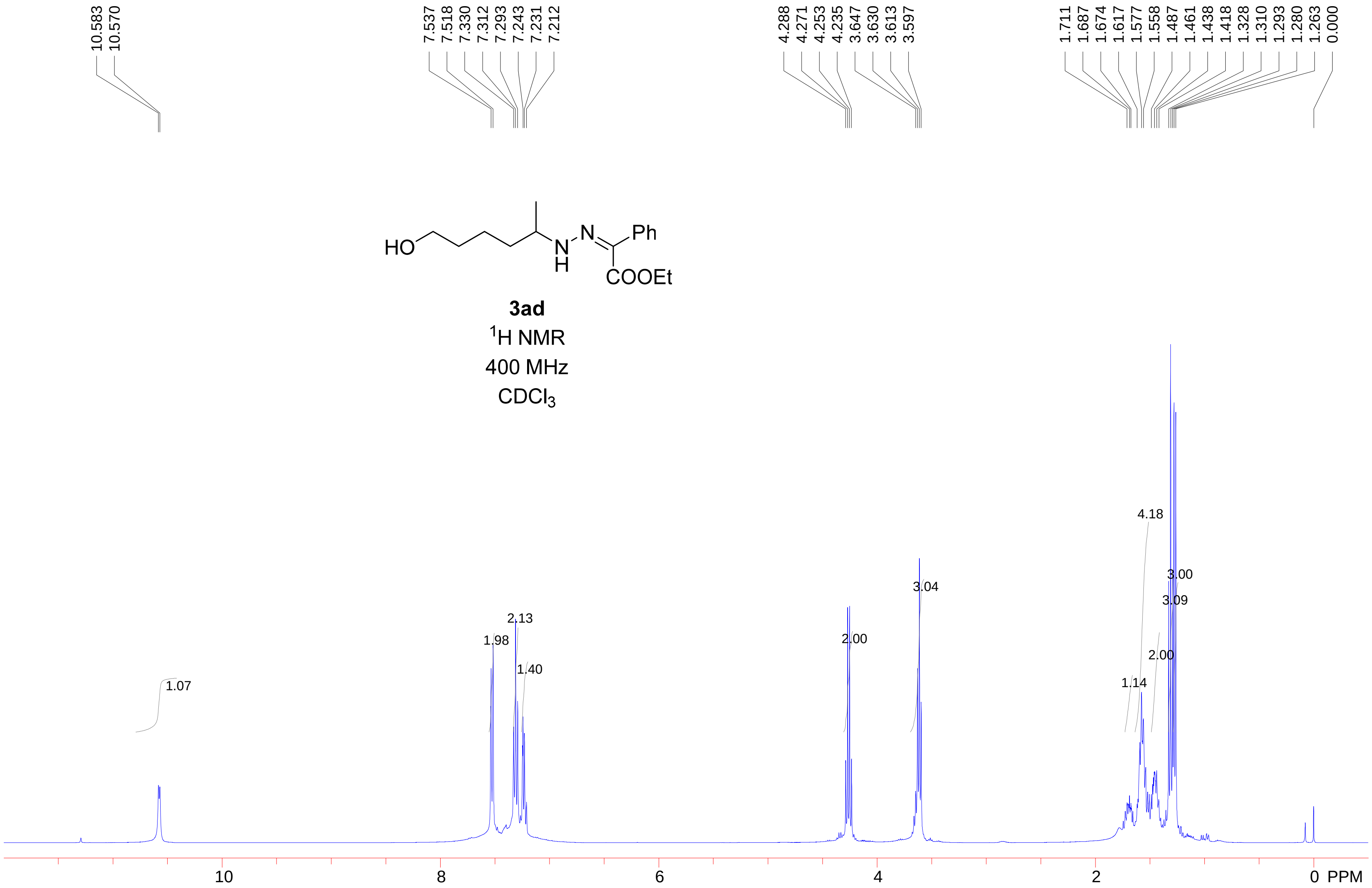

Supplementary Figure 71. ^{1}H NMR spectrum for **3ad**

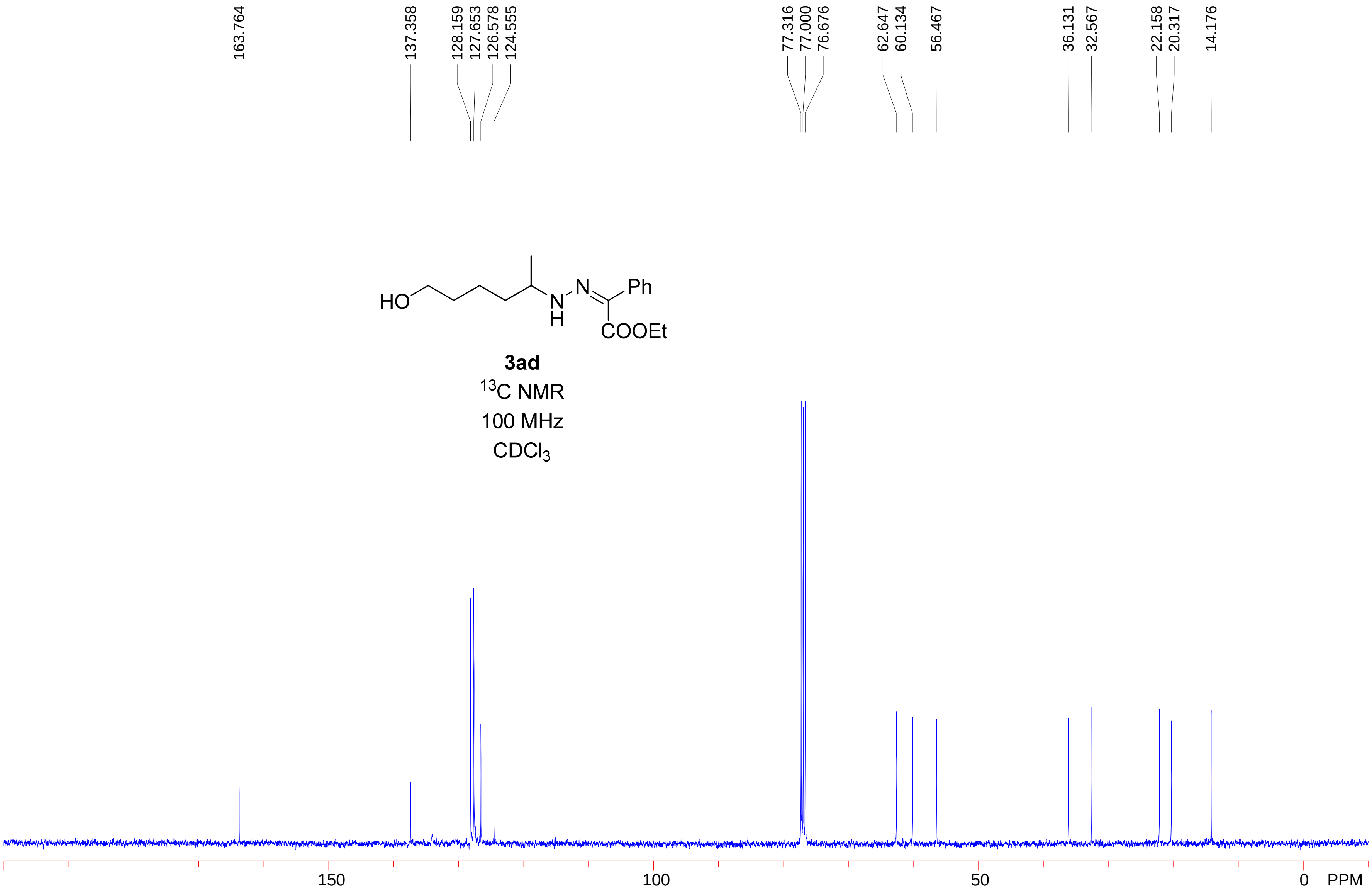

Supplementary Figure 72. ^{13}C NMR spectrum for **3ad**

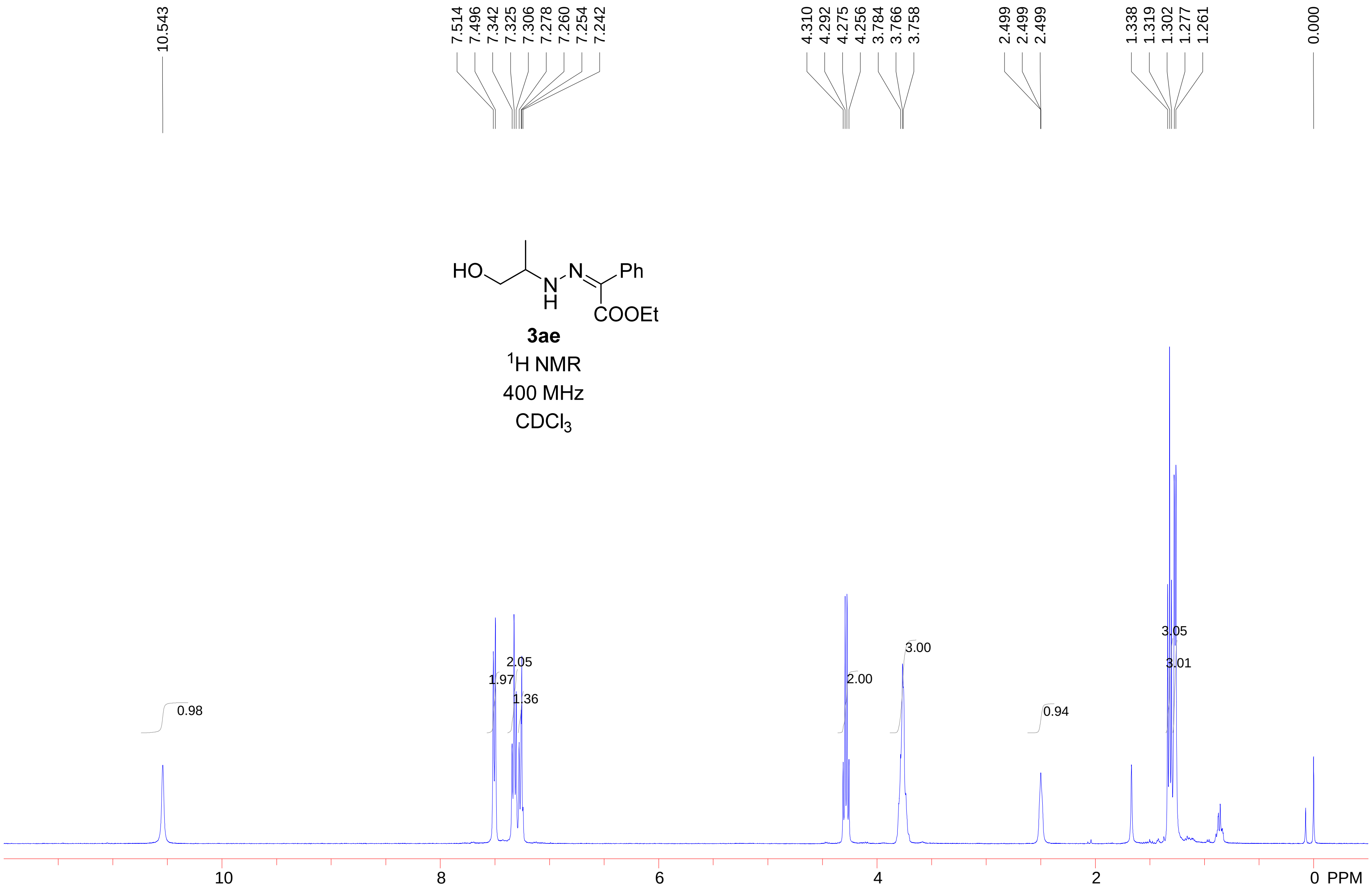

Supplementary Figure 73. ^{1}H NMR spectrum for **3ae**

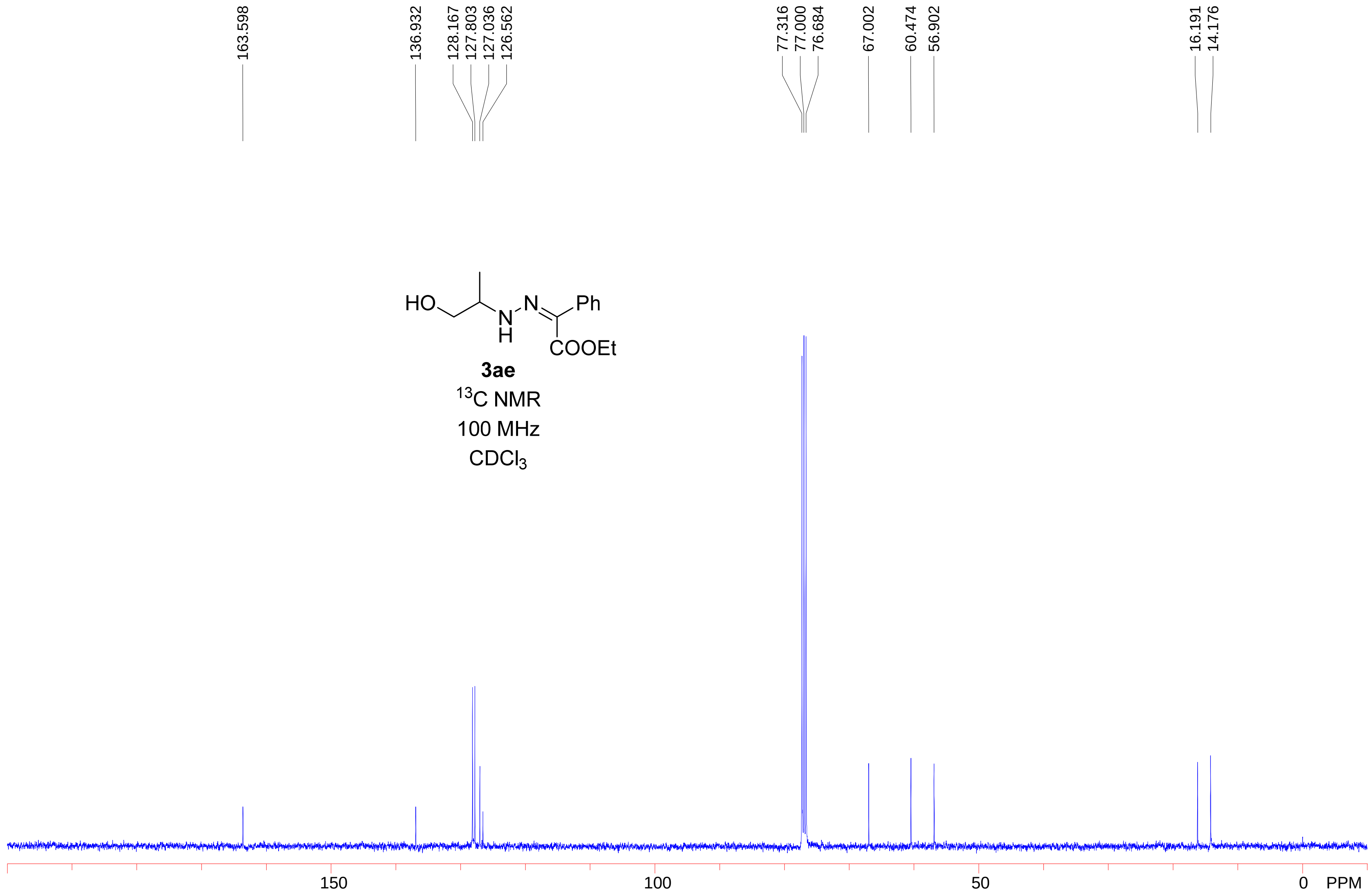

Supplementary Figure 74. ^{13}C NMR spectrum for **3ae**

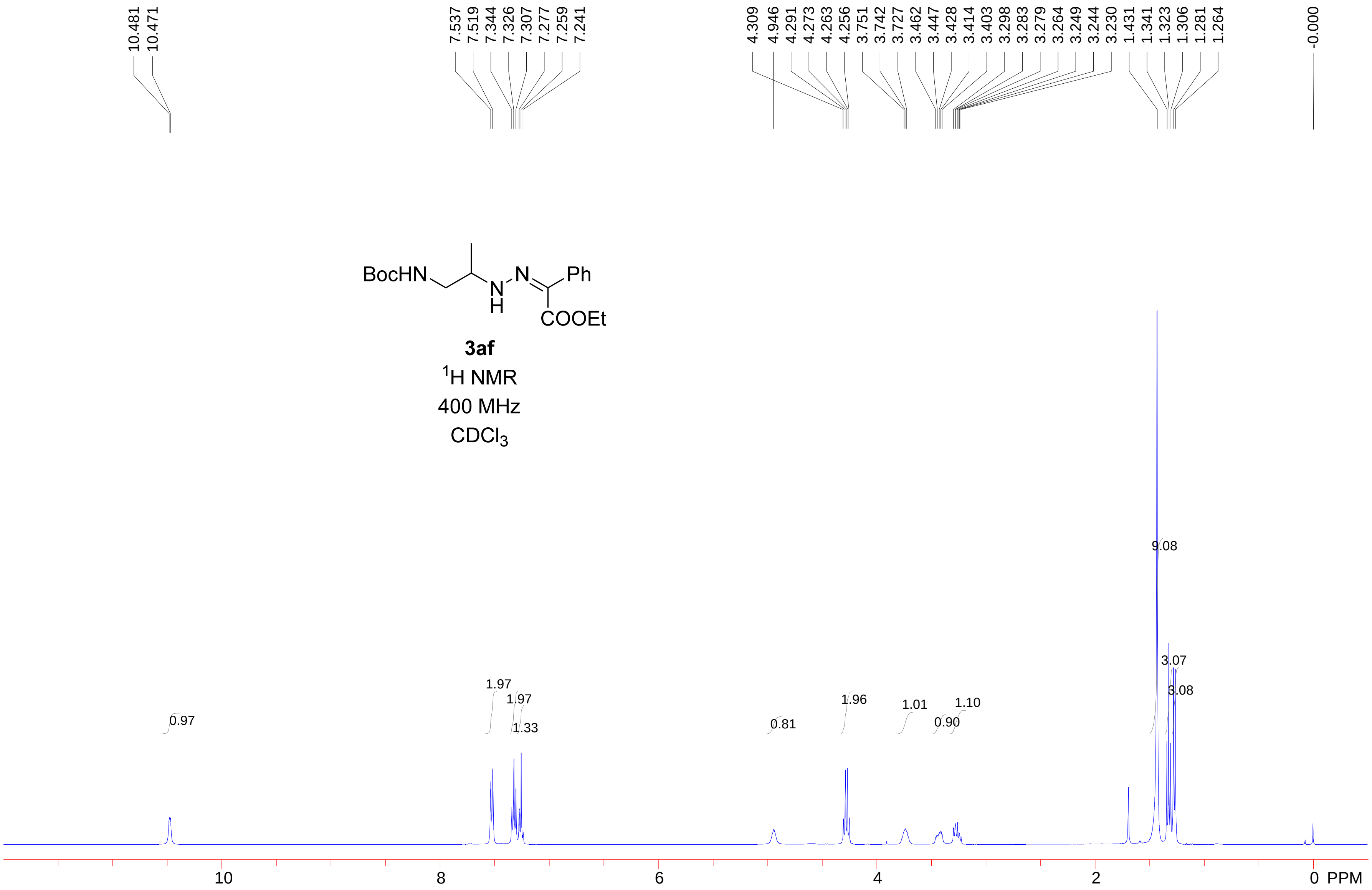

Supplementary Figure 75. ^{1}H NMR spectrum for **3af**

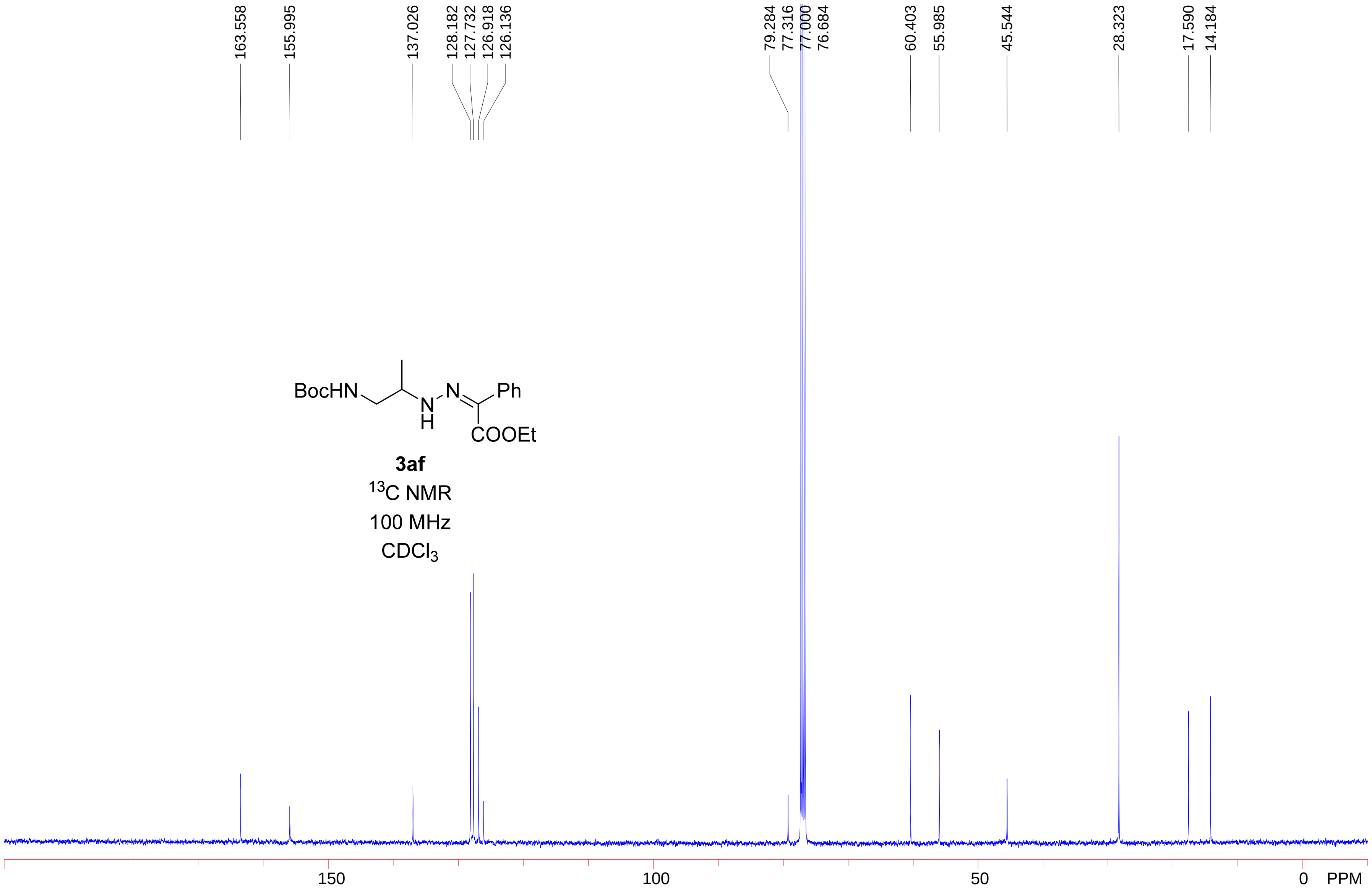

Supplementary Figure 76. ^{13}C NMR spectrum for **3af**

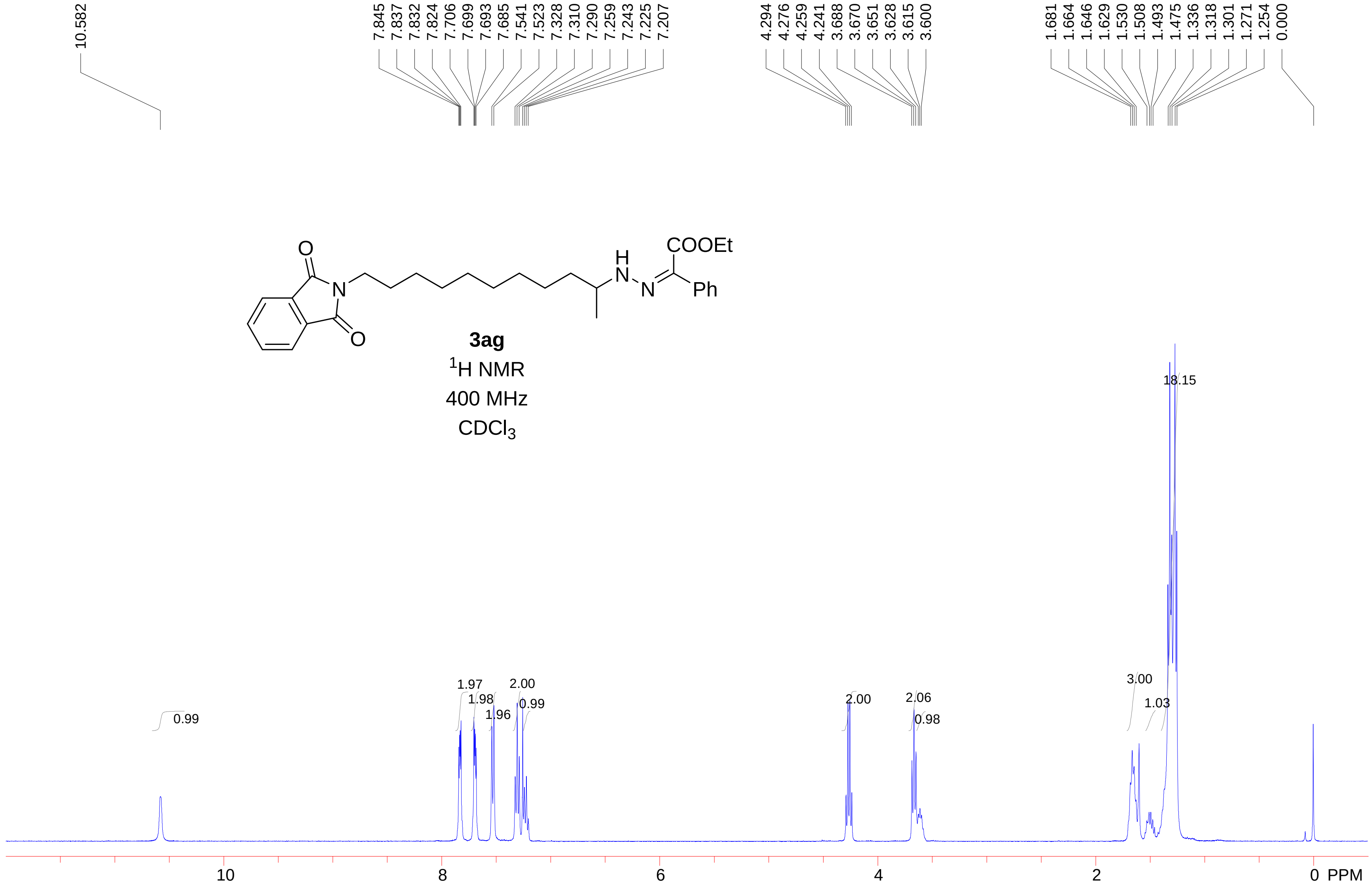

Supplementary Figure 77. ^{1}H NMR spectrum for **3ag**

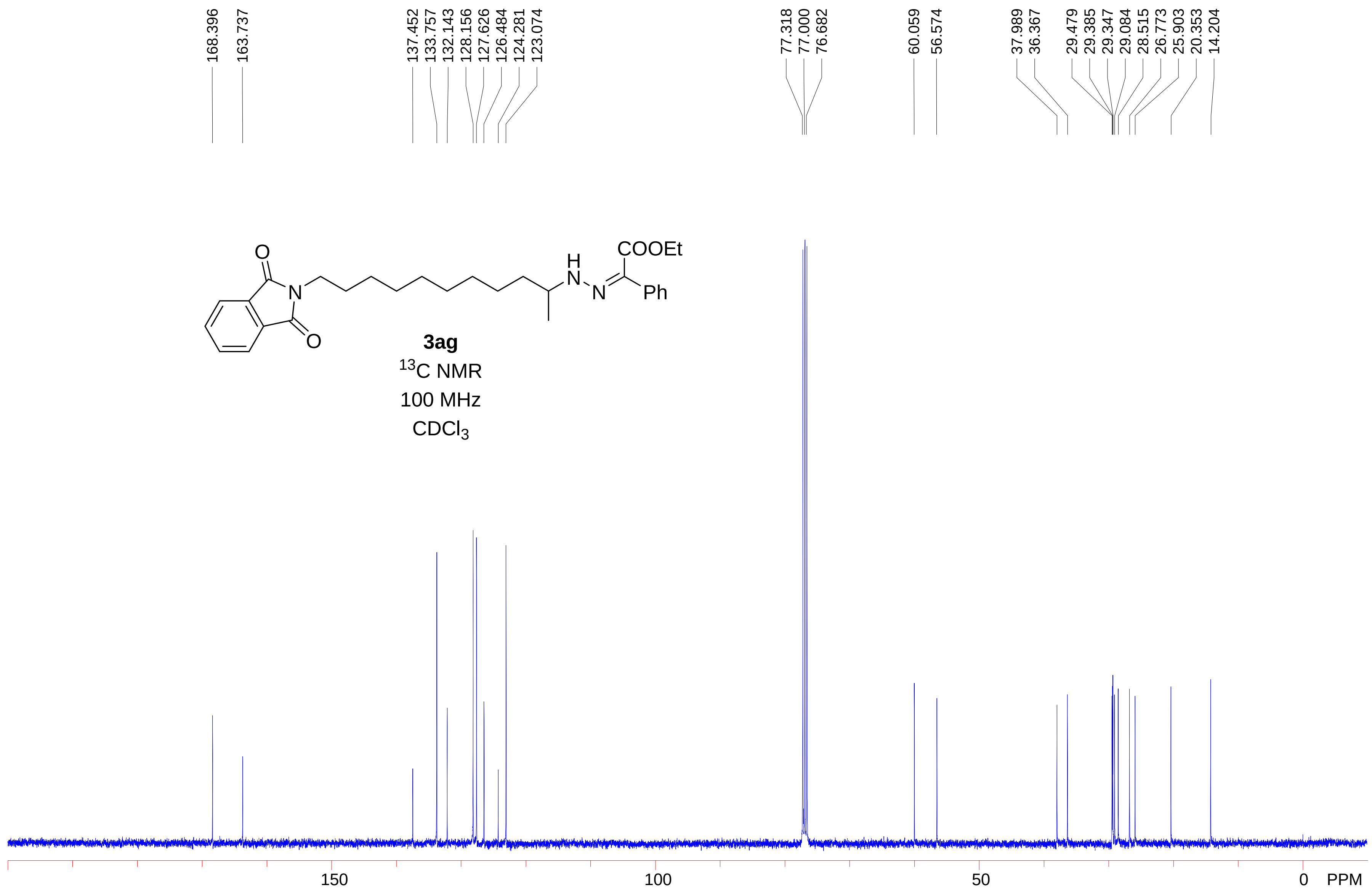

Supplementary Figure 78. ^{13}C NMR spectrum for **3ag**

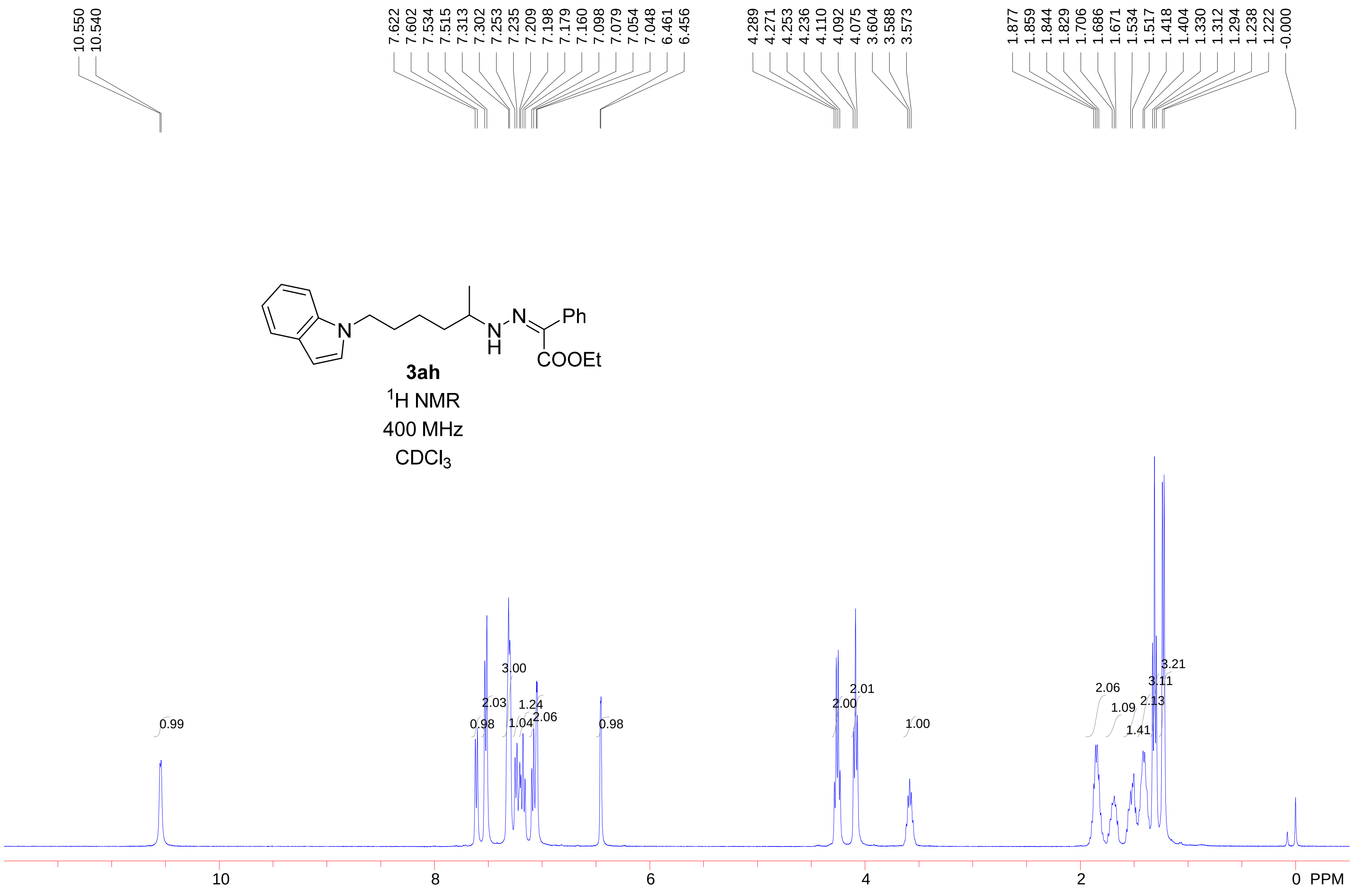

Supplementary Figure 79. ^{1}H NMR spectrum for **3ah**

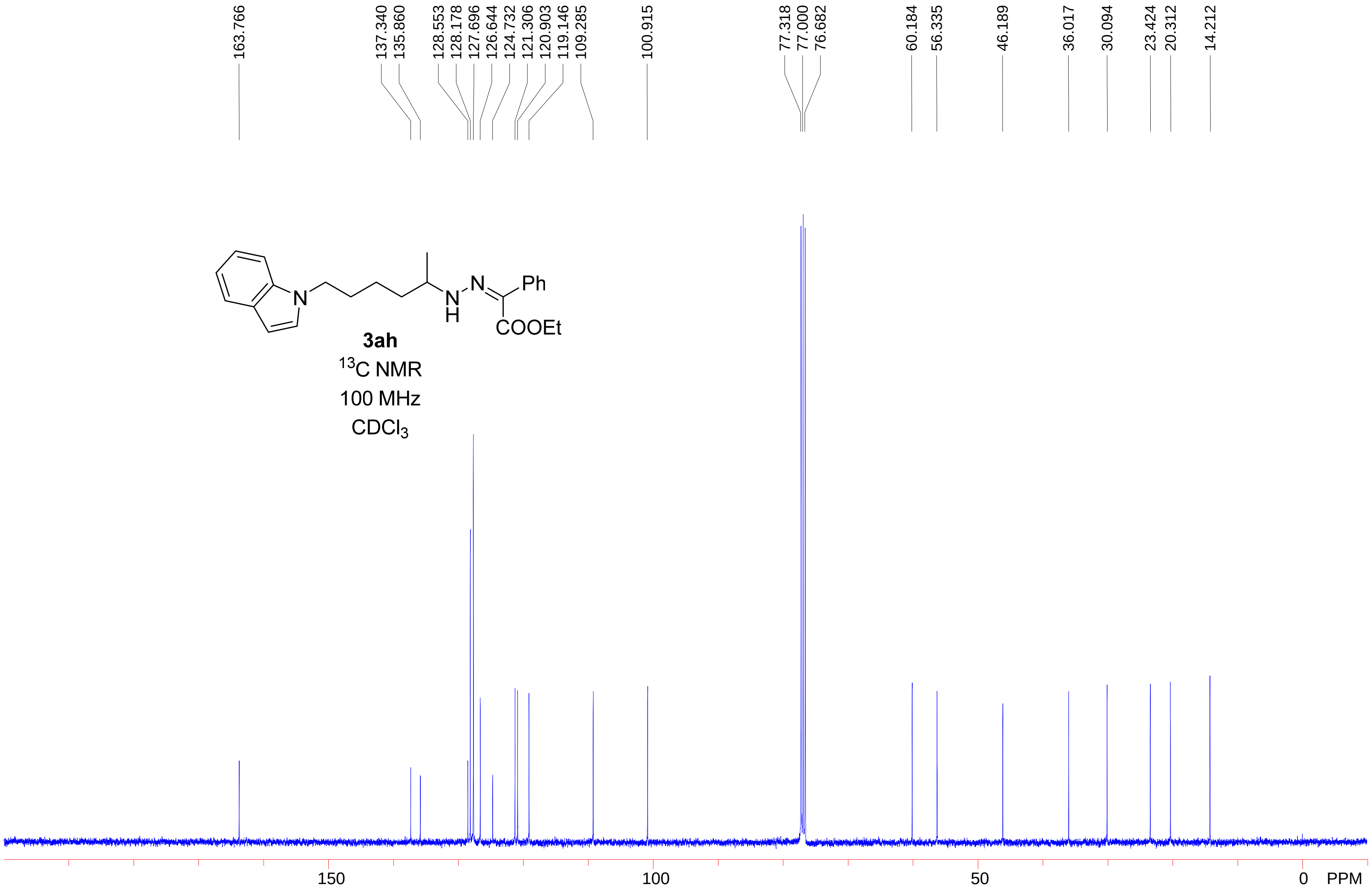

Supplementary Figure 80. ^{13}C NMR spectrum for **3ah**

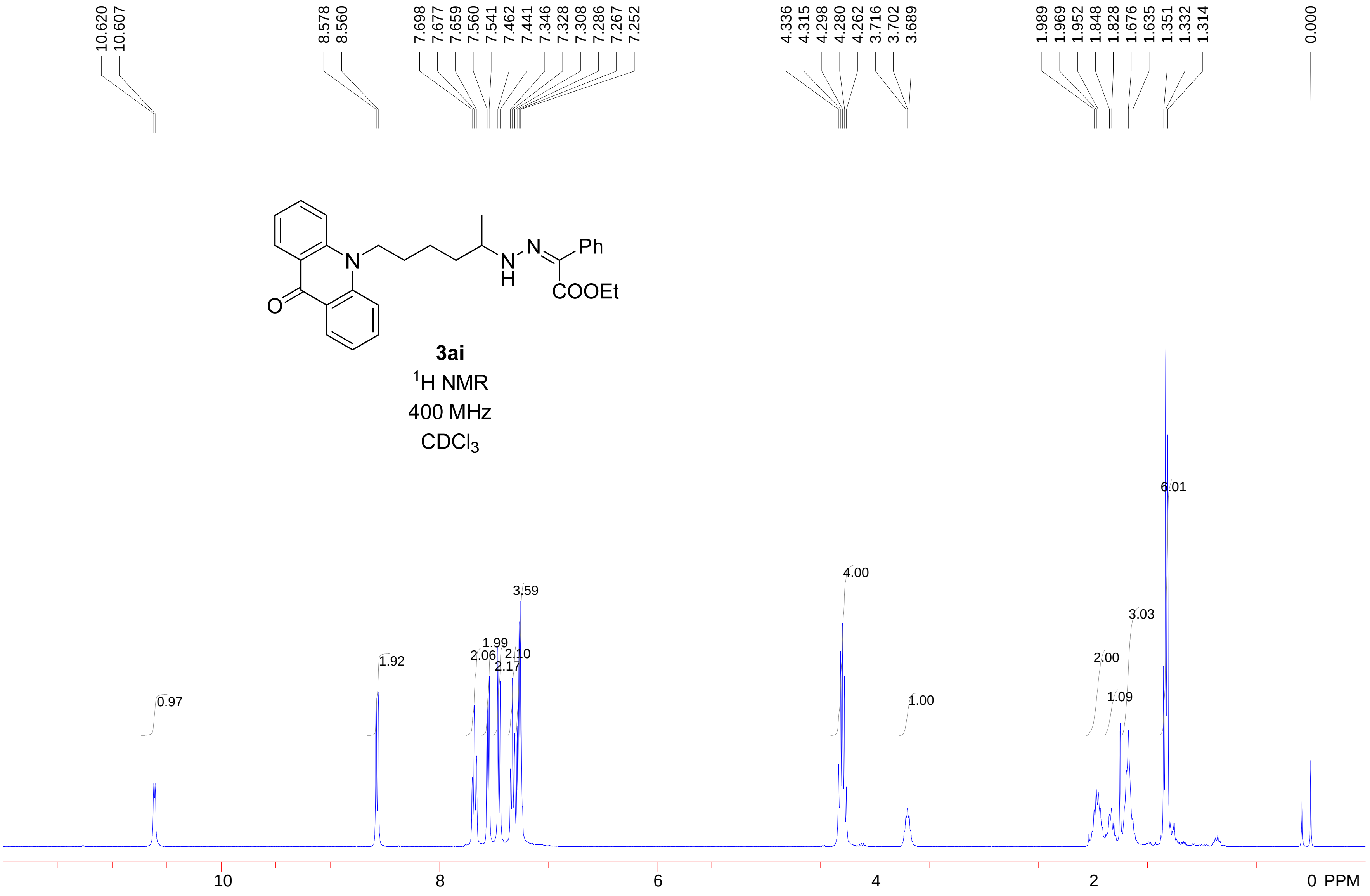

Supplementary Figure 81. ^{1}H NMR spectrum for **3al**

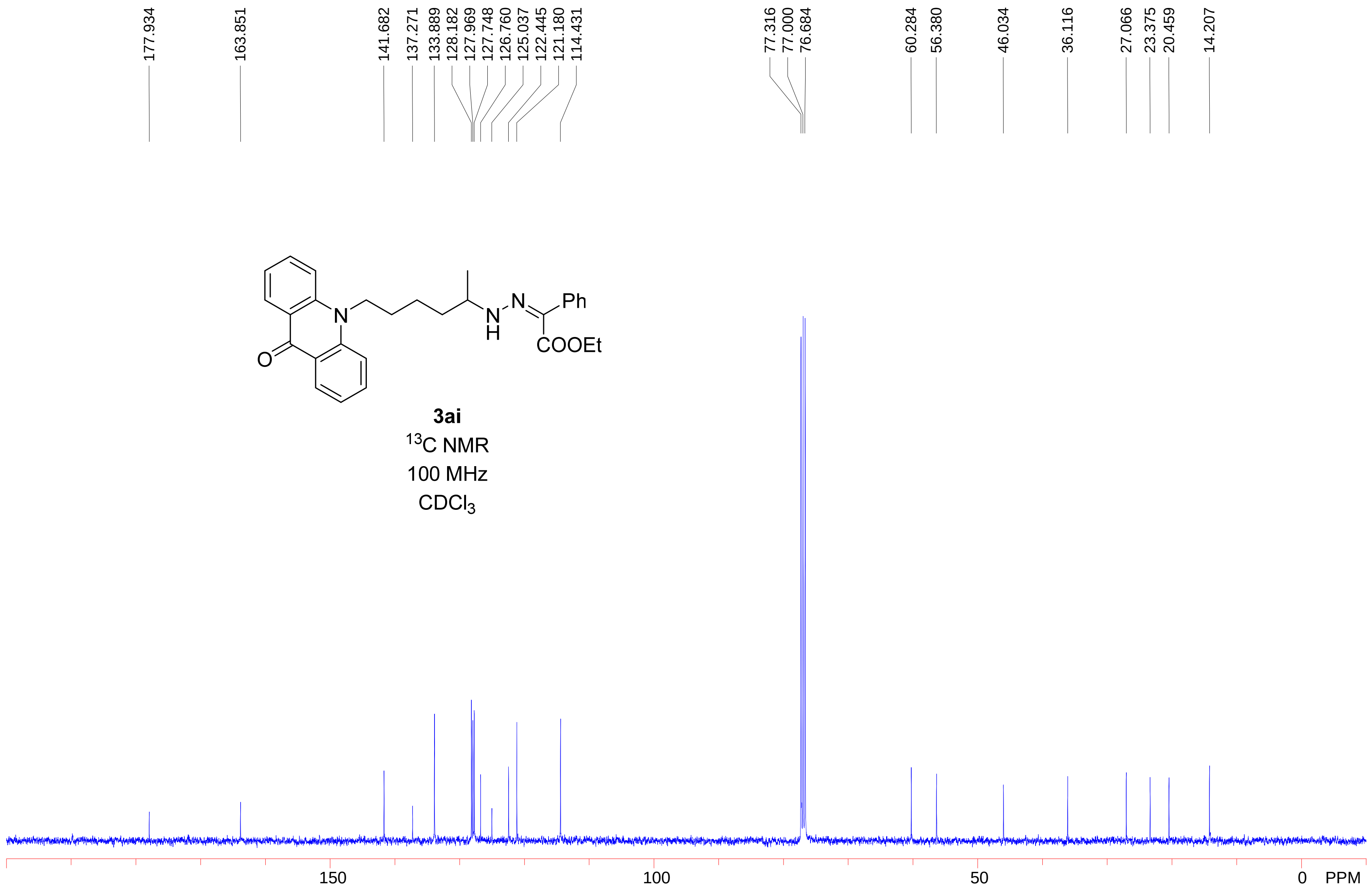

Supplementary Figure 82. ^{13}C NMR spectrum for **3al**

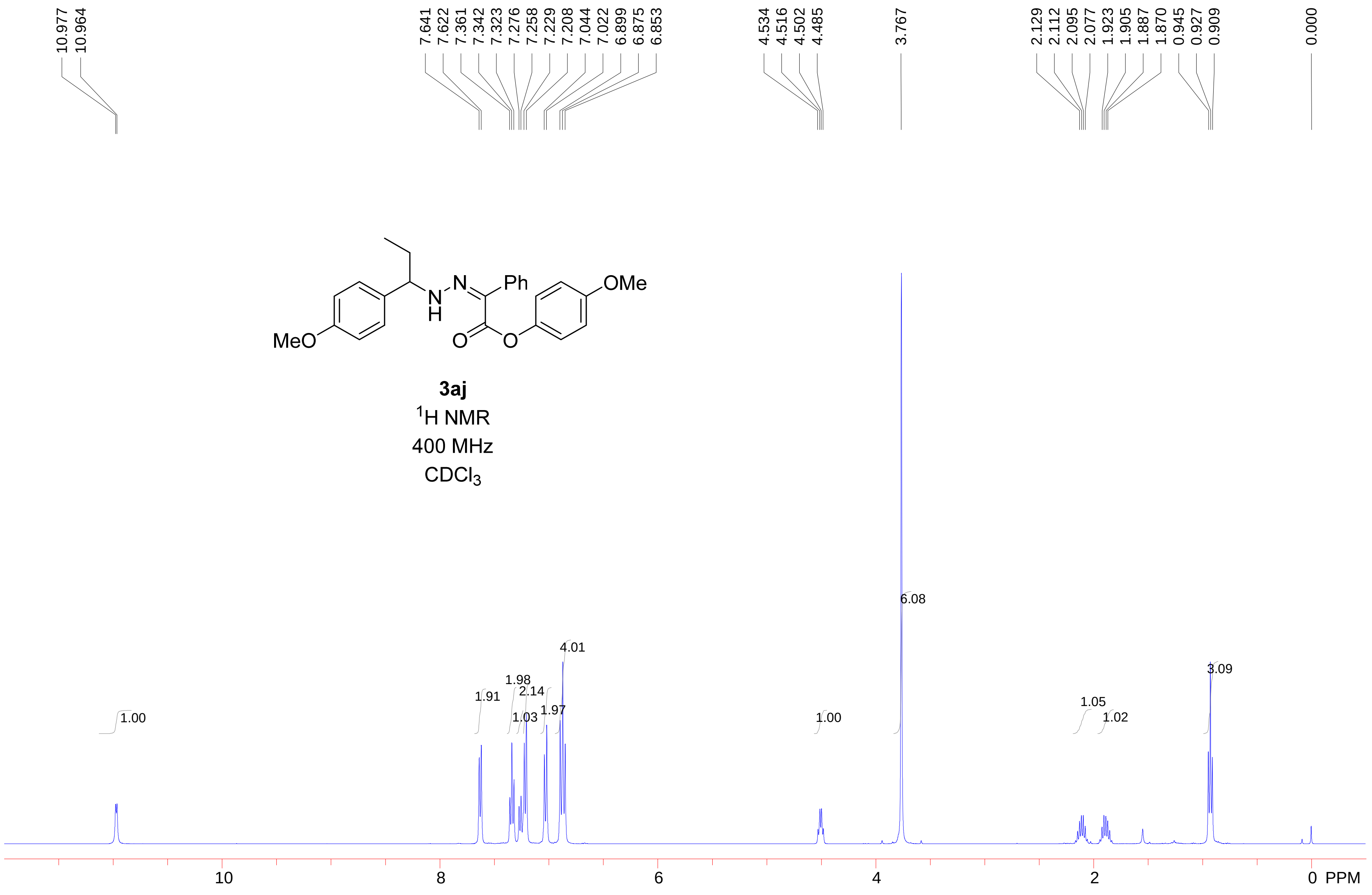

Supplementary Figure 83. ^{1}H NMR spectrum for **3aj**

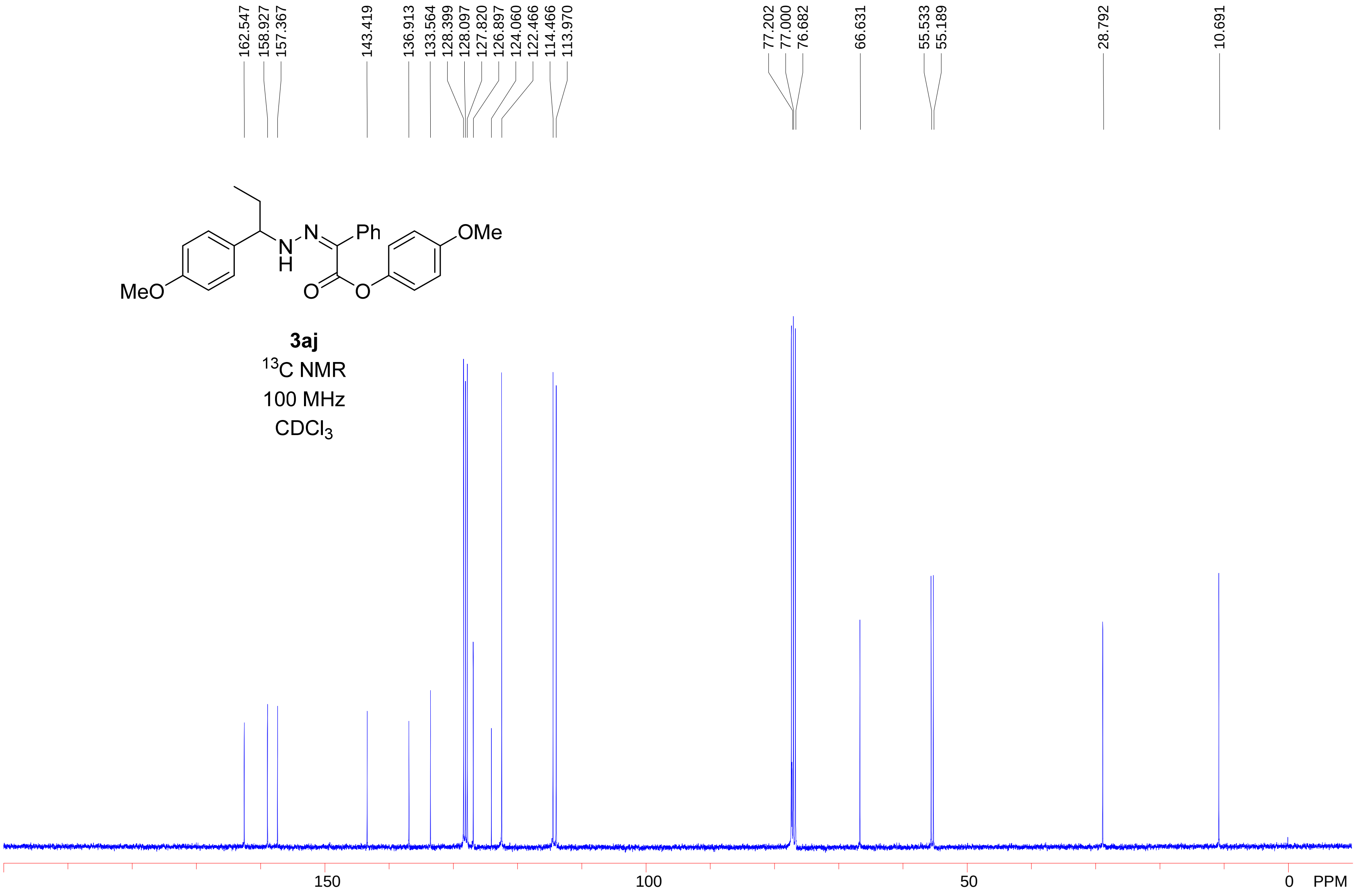

Supplementary Figure 84. ^{13}C NMR spectrum for **3aj**

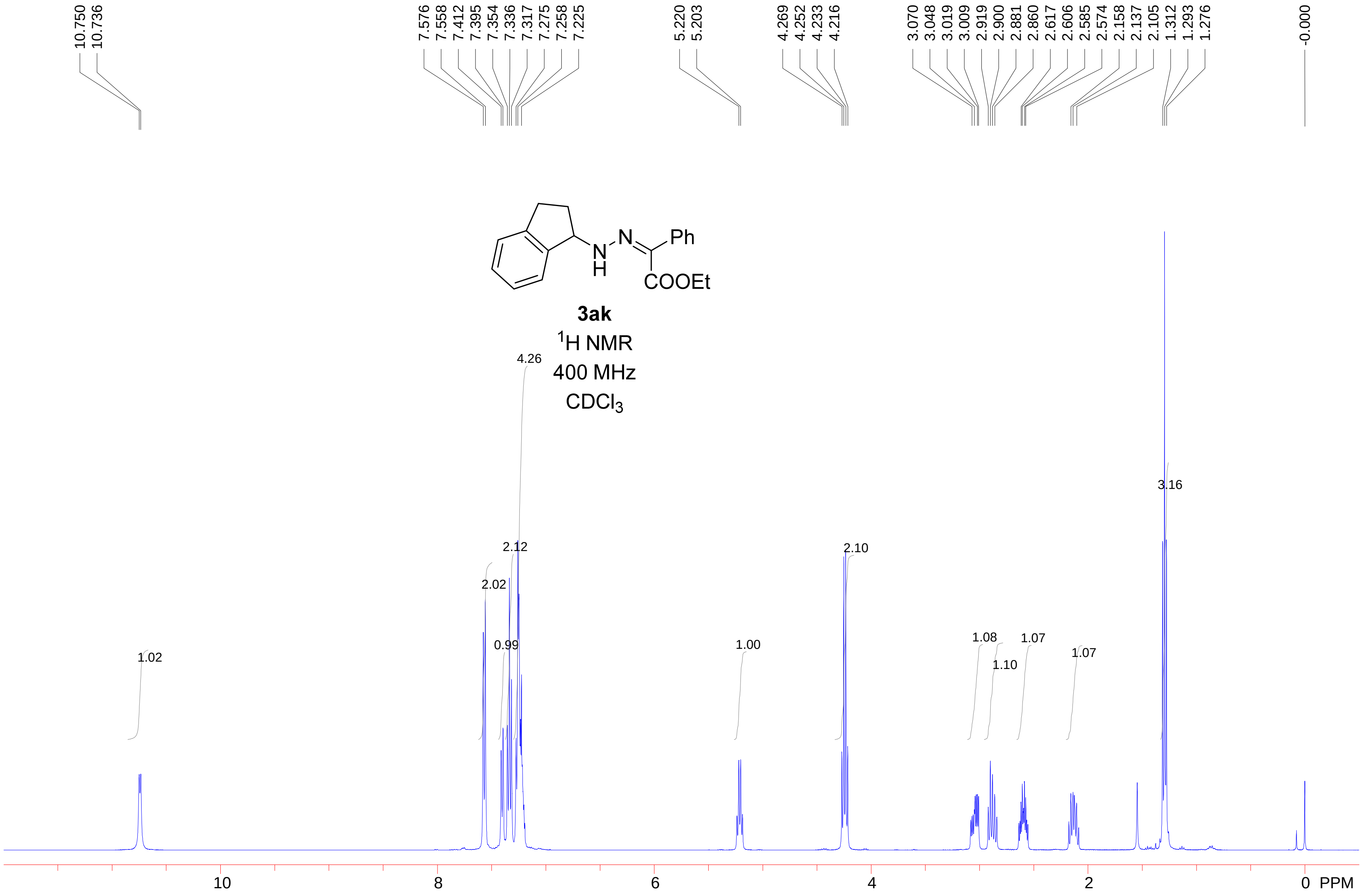

Supplementary Figure 85. ^{1}H NMR spectrum for **3ak**

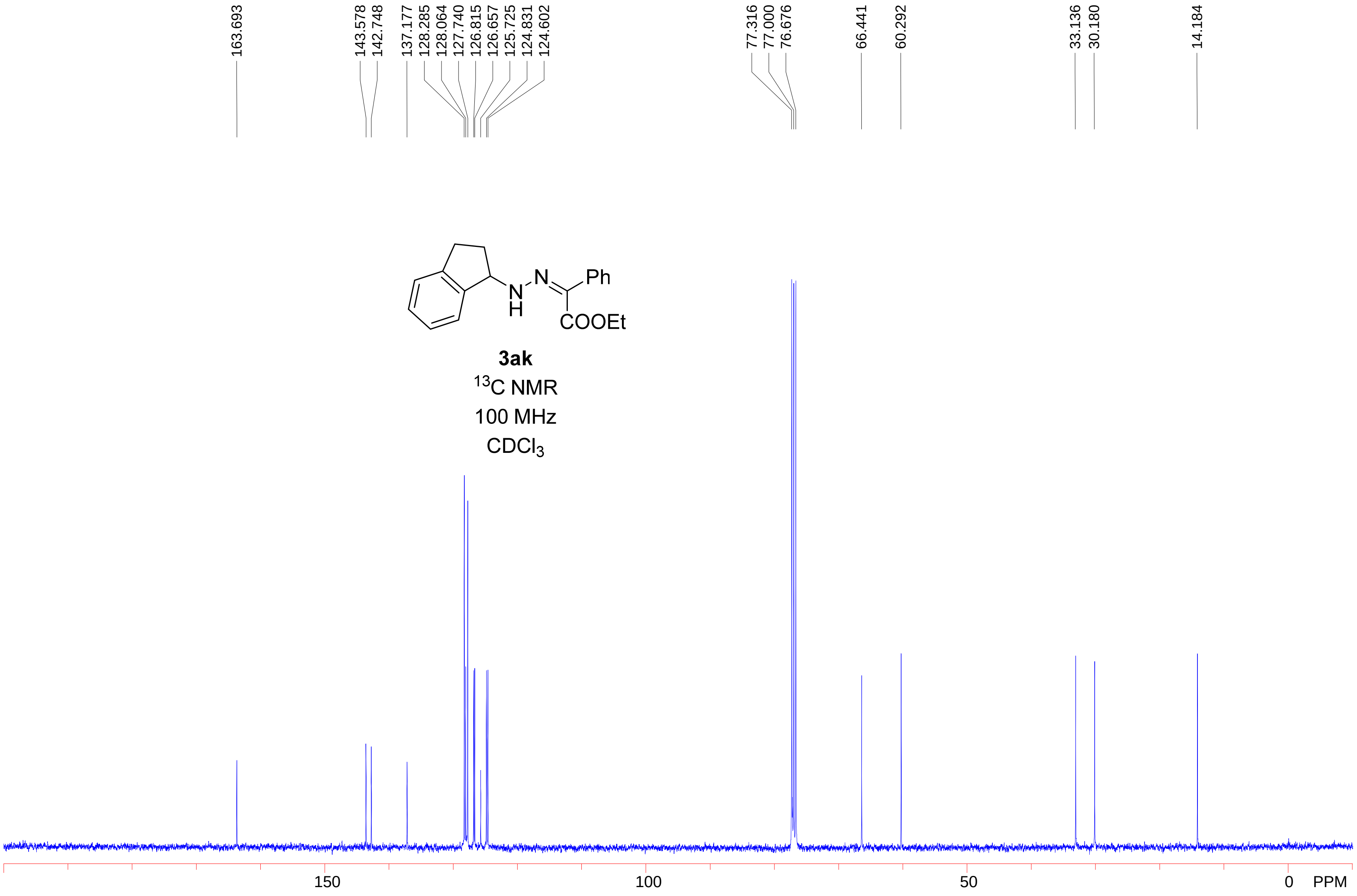

Supplementary Figure 86. ^{13}C NMR spectrum for **3ak**

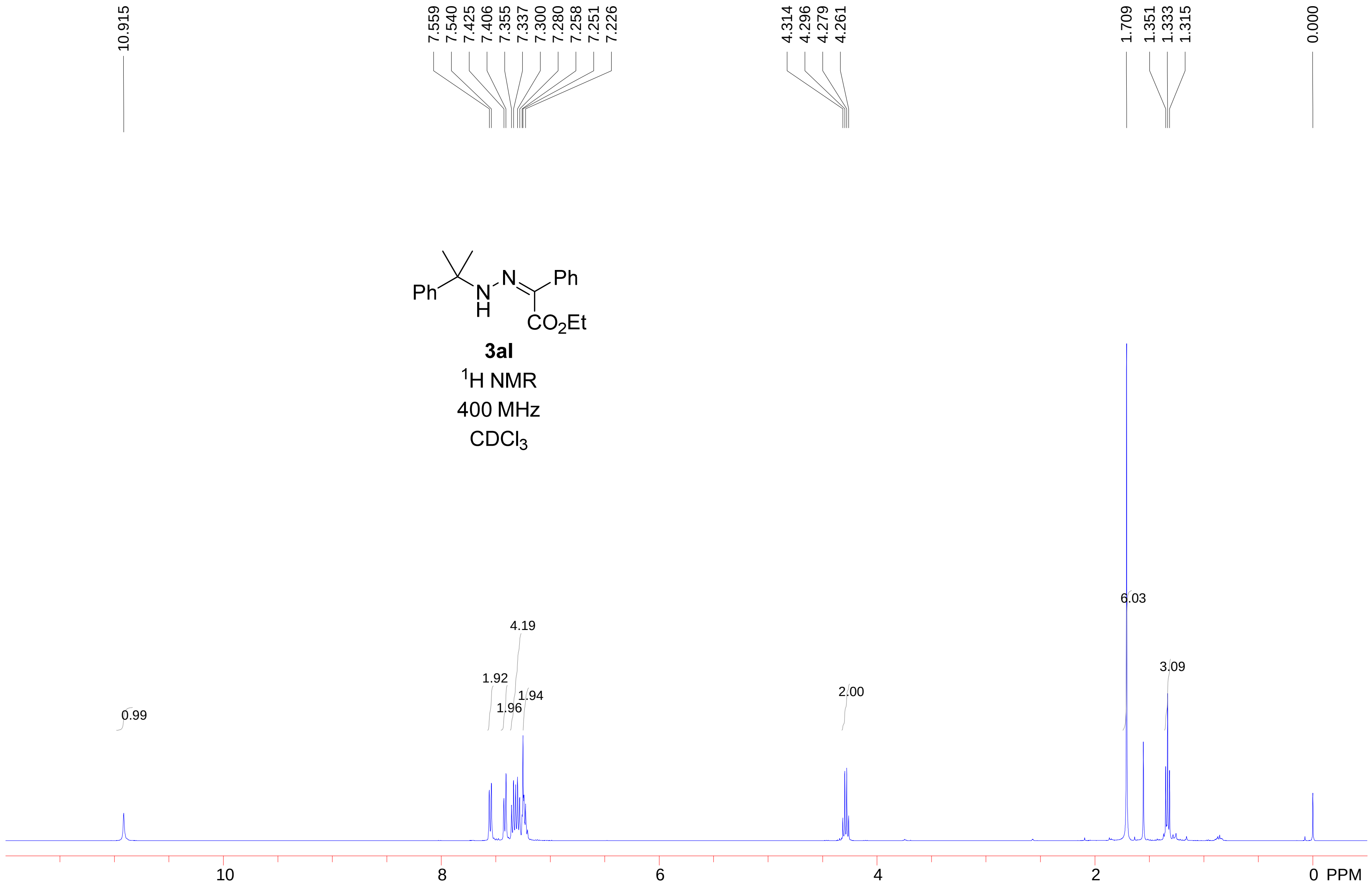

Supplementary Figure 87. ^{1}H NMR spectrum for **3al**

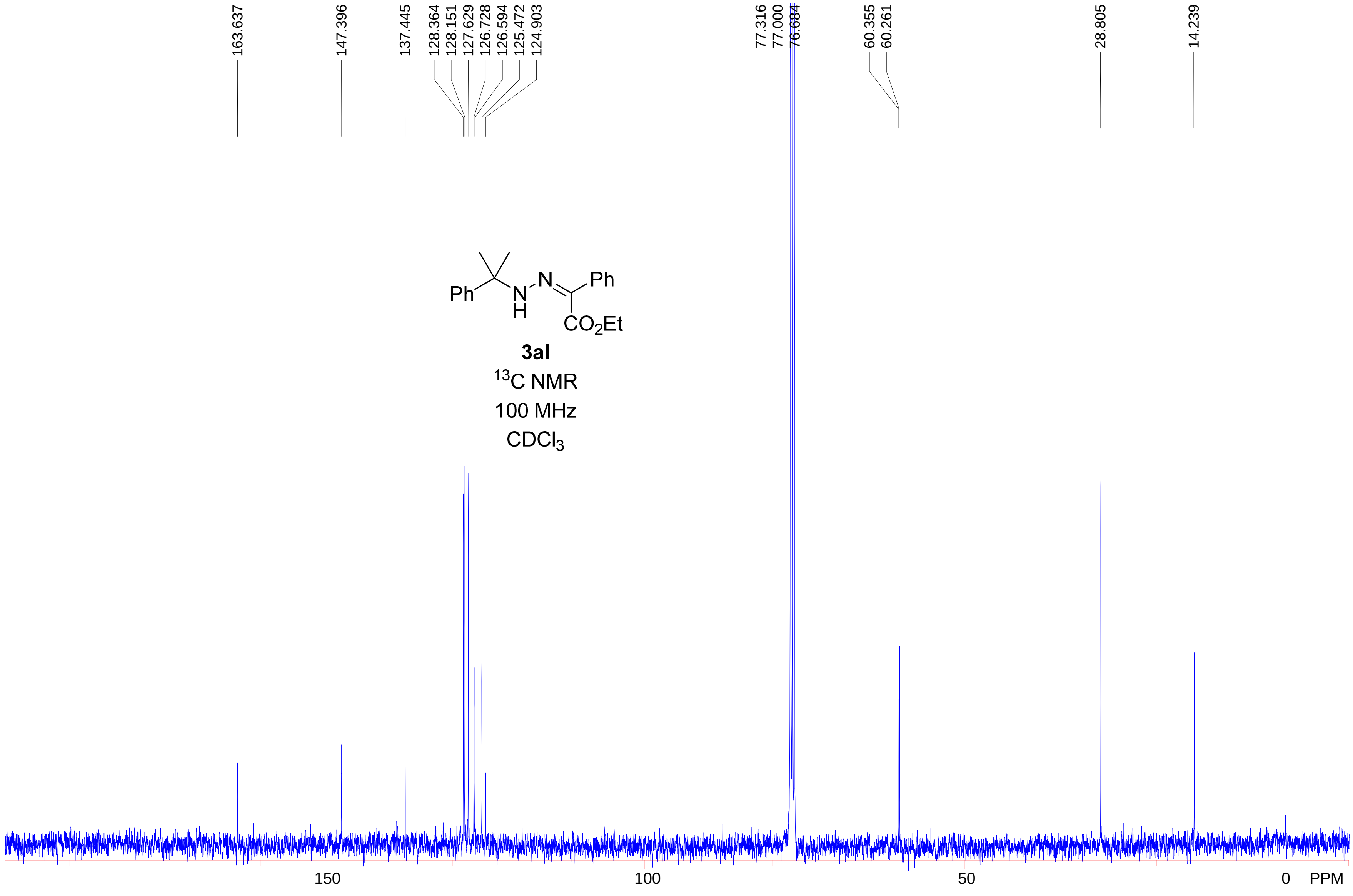

Supplementary Figure 88. ^{13}C NMR spectrum for **3al**

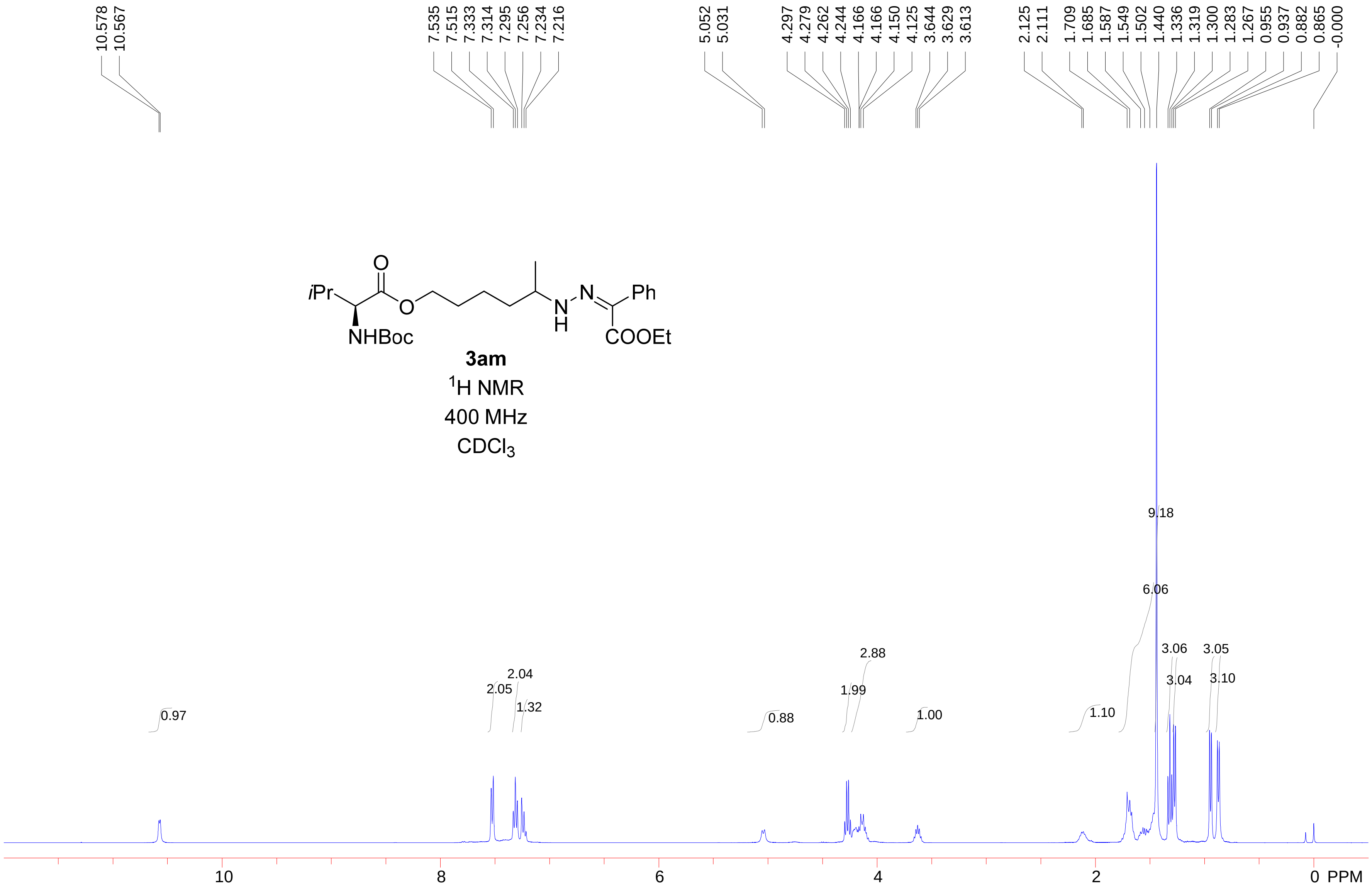

Supplementary Figure 89. ^{1}H NMR spectrum for **3am**

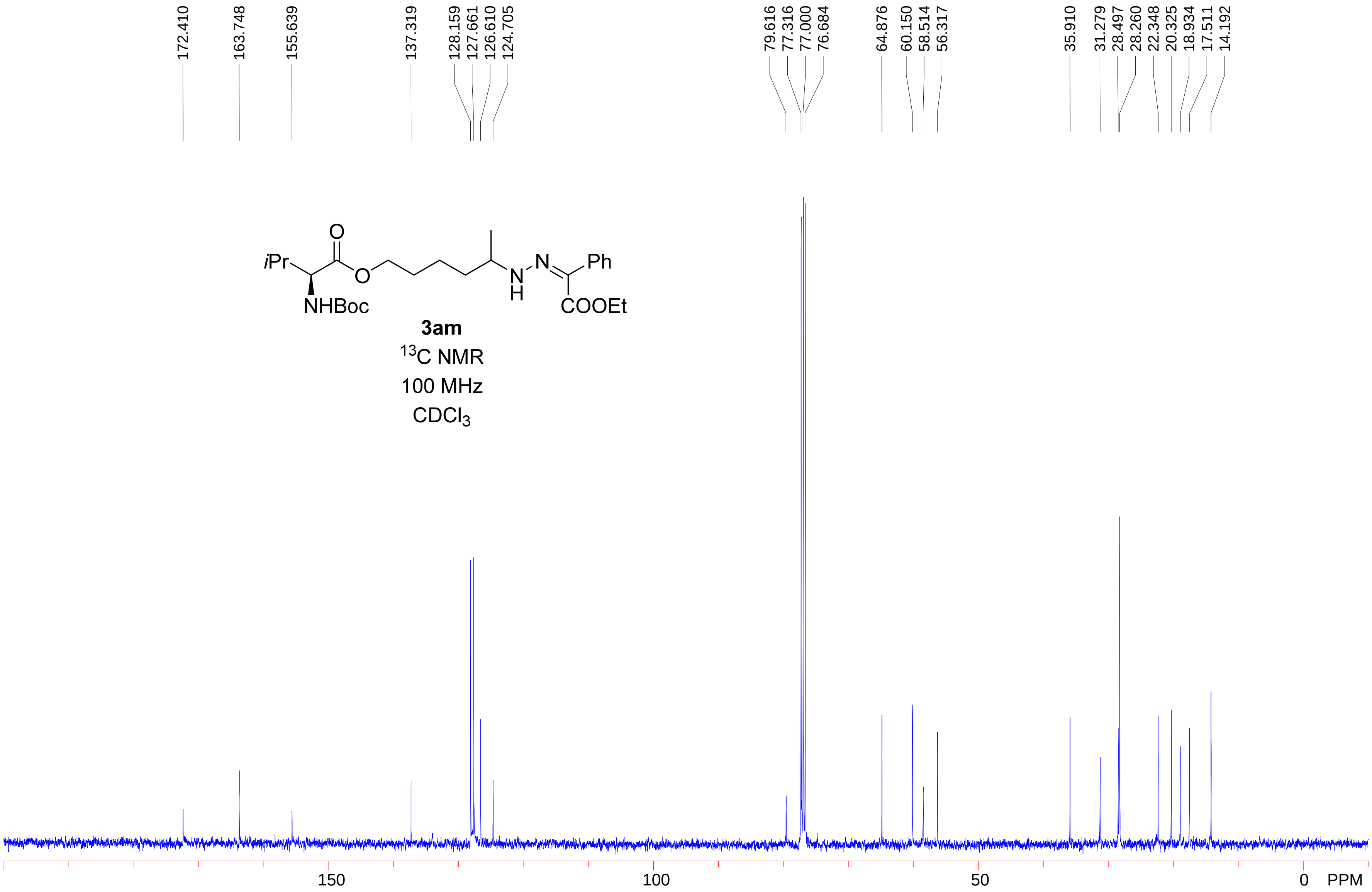

Supplementary Figure 90. ^{13}C NMR spectrum for **3am**

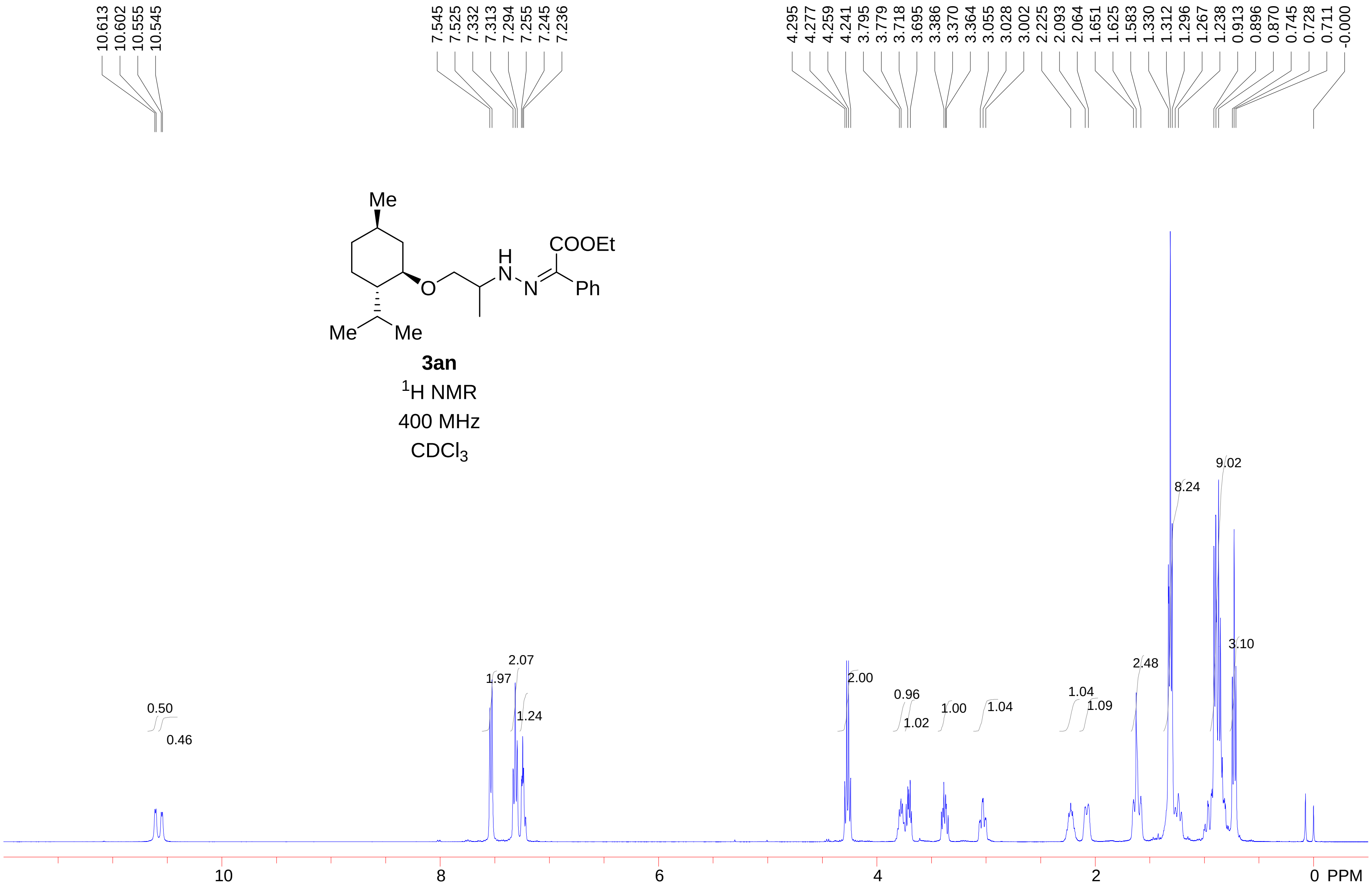

Supplementary Figure 91. ^{1}H NMR spectrum for **3an**

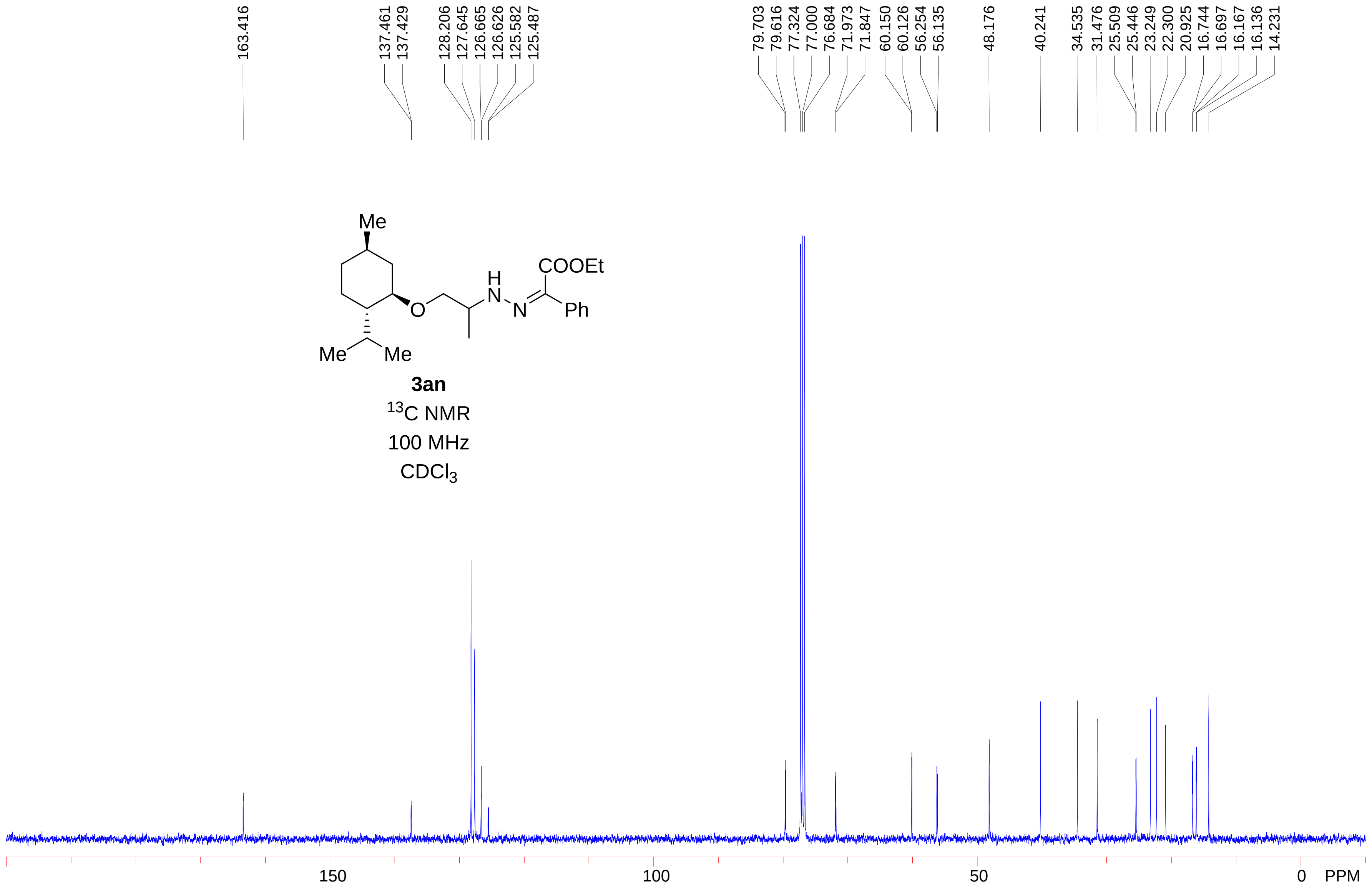

Supplementary Figure 92. ^{13}C NMR spectrum for **3an**

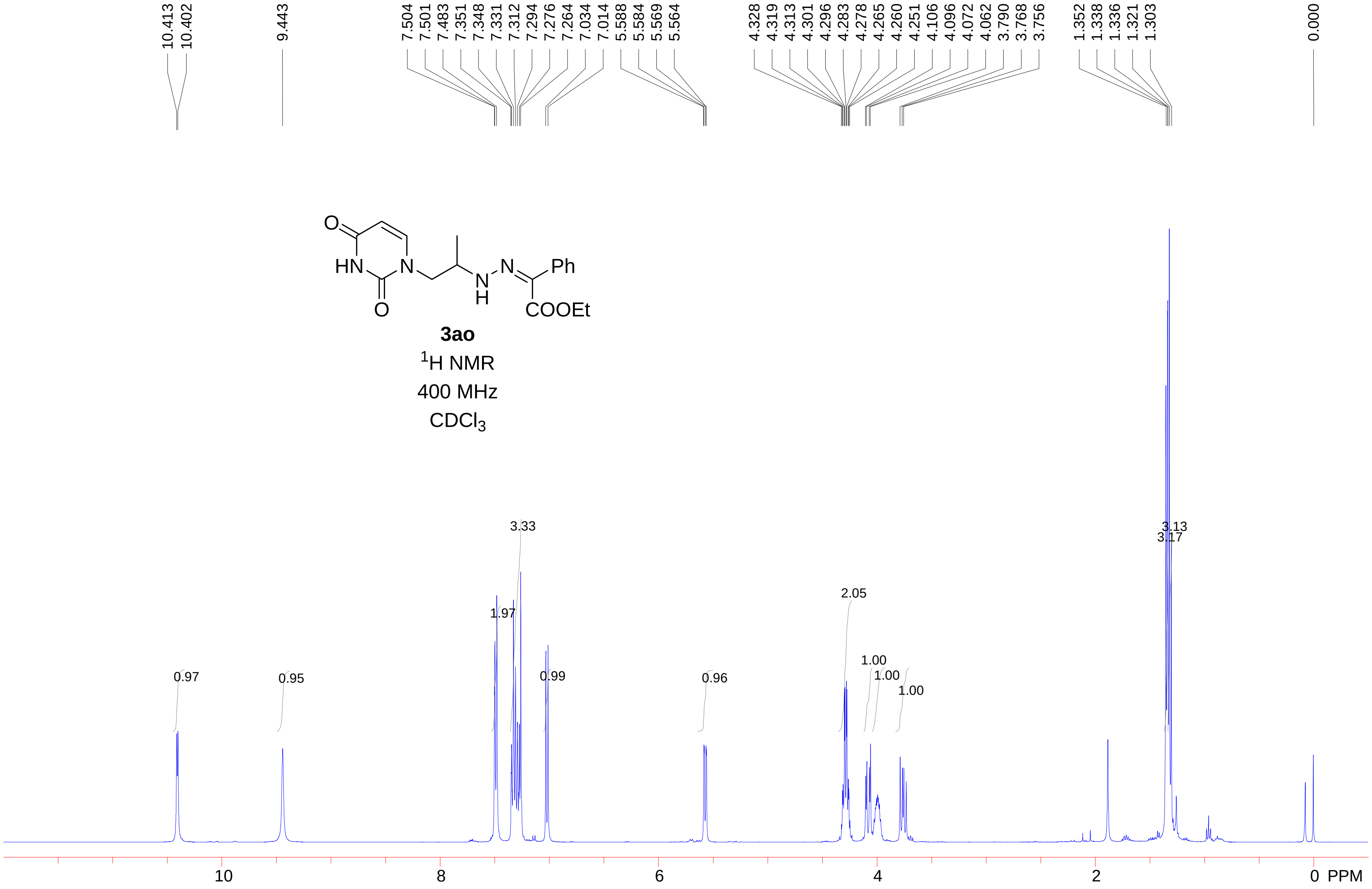

Supplementary Figure 93. ^{1}H NMR spectrum for **3ao**

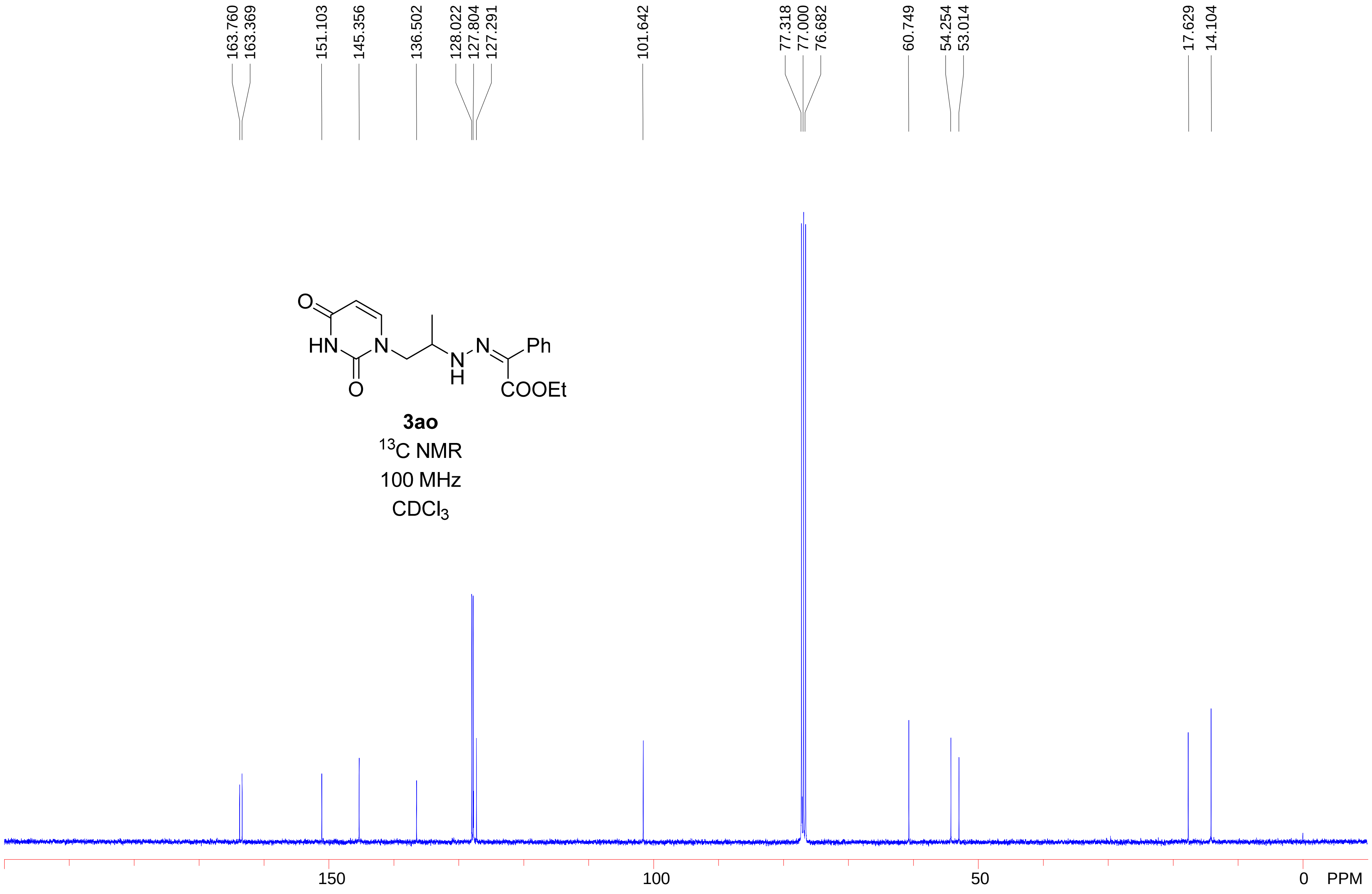

Supplementary Figure 94. ^{13}C NMR spectrum for **3ao**

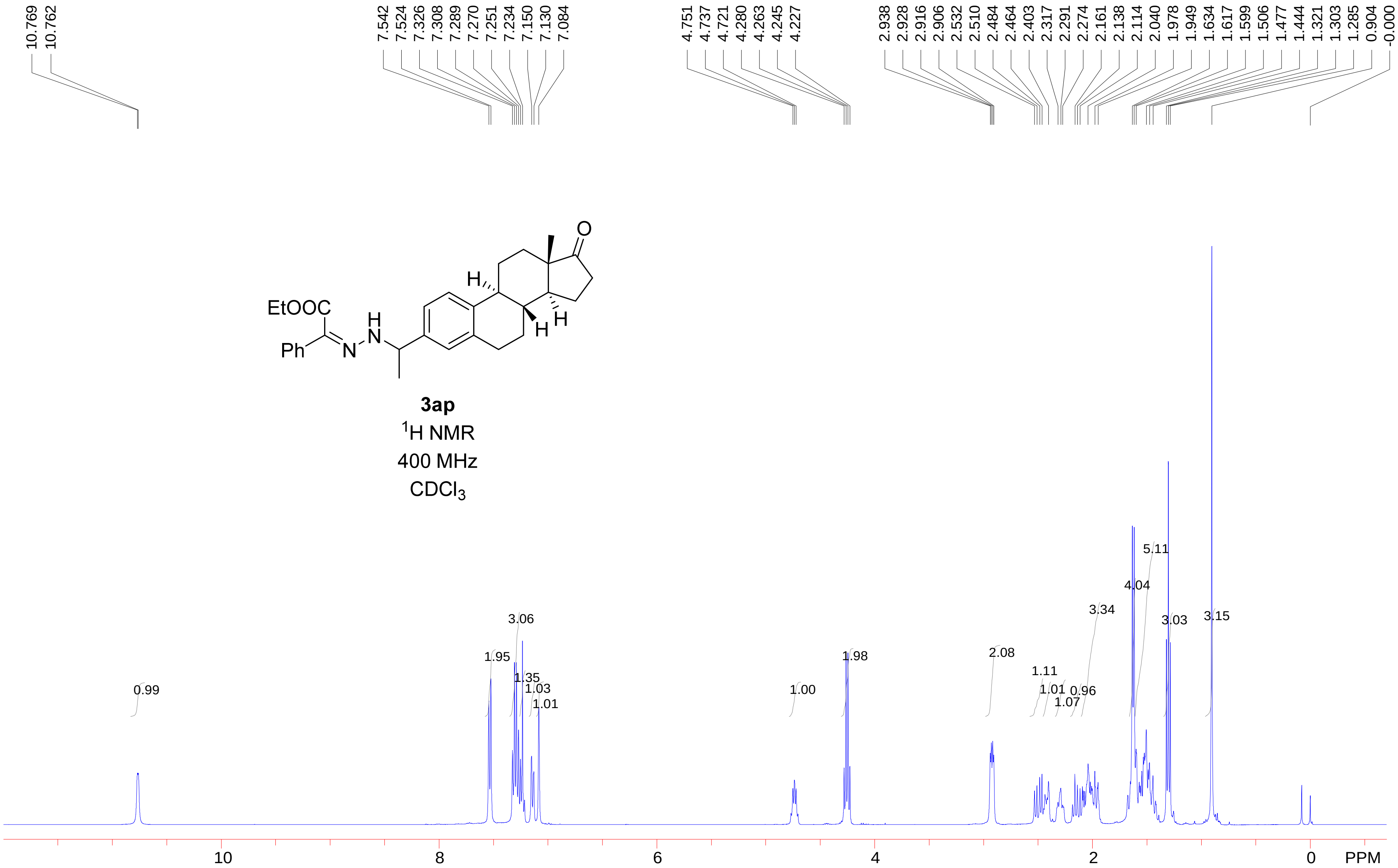

Supplementary Figure 95. ^{1}H NMR spectrum for **3ap**

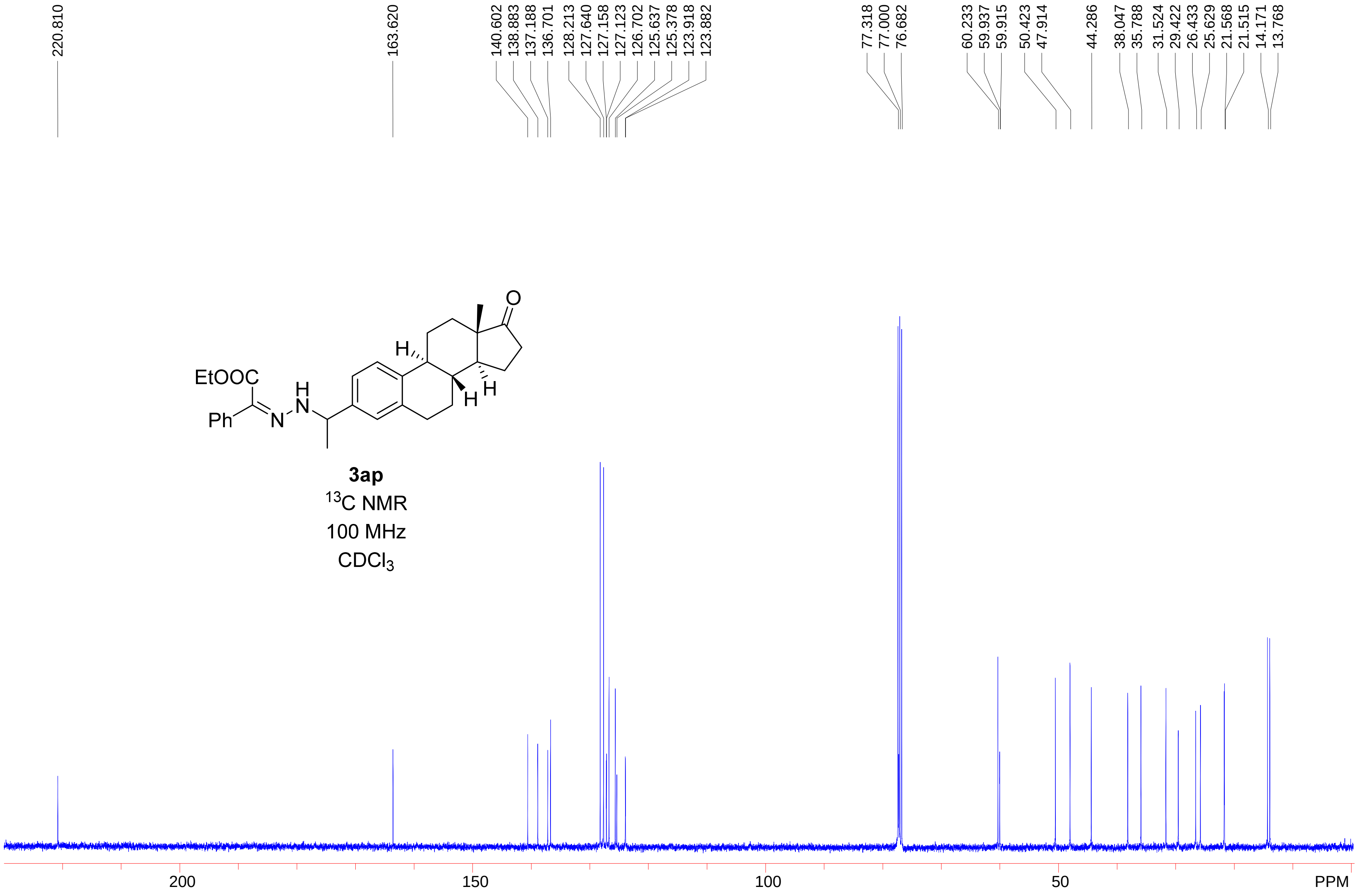

Supplementary Figure 96. ^{13}C NMR spectrum for **3ap**

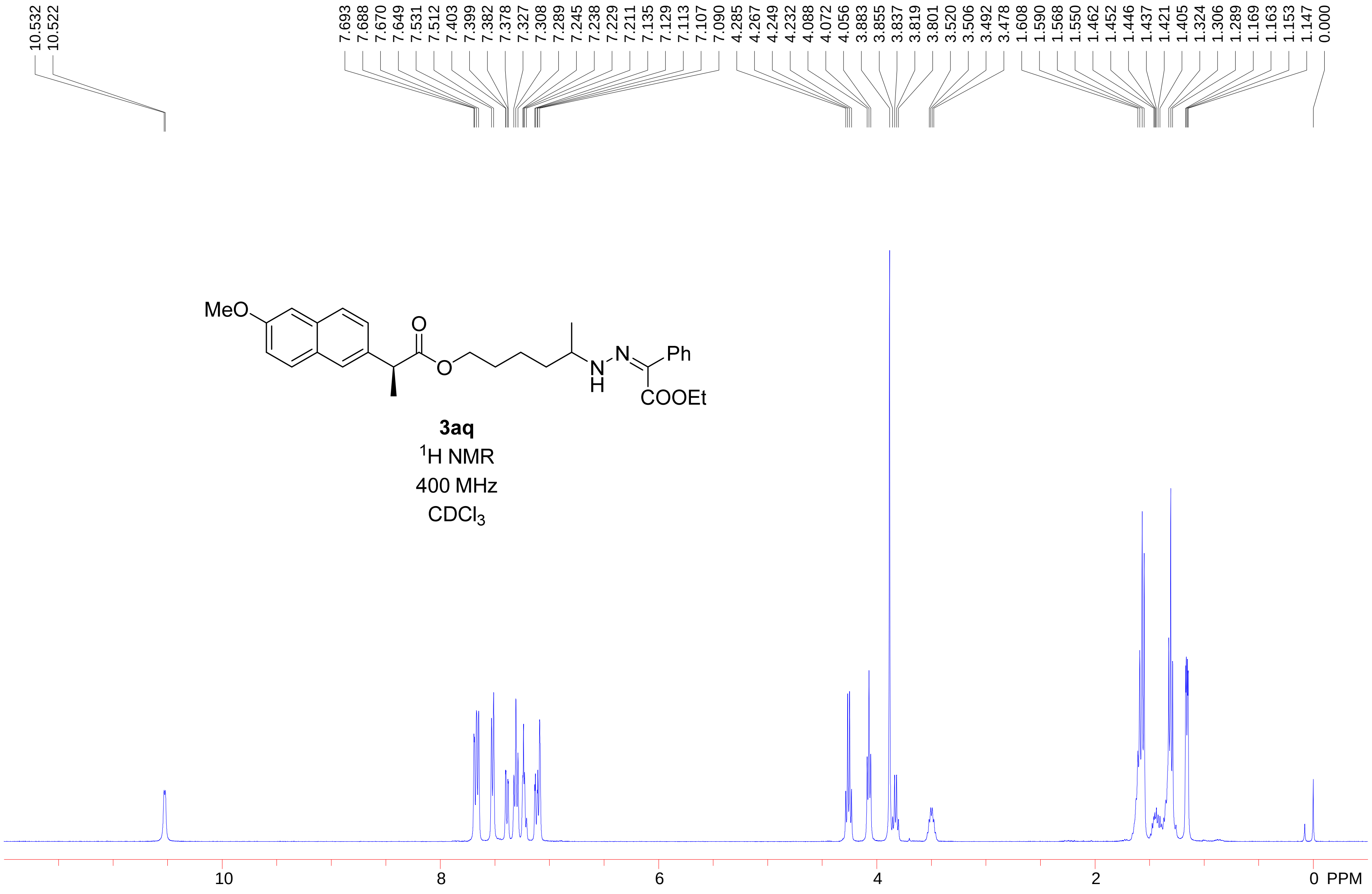

Supplementary Figure 97. ^{1}H NMR spectrum for **3aq**

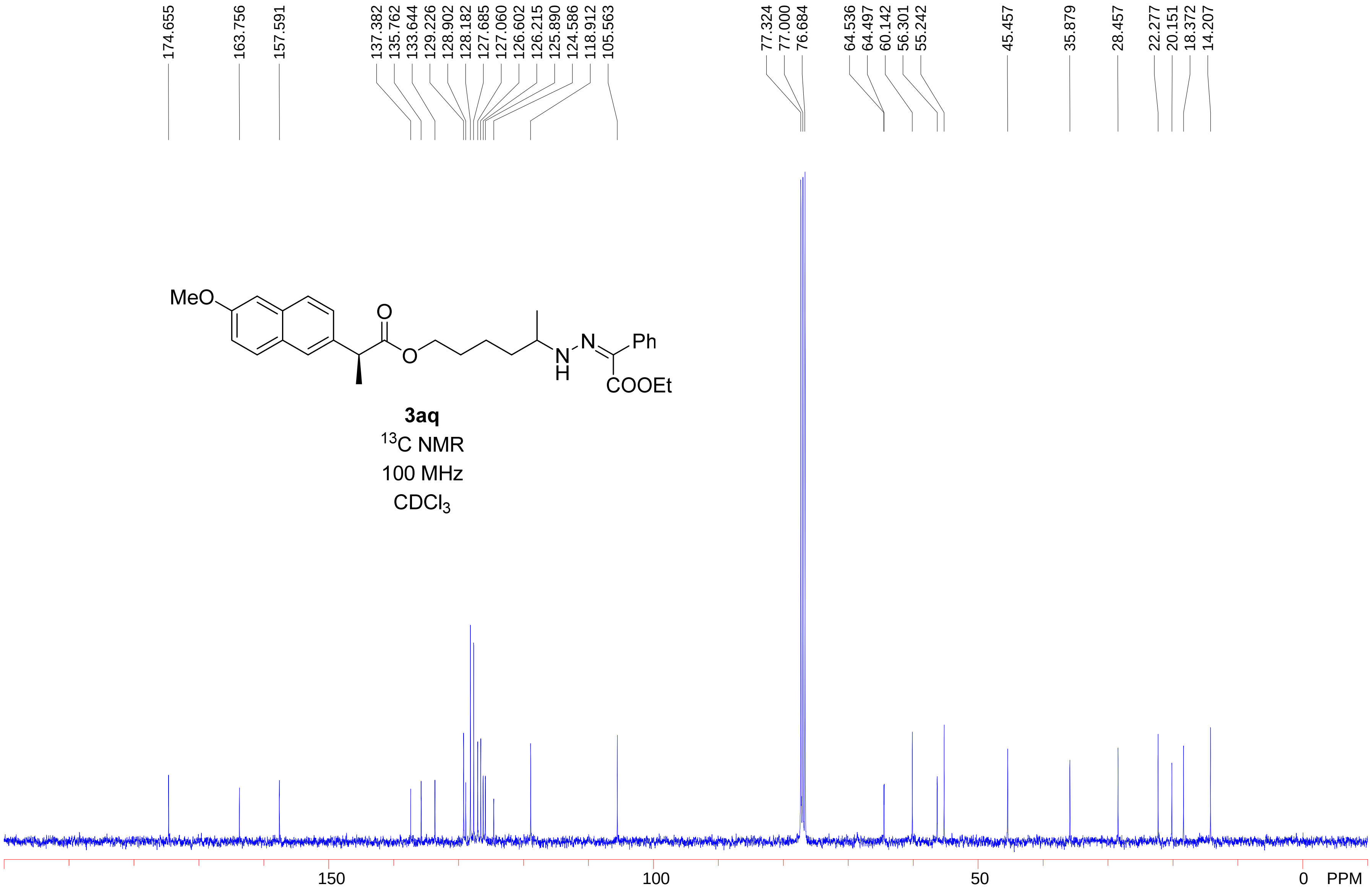

Supplementary Figure 98. ^{13}C NMR spectrum for **3aq**

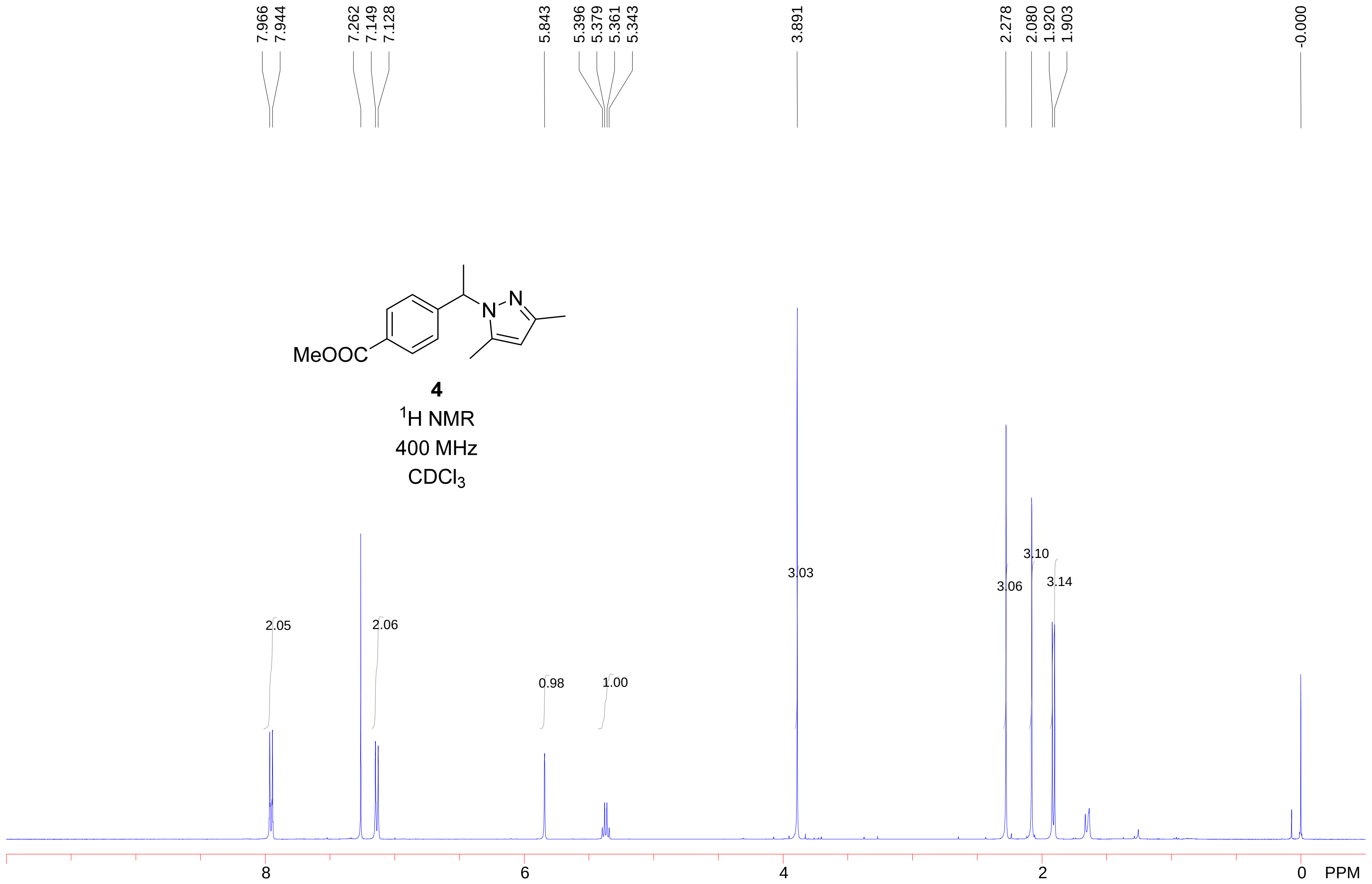

Supplementary Figure 99. ^{1}H NMR spectrum for **4**

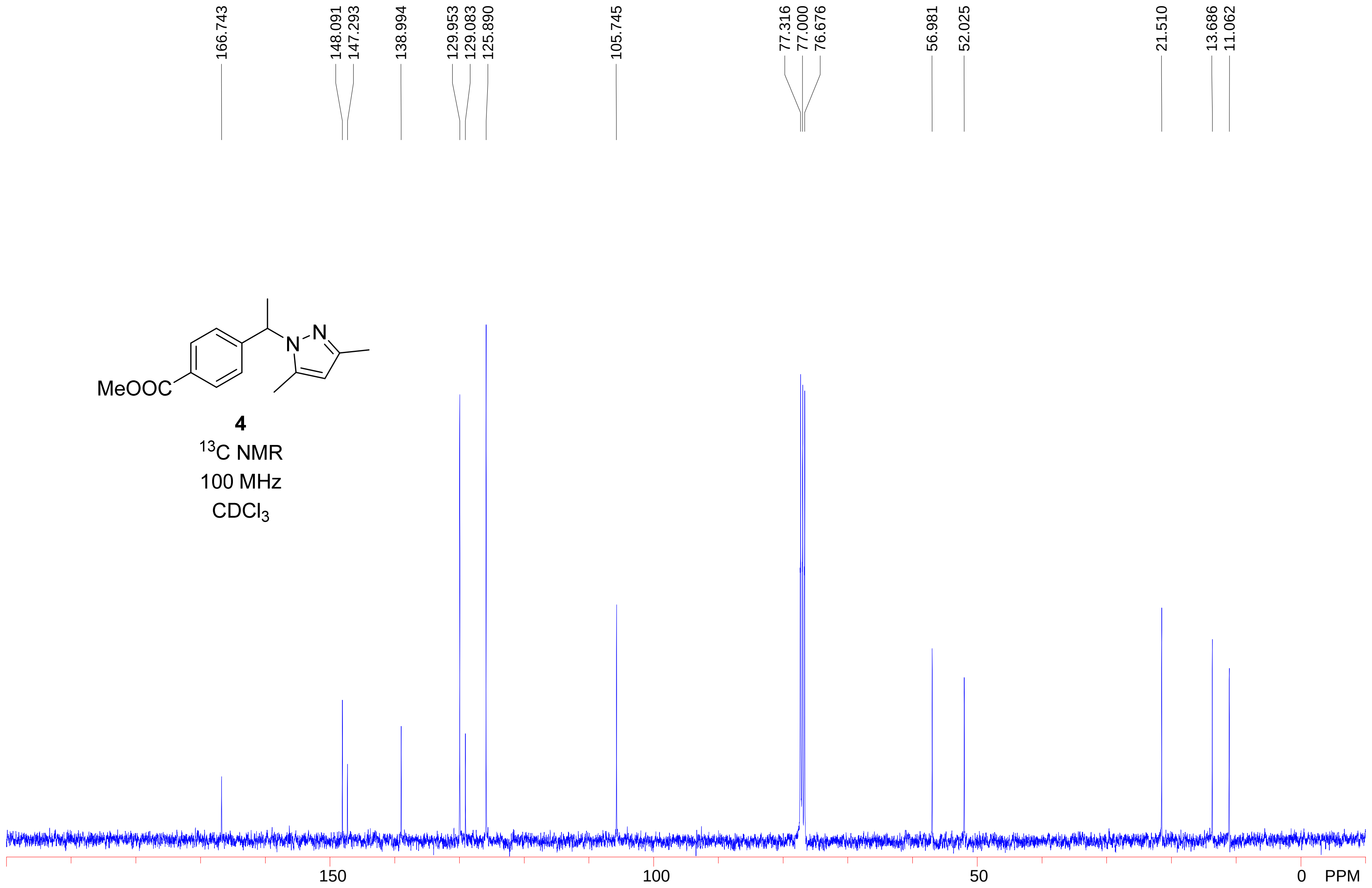

Supplementary Figure 100. ^{13}C NMR spectrum for **4**

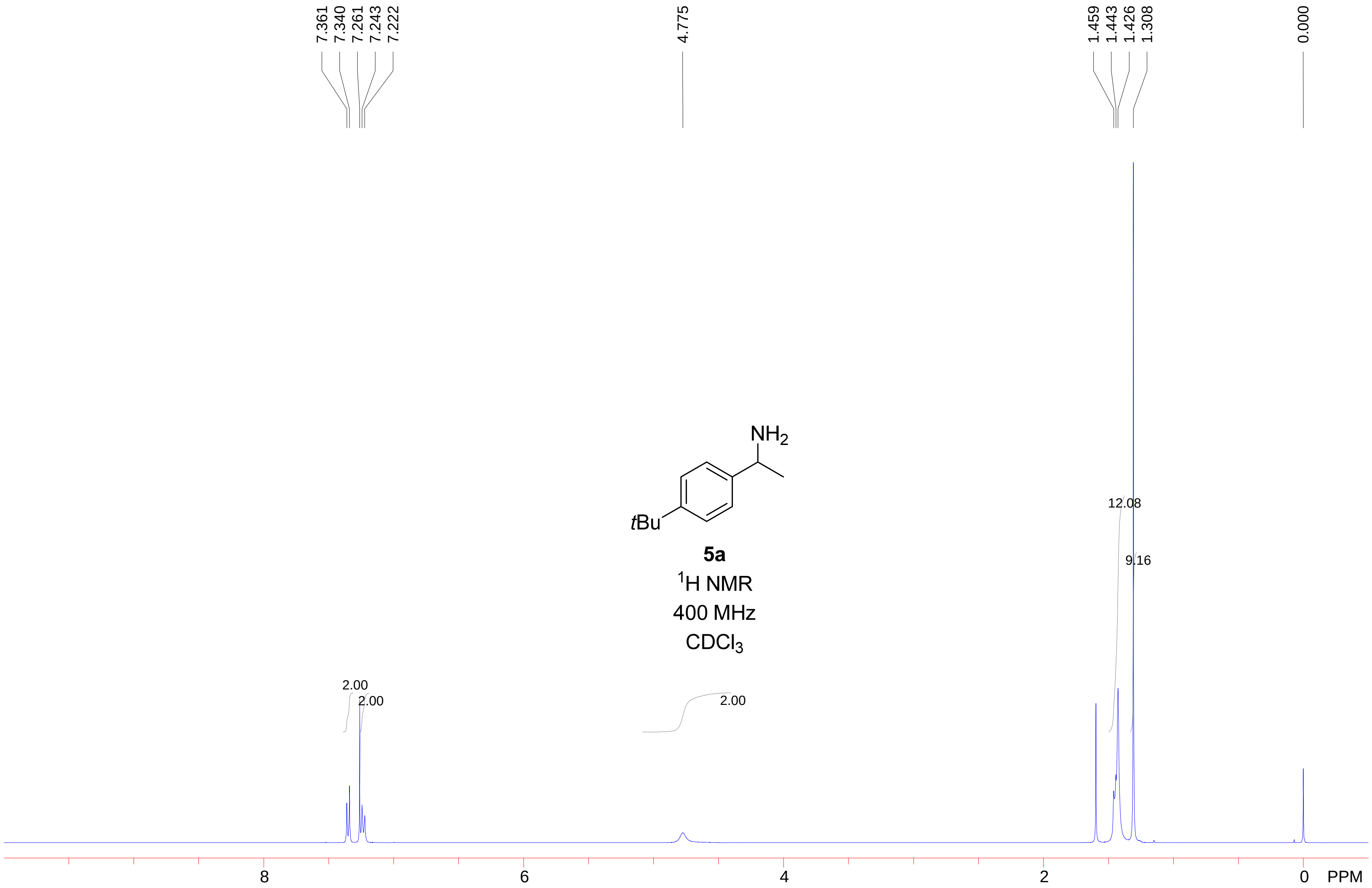

Supplementary Figure 101. ^{1}H NMR spectrum for **5a**

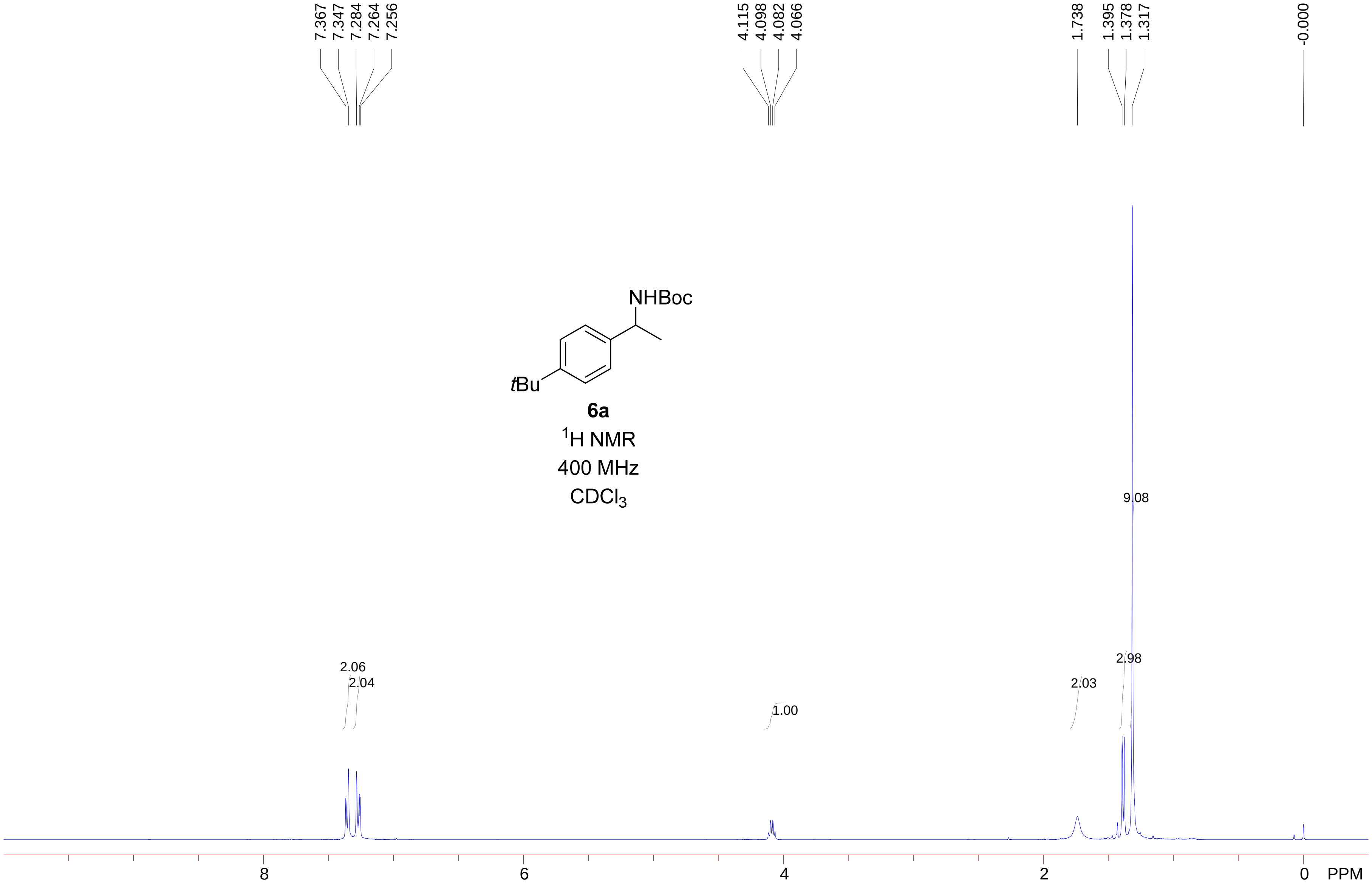

Supplementary Figure 102. ^{1}H NMR spectrum for **6a**

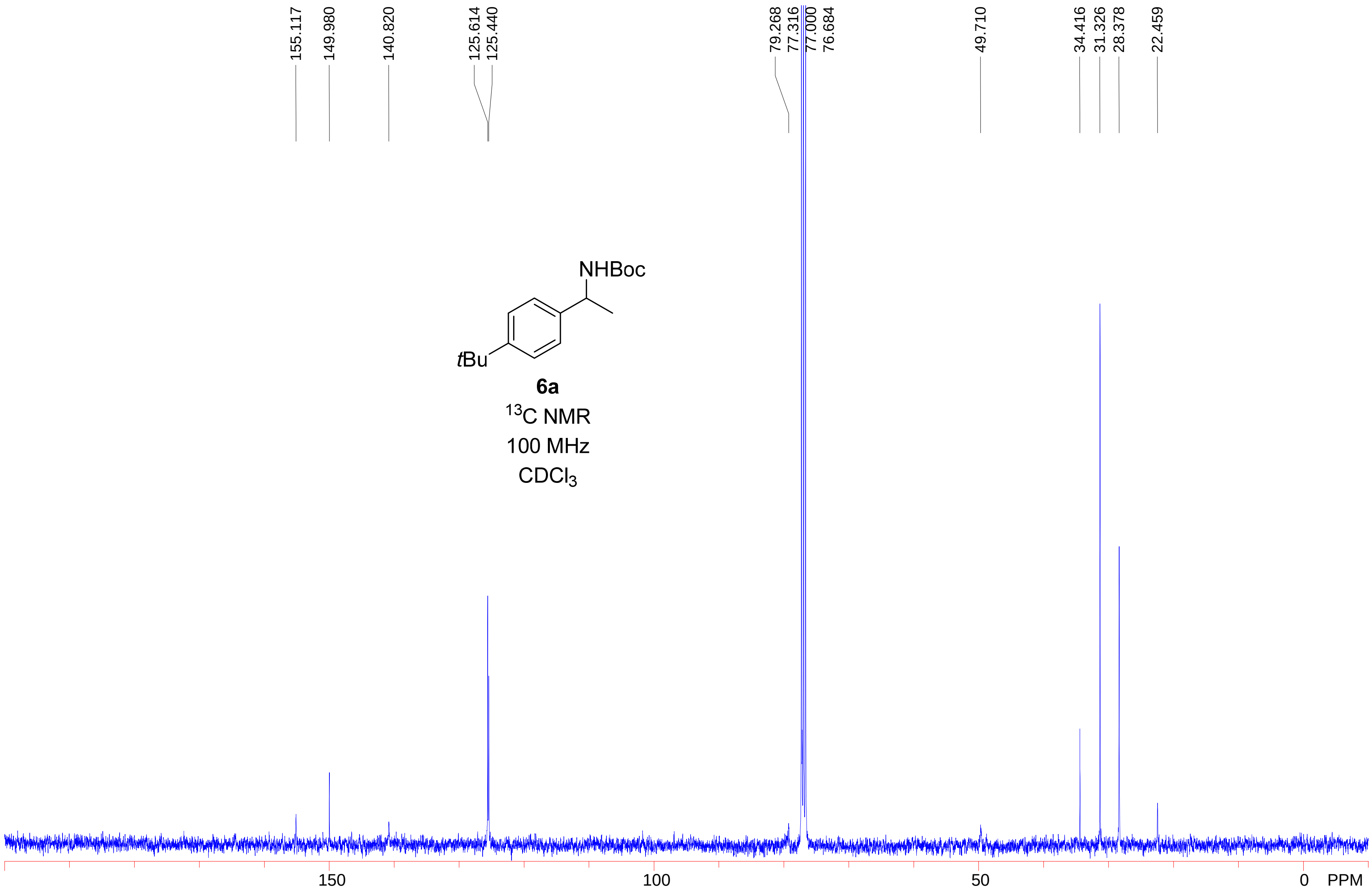

Supplementary Figure 103. ^{13}C NMR spectrum for **6a**

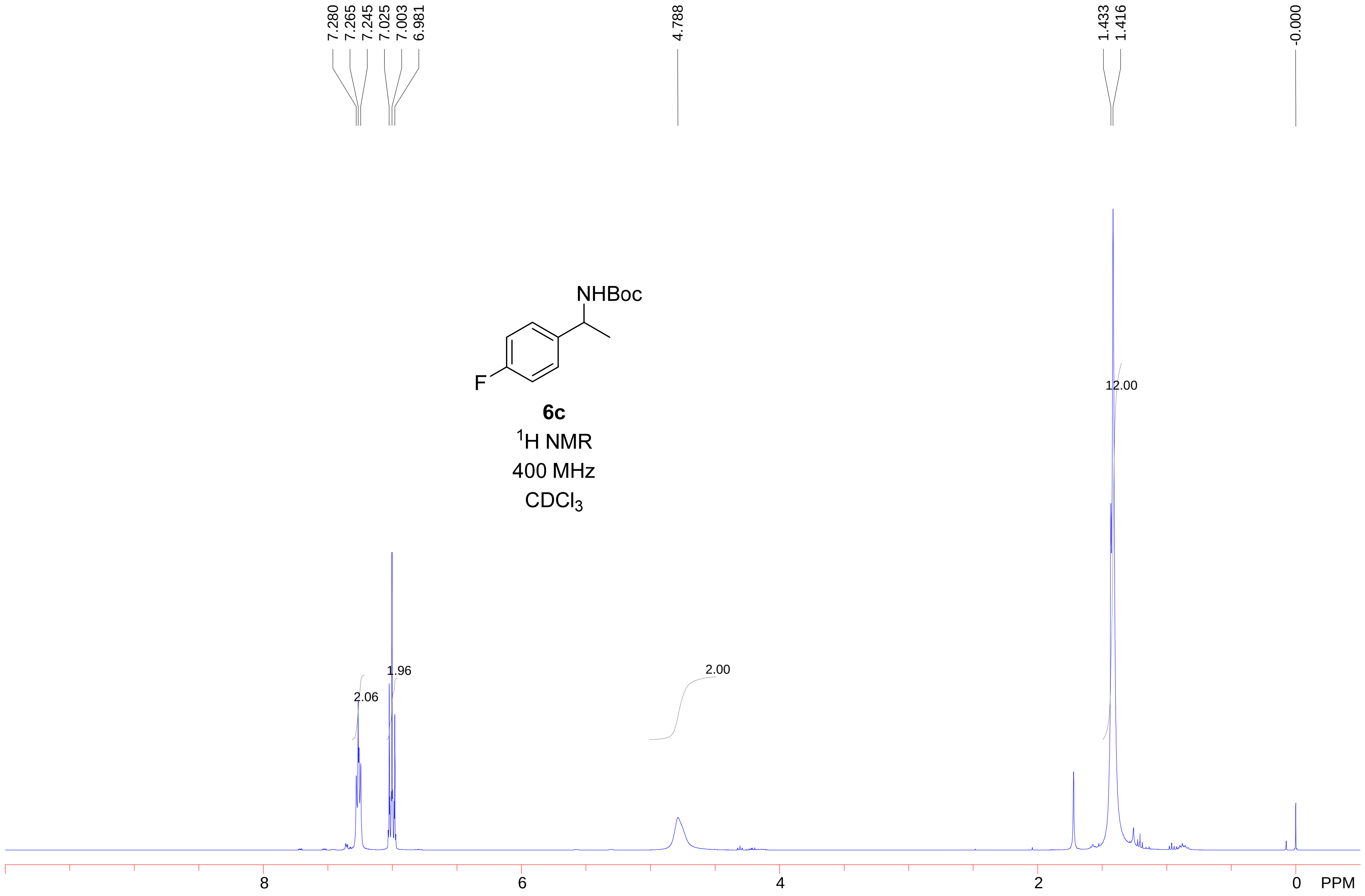

Supplementary Figure 104. ^{1}H NMR spectrum for **6c**

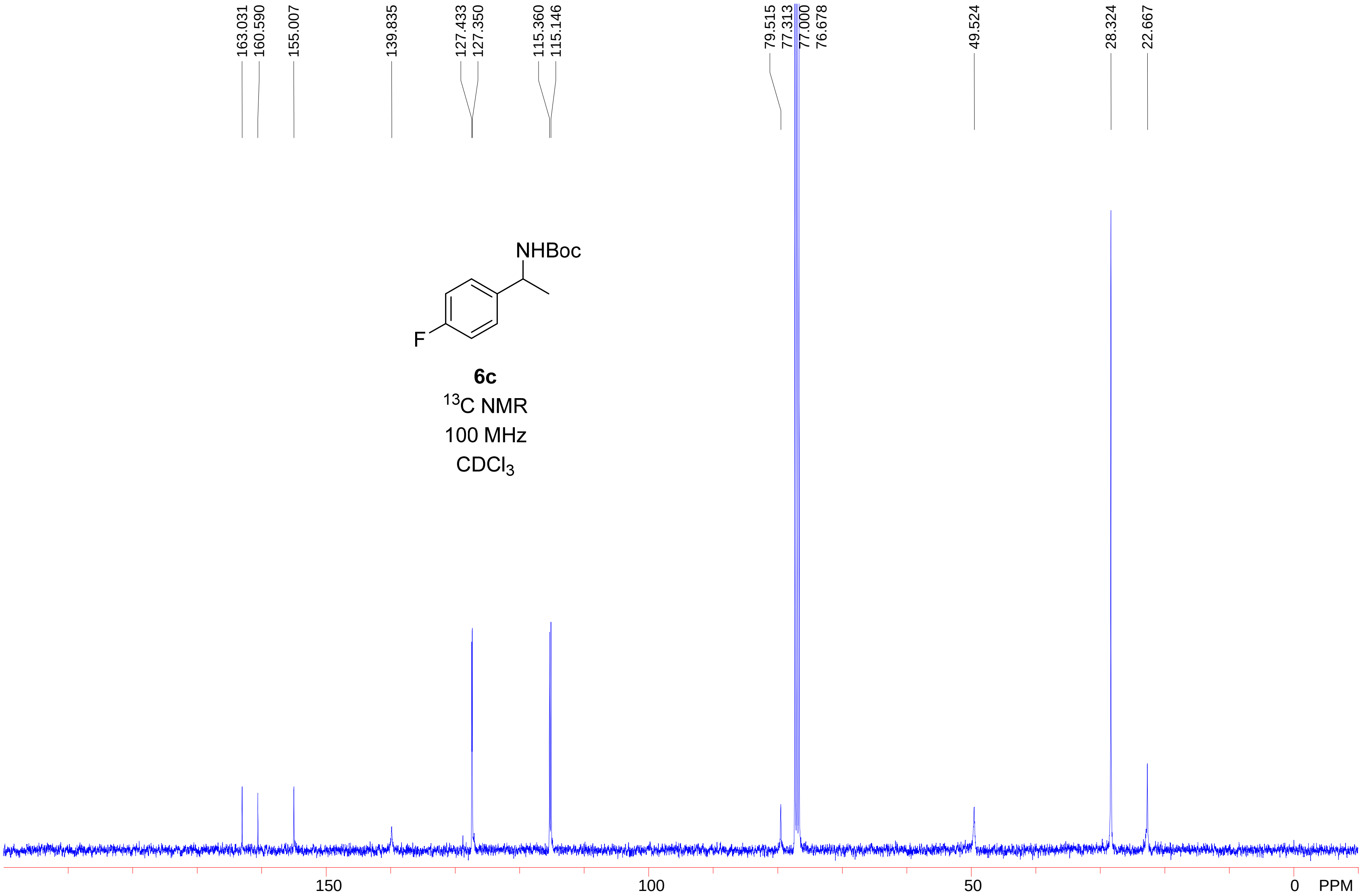

Supplementary Figure 105. ^{13}C NMR spectrum for **6c**

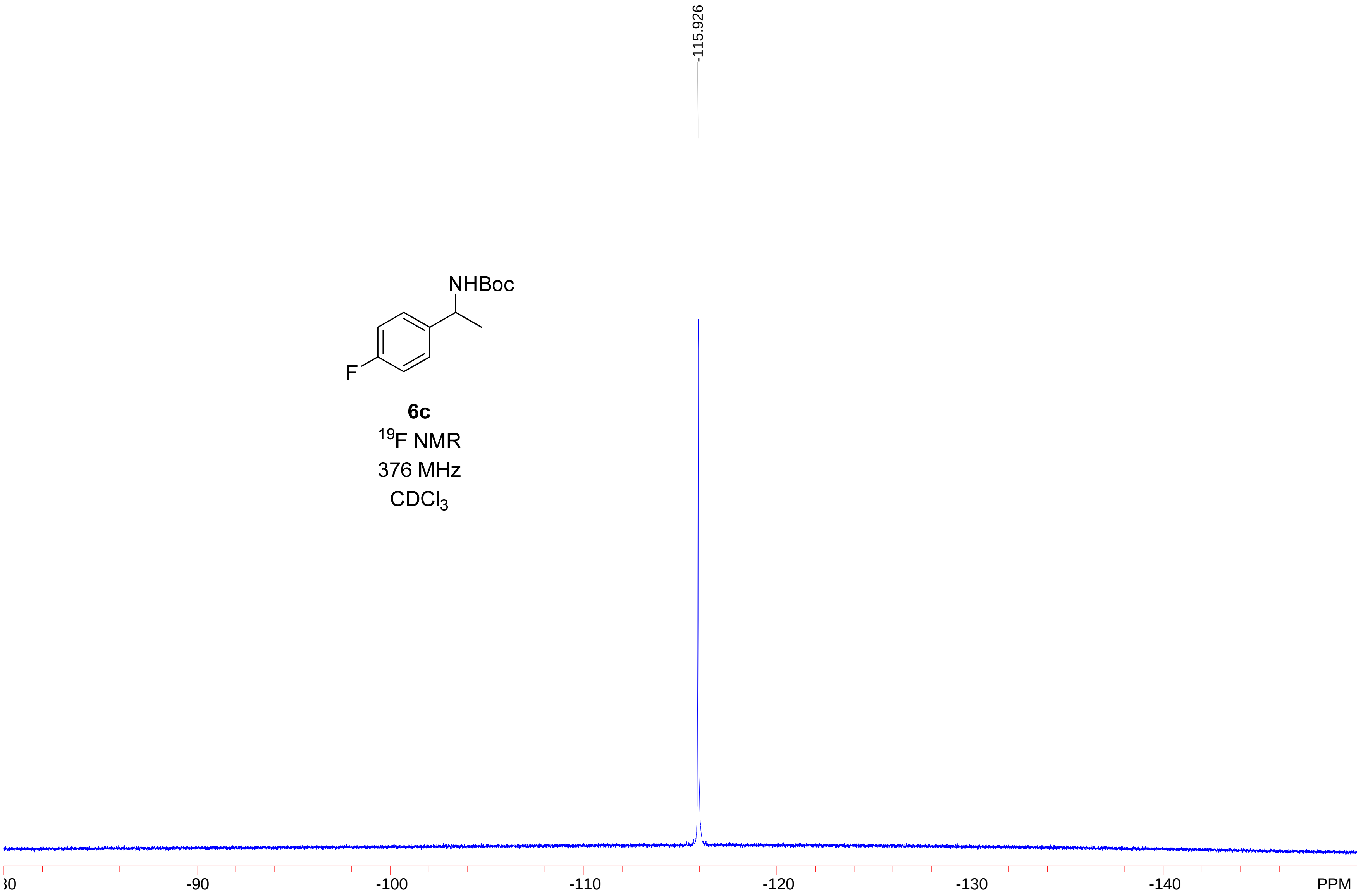

Supplementary Figure 106. ^{19}F NMR spectrum for **6c**

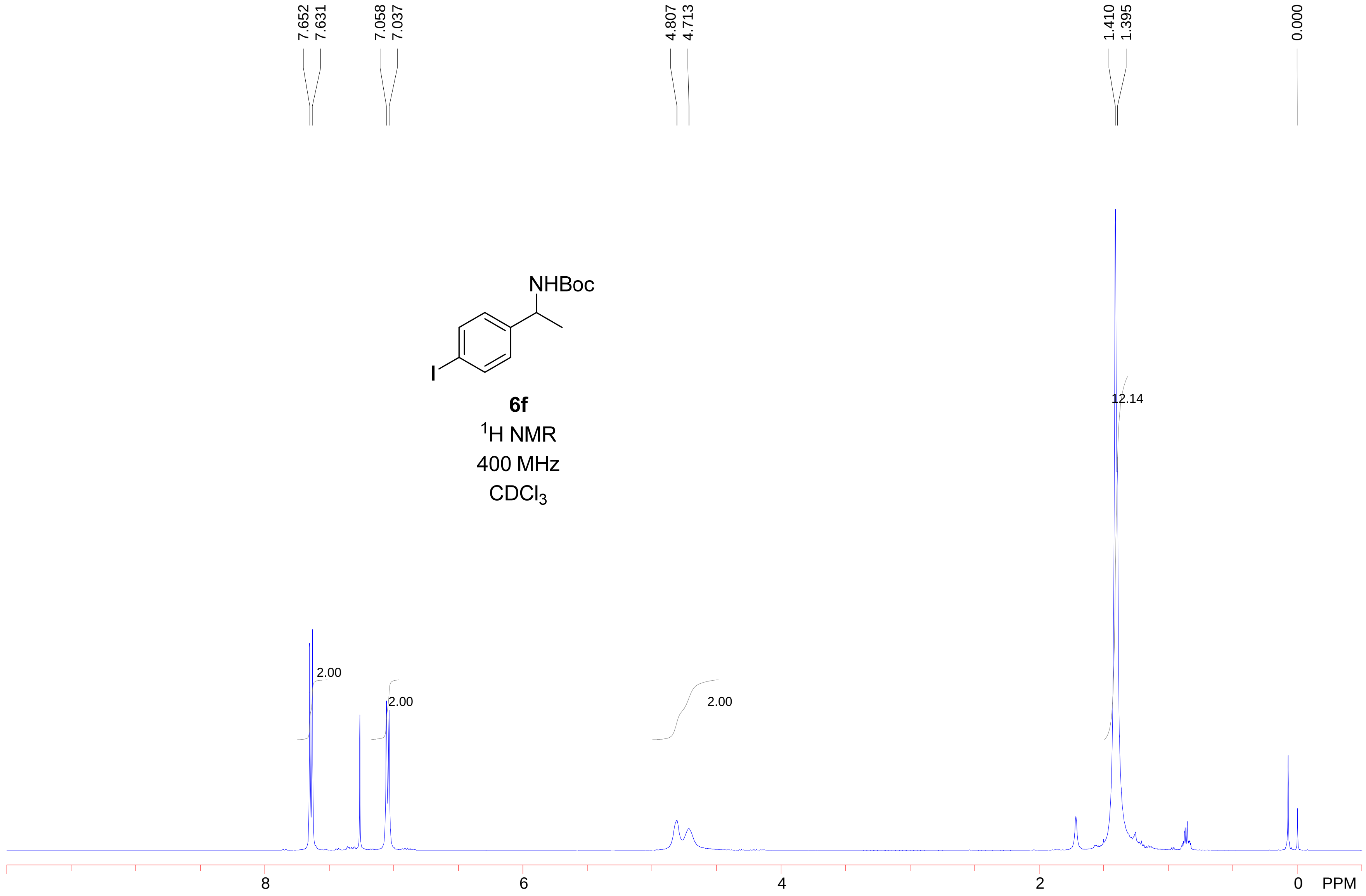

Supplementary Figure 107. ^{1}H NMR spectrum for **6f**

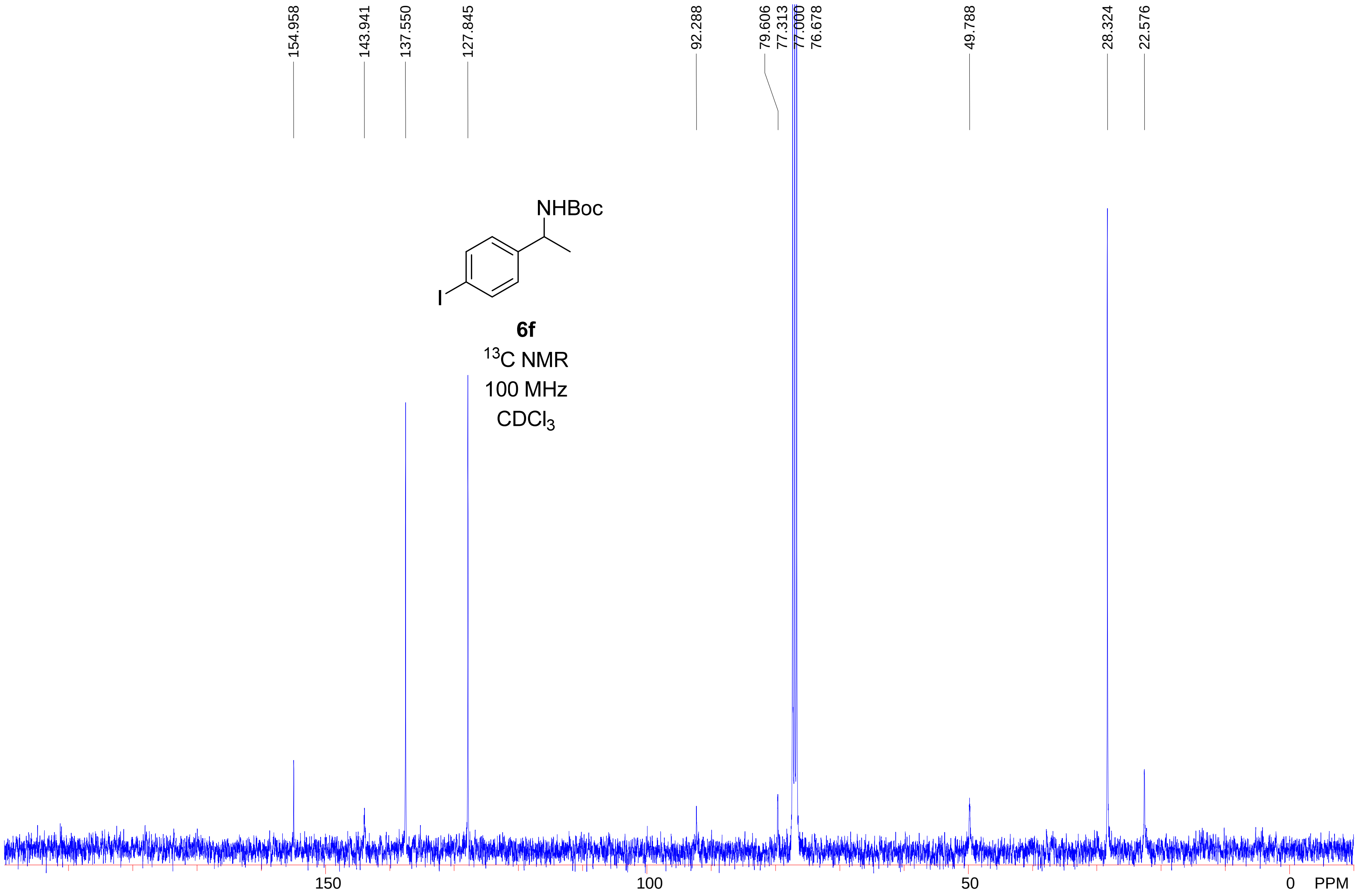

Supplementary Figure 108. ^{13}C NMR spectrum for **6f**

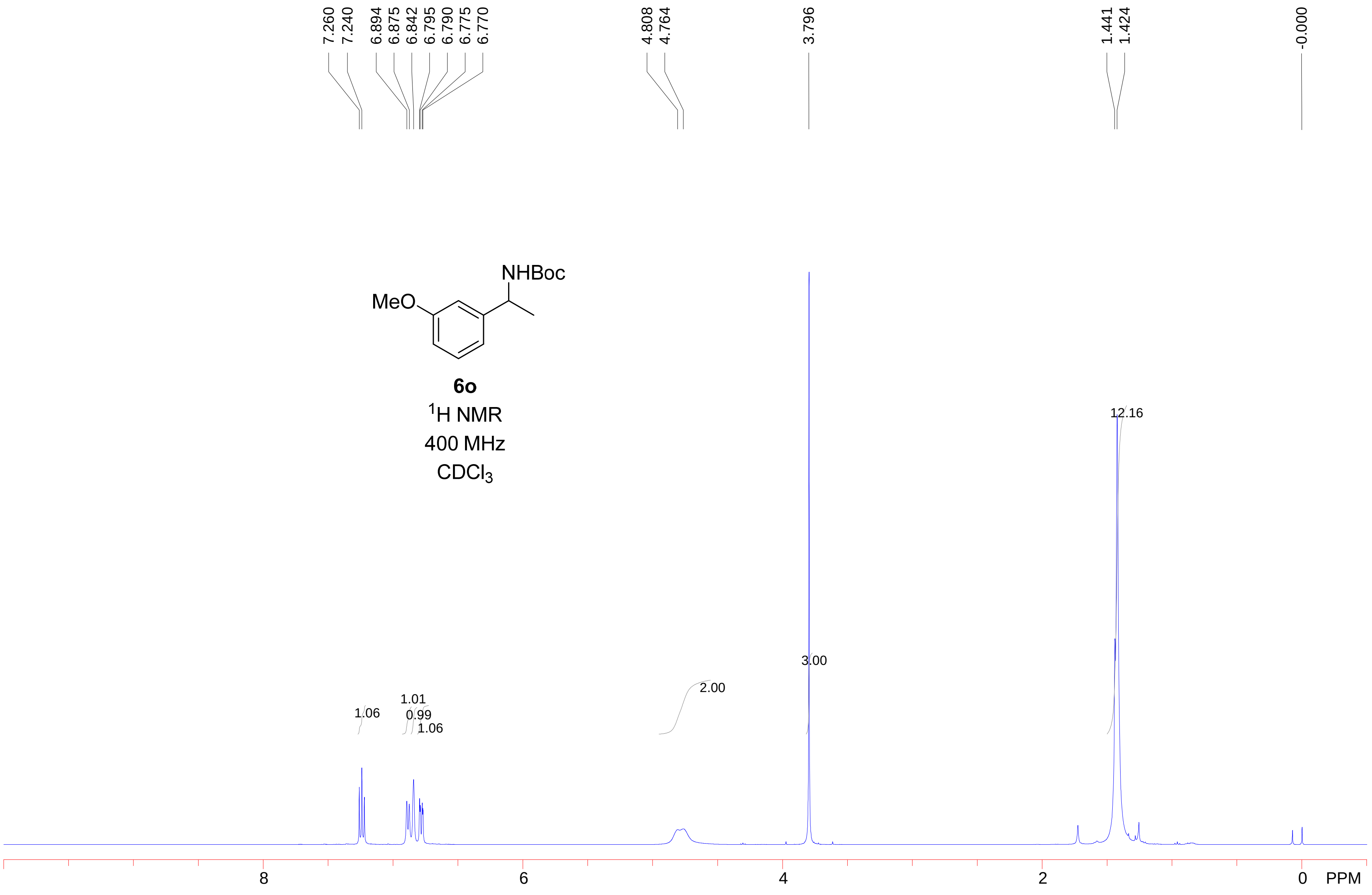

Supplementary Figure 109. ^{1}H NMR spectrum for **6o**

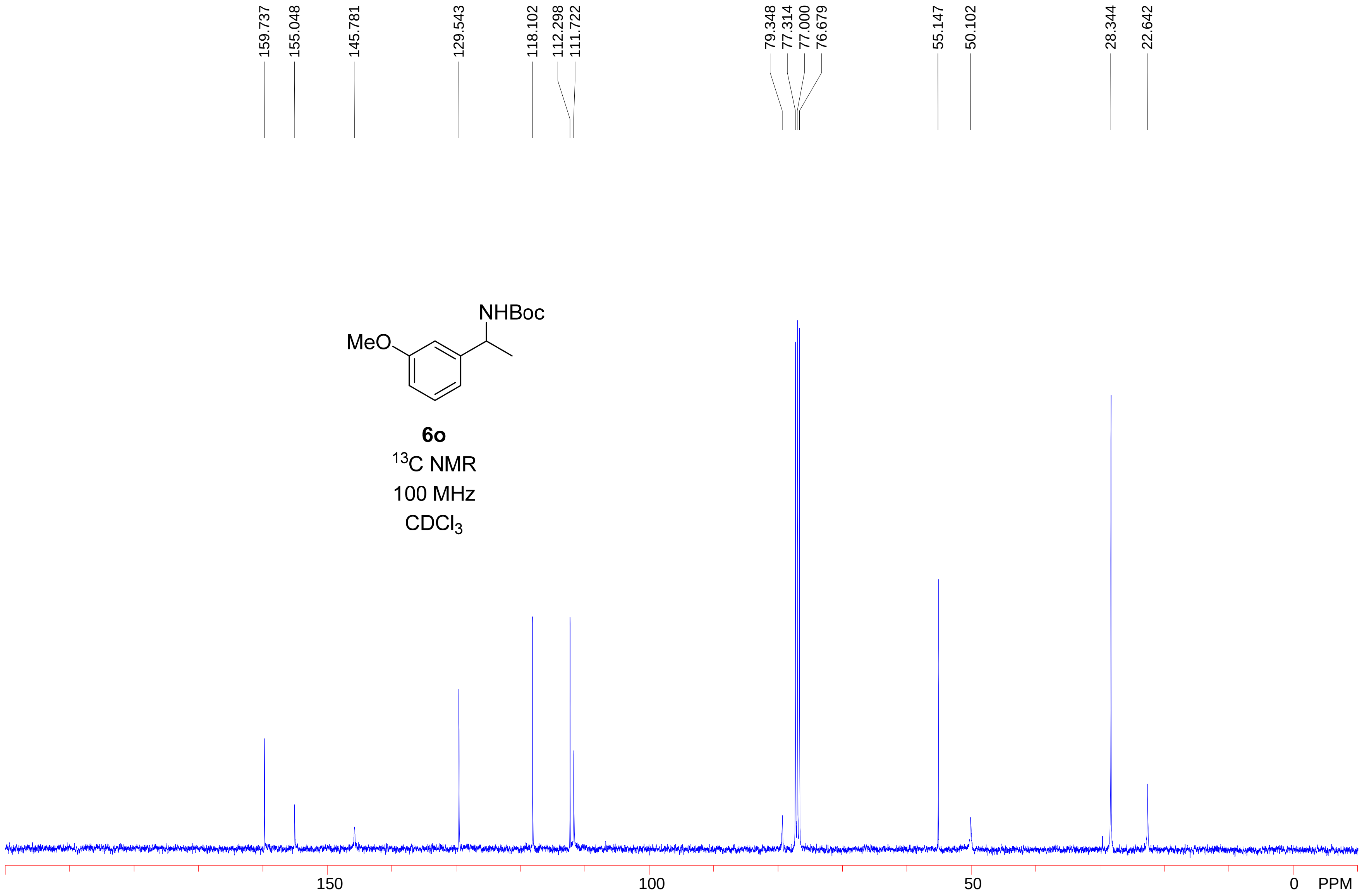

Supplementary Figure 110. ^{13}C NMR spectrum for **6o**

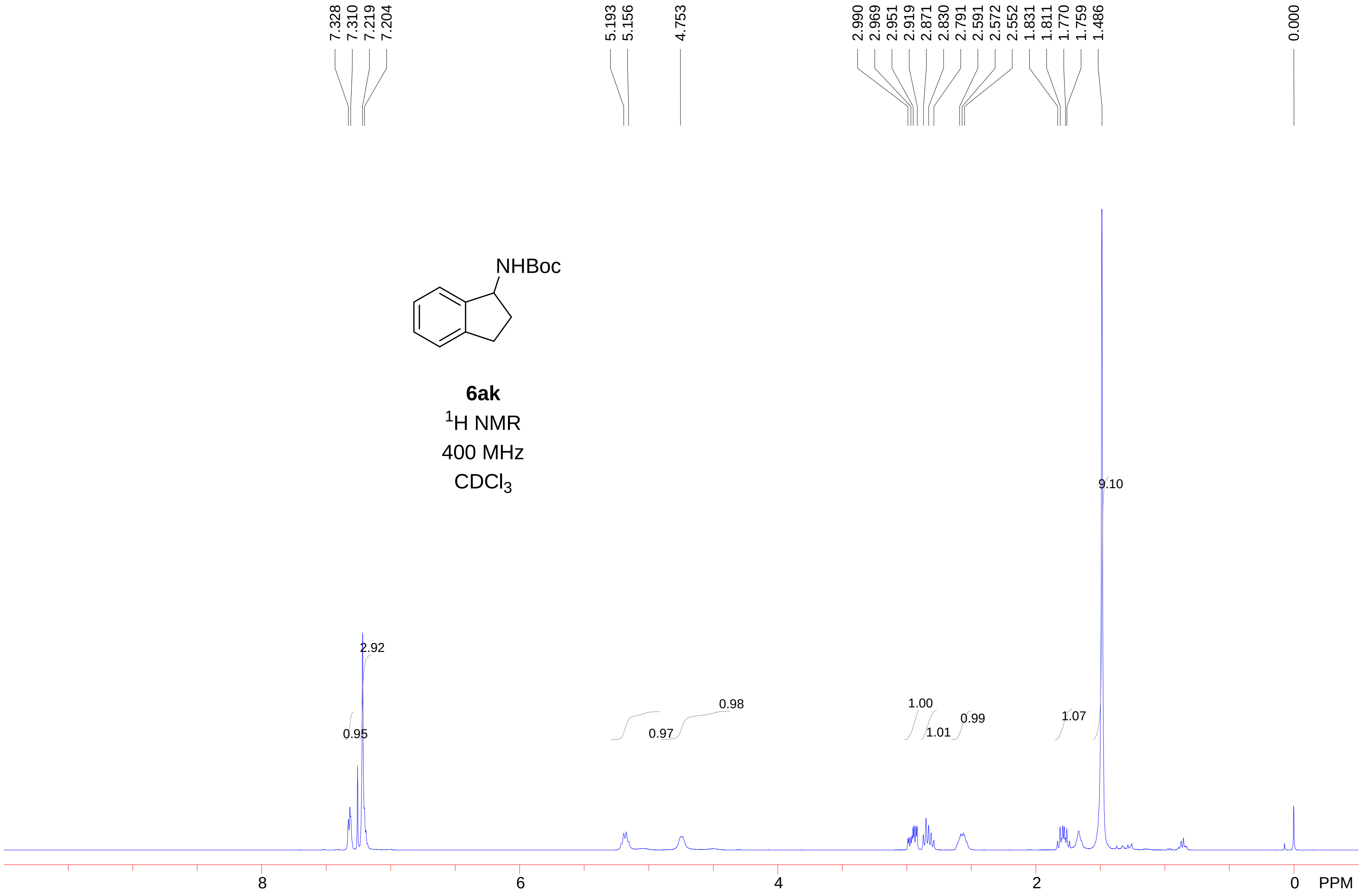

Supplementary Figure 111. ^{1}H NMR spectrum for **6ak**

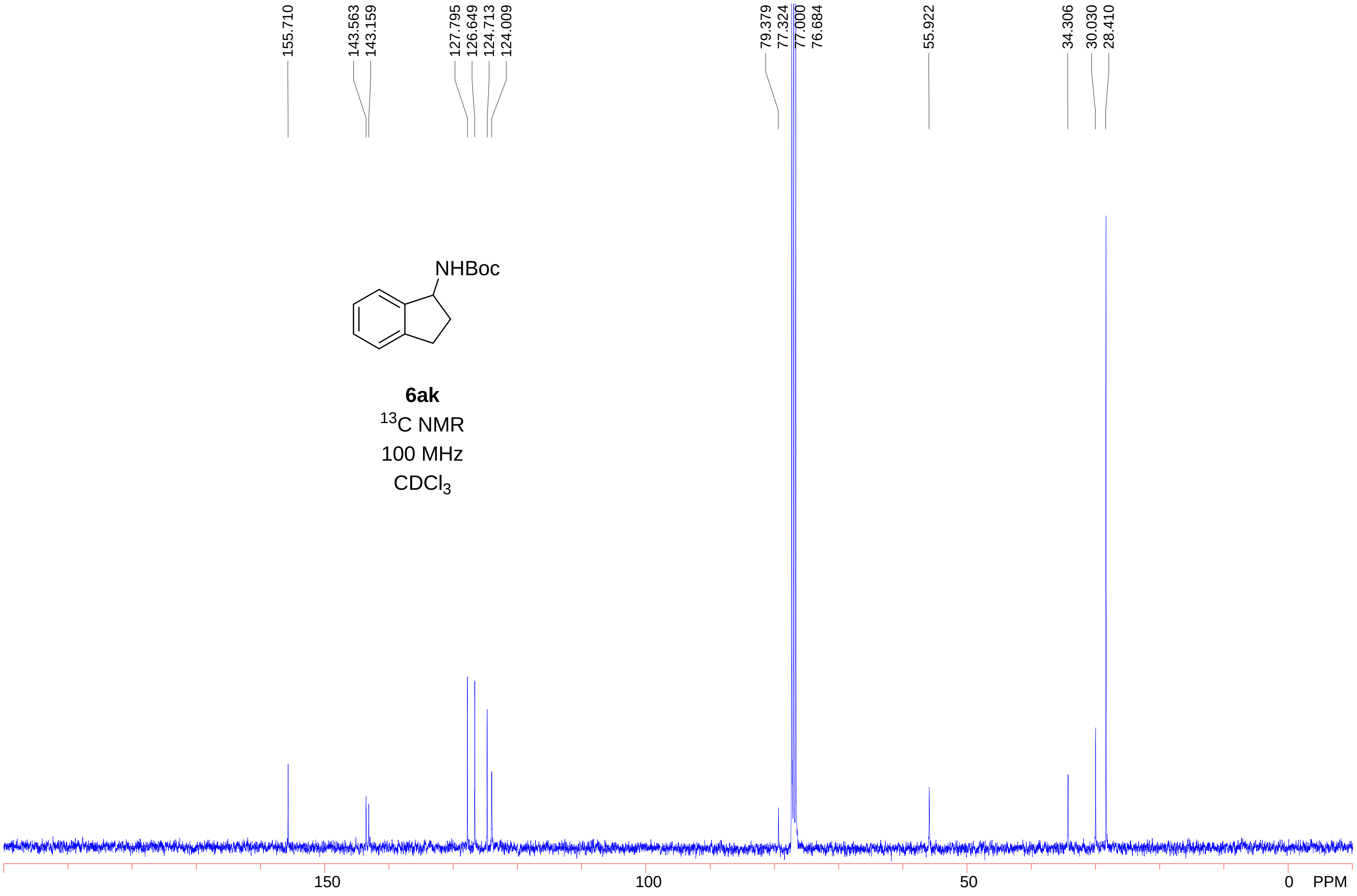

Supplementary Figure 112. ^{13}C NMR spectrum for **6ak**

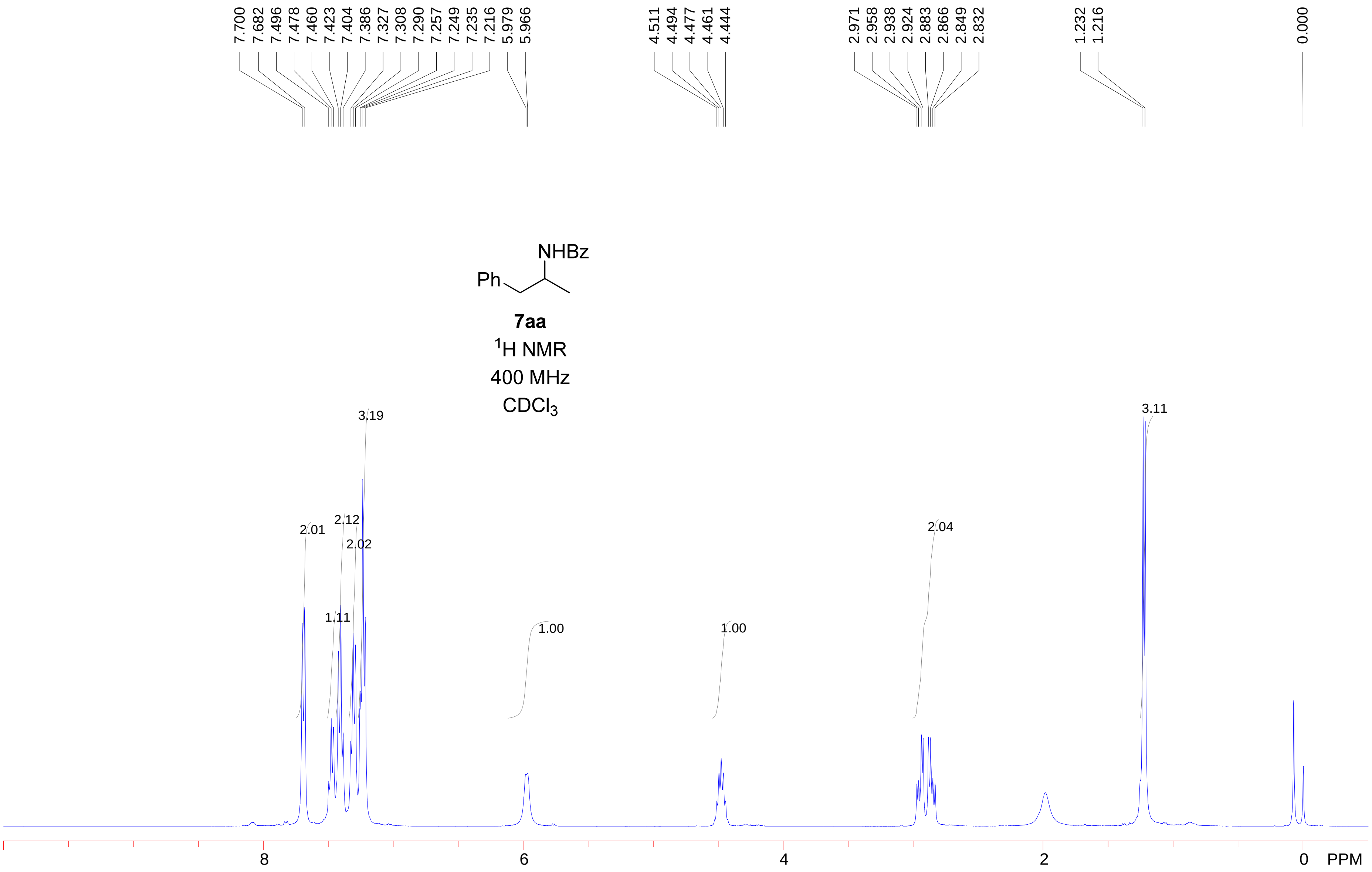

Supplementary Figure 113. ^{1}H NMR spectrum for **7aa**

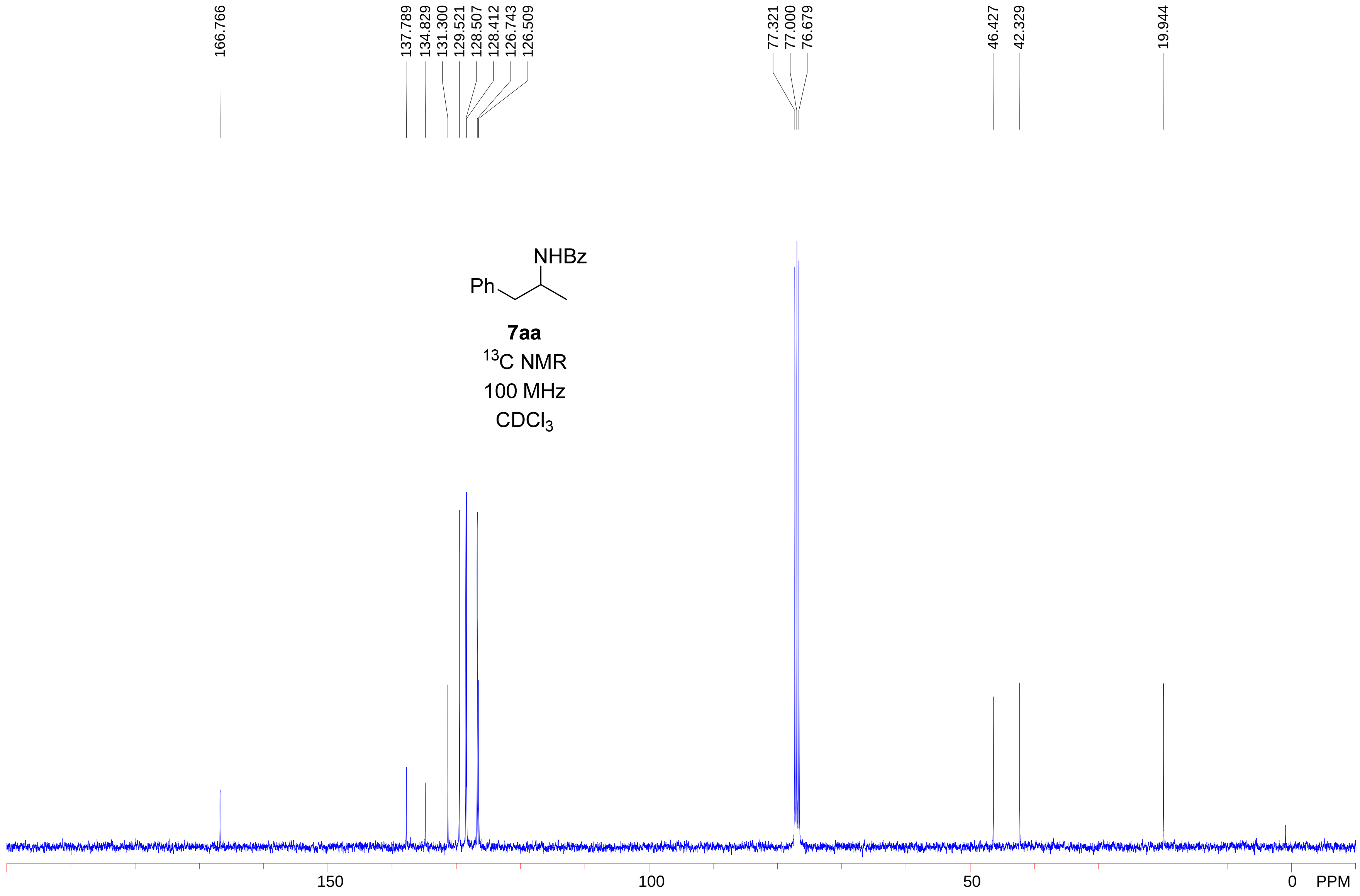

Supplementary Figure 114. ^{13}C NMR spectrum for **7aa**

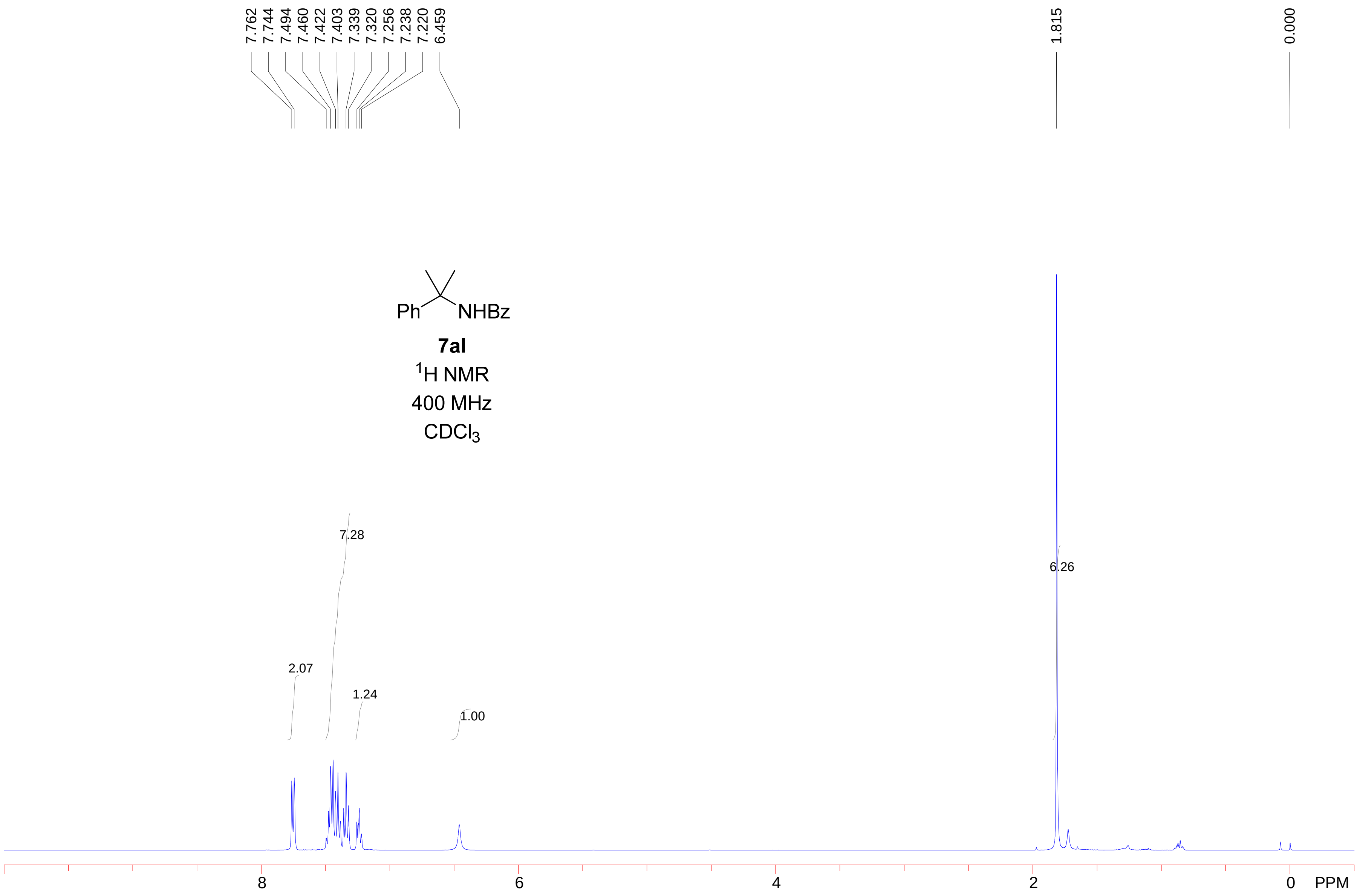

Supplementary Figure 115. ^{1}H NMR spectrum for **7al**

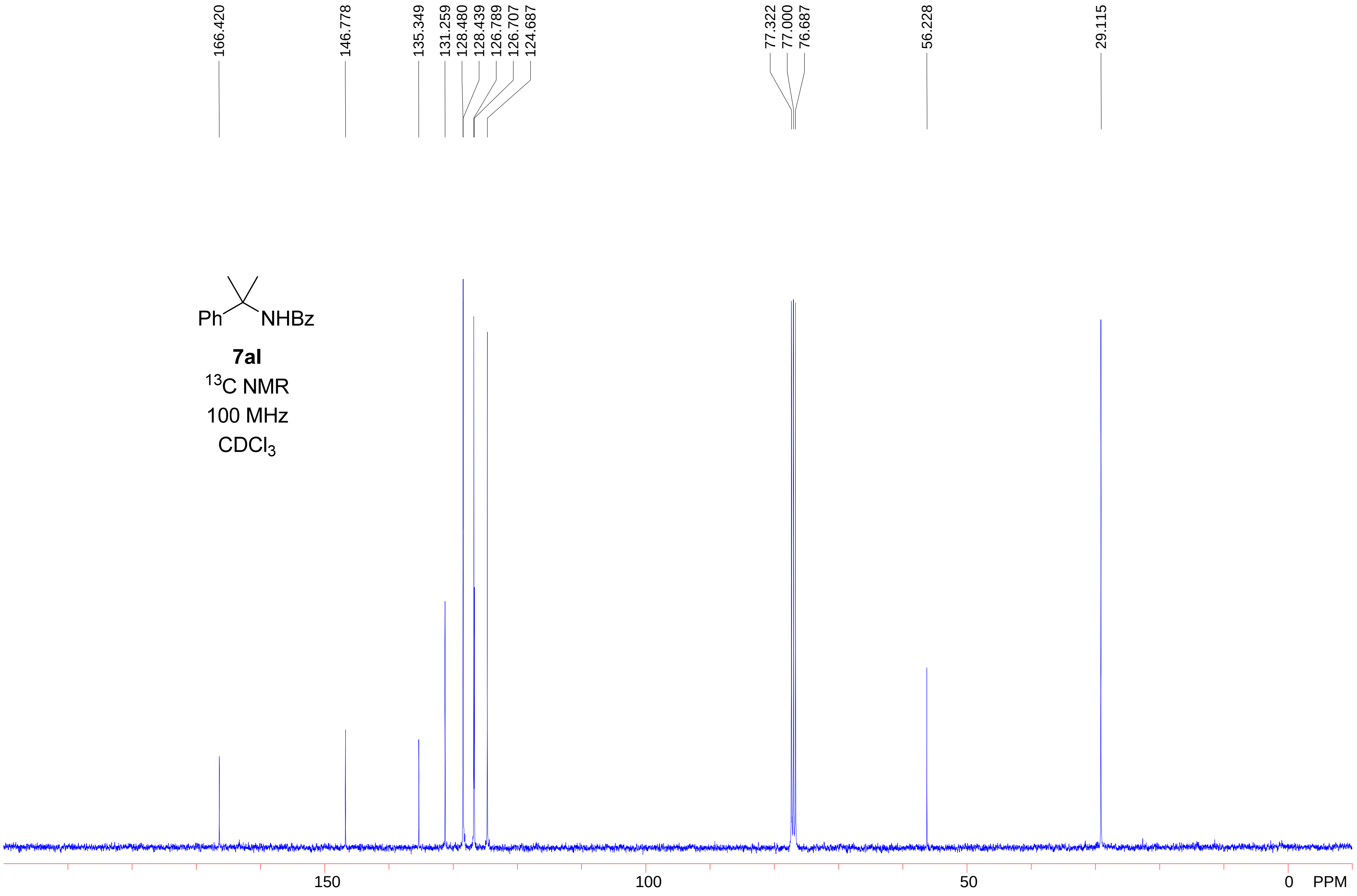

Supplementary Figure 116. ^{13}C NMR spectrum for **7al**

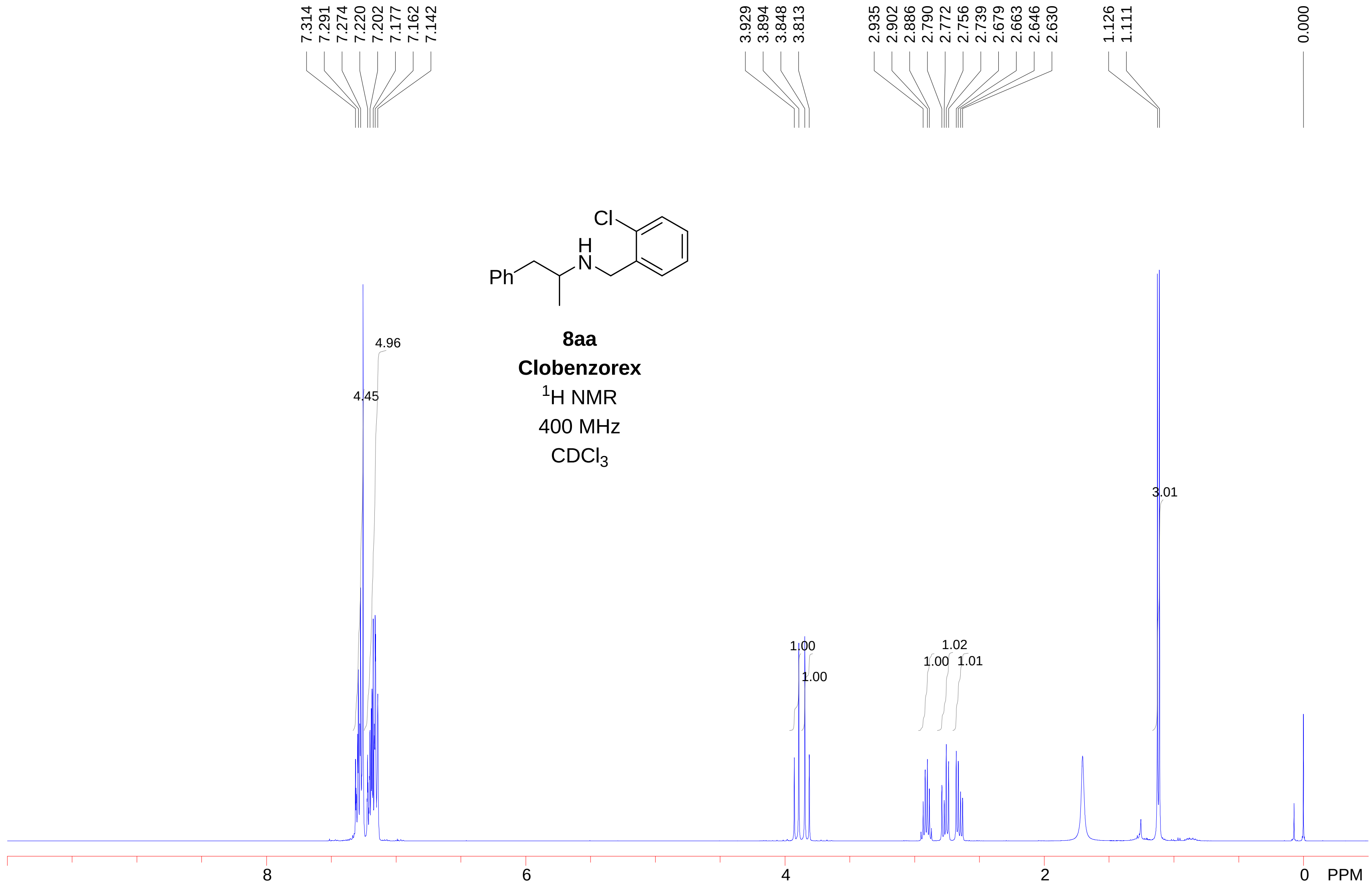

Supplementary Figure 117. ^{1}H NMR spectrum for **8aa**

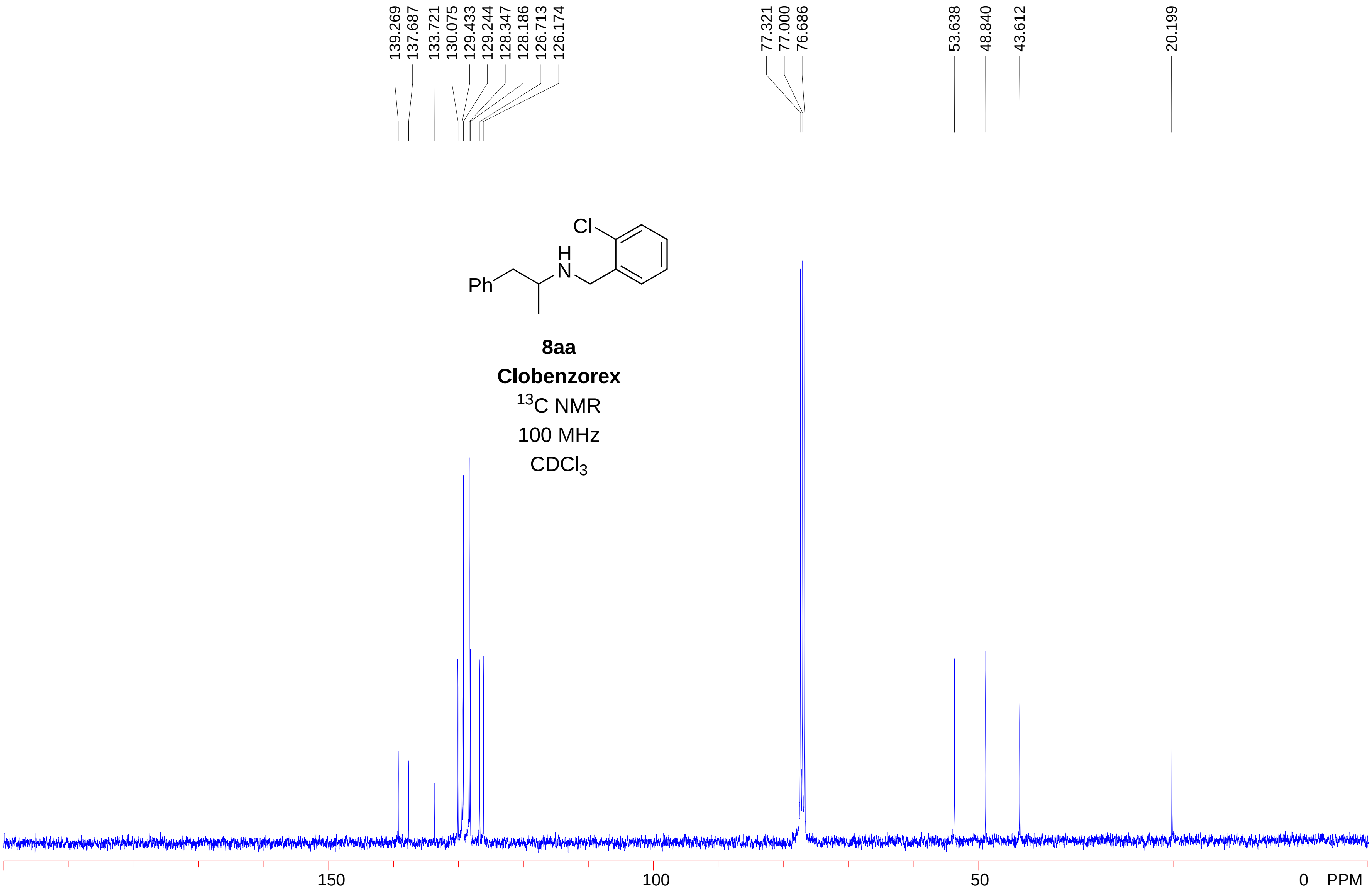

Supplementary Figure 118. ^{13}C NMR spectrum for **8aa**

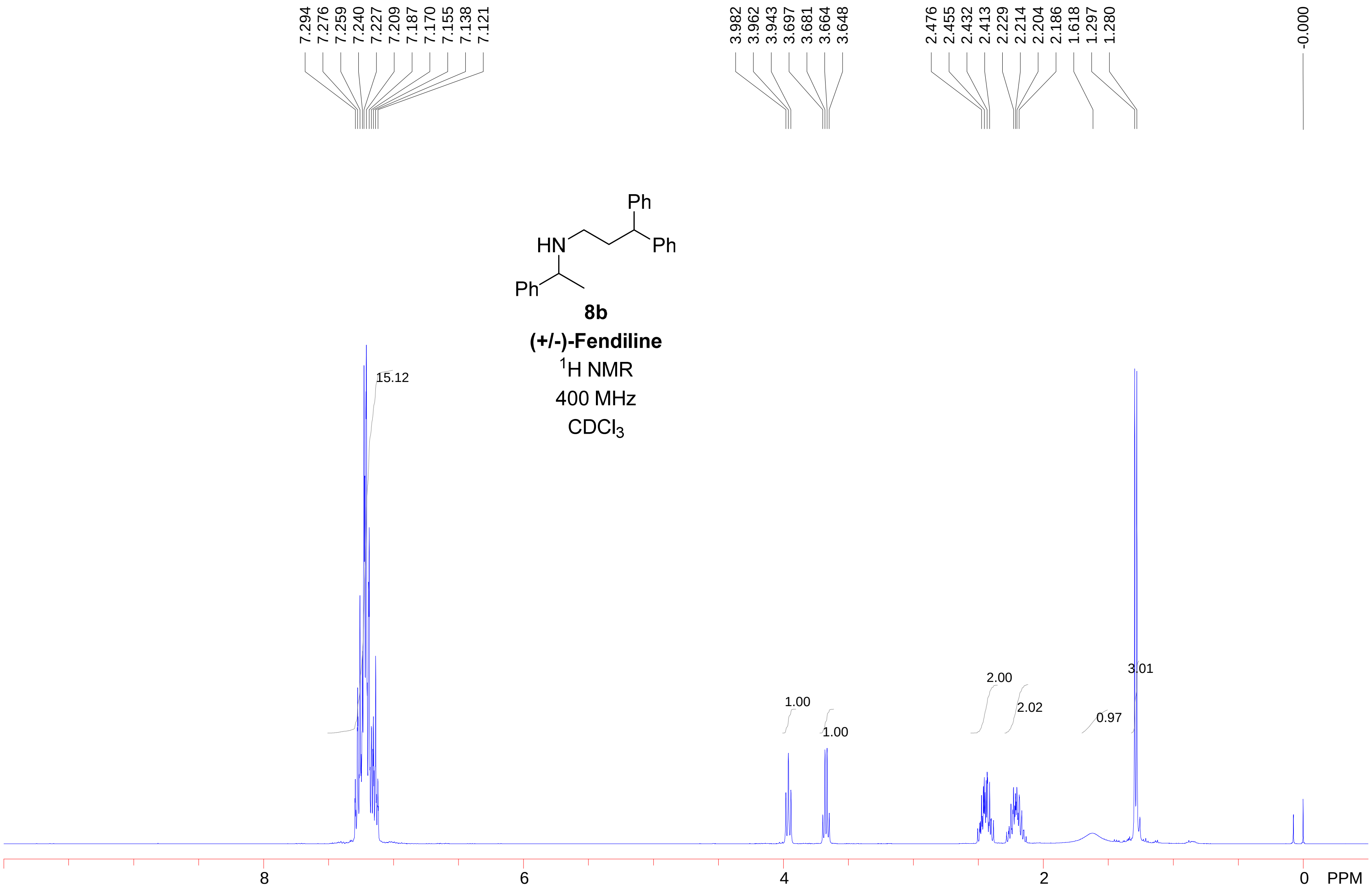

Supplementary Figure 119. ^{1}H NMR spectrum for **8b**

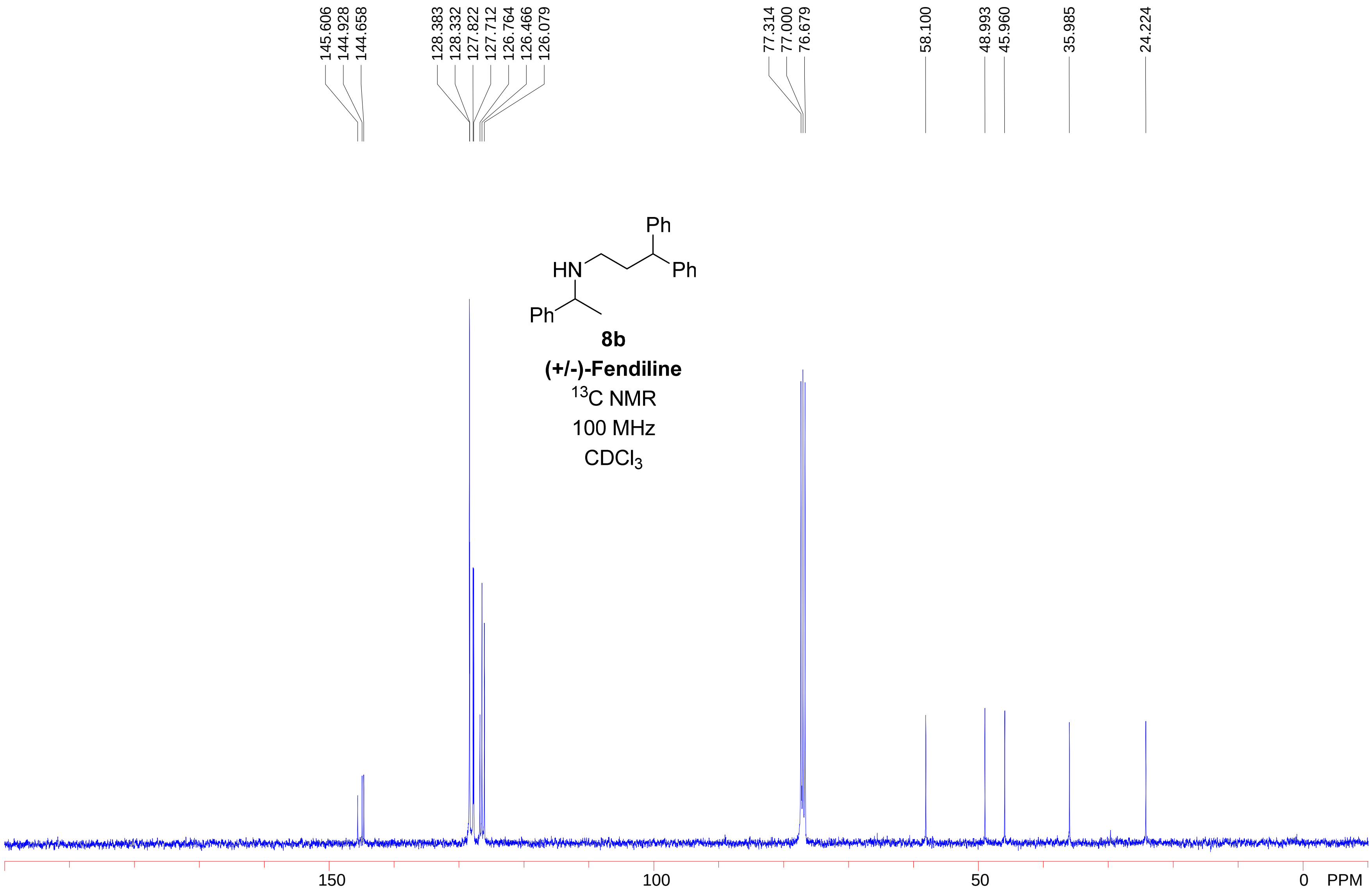

Supplementary Figure 120. ^{13}C NMR spectrum for **8b**

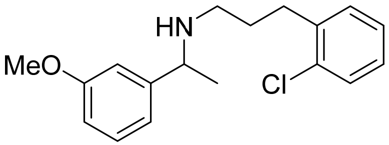

8o
(+/-)-NPS R568
^{1}H NMR
400 MHz
$CDCl_3$

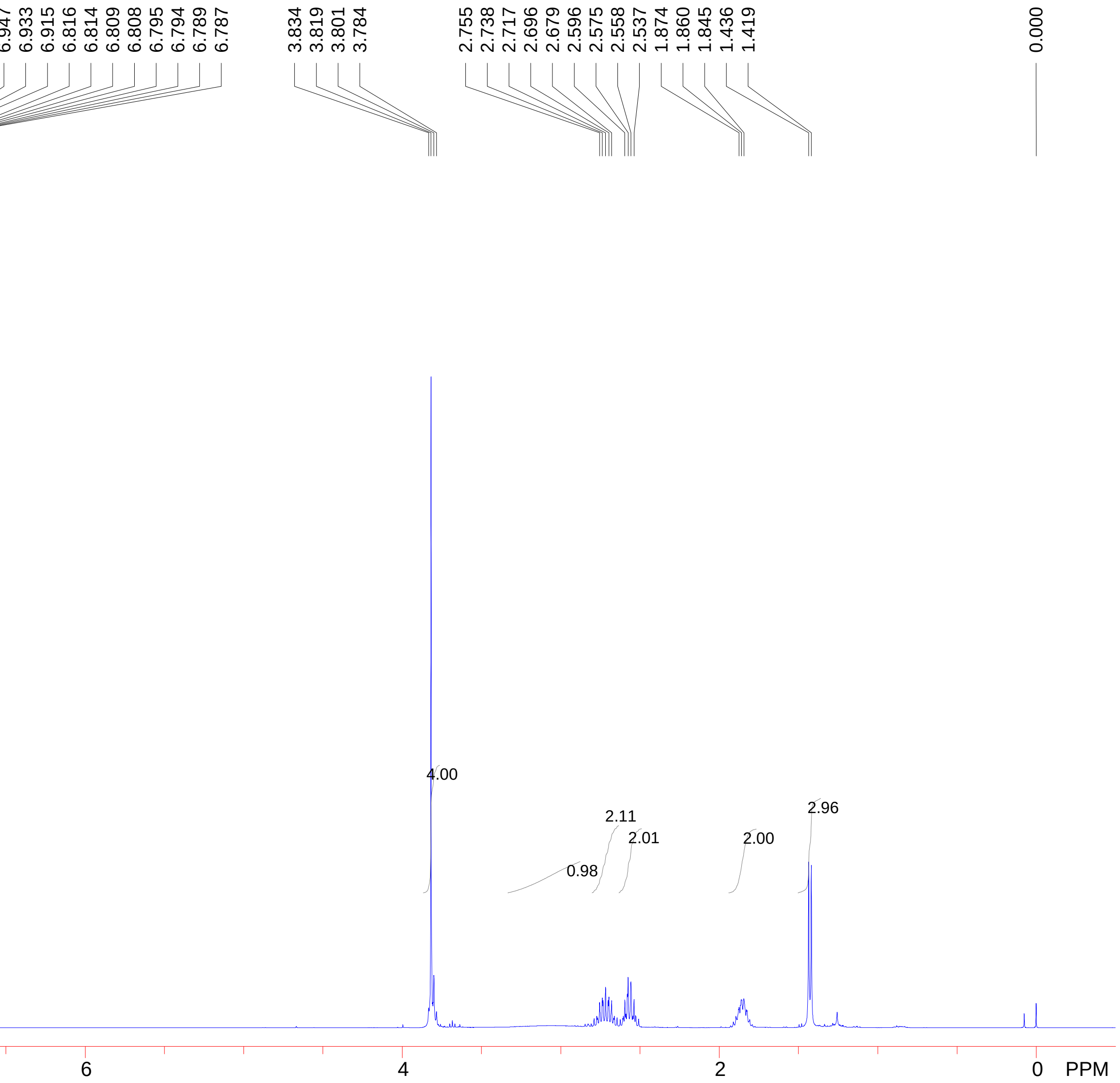

Supplementary Figure 121. ^{1}H NMR spectrum for **8o**

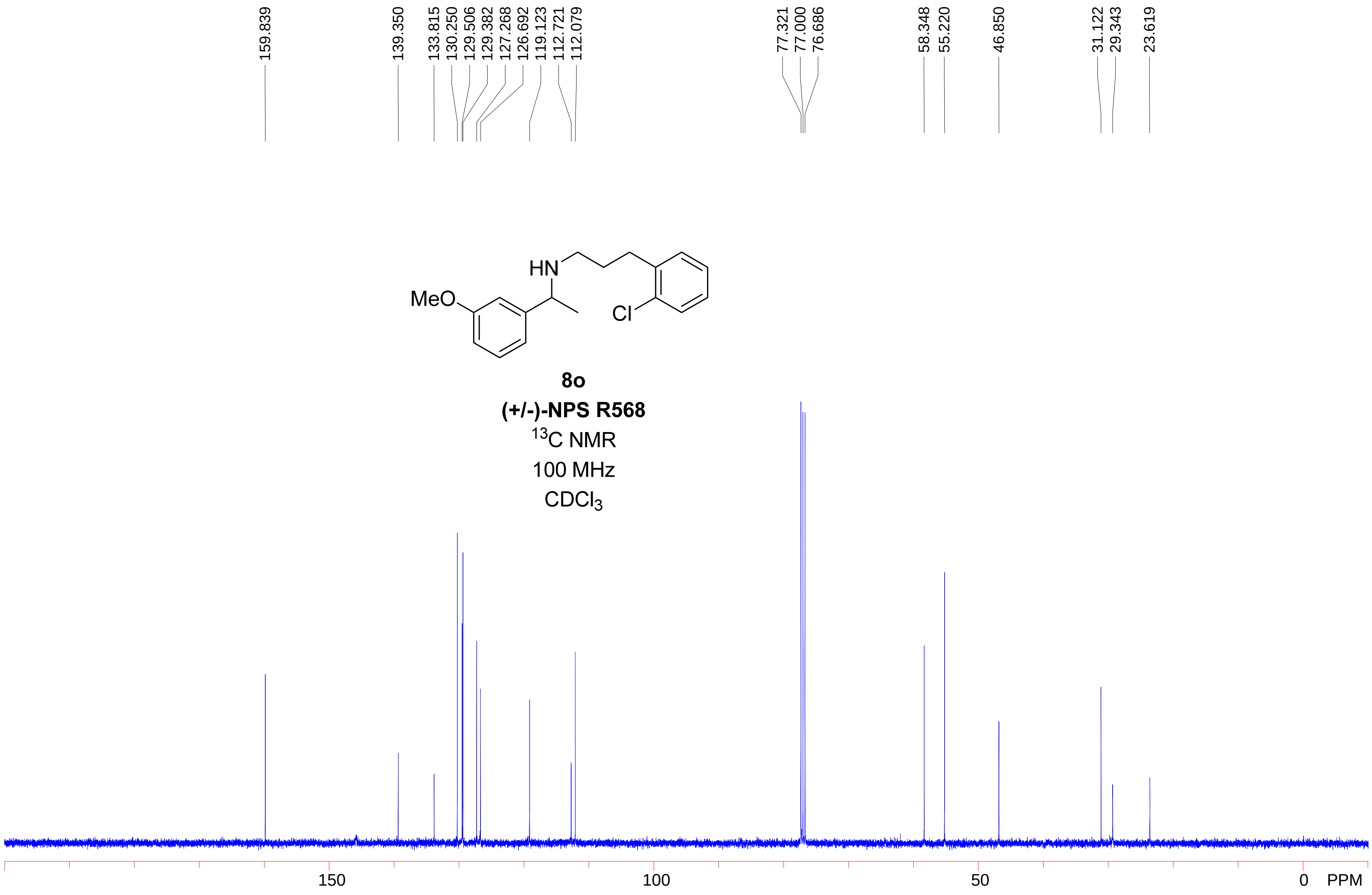

Supplementary Figure 122. ^{13}C NMR spectrum for **8o**

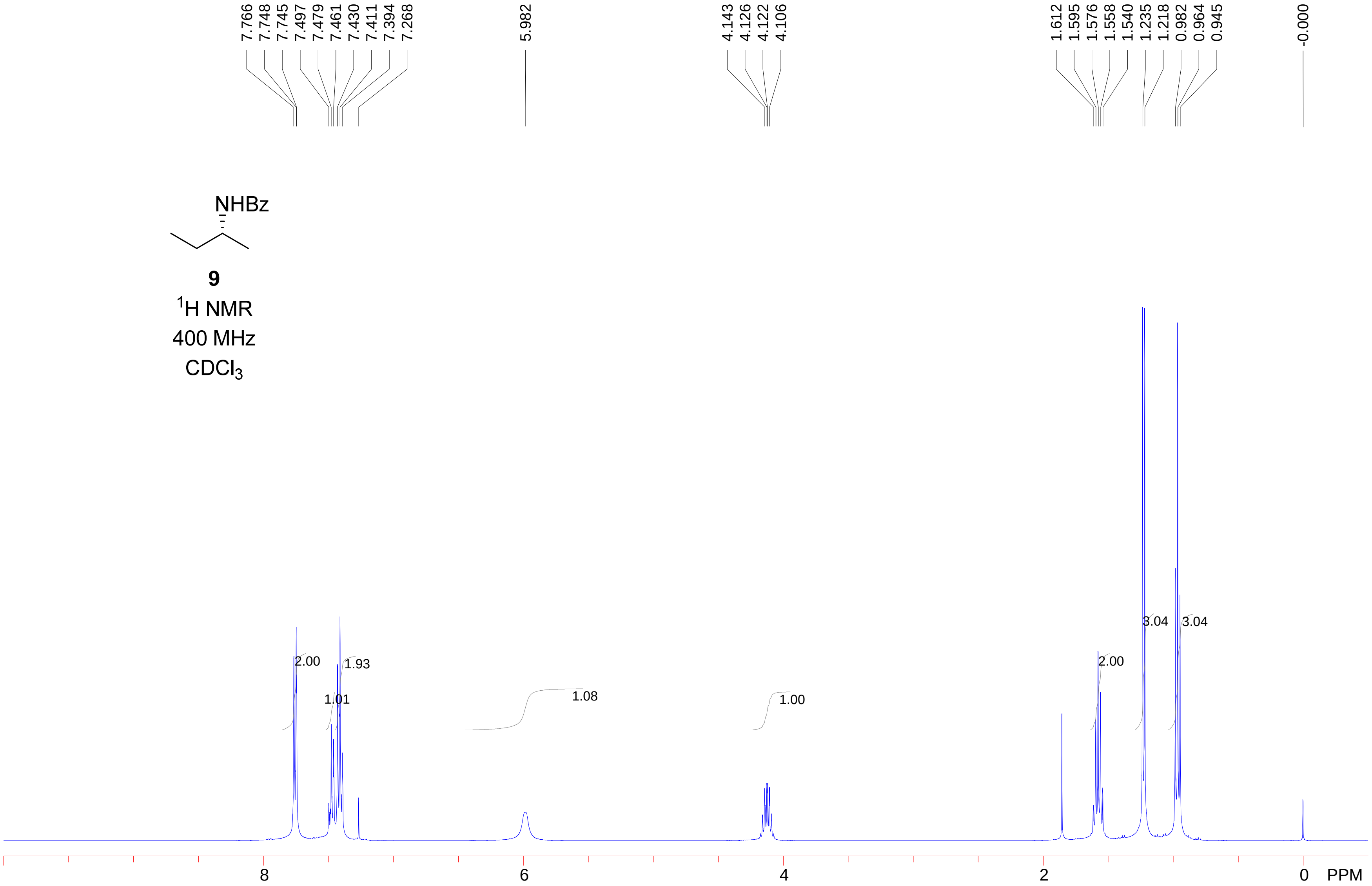

Supplementary Figure 123. ^{1}H NMR spectrum for **9**

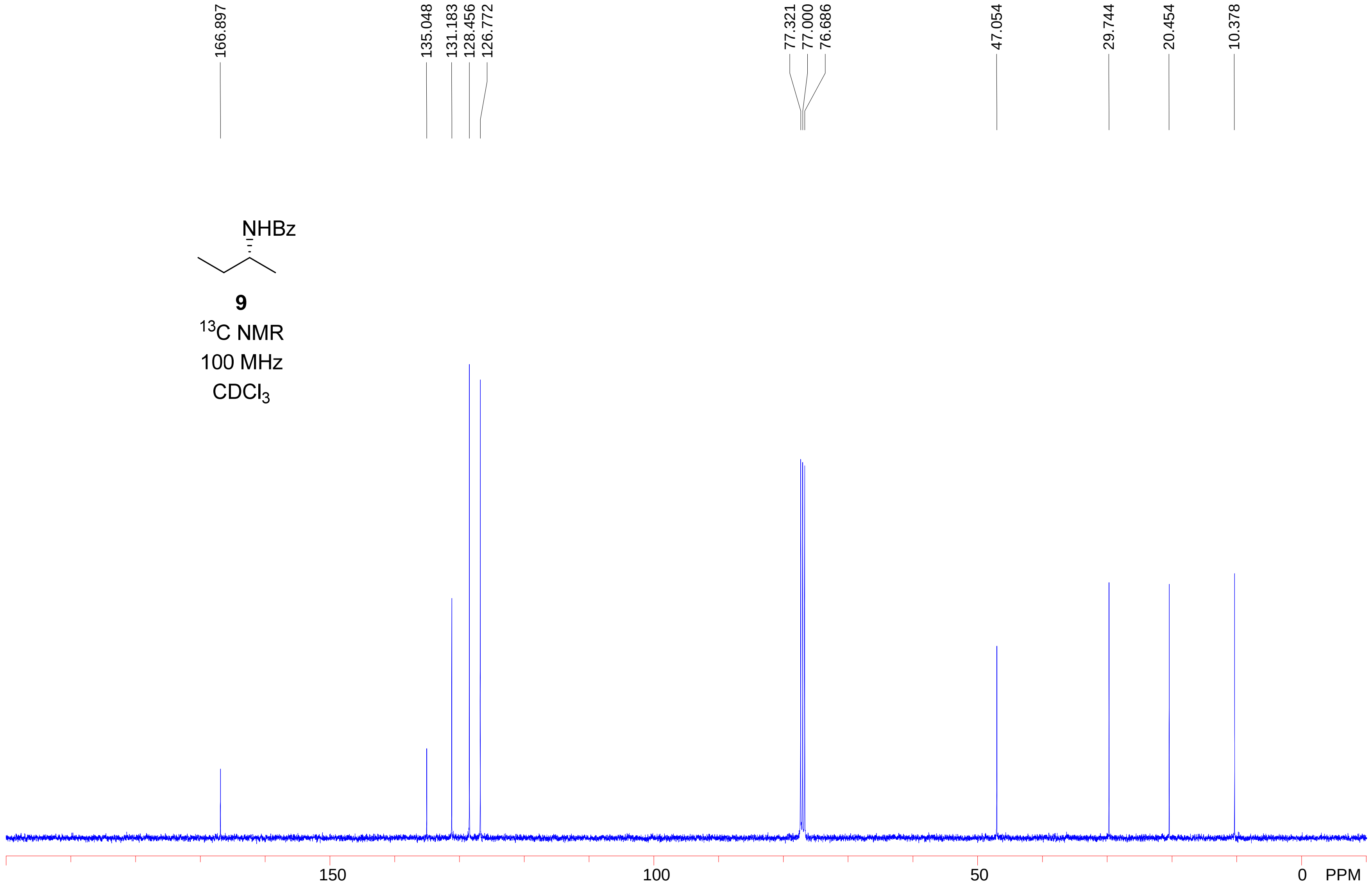

Supplementary Figure 124. ^{13}C NMR spectrum for **9**

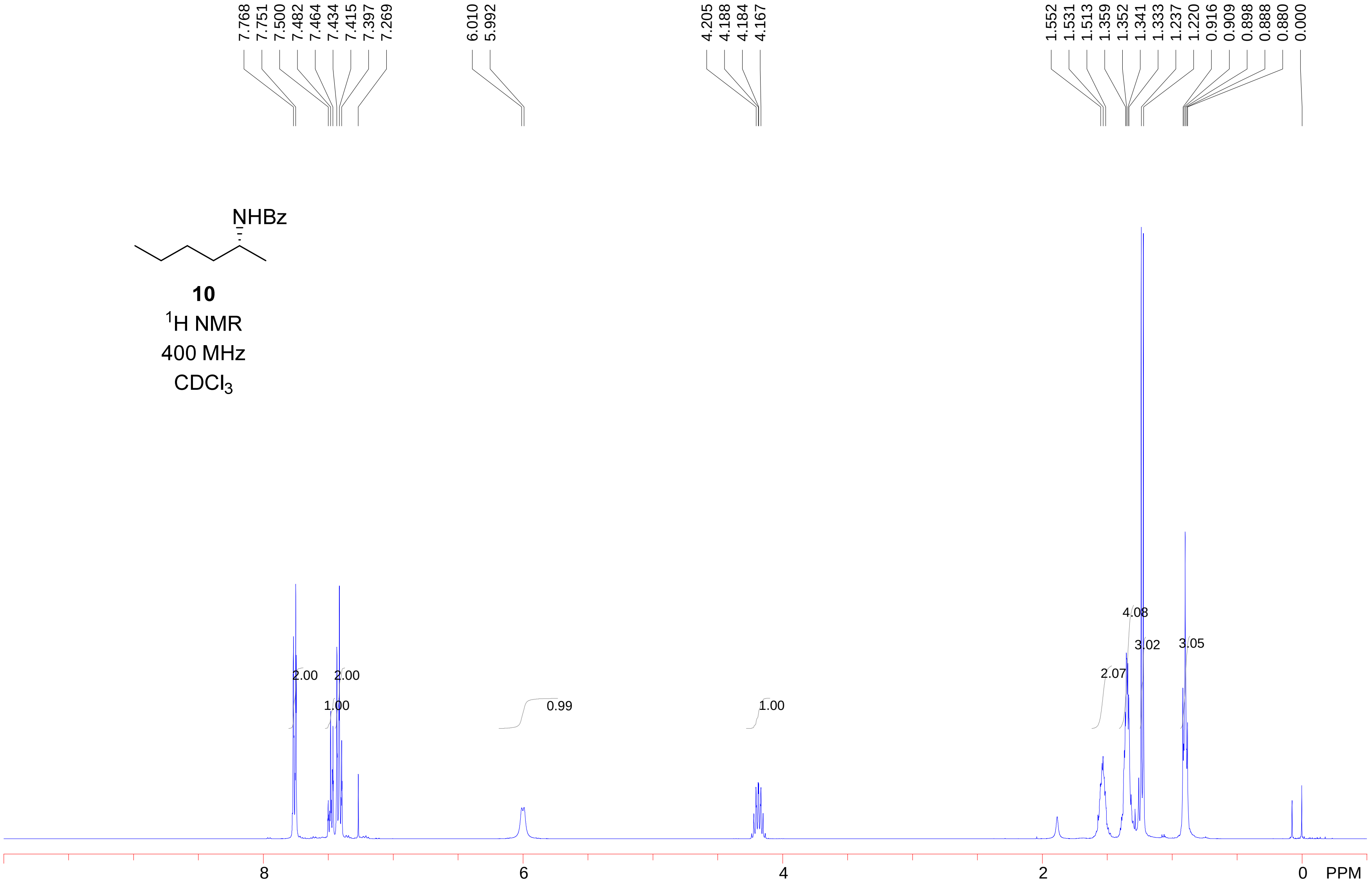

Supplementary Figure 125. ^{1}H NMR spectrum for **10**

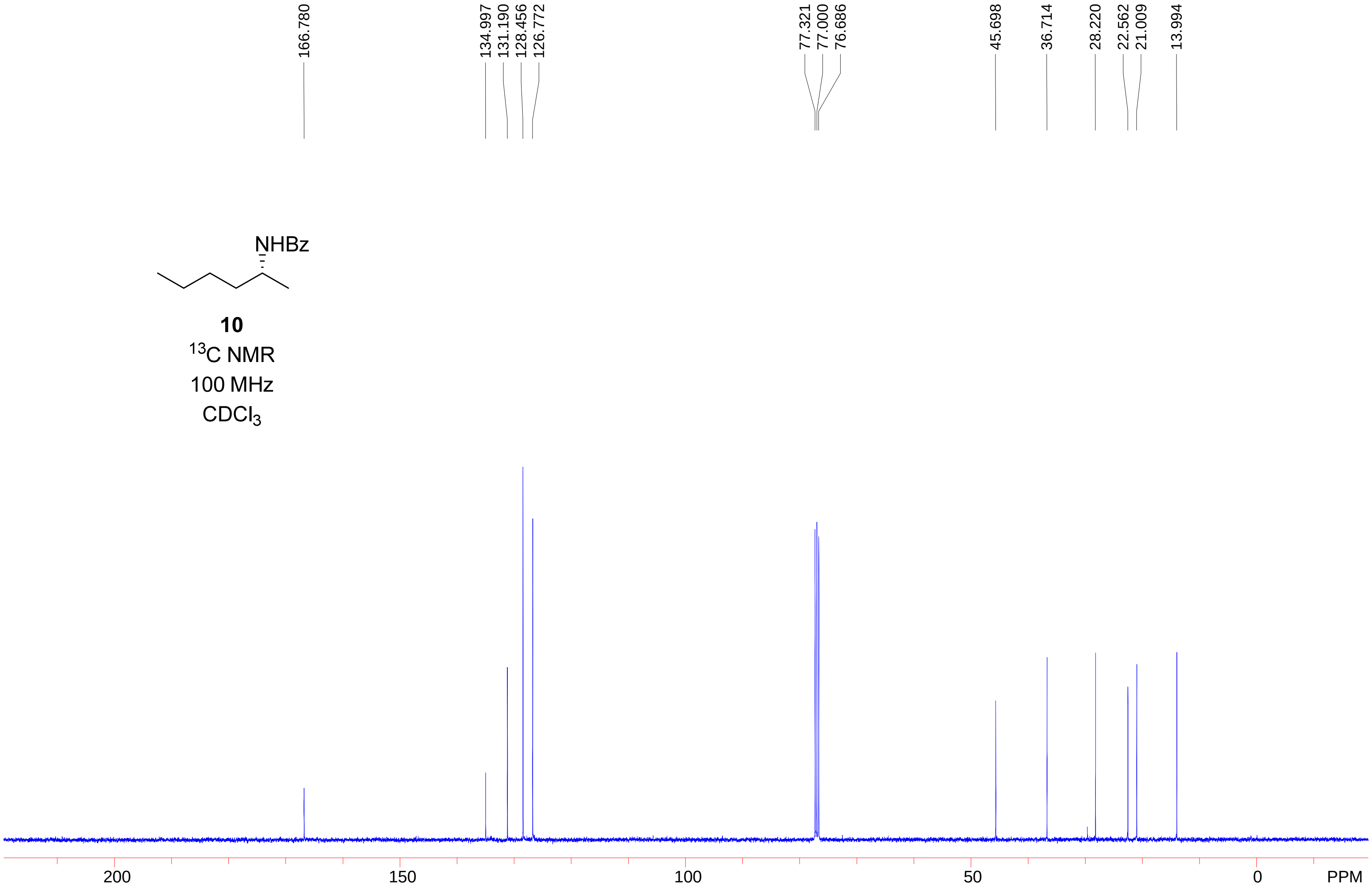

Supplementary Figure 126. ^{13}C NMR spectrum for **10**

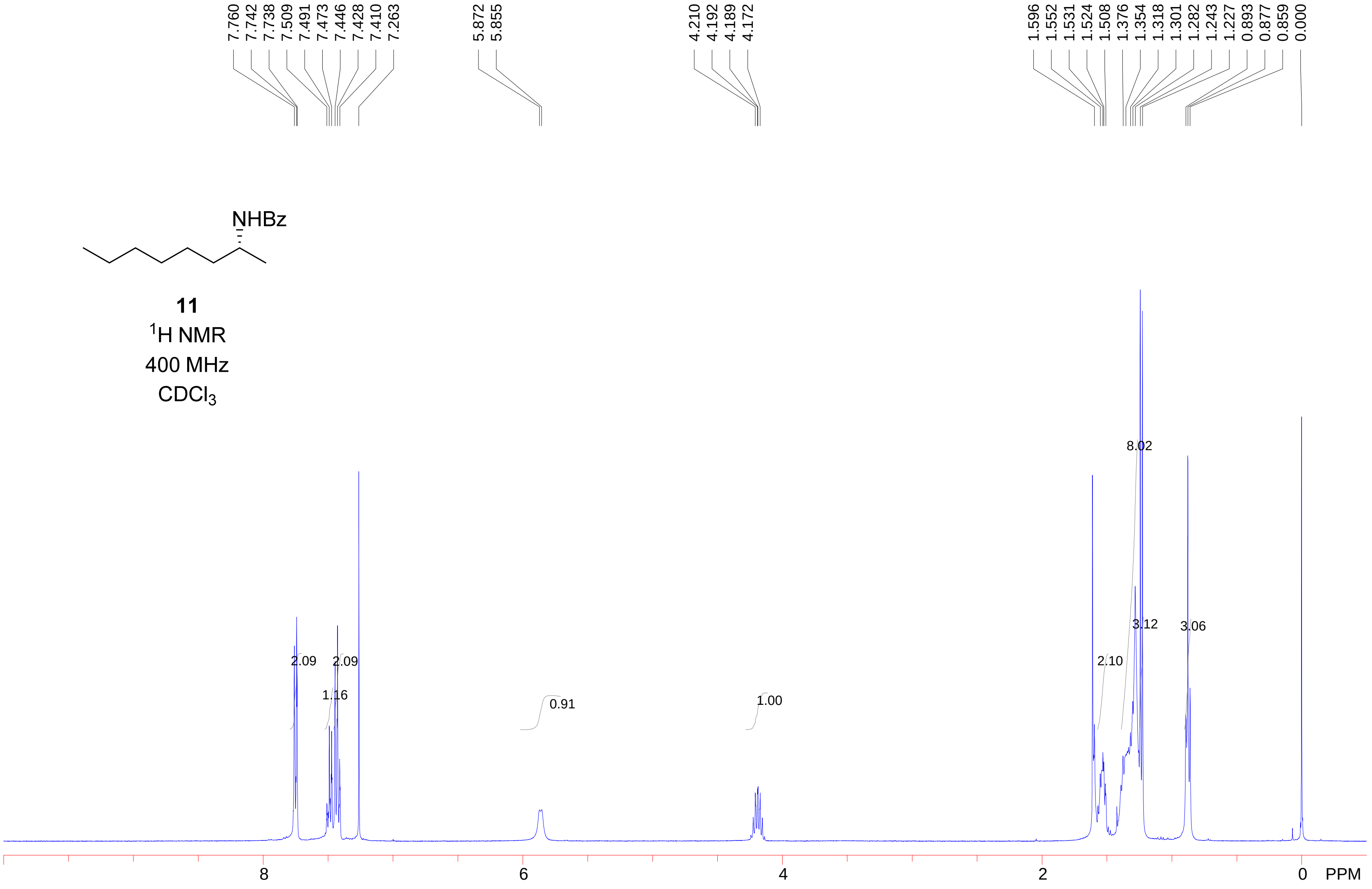

Supplementary Figure 127. ^{1}H NMR spectrum for **11**

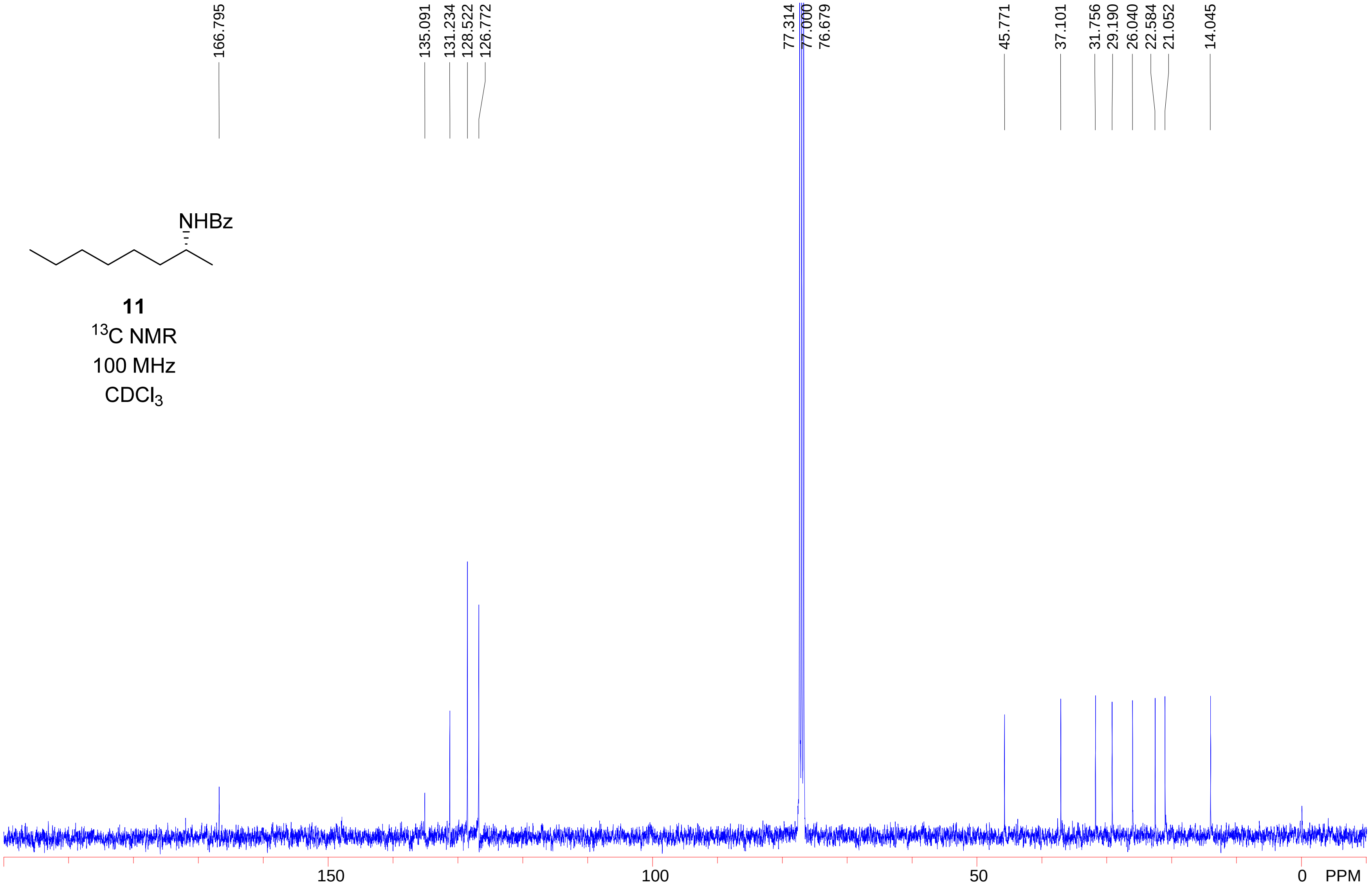

Supplementary Figure 128. ^{13}C NMR spectrum for **11**

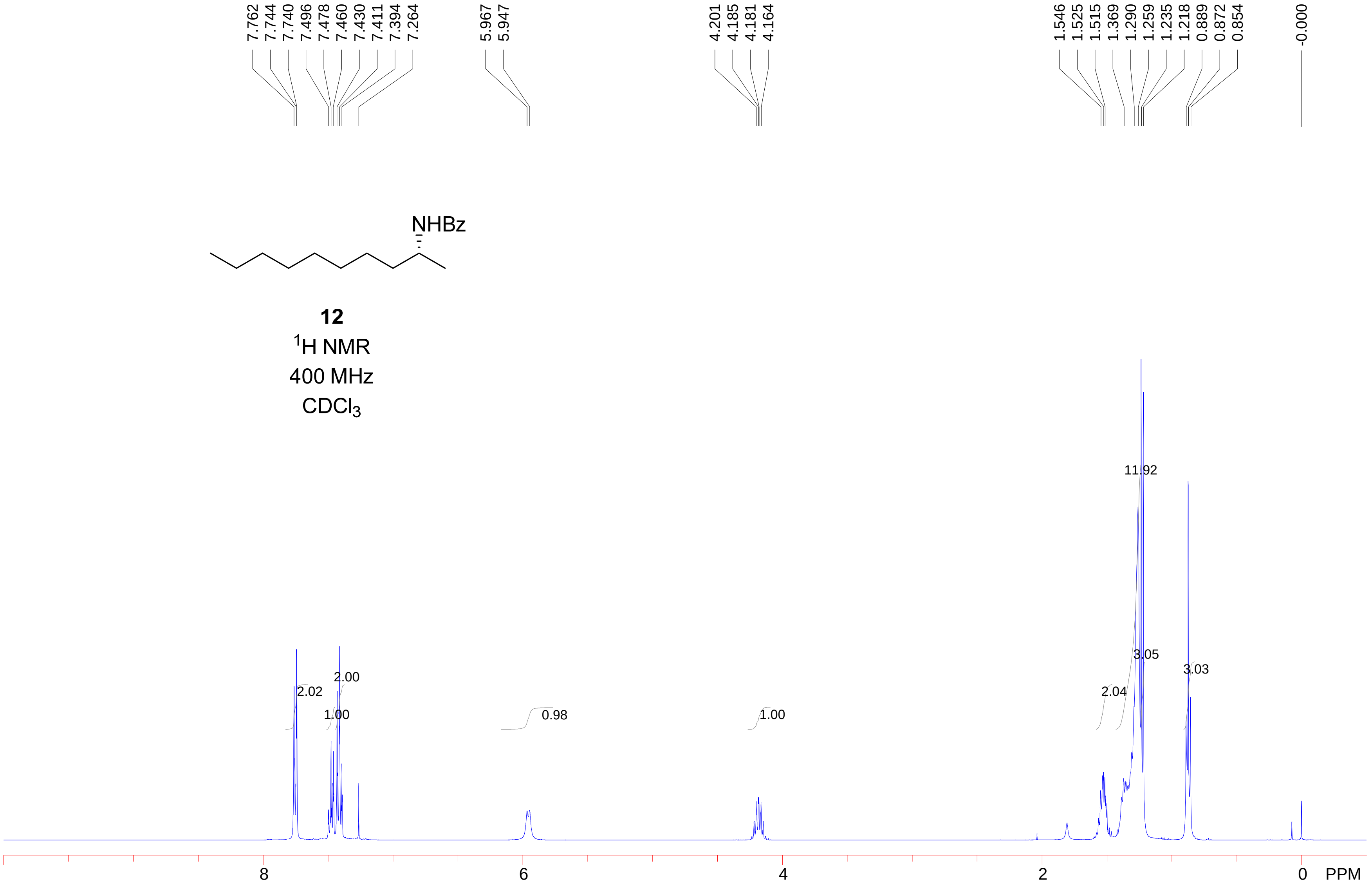

Supplementary Figure 129. ^{1}H NMR spectrum for **12**

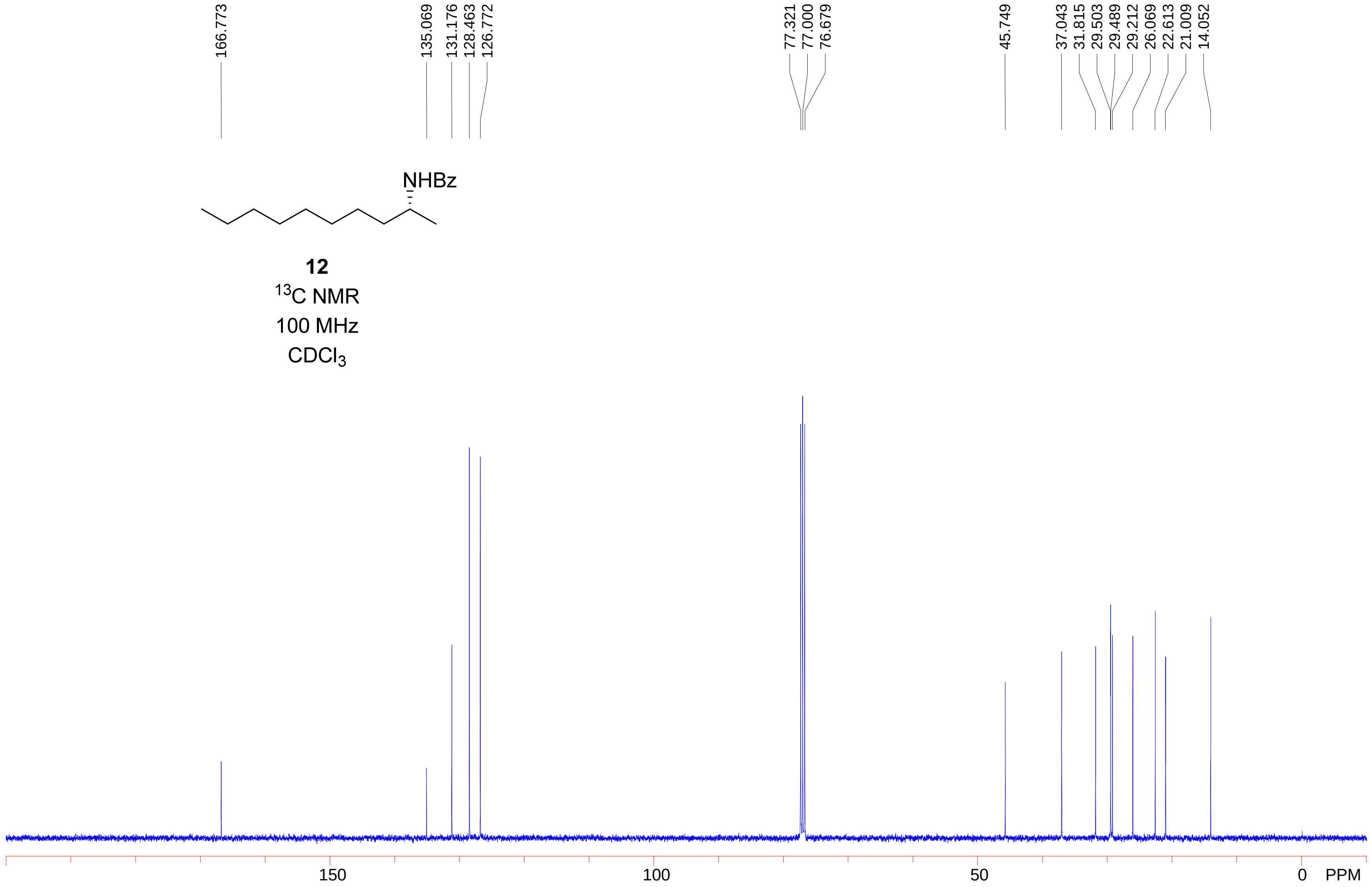

Supplementary Figure 130. ^{13}C NMR spectrum for **12**

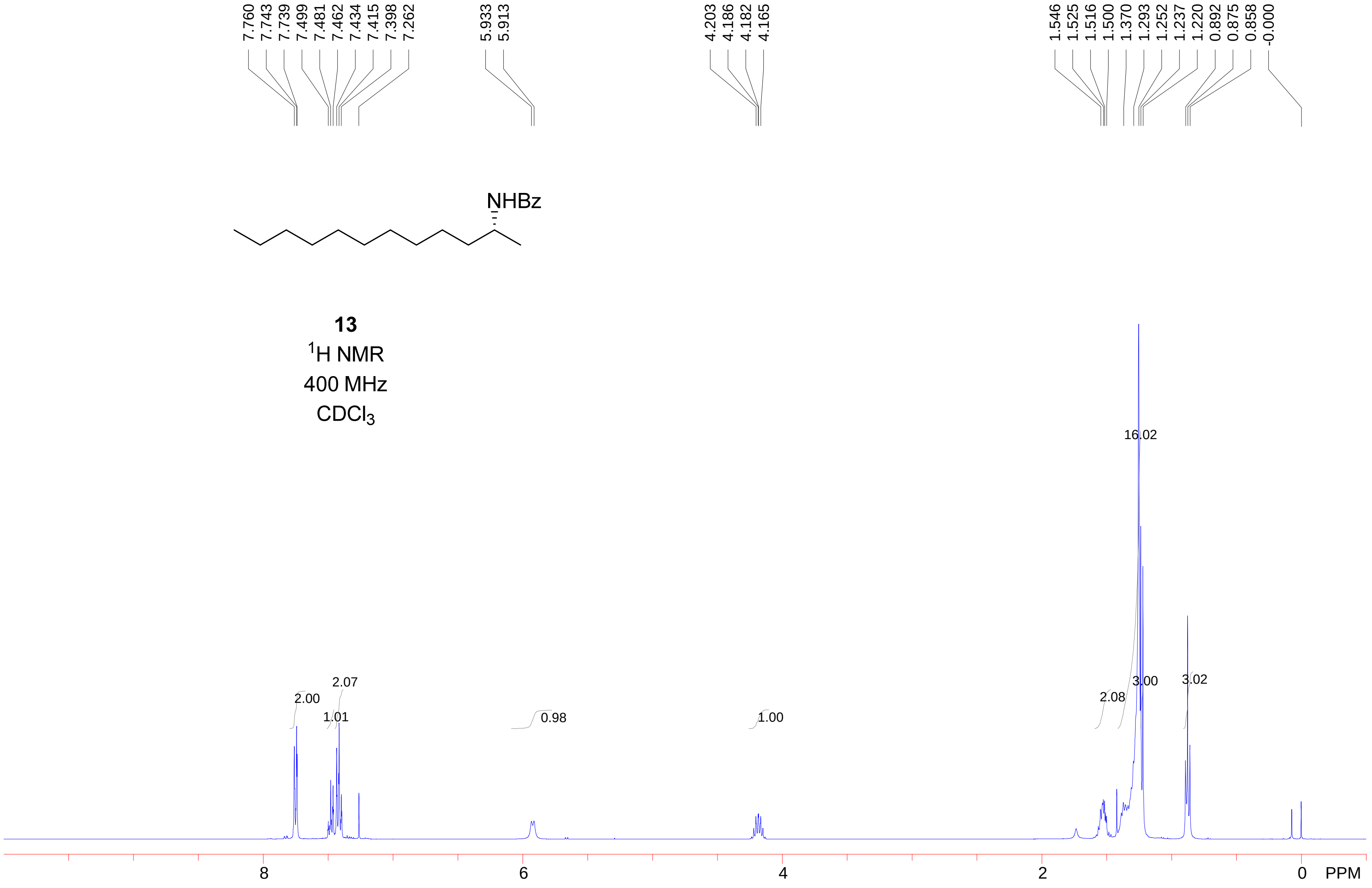

Supplementary Figure 131. ^{1}H NMR spectrum for **13**

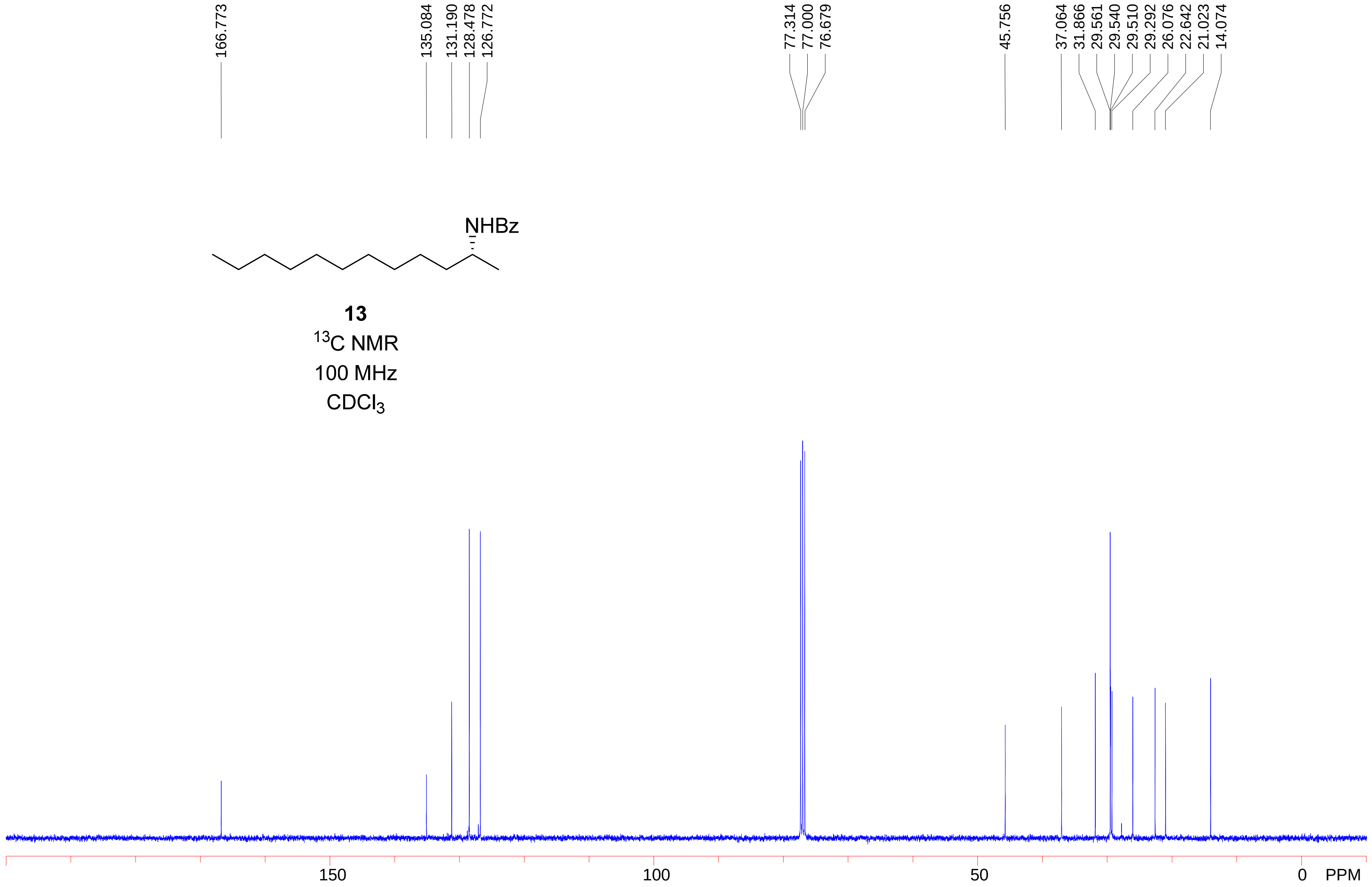

Supplementary Figure 132. ^{13}C NMR spectrum for **13**

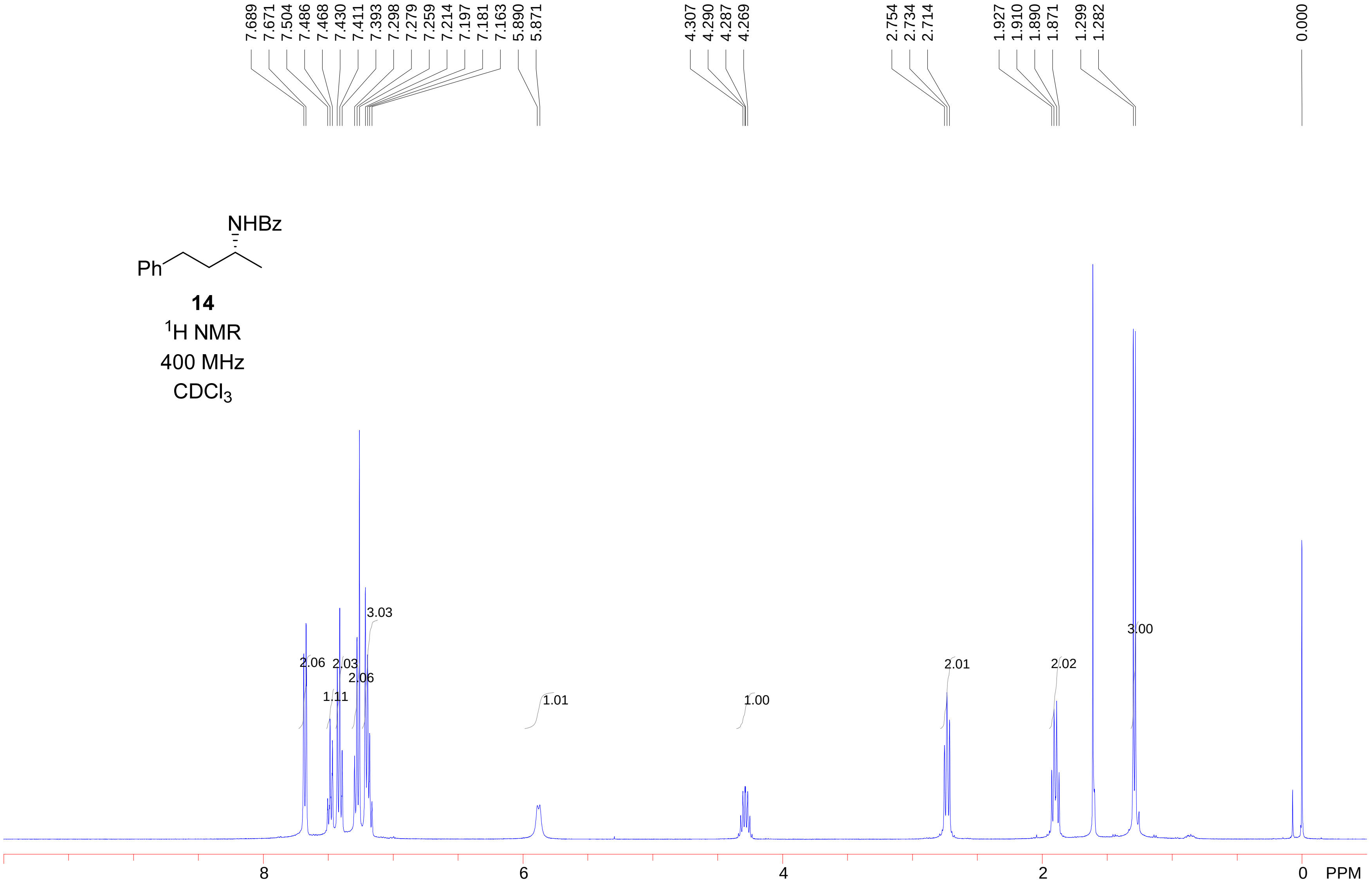

Supplementary Figure 133. ^{1}H NMR spectrum for **14**

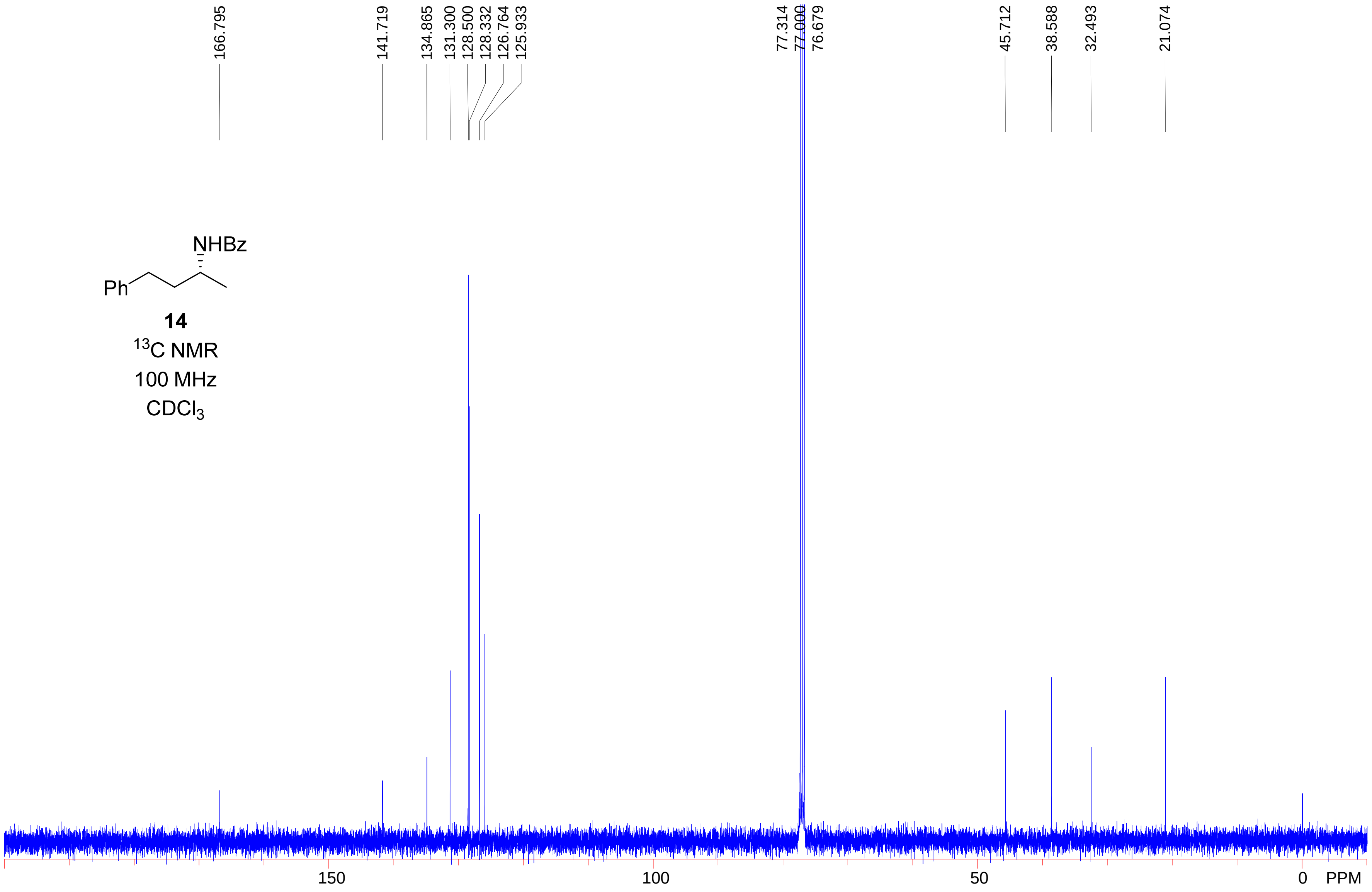

Supplementary Figure 134. ^{13}C NMR spectrum for **14**

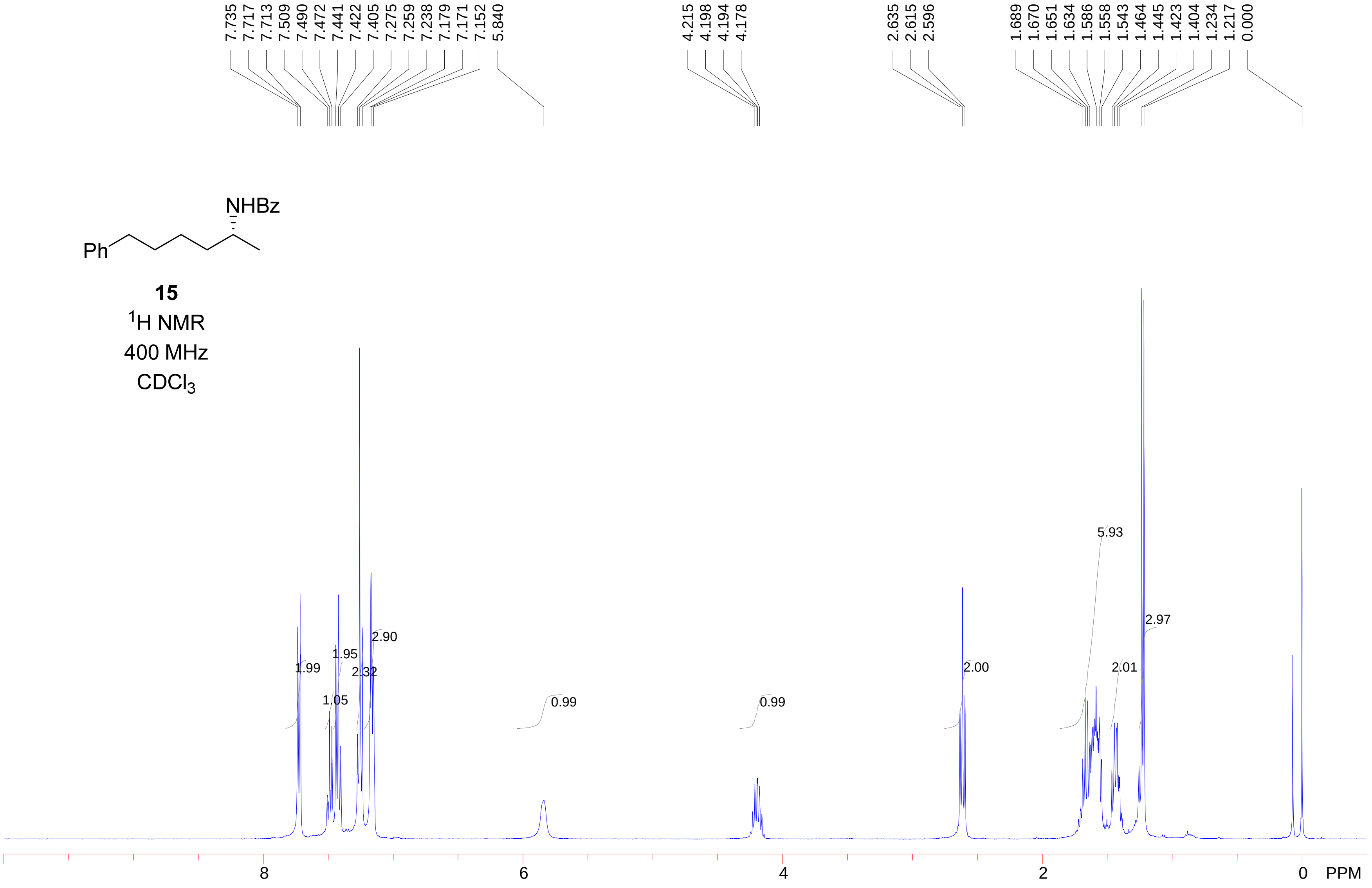

Supplementary Figure 135. ^{1}H NMR spectrum for **15**

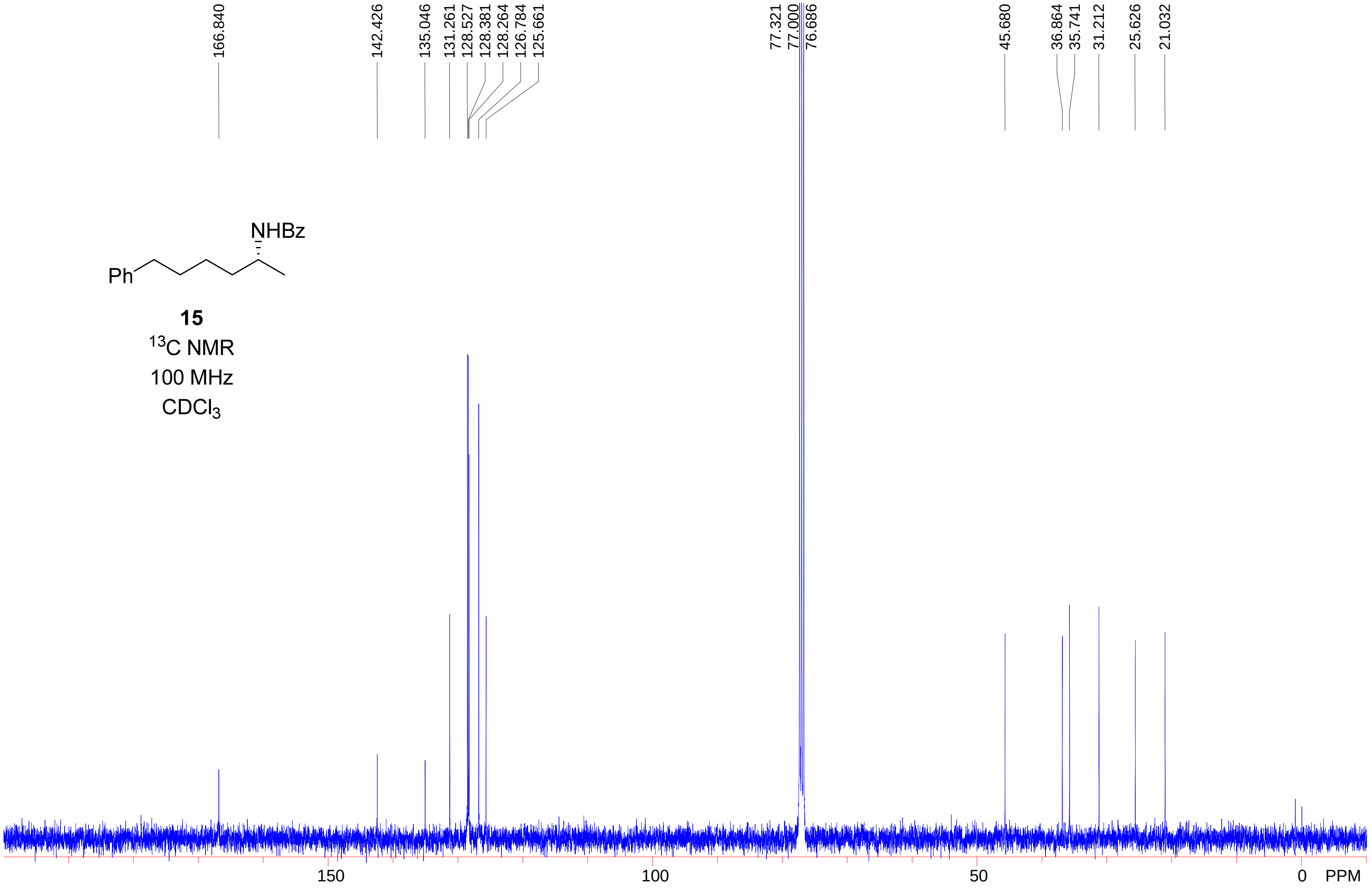

Supplementary Figure 136. ^{13}C NMR spectrum for **15**

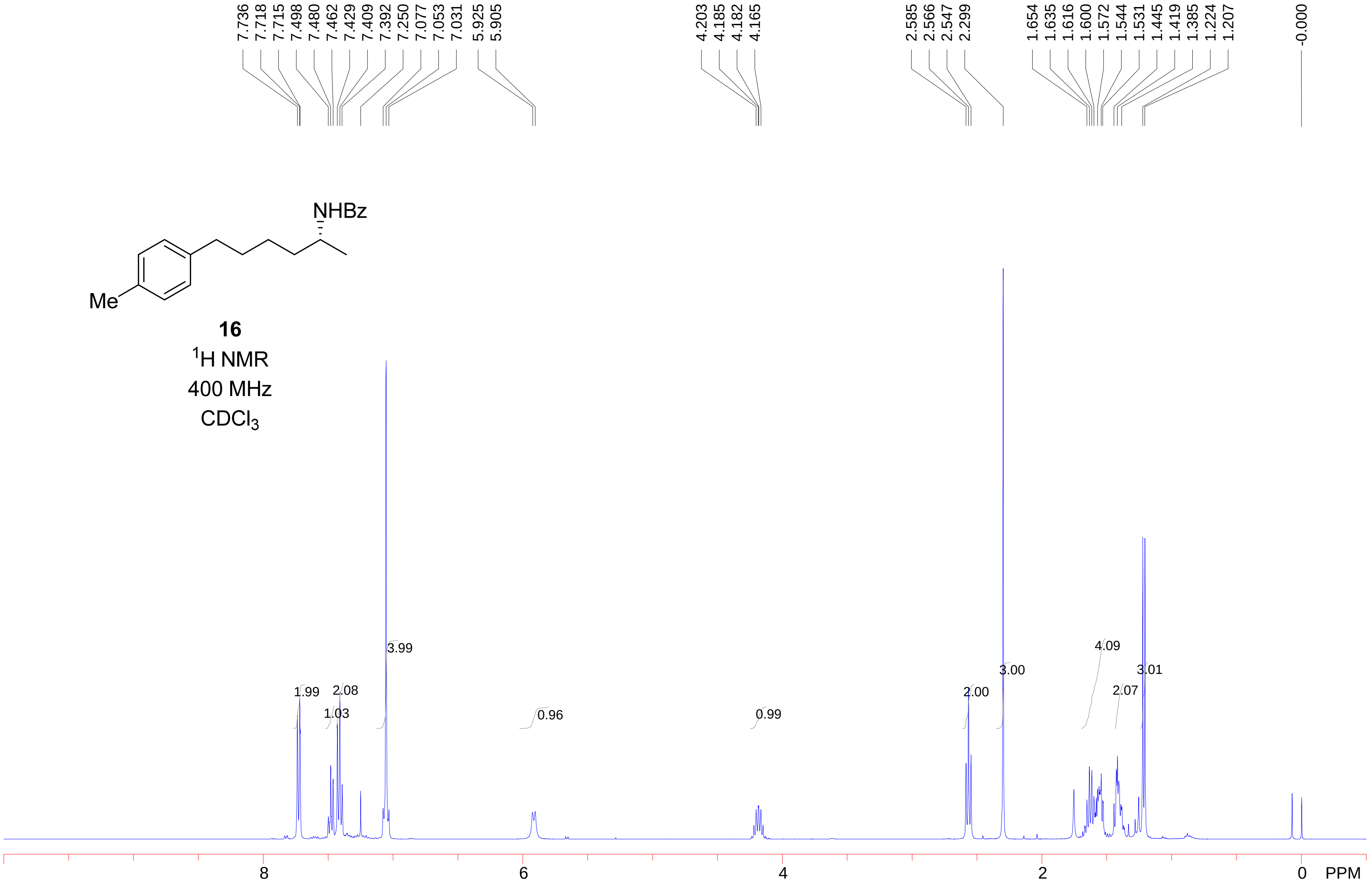

Supplementary Figure 137. ^{1}H NMR spectrum for **16**

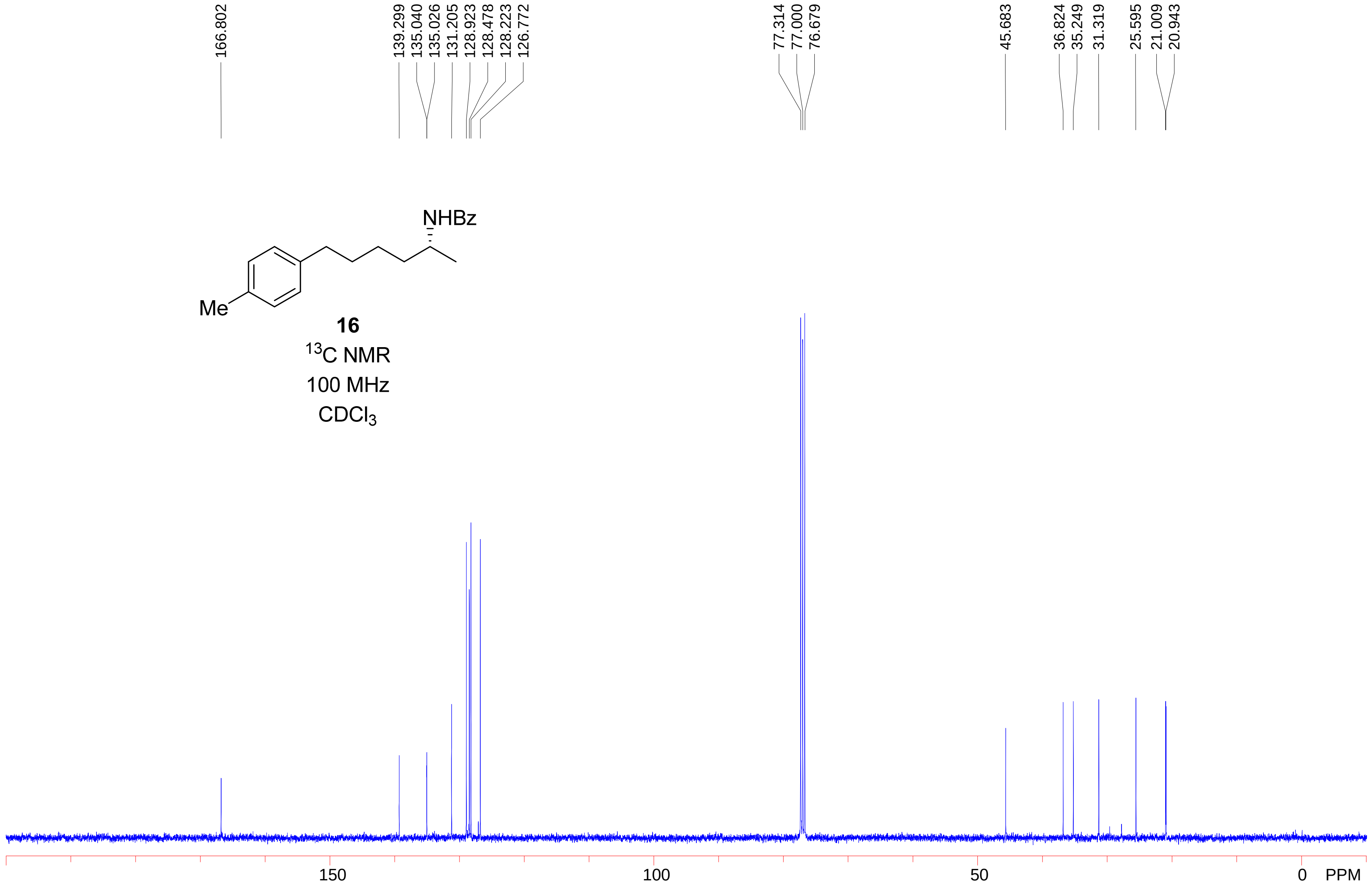

Supplementary Figure 138. ^{13}C NMR spectrum for **16**

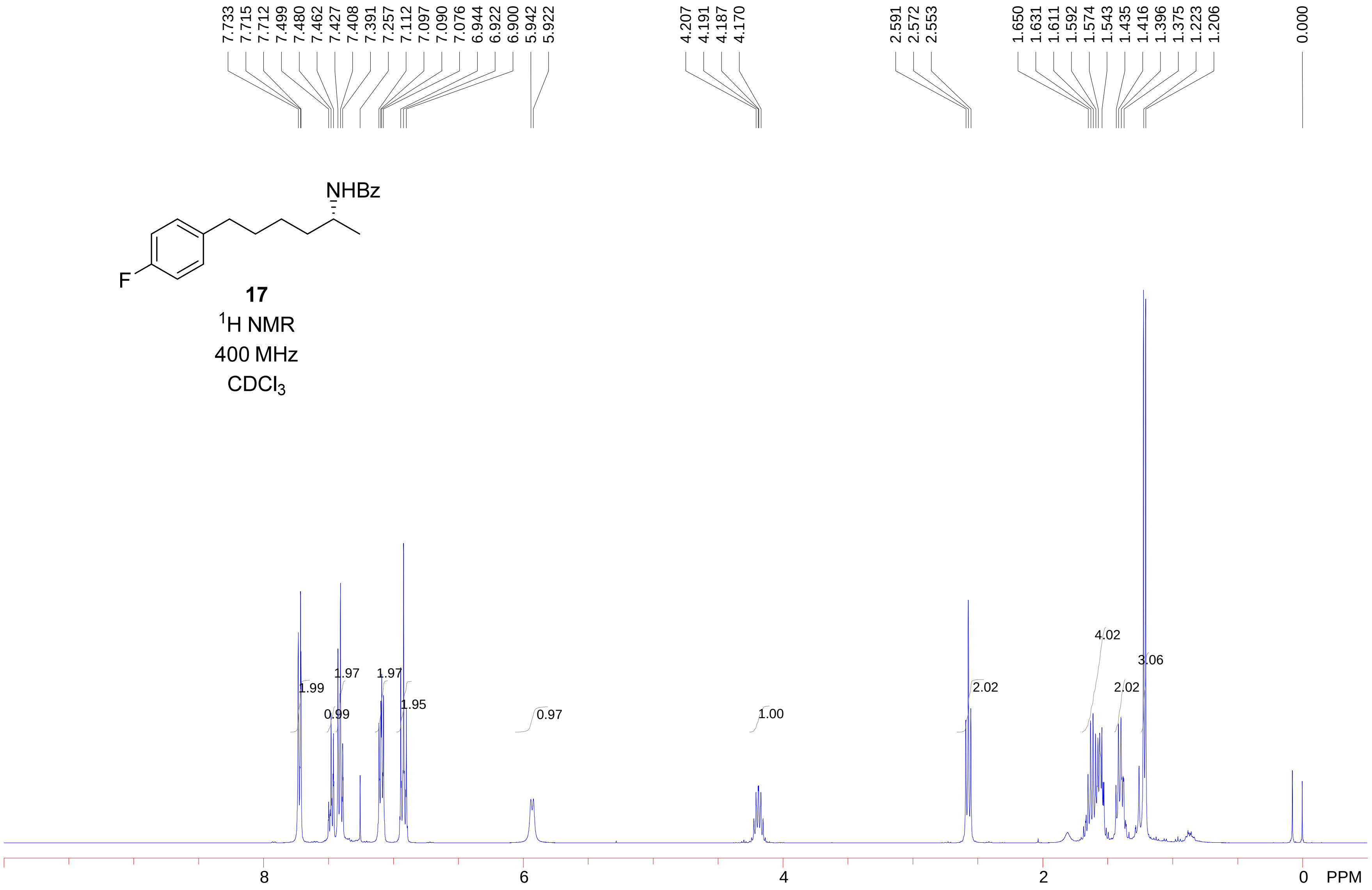

Supplementary Figure 139. ^{1}H NMR spectrum for **17**

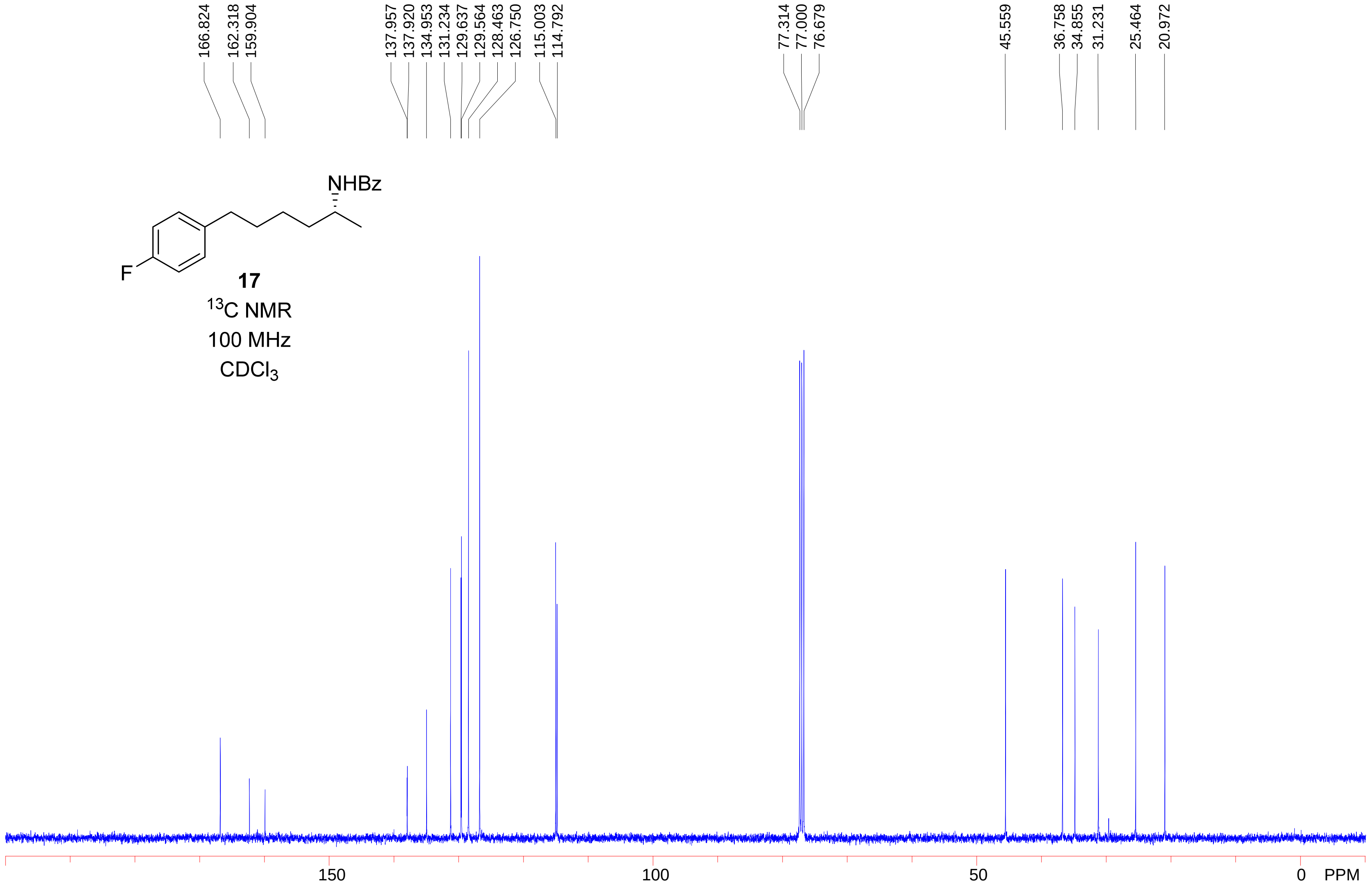

Supplementary Figure 140. ^{13}C NMR spectrum for **17**

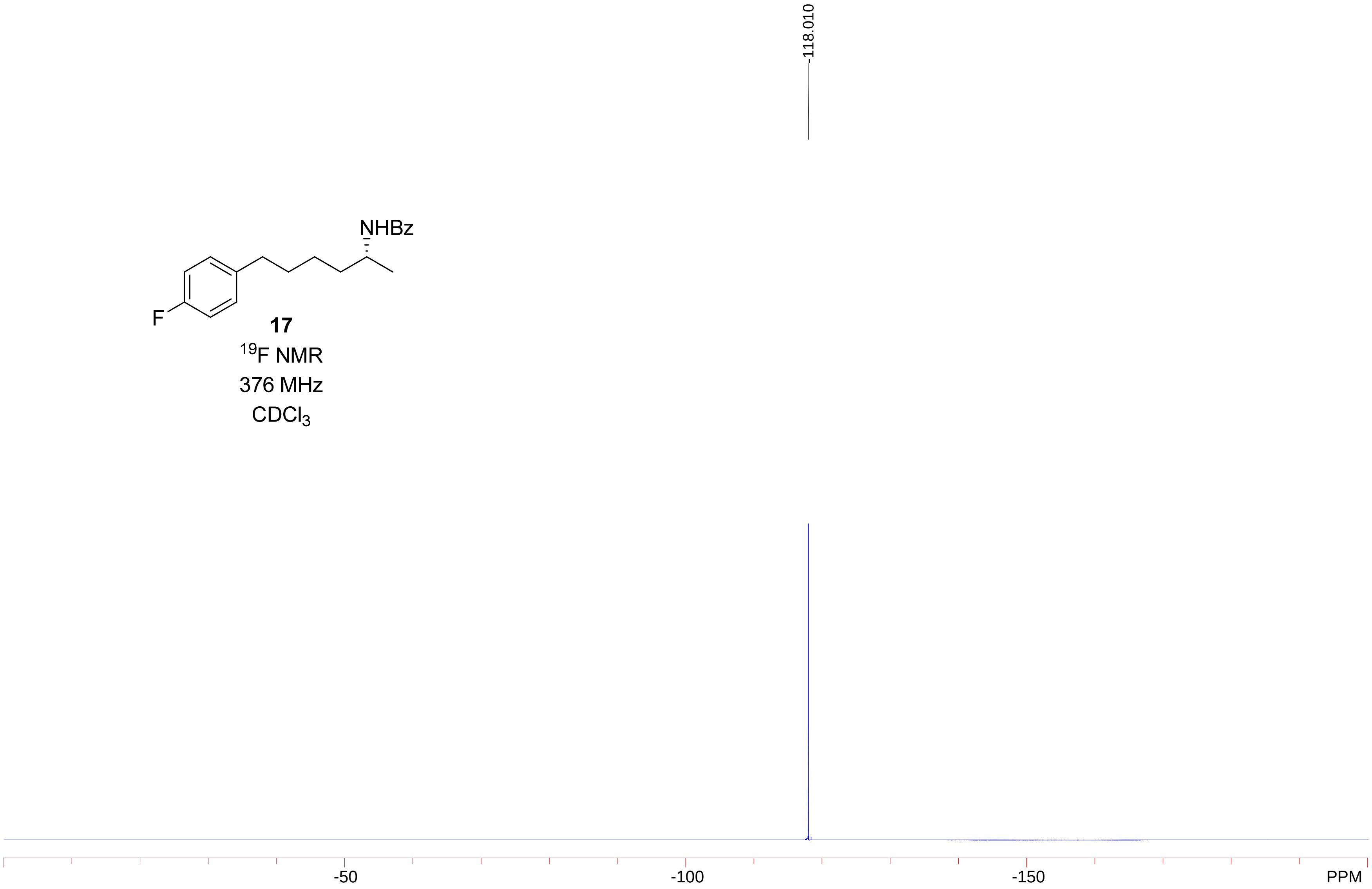

17
^{19}F NMR
376 MHz
$CDCl_3$

Supplementary Figure 141. ^{19}F NMR spectrum for **17**

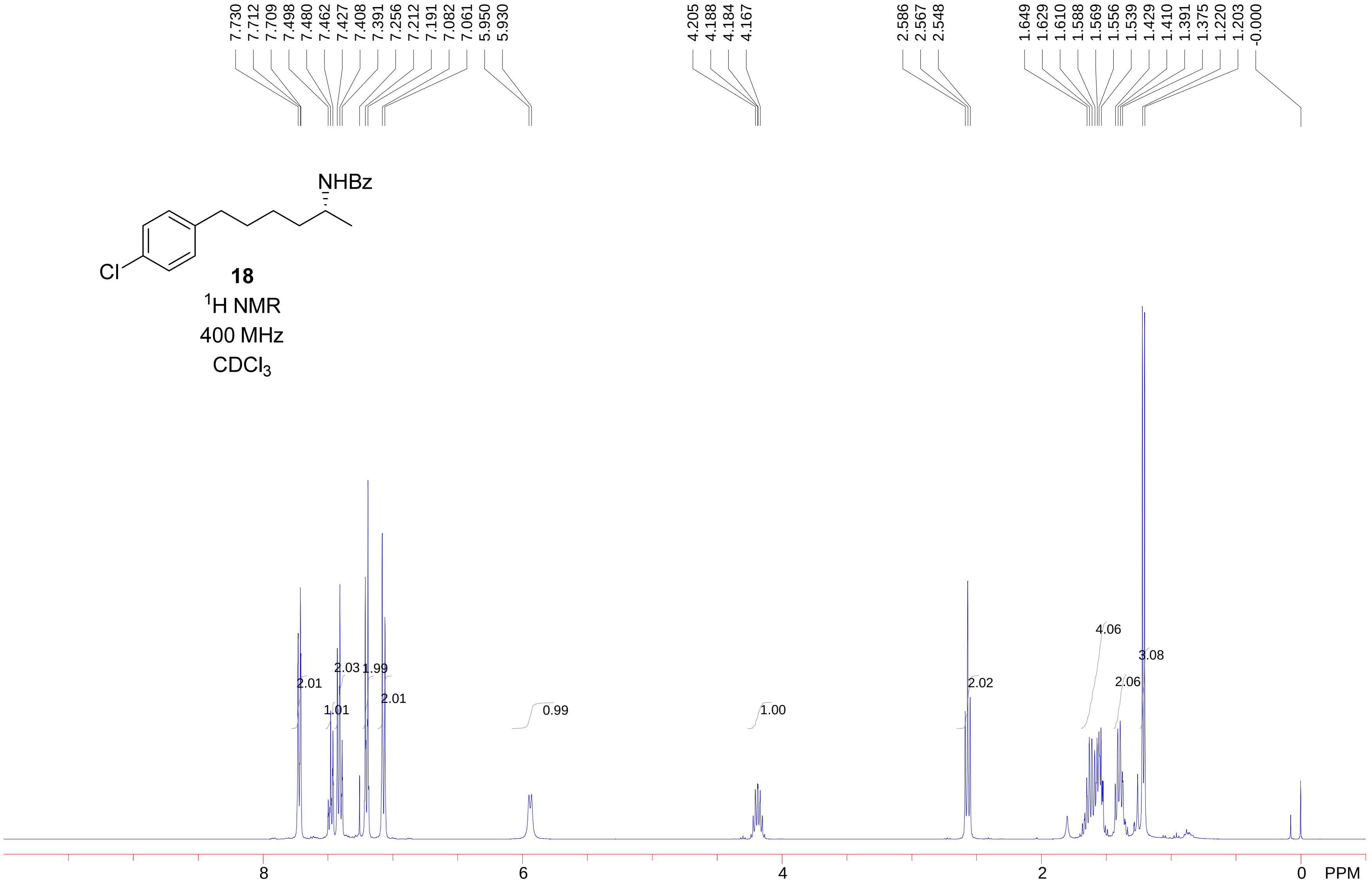

Supplementary Figure 142. ^{1}H NMR spectrum for **18**

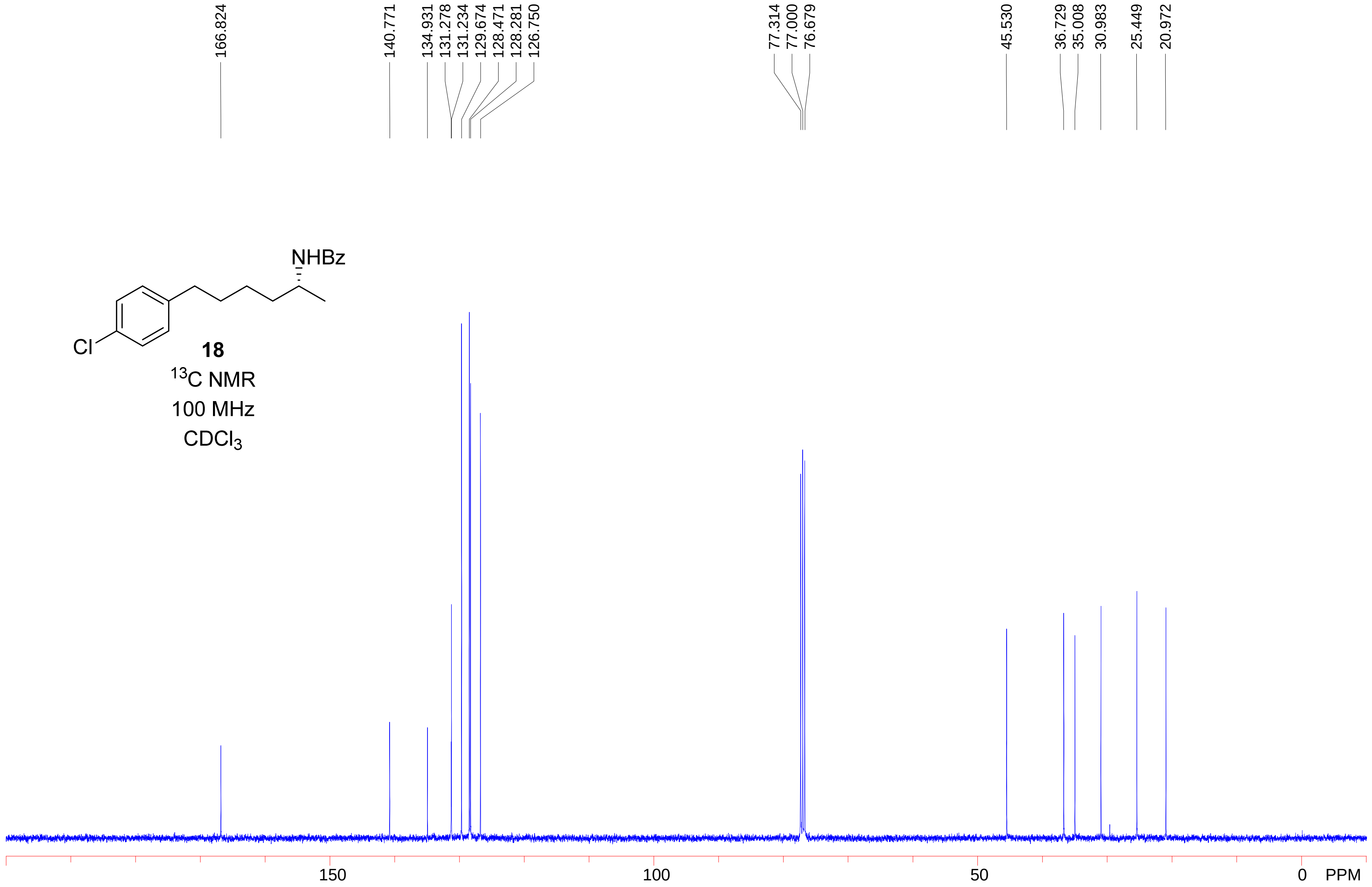

Supplementary Figure 143. ^{13}C NMR spectrum for **18**

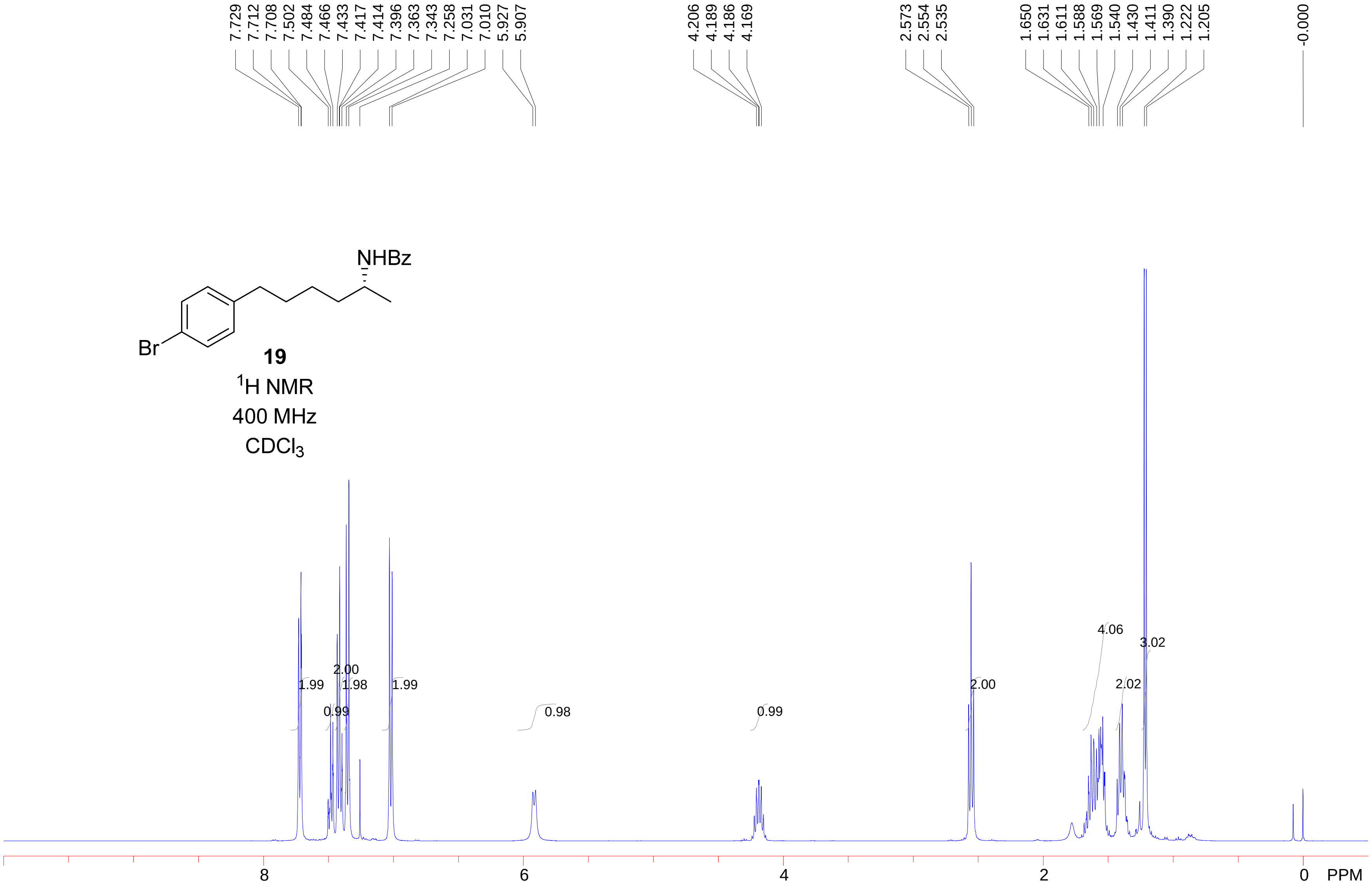

Supplementary Figure 144. ^{1}H NMR spectrum for **19**

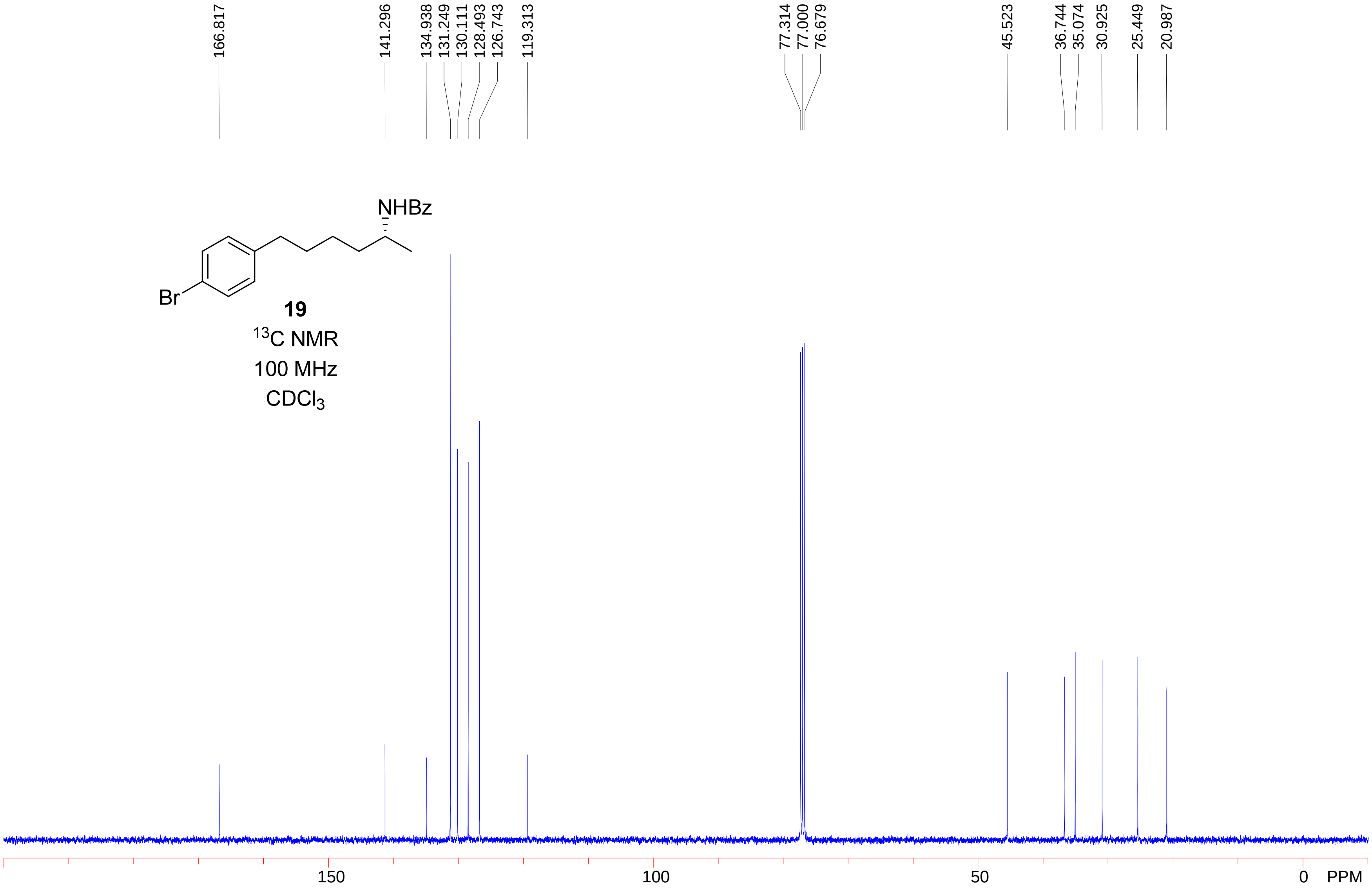

Supplementary Figure 145. ^{13}C NMR spectrum for **19**

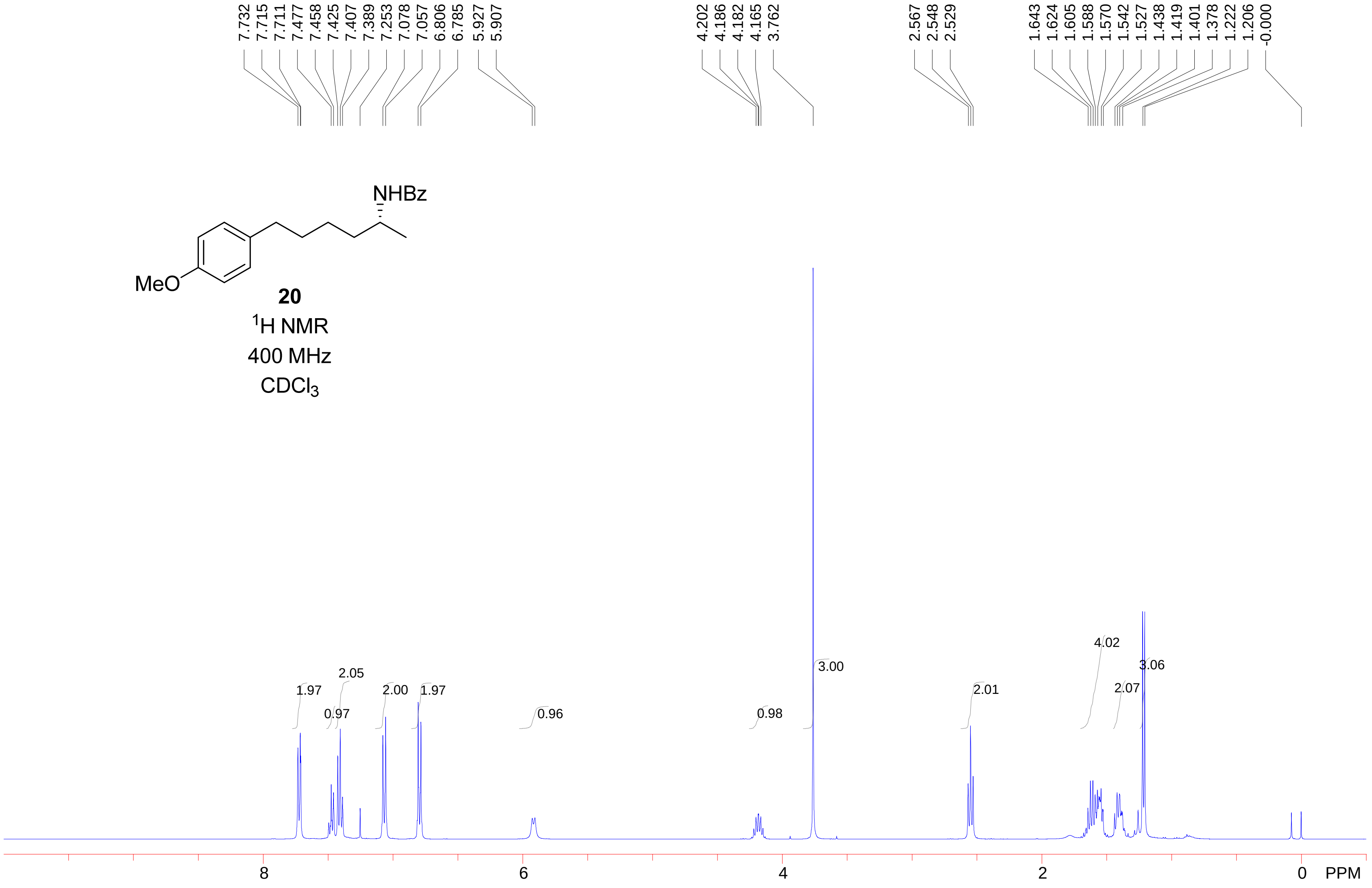

Supplementary Figure 146. ^{1}H NMR spectrum for **20**

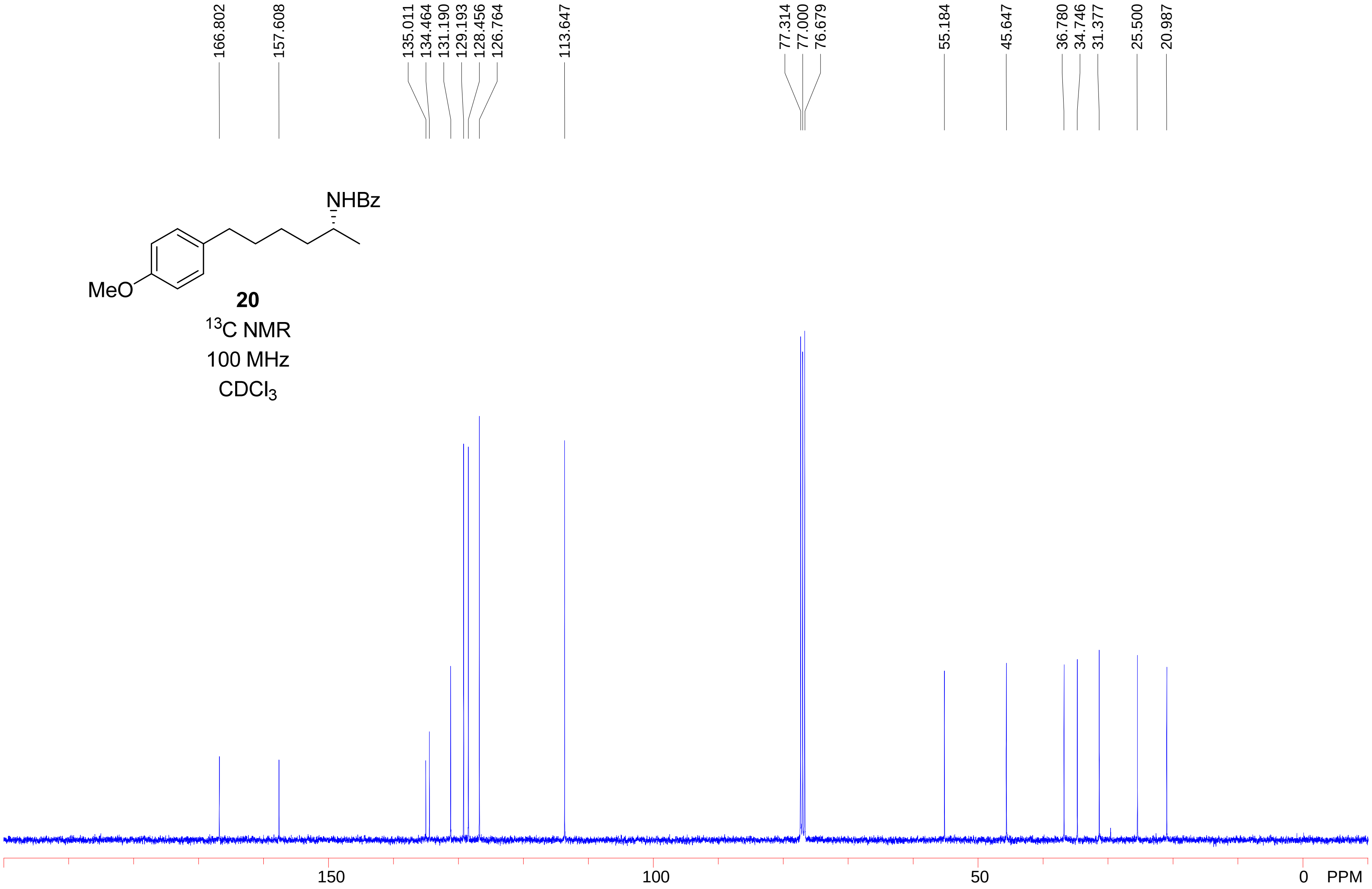

Supplementary Figure 147. ^{13}C NMR spectrum for **20**

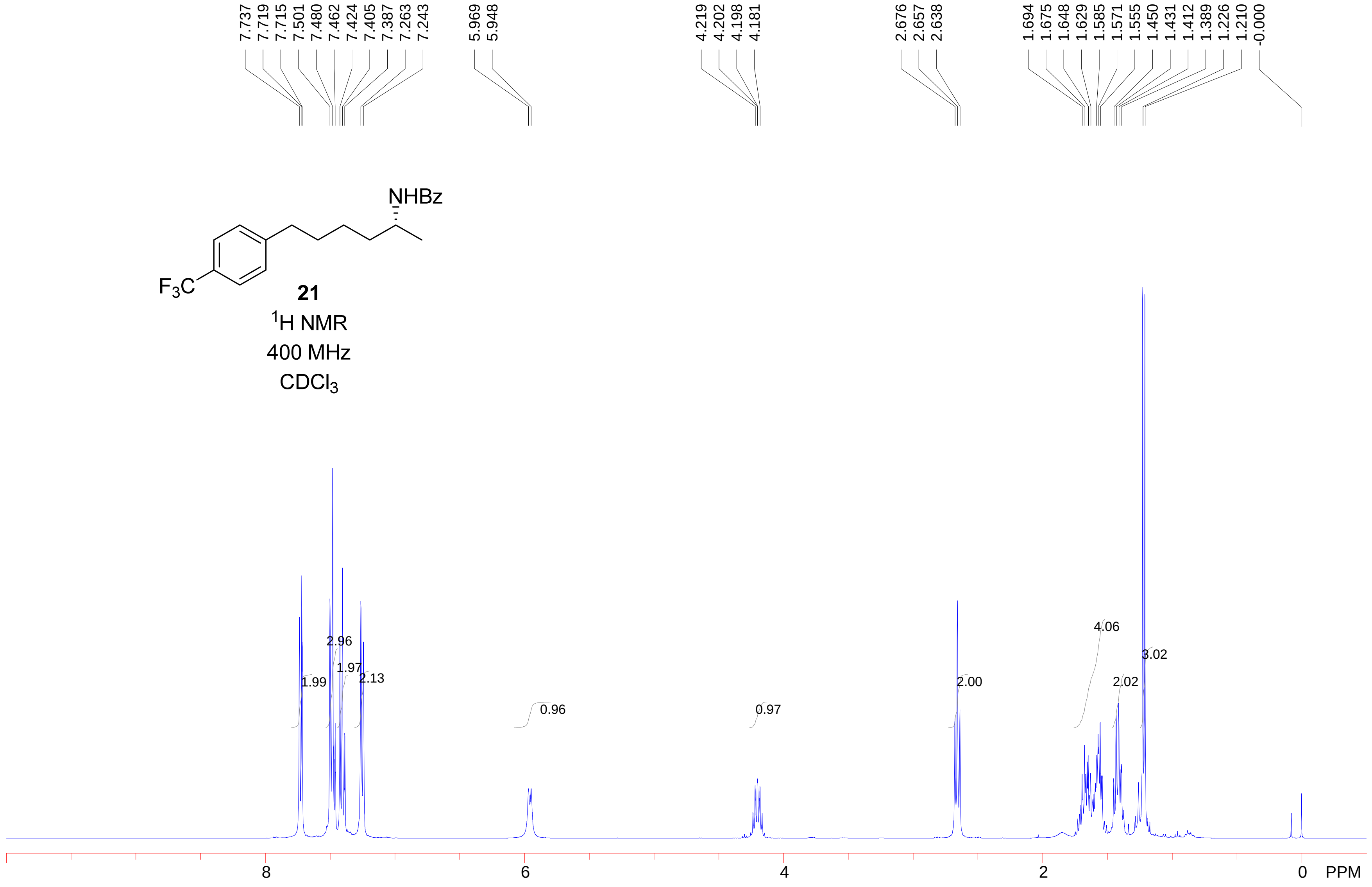

Supplementary Figure 148. ^{1}H NMR spectrum for **21**

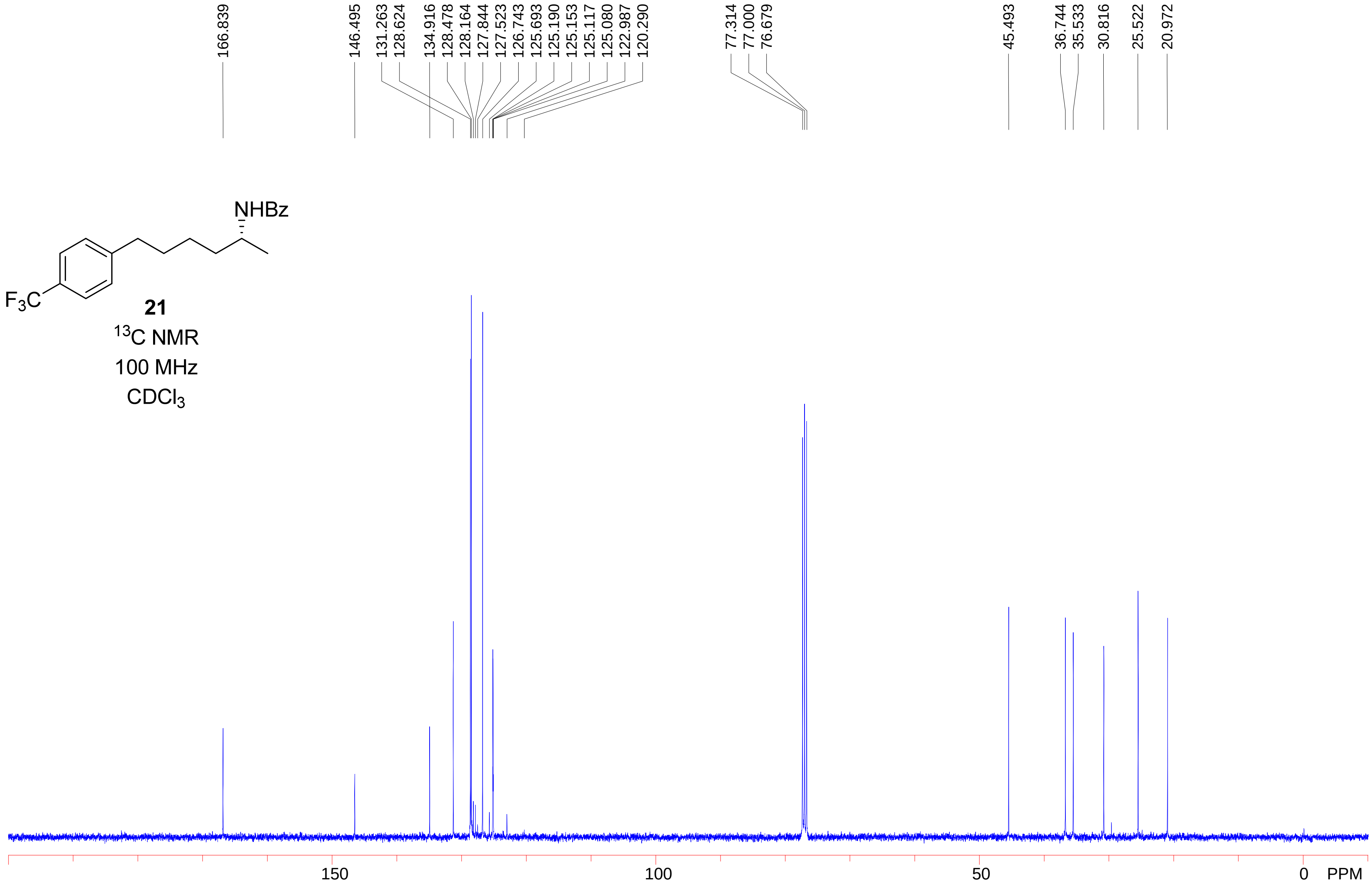

Supplementary Figure 149. ^{13}C NMR spectrum for **21**

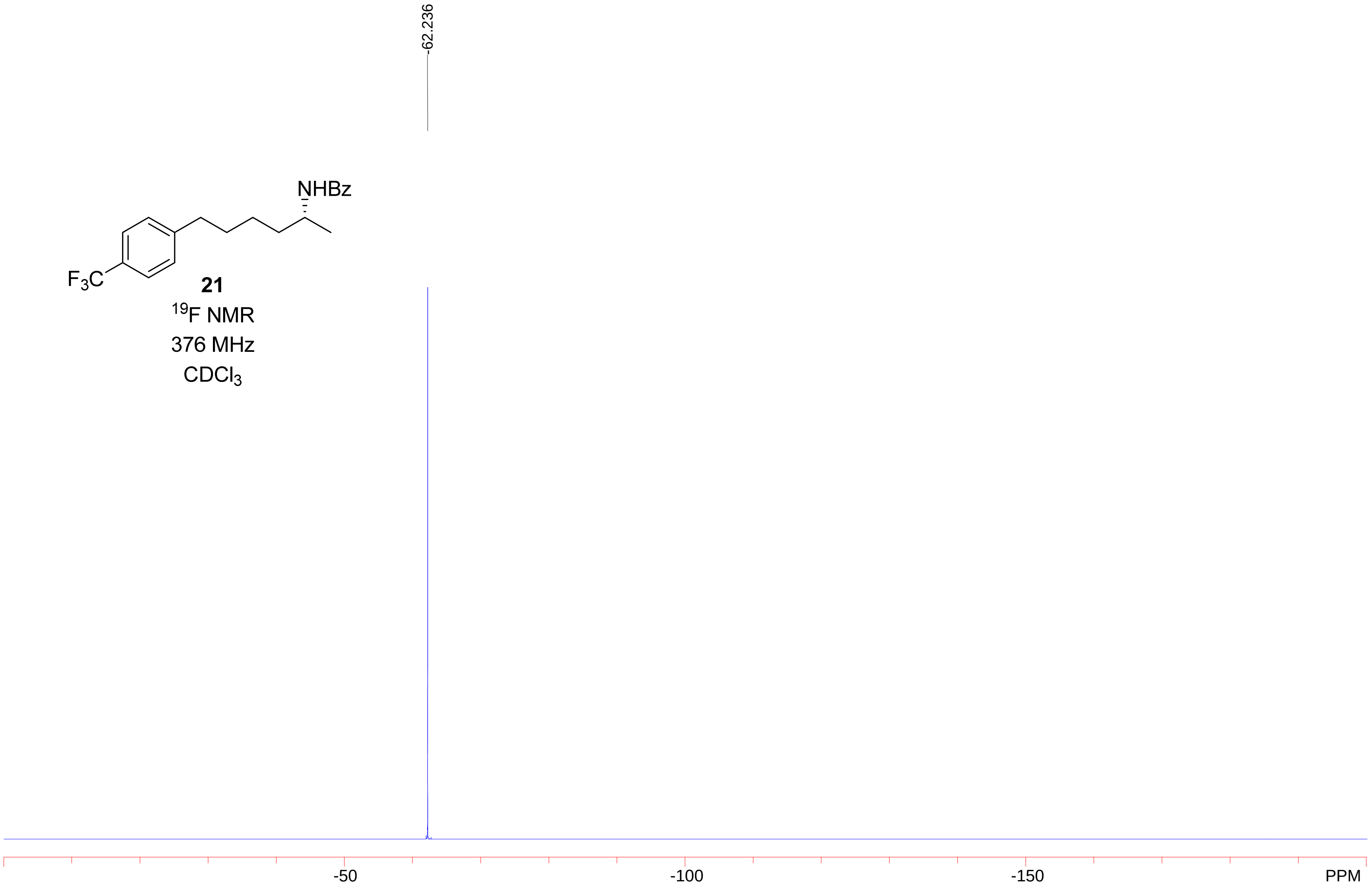

Supplementary Figure 150. ^{19}F NMR spectrum for **21**

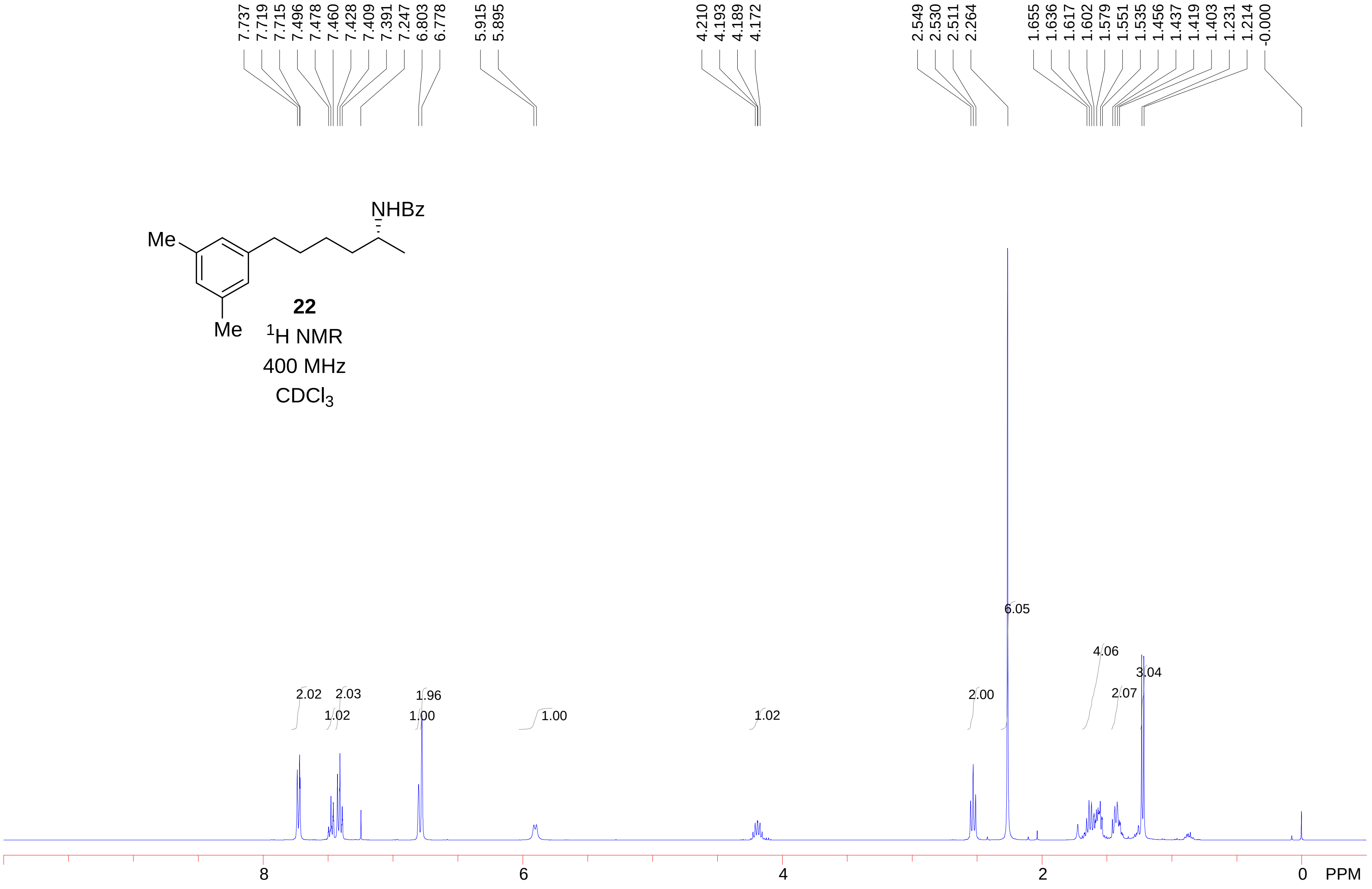

Supplementary Figure 151. ^{1}H NMR spectrum for **22**

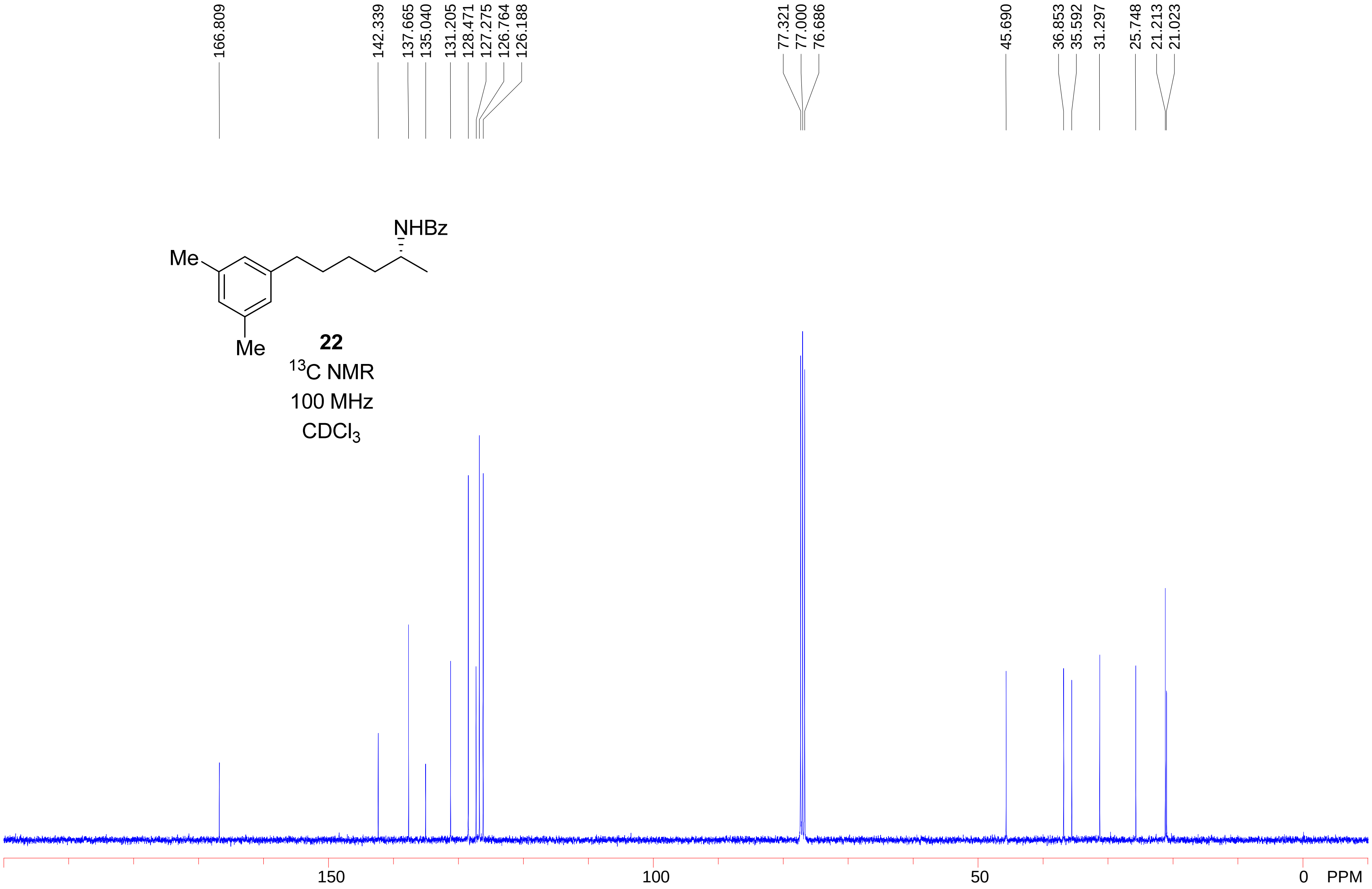

Supplementary Figure 152. ^{13}C NMR spectrum for **22**

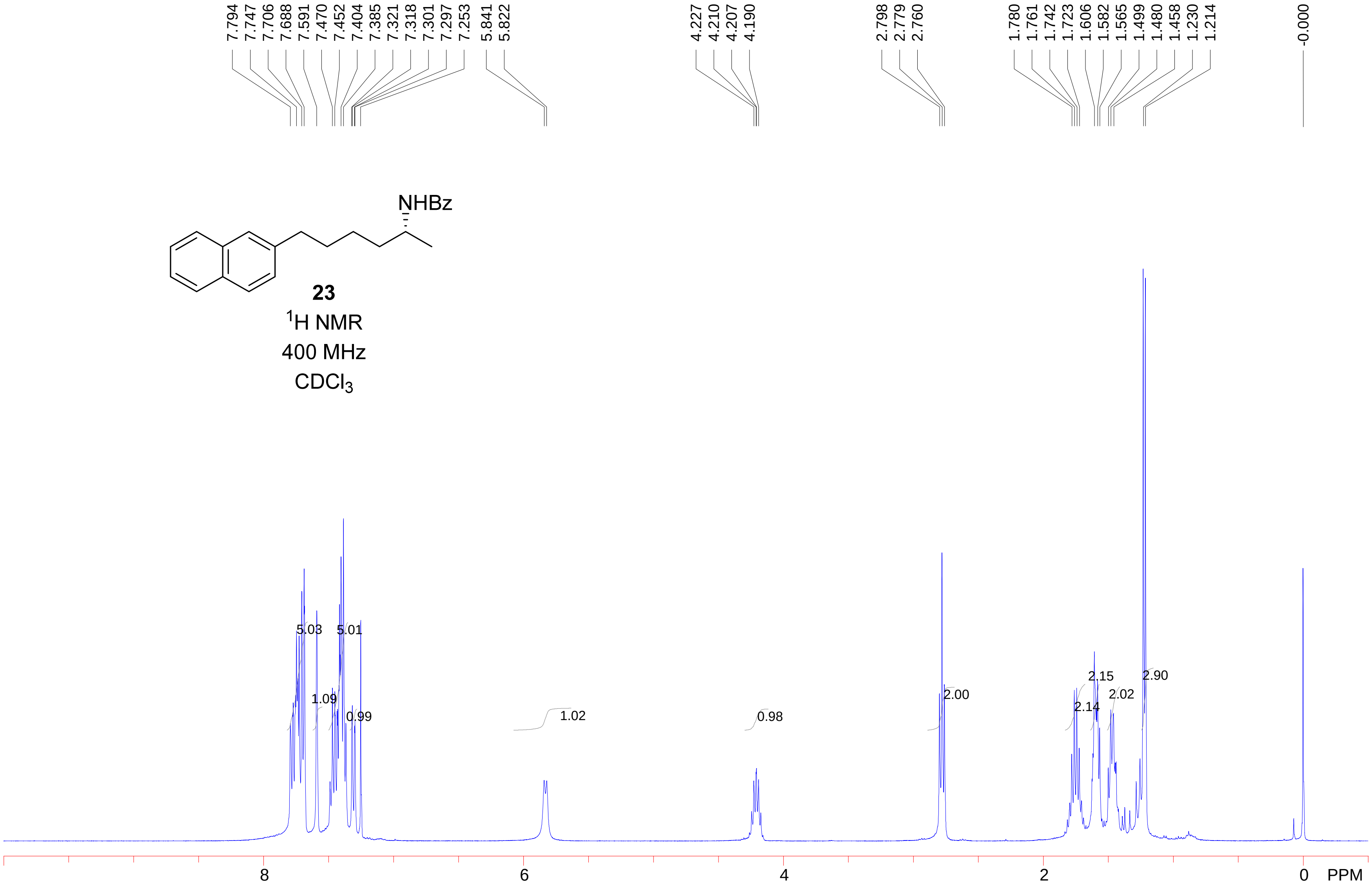

Supplementary Figure 153. ^{1}H NMR spectrum for **23**

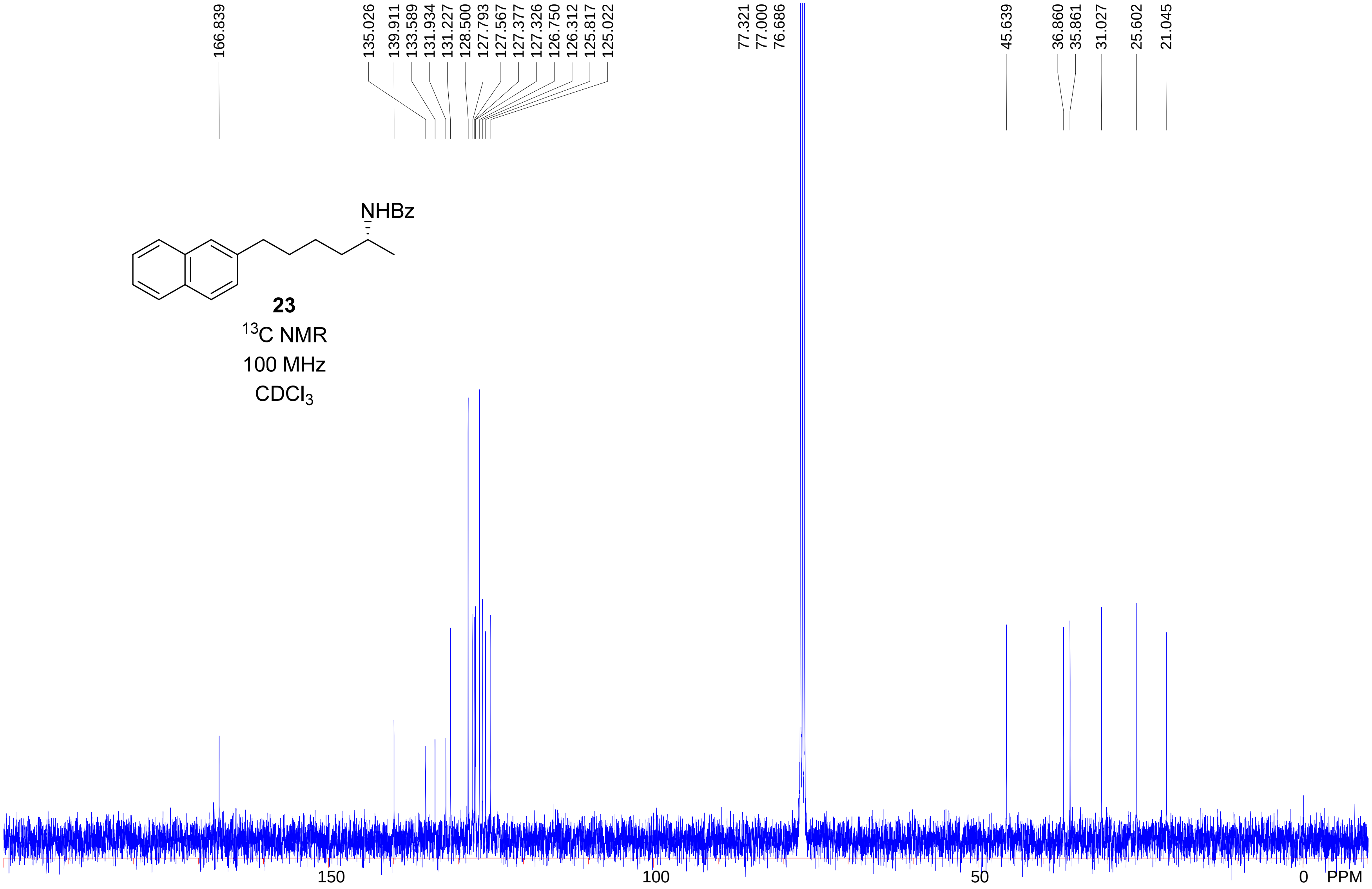

Supplementary Figure 154. ^{13}C NMR spectrum for **23**

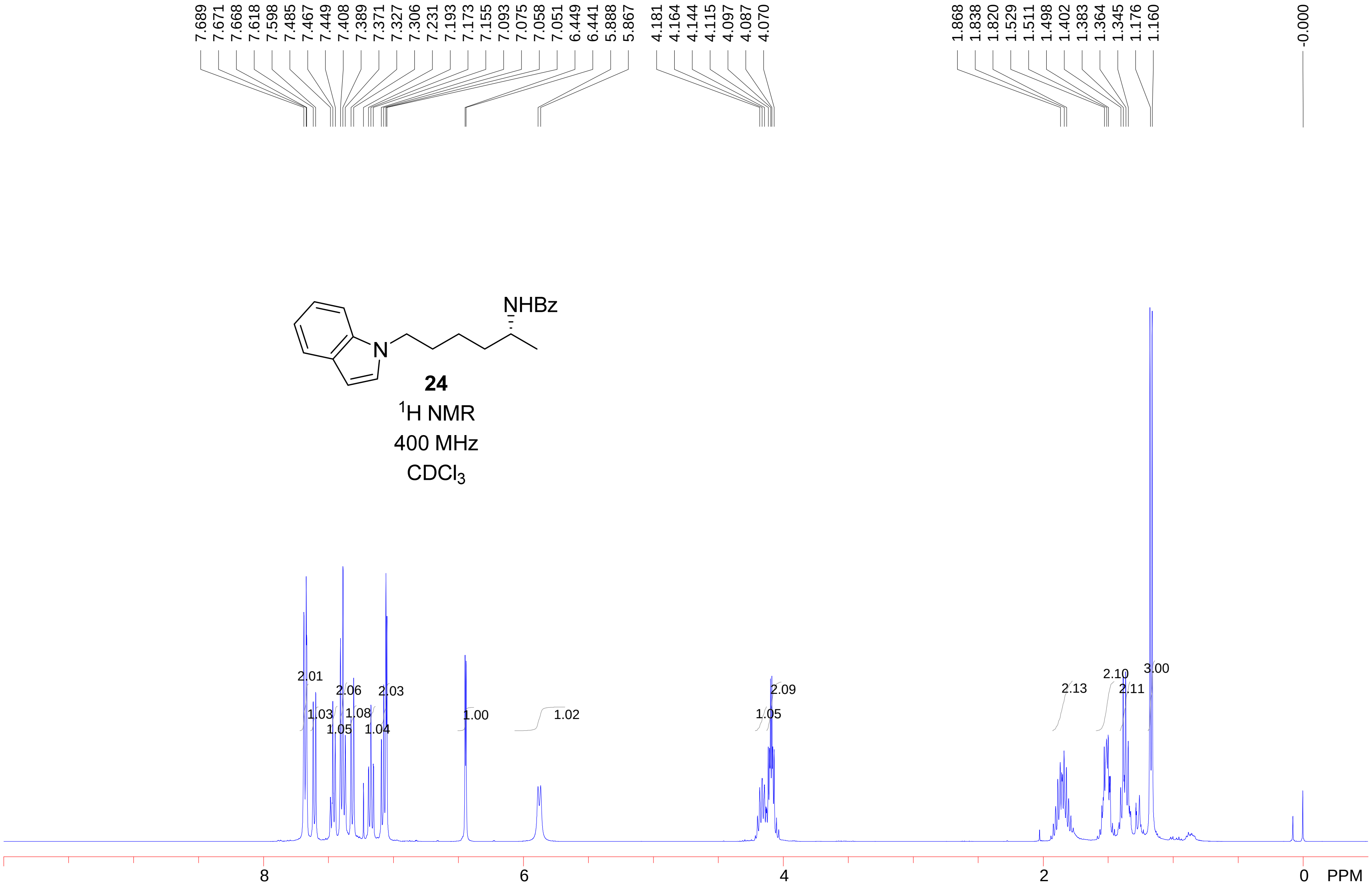

Supplementary Figure 155. ^{1}H NMR spectrum for **24**

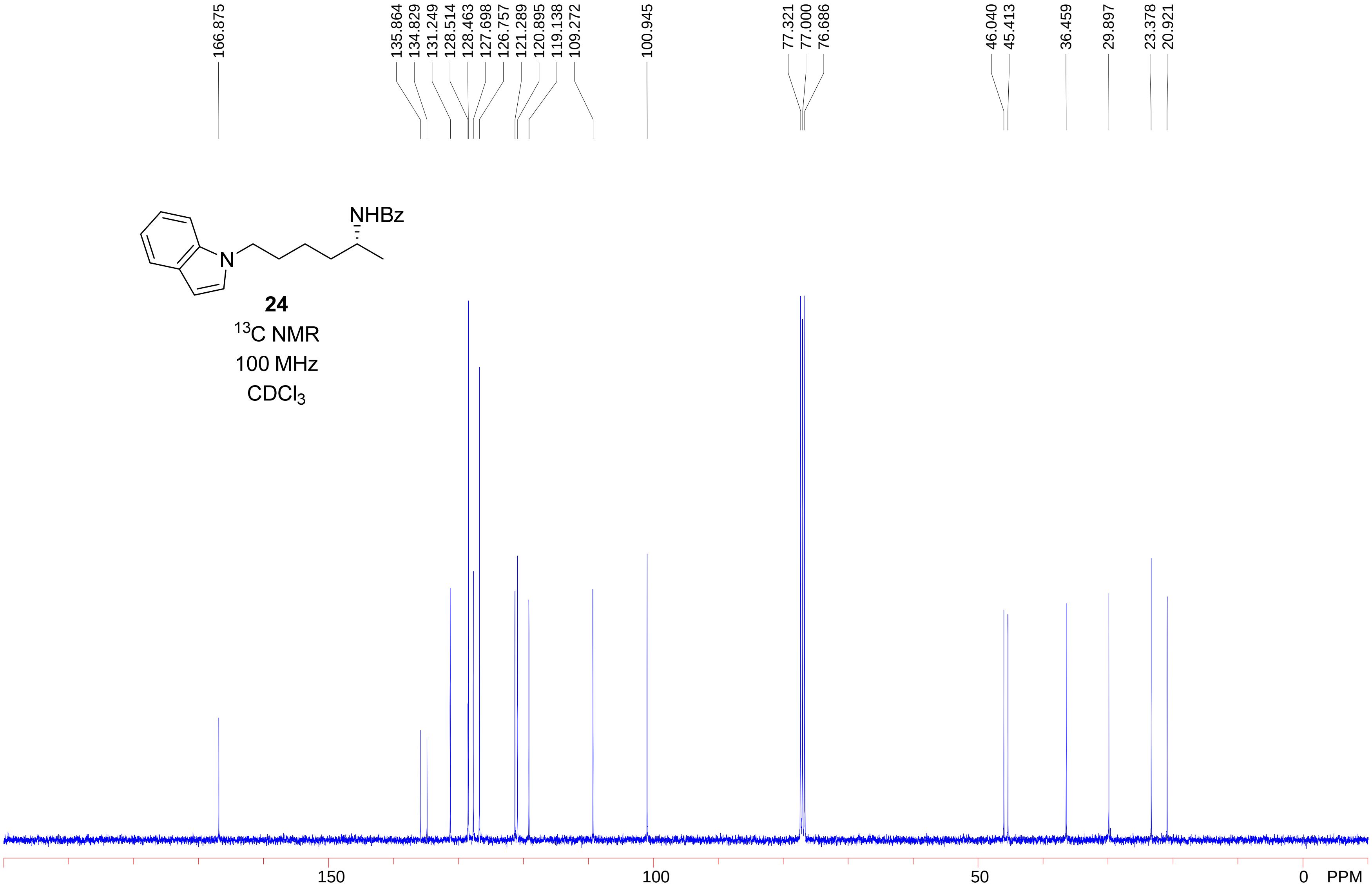

Supplementary Figure 156. ^{13}C NMR spectrum for **24**

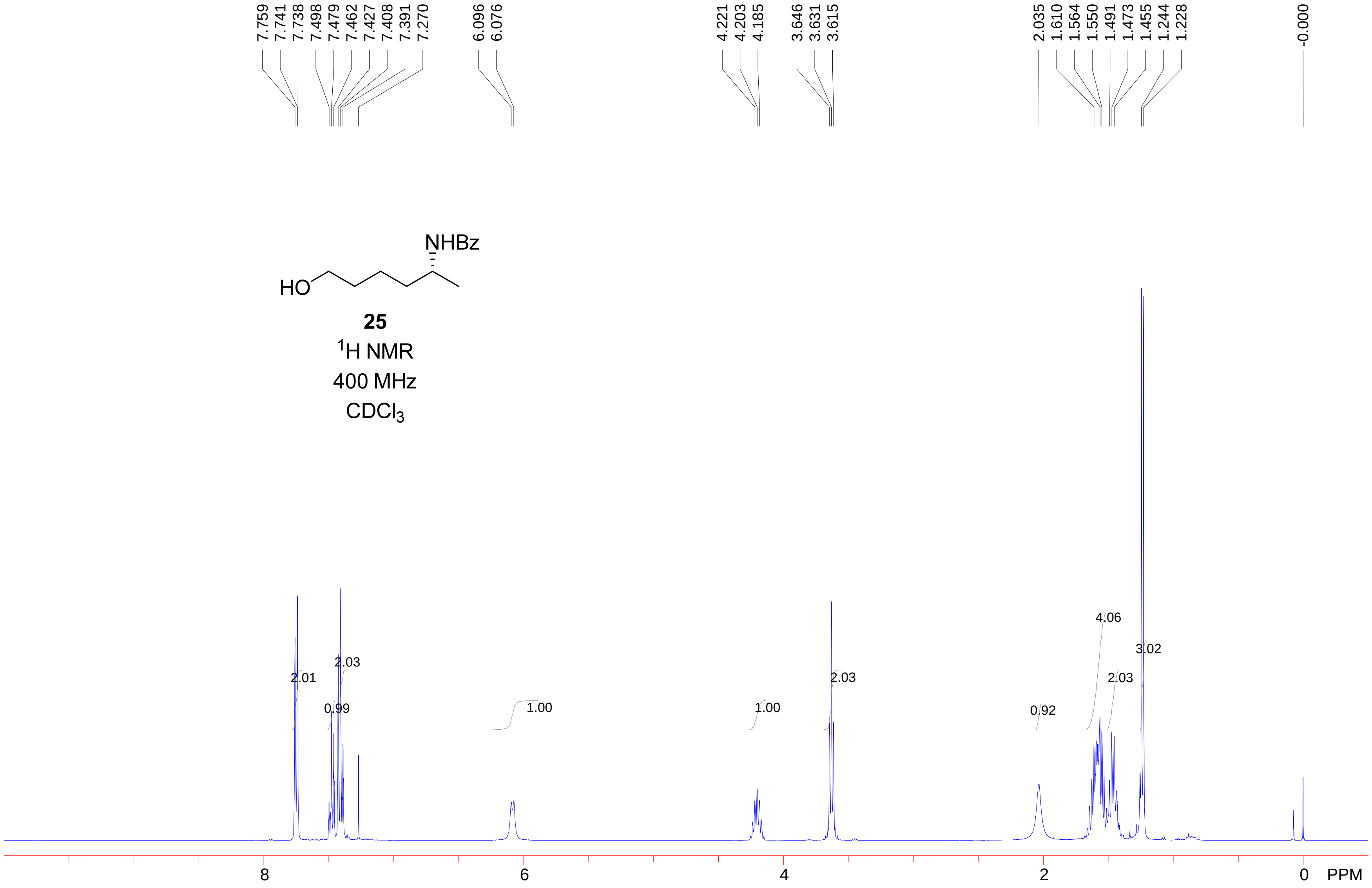

Supplementary Figure 157. ^{1}H NMR spectrum for **25**

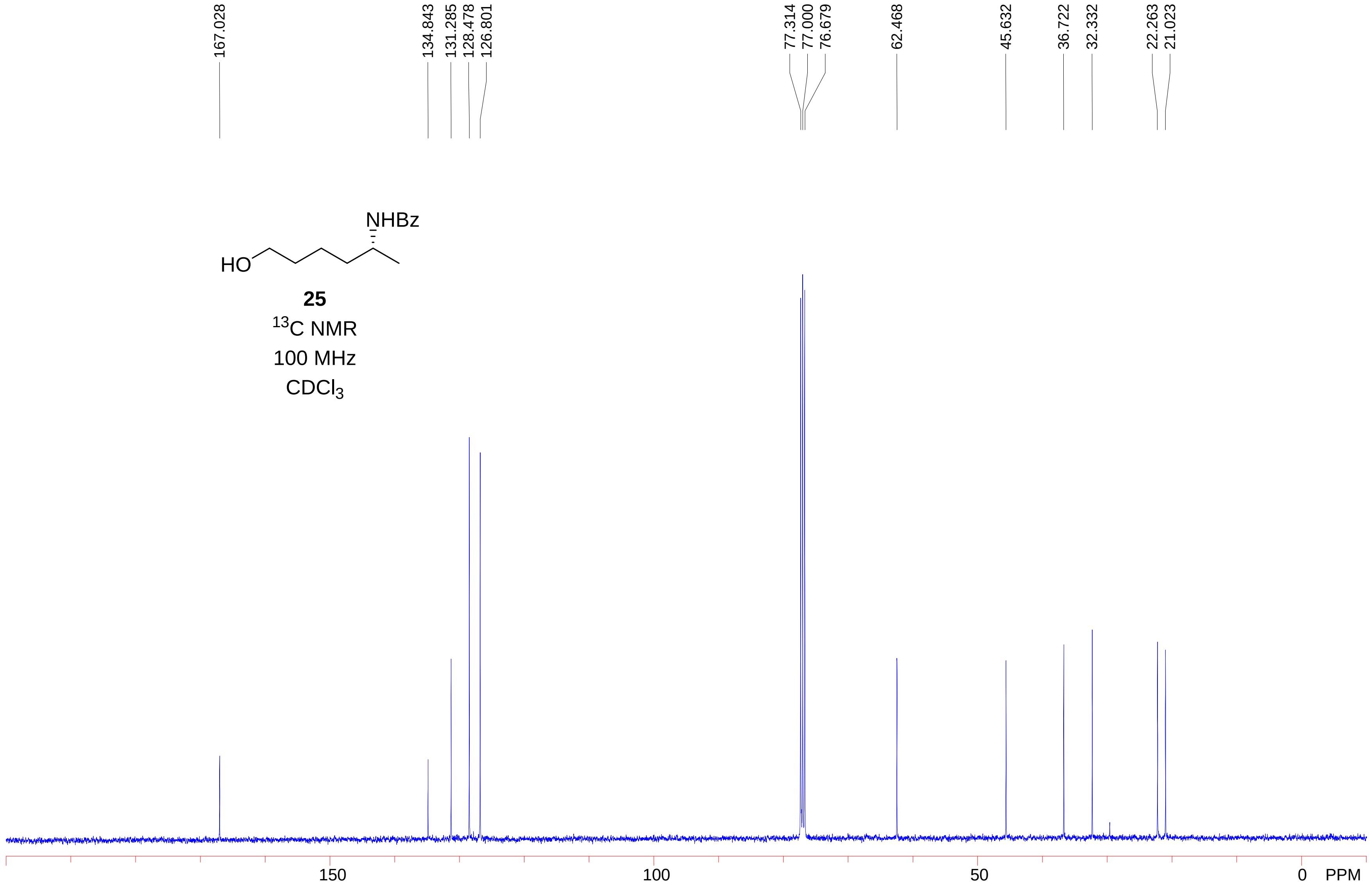

Supplementary Figure 158. ^{13}C NMR spectrum for **25**

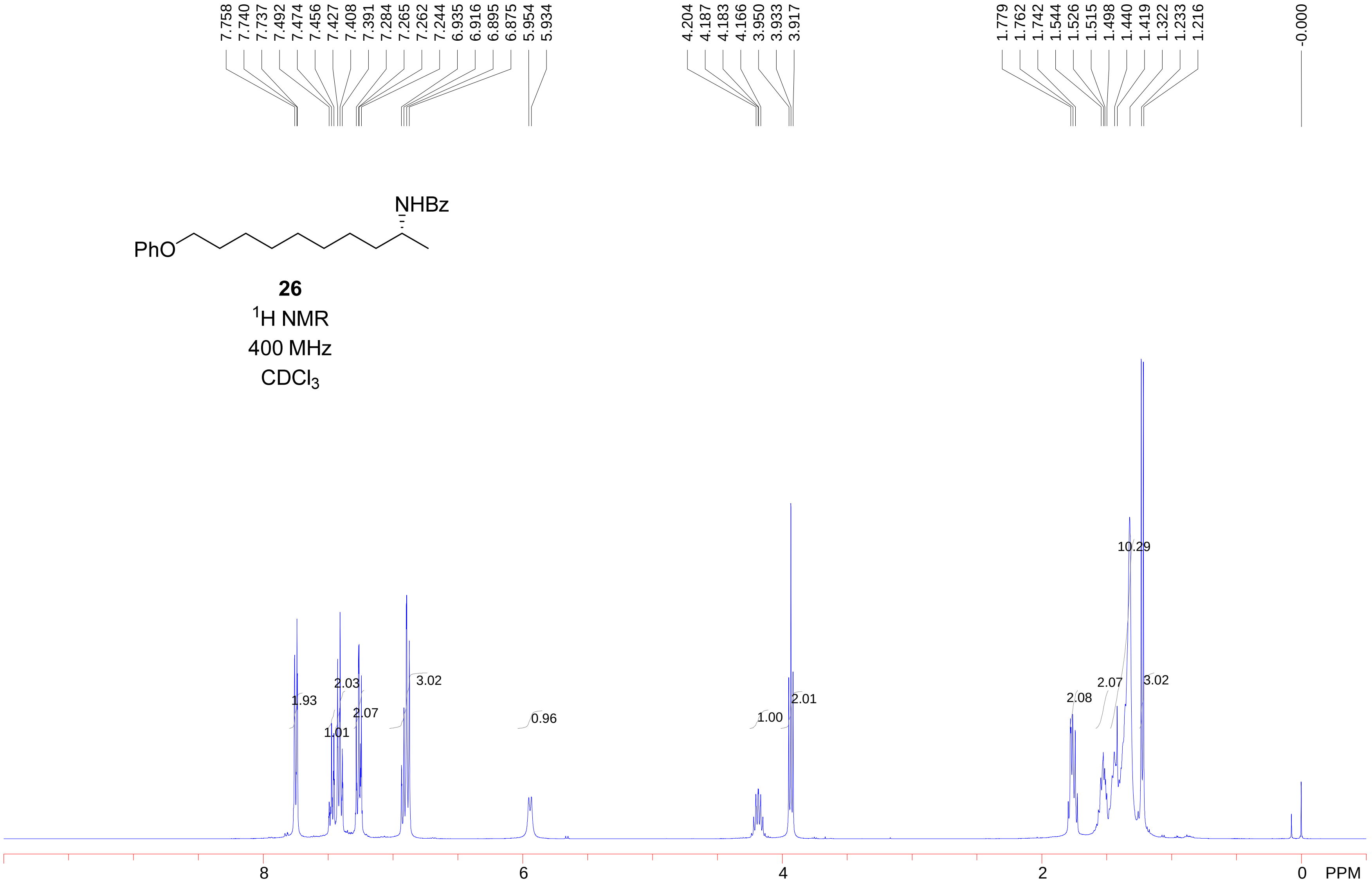

Supplementary Figure 159. ^{1}H NMR spectrum for **26**

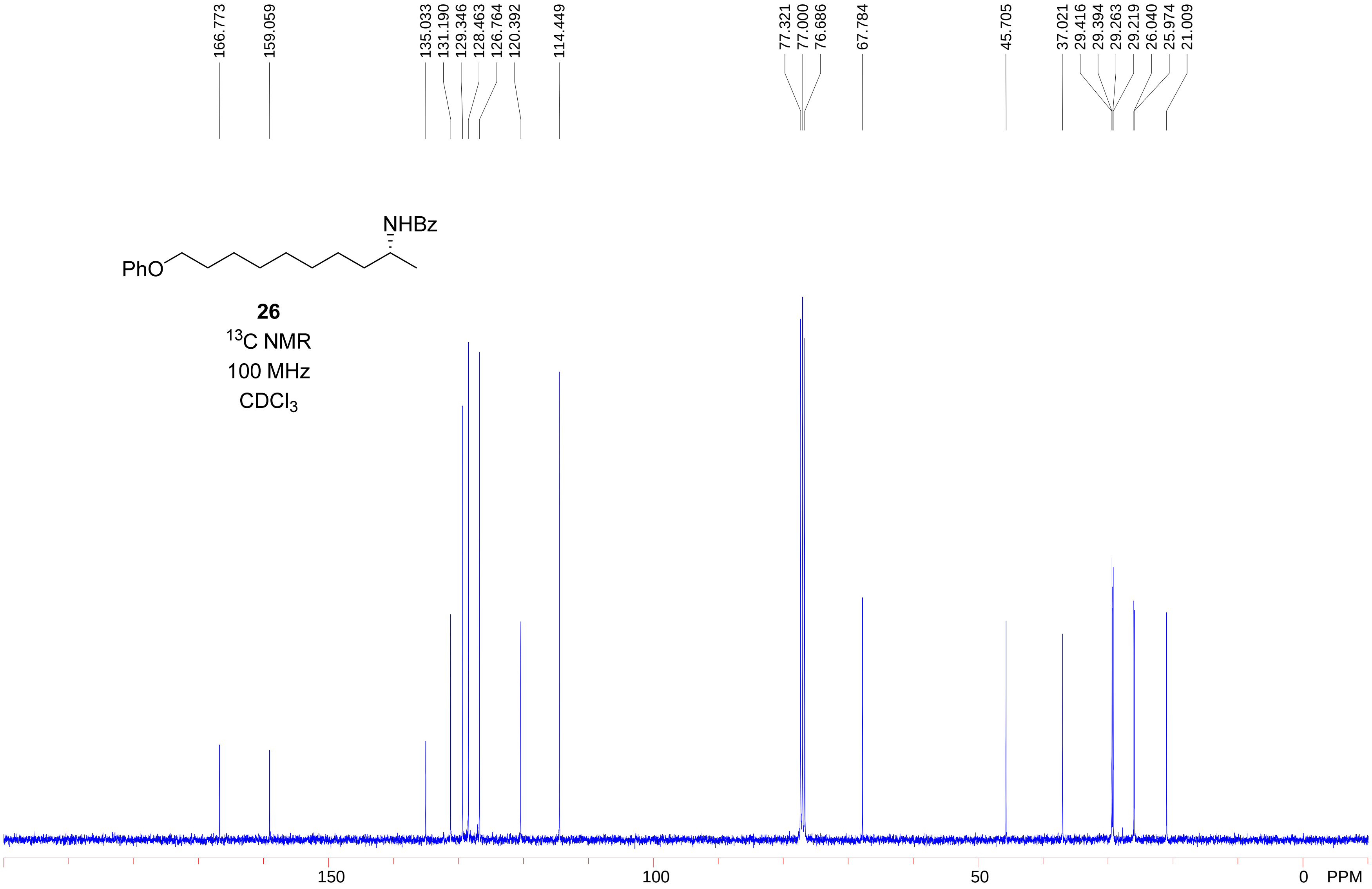

Supplementary Figure 160. ^{13}C NMR spectrum for **26**

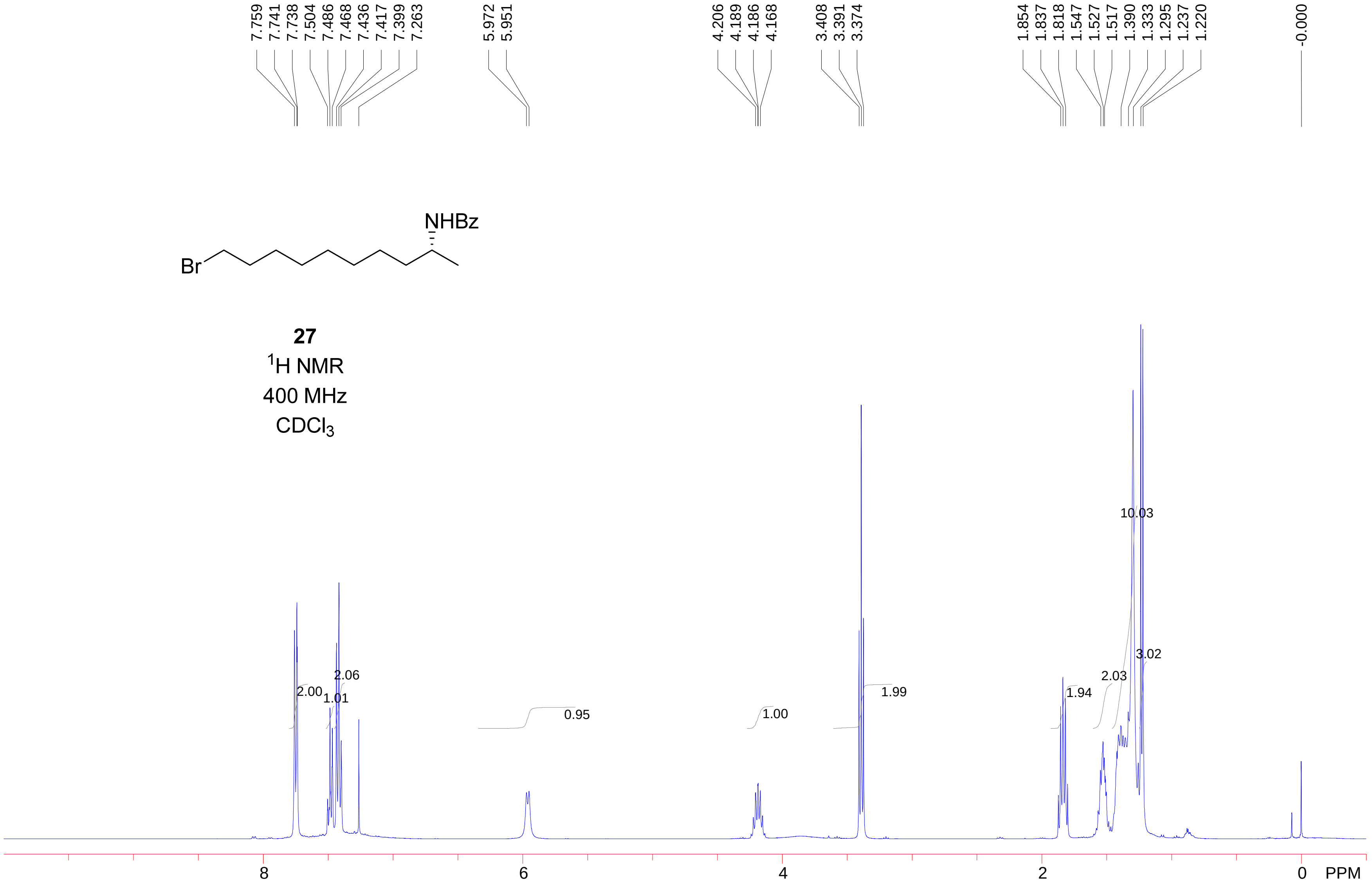

Supplementary Figure 161. ^{1}H NMR spectrum for **27**

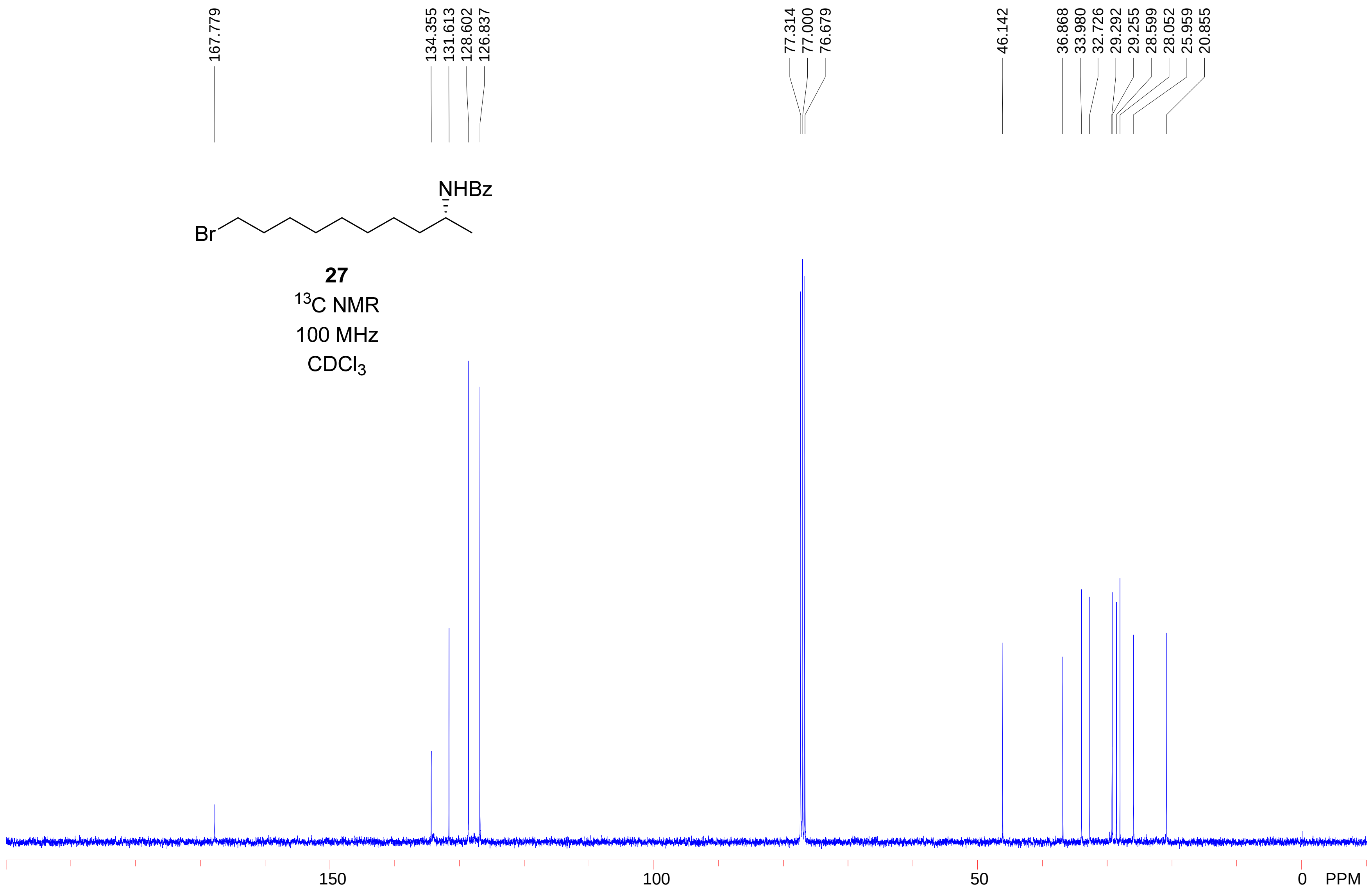

Supplementary Figure 162. ^{13}C NMR spectrum for **27**

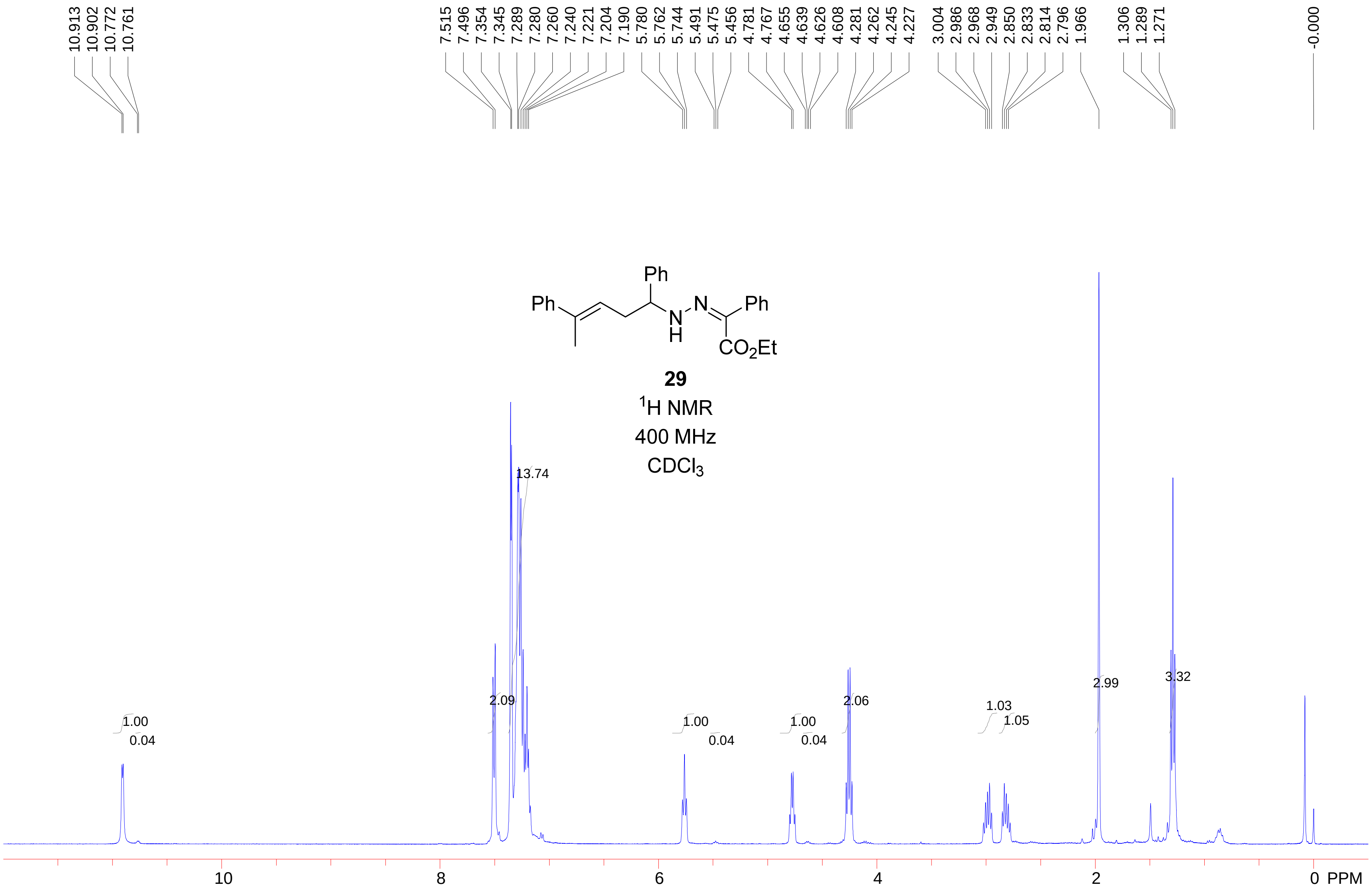

Supplementary Figure 163. ^{1}H NMR spectrum for **29**

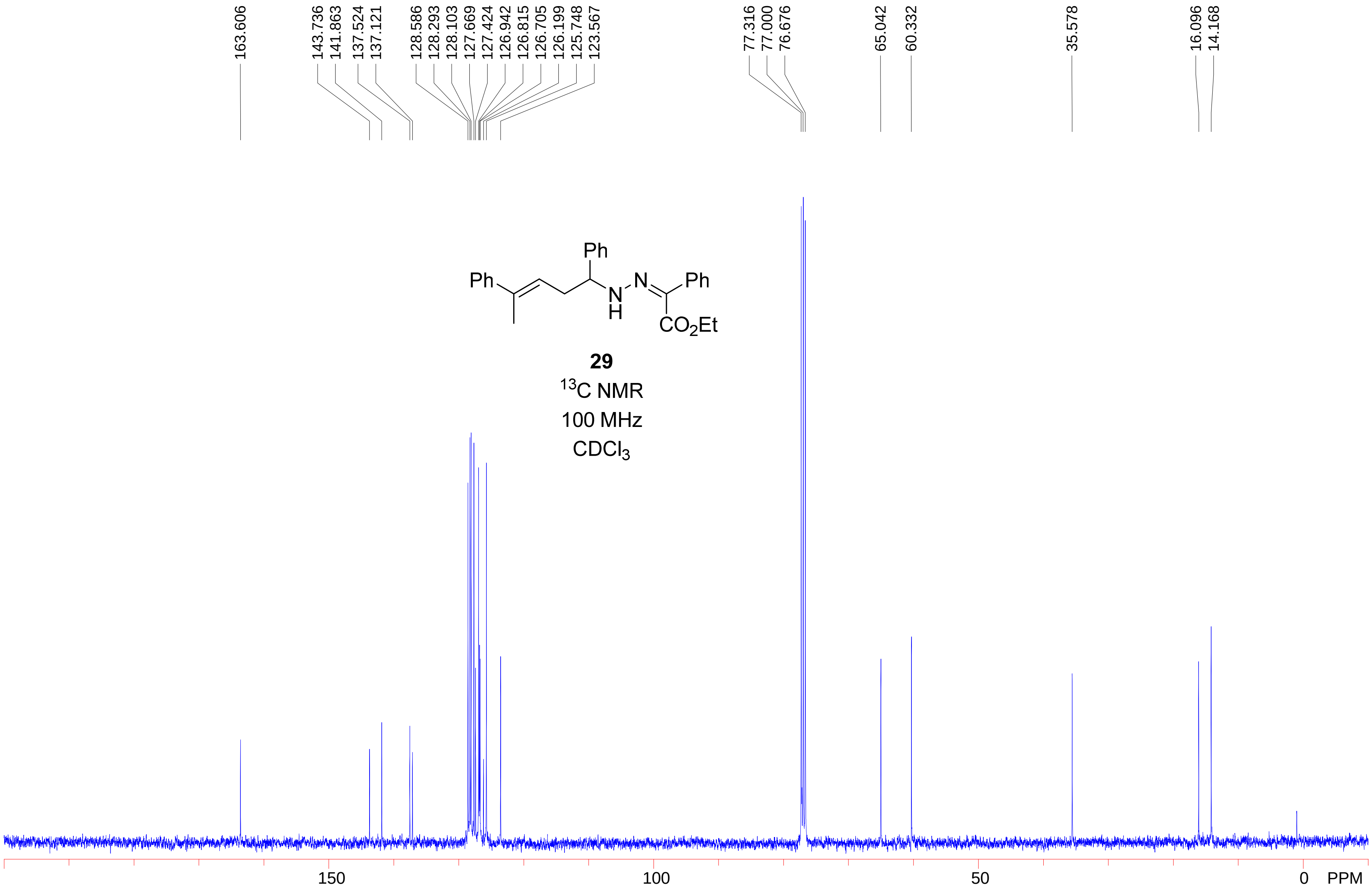

Supplementary Figure 164. ^{13}C NMR spectrum for **29**

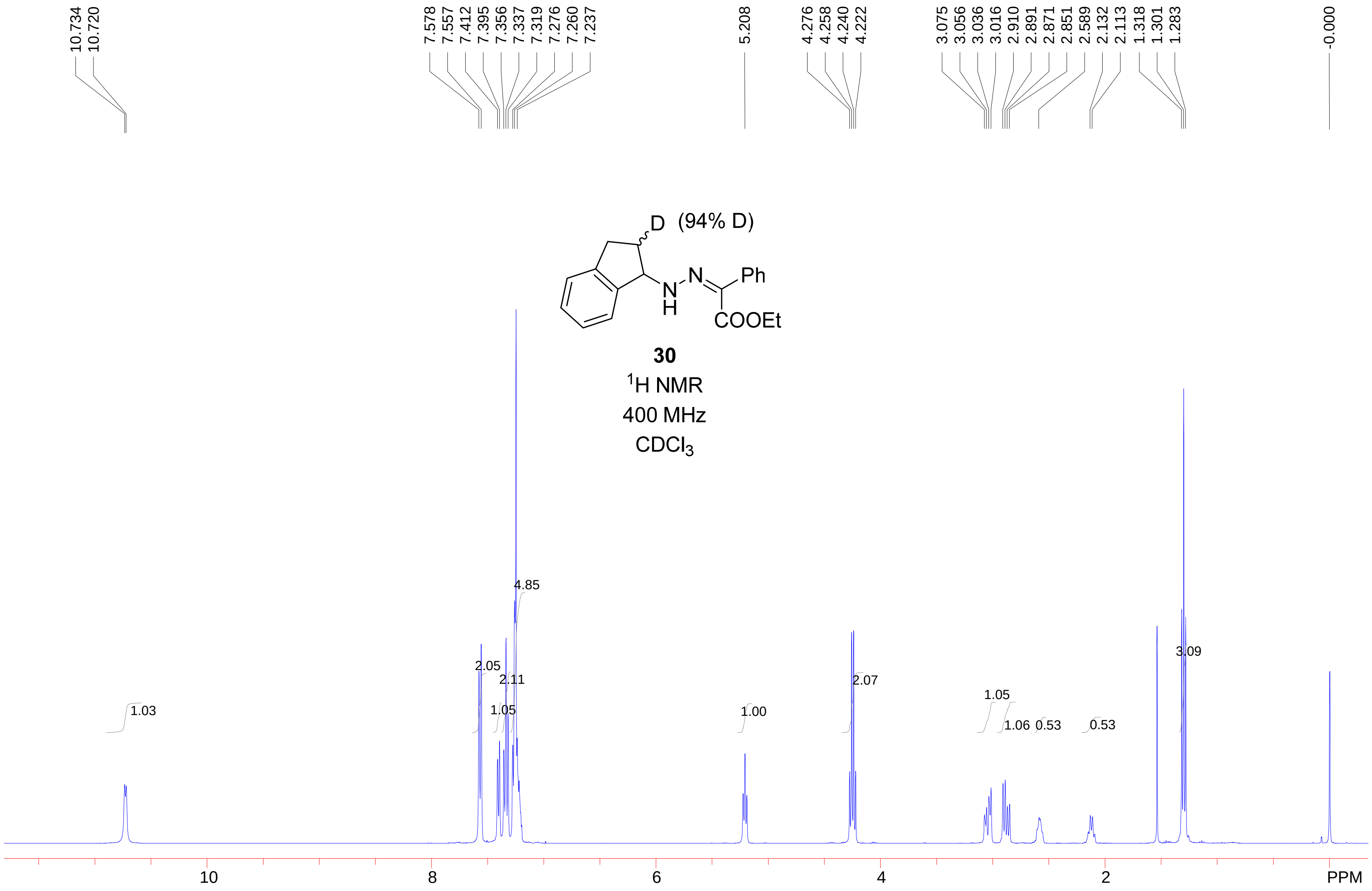

Supplementary Figure 165. ^{1}H NMR spectrum for **30**

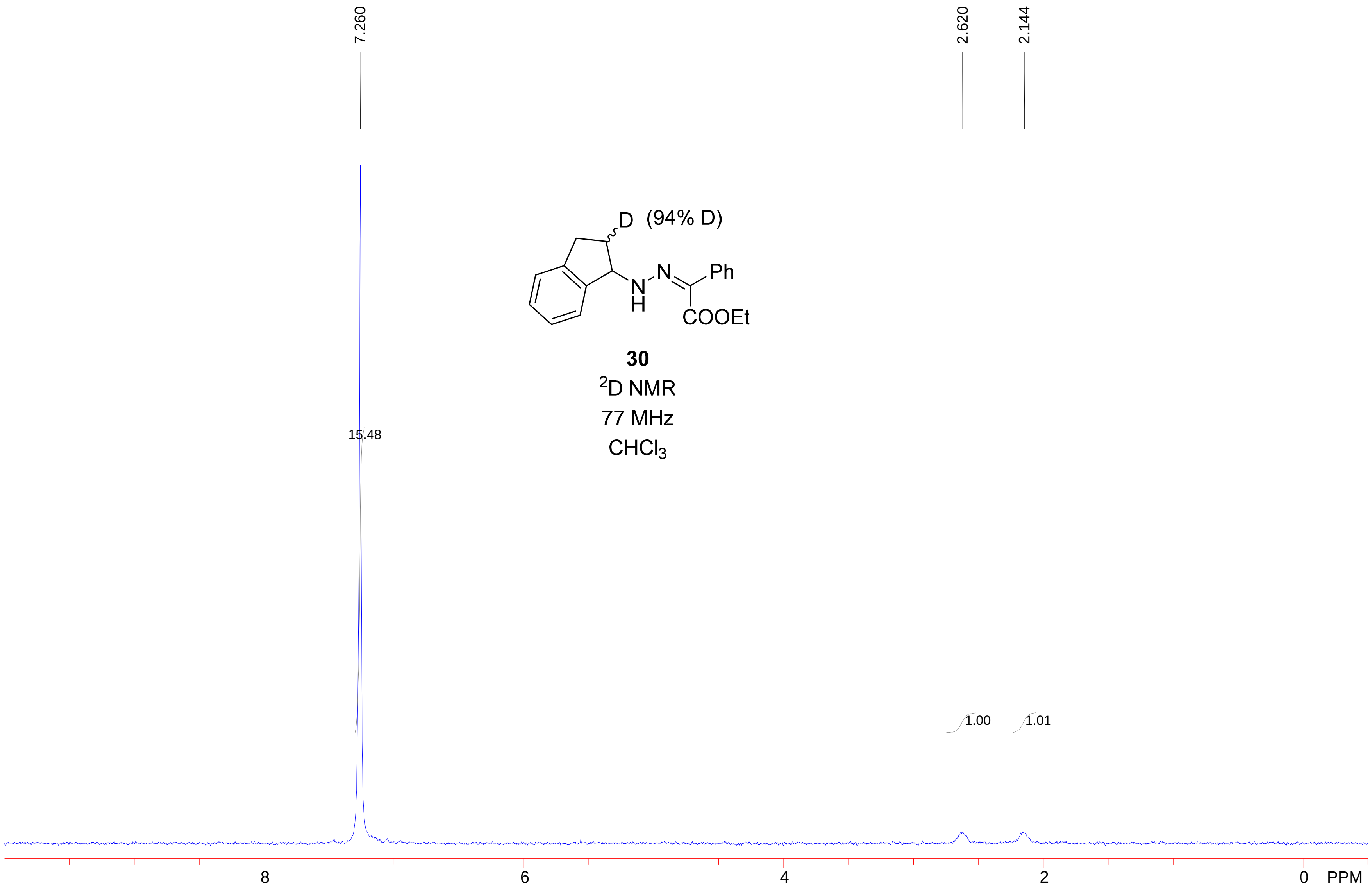

Supplementary Figure 166. ^{2}D NMR spectrum for **30**

HPLC Condition ： OD-H, n-hexane/iPrOH = 98/2, 1.0 ml/min, 254 nm

HPLC Spectra

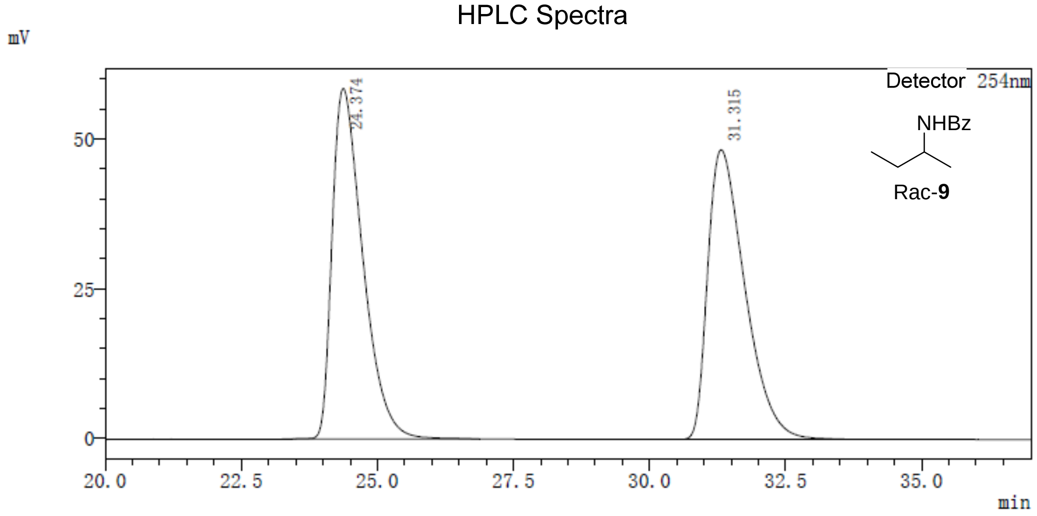

Area Percent Report

Detector 254nm

Number	Rremaining Time	Area	Height	Note	Area %
1	24.374	2301461	58575		50.023
2	31.315	2299348	48388		49.977
总计		4600809	106962		100.000

HPLC Condition OD-H, n-hexane/iPrOH = 98/2, 1.0 ml/min, 254 nm

HPLC Spectra

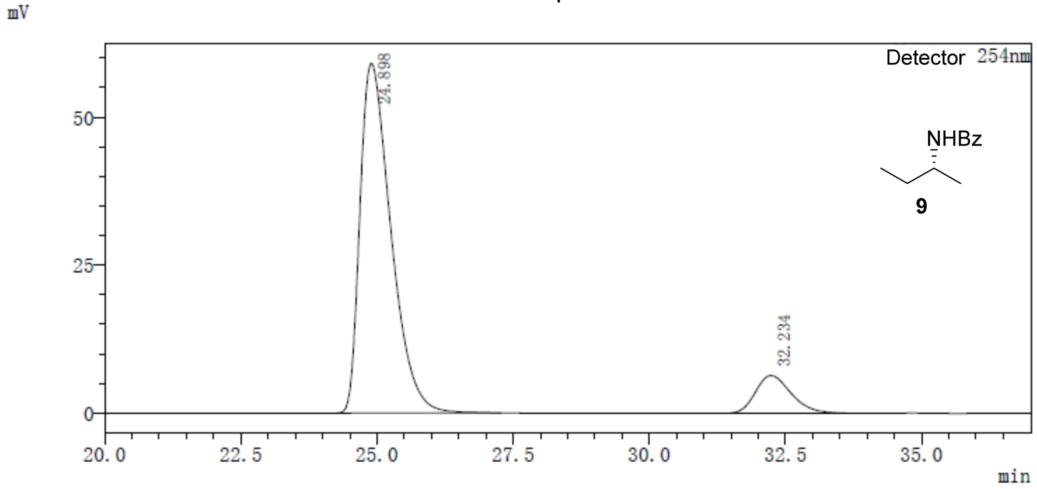

Area Percent Report

Detector 254nm

Number	Rremaining Time	Area	Height	Note	Area %
1	24.898	2333032	59100		88.990
2	32.234	288634	6373		11.010
Total		2621666	65474		100.000

HPLC Condition : OD-H, n-hexane/iPrOH = 98/2, 1.0 ml/min, 220 nm

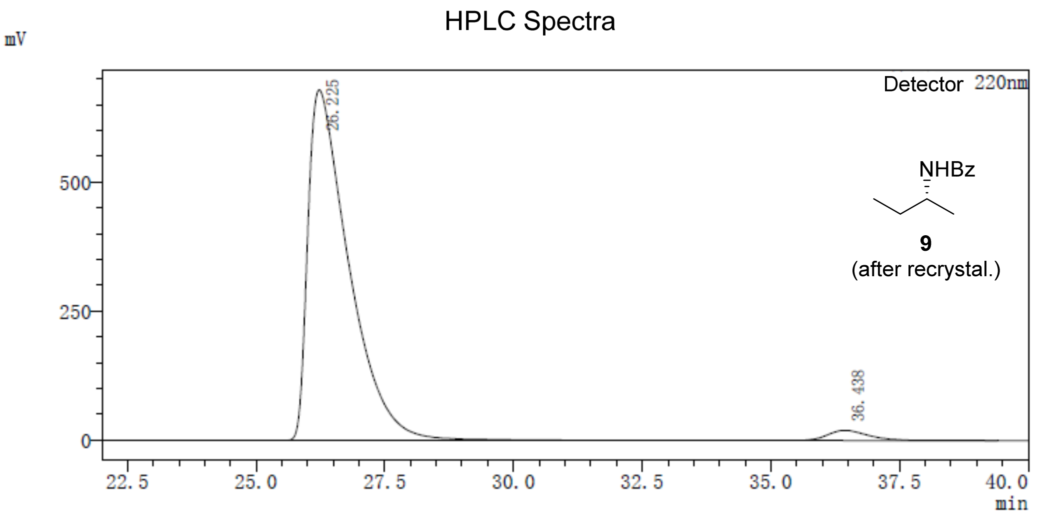

Area Percent Report

Detector 220nm

Number	Rremaining Time	Area	Height	Note	Area %
1	26.225	37651968	680054		97.250
2	36.438	1064842	19499		2.750
Total		38716810	699553		100.000

Supplementary Figure 167. HPLC spectrum for **9**

HPLC Condition : OD-H, n-hexane/iPrOH = 98/2, 1.0 ml/min, 254 nm

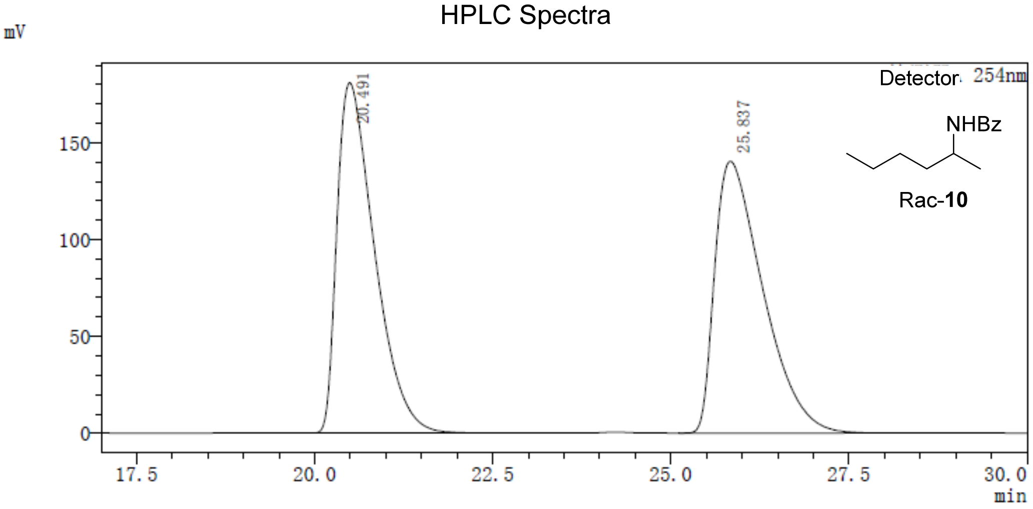

Area Percent Report

Detector 254nm

Number	Rremaining Time	Area	Height	Note	Area %
1	20. 491	6633338	180849		50. 073
2	25. 837	6614085	140264		49. 927
Total		13247423	321113		100. 000

HPLC Condition : OD-H, n-hexane/iPrOH = 98/2, 1.0 ml/min, 254 nm

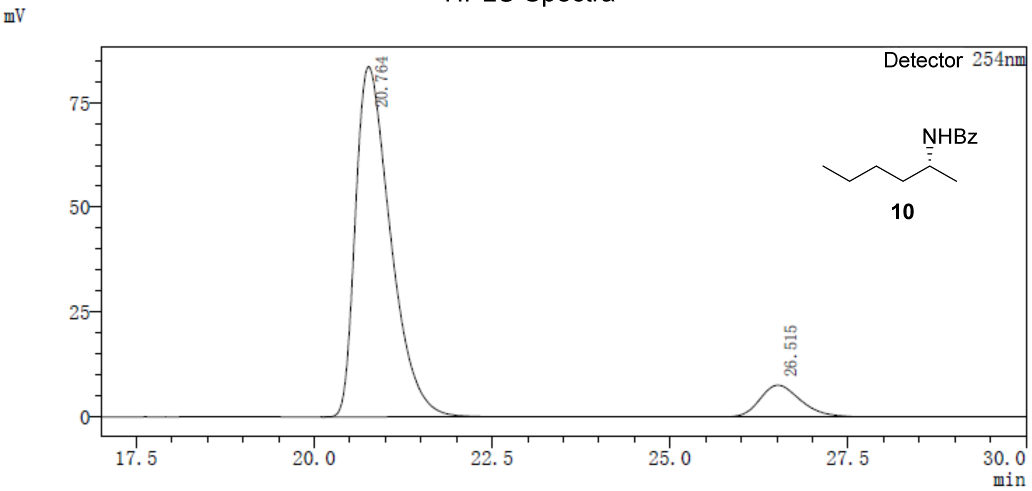

Area Percent Report

Detector 254nm

Number	Rremaining Time	Area	Height	Note	Area %
1	20. 764	2857396	83514		90. 531
2	26. 515	298871	7535		9. 469
Total		3156267	91049		100. 000

Supplementary Figure 168. HPLC spectrum for **10**

HPLC Condition : OJ-H, n-hexane/iPrOH = 99/1, 1.0 ml/min, 254 nm

HPLC Spectra

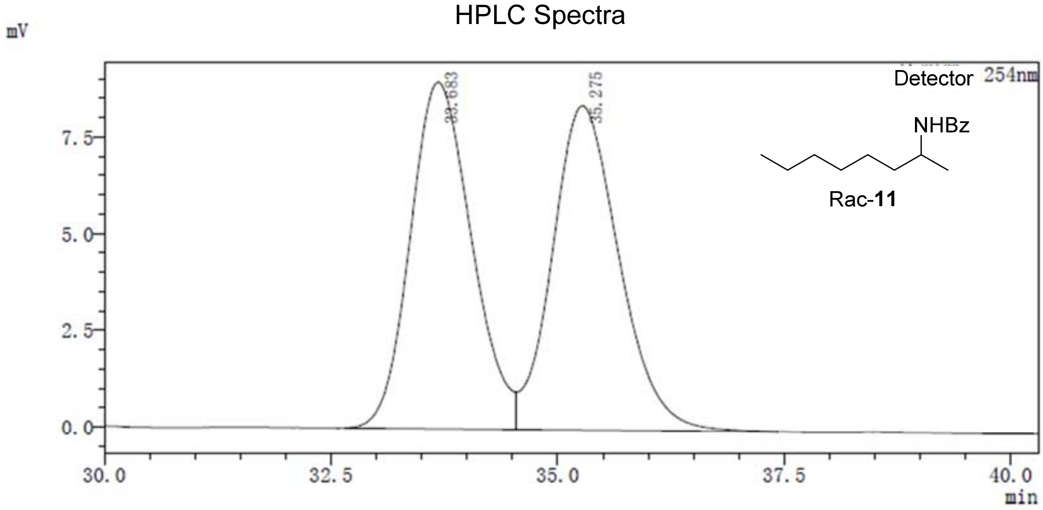

Area Percent Report

Detector 254nm

Number	Rremaining Time	Area	Height	Note	Area %
1	33.683	421086	8953		49.490
2	35.275	429769	8379	V	50.510
Total		850855	17332		100.000

HPLC Condition OD-H, n-hexane/iPrOH = 99/1, 1.0 ml/min, 254 nm

HPLC Spectra

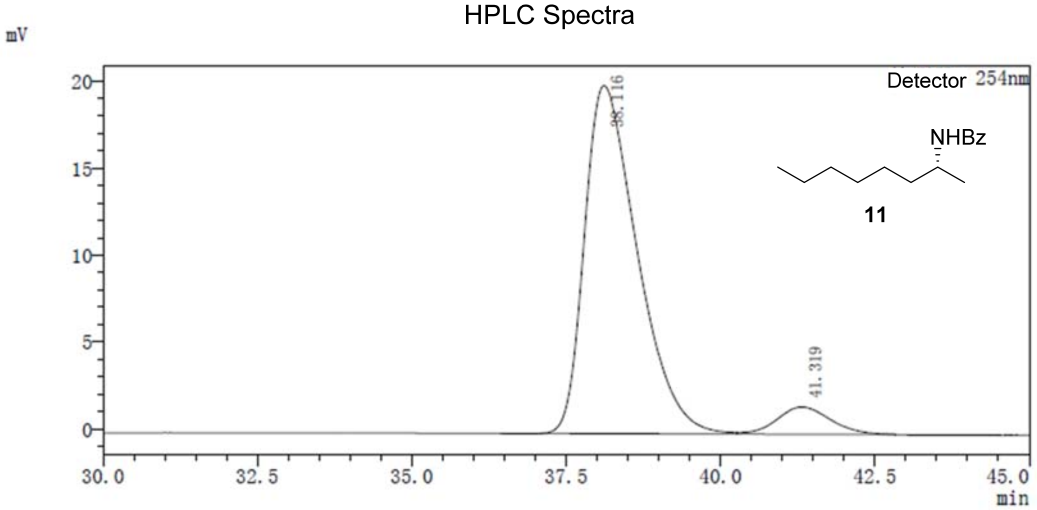

Area Percent Report

Detector 254nm

Number	Rremaining Time	Area	Height	Note	Area %
1	38.116	1188043	19992		92.655
2	41.319	94177	1564	V	7.345
Total		1282220	21556		100.000

HPLC Condition : OJ-H, n-hexane/iPrOH = 99/1, 1.0 ml/min, 254 nm

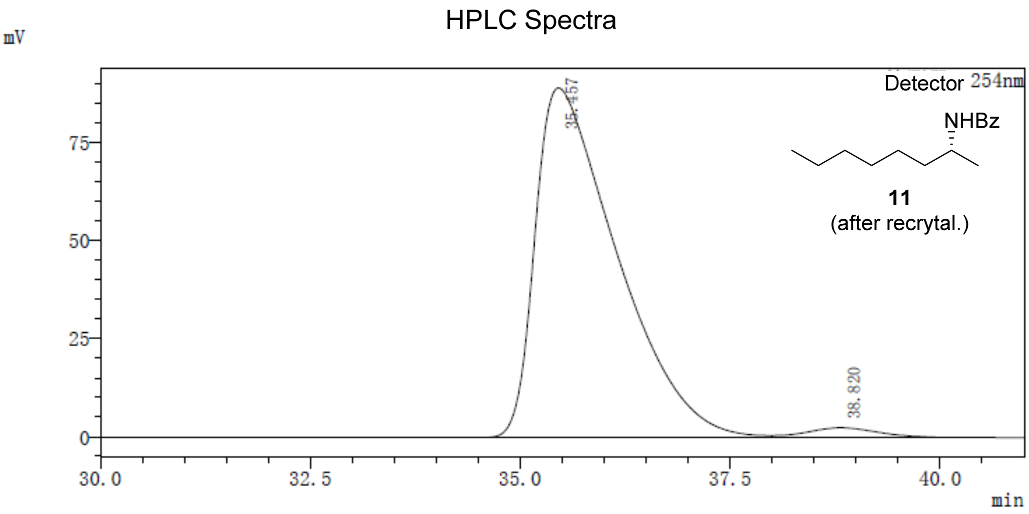

Area Percent Report

Detector 254nm

Number	Rremaining Time	Area	Height	Note	Area %
1	35.457	6039284	89149		97.610
2	38.820	147900	2481	V	2.390
Total		6187183	91630		100.000

Supplementary Figure 169. HPLC spectrum for **11**

HPLC Condition : OD-H, n-hexane/iPrOH = 98/2, 1.0 ml/min, 254 nm

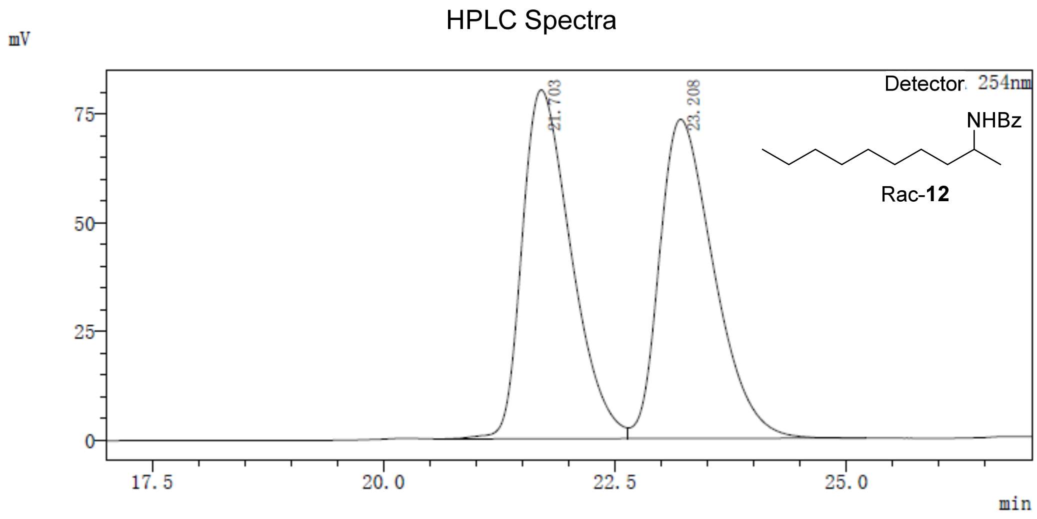

Area Percent Report

Detector 254nm

Number	Rremaining Time	Area	Height	Note	Area %
1	21. 703	2957891	80223		49. 692
2	23. 208	2994587	73404	V	50. 308
Total		5952478	153627		100. 000

HPLC Condition : OD-H, n-hexane/iPrOH = 98/2, 1.0 ml/min, 254 nm

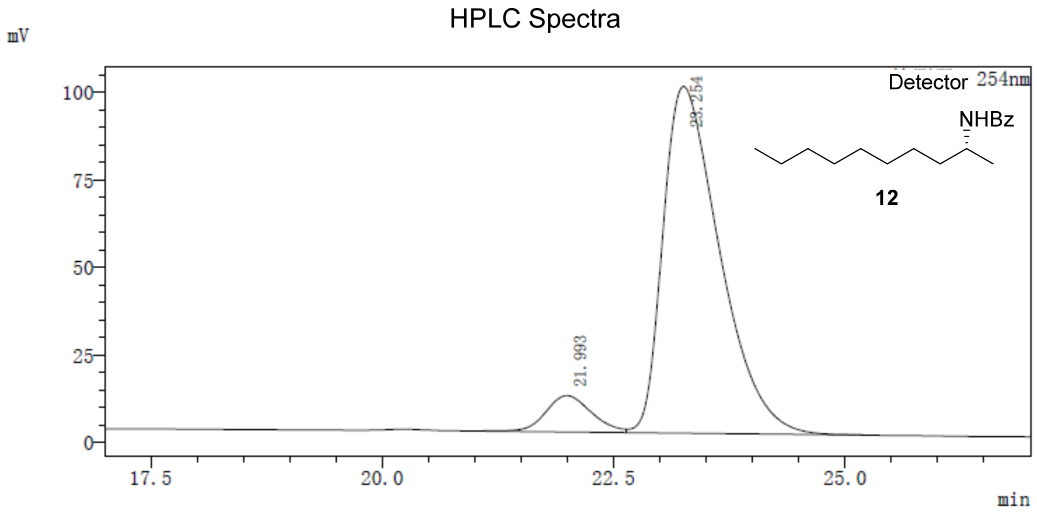

Area Percent Report

Detector 254nm

Number	Rremaining Time	Area	Height	Note	Area %
1	21. 993	359357	10393		7. 839
2	23. 254	4224754	99101	V	92. 161
Total		4584111	109495		100. 000

Supplementary Figure 170. HPLC spectrum for **12**

HPLC Condition : OJ-H, n-hexane/iPrOH = 99/1, 1.0 ml/min, 220 nm

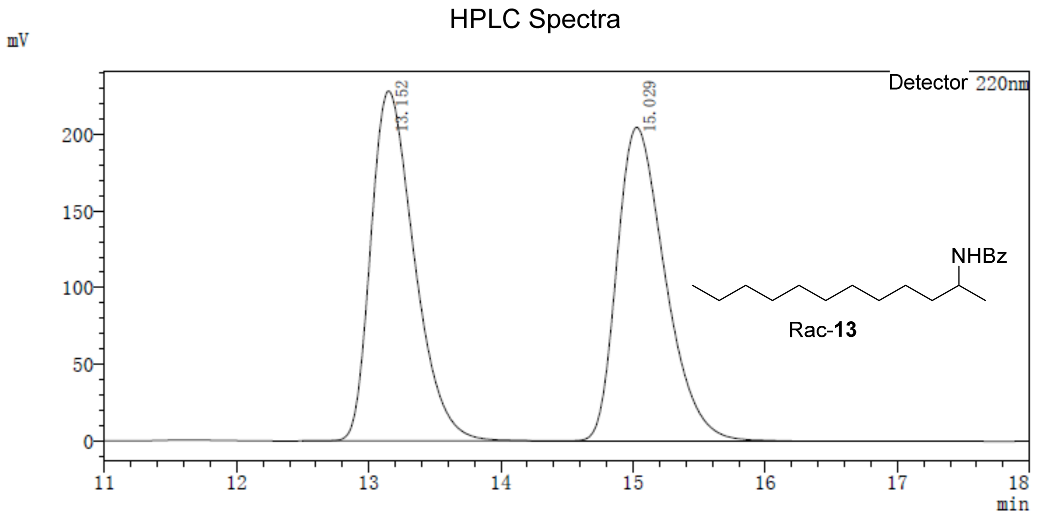

Area Percent Report

Detector 220nm

Number	Rremaining Time	Area	Height	Note	Area %
1	13.152	5230182	228387		50.067
2	15.029	5216172	204841	V	49.933
Total		10446354	433228		100.000

HPLC Condition : OJ-H, n-hexane/iPrOH = 99/1, 1.0 ml/min, 220 nm

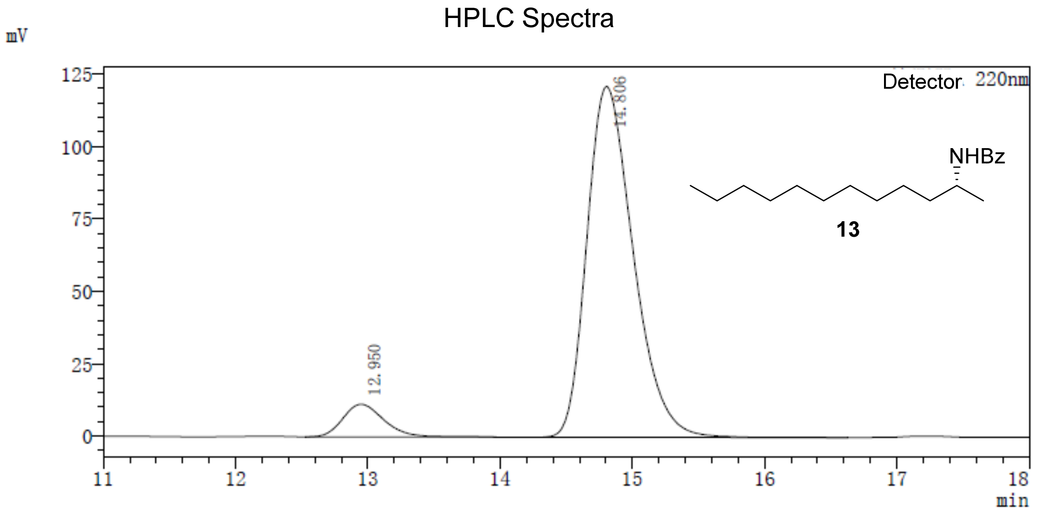

Area Percent Report

Detector 220nm

Number	Rremaining Time	Area	Height	Note	Area %
1	12.950	246888	11291	S	7.704
2	14.806	2957667	121139		92.296
Total		3204555	132430		100.000

Supplementary Figure 171. HPLC spectrum for **13**

HPLC Condition : OD-H, n-hexane/iPrOH = 85/15, 1.0 ml/min, 254 nm

HPLC Spectra

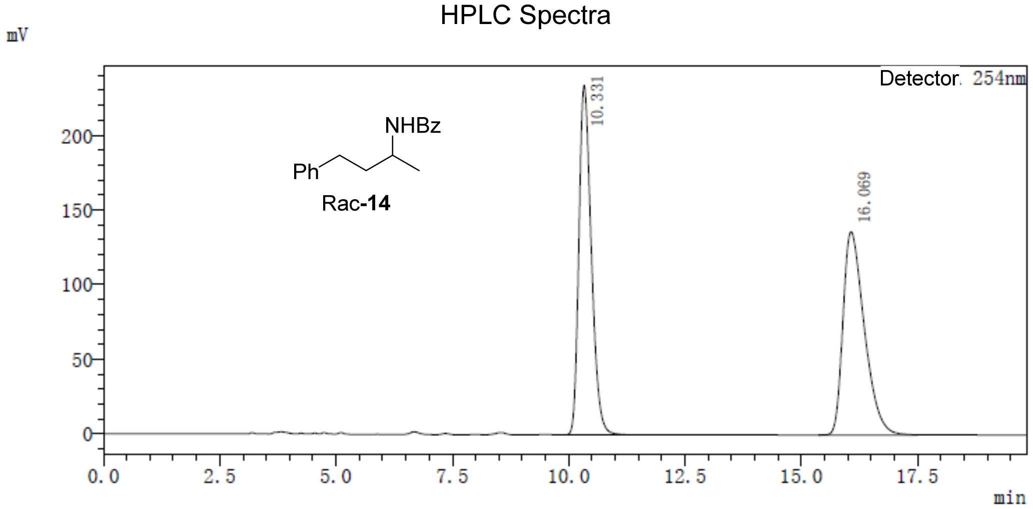

Area Percent Report

Detector 254nm

Number	Rremaining Time	Area	Height	Note	Area %
1	10. 331	4344958	233741		49. 861
2	16. 069	4369207	136133		50. 139
Total		8714165	369874		100. 000

HPLC Condition : OD-H, n-hexane/iPrOH = 85/15, 1.0 ml/min, 254 nm

HPLC Spectra

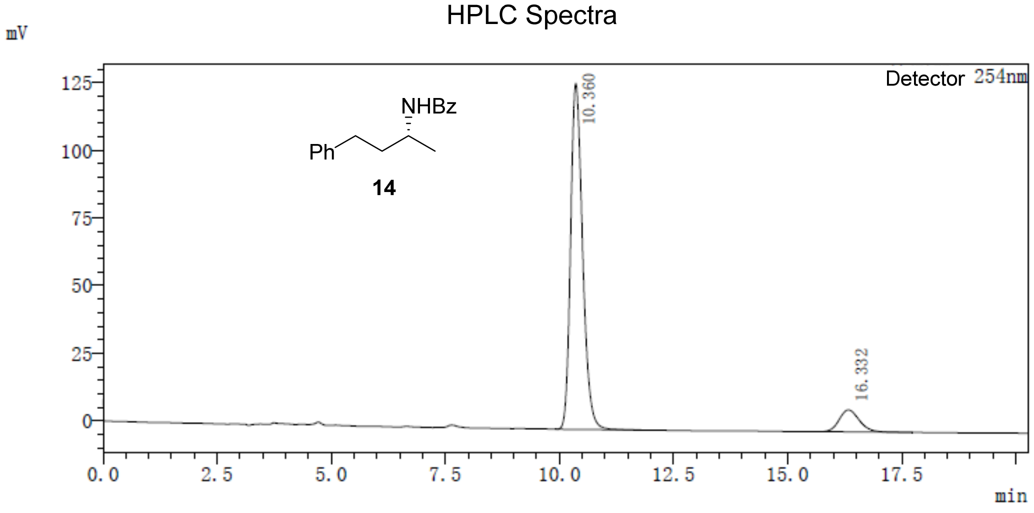

Area Percent Report

Detector 254nm

Number	Rremaining Time	Area	Height	Note	Area %
1	10. 360	2336010	127729		90. 339
2	16. 332	249826	8130		9. 661
Total		2585836	135859		100. 000

HPLC Condition : OD-H, n-hexane/iPrOH = 85/15, 1.0 ml/min, 254 nm

HPLC Spectra

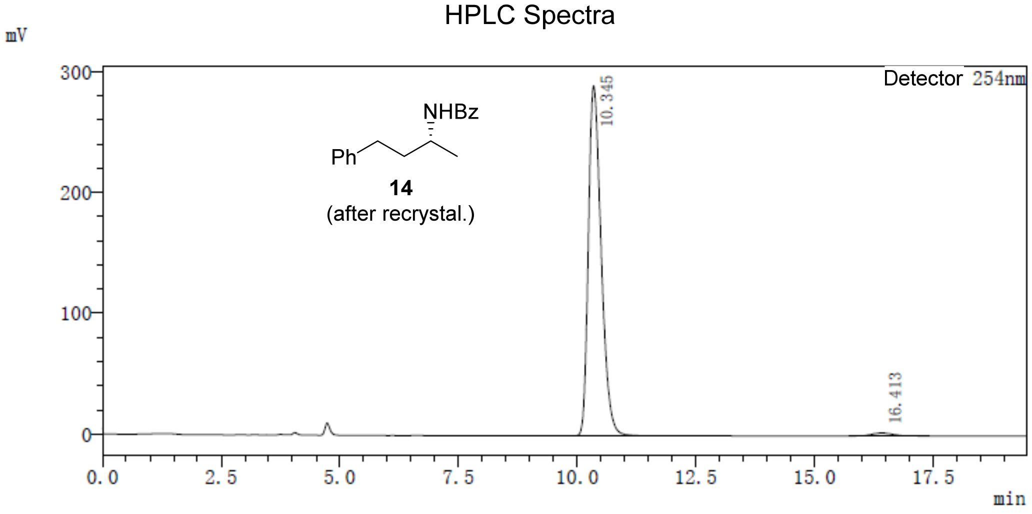

Area Percent Report

Detector 254nm

Number	Rremaining Time	Area	Height	Note	Area %
1	10.345	5433506	289501		98.607
2	16.413	76782	2491		1.393
Total		5510288	291992		100.000

Supplementary Figure 172. HPLC spectrum for **14**

HPLC Condition : AD-H, n-hexane/iPrOH = 96/4, 0.5 ml/min, 220 nm

HPLC Spectra

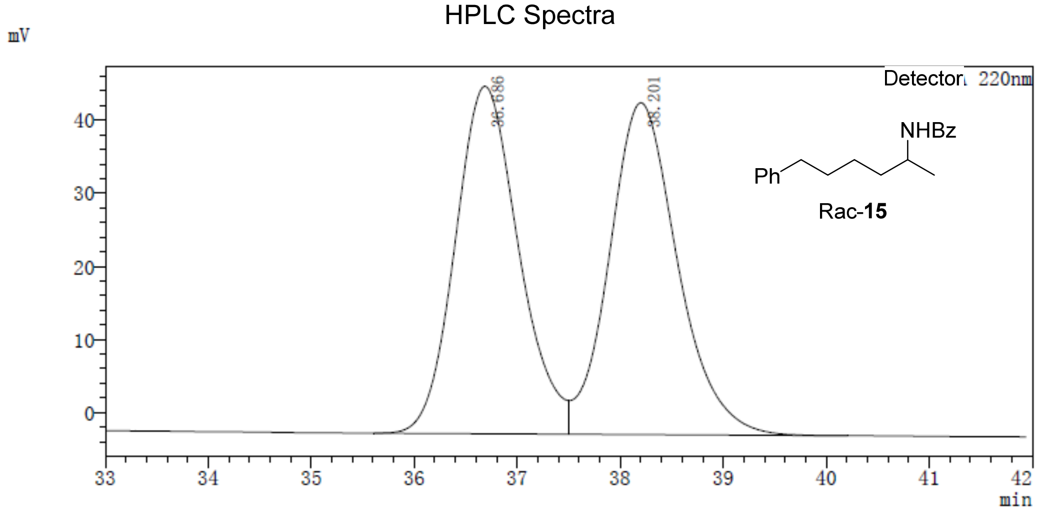

Area Percent Report

Detector: 220nm

Number	Rremaining Time	Area	Height	Note	Area %
1	36.686	2038977	47538		49.343
2	38.201	2093238	45402	V	50.657
Total		4132215	92940		100.000

HPLC Condition : AD-H, n-hexane/iPrOH = 96/4, 0.5 ml/min, 220 nm

HPLC Spectra

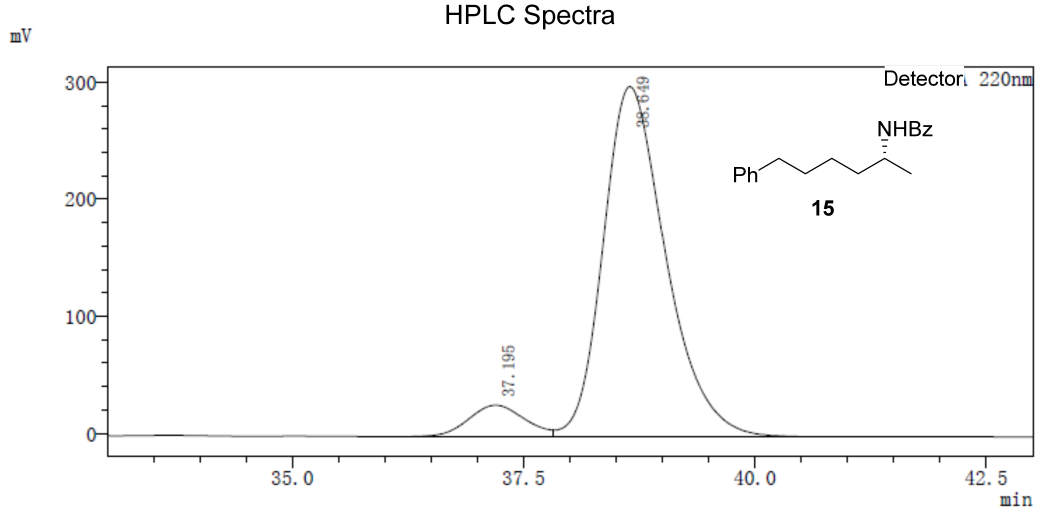

Area Percent Report

Detector: 220nm

Number	Rremaining Time	Area	Height	Note	Area %
1	37.195	1124521	26783		7.400
2	38.649	14071420	299093	V	92.600
Total		15195941	325876		100.000

Supplementary Figure 173. HPLC spectrum for **15**

HPLC Condition : OD-H, n-hexane/iPrOH = 90/10, 1.0 ml/min, 254 nm

HPLC Spectra

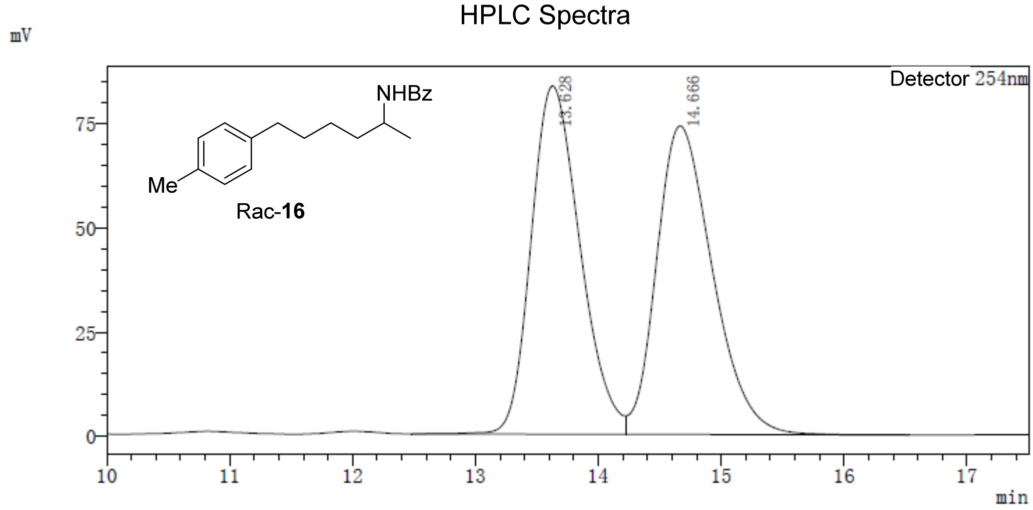

Area Percent Report

Detector 254nm

Number	Rremaining Time	Area	Height	Note	Area %
1	13.628	2316531	83546		49.751
2	14.666	2339751	74036	V	50.249
Total		4656283	157582		100.000

HPLC Condition : OD-H, n-hexane/iPrOH = 90/10, 1.0 ml/min, 254 nm

HPLC Spectra

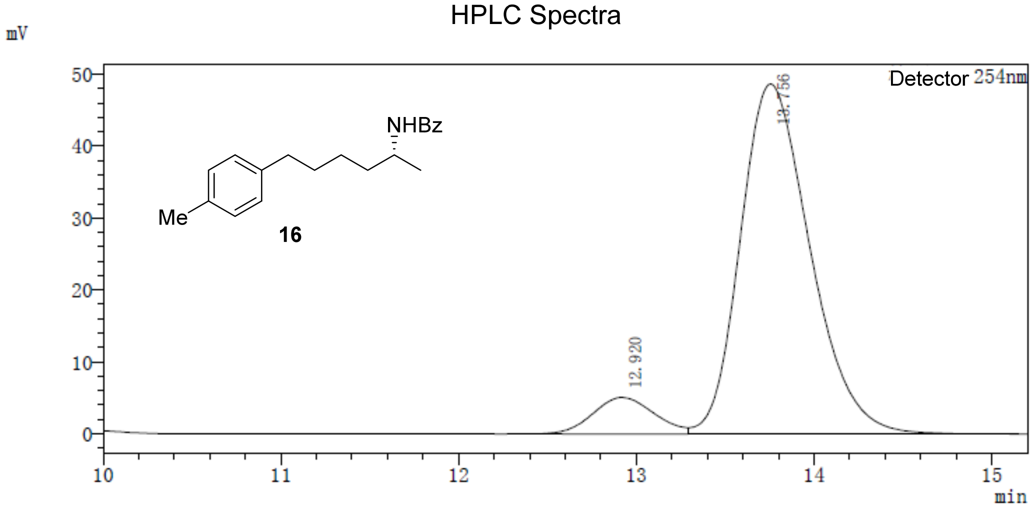

Area Percent Report

Detector 254nm

Number	Rremaining Time	Area	Height	Note	Area %
1	12.920	122015	5078		8.427
2	13.756	1325862	48667	V	91.573
Total		1447877	53746		100.000

Supplementary Figure 174. HPLC spectrum for **16**

HPLC Condition : OD-H, n-hexane/iPrOH = 90/10, 1.0 ml/min, 220 nm

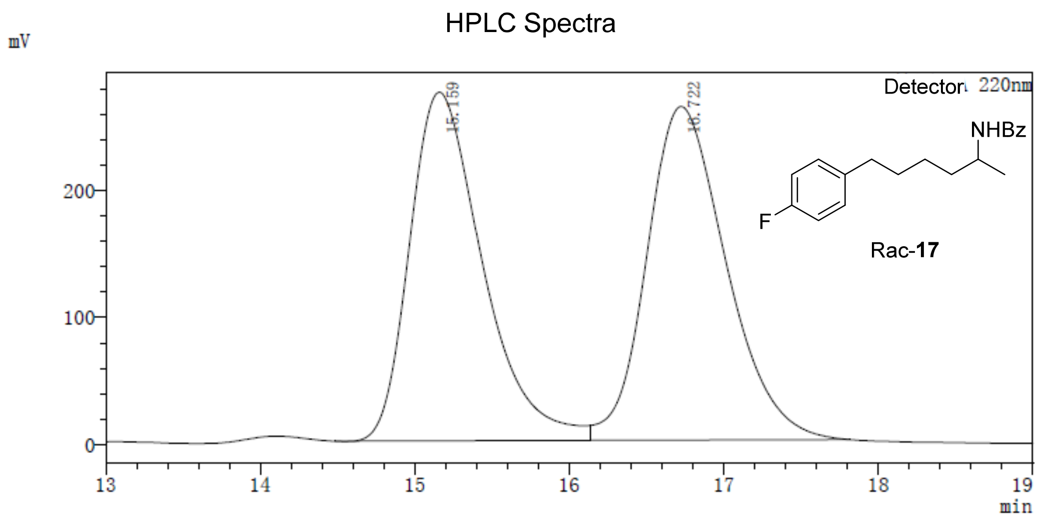

Area Percent Report

Detector 220nm

Number	Rremaining Time	Area	Height	Note	Area %
1	15.159	9026893	275163	M	48.996
2	16.722	9396769	263240	V M	51.004
Total		18423662	538403		100.000

HPLC Condition : OD-H, n-hexane/iPrOH = 90/10, 1.0 ml/min, 220 nm

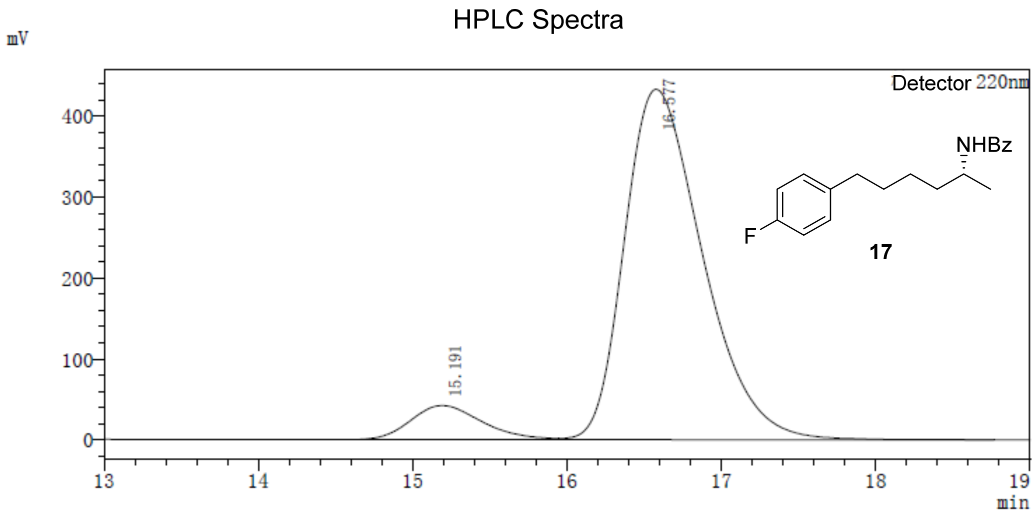

Area Percent Report

Detector 220nm

Number	Rremaining Time	Area	Height	Note	Area %
1	15.191	1353747	42156		8.169
2	16.577	15218376	432770	V	91.831
Total		16572124	474925		100.000

Supplementary Figure 175. HPLC spectrum for **17**

HPLC Condition : OD-H, n-hexane/iPrOH = 90/10, 1.0 ml/min, 220 nm

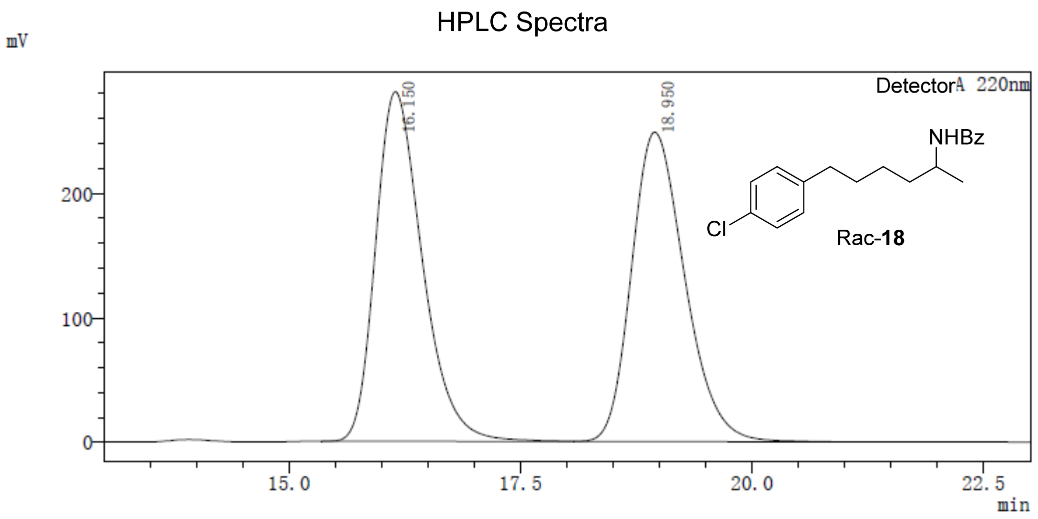

Area Percent Report

Detector 220nm

Number	Rremaining Time	Area	Height	Note	Area %
1	16.150	9902232	280713		49.980
2	18.950	9910156	248512	V	50.020
Total		19812388	529225		100.000

HPLC Condition : OD-H, n-hexane/iPrOH = 90/10, 1.0 ml/min, 220 nm

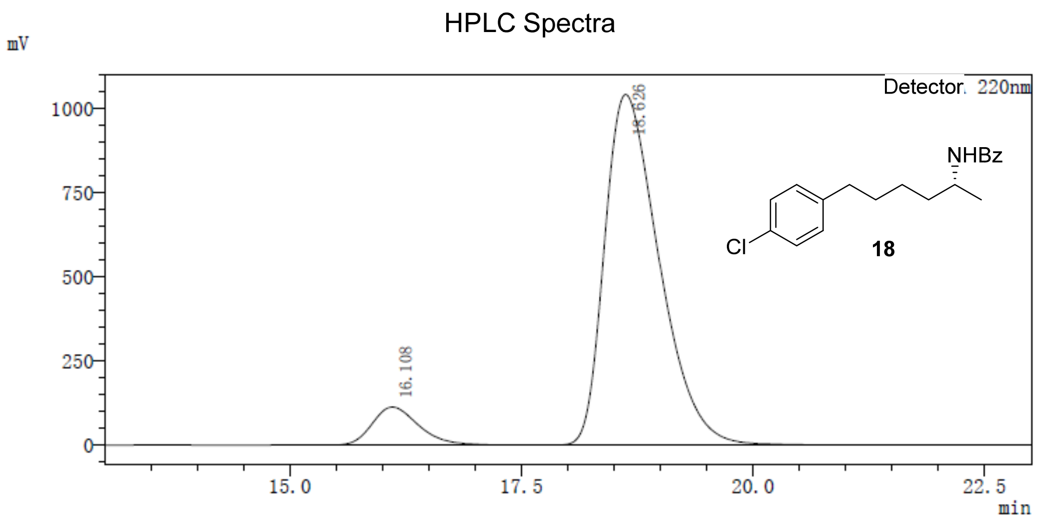

Area Percent Report

Detector 220nm

Number	Rremaining Time	Area	Height	Note	Area %
1	16.108	3967666	113105		8.512
2	18.626	42645249	1042205	V	91.488
Total		46612914	1155310		100.000

Supplementary Figure 176. HPLC spectrum for **16**

HPLC Condition : OD-H, n-hexane/iPrOH = 90/10, 1.0 ml/min, 254 nm

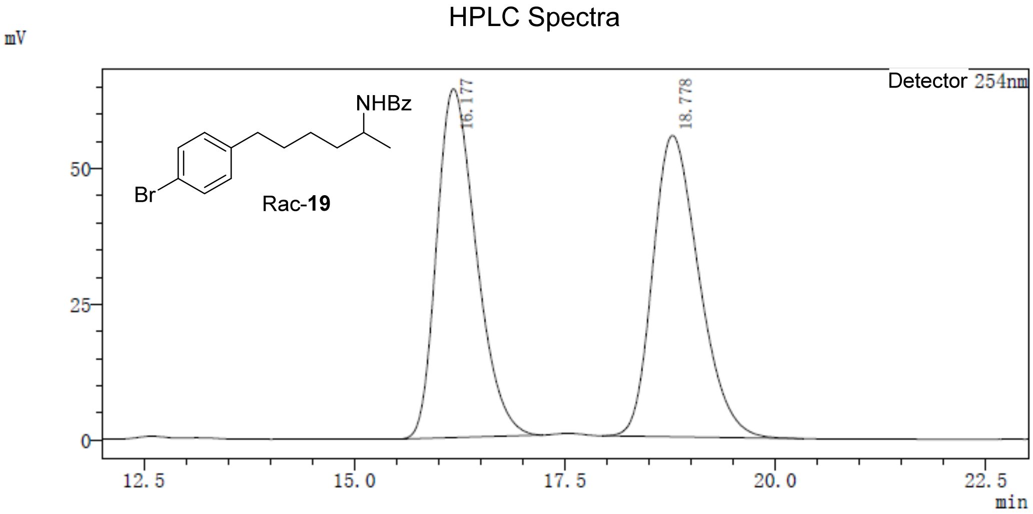

Area Percent Report

Detector 254nm

Number	Rremaining Time	Area	Height	Note	Area %
1	16.177	2099313	64266		49.695
2	18.778	2125048	55530		50.305
Total		4224361	119796		100.000

HPLC Condition : OD-H, n-hexane/iPrOH = 90/10, 1.0 ml/min, 254 nm

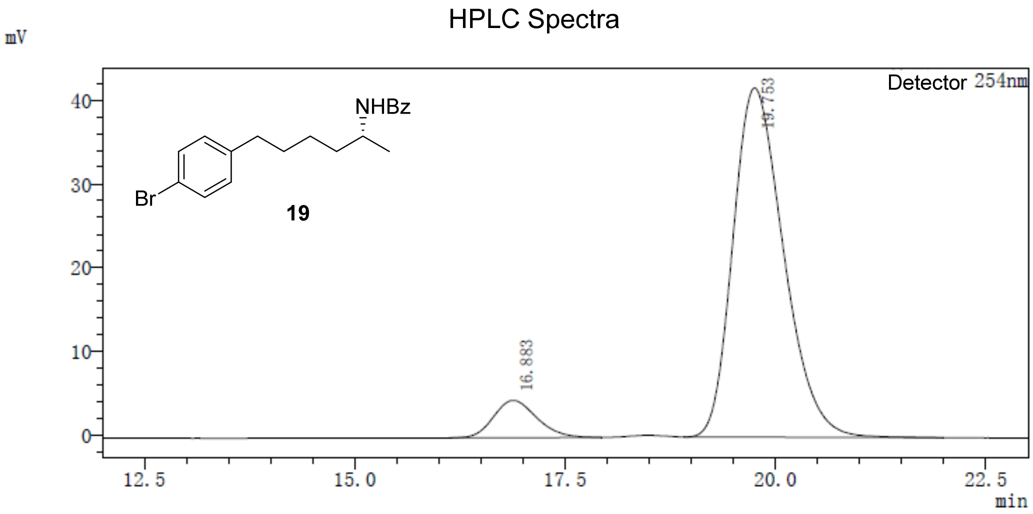

Area Percent Report

Detector 254nm

Number	Rremaining Time	Area	Height	Note	Area %
1	16.883	159107	4444		8.370
2	19.753	1741860	41620		91.630
Total		1900967	46064		100.000

Supplementary Figure 177. HPLC spectrum for **19**

HPLC Condition : OD-H, n-hexane/iPrOH = 90/10, 1.0 ml/min, 254 nm

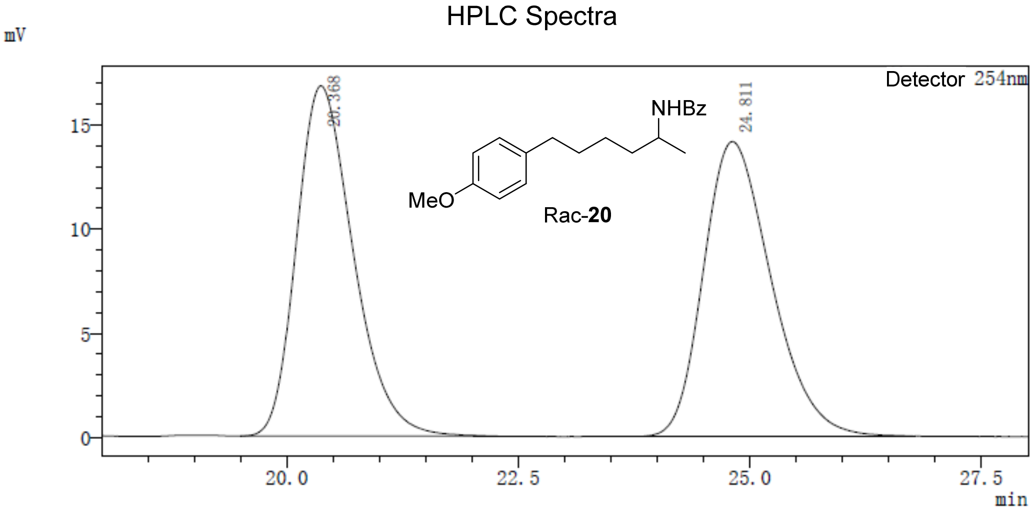

Area Percent Report

Detector 254nm

Number	Rremaining Time	Area	Height	Note	Area %
1	20.368	716572	16812		49.848
2	24.811	720942	14150		50.152
Total		1437514	30962		100.000

HPLC Condition : OD-H, n-hexane/iPrOH = 90/10, 1.0 ml/min, 254 nm

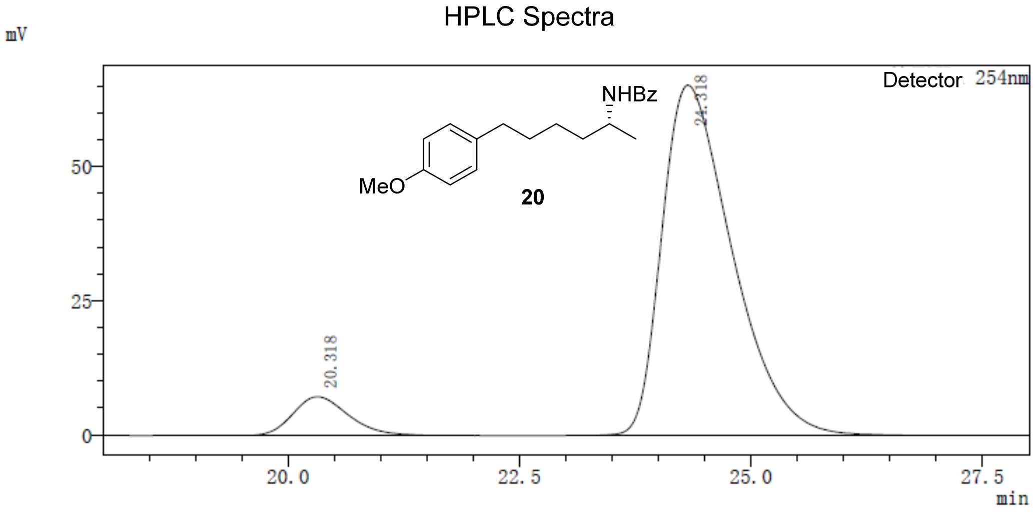

Area Percent Report

Detector 254nm

Number	Rremaining Time	Area	Height	Note	Area %
1	20.318	302244	7231		8.054
2	24.318	3450555	65298		91.946
Total		3752799	72528		100.000

Supplementary Figure 178. HPLC spectrum for **20**

HPLC Condition : OD-H, n-hexane/iPrOH = 90/10, 1.0 ml/min, 254 nm

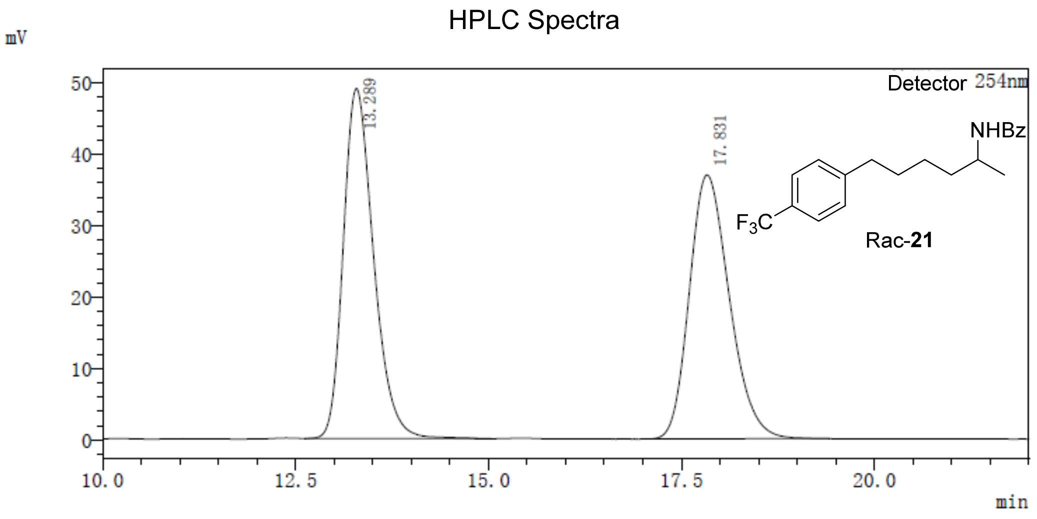

Area Percent Report

Detector 254nm

Number	Rremaining Time	Area	Height	Note	Area %
1	13.289	1339194	48992		50.063
2	17.831	1335832	36953		49.937
Total		2675025	85945		100.000

HPLC Condition : OD-H, n-hexane/iPrOH = 90/10, 1.0 ml/min, 254 nm

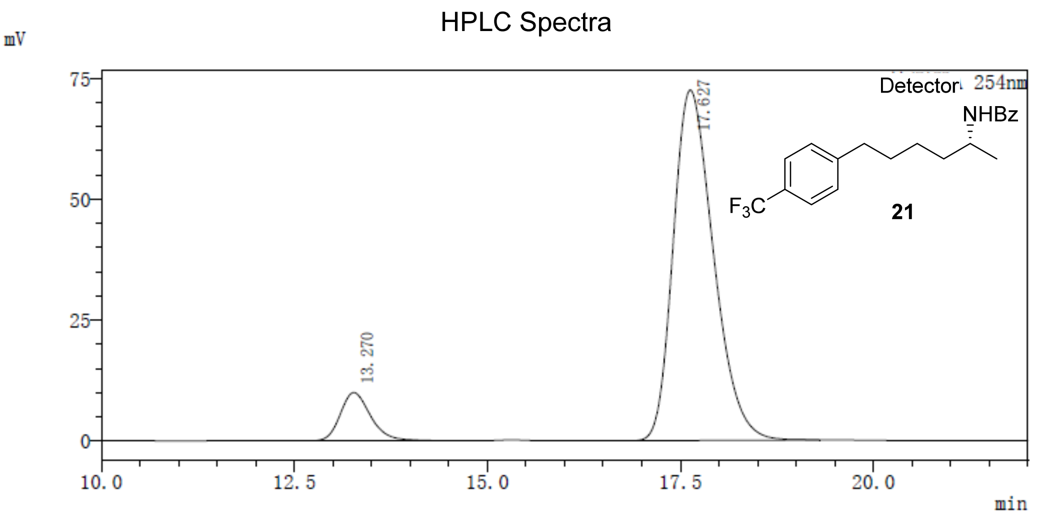

Area Percent Report

Detector 254nm

Number	Rremaining Time	Area	Height	Note	Area %
1	13.270	269698	9915		9.303
2	17.627	2629441	72440		90.697
Total		2899139	82355		100.000

Supplementary Figure 179. HPLC spectrum for **21**

HPLC Condition : OD-H, n-hexane/iPrOH =90/10, 1.0 ml/min, 220 nm

HPLC Spectra

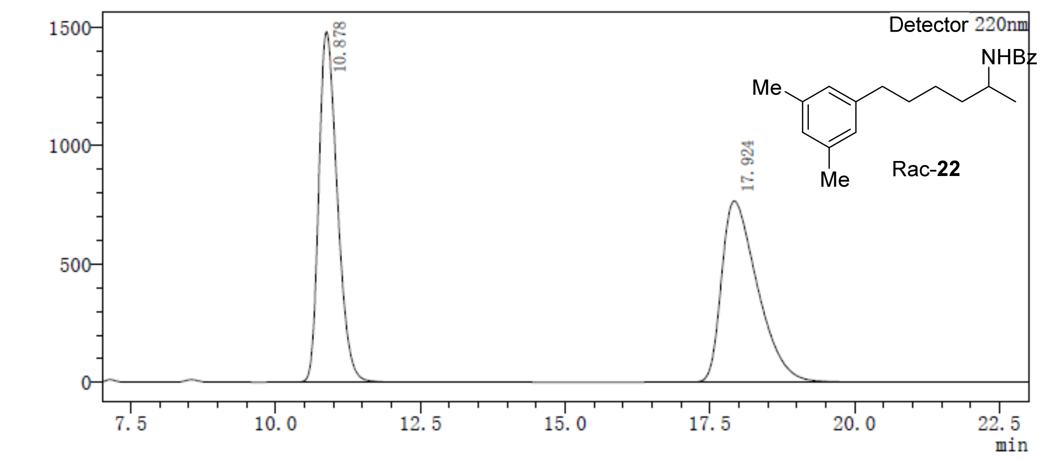

Area Percent Report

Detector 220nm

Number	Rremaining Time	Area	Height	Note	Area %
1	10.878	32850829	1478964		49.773
2	17.924	33150933	764900		50.227
Total		66001762	2243863		100.000

HPLC Condition : OD-H, n-hexane/iPrOH =90/10, 1.0 ml/min, 220 nm

HPLC Spectra

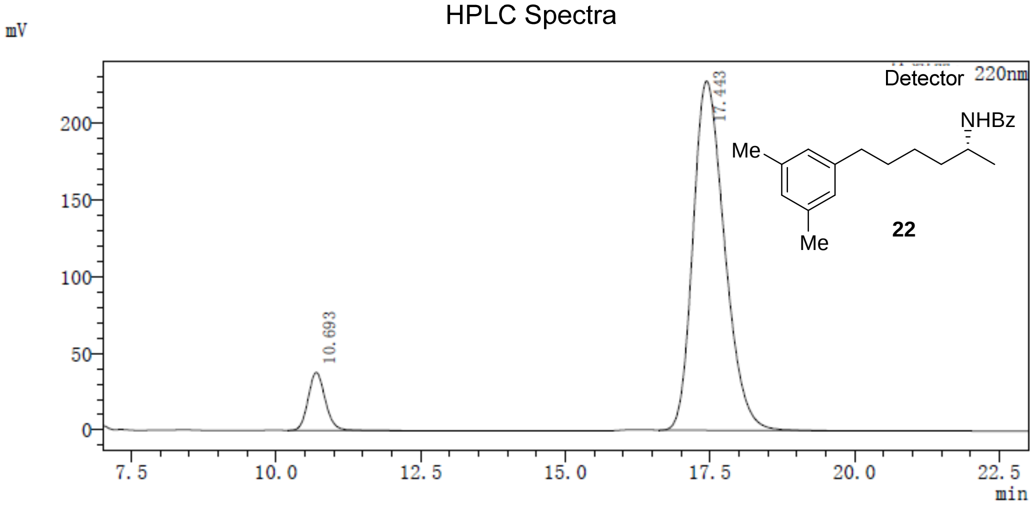

Area Percent Report

Detector 220nm

Number	Rremaining Time	Area	Height	Note	Area %
1	10.693	777931	37744		8.263
2	17.443	8636519	227380		91.737
Total		9414450	265123		100.000

Supplementary Figure 180. HPLC spectrum for **22**

HPLC Condition : OD-H, n-hexane/iPrOH = 85/15, 1.0 ml/min, 220 nm

HPLC Spectra

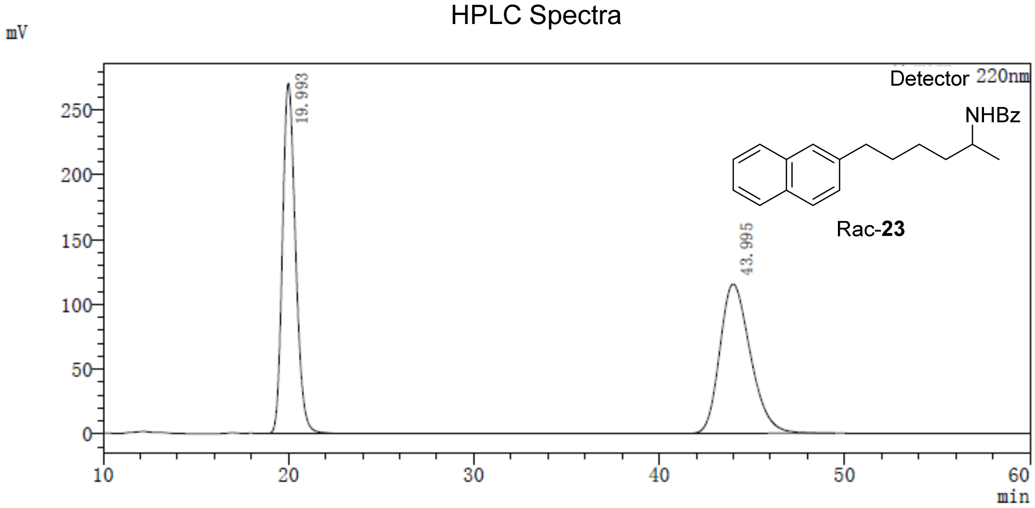

Area Percent Report

Detector 220nm

Number	Rremaining Time	Area	Height	Note	Area %
1	19.993	13219529	270371		49.940
2	43.995	13251296	115241		50.060
Total		26470825	385612		100.000

HPLC Condition : OD-H, n-hexane/iPrOH = 85/15, 1.0 ml/min, 220 nm

HPLC Spectra

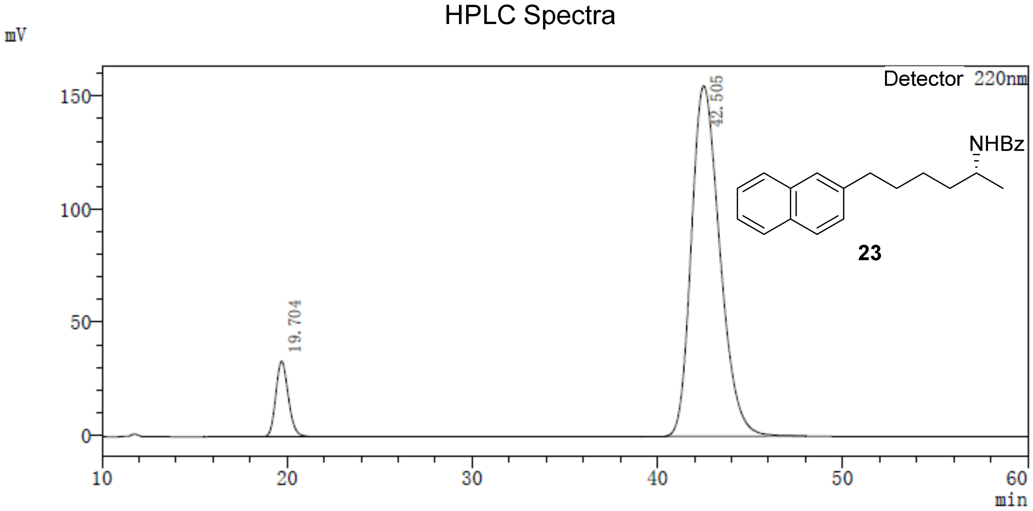

Area Percent Report

Detector 220nm

Number	Rremaining Time	Area	Height	Note	Area %
1	19.704	1570943	33399		8.646
2	42.505	16597735	154967		91.354
Total		18168678	188366		100.000

Supplementary Figure 181. HPLC spectrum for **23**

HPLC Condition : OD-H, n-hexane/iPrOH = 80/20, 1.0 ml/min, 254 nm

HPLC Spectra

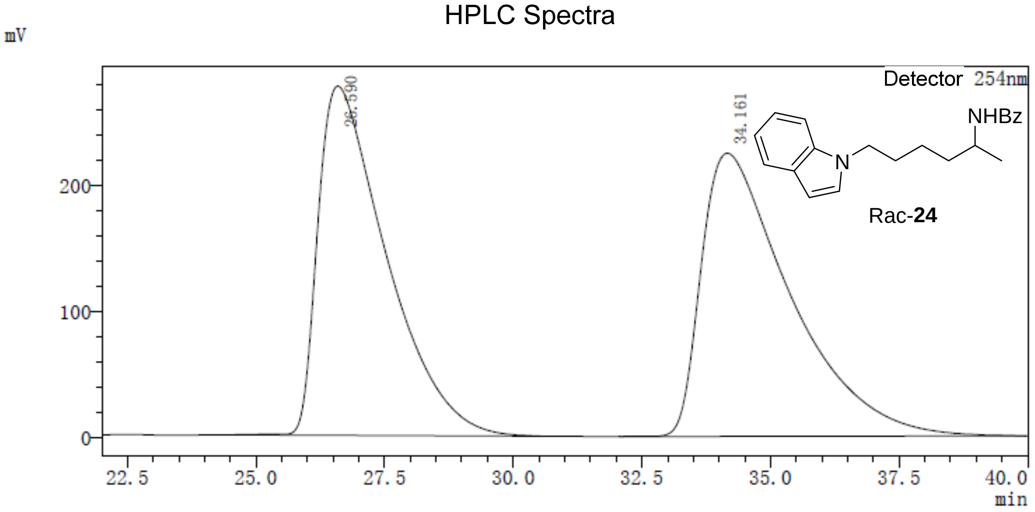

Area Percent Report

Detector 254nm

Number	Rremaining Time	Area	Height	Note	Area %
1	26.590	25611800	276727	M	48.617
2	34.161	27068498	224420	M	51.383
Total		52680298	501148		100.000

HPLC Condition : OD-H, n-hexane/iPrOH = 80/20, 1.0 ml/min, 254 nm

HPLC Spectra

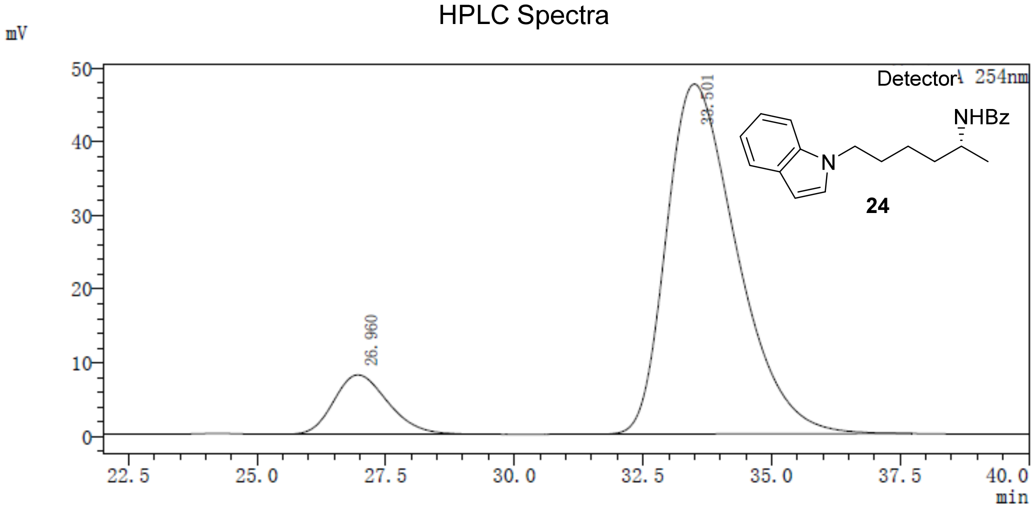

Area Percent Report

Detector 254nm

Number	Rremaining Time	Area	Height	Note	Area %
1	26.960	586710	8042		11.476
2	33.501	4525671	47453		88.524
Total		5112380	55495		100.000

Supplementary Figure 182. HPLC spectrum for **24**

HPLC Condition : OD-H, n-hexane/iPrOH = 90/10, 1.0 ml/min, 254 nm

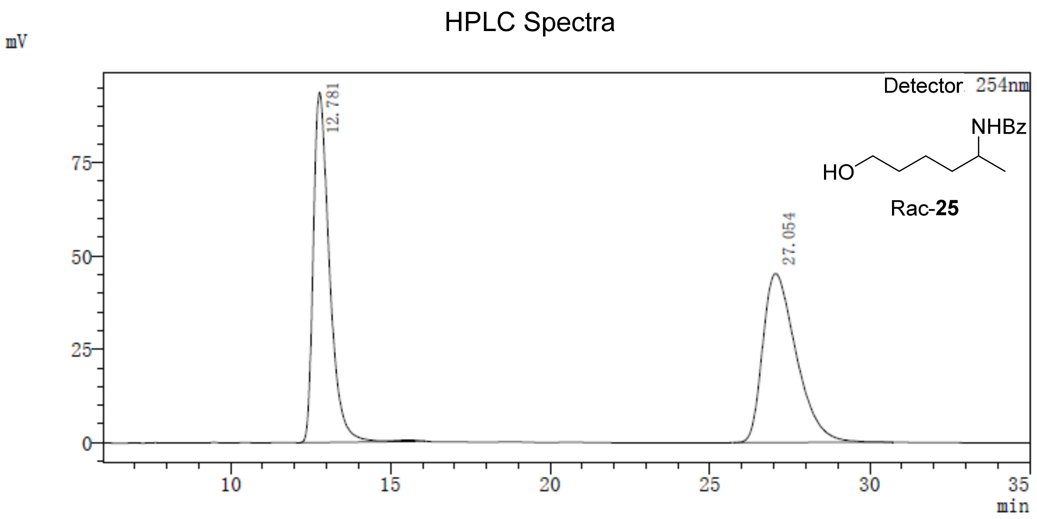

Area Percent Report

Detector 254nm

Number	Rremaining Time	Area	Height	Note	Area %
1	12.781	3305164	93821	M	49.958
2	27.054	3310707	45281		50.042
Total		6615871	139103		100.000

HPLC Condition : OD-H, n-hexane/iPrOH = 90/10, 1.0 ml/min, 220 nm

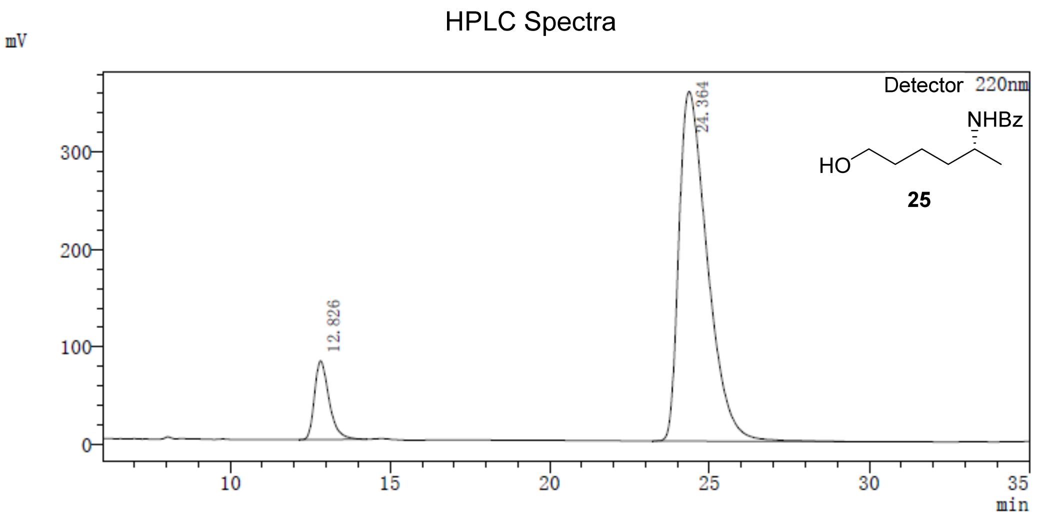

Area Percent Report

Detector 220nm

Number	Rremaining Time	Area	Height	Note	Area %
1	12.826	2579937	80754		10.413
2	24.364	22195706	358626		89.587
Total		24775644	439380		100.000

Supplementary Figure 183. HPLC spectrum for **25**

HPLC Condition : OD-H, n-hexane/iPrOH = 90/10, 1.0 ml/min, 254 nm

HPLC Spectra

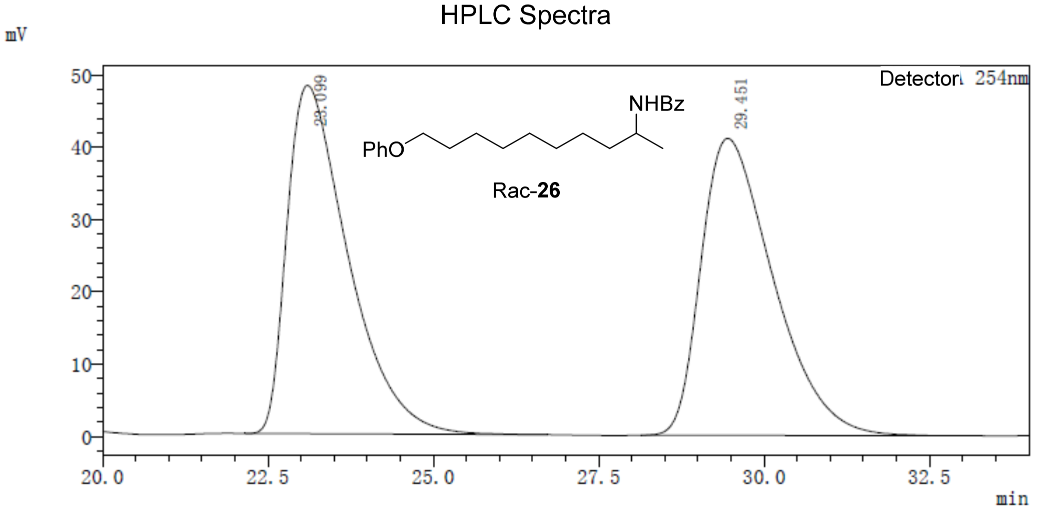

Area Percent Report

Detector: 254nm

Number	Rremaining Time	Area	Height	Note	Area %
1	23.099	3106552	48197		49.867
2	29.451	3123133	41084		50.133
Total		6229685	89281		100.000

HPLC Condition : OD-H, n-hexane/iPrOH = 90/10, 1.0 ml/min, 254 nm

HPLC Spectra

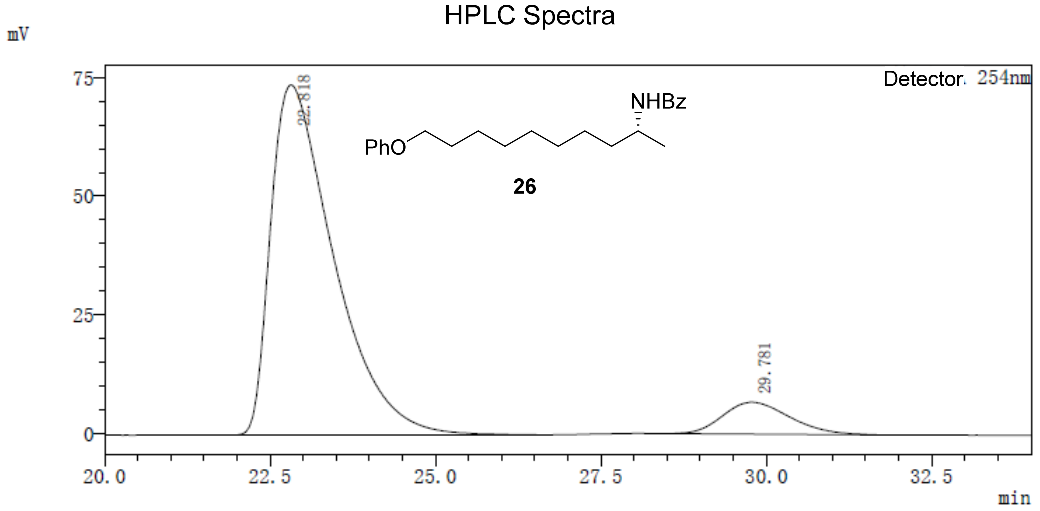

Area Percent Report

Detector: 254nm

Number	Rremaining Time	Area	Height	Note	Area %
1	22.818	4904864	73925		91.190
2	29.781	473838	6711		8.810
Total		5378701	80636		100.000

Supplementary Figure 184. HPLC spectrum for **26**

HPLC Condition : OD-H, n-hexane/iPrOH = 90/10, 1.0 ml/min, 254 nm

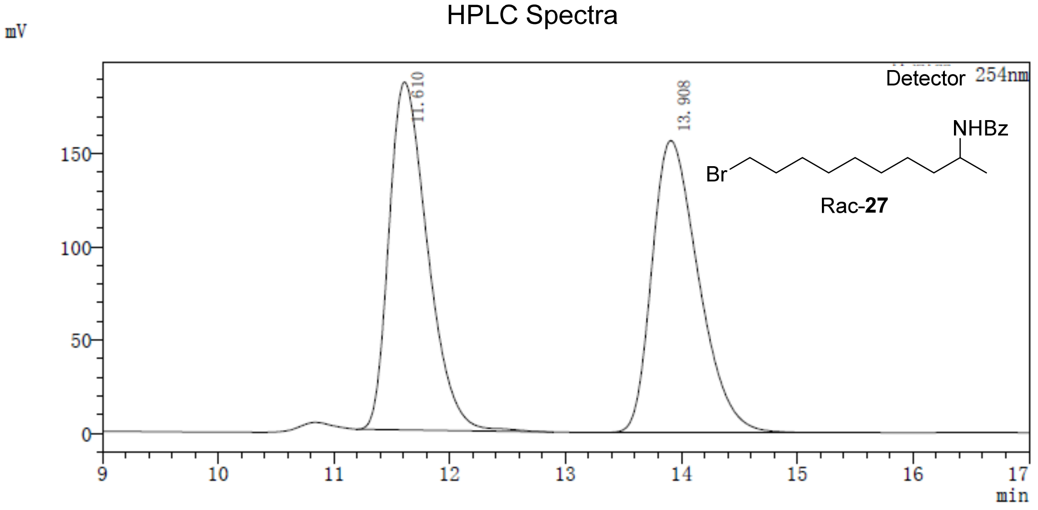

Area Percent Report

Detector 254nm

Number	Rremaining Time	Area	Height	Note	Area %
1	11.610	4323228	186577		49.694
2	13.908	4376494	156599		50.306
Total		8699722	343177		100.000

HPLC Condition : OD-H, n-hexane/iPrOH = 90/10, 1.0 ml/min, 254 nm

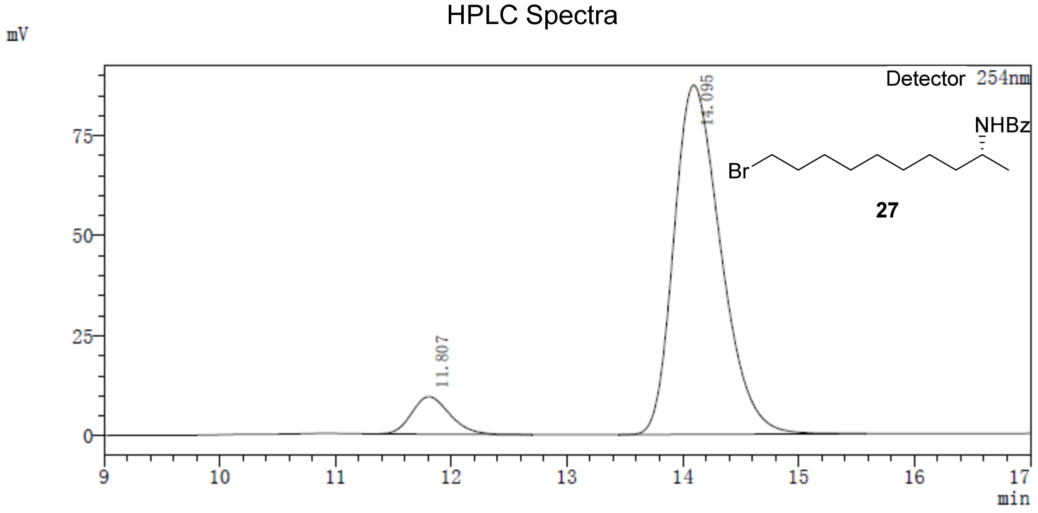

Area Percent Report

Detector 254nm

Number	Rremaining Time	Area	Height	Note	Area %
1	11.807	214372	9334		8.020
2	14.095	2458513	87257		91.980
Total		2672884	96591		100.000

Supplementary Figure 185. HPLC spectrum for **27**

Supplementary Tables

Supplementary Table 1. Optimizations for Hydroamination[a]

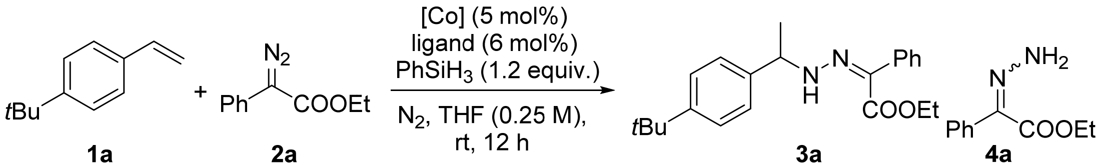

Entry	Cat.	Ligand	Yield[b] of 3a (%)	Recovery[b] of 2a (%)	Yield[b] of 4a (%)
1	**$Co(OAc)_2$**	-	<1	95	<1
2	**$Co(acac)_2$**	-	7	79	<5
3	**Co(tpp)**	-	<1	85	<1
4	**$Co(dmgH)_2(pyr)Cl$**	-	<1	95	<1
5	**$Co(OAc)_2$**	**L1**	<1	92	<1
6	**$Co(OAc)_2$**	**salen**	<1	84	<1
7	**$Co(OAc)_2$**	**PDI**	<1	89	<1
8	**$Co(OAc)_2$**	**bpy**	44	10	24
9	**$Co(OAc)_2$**	**phen**	37	25	17
10	**$Co(OAc)_2$**	**dppe**	<1	47	29
11	**$Co(OAc)_2$**	**L2**	88	<5	<1
12	**$Co(OAc)_2$**	**L3**	92	<5	<1
13	**$Co(OAc)_2$**	**L4**	96	<1	<1
14[c]	**$Co(OAc)_2$**	**L4**	94	<1	<5
15	**$Fe(OAc)_2$**	**L4**	<1	81	<1
16	**$Mn(OAc)_2$**	**L4**	<1	96	<1

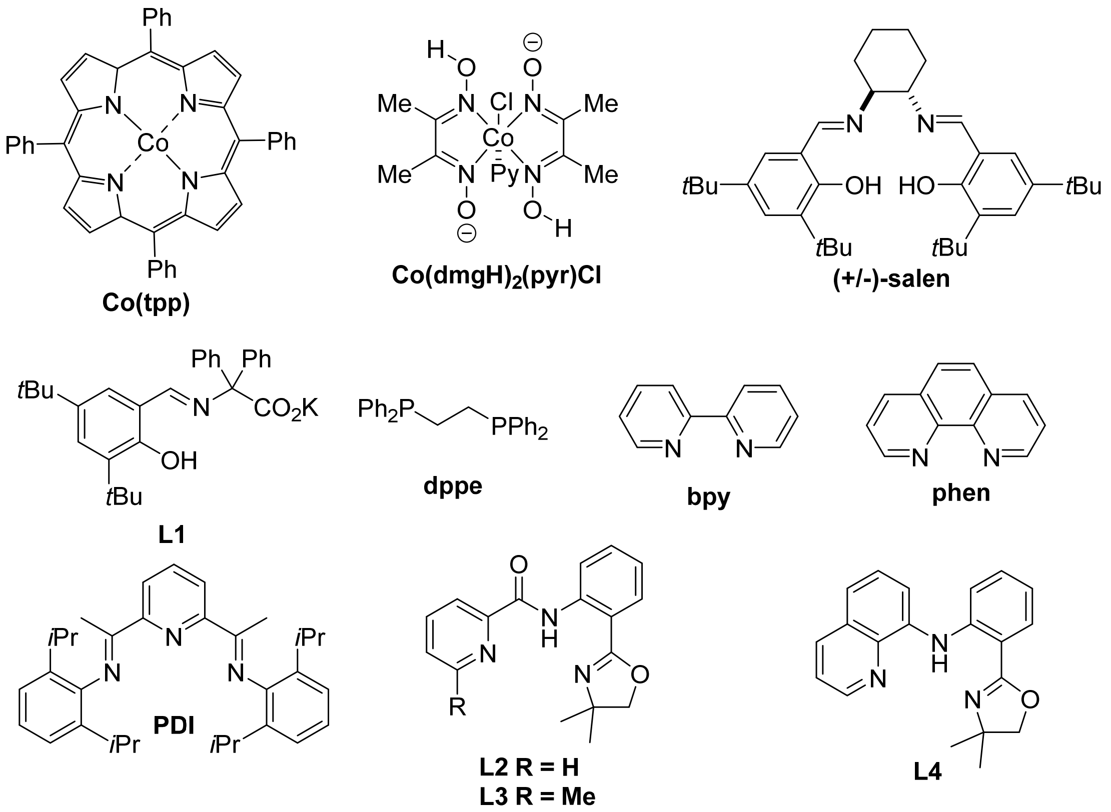

[a]The reaction was conducted using **1a** (0.36 mmol), **2a** (0.3 mmol), [Co] (5 mol %), ligand (6 mol%), $PhSiH_3$ (0.36 mmol) and THF (1.2 mL) under N_2 at rt for 12 h. [b]Determined by ^{1}H NMR using TMSPh as an internal standard. [c]$Co(OAc)_2$ (2.5 mol%), ligand (3 mol%).

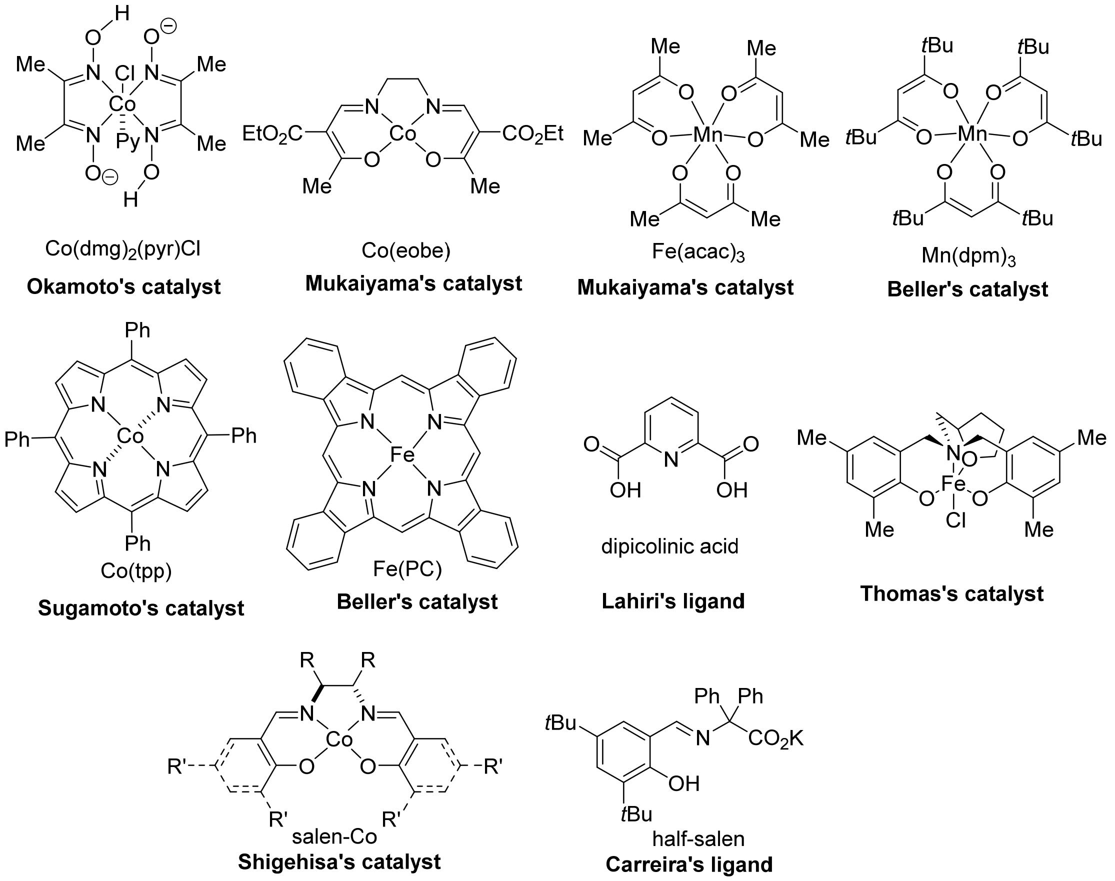

Supplementary Figure 186. Ligand or catalyst for metal-catalyzed alkene radical hydroamination via HAT:

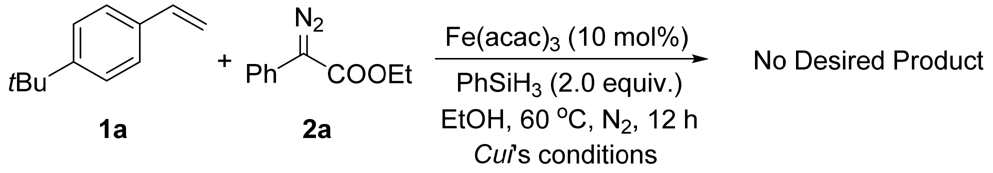

Cui and co-workers[1] reported Fe-catalyzed olefin hydroamination with diazo compounds for hydrazone synthesis via HAT process. A broad scope of alkyl group substituted alkenes could undergo this protocol to deliver corresponding hydroamination products. But when we runned *Cui*'s conditions with 4-*tert*-butylstyrene (**1a**) as a substrate, no desired product was obtained. So, it is highly desirable for the development of hydroamination of vinylarenes.

Supplementary Methods

General Information

Ether, THF, dioxane and toluene were distilled from sodium benzophenone ketyl prior to use. EA (Ethyl acetate) was distilled from potassium carbonate prior to use. NaO*t*Bu (98%) and $Co(OAc)_2$ (99.7%) were purchased from Aladdin and used as received. $Pd_2(dba)_3$ (98%) was purchased from Zhejiang Metallurgical Research Institute and used as received. 1,1'-Bis(diphenylphosphino)ferrocene (dppf) (98%) was purchased from Energy and used as received. 2-Ethoxyethanol was purchased from Adamas-beta and used as received. The other commercially available chemicals were used as received. Phenylsilane was prepared according to the previously reported procedures.[2] NMR spectra were recorded on a Bruker-400 instrument or a Bruker-500 instrument or a WNMR-I 400 instrument. ^{1}H NMR chemical shifts were referenced to tetramethylsilane signal (0 ppm), ^{13}C NMR chemical shifts were referenced to the solvent resonance (77.00 ppm, $CDCl_3$). ^{2}D NMR chemical shifts were referenced to $CDCl_3$ signal (7.26 ppm). The following abbreviations (or combinations) were used to explain multiplicities: s = singlet, d = doublet, t = triplet, m = multiplet, br = broad, q = quadruplet. HPLC analyses were performed on a Shimadzu SPD-20A. High-resolution mass spectra (HRMS) were recorded on Waters GCT Premier (GC-TOF) or Waters XEVO (LC-TOF). IR spectra were recorded on a Perkin-Elmer Spectrum One FTIR spectrometer with diamond ATR accessory. X-ray diffraction data was obtained on Rigaku Gemini A Ultra. Optical rotation data was obtained on PerkinElmer Model 341 Polarimeter. Melting point data was obtained on WRR melting point apparatus.

Procedures for the Preparations of Ligands and Metal Complexes

Co(dmgH)$_2$(pyr)Cl,[3] **PDI,**[4] **(+/-)-Salen,**[5] **L1,**[6] **L2**[7] were prepared according to the previously reported procedures. **S1**,[7]**2**,[8] **S3**[9] were prepared according to the literatures.

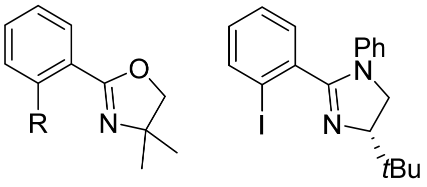

S1: R = NH$_2$
S2: R = Br
S3

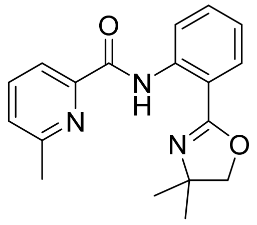

***N*-(2-(4,4-dimethyl-4,5-dihydrooxazol-2-yl)phenyl)-6-methylpicolinamide (L3).**

Prepared according to the previously reported procedure[10] with modification using 6-methylpicolinic acid (2.06 g, 15.0 mmol), **S1** (2.30 g, 12.0 mmol),[7] DMAP (0.170 g, 1.50 mmol), DCC (4.70 g, 22.5 mmol) and DCM (100 mL). After 12 h, the reaction mixture was quenched with DCM (100 mL), washed with water (100 mL), concentrated in vacuo, and purified by column chromatography using PE/EA (10/1, 300 mL) as the eluent to afford **L3** (3.40 g, 11.0 mmol, 92% yield) as a white solid. M.p.: 119.6-120.6 ^{o}C. IR (neat): 3089, 1681, 1643, 1584, 1524, 1446 cm^{-1}. ^{1}H NMR: (400 MHz, CDCl$_3$) δ 13.53 (br, 1H), 9.04 (dd, J = 8.2, 0.8 Hz, 1H), 8.11 (d, J = 7.6 Hz, 1H), 7.90 (dd, J = 8.0, 1.6 Hz, 1H), 7.76 (t, J = 8.0 Hz, 1H), 7.51 (dd, J = 8.2, 1.6 Hz, 1H), 7.31 (d, J = 7.6 Hz, 1H), 7.12 (dd, J = 8.0, 1.6 Hz, 1H), 4.09 (s, 2H), 2.72 (s, 3H), 1.50 (s, 6H); ^{13}C NMR: (100 MHz, CDCl$_3$) δ 164.4, 161.3, 157.1, 150.6, 139.6, 137.3, 132.2, 129.2, 125.8, 122.6, 120.4, 120.1, 114.9, 77.8, 68.3, 28.6, 24.6; HRMS (ESI) calculated for $C_{18}H_{20}N_3O_2$ (M+H$^+$) requires m/z 310.1556, found m/z 310.1560.

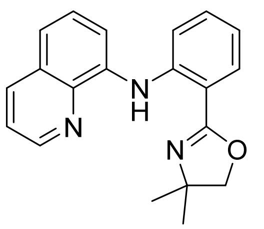

***N*-(2-(4,4-dimethyl-4,5-dihydrooxazol-2-yl)phenyl)quinoline-8-amine (L4).**

Prepared according to a previously reported procedure[11] with modification. To an oven-dried 100 mL flame-dried Schlenk flask, $Pd_2(dba)_3$ (0.31 g, 0.31 mmol), dppf (0.36 g, 0.63 mol), toluene (30 mL), **S2** (3.10 g , 12.5 mmol),[8] 8-aminoquinoline (1.60 g, 12.5 mmol) and *t*BuONa (2.50 g, 25.0 mmol) were added in sequence under the atmosphere of nitrogen. Then, the solution was refluxed for 12 h, thereafter, cooled down to room temperature. All volatiles were removed from the solution via rotary evaporation to give a brown sticky oil. This mixture was added with DCM (60 mL), washed with water (60 mL). The aqueous layer was extracted with DCM (60 mL x 2). The combined organic layers were dried over anhydrous Na_2SO_4, filtered, concentrated, and purified by flash column chromatography using PE (100 mL) to PE/EA (20/1, 500 mL) as the eluent to afford **L4** (3.16 g, 10.0 mmol, 90% yield) as a yellow solid. M.p.: 111.5-112.6 °C. IR (neat): 3254, 2929, 2837, 1700, 1501 cm^{-1}. ^1H NMR: (400 MHz, $CDCl_3$) δ 11.73 (br, 1H), 8.89-8.86 (m, 1H), 8.12-8.08 (m, 1H), 7.87-7.83 (m, 1H), 7.81-7.77 (m, 2H), 7.46-7.30 (m, 4H), 6.89-6.83 (m, 1H), 4.06 (s, 2H), 1.44 (s, 6H); ^{13}C NMR: (100 MHz, $CDCl_3$) δ 161.4, 148.0, 143.5, 140.8, 139.4, 135.8, 131.4, 130.0, 129.1, 126.8, 121.4, 118.6, 118.3, 115.1, 113.7, 111.9, 77.7, 68.1, 28.7; HRMS (ESI) calculated for $C_{20}H_{20}N_3O$ ($M+H^+$) requires m/z 318.1606, found m/z 318.1608.

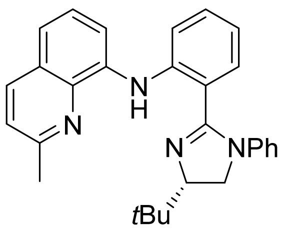

(*S*)-*N*-(2-(4-(*tert*-butyl)-1-phenyl-4,5-dihydro-1*H*-imidazol-2-yl)phenyl)-2-methylquinolin-8-amine (L5)-IPAQ.

Prepared according to a previously reported procedure[11] with modification. To an oven-dried 25 mL flame-dried Schlenk flask, $Pd_2(dba)_3$ (0.0445 g, 0.049 mmol), dppf (0.0547 g, 0.10 mmol), toluene (5.0 mL). **S3** (0.4051 g, 1.0 mmol),[9] 2-methylquinolin-8-amine (0.1607 g, 1.0 mmol) and NaO*t*Bu (0.1940 g, 2.0 mmol) were added in sequence under the atmosphere of nitrogen. Then, the solution was refluxed for 12 h, thereafter, cooled down to room temperature. All volatiles were removed from the solution via rotary evaporation to give a brown sticky

oil. This mixture was added with DCM (60 mL), washed with water (60 mL), extracted with DCM (60 mL x 2). The combined organic layers were dried over anhydrous Na_2SO_4, filtered, concentrated, and purified by flash column chromatography using PE/EA (10/1, 300 mL) to PE/EA (5/1, 500 mL) as the eluent to afford **L5** (0.3066 g, 0.71 mmol, 70% yield) as a light brown solid. M.p.: 80.1-82.2 °C. IR (neat): 3298, 3048, 2954, 1613, 1580, 1521, 1496 cm^{-1}. Optical Rotation: $[\alpha]^{20}_D$ = +137.7 (c 1.00, $CHCl_3$). ^1H NMR (400 MHz, $CDCl_3$): δ 9.93 (br, 1H), 7.97 (d, J = 8.4 Hz, 1H), 7.73 (d, J = 8.4 Hz, 1H), 7.50 (d, J = 8.0 Hz, 1H), 7.31-7.18 (m, 5H), 7.09 (dd, J = 8.0, 7.6 Hz, 2H), 6.91 (dd, J = 7.6, 7.2 Hz, 1H), 6.85-6.71 (m, 3H), 4.11 (dd, J = 10.8, 8.4 Hz, 1H), 4.01 (dd, J = 10.8, 9.2 Hz, 1H), 3.62 (dd, J = 9.2, 8.4 Hz, 1H), 2.74 (s, 3H), 1.00 (s, 9H); ^{13}C NMR (100 MHz, $CDCl_3$): δ 160.0, 156.5, 143.4, 141.7, 139.5, 139.1, 136.0, 130.7, 129.8, 128.5, 127.1, 125.7, 123.0, 122.3, 122.2, 121.2, 120.3, 119.1, 117.2, 109.8, 74.6, 54.0, 34.0, 26.1, 25.3. HRMS (EI) calculated for $C_{29}H_{30}N_4$ (M^+) requires m/z 434.2470, found m/z 434.2466.

Synthesis of Substrates

Alkenes were prepared according to Wittig Reaction or the previously reported procedures.[12] Ethyl 2-diazo-2-phenylacetate (**2a**) *tert*-butyl 2-diazo-2-phenylacetate (**2c**) were prepared according to the previously reported procedures.[13]

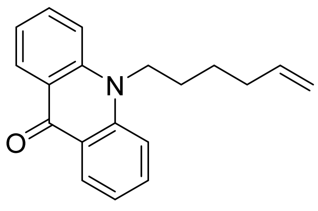

10-(hex-5-en-1-yl)acridin-9(10*H*)-one (1al)

Prepared according to the previously reported procedures[14] using acridin-9(10*H*)-one and 6-bromohex-1-ene as starting materials. M.p.: 110.0-111.4 °C. IR (neat): 3077, 2925, 2865, 1822, 1629, 1592, 1489, 1459 cm^{-1}. ^{1}H NMR: (400 MHz, $CDCl_3$) δ 8.61-8.57 (m, 2H), 7.76-7.70 (m, 2H), 7.52-7.47 (m, 2H), 7.32-7.27 (m, 2H), 5.91-5.80 (m, 1H), 5.13-5.02 (m, 2H), 4.38-4.31 (m, 2H), 2.23 (q, J = 7.2 Hz, 2H), 2.02-1.92 (m, 2H), 1.72-1.65 (m, 2H); ^{13}C NMR: (100 MHz, $CDCl_3$) δ 178.0, 141.7, 137.9, 133.9, 128.0, 122.5, 121.2, 115.4, 114.5, 46.0, 33.3, 26.5, 26.1; HRMS (ESI) calculated for $C_{19}H_{20}NO$ (M+H)$^+$ requires m/z 278.1545, found m/z 278.1553.

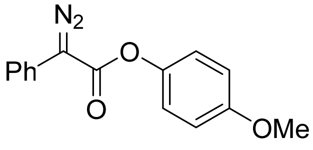

4-methoxyphenyl 2-diazo-2-phenylacetate (2b)

Prepared according to the previously reported procedures[15] using using 2-oxo-2-phenylacetic acid and 4-methoxyphenol as starting materials. IR (neat): 2836, 2089, 1716, 1599, 1503, 1460, 1352 cm^{-1}. ^{1}H NMR: (400 MHz, $CDCl_3$) δ 7.53 (d, J = 8.4 Hz, 2H), 7.41 (t, J = 8.0 Hz, 2H), 7.21 (t, J = 8.0 Hz, 1H), 7.10 (d, J = 8.8 Hz, 2H), 6.92 (d, J = 8.8 Hz, 2H), 3.81 (s, 3H); ^{13}C NMR: (100 MHz, $CDCl_3$) δ 163.9, 157.4, 143.7, 129.0, 126.1, 125.0, 124.0, 122.5, 114.5, 55.6; HRMS (ESI) calculated for $C_{15}H_{13}N_2O_3$ (M+H)$^+$ requires m/z 269.0926, found m/z 269.0932.

<u>**Cobalt-Catalyzed Hydroamination of Alkenes**</u>

General Procedure A for Hydroamination of Alkenes:

A 25 mL Schlenk flask equipped with a magnetic stirrer and a flanging rubber plug was dried with flame under vacuum. When cooled to ambient temperature, it was vacuumed and flushed with N_2 and repeated for three times. To the flask, $Co(OAc)_2$ (0.015 mmol), **L3** or **L4** (0.018 mol), THF (1.2 mL) were added. The flask was degassed and stirred for 30 min at room temperature. Then, $PhSiH_3$ (0.36 mol), diazo compound (0.3 mmol) and alkene (0.36 mmol) were added in sequence. After 12 h, the reaction was quenched with 10 ml of PE and the mixture was filtered through a pad of silica gel and washed with PE/EA (5/1, 50 mL). The combined filtrates were concentrated and purified by flash column chromatography using PE/EA as the eluent to afford the corresponding product.

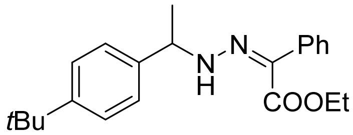

Ethyl (*Z*)-2-(2-(1-(4-(*tert*-butyl)phenyl)ethyl)hydrazono)-2-phenylacetate (3a). Prepared according to the general procedure A using 0.0579 g (0.36 mmol) of 4-*tert*-butylstyrene (**1a**), 48 μL (1.165 g/ml, 0.3 mmol) of ethyl 2-diazo-2-phenylacetate (**2a**), 45 μL (0.877 g/mL, 0.36 mmol) of $PhSiH_3$, 0.0028 g (0.015 mmol) of $Co(OAc)_2$, 0.0059g (0.018 mmol) of **L4**, and 1.2 mL (0.25 M) of THF. After 12 h, the reaction was worked up. The crude mixture was purified by flash column chromatography using PE (50 mL) to PE/EA (100/1, 200 mL) as the eluent to give 0.0974 g (0.86 mmol, 91% yield) of the title compound as a light yellow oil. IR (neat): 3251, 2964, 1737, 1669, 1513, 1449 cm^{-1}. ^{1}H NMR: (400 MHz, $CDCl_3$) δ 10.78 (d, J = 5.2 Hz, 1H), 7.58-7.50 (m, 2H), 7.41-7.33 (m, 2H), 7.33-7.25 (m, 4H), 7.25-7.18 (m, 1H), 4.90-4.65 (m, 1H), 4.24 (q, J = 7.2 Hz, 2H), 1.63 (d, J = 6.8 Hz, 3H), 1.31 (s, 9H), 1.28 (t, J = 7.2 Hz, 3H); ^{13}C NMR: (100 MHz, $CDCl_3$) δ 163.6, 150.2, 140.1, 137.2, 128.2, 127.6, 126.7, 126.2, 125.5, 125.3, 60.2, 59.9, 34.4, 31.3, 21.6, 14.2; HRMS (ESI) calculated for $C_{22}H_{28}N_2O_2Na$ (M+Na$^+$) requires m/z 375.2048, found m/z 375.2050.

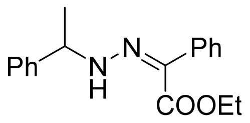

Ethyl (*Z*)-2-phenyl-2-(2-(1-phenylethyl)hydrazono)acetate (3b).

Prepared according to the general procedure A using 0.0380 g (0.36 mmol) of styrene (**1b**), 48 μL (1.165 g/ml, 0.3 mmol) of ethyl 2-diazo-2-phenylacetate (**2a**), 45 μL (0.877 g/mL, 0.36 mmol) of $PhSiH_3$, 0.0028 g (0.015 mmol) of $Co(OAc)_2$, 0.0059 g (0.018 mmol) of **L4**, and 1.2 mL (0.25 M) of THF. After 12 h, the reaction was worked up. The crude mixture was purified by flash column chromatography using PE (50 mL) to PE/EA (100/1, 200 mL) as the eluent to give 0.0813 g (0.27 mmol, 91% yield) of the title compound as a light yellow oil. IR (neat): 3248, 3059, 1735, 1669, 1514 cm^{-1}. ^{1}H NMR: (400 MHz, $CDCl_3$) δ 10.78 (d, J = 3.2 Hz, 1H), 7.52 (d, J = 7.2 Hz, 2H), 7.37-7.20 (m, 8H), 4.84-4.75 (m, 1H), 4.26 (q, J = 7.2 Hz, 2H), 1.64 (d, J = 6.8 Hz, 3H), 1.30 (t, J = 7.2 Hz, 3H); ^{13}C NMR: (100 MHz, $CDCl_3$) δ 163.7, 143.3, 137.2, 128.6, 128.3, 127.7, 127.4, 126.8, 126.5, 125.7, 60.3, 60.2, 21.7, 14.2; HRMS (ESI) calculated for $C_{18}H_{20}N_2NaO_2$ ($M+Na^+$) requires m/z 319.1422, found m/z 319.1414.

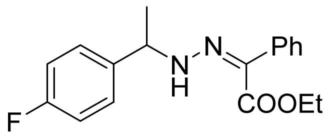

Ethyl (*Z*)-2-(2-(1-(4-fluorophenyl)ethyl)hydrazono)-2-phenylacetate (3c).

Prepared according to the general procedure A using 0.0450 g (0.36 mmol) of 4-fluorostyrene (**1c**), 48 μL (1.165 g/ml, 0.3 mmol) of ethyl 2-diazo-2-phenylacetate (**2a**), 45 μL (0.877 g/mL, 0.36 mmol) of $PhSiH_3$, 0.0028 g (0.015 mmol) of $Co(OAc)_2$, 0.0058 g (0.018 mmol) of **L4**, and 1.2 mL (0.25 M) of THF. After 12 h, the reaction was worked up. The crude mixture was purified by flash column chromatography using PE (50 mL) to PE/EA (50/1, 200 mL) as the eluent to give 0.0731 g (0.82 mmol, 78% yield) of the title compound as a light yellow oil. IR (neat): 3248, 2982, 1671, 1604, 1512, 1447 cm^{-1}. ^{1}H NMR: (400 MHz, $CDCl_3$) δ 10.70 (br, 1H), 7.51 (d, J = 6.8 Hz, 2H), 7.36-7.26 (m, 4H), 7.26-7.18 (m, 1H), 7.02 (t, J = 8.4 Hz, 2H), 4.84-4.68 (m, 1H), 4.26 (q, J = 7.2 Hz, 2H), 1.61 (d, J = 6.8 Hz, 3H), 1.30 (t, J = 7.2 Hz, 3H); ^{13}C NMR: (100 MHz, $CDCl_3$) δ 163.6, 162.1 (d, J = 244.2 Hz), 139.15 (d, J = 3.2 Hz), 137.1, 128.2, 128.14 (d, J = 8.0 Hz), 127.7, 126.9, 126.0, 115.4 (d, J =

21.5 Hz), 60.4, 59.4, 21.6, 14.1; ^{19}F NMR: (376 MHz, $CDCl_3$) δ -115.3; HRMS (ESI) calculated for $C_{18}H_{20}FN_2O_2$ (M+H$^+$) requires m/z 315.1509, found m/z 315.1518.

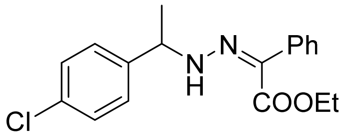

Ethyl (*Z*)-2-(2-(1-(4-chlorophenyl)ethyl)hydrazono)-2-phenylacetate (3d).

Prepared according to the general procedure A using 0.0582 g (0.36 mmol) of 4-chlorostyrene (**1d**), 48 μL (1.165 g/ml, 0.3 mmol) of ethyl 2-diazo-2-phenylacetate (**2a**), 45 μL (0.877 g/mL, 0.36 mmol) of $PhSiH_3$, 0.0027 g (0.015 mmol) of $Co(OAc)_2$, 0.0058 g (0.018 mmol) of **L3**, and 1.2 mL (0.25 M) of THF. After 12 h, the reaction was worked up. The crude mixture was purified by flash column chromatography using PE (50 mL) to PE/EA (100/1, 200 mL) as the eluent to give. 0.0858 g (0.26 mmol, 86% yield) of the title compound as a light yellow oil. IR (neat): 3247, 2981, 1736, 1671, 1598, 1515 cm^{-1}. ^{1}H NMR: (400 MHz, $CDCl_3$) δ 10.72 (d, *J* = 4.8 Hz, 1H), 7.54-7.48 (m, 2H), 7.34-7.19 (m, 7H), 4.78-4.70 (m, 1H), 4.25 (qd, *J* = 7.2, 1.2 Hz, 2H), 1.59 (d, *J* = 7.2 Hz, 3H), 1.29 (t, *J* = 7.2 Hz, 3H); ^{13}C NMR: (100 MHz, $CDCl_3$) δ 163.6, 142.0, 137.0, 133.0, 128.7, 128.2, 127.9, 127.7, 126.9, 126.3, 60.4, 59.4, 21.4, 14.1; HRMS (ESI) calculated for $C_{18}H_{19}ClN_2NaO_2$ (M+Na$^+$) requires m/z 353.1033, found m/z 353.1027.

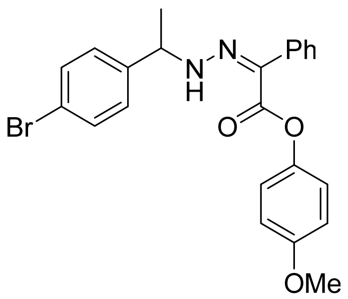

4-methoxyphenyl (*Z*)-2-(2-(1-(4-bromophenyl)ethyl)hydrazono)-2-phenylacetate (3e).

Prepared according to the general procedure A using 0.1130 g (0.36 mmol) of 4-bromostyrene (**1e**), 0.0806 g (0.3 mmol) of 4-methoxyphenyl 2-diazo-2-phenylacetate (**2b**), 45 μL (0.877 g/mL, 0.36 mmol) of $PhSiH_3$, 0.0029 g (0.015 mmol) of $Co(OAc)_2$, 0.0055 g (0.018 mmol) of **L4**, and 1.2 mL (0.25 M) of THF. After 12 h, the reaction was worked up. The crude mixture was purified by flash column chromatography using PE (50 mL) to PE/EA (50/1, 200 mL) as the eluent to give 0.1201 g (0.26 mmol, 88% yield) of the title compound as a light yellow oil. IR (neat): 3257, 2976, 1729, 1683, 1503 cm^{-1}. ^{1}H NMR: (400 MHz, $CDCl_3$) δ 10.86 (d, *J* = 4.4 Hz, 1H), 7.62 (d, *J* = 7.6

Hz, 2H), 7.48-7.44 (m, 2H), 7.38-7.32 (m, 2H), 7.31-7.25 (m, 1H), 7.24-7.17 (m, 2H), 7.04 (d, *J* = 8.8 Hz, 2H), 6.90 (d, *J* = 8.8 Hz, 2H), 4.82-4.74 (m, 1H), 3.78 (s, 3H), 1.60 (d, *J* = 6.8 Hz, 3H); ^{13}C NMR: (100 MHz, $CDCl_3$) δ 162.5, 157.4, 143.3, 142.0, 136.6, 131.7, 128.4, 128.3, 127.9, 127.2, 125.0, 122.4, 121.3, 114.5, 59.8, 55.6, 21.4; HRMS (ESI) calculated for $C_{23}H_{21}BrN_2NaO_3$ (M+Na$^+$) requires m/z 475.0633, found m/z 475.0630.

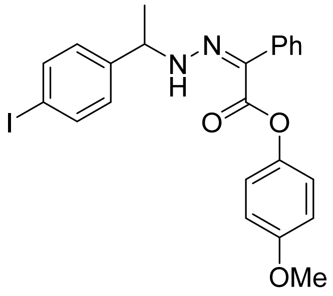

4-methoxyphenyl (*Z*)-2-(2-(1-(4-iodophenyl)ethyl)hydrazono)-2-phenylacetate (3f).

Prepared according to the general procedure A using 0.0827 g (0.36 mmol) of 4-iodostyrene (**1f**), 0.0805 g (0.3 mmol) of 4-methoxyphenyl 2-diazo-2-phenylacetate (**2b**), 45 μL (0.877 g/mL, 0.36 mmol) of $PhSiH_3$, 0.0029 g (0.015 mmol) of $Co(OAc)_2$, 0.0056 g (0.018 mmol) of **L3**, and 1.2 mL (0.25 M) of THF. After 12 h, the reaction was worked up. The crude mixture was purified by flash column chromatography using PE (50 mL) to PE/EA (20/1, 200 mL) as the eluent to give 0.1301 g (0.26 mmol, 87% yield) of the title compound as a light yellow oil. IR (neat): 3252, 2926, 1681, 1596, 1500, 1446 cm^{-1}. ^{1}H NMR: (400 MHz, $CDCl_3$) δ 10.86 (d, *J* = 4.0 Hz, 1H), 7.69-7.59 (m, 4H), 7.38-7.31 (m, 2H), 7.31-7.24 (m, 1H), 7.10-7.01 (m, 4H), 6.93-6.87 (d, *J* = 6.8 Hz, 2H), 4.80-4.72 (m, 1H), 3.78 (s, 3H), 1.59 (d, *J* = 6.8 Hz, 3H); ^{13}C NMR: (100 MHz, $CDCl_3$) δ 162.5, 157.4, 143.3, 142.7, 137.7, 136.6, 128.5, 128.4, 127.9, 127.1, 125.0, 122.4, 114.5, 92.9, 59.9, 55.6, 21.3; HRMS (ESI) calculated for $C_{23}H_{22}IN_2O_3$ (M+H$^+$) requires m/z 501.0675, found m/z 501.0674.

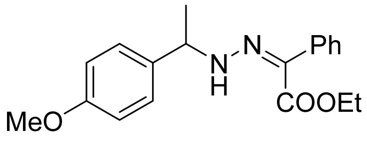

Ethyl (*Z*)-2-(2-(1-(4-methoxyphenyl)ethyl)hydrazono)-2-phenylacetate (3g).

Prepared according to the general procedure A using 0.0484 g (0.36 mmol) of 4-methoxystyrene (**1g**), 48 μL (1.165 g/ml, 0.3 mmol) of ethyl 2-diazo-2-phenylacetate (**2a**), 45 μL (0.877 g/mL, 0.36 mmol) of $PhSiH_3$, 0.0028 g (0.015 mmol) of $Co(OAc)_2$,

0.0057 g (0.018 mmol) of **L4**, and 1.2 mL (0.25 M) of THF. After 12 h, the reaction was worked up. The crude mixture was purified by flash column chromatography using PE (50 mL) to PE/EA (50/1, 200 mL) as the eluent to give 0.0872 g (0.27 mmol, 89% yield) of the title compound as a light yellow oil. IR (neat): 3249, 2979, 2836, 1669, 1611, 1513 cm^{-1}. ^{1}H NMR: (400 MHz, $CDCl_3$) δ 10.72 (br, 1H), 7.52 (d, J = 7.6 Hz, 2H), 7.32-7.21 (m, 5H), 6.88 (d, J = 8.4 Hz, 2H), 4.78-4.71 (m, 1H), 4.25 (q, J = 7.2 Hz, 2H), 3.79 (s, 3H), 1.62 (d, J = 6.8 Hz, 3H), 1.30 (t, J = 7.2 Hz, 3H); ^{13}C NMR: (100 MHz, $CDCl_3$) δ 163.6, 158.9, 137.2, 135.3, 128.2, 127.7, 126.7, 125.4, 114.0, 60.3, 59.6, 55.2, 21.6, 14.2; HRMS (ESI) calculated for $C_{19}H_{23}N_2O_3$ (M+H$^+$) requires m/z 327.1709, found m/z 327.1707.

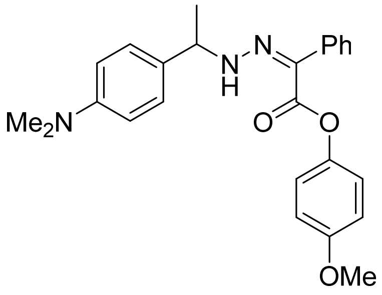

4-methoxyphenyl (*Z*)-2-(2-(1-(4-(dimethylamino)phenyl)ethyl)hydrazono)-2-phenylacetate (3h).

Prepared according to the general procedure A using 0.0532 g (0.36 mmol) of *N*,*N*-dimethyl-4-vinylaniline (**1h**), 0.0806 g (0.3 mmol) of 4-methoxyphenyl 2-diazo-2-phenylacetate (**2b**), 45 μL (0.877 g/mL, 0.36 mmol) of $PhSiH_3$, 0.0029 g (0.015 mmol) of $Co(OAc)_2$, 0.0058 g (0.018 mmol) of **L4**, and 1.2 mL (0.25 M) of THF. After 12 h, the reaction was worked up. The crude mixture was purified by flash column chromatography using PE (50 mL) to PE/EA (15/1, 200 mL) to give 0.1037 g (0.25 mmol, 83% yield) of the title compound as a light yellow oil. IR (neat): 3249, 2971, 1678, 1613, 1499, 1445 cm^{-1}. ^{1}H NMR: (400 MHz, $CDCl_3$) δ 10.87 (d, J = 4.8 Hz, 1H), 7.66 (d, J = 7.6 Hz, 2H), 7.38-7.31 (m, 2H), 7.29-7.23 (m, 1H), 7.23-7.16 (m, 2H), 7.02 (d, J = 9.2 Hz, 2H), 6.87 (d, J = 8.8 Hz, 2H), 6.69 (d, J = 8.8 Hz, 2H), 4.79-4.71 (m, 1H), 3.74 (s, 3H), 2.90 (s, 6H), 1.62 (d, J = 6.8 Hz, 3H); ^{13}C NMR: (100 MHz, $CDCl_3$) δ 162.4, 157.3, 150.1, 143.5, 137.0, 130.1, 128.4, 127.8, 127.4, 126.8, 123.6, 122.5, 114.4, 112.6, 60.1, 55.5, 40.5, 21.4; HRMS (ESI) calculated for $C_{25}H_{27}N_3NaO_3$ (M+Na$^+$) requires m/z 440.1950, found m/z 440.1951.

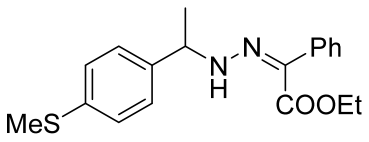

Ethyl (*Z*)-2-(2-(1-(4-(methylthio)phenyl)ethyl)hydrazono)-2-phenylacetate (3i).

Prepared according to the general procedure A using 0.0541 g (0.36 mmol) of methyl(4-vinylphenyl)sulfane (**1i**), 48 μL (1.165 g/ml, 0.3 mmol) of ethyl 2-diazo-2-phenylacetate (**2a**), 45 μL (0.877 g/mL, 0.36 mmol) of $PhSiH_3$, 0.0029 g (0.015 mmol) of $Co(OAc)_2$, 0.0058 g (0.018 mmol) of **L4**, and 1.2 mL (0.25 M) of THF. After 12 h, the reaction was worked up. The crude mixture was purified by flash column chromatography using PE (50 mL) to PE/EA (100/1, 200 mL) as the eluent to give 0.0815 g (0.24 mmol, 79% yield) of the title compound as a light yellow oil. IR (neat): 3247, 2979, 2924, 1734, 1669, 1513 cm^{-1}. ^{1}H NMR: (400 MHz, $CDCl_3$) δ 10.73 (d, J = 4.8 Hz, 1H), 7.54-7.49 (m, 2H), 7.33-7.20 (m, 7H), 4.79-4.71 (m, 1H), 4.29-4.22 (m, 2H), 2.46 (s, 3H), 1.61 (d, J = 7.2 Hz, 3H), 1.30 (t, J = 7.2 Hz, 3H); ^{13}C NMR: (100 MHz, $CDCl_3$) δ 163.6, 140.3, 137.4, 137.1, 128.2, 127.7, 127.0, 126.9, 126.8, 125.8, 60.3, 59.7, 21.5, 15.9, 14.2; HRMS (ESI) calculated for $C_{19}H_{22}N_2NaO_2S$ (M+Na$^+$) requires m/z 365.1300, found m/z 365.1301.

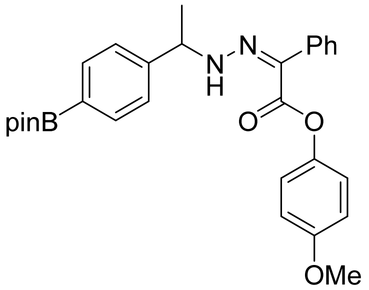

4-methoxyphenyl (Z)-2-phenyl-2-(2-(1-(4-(4,4,5,5-tetramethyl-1,3,2-dioxaborolan-2-yl)phenyl)ethyl)hydrazono)acetate (3j).

Prepared according to the general procedure A using 0.1132 g (0.36 mmol) of 4,4,5,5-tetramethyl-2-(4-vinylphenyl)-1,3,2-dioxaborolane (**1j**), 0.0805 g (0.3 mmol) of 4-methoxyphenyl 2-diazo-2-phenylacetate (**2b**), 45 μL (0.877 g/mL, 0.36 mmol) of $PhSiH_3$, 0.0027 g (0.015 mmol) of $Co(OAc)_2$, 0.0057 g (0.018 mmol) of **L4**, and 1.2 mL (0.25 M) of THF. After 12 h, the reaction was worked up. The crude mixture was purified by flash column chromatography using PE (50 mL) to PE/EA (50/1, 200 mL) to give 0.0758 g (0.15 mmol, 50% yield) of the title compound as a light yellow oil. IR (neat): 2955, 2924, 2089, 1722, 1601, 1503, 1461 cm^{-1}. ^{1}H NMR: (400 MHz, $CDCl_3$) δ 10.93 (d, J = 4.8 Hz, 1H), 7.80 (d, J = 7.2 Hz, 2H), 7.62 (d, J = 7.2 Hz, 2H), 7.37-7.31 (m, 4H), 7.29-

7.24 (m, 1H), 7.04 (d, *J* = 8.8 Hz, 2H), 6.90 (d, *J* = 8.8 Hz, 2H), 4.88-4.80 (m, 1H), 3.78 (s, 3H), 1.63 (d, *J* = 6.8 Hz, 3H), 1.33 (s, 12H); ^{13}C NMR: (100 MHz, $CDCl_3$) δ 162.5, 157.4, 146.1, 143.4, 136.8, 135.2, 128.4, 127.8, 127.0, 125.8, 124.6, 122.4, 114.5, 83.7, 60.6, 55.6, 24.8, 24.8, 21.5; HRMS (ESI) calculated for $C_{29}H_{34}BN_2O_5$ (M+H$^+$) requires m/z 501.2561, found m/z 501.2563.

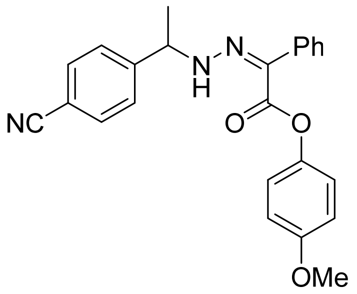

4-methoxyphenyl (*Z*)-2-(2-(1-(4-cyanophenyl)ethyl)hydrazono)-2-phenylacetate (3k). Prepared according to the general procedure A using 0.0466 g (0.36 mmol) of 4-vinylbenzonitrile (**1k**), 0.0806 g (0.3 mmol) of 4-methoxyphenyl 2-diazo-2-phenylacetate (**2b**), 45 µL (0.877 g/mL, 0.36 mmol) of $PhSiH_3$, 0.0028 g (0.015 mmol) of $Co(OAc)_2$, 0.0059 g (0.018 mmol) of **L4**, and 1.2 mL (0.25 M) of THF. After 12 h, the reaction was worked up. The crude mixture was purified by flash column chromatography using PE (50 mL) to PE/EA (20/1, 200 mL) to give 0.0827 g (0.21 mmol, 69% yield) of the title compound as a light yellow oil. IR (neat): 3258, 2929, 2229, 1684, 1605, 1502 cm^{-1}. ^{1}H NMR: (400 MHz, $CDCl_3$) δ 10.84 (d, *J* = 4.0 Hz, 1H), 7.65-7.55 (m, 4H), 7.20 (d, *J* = 8.0 Hz, 2H), 7.37-7.31 (m, 2H), 7.31-7.25 (m, 1H), 7.05 (d, *J* = 8.8 Hz, 2H), 6.90 (d, *J* = 8.8 Hz, 2H), 4.90-4.82 (m, 1H), 3.78 (s, 3H), 1.61 (d, *J* = 7.2 Hz, 3H); ^{13}C NMR: (100 MHz, $CDCl_3$) δ 162.4, 157.5, 148.7, 143.3, 136.4, 132.5, 128.3, 127.9, 127.32, 127.26, 125.9, 122.3, 118.6, 114.5, 111.3, 59.9, 55.6, 21.2; HRMS (ESI) calculated for $C_{24}H_{22}N_3O_3$ (M+H$^+$) requires m/z 400.1661, found m/z 400.1663.

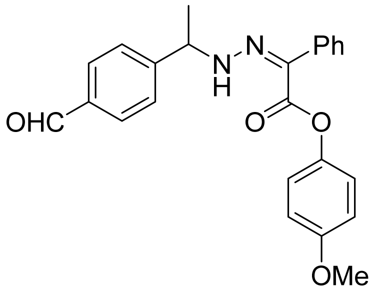

4-methoxyphenyl (*Z*)-2-(2-(1-(4-formylphenyl)ethyl)hydrazono)-2-phenylacetate (3l). Prepared according to the general procedure A using 0.0715 g (0.36 mmol) of 4-vinylbenzaldehyde (**1l**), 0.0804 g (0.3 mmol) of 4-methoxyphenyl 2-diazo-2-phenylacetate (**2b**), 45 µL (0.877 g/mL, 0.36 mmol) of $PhSiH_3$, 0.0028 g (0.015 mmol)

of $Co(OAc)_2$, 0.0059 g (0.018 mmol) of **L4**, and 1.2 mL (0.25 M) of THF. After 12 h, the reaction was worked up. The crude mixture was purified by flash column chromatography using PE (50 mL) to PE/EA (8/1, 200 mL) as the eluent to give 0.1015 g (0.25 mmol, 84% yield) of the title compound as a light yellow oil. IR (neat): 3254, 2957, 2926, 1700, 1606, 1503, 1460 cm^{-1}. ^1H NMR: (400 MHz, $CDCl_3$) δ 10.90 (d, J = 4.4 Hz, 1H), 10.00 (s, 1H), 7.87 (d, J = 8.0 Hz, 2H), 7.61 (d, J = 7.6 Hz, 2H), 7.50 (d, J = 8.0 Hz, 2H), 7.38-7.31 (m, 2H), 7.31-7.26 (m, 1H), 7.06 (d, J = 8.8 Hz, 2H), 6.91 (d, J = 8.8 Hz, 2H), 4.95-4.86 (m, 1H), 3.80 (s, 3H), 1.65 (d, J = 7.2 Hz, 3H); ^{13}C NMR: (100 MHz, $CDCl_3$) δ 191.8, 162.5, 157.5, 150.1, 143.3, 136.5, 135.6, 130.2, 128.4, 127.9, 127.3, 127.1, 125.5, 122.4, 114.5, 60.2, 55.6, 21.3; HRMS (ESI) calculated for $C_{24}H_{23}N_2O_4$ ($M+H^+$) requires m/z 403.1658, found m/z 403.1656.

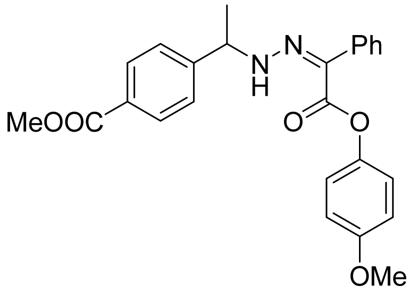

Methyl (*Z*)-4-(1-(2-(2-(4-methoxyphenoxy)-2-oxo-1-phenylethylidene)hydrazinyl)ethyl)benzoate (3m).

Prepared according to the general procedure A using 0.0969 g (0.36 mmol) of methyl 4-vinylbenzoate (**1m**), 0.0806 g (0.3 mmol) of 4-methoxyphenyl 2-diazo-2-phenylacetate (**2b**), 45 μL (0.877 g/mL, 0.36 mmol) of $PhSiH_3$, 0.0027 g (0.015 mmol) of $Co(OAc)_2$, 0.0060 g (0.018 mmol) of **L4**, and 1.2 mL (0.25 M) of THF. After 12 h, the reaction was worked up. The crude mixture was purified by flash column chromatography using PE (50 mL) to PE/EA (10/1, 200 mL) as the eluent to give 0.1193 g (0.28 mmol, 92% yield) of the title compound as a light yellow oil. M.p.: 110.1-111.2 °C. IR (neat): 3255, 2953, 1721, 1683, 1610, 1502, 1438 cm^{-1}. ^1H NMR: (400 MHz, $CDCl_3$) δ 10.90 (d, J = 4.8 Hz, 1H), 8.02 (d, J = 8.0 Hz, 2H), 7.61 (d, J = 7.2 Hz, 2H), 7.40 (d, J = 8.4 Hz, 2H), 7.37-7.31 (m, 2H), 7.31-7.25 (m, 1H), 7.05 (d, J = 9.2 Hz, 2H), 6.90 (d, J = 9.2 Hz, 2H), 4.98-4.84 (m, 1H), 3.90 (s, 3H), 3.79 (s, 3H), 1.64 (d, J = 7.2 Hz, 3H); ^{13}C NMR: (100 MHz, $CDCl_3$) δ 166.8, 162.5, 157.4, 148.2, 143.3, 136.6, 130.0, 129.3, 128.4, 127.9, 127.2, 126.5, 125.3, 122.4, 114.5, 60.2, 55.6, 52.0, 21.4; HRMS (ESI) calculated for $C_{25}H_{25}N_2O_5$ ($M+H^+$) requires m/z 433.1763, found m/z 433.1761.

X-ray Diffraction of 3m, CCDC 1944446

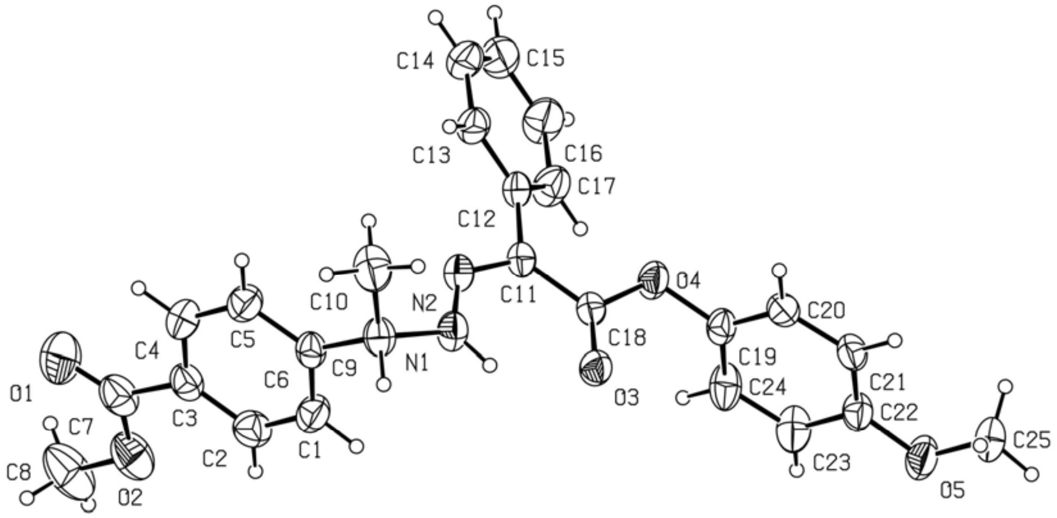

Supplementary Figure 187

Bond precision:	C-C = 0.0037 A	Wavelength=0.71073	
Cell:	a=6.4694(3)	b=12.0243(5)	c=15.2986(6)
	alpha=107.002(1)	beta=90.604(1)	gamma=95.440(2)
Temperature: 296 K			
	Calculated	Reported	
Volume	1132.02(8)	1132.02(8)	
Space group	P -1	P -1	
Hall group	-P 1	-P 1	
Moiety formula	C25 H24 N2 O5	C25 H24 N2 O5	
Sum formula	C25 H24 N2 O5	C25 H24 N2 O5	
Mr	432.46	432.46	
Dx,g cm-3	1.269	1.269	
Z	2	2	
Mu (mm-1)	0.089	0.089	
F000	456.0	456.0	
F000’	456.23		
h,k,lmax	8,15,19	8,14,19	
Nref	4616	4579	
Tmin,Tmax	0.976,0.982	0.694,0.745	

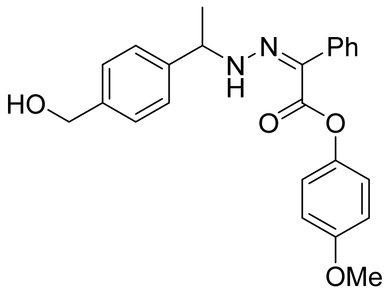

4-methoxyphenyl (*Z*)-2-(2-(1-(4-(hydroxymethyl)phenyl)ethyl)hydrazono)-2-phenylacetate (3n).

Prepared according to the general procedure A using 0.0485 g (0.36 mmol) of (4-vinylphenyl)methanol (**1n**), 0.0805 g (0.3 mmol) of 4-methoxyphenyl 2-diazo-2-phenylacetate (**2b**), 45 μL (0.877 g/mL, 0.36 mmol) of $PhSiH_3$, 0.0028 g (0.015 mmol) of $Co(OAc)_2$, 0.0059 g (0.018 mmol) of **L4**, and 1.2 mL (0.25 M) of THF. After 12 h, the reaction was worked up. The crude mixture was purified by flash column chromatography using PE (50 mL) to PE/EA (5/1, 600 mL) as the eluent to give 0.0924 g (0.23 mmol, 76% yield) of the title compound as a light yellow oil. IR (neat): 3256, 2957, 2926, 1680, 1600, 1502, 1462 cm^{-1}. ^1H NMR: (400 MHz, $CDCl_3$) δ 10.90 (d, J = 4.8 Hz, 1H), 7.63 (d, J = 7.2 Hz, 2H), 7.38-7.32 (m, 6H), 7.30-7.26 (m, 1H), 7.04 (d, J = 8.8 Hz, 2H), 6.90 (d, J = 8.8 Hz, 2H), 4.88-4.80 (m, 1H), 4.67 (s, 2H), 3.79 (s, 3H), 1.70 (br, 1H), 1.64 (d, J = 6.4 Hz, 3H); ^{13}C NMR: (100 MHz, $CDCl_3$) δ 162.5, 157.4, 143.4, 142.3, 140.2, 136.8, 128.4, 127.9, 127.3, 127.0, 126.7, 124.6, 122.4, 114.5, 64.9, 60.3, 55.6, 21.5; HRMS (ESI) calculated for $C_{24}H_{24}N_2NaO_4$ ($M+Na^+$) requires m/z 427.1634, found m/z 427.1631.

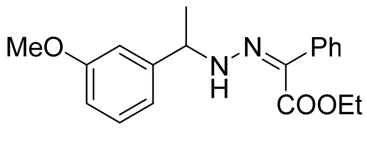

Ethyl (*Z*)-2-(2-(1-(3-methoxyphenyl)ethyl)hydrazono)-2-phenylacetate (3o).

Prepared according to the general procedure A using 0.0484 g (0.36 mmol) of 3-methoxystyrene (**1o**), 48 μL (1.165 g/ml, 0.3 mmol) of ethyl 2-diazo-2-phenylacetate (**2a**), 45 μL (0.877 g/mL, 0.36 mmol) of $PhSiH_3$, 0.0026 g (0.015 mmol) of $Co(OAc)_2$, 0.0058 g (0.018 mmol) of **L4**, and 1.2 mL (0.25 M) of THF. After 12 h, the reaction was worked up. The crude mixture was purified by flash column chromatography using PE (50 mL) to PE/EA (30/1, 200 mL) as the eluent to give 0.0930 g (0.29 mmol, 95% yield) of the title compound as a light yellow oil. IR (neat): 3250, 2927, 1669, 1600, 1514 cm^{-1}. ^1H NMR: (400 MHz, $CDCl_3$) δ 10.77 (d, J = 4.4 Hz, 1H), 7.52 (d, J = 7.2

Hz, 2H), 7.34-7.22 (m, 4H), 6.93 (d, *J* = 7.6 Hz, 1H), 6.91-6.87 (m, 1H), 6.81 (dd, *J* = 8.0, 2.0 Hz, 1H), 4.81-8.71 (m, 1H), 4.26 (q, *J* = 7.2 Hz, 2H), 3.79 (s, 3H), 1.63 (d, *J* = 7.2 Hz, 3H), 1.30 (t, *J* = 7.2 Hz, 3H); ^{13}C NMR: (100 MHz, $CDCl_3$) δ 163.6, 159.8, 145.0, 137.1, 129.7, 128.2, 127.7, 126.8, 125.7, 118.8, 112.5, 112.4, 60.3, 60.2, 55.2, 21.6, 14.2; HRMS (ESI) calculated for $C_{19}H_{23}N_2O_3$ (M+H$^+$) requires m/z 327.1703, found m/z 327.1702.

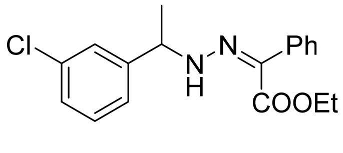

Ethyl (*Z*)-2-(2-(1-(3-chlorophenyl)ethyl)hydrazono)-2-phenylacetate (3p).

Prepared according to the general procedure A using 0.0490 g (0.36 mmol) of 3-chlorostyrene (**1p**), 48 µL (1.165 g/ml, 0.3 mmol) of ethyl 2-diazo-2-phenylacetate (**2a**), 45 µL (0.877 g/mL, 0.36 mmol) of $PhSiH_3$, 0.0026 g (0.015 mmol) of $Co(OAc)_2$, 0.0061 g (0.019 mmol) of **L4**, and 1.2 mL (0.25 M) of THF. After 12 h, the reaction was worked up. The crude mixture was purified by flash column chromatography using PE (50 mL) to PE/EA (70/1, 200 mL) as the eluent to give 0.0933 g (0.28 mmol, 95% yield) of the title compound as a light yellow oil. IR (neat): 3245, 2926, 1674, 1595, 1517 cm^{-1}. ^{1}H NMR: (400 MHz, $CDCl_3$) δ 10.85 (d, *J* = 3.2 Hz, 1H), 7.50 (d, *J* = 7.2 Hz, 2H), 7.40-7.33 (m, 2H), 7.33-7.26 (m, 3H), 7.24-7.16 (m, 2H), 5.27-5.18 (m, 1H), 4.29 (q, *J* = 7.2 Hz, 2H), 1.63 (d, *J* = 6.8 Hz, 3H), 1.33 (t, *J* = 7.2 Hz, 3H); ^{13}C NMR: (100 MHz, $CDCl_3$) δ 163.6, 145.7, 137.0, 134.4, 129.9, 128.3, 127.7, 127.5, 127.0, 126.8, 126.4, 124.7 , 60.5, 59.7, 21.5, 14.2; HRMS (ESI) calculated for $C_{18}H_{20}ClN_2O_2$ (M+H$^+$) requires m/z 331.1208, found m/z 331.1209.

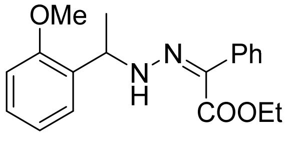

Ethyl (*Z*)-2-(2-(1-(2-methoxyphenyl)ethyl)hydrazono)-2-phenylacetate (3q).

Prepared according to the general procedure A using 0.0487 g (0.36 mmol) of 2-methoxystyrene (**1q**), 48 µL (1.165 g/ml, 0.3 mmol) of ethyl 2-diazo-2-phenylacetate (**2a**), 45 µL (0.877 g/mL, 0.36 mmol) of $PhSiH_3$, 0.0027 g (0.015 mmol) of $Co(OAc)_2$, 0.0056 g (0.018 mmol) of **L4**, and 1.2 mL (0.25 M) of THF. After 12 h, the reaction was worked up. The crude mixture was purified by flash column

chromatography using PE (50 mL) to PE/EA (50/1, 200 mL) as the eluent to give 0.0891 g (0.27 mmol, 91% yield) of the title compound as a light yellow oil. IR (neat): 3259, 2924, 1670, 1600, 1513, 1462 cm^{-1}. ^{1}H NMR: (400 MHz, $CDCl_3$) δ 11.00 (br, 1H), 7.51 (d, J = 7.6 Hz, 2H), 7.34-7.17 (m, 5H), 6.98-6.84 (m, 2H), 5.15-5.03 (m, 1H), 4.27 (q, J = 7.2 Hz, 2H), 3.87 (s, 3H), 1.60 (d, J = 6.8 Hz, 3H), 1.31 (t, J = 7.2 Hz, 3H); ^{13}C NMR: (100 MHz, $CDCl_3$) δ 163.6, 156.6, 137.4, 131.6, 128.3, 127.6, 127.1, 126.6, 125.1, 120.7, 110.7, 60.1, 56.7, 55.3, 20.7, 14.2; HRMS (ESI) calculated for $C_{19}H_{22}N_2NaO_3$ (M+Na$^+$) requires m/z 349.1523, found m/z 349.1526.

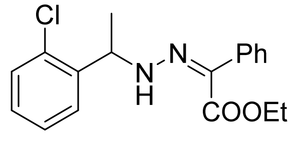

Ethyl (*Z*)-2-(2-(1-(2-chlorophenyl)ethyl)hydrazono)-2-phenylacetate (3r).

Prepared according to the general procedure A using 0.0502 g (0.36 mmol) of 2-chlorostyrene (**1r**), 48 μL (1.165 g/ml, 0.3 mmol) of ethyl 2-diazo-2-phenylacetate (**2a**), 45 μL (0.877 g/mL, 0.36 mmol) of $PhSiH_3$, 0.0027 g (0.015 mmol) of $Co(OAc)_2$, 0.0061 g (0.018 mmol) of **L4**, and 1.2 mL (0.25 M) of THF. After 12 h, the reaction was worked up. The crude mixture was purified by flash column chromatography using PE (50 mL) to PE/EA (80/1, 200 mL) as the eluent to give 0.089 g (0.27 mmol, 90% yield) of the title compound as a light yellow oil. IR (neat): 3248, 2927, 1673, 1517, 1444 cm^{-1}. ^{1}H NMR: (400 MHz, $CDCl_3$) δ 10.71 (d, J = 3.2 Hz, 1H), 7.50 (d, J = 7.6 Hz, 2H), 7.36-7.27 (m, 4H), 7.26-7.18 (m, 3H), 4.81-4.71 (m, 1H), 4.28 (q, J = 7.2 Hz, 2H), 1.62 (d, J = 6.4 Hz, 3H), 1.32 (t, J = 7.2 Hz, 3H); ^{13}C NMR: (100 MHz, $CDCl_3$) δ 163.6, 141.3, 137.0, 132.7, 129.8, 128.32, 128.27, 127.7, 127.4, 127.2, 126.9, 126.3, 60.4, 57.0, 20.3, 14.2; HRMS (ESI) calculated for $C_{18}H_{19}ClN_2NaO_2$ (M+Na$^+$) requires m/z 353.1027, found m/z 353.1028.

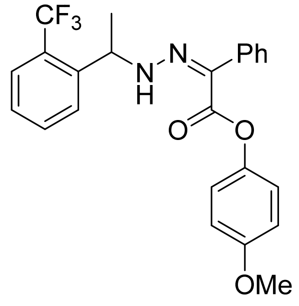

4-methoxyphenyl (*Z*)-2-phenyl-2-(2-(1-(2-(trifluoromethyl)phenyl)ethyl)hydrazono)acetate (3s).

Prepared according to the general procedure A using 0.0621 g (0.36 mmol) of 2-(trifluoromethyl)styrene (**1s**), 0.0801 g (0.3 mmol) of 4-methoxyphenyl 2-diazo-2-phenylacetate (**2b**), 45

μL (0.877 g/mL, 0.36 mmol) of $PhSiH_3$, 0.0029 g (0.015 mmol) of $Co(OAc)_2$, 0.0060 g (0.018 mmol) of **L4**, and 1.2 mL (0.25 M) of THF. After 12 h, the reaction was worked up. The crude mixture was purified by flash column chromatography using PE (50 mL) to PE/EA (20/1, 200 mL) as the eluent to give 0.1234 g (0.28 mmol, 93% yield) of the title compound as a light yellow oil. IR (neat): 3260, 2957, 1685, 1604, 1503, 1457 cm^{-1}. ^{1}H NMR: (400 MHz, $CDCl_3$) δ 10.95 (d, J = 3.6 Hz, 1H), 7.67-7.61 (m, 3H), 7.61-7.51 (m, 2H), 7.38-7.31 (m, 3H), 7.31-7.24 (m, 1H), 7.05 (d, J = 9.2 Hz, 2H), 6.90 (d, J = 9.2 Hz, 2H), 5.31-5.21 (m, 1H), 3.79 (s, 3H), 1.65 (d, J = 6.8 Hz, 3H); ^{13}C NMR: (100 MHz, $CDCl_3$) δ 162.5, 157.5, 143.4, 142.5, 136.7, 132.4, 128.3, 127.9, 127.8, 127.4, 127.3, 127.1, 125.7 (q, J = 5.6 Hz), 125.1, 122.4, 114.5, 55.7, 55.6, 22.1; ^{19}F NMR: (376 MHz, $CDCl_3$) δ -58.2; HRMS (ESI) calculated for $C_{24}H_{22}F_3N_2O_3$ (M+H$^+$) requires m/z 443.1583, found m/z 443.1586.

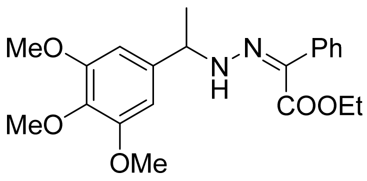

Ethyl (*Z*)-2-phenyl-2-(2-(1-(3,4,5-trimethoxyphenyl)ethyl)hydrazono)acetate (3t). Prepared according to the general procedure A using 0.0700 g (0.36 mmol) of 1,2,3-trimethoxy-5-vinylbenzene (**1t**), 48 μL (1.165 g/ml, 0.3 mmol) of ethyl 2-diazo-2-phenylacetate (**2a**), 45 μL (0.877 g/mL, 0.36 mmol) of $PhSiH_3$, 0.0026 g (0.015 mmol) of $Co(OAc)_2$, 0.0056 g (0.018 mmol) of **L3**, and 1.2 mL (0.25 M) of THF. After 12 h, the reaction was worked up. The crude mixture was purified by flash column chromatography using PE (50 mL) to PE/EA (10/1, 200 mL) as the eluent to give 0. 0834 g (0.22 mmol, 72% yield) of the title compound as a light yellow oil. IR (neat): 3250, 2980, 1736, 1671, 1592, 1508 cm^{-1}. ^{1}H NMR: (400 MHz, $CDCl_3$) δ 10.72 (d, J = 4.4 Hz, 1H), 7.53 (d, J = 7.2 Hz, 2H), 7.34-7.27 (m, 2H), 7.27-7.21 (m, 1H), 6.57 (s, 2H), 4.75-4.66 (m, 1H), 4.27 (q, J = 7.2 Hz, 2H), 3.86 (s, 6H), 3.84 (s, 3H), 1.63 (d, J = 6.8 Hz, 3H), 1.30 (t, J = 7.2 Hz, 3H); ^{13}C NMR: (100 MHz, $CDCl_3$) δ 163.5, 153.2, 138.9, 137.1, 137.0, 128.1, 127.6, 126.8, 125.9, 103.5, 60.7, 60.3, 56.0, 21.4, 14.1; HRMS (ESI) calculated for $C_{21}H_{27}N_2O_5$ (M+H$^+$) requires m/z 387.1914, found m/z 387.1902.

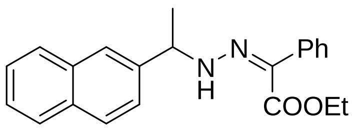

Ethyl (*Z*)-2-(2-(1-(naphthalen-2-yl)ethyl)hydrazono)-2-phenylacetate (3u).

Prepared according to the general procedure A using 0.0555 g (0.36 mmol) of 2-vinylnaphthalene (**1u**), 48 μL (1.165 g/ml, 0.3 mmol) of ethyl 2-diazo-2-phenylacetate (**2a**), 45 μL (0.877 g/mL, 0.36 mmol) of $PhSiH_3$, 0.0027 g (0.015 mmol) of $Co(OAc)_2$, 0.0056 g (0.018 mmol) of **L4**, and 1.2 mL (0.25 M) of THF. After 12 h, the reaction was worked up. The crude mixture was purified by flash column chromatography using PE (50 mL) to PE/EA (30/1, 200 mL) as the eluent to give 0.0956 g (0.28 mmol, 92% yield) of the title compound as a light yellow oil. IR (neat): 3248, 2977, 1670, 1600, 1513, 1447 cm^{-1}. ^{1}H NMR: (400 MHz, $CDCl_3$) δ 10.87 (d, *J* = 4.8 Hz, 1H), 7.86-7.79 (m, 3H), 7.77 (s, 1H), 7.55-7.50 (m, 2H), 7.50-7.43 (m, 3H), 7.34-7.26 (m, 2H), 7.26-7.20 (m, 1H), 5.00-4.90 (m, 1H), 4.26 (qd, *J* = 7.2, 1.2 Hz, 2H), 1.72 (d, *J* = 6.8 Hz, 3H), 1.30 (t, *J* = 7.2 Hz, 3H); ^{13}C NMR: (100 MHz, $CDCl_3$) δ 163.7, 140.8, 137.1, 133.4, 132.8, 128.5, 128.3, 127.9, 127.7, 127.6, 126.8, 126.1, 125.9, 125.8, 125.1, 124.9, 60.4, 60.3, 21.5, 14.2; HRMS (ESI) calculated for $C_{22}H_{23}N_2O_2$ (M+H$^+$) m/z 347.1754, found m/z 347.1762.

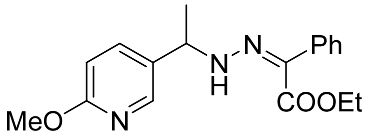

Ethyl (*Z*)-2-(2-(1-(6-methoxypyridin-3-yl)ethyl)hydrazono)-2-phenylacetate (3v).

Prepared according to the general procedure A using 0.0487 g (0.36 mmol) of 2-methoxy-5-vinylpyridine (**1v**), 48 μL (1.165 g/ml, 0.3 mmol) of ethyl 2-diazo-2-phenylacetate (**2a**), 45 μL (0.877 g/mL, 0.36 mmol) of $PhSiH_3$, 0.0027 g (0.015 mmol) of $Co(OAc)_2$, 0.0055 g (0.018 mmol) of **L3**, and 1.2 mL (0.25 M) of THF. After 12 h, the reaction was worked up. The crude mixture was purified by flash column chromatography using PE (50 mL) to PE/EA (100/1, 200 mL) as the eluent to give 0.0701 g (0.21 mmol, 71% yield) of the title compound as a light yellow oil. IR (neat): 3255, 2927, 1672, 1609, 1495 cm^{-1}. ^{1}H NMR: (400 MHz, $CDCl_3$) δ 10.66 (d, *J* = 3.6 Hz, 1H), 8.14 (d, *J* = 1.6 Hz, 1H), 7.57 (dd, *J* = 8.4, 2.0, Hz, 1H), 7.50 (d, *J* = 7.6 Hz, 2H), 7.34-7.27 (m, 2H), 7.27-7.21 (m, 1H), 6.73 (d, *J* = 8.4 Hz, 1H), 4.79-4.71 (m, 1H), 4.26 (q, *J* = 7.2 Hz, 2H), 3.92 (s, 3H), 1.62 (d, *J* = 6.8 Hz, 3H), 1.30 (t, *J*

= 7.2 Hz, 3H); ^{13}C NMR: (100 MHz, $CDCl_3$) δ 163.6, 163.5, 145.1, 137.1, 136.9, 131.5, 128.2, 127.7, 126.9, 126.3, 110.9, 60.4, 57.2, 53.4, 21.1, 14.1; HRMS (ESI) calculated for $C_{18}H_{21}N_3NaO_3$ (M+Na$^+$) requires m/z 350.1475, found m/z 350.1485

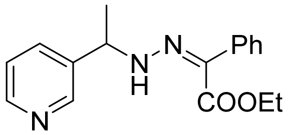

Ethyl (*Z*)-2-phenyl-2-(2-(1-(pyridin-3-yl)ethyl)hydrazono)acetate (3w).

Prepared according to the general procedure A using 0.0379 g (0.36 mmol) of 3-vinylpyridine (**1w**), 48 µL (1.165 g/ml, 0.3 mmol) of ethyl 2-diazo-2-phenylacetate (**2a**), 45 µL (0.877 g/mL, 0.36 mmol) of $PhSiH_3$, 0.0028 g (0.015 mmol) of $Co(OAc)_2$, 0.0060 g (0.018 mmol) of **L4**, and 1.2 mL (0.25 M) of THF. After 12 h, the reaction was worked up. The crude mixture was purified by flash column chromatography using PE (50 mL) to PE/EA (3/1, 200 mL) as the eluent to give 0.0807 g (0.28 mmol, 92% yield) of the title compound as a light yellow oil. IR (neat): 3252, 2929, 1672, 1578, 1516 cm^{-1}. ^{1}H NMR: (400 MHz, $CDCl_3$) δ 10.71 (d, *J* = 4.0 Hz, 1H), 8.61 (d, *J* = 1.6 Hz, 1H), 8.53 (dd, *J* = 4.4, 1.2 Hz, 1H), 7.70-7.62 (m, 1H), 7.52-7.45 (m, 2H), 7.35-7.21 (m, 4H), 4.87-4.77 (m, 1H), 4.28 (q, *J* = 7.2 Hz, 2H), 1.65 (d, *J* = 6.8 Hz, 3H), 1.31 (t, *J* = 7.2 Hz, 3H); ^{13}C NMR: (100 MHz, $CDCl_3$) δ 163.5, 148.7, 148.5, 138.9, 136.8, 134.0, 128.2, 127.7, 127.0, 126.9, 123.5, 60.5, 57.7, 21.2, 14.1; HRMS (ESI) calculated for $C_{17}H_{20}N_3O_2$ (M+H$^+$) requires m/z 298.1550, found m/z 298.1563.

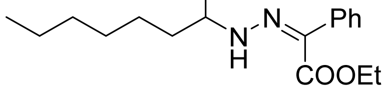

Ethyl (*Z*)-2-(2-(octan-2-yl)hydrazono)-2-phenylacetate (3x).

Prepared according to the general procedure A using 0.0679 g (0.6 mmol) of 1-octene (**1x**), 48 µL (1.165 g/ml, 0.3 mmol) of ethyl 2-diazo-2-phenylacetate (**2a**), 45 µL (0.877 g/mL, 0.36 mmol) of $PhSiH_3$, 0.0027 g (0.015 mmol) of $Co(OAc)_2$, 0.0057 g (0.018 mmol) of **L4**, and 1.2 mL (0.25 M) of THF. After 12 h, the reaction was worked up. The crude mixture was purified by flash column chromatography using PE (50 mL) to PE/EA (100/1, 200 mL) as the eluent to give 0.0703 g (0.23 mmol, 77% yield) of the title compound as a light yellow oil. IR (neat): 3249, 2927, 1669, 1514, 1459 cm^{-1}. ^{1}H

NMR: (400 MHz, $CDCl_3$) δ 10.59 (d, J = 3.6 Hz, 1H), 7.54 (d, J = 7.2 Hz, 2H), 7.36-7.26 (m, 2H), 7.25-7.19 (m, 1H), 4.27 (q, J = 7.2 Hz, 2H), 3.69-3.55 (m, 1H), 1.74-1.60 (m, 1H), 1.55-1.45 (m, 1H), 1.44-1.20 (m, 14H), 0.880 (t, J = 6.4 Hz, 3H); ^{13}C NMR: (100 MHz, $CDCl_3$) δ 163.8, 137.5, 128.2, 127.7, 126.5, 124.3, 60.1, 56.6, 36.4, 31.7, 29.2, 25.9, 22.6, 20.4, 14.2, 14.0; HRMS (ESI) calculated for $C_{18}H_{29}N_2O_2$ (M+H$^+$) requires m/z 305.2224, found m/z 305.2238.

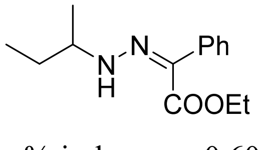

Ethyl (*Z*)-2-(2-(*sec*-butyl)hydrazono)-2-phenylacetate (3y). Prepared according to the general procedure A using 0.3370 g (10 w% in hexane, 0.60 mmol) of butene (**1y**), 48 μL (1.165 g/ml, 0.3 mmol) of ethyl 2-diazo-2-phenylacetate (**2a**), 45 μL (0.877 g/mL, 0.36 mmol) of $PhSiH_3$, 0.0029 g (0.015 mmol) of $Co(OAc)_2$, 0.0059 g (0.018 mmol) of **L4**, and 1.2 mL (0.25 M) of THF. After 12 h, the reaction was worked up. The crude mixture was purified by flash column chromatography using PE (50 mL) to PE/EA (100/1, 200 mL) as the eluent to give 0.0536 g (0.22 mmol, 72% yield) of the title compound as a pale yellow oil. IR (near): 3247, 2975, 2933, 2876, 1669, 1515, 1450, 1372 cm^{-1}. ^1H NMR: (400 MHz, $CDCl_3$) δ 10.60 (d, J = 3.2 Hz, 1H), 7.54 (d, J = 7.2 Hz, 2H), 7.31 (dd, J = 8.0, 7.2 Hz, 2H), 7.27-7.19 (m, 1H), 4.27 (q, J = 6.8 Hz, 2H), 3.62-3.50 (m, 1H), 1.77-1.64 (m, 1H), 1.63-1.51 (m, 1H), 1.32 (t, J = 6.8 Hz, 3H), 1.27 (d, J = 6.8 Hz, 3H), 0.97 (t, J = 7.6 Hz, 3H); ^{13}C NMR: (100 MHz, $CDCl_3$) δ 163.8, 137.5, 128.2, 127.7, 126.6, 124.4, 60.1, 57.9, 29.3, 19.7, 14.2, 10.3; HRMS (ESI) calculated for $C_{14}H_{21}N_2O_2$ (M+H$^+$) requires m/z 249.1603, found m/z 249.1607.

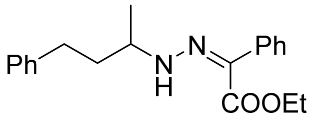

Ethyl (*Z*)-2-phenyl-2-(2-(1-(4-(trifluoromethyl)phenyl)ethyl)hydrazono)acetate (3z). Prepared according to the general procedure A using 88 μL (0.88 g/mL, 0.60 mmol) of 4-phenyl-1-butene (**1z**), 48 μL (1.165 g/ml, 0.3 mmol) of ethyl 2-diazo-2-phenylacetate (**2a**), 45 μL (0.877 g/mL, 0.60 mmol) of $PhSiH_3$, 0.0027 g (0.015 mmol) of $Co(OAc)_2$, 0.0057 g (0.018 mmol) of **L4**, and 1.2 mL (0.25 M) of THF. After 12 h, the reaction was worked up. The crude mixture was purified by flash column chromatography using

PE (50 mL) to PE/EA (60/1, 200 mL) as the eluent to give 0.0744 g (0.23 mmol, 76% yield) of the title compound as a pale yellow oil. IR (neat): 3246, 3027, 1666, 1513, 1449 cm^{-1}. ^{1}H NMR: (400 MHz, $CDCl_3$) δ 10.64 (d, J = 4.8 Hz, 1H), 7.55 (d, J = 7.2 Hz, 2H), 7.36-7.28 (m, 2H), 7.28-7.20 (m, 3H), 7.20-7.10 (m, 3H), 4.26 (q, J = 6.8 Hz, 2H), 3.72-3.57 (m, 1H), 2.80-2.60 (m, 2H), 2.10-1.94 (m, 1H), 1.92-1.77 (m, 1H), 1.38-1.22 (m, 6H); ^{13}C NMR: (100.9 MHz, $CDCl_3$) δ 163.7, 141.7, 137.4, 128.35, 128.31, 128.2, 127.6, 126.6, 125.8, 124.7, 60.1, 55.9, 38.1, 32.3, 20.4, 14.2; HRMS (ESI) calculated for $C_{20}H_{25}N_2O_2$ (M+H$^+$) requires m/z 325.1916, found m/z 325.1920.

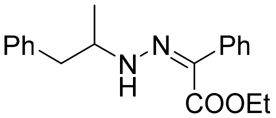

Ethyl (*Z*)-2-phenyl-2-(2-(1-phenylpropan-2-yl)hydrazono)acetate (3aa).

Prepared according to the general procedure A using 0.0425 g (0.36 mmol) of allylbenzene (**1aa**), 48 μL (1.165 g/ml, 0.3 mmol) of ethyl 2-diazo-2-phenylacetate (**2a**), 45 μL (0.877 g/mL, 0.36 mmol) of $PhSiH_3$, 0.0027 g (0.015 mmol) of $Co(OAc)_2$, 0.0062 g (0.018 mmol) of **L4**, and 1.2 mL (0.25 M) of THF. After 12 h, the reaction was worked up. The crude mixture was purified by flash column chromatography using PE (50 mL) to PE/EA (50/1, 200 mL) as the eluent to give 0.0698 g (0.23 mmol, 75% yield) of the title compound as a light yellow oil. IR (neat): 3246, 2928, 1670, 1516, 1450 cm^{-1}. ^{1}H NMR: (400 MHz, $CDCl_3$) δ 10.51 (d, J = 4.0 Hz, 1H), 7.50 (d, J = 7.6 Hz, 2H), 7.35-7.26 (m, 4H), 7.25-7.15 (m, 4H), 4.26 (q, J = 7.2 Hz, 2H), 3.89 (m, 1H), 3.04 (dd, J = 13.6, 6.8 Hz, 1H), 2.81 (dd, J = 13.6, 6.8 Hz, 1H), 1.35-1.24 (m, 6H); ^{13}C NMR: (100 MHz, $CDCl_3$) δ 163.6, 138.4, 137.3, 129.4, 128.4, 128.2, 127.7, 126.7, 126.3, 125.2, 60.2, 57.6, 43.1, 19.5, 14.2; HRMS (ESI) calculated for $C_{19}H_{23}N_2O_2$ (M+H$^+$) requires m/z 311.1754, found m/z 311.1764.

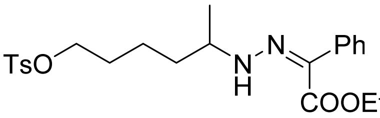

Ethyl (*Z*)-2-phenyl-2-(2-(6-(tosyloxy)hexan-2-yl)hydrazono)acetate (3ab).

Prepared according to the general procedure A using 0.0923 g (0.36 mmol) of hex-5-en-1-yl 4-methylbenzenesulfonate (**1ab**), 49 μL (1.165 g/ml, 0.3 mmol) of ethyl 2-diazo-2-phenylacetate (**2a**), 45 μL (0.877 g/mL, 0.36 mmol) of $PhSiH_3$, 0.0029 g (0.015

mmol) of $Co(OAc)_2$, 0.0058 g (0.018 mmol) of **L4**, and 1.2 mL (0.25 M) of THF. After 12 h, the reaction was worked up. The crude mixture was purified by flash column chromatography using PE (50 mL) to PE/EA (10/1, 200 mL) as the eluent to give 0.0963 g (0.22 mmol, 72% yield) of the title compound as a colorless oil. IR (neat): 3248, 2974, 1667, 1598, 1514, 1449, 1361 cm^{-1}. ^1H NMR: (400 MHz, $CDCl_3$) δ 10.51 (br, 1H), 7.78 (d, J = 8.0 Hz, 2H), 7.51 (d, J = 7.2 Hz, 2H), 7.36-7.27 (m, 4H), 7.27-7.19 (m, 1H), 4.27 (q, J = 7.2 Hz, 2H), 4.02 (t, J = 6.4 Hz, 2H), 3.64-3.48 (m, 1H), 2.43 (s, 3H), 1.73-1.55 (m, 3H), 1.52-1.36 (m, 3H), 1.32 (t, J = 7.2 Hz, 3H), 1.23 (d, J = 6.8 Hz, 3H); ^{13}C NMR: (100 MHz, $CDCl_3$) δ 163.8, 144.7, 137.3, 133.1, 129.8, 128.2, 127.9, 127.7, 126.7, 124.8, 70.3, 60.2, 56.3, 35.7, 28.7, 21.9, 21.6, 20.3, 14.2; HRMS (ESI) calculated for $C_{23}H_{31}N_2O_5S$ (M+H$^+$) requires m/z 447.1954, found m/z 447.1963.

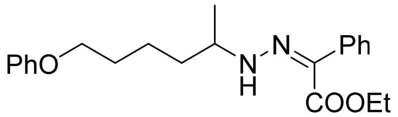

Ethyl (*Z*)-2-(2-(6-phenoxyhexan-2-yl)hydrazono)-2-phenylacetate (3ac).

Prepared according to the general procedure A using 0.0643 g (0.36 mmol) of (hex-5-en-1-yloxy)benzene (**1ac**), 49 μL (1.165 g/ml, 0.3 mmol) of ethyl 2-diazo-2-phenylacetate (**2a**), 45 μL (0.877 g/mL, 0.36 mmol) of $PhSiH_3$, 0.0029 g (0.015 mmol) of $Co(OAc)_2$, 0.0061 g (0.018 mmol) of **L4**, and 1.2 mL (0.25 M) of THF. After 12 h, the reaction was worked up. The crude mixture was purified by flash column chromatography using PE (50 mL) to PE/EA (60/1, 200 mL) as the eluent to give 0.0684 g (0.19 mmol, 62% yield) of the title compound as a pale yellow oil. IR (neat): 3247, 2938, 1667, 1600, 1514, 1371 cm^{-1}. ^1H NMR: (400 MHz, $CDCl_3$) δ 10.60 (d, J = 4.8 Hz, 1H), 7.53 (d, J = 8.0 Hz, 2H), 7.36-7.19 (m, 5H), 6.98-6.82 (m, 3H), 4.27 (q, J = 7.2 Hz, 2H), 3.96 (t, J = 6.4 Hz, 2H), 3.73-3.59 (m, 1H), 1.90-1.68 (m, 3H), 2.66-1.50 (m, 3H), 1.36-1.26 (m, 6H); ^{13}C NMR: (100 MHz, $CDCl_3$) δ 163.8, 159.0, 137.4, 129.4, 128.2, 127.7, 126.6, 124.6, 120.5, 114.5, 67.6, 60.1, 56.5, 36.1, 29.2, 22.6, 20.3, 14.2; HRMS (ESI) calculated for $C_{22}H_{29}N_2O_3$ (M+H$^+$) requires m/z 369.2178, found m/z 369.2183.

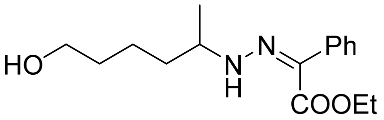

Ethyl (*Z*)-2-(2-(6-hydroxyhexan-2-yl)hydrazono)-2-phenylacetate (3ad).

Prepared according to the general procedure A using 0.0375 g (0.36 mmol) of hex-5-en-1-ol (**1ad**), 49 μL (1.165 g/ml, 0.3 mmol) of ethyl 2-diazo-2-phenylacetate (**2a**), 45 μL (0.877 g/mL, 0.36 mmol) of $PhSiH_3$, 0.0028g (0.015 mmol) of $Co(OAc)_2$, 0.0059g (0.018 mmol) of **L4**, and 1.2 mL (0.25 M) of THF. After 12 h, the reaction was worked up. The crude mixture was purified by flash column chromatography using PE (50 mL) to PE/EA (2/1, 200 mL) as the eluent to give 0.0570 g (0.20 mmol, 65% yield) of the title compound as a colorless oil. IR (neat): 3253, 2936, 1667, 1514, 1448, 1371 cm^{-1}. ^1H NMR: (400 MHz, $CDCl_3$) δ 10.58 (d, J = 5.2 Hz, 1H), 7.53 (d, J = 7.6 Hz, 2H), 7.31 (t, J = 7.6 Hz, 2H), 7.26-7.20 (m, 1H), 4.26 (q, J = 7.2 Hz, 2H), 3.70-3.3.57 (m, 3H), 1.74-1.65 (m, 1H), 1.64-1.52 (m, 4H), 1.52-1.41 (m, 2H), 1.31 (t, J = 7.2 Hz, 3H), 1.27 (d, J = 7.2 Hz, 3H); ^{13}C NMR: (100 MHz, $CDCl_3$) δ 163.8, 137.4, 128.2, 127.7, 126.6, 124.6, 62.6, 60.1, 56.5, 36.1, 32.6, 22.2, 20.3, 14.2; HRMS (ESI) calculated for $C_{16}H_{25}N_2O_3$ (M+H$^+$) requires m/z 293.1865, found m/z 293.1879.

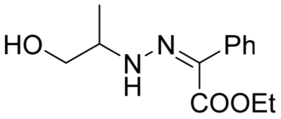

Ethyl (*Z*)-2-(2-(1-hydroxypropan-2-yl)hydrazono)-2-phenylacetate (3ae).

Prepared according to the general procedure A using 25 μL (0.854 g/mL, 0.36 mmol) of allyl alcohol (**1ae**), 49 μL (1.165 g/ml, 0.3 mmol) of ethyl 2-diazo-2-phenylacetate (**2a**), 45 μL (0.877 g/mL, 0.36 mmol) of $PhSiH_3$, 0.0026 g (0.015 mmol) of $Co(OAc)_2$, 0.0058g (0.018 mmol) of **L4**, and 1.2 mL (0.25 M) of THF. After 12 h, the reaction was worked up. The crude mixture was purified by flash column chromatography using PE (50 mL) to PE/EA (5/1, 200 mL) as the eluent to give 0.0370 g (0.15 mmol, 49% yield) of the title compound as a colorless oil. ^1H NMR: (400 MHz, $CDCl_3$) δ 10.54 (br, 1H), 7.50 (d, J = 7.2 Hz, 2H), 7.36-7.29 (m, 2H), 7.29-7.24 (m, 1H), 4.28 (q, J = 7.2 Hz, 2H), 3.84-3.69 (m, 3H), 2.50 (br, 1H), 1.32 (t, J = 7.2 Hz, 3H), 1.27 (d, J = 6.4 Hz, 3H); ^{13}C NMR: (100 MHz, $CDCl_3$) δ 163.6, 136.9, 128.2, 127.8, 127.0, 126.6, 67.0, 60.5, 56.9, 16.2, 14.2; HRMS (ESI) calculated for $C_{13}H_{19}N_2O_3$ (M+H$^+$) requires m/z 251.1396, found m/z 251.1400.

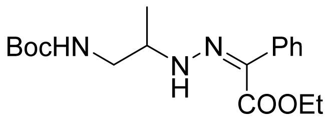

Ethyl (*Z*)-2-(2-(1-((*tert*-butoxycarbonyl)amino)propan-2-yl)hydrazono)-2-phenylacetate (3af).

Prepared according to the general procedure A using 0.0570 g (0.36 mmol) of *tert*-butyl-*N*-allycarbamate (**1af**), 49 μL (1.165 g/ml, 0.3 mmol) of ethyl 2-diazo-2-phenylacetate (**2a**), 45 μL (0.877 g/mL, 0.36 mmol) of $PhSiH_3$, 0.0028 g (0.015 mmol) of $Co(OAc)_2$, 0.0059 g (0.018 mmol) of **L4**, and 1.2 mL (0.25 M) of THF. After 12 h, the reaction was worked up. The crude mixture was purified by flash column chromatography using PE (50 mL) to PE/EA (10/1, 200 mL) as the eluent to give 0.0838 g (0.25 mmol, 84% yield) of the title compound as a light yellow oil. IR (neat): 3402, 2930, 1709, 1513, 1451 cm^{-1}. ^1H NMR: (400 MHz, $CDCl_3$) δ 10.48 (d, J = 4.0 Hz, 1H), 7.53 (d, J = 7.2 Hz, 2H), 7.36-7.29 (m, 2H), 7.29-7.23 (m, 1H), 4.95 (s, 1H), 4.28 (q, J = 7.2 Hz, 2H), 3.80-3.66 (m, 1H), 3.50-3.36 (m, 1H), 3.33-3.21 (m, 1H), 1.43 (s, 9H), 1.32 (t, J = 7.2 Hz, 3H), 1.27 (d, J = 6.8 Hz, 3H); ^{13}C NMR: (100 MHz, $CDCl_3$) δ 163.6, 156.0, 137.0, 128.2, 127.7, 126.9, 126.1, 79.3, 60.4, 56.0, 45.5, 28.3, 17.6, 14.2; HRMS (ESI) calculated for $C_{18}H_{28}N_3O_4$ (M+H$^+$) requires m/z 350.2080, found m/z 350.2080.

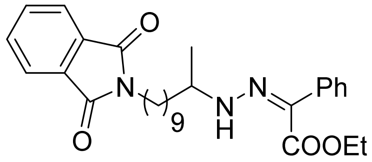

Ethyl (*Z*)-2-(2-(1-(1,3-dioxoisoindolin-2-yl)propan-2-yl)hydrazono)-2-phenylacetate (3ag).

Prepared according to the general procedure A using 0.1079 g (0.36 mmol) of 2-(undec-10-en-1-yl)isoindoline-1,3-dione (**1ag**), 48 μL (1.165 g/ml, 0.3 mmol) of ethyl 2-diazo-2-phenylacetate (**2a**), 45 μL (0.877 g/mL, 0.36 mmol) of $PhSiH_3$, 0.0027 g (0.015 mmol) of $Co(OAc)_2$, 0.0058 g (0.018 mmol) of **L4**, and 1.2 mL (0.25 M) of THF. After 12 h, the reaction was worked up. The crude mixture was purified by flash column chromatography using PE (50 mL) to PE/EA (15/1, 200 mL) as the eluent to give 0.1043 g (0.18 mmol, 61% yield) of the title compound as a light yellow oil. IR (neat): 3250, 2929, 1714, 1667, 1514 cm^{-1}. ^1H NMR: (400 MHz, $CDCl_3$) δ 10.58 (br, 1H), 7.87-7.79 (m, 2H), 7.74-7.65 (m, 2H), 7.56-7.50 (m, 2H), 7.35-7.26 (m, 2H), 7.25-7.20 (m, 1H), 4.27 (q, J = 7.2 Hz, 2H), 3.67 (t, J = 7.2 Hz, 2H), 3.65-3.56 (m, 1H), 1.72-1.62 (m, 3H), 1.55-1.45 (m, 1H), 1.40-1.23 (m, 18H); ^{13}C NMR:

(100 MHz, $CDCl_3$) δ 168.4, 163.7, 137.5, 133.8, 132.1, 128.2, 127.6, 126.5, 124.3, 123.1, 60.1, 56.6, 38.0, 36.4, 29.5, 29.4, 29.3, 29.1, 28.5, 26.8, 25.9, 20.4, 14.2; HRMS (ESI) calculated for $C_{29}H_{37}N_3NaO_4$ $(M+Na^+)$ requires m/z 514.2676, found m/z 514.2675.

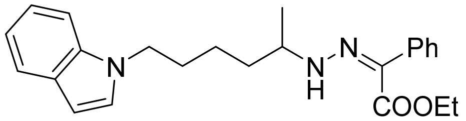

Ethyl (*Z*)-2-(2-(6-(1*H*-indol-1-yl)hexan-2-yl)hydrazono)-2-phenylacetate (3ah).

Prepared according to the general procedure A using 0.0761 g (0.36 mmol) of 1-(hex-5-en-1-yl)-1*H*-indole (**1ah**), 48 μL (1.165 g/ml, 0.3 mmol) of ethyl 2-diazo-2-phenylacetate (**2a**), 45 μL (0.877 g/mL, 0.36 mmol) of $PhSiH_3$, 0.0028 g (0.015 mmol) of $Co(OAc)_2$, 0.0058 g (0.018 mmol) of **L4**, and 1.2 mL (0.25 M) of THF. After 12 h, the reaction was worked up. The crude mixture was purified by flash column chromatography using PE (50 mL) to PE/EA (30/1, 200 mL) as the eluent to give 0.0799 g (0.20 mmol, 68% yield) of the title compound as a light yellow oil. IR (neat): 3246, 2927, 1665, 1512, 1460 cm^{-1}. ^1H NMR: (400 MHz, $CDCl_3$) δ 10.55 (br, J = 4.0 Hz, 1H), 7.61 (d, J = 8.0 Hz, 1H), 7.52 (d, J = 7.6 Hz, 2H), 7.37-7.27 (m, 3H), 7.26-7.21 (m, 1H), 7.21-7.13 (m, 1H), 7.11-7.01 (m, 2H), 6.46 (d, J = 2.0 Hz, 1H), 4.26 (q, J = 7.2 Hz, 2H), 4.09 (t, J = 7.2 Hz, 2H), 3.65-3.52 (m, 1H), 1.94-1.77 (m, 2H), 1.76-1.63 (m, 1H), 1.58-1.46 (m, 1H), 1.46-1.35 (m, 2H), 1.31 (t, J = 7.2 Hz, 3H), 1.23 (d, J = 6.4 Hz, 3H); ^{13}C NMR: (100 MHz, $CDCl_3$) δ 163.8, 137.3, 135.9, 128.6, 128.2, 127.7, 126.6, 124.7, 121.3, 120.9, 119.1, 109.3, 100.9, 60.2, 56.3, 46.2, 36.0, 30.1, 23.4, 20.3, 14.2; HRMS (ESI) calculated for $C_{24}H_{29}N_3NaO_2$ $(M+Na^+)$ requires m/z 414.2152, found m/z 414.2156.

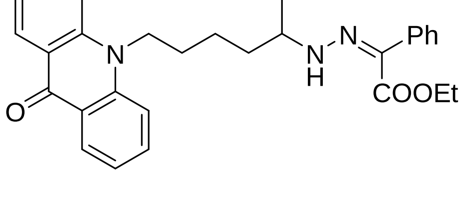

Ethyl (*Z*)-2-(2-(6-(9-oxoacridin-10(9*H*)-yl)hexan-2-yl)hydrazono)-2-phenylacetate (3ai).

Prepared according to the general procedure A using 0.1007 g (0.36 mmol) of 10-(hex-5-en-1-yl)-10,10a-dihydroacridin-9(8*H*)-one (**1ai**), 49 μL (1.165 g/ml, 0.3 mmol) of ethyl 2-diazo-2-phenylacetate (**2a**), 45 μL (0.877

g/mL, 0.36 mmol) of $PhSiH_3$, 0.0026 g (0.015 mmol) of $Co(OAc)_2$, 0.0056 g (0.018 mmol) of **L4**, and 1.2 mL (0.25 M) of THF. After 12 h, the reaction was worked up. The crude mixture was purified by flash column chromatography using PE (50 mL) to PE/EA (5/1, 200 mL) as the eluent to give 0.1051 g (0.23 mmol, 75% yield) of the title compound as a golden to brown viscous oil. IR (neat): 3244, 2933, 2241, 1634, 1602, 1491, 1375 cm^{-1}. ^1H NMR: (400 MHz, $CDCl_3$) δ 10.61 (d, J = 4.8 Hz, 1H), 8.57 (d, J = 7.2 Hz, 2H), 7.68 (t, J = 8.4 Hz, 2H), 7.55 (d, J = 7.2 Hz, 2H), 7.45 (d, J = 8.4 Hz, 2H), 7.33 (t, J = 7.2 Hz, 2H), 7.30-7.22 (m, 3H), 4.37-4.22 (m, 4H), 3.76-3.62 (m, 1H), 2.05-1.89 (m, 2H), 1.89-1.78 (m, 1H), 1.73-1.59 (m, 3H), 1.39-1.27 (m, 6H); ^{13}C NMR: (100 MHz, $CDCl_3$) δ 177.9, 163.9, 141.7, 137.3, 133.9, 128.2, 128.0, 127.7, 126.8, 125.0, 122.4, 121.2, 114.4, 60.3, 56.4, 46.0, 36.1, 27.1, 23.4, 20.5, 14.2; HRMS (ESI) calculated for $C_{29}H_{32}N_3O_3$ $(M+H^+)$ requires m/z 470.2444, found m/z 470.2449.

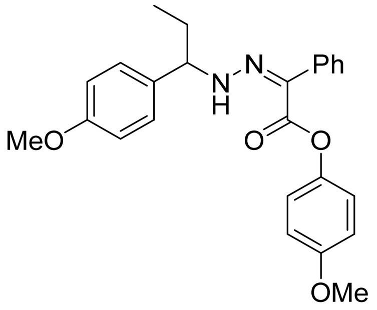

4-methoxyphenyl (*Z*)-2-(2-(1-(4-methoxyphenyl)propyl)hydrazono)-2-phenylacetate (3aj).

Prepared according to the general procedure A using 0.0534 g (0.36 mmol) of *trans*-anethole (**1aj**), 0.0805 mg (0.3 mmol) of 4-methoxyphenyl 2-diazo-2-phenylacetate (**2b**), 45 μL (0.877 g/mL, 0.36 mmol) of $PhSiH_3$, 0.0027 g (0.015 mmol) of $Co(OAc)_2$, 0.0056 g (0.018 mmol) of **L3**, and 1.2 mL (0.25 M) of THF. After 12 h, the reaction was worked up. The crude mixture was purified by flash column chromatography using PE (50 mL) to PE/EA (20/1, 200 mL) as the eluent to give 0.1042 g (0.25 mmol, 83% yield) of the title compound as a light yellow oil. IR (neat): 3251, 2965, 1668, 1609, 1512 cm^{-1}. ^1H NMR: (400 MHz, $CDCl_3$) δ 10.97 (d, 1H), 7.63 (d, J = 7.6 Hz, 2H), 7.39-7.30 (m, 2H), 7.27 (d, J = 7.2 Hz, 1H), 7.22 (d, J = 8.4 Hz, 2H), 7.03 (d, J = 8.8 Hz, 2H), 6.92-6.83 (m, 4H), 4.55-4.46 (m, 1H), 3.77 (s, 6H), 2.17-2.04 (m, 1H), 1.96-1.82 (m, 1H), 0.93 (t, J = 7.2 Hz, 3H); ^{13}C NMR: (100 MHz, $CDCl_3$) δ 162.5, 158.9, 157.4, 143.4, 136.9, 133.6, 128.4, 128.1, 127.8, 126.9, 124.1, 122.5, 114.5, 114.0, 66.6, 55.5, 55.2, 28.8, 10.7; HRMS (ESI) calculated for $C_{25}H_{27}N_2O_4$

(M+H$^+$) requires m/z 419.1965, found m/z 419.1969

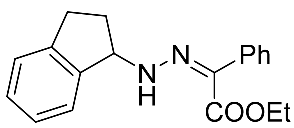

Ethyl (*Z*)-2-(2-(2,3-dihydro-1*H*-inden-1-yl)hydrazono)-2-phenylacetate (3ak).

Prepared according to the general procedure A using 0.0701 g (0.36 mmol) of indene (**1ak**), 48 μL (1.165 g/ml, 0.3 mmol) of ethyl 2-diazo-2-phenylacetate (**2a**), 45 μL (0.877 g/mL, 0.36 mmol) of $PhSiH_3$, 0.0027 g (0.015 mmol) of $Co(OAc)_2$, 0.0059 g (0.018 mmol) of **L4**, and 1.2 mL (0.25 M) of THF. After 12 h, the reaction was worked up. The crude mixture was purified by flash column chromatography using PE (50 mL) to PE/EA (100/1, 200 mL) as the eluent to give 0.0796 g (0.26 mmol, 86% yield) of the title compound as a light yellow oil. IR (neat): 3248, 3027, 2978, 2939, 1735, 1670, 1601, 1514cm^{-1}. ^{1}H NMR: (400 MHz, $CDCl_3$) δ 10.74 (d, J = 5.6 Hz, 1H), 7.57 (d, J = 7.2 Hz, 2H), 7.42-7.38 (m, 1H), 7.37-7.31 (m, 2H), 7.28-7.20 (m, 4H), 5.25-5.18 (m, 1H), 4.24 (q, J = 6.8 Hz, 2H), 3.09-3.00 (m, 1H), 2.93-2.83 (m, 1H), 2.64-2.55 (m, 1H), 2.18-2.08 (m, 1H), 1.29 (t, J = 7.2 Hz, 3H); ^{13}C NMR: (100 MHz, $CDCl_3$) δ 163.7, 143.6, 142.7, 137.2, 128.3, 128.1, 127.7, 126.8, 126.7, 125.7, 124.8, 124.6, 66.4, 60.3, 33.1, 30.2, 14.2; HRMS (ESI) calculated for $C_{19}H_{21}N_2O_2$ (M+H$^+$) requires m/z 309.1603, found m/z 309.1609.

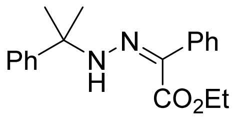

Ethyl (*Z*)-2-phenyl-2-(2-(2-phenylpropan-2-yl)hydrazono)acetate (3al).

Prepared according to the general procedure A using 0.0701 g (0.6 mmol) of 2-phenyl-1-propene (**1al**), 48 μL (1.165 g/ml, 0.3 mmol) of ethyl 2-diazo-2-phenylacetate (**2a**), 45 μL (0.877 g/mL, 0.36 mmol) of $PhSiH_3$, 0.0029 g (0.015 mmol) of $Co(OAc)_2$, 0.0058 g (0.018 mmol) of **L4**, and 1.2 mL (0.25 M) of THF. After 12 h, the reaction was worked up. The crude mixture was purified by flash column chromatography using PE (50 mL) to PE/EA (100/1, 200 mL) as the eluent to give 0.0740 g (0.24 mmol, 79% yield) of the title compound as a light yellow oil. IR (neat): 3254, 2957, 2926, 1737, 1497 cm^{-1}. ^{1}H NMR: (400 MHz, $CDCl_3$) δ 10.92 (s, 1H), 7.55 (d, J = 7.6 Hz, 2H), 7.42 (d, J = 7.6 Hz, 2H), 7.36-7.27 (m, 4H), 7.25-7.20 (m, 2H),

4.29 (q, J = 7.2 Hz, 2H), 1.71 (s, 6H), 1.33 (t, J = 7.2 Hz, 3H); ^{13}C NMR: (100 MHz, $CDCl_3$) δ 163.6, 147.4, 137.4, 128.4, 128.2, 127.6, 126.7, 126.6, 125.5, 124.9, 60.4, 60.3, 28.8, 14.2; HRMS (ESI) calculated for $C_{19}H_{22}N_2NaO_2$ (M+Na$^+$) requires m/z 333.1579, found m/z 333.1581.

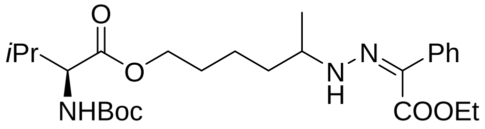

5-(2-((*Z*)-2-ethoxy-2-oxo-1-phenylethylidene)hydrazinyl)hexyl (*tert*-butoxycarbonyl)-*L*-valinate (3am).

Prepared according to the general procedure A using 0.1100 g (0.36 mmol) of hex-5-en-1-yl (*tert*-butoxycarbonyl)-*L*-valinate (**1am**),[16] 49 μL (1.165 g/ml, 0.3 mmol) of ethyl 2-diazo-2-phenylacetate (**2a**), 45 μL (0.877 g/mL, 0.36 mmol) of $PhSiH_3$, 0.0029 g (0.015 mmol) of $Co(OAc)_2$, 0.0060 g (0.018 mmol) of **L4**, and 1.2 mL (0.25 M) of THF. After 12 h, the reaction was worked up. The crude mixture was purified by flash column chromatography using PE (50 mL) to PE/EA (10/1, 200 mL) as the eluent to give 0.1144 g (0.23 mmol, 78% yield, *dr* = 1:1) of the title compound as a colorless oil. IR (neat): 3371, 3250, 2975, 2876, 2821, 1718, 1669, 1513, 1392, 1367 cm^{-1}. ^{1}H NMR: (400 MHz, $CDCl_3$) δ 10.57 (d, J = 4.4 Hz, 1H), 7.53 (d, J = 8.0 Hz, 2H), 7.35-7.27 (m, 2H), 7.27-7.20 (m, 1H), 5.04 (d, J = 8.4 Hz, 1H), 4.27 (q, J = 7.2 Hz, 2H), 4.22-4.06 (m, 3H), 3.70-3.56 (m, 1H), 2.20-2.00 (m, 1H), 1.78-1.46 (m, 6H), 1.44 (s, 9H), 1.32 (t, J = 7.2 Hz, 3H), 1.27 (d, J = 6.4 Hz, 3H), 0.95 (d, J = 7.2 Hz, 3H), 0.87 (d, J = 7.2 Hz, 3H); HRMS (ESI) calculated for $C_{26}H_{42}N_3O_6$ (M+H$^+$) requires m/z 492.3074, found m/z 492.3080.

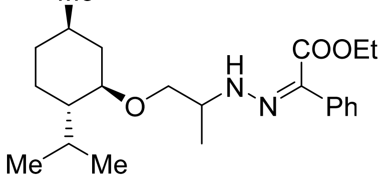

Ethyl (*Z*)-2-(2-(1-(((1*R*,2*S*,5*R*)-2-isopropyl-5-methylcyclohexyl)oxy)propan-2-yl)hydrazono)-2-phenylacetate (3an).

Prepared according to the general procedure A using 0.0715 g (0.36 mmol) of (1*S*,2*R*,4*R*)-2-(allyloxy)-1-isopropyl-4-methylcyclohexane (**1an**),[17] 49 μL (1.165 g/ml, 0.3 mmol) of ethyl 2-diazo-2-phenylacetate (**2a**), 45 μL (0.877 g/mL, 0.36 mmol) of $PhSiH_3$, 0.0029 g (0.015 mmol) of $Co(OAc)_2$, 0.0058 g

(0.018 mmol) of **L4**, and 1.2 mL (0.25 M) of THF. After 12 h, the reaction was worked up. The crude mixture was purified by flash column chromatography using PE (50 mL) to PE/EA (5/1, 200 mL) as the eluent to give 0.0845 g (0.22 mmol, 72% yield, *dr* = 1:1) of the title compound as a pale yellow oil. ^{1}H NMR: (400 MHz, $CDCl_3$) δ 10.61 (d, J = 4.4 Hz, 0.5H), 10.55 (d, J = 4.4 Hz, 0.5H), 7.54 (d, J = 8.0 Hz, 2H), 7.35-7.27 (m, 2H), 7.27-7.20 (m, 1H), 4.27 (q, J = 6.8 Hz, 2H), 2.85-3.74 (m, 1H), 3.74-3.65 (m, 1H), 3.42-3.32 (m, 1H), 3.10-2.94 (m, 1H), 2.30-2.14 (m, 1H), 2.14-2.00 (m, 1H), 1.68-1.55 (m, 2H), 1.40-1.19 (m, 8H), 1.10-0.80 (m, 9H), 0.73 (t, J = 6.8 Hz, 3H); HRMS (ESI) calculated for $C_{23}H_{36}N_2NaO_3$ (M+Na$^+$) requires m/z 411.2624, found m/z 411.2621.

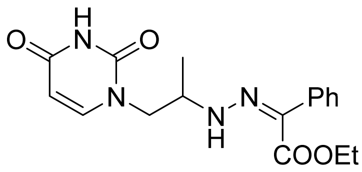

Ethyl (*Z*)-2-(2-(1-(2,4-dioxo-3,4-dihydropyrimidin-1(2*H*)-yl)propan-2-yl)hydrazono)-2-phenylacetate (3ao).

Prepared according to the general procedure A using 0.0549 g (0.36 mmol) of 1-allylpyrimidine-2,4(1*H*,3*H*)-dione (**1ao**),[18] 48 μL (1.165 g/ml, 0.3 mmol) of ethyl 2-diazo-2-phenylacetate (**2a**), 45 μL (0.877 g/mL, 0.36 mmol) of $PhSiH_3$, 0.0028 g (0.015 mmol) of $Co(OAc)_2$, 0.0058 g (0.018 mmol) of **L4**, and 1.2 mL (0.25 M) of THF. After 12 h, the reaction was worked up, washed with DCM/MeOH (1:1, 50 mL). The crude mixture was purified by flash column chromatography using PE (50 mL) to PE/EA (1/2, 200 mL) as the eluent to give 0.0592 g (0.18 mmol, 59% yield) of the title compound as a light yellow oil. IR (neat): 3245, 3057, 1679, 1515, 1454, 1381 cm^{-1}. ^{1}H NMR: (400 MHz, $CDCl_3$) δ 10.41 (d, J = 4.4 Hz, 1H), 9.44 (s, 1H), 7.53-7.45 (m, 2H), 7.37-7.27 (m, 3H), 7.02 (d, J = 8.0 Hz, 1H), 5.58 (dd, J = 8.0, 1.6 Hz, 1H), 4.35-4.24 (qd, J = 7.2, 2.0 Hz, 2H), 4.08 (dd, J = 13.6, 4.0 Hz, 1H), 4.04-3.94 (m, 1H), 3.76 (dd, J = 13.6, 8.8 Hz, 1H), 1.35 (d, J = 5.6 Hz, 3H), 1.32 (t, J = 7.2 Hz, 3H); ^{13}C NMR: (100 MHz, $CDCl_3$) δ 163.8, 163.4, 151.1, 145.4, 136.5, 128.0, 127.8, 127.7, 127.3, 101.6, 60.7, 54.3, 53.0, 17.6, 14.1; HRMS (ESI) calculated for $C_{17}H_{21}N_4O_4$ (M+H$^+$) requires m/z 345.1557, found m/z 345.1567.

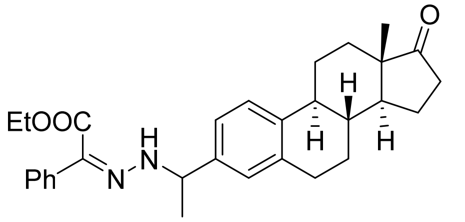

Ethyl (*Z*)-2-(2-(1-((8*R*,9*S*,13*S*,14*S*)-13-methyl-17-oxo-7,8,9,11,12,13,14,15,16,17-decahydro-6*H*-cyclopenta[a]phenanthren-3-yl)ethyl)hydrazono)-2-phenylacetate (3ap).

Prepared according to the general procedure A using 0.1009 g (0.36 mmol) of (8*R*,9*S*,13*S*,14*S*)-13-methyl-3-vinyl-6,7,8,9,11,12,13,14,15,16-decahydro-17*H*-cyclopenta[a]phenanthren-17-one (**1ap**),[19] 48 μL (1.165 g/ml, 0.3 mmol) of ethyl 2-diazo-2-phenylacetate (**2a**), 45 μL (0.877 g/mL, 0.36 mmol) of $PhSiH_3$, 0.0027 g (0.015 mmol) of $Co(OAc)_2$, 0.0057 g (0.018 mmol) of **L4**, and 1.2 mL (0.25 M) of THF. After 12 h, the reaction was worked up. The crude mixture was purified by flash column chromatography using PE (50 mL) to PE/EA (10/1, 200 mL) as the eluent to give 0.1258 g (0.27 mmol, 89% yield, *dr* = 1:1) of the title compound as a light yellow oil. IR (neat): 3248, 2925, 1739, 1668, 1511 cm^{-1}. ^1H NMR: (400 MHz, $CDCl_3$) δ 10.77 (d, J = 2.8 Hz, 1H), 7.53 (d, J = 7.2 Hz, 2H), 7.35-7.26 (m, 3H), 7.26-7.20 (m, 1H), 7.17-7.11 (m, 1H), 7.08 (s, 1H), 4.79-4.68 (m, 1H), 4.26 (q, J = 7.2 Hz, 2H), 2.92 (dd, J = 8.8, 4.0 Hz, 2H), 2.55-2.45 (m, 1H), 2.45-2.39 (m, 1H), 2.35-2.25 (m, 1H), 2.20-2.10 (m, 1H), 2.10-1.90 (m, 3H), 1.65-1.60 (m, 4H), 1.60-1.45 (m, 5H), 1.30 (t, J = 7.2 Hz, 3H), 0.90 (s, 3H); HRMS (ESI) calculated for $C_{30}H_{36}N_2NaO_3$ ($M+Na^+$) requires m/z 495.2618, found m/z 495.2613.

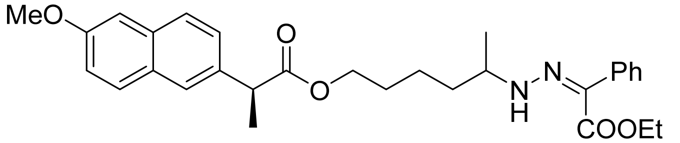

5-(2-((*Z*)-2-ethoxy-2-oxo-1-phenylethylidene)hydrazinyl)hexyl (2*S*)-2-(6-methoxynaphthalen-2-yl)propanoate (3aq).

Prepared according to the general procedure A using 0.1125 g (0.36 mmol) of hex-5-en-1-yl (*S*)-2-(6-methoxynaphthalen-2-yl)propanoate (**1aq**),[20] 48 μL (1.165 g/ml, 0.3 mmol) of ethyl 2-diazo-2-phenylacetate (**2a**), 45 μL (0.877 g/mL, 0.36 mmol) of $PhSiH_3$, 0.0027 g (0.015 mmol) of $Co(OAc)_2$, 0.0061 g (0.019 mmol) of **L4**, and 1.2 mL (0.25 M) of THF. After 12 h, the reaction was worked up. The crude mixture was

purified by flash column chromatography using PE (50 mL) to PE/EA (18/1, 200 mL) as the eluent to give 0.0930 g (0.18 mmol, 61% yield, *dr* = 1:1) of the title compound as a light yellow oil. IR (neat): 3250, 2937, 1731, 1666, 1606 cm^{-1}. ^1H NMR: (400 MHz, $CDCl_3$) δ 10.53 (d, J = 4.0 Hz, 1H), 7.72-7.62 (m, 3H), 7.52 (d, J = 7.6 Hz, 2H), 7.39 (dd, J = 8.4, 1.6 Hz, 1H), 7.35-7.27 (m, 2H), 7.26-7.20 (m, 1H), 7.15-7.06 (m, 2H), 4.26 (q, J = 7.2 Hz, 2H), 4.07 (t, J = 6.4 Hz, 2H), 3.88 (s, 3H), 3.83 (q, J = 7.2 Hz, 1H), 3.55-3.44 (m, 1H), 1.62-1.53 (m, 6H), 1.50-1.34 (m, 2H), 1.33-1.27 (m, 4H), 1.16 (dd, J = 6.4, 2.4 Hz, 3H); HRMS (ESI) calculated for $C_{30}H_{37}N_2O_5$ ($M+H^+$) requires m/z 505.2697, found m/z 505.2707.

Gram-scale Reaction and Further Derivatizations

Gram-scale Reaction

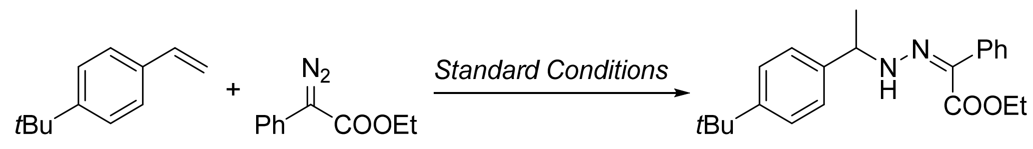

A 100 mL Schlenk flask equipped with a magnetic stirrer and a flanging rubber plug was dried with flame under vacuum. When cooled to ambient temperature, it was vacuumed and flushed with N_2 and repeated for three times. To the flask, $Co(OAc)_2$ 0.0442 g (0.25 mmol), **L4** 0.0943 g (0.30 mmol), THF (20 ml) were added. The flask was degassed and stirred for 30 min at room temperature. Then, $PhSiH_3$ (740 μL, 0.877 g/mL, 6 mmol), ethyl 2-diazo-2-phenylacetate (**2a**) (800 μL, 1.165 g/ml, 5 mmol) and 4-*tert*-butylstyrene (**1a**) (1.1 mL, 0.875 g/mL, 6 mmol) were added in sequence. After 12 h, the reaction was quenched with 50 ml of PE and the mixture was filtered through a pad of silica gel and washed with PE/EA (5/1, 100 mL). The combined filtrates ware concentrated and purified by flash column chromatography using PE/EA (80/1, 400 mL) as the eluent to afford 1.6498 g (4.68 mmol, 94% yield) of the title compound as a pale yellow oil.

Further Derivatizations

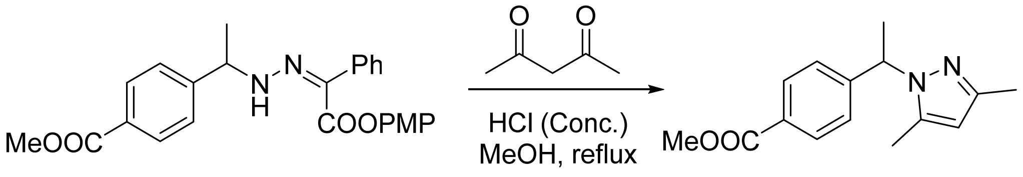

3m 4

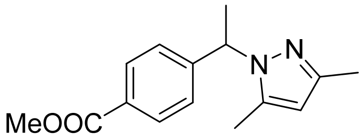

Methyl 4-(1-(3,5-dimethyl-1*H*-pyrazol-1-yl)ethyl)benzoate (4)

Prepared according to a previously reported procedure.[21] To a 25 mL round bottomed flask equipped with a condenser and a magnetic stirrer was added 0.1290 g (0.3 mmol) of **3m**, 3.0 mL (0.10 M) of methanol, 123 μL (0.975 g/mL, 1.2 mmol) of acetylacetone and 250 μL (3.0 mmol) of Conc. HCl. The reaction mixture was warmed up to 70 °C, and stirred for 12 h. After cooling to room temperature, the reaction mixture was quenched with water (20 mL) and extracted with EA (20mL x 3). The combined organic layers were dried over anhydrous Na_2SO_4, filtered, and concentrated by rotary evaporation. Purification by column chromatography using PE (50 mL) to PE/EA (10/1, 200 mL) with 1% of Et_3N as the eluent to afford 0.0666 g (0.26 mmol, 86% yield) of the title compound as a colorless oil. IR (neat): 2989, 2945, 1724, 1612, 1555, 1437, 1379 cm^{-1}. ^{1}H NMR: (400 MHz, $CDCl_3$) δ 7.96 (d, J = 8.4 Hz, 2H), 7.14 (d, J = 8.4 Hz, 2H), 5.84 (s, 1H), 5.37 (q, J = 7.2 Hz, 1H), 3.89 (s, 3H), 2.28 (s, 3H), 2.08 (s, 3H), 1.91 (d, J = 7.2 Hz, 3H); ^{13}C NMR: (100 MHz, $CDCl_3$) δ 166.7, 148.1, 147.3, 139.0, 130.0, 129.1, 125.9, 105.7, 57.0, 52.0, 21.5, 13.7, 11.1; HRMS (ESI) calculated for $C_{15}H_{19}N_2O_2$ (M+H$^+$) requires m/z 259.1447, found m/z 259.1460.

General Procedure B for a three-step reaction from alkenes to Boc group protected amines

Step B1. Hydroamination of alkenes: A 25 mL Schlenk flask equipped with a magnetic stirrer and a flanging rubber plug was dried with flame under vacuum. When cooled to ambient temperature, it was vacuumed and flushed with N_2 and repeated for three times. To the flask, $Co(OAc)_2$ (0.015 mmol), **L3** or **L4** (0.018 mol), THF (1.2 mL) were added. The flask was degassed and stirred for 30 min at room temperature. Then,

$PhSiH_3$ (0.36 mol), diazo compound (0.3 mmol) and alkene (0.36 mmol) were added in sequence. After 12 h, the reaction was quenched with 10 ml of PE and the mixture was filtered through a pad of silica gel and washed with PE/EA (5/1, 50 mL). The combined filtrates ware concentrated to afford a yellow oil.

Step B2. Cleavage of *N-N* bond according to the previously reported procedure[22] with modification: To the above suspension, AcOH-THF-H_2O (3:1:1 v/v/v, 3 mL) was added, followed by addition of activated Zn powder (0.5 g, 7.5 mmol) in several portions at room temperature. After that, the mixed solution was warmed up to 60 ^oC and stirred until completion monitored by TLC (usually 3 h). Then, the reaction mixture was cooled down to room temperature and quenched with water (20 mL). The reaction mixture was basified with a solution of NaOH (6 N) until the solution turned clear (pH>10) and then extracted with Et_2O (20 mL x 4). The combined organic layers were dried over anhydrous Na_2SO_4, filtered, concentrated to give a yellow oil which was used for the next step without further purification.

Step 3. Protection of free amines with Boc group: To the above oil, 3 mL (0.1 M) of THF, 0.20 g (0.9 mmol) of Boc_2O and 0.10 g (0.9 mmol) of Na_2CO_3 were added, followed by 2 h stirring. The mixture was quenched with water (20 mL) and then extracted with Et_2O (20 mL x 4). The combined organic layers were dried over anhydrous Na_2SO_4, filtered, concentrated, and purified by flash column chromatography using PE/EA as the eluent to give the corresponding product.

General Procedure C for a three-step reaction from alkenes to Bz group protected amines

Step C1 and **C2** were the same as **Step B1** and **B2** in **General Procedure B**.

Step C3. Protection of Free Amines with Bz group: To the above oil, 3 mL (0.1 M) of THF, 55 μL (1.211 g/mL, 0.45 mmol) of BzCl and 84 μL (0.728 g/mL, 0.6 mmol) of Et_3N were added, followed by 2 h stirring. The mixture was quenched with water (20 mL) and then extracted with Et_2O (20 mL x 4). The combined organic layers were dried over anhydrous Na_2SO_4, filtered, concentrated, and purified by flash column chromatography using PE/EA as the eluent to give the corresponding product.

General Procedure D for a three-step reaction from alkenes to Alkyl group protected amines

Step D1 and **D2** were the same as **Step B1** and **B2** in **General Procedure B**.

Step D3. Protection of Free Amines with Alkyl groups: To the above oil, 3 mL (0.1 M) of methanol, (0.3 mmol) of aldehyde were added. The resulting solution was stirred for 10 min. Then, 0.0189 g (0.3 mmol, 1 equiv.) of $NaBH_3CN$ was added followed by addition of HCl (Conc. 0.25 mL). After stirring overnight, the reaction was quenched with water, basified with solid potassium hydroxide, extracted with EA (50 mL x 3). The combined organic layers were dried over anhydrous Na_2SO_4, filtered, concentrated, and purified by flash column chromatography using PE/EA as the eluent to give the corresponding product.

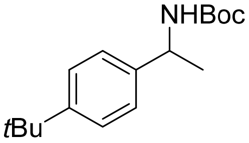

***Tert*-butyl (1-(4-(*tert*-butyl)phenyl)ethyl)carbamate (6a).** Prepared according to General Procedure B using 60 μL (0.875 g/mL, 0.36 mmol) of 1-(*tert*-butyl)-4-vinylbenzene (**1a**), 48 μL (1.165 g/ml, 0.3 mmol) of ethyl 2-diazo-2-phenylacetate (**2a**), 45 μL (0.877 g/mL, 0.36 mmol) of $PhSiH_3$, 0.0029 g (0.015 mmol) of $Co(OAc)_2$, 0.0058 g (0.018 mmol) of **L4**, 1.2 mL (0.25 M) of THF, 0.49 g (7.5 mmol) of Zn, 3 mL (0.1 M) of mixture AcOH-THF-H_2O (3/1/1, v/v/v), 180 μL (0.95 g/mL, 0.9 mmol) of Boc_2O, 0.10 g (0.9 mmol) of Na_2CO_3 and 3 mL (0.1 M) of THF. Upon completion, the reaction was worked up, and purified by flash column chromatography using PE/EA (20/1, 200 mL) with 1% of Et_3N as the eluent to give 0.0461 g (0.17 mmol, 55% yield) of the title compound as a white solid. M.p.: 90.0-91.2 °C. IR (neat): 3348, 2962, 2927, 1690, 1522, 1455, 1367cm^{-1}. ^1H NMR: (400 MHz, $CDCl_3$) δ 7.35 (d, J = 8.4 Hz, 2H), 7.23 (d, J = 8.4 Hz, 2H), 5.0 (m, 2H), 1.50-1.37 (m, 12H), 1.31 (s, 9H); ^{13}C NMR: (100 MHz, $CDCl_3$) δ 155.1, 150.0, 140.8, 125.6, 125.4, 79.3, 49.7, 34.4, 31.3, 28.4, 22.5; HRMS (ESI) calculated for $C_{17}H_{27}NNaO_2$ (M+Na$^+$) requires m/z 300.1939, found m/z 300.1945.

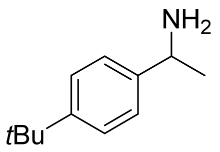

1-(4-(*tert*-butyl)phenyl)ethan-1-amine (5a)

Prepared according to **Step B1** and **B2** in **General Procedure B** using 1-(*tert*-butyl)-4-vinylbenzene (**1a**). ^{1}H NMR: (400 MHz, $CDCl_3$) δ 7.36 (d, J = 8.0 Hz, 2H), 7.27 (d, J = 8.0 Hz, 2H), 4.09 (q, J = 6.4 Hz, 1H), 1.74 (br, 2H), 1.39 (d, J = 6.4 Hz, 3H), 1.32 (s, 9H); The NMR spectra were consistent with the spectra reported in the literature.[23]

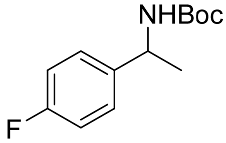

***Tert*-butyl (1-(4-fluorophenyl)ethyl)carbamate (6c).**

Prepared according to the General Procedure B using 0.0450 g (0.36 mmol) of 4-fluorostyrene (**1c**), 48 μL (1.165 g/ml, 0.3 mmol) of ethyl 2-diazo-2-phenylacetate (**2a**), 45 μL (0.877 g/mL, 0.36 mmol) of $PhSiH_3$, 0.0029 g (0.015 mmol) of $Co(OAc)_2$, 0.0058 g (0.018 mmol) of **L4**, 1.2 mL (0.25 M) of THF, 0.49 g (7.5 mmol) of Zn, 3 mL (0.1 M) of mixture AcOH-THF-H_2O (3/1/1, v/v/v), 180 μL (0.95 g/mL, 0.9 mmol) of Boc_2O, 0.10 g (0.9 mmol) of Na_2CO_3 and 3 mL (0.1 M) of THF. Upon completion, the worked up, and purified by flash column chromatography using PE/EA (50/1, 200 mL) with 1% of Et_3N as the eluent to give 0.0503 g (0.21 mmol, 70% yield) of the title compound as a white solid. M.p.: 84.3-85.3 ^{o}C. IR (neat): 3450, 3338, 2978, 2932, 1701, 1605, 1511, 1455, 1369 cm^{-1}. ^{1}H NMR: (400 MHz, $CDCl_3$) δ 7.29-7.23 (m, 2H), 7.04-6.97 (m, 2H), 4.85-4.70 (m, 2H), 1.45-1.38 (m, 12H); ^{13}C NMR: (100 MHz, $CDCl_3$) δ 161.9 (d, J = 247 Hz), 155.0, 139.8, 127.4 (d, J = 8 Hz), 115.2 (d, J = 22 Hz), 79.5, 49.5, 28.3, 22.7; ^{19}F NMR: (376 MHz, $CDCl_3$) δ -115.9; HRMS (ESI) calculated for $C_{13}H_{19}FNO_2$ (M+H$^+$) requires m/z 240.1400, found m/z 240.1397.

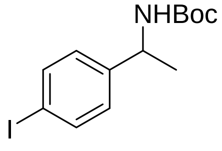

***Tert*-butyl (1-(4-iodophenyl)ethyl)carbamate (6f).**

Prepared according to the General Procedure B using 80 μL (1.06 g/mL, 0.36 mmol) of 4-iodostyrene (**1f**), 48 μL (1.165 g/ml, 0.3 mmol) of ethyl 2-diazo-2-phenylacetate (**2a**), 45 μL (0.877 g/mL, 0.36 mmol) of $PhSiH_3$, 0.0029 g (0.015 mmol) of $Co(OAc)_2$, 0.0058 g (0.018 mmol) of **L4**, 1.2 mL (0.25 M) of THF, 0.49 g (7.5 mmol) of Zn, 3 mL (0.1 M) of mixture AcOH-THF-H_2O

(3/1/1, v/v/v), 180 μL (0.95 g/mL, 0.9 mmol) of Boc_2O, 0.10 g (0.9 mmol) of Na_2CO_3 and 3 mL (0.1 M) of THF. Upon completion, the reaction was worked up, and purified by flash column chromatography using PE/Et_2O (20/1, 200 mL) with 1% of Et_3N as the eluent to give 0.0575 g (0.17 mmol, 55% yield) of the title compound as a white solid. M.p.: 140.1-141.7 °C. IR (neat): 3373, 2957, 2925, 1685, 1511, 1453, 1370 cm^{-1}. ^{1}H NMR: (400 MHz, $CDCl_3$) δ 7.64 (d, J = 8.4 Hz, 2H), 7.05 (d, J = 8.4 Hz, 2H), 4.86-4.66 (m, 2H), 1.45-1.37 (m, 12H); ^{13}C NMR: (100 MHz, $CDCl_3$) δ 154.9, 143.9, 137.5, 127.8, 92.2, 79.6, 49.7, 28.3, 22.5; HRMS (ESI) calculated for $C_{13}H_{19}INO_2$ (M+H$^+$) requires m/z 348.0460, found m/z 348.0466.

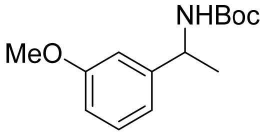

***Tert*-butyl (1-(3-methoxyphenyl)ethyl)carbamate (6o).**

Prepared according to the General Procedure B using 0.0483 g (0.36 mmol) of 3-methoxystyrene (**1o**), 48 μL (1.165 g/ml, 0.3 mmol) of ethyl 2-diazo-2-phenylacetate (**2a**), 45 μL (0.877 g/mL, 0.36 mmol) of $PhSiH_3$, 0.0028 g (0.015 mmol) of $Co(OAc)_2$, 0.0059 g (0.018 mmol) of **L4**, 1.2 mL (0.25 M) of THF, 0.49 g (7.5 mmol) of Zn, 3 mL (0.1 M) of mixture AcOH-THF-H_2O (3/1/1, v/v/v), 180 μL (0.95 g/mL, 0.9 mmol) of Boc_2O, 0.10 g (0.9 mmol) of Na_2CO_3 and 3 mL (0.1 M) of THF. Upon completion, the reaction was worked up, and purified by flash column chromatography using PE/EA (13/1, 200 mL) as the eluent to give 0.0565 g (0.225 mmol,75% yield) of the title compound as a white solid. M.p.: 59.8-60.8 °C. IR (neat): 3344, 2972, 1702, 1604, 1492, 1456 cm^{-1}. ^{1}H NMR: (400 MHz, $CDCl_3$) δ 7.28-7.20 (m, 1H), 6.89 (d, J = 7.6 Hz, 1H), 6.80-6.76 (s, 1H), 6.78 (m, 1H), 4.95-4.55 (m, 2H), 3.80 (s, 3H), 1.44 (s, 3H), 1.42 (s, 9H); ^{13}C NMR: (100 MHz, $CDCl_3$) δ 159.7, 155.0, 145.8, 129.5, 118.1, 112.3, 111.7, 79.3, 55.1, 50.1, 28.3, 22.6; HRMS (ESI) calculated for $C_{14}H_{22}NO$ (M+H$^+$) requires m/z 252.1594, found m/z 252.1606.

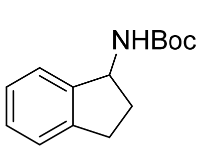

***Tert*-butyl (2,3-dihydro-1*H*-inden-1-yl)carbamate (6ak).**

Prepared according to the General Procedure B using 0.0420 g (0.36 mmol) of 1*H*-indene (**1ak**), 48 μL (1.165 g/ml, 0.3 mmol) of ethyl 2-diazo-2-phenylacetate (**2a**), 45 μL (0.877 g/mL, 0.36 mmol) of $PhSiH_3$, 0.0028 g (0.015

mmol) of $Co(OAc)_2$, 0.0059 g (0.018 mmol) of **L4**, 1.2 mL (0.25 M) of THF, 0.49 g (7.5 mmol) of Zn, 3 mL (0.1 M) of mixture AcOH-THF-H_2O (3/1/1, v/v/v), 180 μL (0.95 g/mL, 0.9 mmol) of Boc_2O, 0.10 g (0.9 mmol) of Na_2CO_3 and 3 mL (0.1 M) of THF. Upon completion, the reaction was worked up, and purified by flash column chromatography using PE/EA (20/1, 200 mL) with 1% of Et_3N as the eluent to give 0.0398 g (0.18 mmol, 60% yield) of the title compound as a white solid. M.p.: 93.5-94.5 °C. IR (neat): 3335, 2975, 1697, 1518, 1458, 1367, 1246, 1172 cm^{-1}. ^1H NMR: (400 MHz, $CDCl_3$) δ 7.34-7.30 (m, 1H), 7.23-7.19 (m, 3H), 5.23-5.13 (m, 1H), 4.75 (br, 1H), 3.00-2.91 (m, 1H), 2.88-2.78 (m, 1H), 2.62-2.52 (m, 1H), 1.84-1.75 (m, 1H), 1.49 (s, 9H); ^{13}C NMR: (100 MHz, $CDCl_3$) δ 155.7, 143.6, 143.2, 127.8, 126.6, 124.7, 124.0, 79.4, 55.9, 34.3, 30.0, 28.4; HRMS (ESI) calculated for $C_{14}H_{20}NO_2$ (M+H^+) requires m/z 234.1494, found m/z 234.1498.

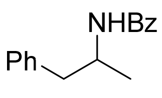

_N_-(1-phenylpropan-2-yl)benzamide (7aa).

Prepared according to the General Procedure C using 0.0422 g (0.36 mmol) of allylbenzene (**1aa**), 48 μL (1.165 g/ml, 0.3 mmol) of ethyl 2-diazo-2-phenylacetate (**2a**), 45 μL (0.877 g/mL, 0.36 mmol) of $PhSiH_3$, 0.0026 g (0.015 mmol) of $Co(OAc)_2$, 0.0058 g (0.018 mmol) of **L4**, 1.2 mL (0.25 M) of THF, 0.49 g (7.5 mmol) of Zn, 3 mL (0.1 M) of mixture AcOH-THF-H_2O (3/1/1, v/v/v), 52 μL (1.211 g/mL, 0.45 mmol) of BzCl and 84 μL (0.728 g/mL, 0.6 mmol) of Et_3N and 3 mL (0.1 M) of THF. Upon completion, the reaction was worked up, and purified by flash column chromatography using PE/EA (50/1, 200 mL) as the eluent to give 0.0381 g (0.16 mmol, 53% yield) of the title compound as a white solid. ^1H NMR: (400 MHz, $CDCl_3$) δ 7.69 (d, J = 4.8 Hz, 2H), 7.51-7.45 (m, 1H), 7.44-7.37 (m, 2H), 7.34-7.27 (m, 2H), 7.27-7.19 (m, 3H), 5.97 (d, J = 5.2 Hz, 1H), 4.55-4.40 (m, 1H), 3.00-2.80 (m, 2H), 1.22 (d, J = 6.4 Hz, 3H); ^{13}C NMR: (100 MHz, $CDCl_3$) δ 166.8, 137.8, 134.8, 131.3, 129.5, 128.5, 128.4, 126.7, 126.5, 46.4, 42.3, 19.9; The NMR spectra were consistent with the spectra reported in the literature.[24]

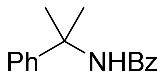

***N*-(2-phenylpropan-2-yl)benzamide (7al).**

Prepared according to the General Procedure C using 0.0430 g (0.36 mmol) of 2-phenyl-1-propene (**1al**), 48 μL (1.165 g/ml, 0.3 mmol) of ethyl 2-diazo-2-phenylacetate (**2a**), 45 μL (0.877 g/mL, 0.36 mmol) of $PhSiH_3$, 0.0029 g (0.015 mmol) of $Co(OAc)_2$, 0.0057 g (0.018 mmol) of **L4**, 1.2 mL (0.25 M) of THF, 0.49 g (7.5 mmol) of Zn, 3 mL (0.1 M) of mixture AcOH-THF-H_2O (3/1/1, v/v/v), 50 μL (1.211 g/mL, 0.45 mmol) of BzCl and 80 μL (0.728 g/mL, 0.6 mmol) of Et_3N and 3 mL (0.1 M) of THF. Upon completion, the reaction was worked up, and purified by flash column chromatography using PE/EA (50/1, 200 mL) with 1% of Et_3N as the eluent to give 0.0420 g (0.18 mmol, 59% yield) of the title compound as a white solid. ^{1}H NMR: (400 MHz, $CDCl_3$) δ 7.75 (d, J = 7.2 Hz, 2H), 7.50-7.31 (m, 7H), 7.26-7.21 (m, 1H), 6.46 (br, 1H), 1.82 (s, 6H); ^{13}C NMR: (100 MHz, $CDCl_3$) δ 166.4, 146.8, 135.3, 131.3, 128.5, 128.4, 126.8, 126.7, 124.7, 56.2, 29.1. The NMR spectra were consistent with the spectra reported in the literature.[25]

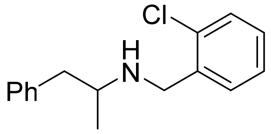

Clobenzorex

***N*-(2-chlorobenzyl)-1-phenylpropan-2-amine (8aa).**

Prepared according to the General Procedure D using 48 μL (0.892 g/mL, 0.36 mmol) of allylbenzene (**1aa**), 49 μL (1.165 g/ml, 0.3 mmol) of ethyl 2-diazo-2-phenylacetate (**2a**), 45 μL (0.877 g/mL, 0.36 mmol) of $PhSiH_3$, 0.0026 g (0.015 mmol) of $Co(OAc)_2$, 0.0059 g (0.018 mmol) of **L4**, 1.2 mL (0.25 M) of THF, 0.49 g (7.5 mmol) of Zn, 3 mL (0.1 M) of mixture AcOH-THF-H_2O (3/1/1, v/v/v), 34 μL (1.248 g/mL, 0.3 mmol) of 2-chlorobenzaldehyde, 0.0208 g (0.3 mmol) of $NaBH_3CN$, 0.25 mL (3 mmol) of HCl and 3 mL (0.1 M) of methanol. Upon completion, the worked up, and purified by flash column chromatography using PE/EA (50/1, 200 mL) with 1% of Et_3N as the eluent to give 0.0330 g (0.13 mmol, 42% yield) of the title compound as a colorless oil. ^{1}H NMR: (400 MHz, $CDCl_3$) δ 7.32-7.24 (m, 4H), 7.24-7.10 (m, 5H), 3.91 (d, J = 14.0 Hz, 1H), 3.83 (d, J = 14.0 Hz, 1H), 2.97-2.85 (m, 1H), 2.76 (dd, J = 13.6, 7.2 Hz, 1H), 2.66 (dd, J = 13.6, 6.4 Hz, 1H), 1.12 (d, J = 6.4 Hz, 3H); ^{13}C NMR: (100 MHz, $CDCl_3$) δ 139.3, 137.7, 133.7, 130.1, 129.4, 129.2, 128.3,

128.2, 126.7, 126.2, 53.6, 48.8, 43.6, 20.2; The NMR spectra were consistent with the spectra reported in the literature.[26]

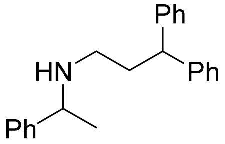

(+/-)-Fendiline

3,3-diphenyl-*N*-(1-phenylethyl)propan-1-amine (8b).

Prepared according to the procedure **c** using 43 µL (0.906 g/mL, 0.36 mmol) of styrene (**1b**), 49 µL (1.165 g/ml, 0.3 mmol) of ethyl 2-diazo-2-phenylacetate (**2a**), 45 µL (0.877 g/mL, 0.36 mmol) of $PhSiH_3$, 0.0029 g (0.015 mmol) of $Co(OAc)_2$, 0.0060 g (0.019 mmol) of **L4**, 1.2 mL (0.25 M) of THF, 0.49 g (7.5 mmol) of Zn, 3 mL (0.1 M) of mixture AcOH-THF-H_2O (3/1/1, v/v/v), 0.0674 (0.3 mmol) of 3,3-diphenylpropanal, 0.0190 g (0.3 mmol) of $NaBH_3CN$, 0.25 mL (3 mmol) of concentrated hydrochloric acid and 3 mL (0.1 M) of methanol. Upon completion, the reaction was worked up, and purified by thin-layer chromatography using PE/EA (10/1, 100 mL) as the eluent to give 0.0333 g (0.11 mmol, 35% yield) of the title compound as a colorless oil. ^{1}H NMR: (400 MHz, $CDCl_3$) δ 7.50-7.00 (m, 15H), 3.96 (t, J = 8.0 Hz, 1H), 3.67 (q, J = 6.4 Hz, 1H), 2.52-2.36 (m, 2H), 2.30-2.10 (m, 2H), 1.62 (br, 1H) 1.28 (d, J = 6.4 Hz, 3H); ^{13}C NMR: (100 MHz, $CDCl_3$) δ 145.6, 144.9, 144.7, 128.4, 128.3, 127.8, 127.7, 126.8, 126.5, 126.1, 58.1, 49.0, 46.0, 36.0, 24.2; The NMR spectra were consistent with the spectra reported in the literature.[27]

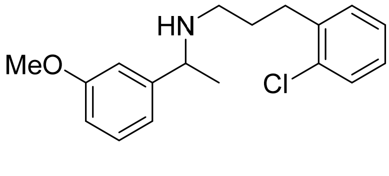

(+/-)-NPS *R*-568

3-(2-chlorophenyl)-*N*-(1-(3-methoxyphenyl)ethyl)propan-1-amine (8o).

Prepared according to the procedure **c** using 0.0483 g (0.36 mmol) of 1-methoxy-3-vinylbenzene (**1o**), 49 µL (1.165 g/ml, 0.3 mmol) of ethyl 2-diazo-2-phenylacetate (**2a**), 45 µL (0.877 g/mL, 0.36 mmol) of $PhSiH_3$, 0.0029 g (0.015 mmol) of $Co(OAc)_2$, 0.0059 g (0.018 mmol) of **L4**, 1.2 mL (0.25 M) of THF, 0.49 g (7.5 mmol) of Zn, 3 mL (0.1 M) of mixture AcOH-THF-H_2O (3/1/1, v/v/v), 0.0537 g (0.3 mmol) of 3-(2-chlorophenyl)propanal, 0.0208 g (0.3 mmol) of $NaBH_3CN$, 0.25 mL (3 mmol) of concentrated hydrochloric acid and 3 mL (0.1 M) of methanol. Upon completion, the

reaction was worked up, and purified by thin-layer chromatography using PE/EA (10/1, 100 mL) as the eluent to give 0.0290 g (0.10 mmol, 32% yield) of the title compound as a colorless oil. ^{1}H NMR: (400 MHz, $CDCl_3$) δ 7.32-7.22 (m, 2H), 7.19-7.06 (m, 3H), 6.97-6.90 (m, 2H), 6.83-6.77 (m, 1H), 3.82 (m, 4H), 3.07 (br, 1H), 2.80-2.63 (m, 2H), 2.63-2.49 (m, 2H), 1.94-1.76 (m, 2H), 1.43 (d, J = 6.8 Hz, 3H); ^{13}C NMR: (100 MHz, $CDCl_3$) δ 159.8, 139.3, 133.8, 130.2, 129.5, 129.4, 127.3, 126.7, 119.1, 112.7, 112.1, 58.3, 55.2, 46.8, 31.1, 29.3, 23.6; The NMR spectra were consistent with the spectra reported in the literature.[28]

<u>**Asymmetric Hydroamination of Alkenes**</u>

General Procedure E for Asymmetric Hydroamination of Alkenes:

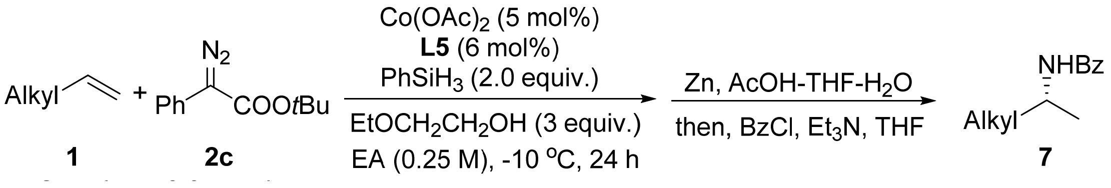

Step E1. A 25 mL Schlenk flask equipped with a magnetic stirrer and a flanging rubber plug was dried with flame under vacuum. When cooled to ambient temperature, it was vacuumed and flushed with N_2 and repeated for three times. To the flask, $Co(OAc)_2$ (0.015 mmol), **L5** (0.018 mol), EA (1.2 mL), 2-ethylethanol 100 μL, 0.93 g/mL, 0.9 mmol) were added. The flask was degassed and cooled down to -10 °C and stirred for 30 min. Then, $PhSiH_3$ (0.36 mol), diazo compound (0.3 mmol) and alkene (0.36 mmol) were added in sequence. After 24 h, the reaction was warmed up to room temperature and quenched with 10 ml of PE and the mixture was filtered through a pad of silica gel and washed with PE/EA (5/1, 50 mL). The combined filtrates were concentrated to afford a yellow oil.

Step E2 was the same as **Step B2** in **General Procedure B**.

Step E3 was the same as **Step C3** in **General Procedure C**.

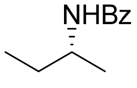

(*R*)-*N*-(*sec*-butyl)benzamide (9)

Prepared according to the general procedure E, using 0.3370 g (10 w% in hexane, 0.6 mmol) of 1-butene (**1x**), 58 μL (1.129 g/ml, 0.3 mmol) of *tert*-butyl 2-diazo-2-phenylacetate (**2c**), 74 μL (0.877 g/mL, 0.6 mmol) of $PhSiH_3$, 100 μL (0.93 g/mL, 0.9 mmol) of 2-ethoxyethanol, 0.0029 g (0.015 mmol) of $Co(OAc)_2$, 0.0079 g (0.018 mmol) of **L5**, 1.2 mL (0.25 M) of EA, and 0.49 g (7.5 mmol) of Zn, 3 mL (0.1 M) of mixture AcOH-THF-H_2O (3/1/1, v/v/v), 85 μL (0.728 g/mL, 0.6 mmol) of Et_3N, 40 μL (0.45 mmol) of BzCl and 3 mL (0.1 M) of THF. The title compound (0.0354 g, 67% yield, 89.0/11.0 *er*) was obtained as a white solid after silica-gel column chromatography (PE/EA = 5/1, 500 mL).

Recrystallization from the hexane/DCM was then performed as follows. The title compound (0.0354 g, 0.200 mmol, 89.0/11.0 er) was dissolved in minimum DCM, followed by a few drops of hexane. Colorless needles were appeared on standing, which were collected by filtration and washed with hexane. The same procedure was used for a second recrystallization, furnishing the title compound (0.0140 g, 0.079 mmol, 40%, 97.3/2.7 er) as a courless needle. Optical Rotation: $[\alpha]^{20}_{D}$ = -25.0 (c 0.70, $CHCl_3$). *lit.* $[\alpha]^{19}_{D}$ = +12.5 (c 1, $CHCl_3$ (*S*)).[29]

The *er* was determined by HPLC, HPLC conditions: Chiralcel OD-H, *n*-hexane/*i*-PrOH = 98/2, flow rate = 1.0 mL/min, n = 254 nm, retention times (t_r) = 24.9 min (major) and 32.2 min (minor).

^{1}H NMR: (400 MHz, $CDCl_3$) δ 7.79-7.71 (m, 2H), 7.52-7.45 (m, 1H), 7.45-7.38 (m, 2H), 5.98 (br, 1H), 4.20-4.04 (m, 1H), 1.63-1.52 (m, 2H), 1.23 (d, J = 6.8 Hz, 3H), 0.96 (t, J = 7.2 Hz, 3H); ^{13}C NMR: (100 MHz, $CDCl_3$) δ 166.9, 135.0, 131.2, 128.5, 126.8, 47.1, 29.7, 20.5, 10.4; The NMR spectra were consistent with the spectra reported in the literature.[29]

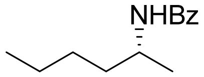

(*R*)-*N*-(hexan-2-yl)benzamide (10)

Prepared according to the general procedure E**,** using 74 μL (0.678 g/mL, 0.6 mmol) of 1-hexene (**1ar**), 58 μL (1.129 g/ml, 0.3 mmol) of *tert*-butyl 2-diazo-2-phenylacetate (**2c**), 74 μL (0.877 g/mL, 0.6 mmol) of $PhSiH_3$, 100 μL (0.93 g/mL, 0.9 mmol) of 2-ethoxyethanol, 0.0028 g (0.015 mmol) of $Co(OAc)_2$, 0.0079 g (0.018 mmol) of **L5**, 1.2 mL (0.25 M) of EA, and 0.49 g (7.5 mmol) of Zn, 3 mL (0.1 M) of mixture AcOH-THF-H_2O (3/1/1, v/v/v), 85 μL (0.728 g/mL, 0.6 mmol) of Et_3N, 40 μL (0.45 mmol) of BzCl and 3 mL (0.1 M) of THF. The resulting suspension was added 0.1 ml of TFA and 1 mL of DCM. The mixture was stirred overnight for hydrolyzsis of side product (*tert*-butyl 2-benzamido-2-phenylacetate). Then the mixture was concentrated. The title compound (0.0372 g, 60% yield, 90.5/9.5 *er*) was obtained as a white solid after silica-gel column chromatography (PE/EA = 10/1, 500 mL). Optical Rotation: $[\alpha]^{20}_{D}$ = -15.3 (c 1.000, $CHCl_3$).

The *er* was determined by HPLC, HPLC conditions: Chiralcel OD-H, *n*-hexane/*i*-PrOH

= 98/2, flow rate = 1.0 mL/min, n = 254 nm, retention times (t_r) = 20.8 min (major) and 26.5 min (minor).

M.p.: 81.2-83.3 °C. IR (neat): 3303, 2956, 2928, 2856, 1633, 1538, 1462 1312 cm^{-1}. ^{1}H NMR: (400 MHz, $CDCl_3$) δ 7.79-7.72 (m, 2H), 7.52-7.45 (m, 1H), 7.45-7.38 (m, 2H), 6.00 (d, J = 7.2 Hz, 1H), 4.26-4.12 (m, 1H), 1.62-1.46 (m, 2H), 1.42-1.30 (m, 4H), 1.23 (d, J = 6.8 Hz, 3H), 0.90 (t, J = 6.8 Hz, 3H); ^{13}C NMR: (100 MHz, $CDCl_3$) δ 166.8, 135.0, 131.2, 128.5, 126.8, 45.7, 36.7, 28.2, 22.6, 21.0, 14.0; HRMS (ESI) calculated for $C_{13}H_{19}NNaO$ ($M+Na^+$) requires m/z 228.1359, found m/z 288.1358.

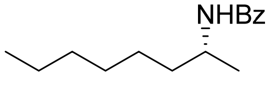

(*R*)-*N*-(octan-2-yl)benzamide (11).

Prepared according to the general procedure E**,** using 103 μL (0.715 g/mL, 0.6 mmol) of 1-octene (**1y**), 58 μL (1.129 g/ml, 0.3 mmol) of *tert*-butyl 2-diazo-2-phenylacetate (**2c**), 74 μL (0.877 g/mL, 0.6 mmol) of $PhSiH_3$, 100 μL (0.93 g/mL, 0.9 mmol) of 2-ethoxyethanol, 0.0028 g (0.015 mmol) of $Co(OAc)_2$, 0.0079 g (0.018 mmol) of **L5**, 1.2 mL (0.25 M) of EA, and 0.49 g (7.5 mmol) of Zn, 3 mL (0.1 M) of mixture AcOH-THF-H_2O (3/1/1, v/v/v), 85 μL (0.728 g/mL, 0.6 mmol) of Et_3N, 40 μL (0.45 mmol) of BzCl and 3 mL (0.1 M) of THF. The resulting suspension was added 0.1 ml of TFA and 1 mL of DCM. The mixture was stirred overnight for hydrolyzsis of side product (*tert*-butyl 2-benzamido-2-phenylacetate). Then the mixture was concentrated. The title compound (0.0442 g, 63% yield, 92.5:7.5 *er*) was obtained as a white solid after silica-gel column chromatography (PE/EA = 10/1, 500 mL).

Recrystallization from the hexane/DCM was then performed as follows. The title compound (0.0442 g, 0.19 mmol, 92.5:7.5 *er*) was dissolved in minimum DCM, followed by a few drops of hexane. Colorless needles were appeared on standing, which were collected by filtration and washed with hexane, furnishing the title compound (0.0328 g, 0.141mmol, 97.5:2.5 *er*) as a courless needle. Optical Rotation: $[\alpha]^{20}_{D}$ = -18.1 (c 1.05, $CHCl_3$). *lit.* $[\alpha]^{25}_{D}$ = -15.7 (c 3.55, $CHCl_3$ (*R*)).[30]

The *er* was determined by HPLC, HPLC conditions: Chiralcel OJ-H, *n*-hexane/*i*-PrOH = 99/1, flow rate = 1.0 mL/min, n = 254 nm, retention times (t_r) = 33.7 min (major) and 35.3 min (minor).

^{1}H NMR: (400 MHz, $CDCl_3$) δ 7.75 (d, J = 8.4 Hz, 2H), 7.53-7.46 (m, 1H), 7.46-7.39 (m, 2H), 5.85 (d, J = 6.4 Hz, 1H), 4.26-4.13 (m, 1H), 1.57-1.47 (m, 2H), 1.42-1.25 (m, 8H), 1.24 (d, J = 6.8 Hz, 3H), 0.88 (t, J = 6.4 Hz, 3H); ^{13}C NMR: (100 MHz, $CDCl_3$) δ 166.8, 135.1, 131.2, 128.5, 126.8, 45.8, 37.1, 31.8, 29.2, 26.0, 22.6, 21.1, 14.0;
The NMR spectra were consistent with the spectra reported in the literature.[31]

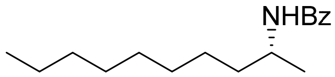

(*R*)-*N*-(decan-2-yl)benzamide (12)

Prepared according to the general procedure E**,** using 113 μL (0.741 g/mL, 0.6 mmol) of 1-decene (**1as**), 58 μL (1.129 g/ml, 0.3 mmol) of *tert*-butyl 2-diazo-2-phenylacetate (**2c**), 74 μL (0.877 g/mL, 0.6 mmol) of $PhSiH_3$, 100 μL (0.93 g/mL, 0.9 mmol) of 2-ethoxyethanol, 0.0028 g (0.015 mmol) of $Co(OAc)_2$, 0.0078 g (0.018 mmol) of **L5**, 1.2 mL (0.25 M) of EA, and 0.49 g (7.5 mmol) of Zn, 3 mL (0.1 M) of mixture AcOH-THF-H_2O (3/1/1, v/v/v), 85 μL (0.728 g/mL, 0.6 mmol) of Et_3N, 40 μL (0.45 mmol) of BzCl and 3 mL (0.1 M) of THF. The resulting suspension was added 0.1 ml of TFA and 1 mL of DCM. The mixture was stirred overnight for hydrolyzsis of side product (*tert*-butyl 2-benzamido-2-phenylacetate). Then the mixture was concentrated. The title compound (0.0608 g, 78% yield, 92.2/7.8 *er*) was obtained as a white solid after silica-gel column chromatography (PE/EA = 7/1, 500 mL). Optical Rotation: $[\alpha]^{20}_D$ = -14.0 (c 0.990, $CHCl_3$).
The *er* was determined by HPLC, HPLC conditions: Chiralcel OD-H, *n*-hexane/*i*-PrOH = 98/2, flow rate = 1.0 mL/min, n = 254 nm, retention times (t_r) = 22.0 min (minor) and 23.3 min (major).
M.p.: 97.9-99.3 °C. IR (neat): 3287, 2957, 2920, 2850, 1633, 1541, 1461, 1351, 1312 cm^{-1}. ^{1}H NMR: (400 MHz, $CDCl_3$) δ 7.79-7.72 (m, 2H), 7.52-7.45 (m, 1H), 7.45-7.38 (m, 2H), 5.96 (d, J = 8.0 Hz, 1H), 4.25-4.10 (m, 1H), 1.62-1.45 (m, 2H), 1.45-1.24 (m, 12H), 1.23 (d, J = 6.8 Hz, 3H), 0.87 (t, J = 6.8 Hz, 3H); ^{13}C NMR: (100 MHz, $CDCl_3$) δ 166.8, 135.1, 131.2, 128.5, 126.8, 45.7, 37.0, 31.8, 29.50, 29.49, 29.2, 26.1, 22.6, 21.0, 14.1; HRMS (ESI) calculated for $C_{17}H_{27}NNaO$ (M+Na$^+$) requires m/z 284.1985, found m/z 284.1986.

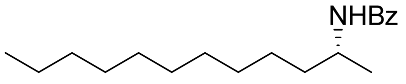

(*R*)-*N*-(dodecan-2-yl)benzamide (13)

Prepared according to the general procedure E**,** using 0.1034 g (0.6 mmol) of 1-dodecene (**1at**), 58 μL (1.129 g/ml, 0.3 mmol) of *tert*-butyl 2-diazo-2-phenylacetate (**2c**), 74 μL (0.877 g/mL, 0.6 mmol) of $PhSiH_3$, 100 μL (0.93 g/mL, 0.9 mmol) of 2-ethoxyethanol, 0.0027 g (0.015 mmol) of $Co(OAc)_2$, 0.0077 g (0.018 mmol) of **L5**, 1.2 mL (0.25 M) of EA, and 0.49 g (7.5 mmol) of Zn, 3 mL (0.1 M) of mixture AcOH-THF-H_2O (3/1/1, v/v/v), 85 μL (0.728 g/mL, 0.6 mmol) of Et_3N, 40 μL (0.45 mmol) of BzCl and 3 mL (0.1 M) of THF. The resulting suspension was added 0.1 ml of TFA and 1 mL of DCM. The mixture was stirred overnight for hydrolyzsis of side product (*tert*-butyl 2-benzamido-2-phenylacetate). Then the mixture was concentrated. The title compound (0.0513 g, 59% yield, 92.3/7.7 *er*) was obtained as a white solid after silica-gel column chromatography (PE/EA = 10/1, 500 mL). Optical Rotation: $[\alpha]^{20}_{D}$ = -9.4 (c 1.015, $CHCl_3$).

The *er* was determined by HPLC, HPLC conditions: Chiralcel OJ-H, *n*-hexane/*i*-PrOH = 99/1, flow rate = 1.0 mL/min, n = 254 nm, retention times (t_r) = 13.0 min (minor) and 14.8 min (major).

M.p.: 102.3-103.2 °C. IR (neat): 3286, 2957, 2919, 2850, 1634, 1542, 1464, 1313 cm^{-1}. ^1H NMR: (400 MHz, $CDCl_3$) δ 7.78-7.71 (m, 2H), 7.51-7.45 (m, 1H), 7.45-7.38 (m, 2H), 5.92 (d, J = 8.0 Hz, 1H), 4.25-4.10 (m, 1H), 1.60-1.45 (m, 2H), 1.44-1.25 (m, 16H), 1.23 (d, J = 6.8 Hz, 3H), 0.88 (t, J = 6.8 Hz, 3H); ^{13}C NMR: (100 MHz, $CDCl_3$) δ 166.8, 135.1, 131.2, 128.5, 126.8, 45.8, 37.1, 31.9, 29.6, 29.54, 29.51, 29.3, 26.1, 22.6, 21.0, 14.1; HRMS (ESI) calculated for $C_{19}H_{32}NO$ (M+H^+) requires m/z 290.2478, found m/z 290.2479.

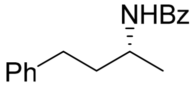

(*R*)-*N*-(4-phenylbutan-2-yl)benzamide (14).

Prepared according to the general procedure E**,** using 88 μL (0.88 g/mL, 0.6 mmol) of 4-phenyl-1-butene (**1z**), 58 μL (1.129 g/ml, 0.3 mmol) of *tert*-butyl 2-diazo-2-phenylacetate (**2c**), 74 μL (0.877 g/mL, 0.6 mmol) of $PhSiH_3$, 100 μL (0.93 g/mL, 0.9 mmol) of 2-ethoxyethanol, 0.0029 g (0.015 mmol) of $Co(OAc)_2$, 0.0079 g (0.018 mmol) of **L5**, 1.2 mL (0.25 M) of EA, and 0.49 g (7.5 mmol) of Zn, 3 mL (0.1

M) of mixture AcOH-THF-H_2O (3/1/1, v/v/v), 85 μL (0.728 g/mL, 0.6 mmol) of Et_3N, 40 μL (0.45 mmol) of BzCl and 3 mL (0.1 M) of THF. The title compound (0.0362 g, 48% yield, 90.0:10.0 er) was obtained as a white solid after silica-gel column chromatography (PE/EA = 10/1, 500 mL).

Recrystallization from the hexane/DCM was then performed as follows. The title compound (0.0362 g, 0.144 mmol, 90.0:10.0 *er*) was dissolved in minimum DCM, followed by a few drops of hexane. Colorless needles were appeared on standing, which were collected by filtration and washed with hexane. The same procedure was used for a second recrystallization, furnishing the title compound (0.0326 g, 0.13 mmol, 89%, 98.5.:1.5 *er*) as a courless needle. Optical Rotation: $[\alpha]^{20}_D$ = -7.1 (c 1.05, CH_2Cl_2). *lit.* $[\alpha]^{25}_D$ = 6 (c 0.25, CH_2Cl_2, 10.4:89.6 *er*, (*S*)).[32]

The *er* was determined by HPLC, HPLC conditions: Chiralcel OJ-H, *n*-hexane/*i*-PrOH = 85/15, flow rate = 1.0 mL/min, n = 254 nm, retention times (t_r) = 10.3 min (major) and 16.1 min (minor).

^{1}H NMR: (400 MHz, $CDCl_3$) δ 7.68 (d, *J* = 8.0 Hz, 2H), 7.53-7.45 (m, 1H), 7.45-7.37 (m, 2H), 7.31-7.24 (m, 2H), 7.24-7.14 (m, 3H), 5.86 (d, *J* = 7.2 Hz, 1H), 4.36-4.22 (m, 1H), 2.74 (t, *J* = 8.0 Hz, 2H), 1.90 (dt, *J* = 8.0, 7.2 Hz, 2H), 1.29 (d, *J* = 6.4 Hz, 3H); ^{13}C NMR: (100 MHz, $CDCl_3$) δ 166.8, 141.7, 134.9, 131.3, 128.5, 128.3, 126.8, 125.9, 45.7, 38.6, 32.5, 21.1; The NMR spectra were consistent with the spectra reported in the literature.[32]

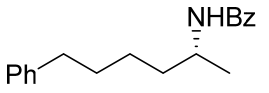

(*R*)-*N*-(6-phenylhexan-2-yl)benzamide (15)

Prepared according to the general procedure E**,** using 0.0953 g (0.6 mmol) of hex-5-en-1-ylbenzene (**1au**), 58 μL (1.129 g/ml, 0.3 mmol) of *tert*-butyl 2-diazo-2-phenylacetate (**2c**), 74 μL (0.877 g/mL, 0.6 mmol) of $PhSiH_3$, 100 μL (0.93 g/mL, 0.9 mmol) of 2-ethoxyethanol, 0.0028 g (0.015 mmol) of $Co(OAc)_2$, 0.0078 g (0.018 mmol) of **L5**, 1.2 mL (0.25 M) of EA, and 0.49 g (7.5 mmol) of Zn, 3 mL (0.1 M) of mixture AcOH-THF-H_2O (3/1/1, v/v/v), 85 μL (0.728 g/mL, 0.6 mmol) of Et_3N, 40 μL (0.45 mmol) of BzCl and 3 mL (0.1 M) of THF. The title compound (0.0628 g, 74% yield, 92.6/7.4 er) was obtained as a white solid after silica-gel column

chromatography (PE/EA = 10/1, 1000 mL). Optical Rotation: $[\alpha]^{20}_{D}$ = -5.0 (c 0.995, $CHCl_3$).

The *er* was determined by HPLC, HPLC conditions: Chiralcel AD-H, *n*-hexane/*i*-PrOH = 96/4, flow rate = 0.5 mL/min, n = 254 nm, retention times (t_r) = 37.2 min (minor) and 38.6 min (major).

M.p.: 86.7-88.2 °C. IR (neat): 3308, 3027, 2930, 2856, 1633, 1538, 1456, 1309 cm^{-1}. ^1H NMR: (400 MHz, $CDCl_3$) δ 7.77-7.68 (m, 2H), 7.53-7.46 (m, 1H), 7.46-7.39 (m, 2H), 7.30-7.22 (m, 2H), 7.20-7.13 (m, 3H), 5.84 (br, 1H), 4.26-4.13 (m, 1H), 2.62 (t, *J* = 8.0 Hz, 2H), 1.74-1.52 (m, 6H), 1.48-1.36 (m, 2H), 1.23 (d, *J* = 6.8 Hz, 3H); ^{13}C NMR: (100 MHz, $CDCl_3$) δ 166.8, 142.4, 135.0, 131.3, 128.5, 128.4, 128.3, 126.8, 125.7, 45.7, 36.9, 35.7, 31.2, 25.6, 21.0; HRMS (ESI) calculated for $C_{19}H_{24}NO$ (M+H$^+$) requires m/z 282.1852, found m/z 282.1852.

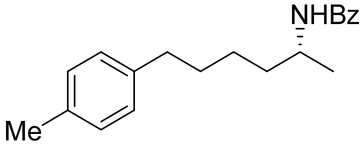

(*R*)-*N*-(6-(*p*-tolyl)hexan-2-yl)benzamide (16)

Prepared according to the general procedure E**,** using 0.1048 g (0.6 mmol) of 1-(hex-5-en-1-yl)-4-methylbenzene (**1av**), 58 µL (1.129 g/ml, 0.3 mmol) of *tert*-butyl 2-diazo-2-phenylacetate (**2c**), 74 µL (0.877 g/mL, 0.6 mmol) of $PhSiH_3$, 100 µL (0.93 g/mL, 0.9 mmol) of 2-ethoxyethanol, 0.0027 g (0.015 mmol) of $Co(OAc)_2$, 0.0079 g (0.018 mmol) of **L5**, 1.2 mL (0.25 M) of EA, and 0.49 g (7.5 mmol) of Zn, 3 mL (0.1 M) of mixture AcOH-THF-H_2O (3/1/1, v/v/v), 85 µL (0.728 g/mL, 0.6 mmol) of Et_3N, 40 µL (0.45 mmol) of BzCl and 3 mL (0.1 M) of THF. The title compound (0.0481 g, 54% yield, 91.6/8.4 er) was obtained as a white solid after silica-gel column chromatography (PE/EA = 8/1, 1000 mL). Optical Rotation: $[\alpha]^{20}_{D}$ = -7.8 (c 0.985, $CHCl_3$).

The *er* was determined by HPLC, HPLC conditions: Chiralcel OD-H, *n*-hexane/*i*-PrOH = 90/10, flow rate = 1.0 mL/min, n = 254 nm, retention times (t_r) = 12.9 min (minor) and 13.8 min (major).

M.p.: 120.4-121.4 °C. IR (neat): 3328, 2967, 2931, 2855, 1632, 1529, 1454, 1314 cm^{-1}. ^1H NMR: (400 MHz, $CDCl_3$) δ 7.76-7.70 (m, 2H), 7.51-7.45 (m, 1H), 7.45-7.38 (m, 2H), 7.10-7.01 (m, 4H), 5.92 (d, 1H), 4.25-4.12 (m, 1H), 2.57 (t, *J* = 7.6 Hz, 2H), 2.30

(s, 3H), 1.70-1.50 (m, 4H), 1.46-1.32 (m, 2H), 1.22 (d, *J* = 6.8 Hz, 3H); ^{13}C NMR: (100 MHz, $CDCl_3$) *δ* 166.8, 139.3, 135.04, 135.03, 131.2, 128.9, 128.5, 128.2, 126.8, 45.7, 36.8, 35.2, 31.3, 25.6, 21.0, 20.9; HRMS (ESI) calculated for $C_{20}H_{25}NNaO$ (M+Na$^+$) requires m/z 318.1828, found m/z 318.1827.

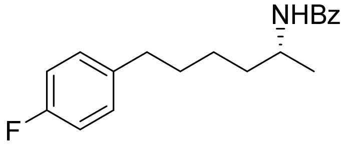

(*R*)-*N*-(6-(4-fluorophenyl)hexan-2-yl)benzamide (17)

Prepared according to the general procedure E**,** using 0.1067 g (0.6 mmol) of 1-fluoro-4-(hex-5-en-1-yl)benzene (**1aw**), 58 μL (1.129 g/ml, 0.3 mmol) of *tert*-butyl 2-diazo-2-phenylacetate (**2c**), 74 μL (0.877 g/mL, 0.6 mmol) of $PhSiH_3$, 100 μL (0.93 g/mL, 0.9 mmol) of 2-ethoxyethanol, 0.0027 g (0.015 mmol) of $Co(OAc)_2$, 0.0079 g (0.018 mmol) of **L5**, 1.2 mL (0.25 M) of EA, and 0.49 g (7.5 mmol) of Zn, 3 mL (0.1 M) of mixture AcOH-THF-H_2O (3/1/1, v/v/v), 85 μL (0.728 g/mL, 0.6 mmol) of Et_3N, 40 μL (0.45 mmol) of BzCl and 3 mL (0.1 M) of THF. The title compound (0.0577 g, 64% yield, 91.8/8.2 er) was obtained as a white solid after silica-gel column chromatography (PE/EA = 10/1, 1000 mL). Optical Rotation: $[\alpha]^{20}_{D}$ = -10.1 (c 0.985, $CHCl_3$).

The *er* was determined by HPLC, HPLC conditions: Chiralcel OD-H, *n*-hexane/*i*-PrOH = 90/10, flow rate = 1.0 mL/min, n = 254 nm, retention times (t_r) = 15.2 min (minor) and 16.6 min (major).

M.p.: 107.3-108.5 °C. IR (neat): 3306, 2930, 2856, 1632, 1538, 1511, 1311 cm^{-1}. ^{1}H NMR: (400 MHz, $CDCl_3$) *δ* 7.77-7.69 (m, 2H), 7.51-7.45 (m, 1H), 7.44-7.37 (m, 2H), 7.09 (dd, *J* = 8.8, 6.0 Hz, 2H), 6.98-6.87 (m, 2H), 5.93 (d, *J* = 8.0 Hz, 1H), 4.25-4.24 (m, 1H), 2.57 (t, *J* = 7.6 Hz, 2H), 1.70-1.50 (m, 4H), 1.46-1.34 (m, 2H), 1.21 (d, *J* = 6.8 Hz, 3H); ^{13}C NMR: (100 MHz, $CDCl_3$) *δ* 166.8, 161.2 (d, *J* = 243.3 Hz), 137.9 (d, *J* = 3.7 Hz), 135.0, 131.2, 129.6, (d, *J* = 7.6 Hz), 128.5, 126.8, 114.9 (d, *J* = 20.9 Hz), 45.6, 36.8, 34.9, 31.2, 25.5, 21.0; ^{19}F NMR: (376 MHz, $CDCl_3$) *δ* -118.0. HRMS (ESI) calculated for $C_{19}H_{22}FNNaO$ (M+H$^+$) requires m/z 322.1578, found m/z 332.1579.

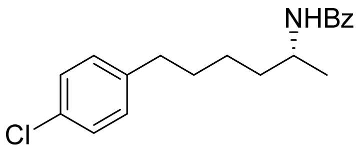

(*R*)-*N*-(6-(4-chlorophenyl)hexan-2-yl)benzamide (18)

Prepared according to the general procedure E, using 0.1162 g (0.6 mmol) of 1-chloro-4-(hex-5-en-1-yl)benzene (**1ax**), 58 μL (1.129 g/ml, 0.3 mmol) of *tert*-butyl 2-diazo-2-phenylacetate (**2c**), 74 μL (0.877 g/mL, 0.6 mmol) of $PhSiH_3$, 100 μL (0.93 g/mL, 0.9 mmol) of 2-ethoxyethanol, 0.0028 g (0.015 mmol) of $Co(OAc)_2$, 0.0079 g (0.018 mmol) of **L5**, 1.2 mL (0.25 M) of EA, and 0.49 g (7.5 mmol) of Zn, 3 mL (0.1 M) of mixture AcOH-THF-H_2O (3/1/1, v/v/v), 85 μL (0.728 g/mL, 0.6 mmol) of Et_3N, 40 μL (0.45 mmol) of BzCl and 3 mL (0.1 M) of THF. The title compound (0.0675 g, 71% yield, 91.5/8.5 er) was obtained as a white solid after silica-gel column chromatography (PE/EA = 10/1, 1200 mL). Optical Rotation: $[\alpha]^{20}_D$ = -8.7 (c 1.010, $CHCl_3$).

The *er* was determined by HPLC, HPLC conditions: Chiralcel OD-H, *n*-hexane/*i*-PrOH = 90/10, flow rate = 1.0 mL/min, n = 254 nm, retention times (t_r) = 16.1 min (minor) and 18.6 min (major).

M.p.: 107.2-109.0 °C. IR (neat): 3298, 2930, 2856, 1630, 1536, 1491, 1311 cm^{-1}. ^1H NMR: (400 MHz, $CDCl_3$) δ 7.77-7.69 (m, 2H), 7.51-7.45 (m, 1H), 7.45-7.37 (m, 2H), 7.20 (d, J = 8.4 Hz, 2H), 7.07 (d, J = 8.4 Hz, 2H), 5.94 (d, J = 8.0 Hz, 1H), 4.26-4.11 (m, 1H), 2.57 (t, J = 7.6 Hz, 2H), 1.71-1.50 (m, 4H), 1.45-1.34 (m, 2H), 1.21 (d, J = 6.8 Hz, 3H); ^{13}C NMR: (100 MHz, $CDCl_3$) δ 166.8, 140.8, 134.9, 131.3, 131.2, 129.7, 128.5, 128.3, 126.8, 45.5, 36.7, 35.0, 31.0, 25.4, 21.0; HRMS (ESI) calculated for $C_{19}H_{22}ClNNaO$ (M+Na^+) requires m/z 338.1282, found m/z 338.1279.

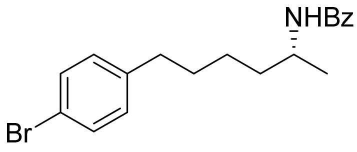

(*R*)-*N*-(6-(4-bromophenyl)hexan-2-yl)benzamide (19)

Prepared according to the general procedure E, using 0.1445 g (0.6 mmol) of 1-bromo-4-(hex-5-en-1-yl)benzene (**1ay**), 58 μL (1.129 g/ml, 0.3 mmol) of *tert*-butyl 2-diazo-2-phenylacetate (**2c**), 74 μL (0.877 g/mL, 0.6 mmol) of $PhSiH_3$, 100 μL (0.93 g/mL, 0.9 mmol) of 2-ethoxyethanol, 0.0028 g (0.015 mmol) of $Co(OAc)_2$, 0.0077 g (0.018 mmol) of **L5**, 1.2 mL (0.25 M) of EA, and 0.49 g (7.5 mmol) of Zn, 3 mL (0.1 M) of mixture AcOH-THF-H_2O (3/1/1, v/v/v), 85 μL (0.728 g/mL, 0.6 mmol) of Et_3N, 40 μL (0.45 mmol) of

BzCl and 3 mL (0.1 M) of THF. The title compound (0.0657 g, 61% yield, 91.6/8.4 er) was obtained as a white solid after silica-gel column chromatography (PE/EA = 10/1, 1000 mL). Optical Rotation: $[\alpha]^{20}_{D}$ = -7.8 (c 1.000, $CHCl_3$).

The *er* was determined by HPLC, HPLC conditions: Chiralcel OD-H, *n*-hexane/*i*-PrOH = 90/10, flow rate = 1.0 mL/min, n = 254 nm, retention times (t_r) = 16.9 min (minor) and 19.8 min (major).

M.p.: 108.7-110.3 °C. IR (neat): 3307, 2930, 2856, 1630, 1533, 1489, 1309 cm^{-1}. ^1H NMR: (400 MHz, $CDCl_3$) δ 7.77-7.69 (m, 2H), 7.52-7.45 (m, 1H), 7.45-7.37 (m, 2H), 7.35 (d, J = 8.0 Hz, 2H), 7.02 (d, J = 8.0 Hz, 2H), 5.92 (d, J = 8.0 Hz, 1H), 4.26-4.21 (m, 1H), 2.55 (t, J = 7.6 Hz, 2H), 1.70-1.50 (m, 4H), 1.45-1.35 (m, 2H), 1.21 (d, J = 6.8 Hz, 3H); ^{13}C NMR: (100 MHz, $CDCl_3$) δ 166.8, 141.3, 134.9, 131.2, 130.1, 128.5, 126.7, 119.3, 45.5, 36.7, 35.1, 30.9, 25.4, 21.0; HRMS (ESI) calculated for $C_{19}H_{23}BrNO$ (M+H^+) requires m/z 360.0958, found m/z 360.0956.

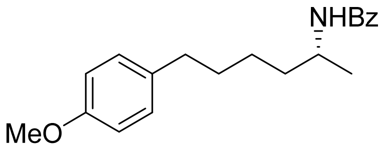

(*R*)-*N*-(6-(4-methoxyphenyl)hexan-2-yl)benzamide (20)

Prepared according to the general procedure E**,** using 0.1135 g (0.6 mmol) of 1-(hex-5-en-1-yl)-4-methoxybenzene (**1az**), 58 μL (1.129 g/ml, 0.3 mmol) of *tert*-butyl 2-diazo-2-phenylacetate (**2c**), 74 μL (0.877 g/mL, 0.6 mmol) of $PhSiH_3$, 100 μL (0.93 g/mL, 0.9 mmol) of 2-ethoxyethanol, 0.0028 g (0.015 mmol) of $Co(OAc)_2$, 0.0078 g (0.018 mmol) of **L5**, 1.2 mL (0.25 M) of EA, and 0.49 g (7.5 mmol) of Zn, 3 mL (0.1 M) of mixture AcOH-THF-H_2O (3/1/1, v/v/v), 85 μL (0.728 g/mL, 0.6 mmol) of Et_3N, 40 μL (0.45 mmol) of BzCl and 3 mL (0.1 M) of THF. The title compound (0.0578 g, 62% yield, 91.9/8.1 er) was obtained as a white solid after silica-gel column chromatography (PE/EA = 7/1, 700 mL). Optical Rotation: $[\alpha]^{20}_{D}$ = -11.6 (c 0.995, $CHCl_3$).

The *er* was determined by HPLC, HPLC conditions: Chiralcel OD-H, *n*-hexane/*i*-PrOH = 90/10, flow rate = 1.0 mL/min, n = 254 nm, retention times (t_r) = 20.3 min (minor) and 24.3 min (major).

M.p.: 112.2-114.0 °C. IR (neat): 3309, 2930, 2855, 1631, 1536, 1514, 1462, 1306 cm^{-1}.

^1H NMR: (400 MHz, $CDCl_3$) δ 7.76-7.68 (m, 2H), 7.51-7.44 (m, 1H), 7.44-7.36 (m, 2H), 7.07 (d, J = 8.4 Hz, 2H), 6.80 (d, J = 8.4 Hz, 2H), 5.92 (d, J = 8.0 Hz, 1H), 4.25-4.12 (m, 1H), 3.76 (s, 3H), 2.55 (t, J = 7.6 Hz, 2H), 1.70-1.50 (m, 4H), 1.45-1.35 (m, 2H), 1.21 (d, J = 6.4 Hz, 3H); ^{13}C NMR: (100 MHz, $CDCl_3$) δ 166.8, 157.6, 135.0, 134.5, 131.2, 129.2, 128.5, 126.8, 113.6, 55.2, 45.6, 36.8, 34.7, 31.4, 25.5, 21.0; HRMS (ESI) calculated for $C_{20}H_{25}NNaO_2$ (M+Na$^+$) requires m/z 334.1778, found m/z 334.1778.

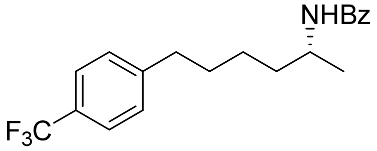

(*R*)-*N*-(6-(4-(trifluoromethyl)phenyl)hexan-2-yl)benzamide (21)

Prepared according to the general procedure E**,** using 0.1372 g (0.6 mmol) of 1-(hex-5-en-1-yl)-4-(trifluoromethyl)benzene (**1aaa**), 58 μL (1.129 g/ml, 0.3 mmol) of *tert*-butyl 2-diazo-2-phenylacetate (**2c**), 74 μL (0.877 g/mL, 0.6 mmol) of $PhSiH_3$, 100 μL (0.93 g/mL, 0.9 mmol) of 2-ethoxyethanol, 0.0027 g (0.015 mmol) of $Co(OAc)_2$, 0.0078 g (0.018 mmol) of **L5**, 1.2 mL (0.25 M) of EA, and 0.49 g (7.5 mmol) of Zn, 3 mL (0.1 M) of mixture AcOH-THF-H_2O (3/1/1, v/v/v), 85 μL (0.728 g/mL, 0.6 mmol) of Et_3N, 40 μL (0.45 mmol) of BzCl and 3 mL (0.1 M) of THF. The title compound (0.0775 g, 74% yield, 90.7/9.3 er) was obtained as a white solid after silica-gel column chromatography (PE/EA = 10/1, 1000 mL). Optical Rotation: $[\alpha]^{20}_D$ = -7.4 (c 0.995, $CHCl_3$).

The *er* was determined by HPLC, HPLC conditions: Chiralcel OD-H, *n*-hexane/*i*-PrOH = 90/10, flow rate = 1.0 mL/min, n = 254 nm, retention times (t_r) = 13.3 min (minor) and 17.6 min (major).

M.p.: 122.6-124.3 °C. IR (neat): 3307, 2932, 2856, 1630, 1533, 1462, 1330 cm^{-1}. ^1H NMR: (400 MHz, $CDCl_3$) δ 7.80-7.68 (m, 2H), 7.54-7.45 (m, 3H), 7.45-7.36 (m, 2H), 7.25 (d, J = 8.0 Hz, 2H), 5.96 (d, J = 8.4 Hz, 1H), 4.26-4.10 (m, 1H), 2.66 (t, J = 7.6 Hz, 2H), 1.76-1.50 (m, 4H), 1.48-1.34 (m, 2H), 1.22 (d, J = 6.4 Hz, 3H); ^{13}C NMR: (100 MHz, $CDCl_3$) δ 166.8, 146.5, 134.9, 131.3, 128.6, 128.5, 128.0 (q, J = 32.1 Hz), 126.7, 125.1 (q, J = 3.7 Hz), 124.3 (q, J = 272.4 Hz), 45.5, 36.7, 35.5, 30.8, 25.5, 21.0; ^{19}F NMR: (376 MHz, $CDCl_3$) δ -62.2. HRMS (ESI) calculated for $C_{20}H_{22}F_3NNaO$

(M+Na$^+$) requires m/z 372.1546, found m/z 372.1543.

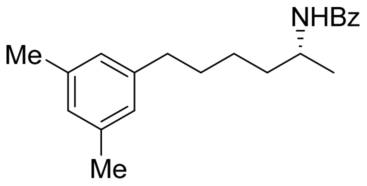

(*R*)-*N*-(6-(3,5-dimethylphenyl)hexan-2-yl)benzamide (22)

Prepared according to the general procedure E, using 0.1115 g (0.6 mmol) of 1-(hex-5-en-1-yl)-3,5-dimethylbenzene (**1aab**), 58 μL (1.129 g/ml, 0.3 mmol) of *tert*-butyl 2-diazo-2-phenylacetate (**2c**), 74 μL (0.877 g/mL, 0.6 mmol) of $PhSiH_3$, 100 μL (0.93 g/mL, 0.9 mmol) of 2-ethoxyethanol, 0.0027 g (0.015 mmol) of $Co(OAc)_2$, 0.0079 g (0.018 mmol) of **L5**, 1.2 mL (0.25 M) of EA, and 0.49 g (7.5 mmol) of Zn, 3 mL (0.1 M) of mixture AcOH-THF-H_2O (3/1/1, v/v/v), 85 μL (0.728 g/mL, 0.6 mmol) of Et_3N, 40 μL (0.45 mmol) of BzCl and 3 mL (0.1 M) of THF. The title compound (0.0553 g, 60% yield, 91.7/8.3 er) was obtained as a white solid after silica-gel column chromatography (PE/EA = 10/1, 1000 mL). Optical Rotation: $[\alpha]^{20}_D$ = -8.3 (c 1.020, $CHCl_3$).

The *er* was determined by HPLC, HPLC conditions: Chiralcel OD-H, *n*-hexane/*i*-PrOH = 90/10, flow rate = 1.0 mL/min, n = 254 nm, retention times (t_r) = 10.7 min (minor) and 17.4 min (major).

M.p.: 109.8-111.2 °C. IR (neat): 3304, 2926, 2854, 1633, 1537, 1462, 1309 cm^{-1}. ^{1}H NMR: (400 MHz, $CDCl_3$) δ 7.77-7.69 (m, 2H), 7.52-7.45 (m, 1H), 7.45-7.37 (m, 2H), 6.80 (s, 1H), 6.78 (s, 2H), 5.90 (d, *J* = 8.0 Hz, 1H), 4.26-4.12 (m, 1H), 2.53 (t, *J* = 7.6 Hz, 2H), 2.26 (s, 6H), 1.70-1.50 (m, 4H), 1.48-1.36 (m, 2H), 1.22 (d, *J* = 6.8 Hz, 3H); ^{13}C NMR: (100 MHz, $CDCl_3$) δ 166.8, 142.3, 137.7, 135.0, 131.2, 128.5, 127.3, 126.8, 126.2, 45.7, 36.9, 35.6, 31.3, 25.7, 21.2, 21.0; HRMS (ESI) calculated for $C_{21}H_{28}NO$ (M+H$^+$) requires m/z 310.2165, found m/z 310.2166.

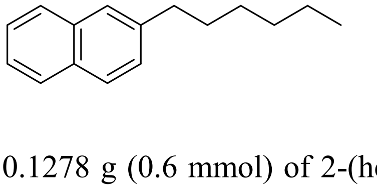

(*R*)-*N*-(6-(naphthalen-2-yl)hexan-2-yl)benzamide (23)

Prepared according to the general procedure E, using 0.1278 g (0.6 mmol) of 2-(hex-5-en-1-yl)naphthalene (**1aac**), 58 μL (1.129 g/ml, 0.3 mmol) of *tert*-butyl 2-diazo-2-phenylacetate (**2c**), 74 μL (0.877 g/mL, 0.6 mmol) of

PhSiH$_3$, 100 μL (0.93 g/mL, 0.9 mmol) of 2-ethoxyethanol, 0.0027 g (0.015 mmol) of Co(OAc)$_2$, 0.0079 g (0.018 mmol) of **L5**, 1.2 mL (0.25 M) of EA, and 0.49 g (7.5 mmol) of Zn, 3 mL (0.1 M) of mixture AcOH-THF-H$_2$O (3/1/1, v/v/v), 85 μL (0.728 g/mL, 0.6 mmol) of Et$_3$N, 40 μL (0.45 mmol) of BzCl and 3 mL (0.1 M) of THF. The title compound (0.0726 g, 73% yield, 91.4/8.6 er) was obtained as a white solid after silica-gel column chromatography (PE/EA = 10/1, 1000 mL). Optical Rotation: $[\alpha]^{20}_{D}$ = -5.2 (c 0.985, CHCl$_3$).

The *er* was determined by HPLC, HPLC conditions: Chiralcel OD-H, *n*-hexane/*i*-PrOH = 85/15, flow rate = 1.0 mL/min, n = 254 nm, retention times (t_r) = 19.7 min (minor) and 42.5 min (major).

M.p.: 118.7-120.3 ^oC. IR (neat): 3306, 2929, 2855, 1633, 1537, 1457, 1309 cm^{-1}. ^{1}H NMR: (400 MHz, CDCl$_3$) δ 7.84-7.66 (m, 5H), 7.59 (s, 1H), 7.52-7.34 (m, 5H), 7.31 (dd, *J* = 8.0, 1.2 Hz, 1H), 5.83 (d, *J* = 7.6 Hz, 1H), 4.30-4.12 (m, 1H), 2.78 (t, *J* = 7.6 Hz, 2H), 1.84-1.68 (m, 2H), 1.66-1.54 (m, 2H), 1.52-1.40 (m, 2H), 1.22 (d, *J* = 6.4 Hz, 3H); ^{13}C NMR: (100 MHz,) δ 166.8, 139.9, 135.0, 133.6, 131.9, 131.2, 128.5, 127.8, 127.6, 127.4, 127.3, 126.7, 126.3, 125.8, 125.0 , 45.6, 36.9, 35.9, 31.0, 25.6, 21.0; HRMS (ESI) calculated for $C_{23}H_{25}NNaO$ (M+Na$^+$) requires m/z 354.1828, found m/z 354.1828.

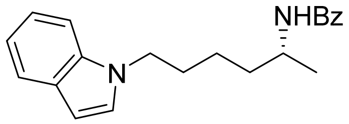

(*R*)-*N*-(6-(1*H*-indol-1-yl)hexan-2-yl)benzamide (24)

Prepared according to the general procedure E**,** using 0.1172 g (0.6 mmol) of 1-(hex-5-en-1-yl)-1*H*-indole (**1ah**), 58 μL (1.129 g/ml, 0.3 mmol) of *tert*-butyl 2-diazo-2-phenylacetate (**2c**), 74 μL (0.877 g/mL, 0.6 mmol) of PhSiH$_3$, 100 μL (0.93 g/mL, 0.9 mmol) of 2-ethoxyethanol, 0.0029 g (0.015 mmol) of Co(OAc)$_2$, 0.0079 g (0.018 mmol) of **L5**, 1.2 mL (0.25 M) of EA, and 0.49 g (7.5 mmol) of Zn, 3 mL (0.1 M) of mixture AcOH-THF-H$_2$O (3/1/1, v/v/v), 85 μL (0.728 g/mL, 0.6 mmol) of Et$_3$N, 40 μL (0.45 mmol) of BzCl and 3 mL (0.1 M) of THF. The title compound (0.0705 g, 73% yield, 88.5/11.5 er) was obtained as a yellow solid after silica-gel column chromatography (PE/EA = 5/1, 1000 mL). Optical Rotation: $[\alpha]^{20}_{D}$ = +6.1 (c 0.990, CHCl$_3$).

The *er* was determined by HPLC, HPLC conditions: Chiralcel OD-H, *n*-hexane/*i*-PrOH = 80/20, flow rate = 1.0 mL/min, n = 254 nm, retention times (t_r) = 26.9 min (minor) and 33.5 min (major).

M.p.: 98.2-100.0 °C. IR (neat): 3312, 3057, 2927, 2859, 1635, 1538, 1460, 1314 cm^{-1}. ^{1}H NMR: (400 MHz, $CDCl_3$) δ 7.72-7.64 (m, 2H), 7.61 (d, J = 8.0 Hz, 1H), 7.50-7.43 (m, 1H), 7.43-7.35 (m, 2H), 7.32 (d, J = 8.4 Hz, 1H), 7.21-7.14 (m, 1H), 7.11-7.03 (m, 2H), 6.45 (d, J = 3.2 Hz, 1H), 5.88 (d, J = 8.4 Hz, 1H), 4.23-4.02 (m, 3H), 1.96-1.74 (m, 2H), 1.60-1.45 (m, 2H), 1.43-1.30 (m, 2H), 1.17 (d, J = 6.4 Hz, 3H); ^{13}C NMR: (100 MHz, $CDCl_3$) δ 166.9, 135.9, 134.8, 131.2, 128.51, 128.46, 127.7, 126.8, 121.3, 120.9, 119.1, 109.3, 100.9, 46.0, 45.4, 36.5, 29.9, 23.4, 20.9; HRMS (ESI) calculated for $C_{21}H_{24}N_2NaO$ (M+Na$^+$) requires m/z 343.1781, found m/z 343.1782.

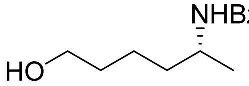

(*R*)-*N*-(6-hydroxyhexan-2-yl)benzamide (25)

Prepared according to the general procedure E**,** using 0.0611 g (0.6 mmol) of hex-5-en-1-ol (**1ad**), 58 μL (1.129 g/ml, 0.3 mmol) of *tert*-butyl 2-diazo-2-phenylacetate (**2c**), 74 μL (0.877 g/mL, 0.6 mmol) of $PhSiH_3$, 100 μL (0.93 g/mL, 0.9 mmol) of 2-ethoxyethanol, 0.0029 g (0.015 mmol) of $Co(OAc)_2$, 0.0080 g (0.018 mmol) of **L5**, 1.2 mL (0.25 M) of EA, and 0.49 g (7.5 mmol) of Zn, 3 mL (0.1 M) of mixture AcOH-THF-H_2O (3/1/1, v/v/v), 85 μL (0.728 g/mL, 0.6 mmol) of Et_3N, 40 μL (0.45 mmol) of BzCl and 3 mL (0.1 M) of THF. The title compound (0.0254 g, 38% yield, 89.6/10.4 er) was obtained as a pale yellow solid after silica-gel column chromatography (PE/EA = 1/1, 500 mL). Optical Rotation: $[\alpha]^{20}_D$ = -16.7 (c 0.585, $CHCl_3$).

The *er* was determined by HPLC, HPLC conditions: Chiralcel OD-H, *n*-hexane/*i*-PrOH = 90/10, flow rate = 1.0 mL/min, n = 254 nm, retention times (t_r) = 12.8 min (minor) and 24.4 min (major).

M.p.: 91.7-93.2 °C. IR (neat): 3299, 2935, 2864, 1637, 1544, 1455, 1308 cm^{-1}. ^{1}H NMR: (400 MHz, $CDCl_3$) δ 7.79-7.72 (m, 2H), 7.51-7.45 (m, 1H), 7.44-7.37 (m, 2H), 6.09 (d, J = 8.0 Hz, 1H), 4.27-4.13 (m, 1H), 3.63 (t, J = 6.4 Hz, 2H), 2.03 (br, 1H), 1.70-1.51 (m, 4H), 1.51-1.40 (m, 2H), 1.24 (d, J = 6.4 Hz, 3H); ^{13}C NMR: (100 MHz, $CDCl_3$) δ

167.0, 134.8, 131.3, 128.5, 126.8, 62.5, 45.6, 36.7, 32.3, 22.3, 21.0; HRMS (ESI) calculated for $C_{13}H_{19}NNaO_2$ (M+Na$^+$) requires m/z 244.1308, found m/z 244.1308.

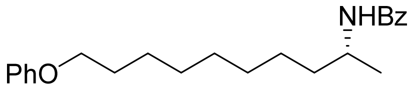

(*R*)-*N*-(10-phenoxydecan-2-yl)benzamide (26)

Prepared according to the general procedure E**,** using 0.1312 g (0.6 mmol) of (dec-9-en-1-yloxy)benzene (**1aad**), 58 μL (1.129 g/ml, 0.3 mmol) of *tert*-butyl 2-diazo-2-phenylacetate (**2c**), 74 μL (0.877 g/mL, 0.6 mmol) of $PhSiH_3$, 100 μL (0.93 g/mL, 0.9 mmol) of 2-ethoxyethanol, 0.0029 g (0.015 mmol) of $Co(OAc)_2$, 0.0079 g (0.018 mmol) of **L5**, 1.2 mL (0.25 M) of EA, and 0.49 g (7.5 mmol) of Zn, 3 mL (0.1 M) of mixture AcOH-THF-H_2O (3/1/1, v/v/v), 85 μL (0.728 g/mL, 0.6 mmol) of Et_3N, 40 μL (0.45 mmol) of BzCl and 3 mL (0.1 M) of THF. The title compound (0.0674 g, 64% yield, 91.2/8.8 er) was obtained as a white solid after silica-gel column chromatography (PE/EA = 10/1, 1000 mL). Optical Rotation: $[\alpha]^{20}_D$ = -10.0 (c 1.010, $CHCl_3$).

The *er* was determined by HPLC, HPLC conditions: Chiralcel OD-H, *n*-hexane/*i*-PrOH = 90/10, flow rate = 1.0 mL/min, n = 254 nm, retention times (t_r) = 22.8 min (major) and 29.8 min (minor).

M.p.: 84.7-86.3 °C. IR (neat): 3305, 2927, 2854, 1633, 1538, 1495, 1301 cm^{-1}. ^{1}H NMR: (400 MHz, $CDCl_3$) δ 7.79-7,71 (m, 2H), 7.52-7.44 (m, 1H), 7.44-7.37 (m, 2H), 7.31-7.22 (m, 2H), 6.96-6.82 (m, 3H), 5.94 (d, *J* = 8.0 Hz, 1H), 4.25-4.12 (m, 1H), 3.93 (t, *J* = 6.8 Hz, 2H), 1.82-1.70 (m, 2H), 1.62-1.49 (m, 2H), 1.49-1.27 (m, 10H), 1.22 (d, *J* = 6.8 Hz, 3H); ^{13}C NMR: (100 MHz, $CDCl_3$) δ 166.8, 159.1, 135.0, 131.2, 129.3, 128.5, 126.8, 120.4, 114.4, 67.8, 45.7, 37.0, 29.42, 29.39, 29.3, 29.2, 26.04, 25.97, 21.0; HRMS (ESI) calculated for $C_{23}H_{31}NNaO_2$ (M+Na$^+$) requires m/z 376.2247, found m/z 376.2248.

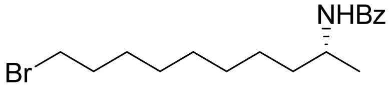

(*R*)-*N*-(10-bromodecan-2-yl)benzamide (27)

Prepared according to the general procedure E**,** using 0.1293 g (0.6 mmol) of 10-bromodec-1-ene (**1aae**), 58 μL (1.129 g/ml, 0.3 mmol) of *tert*-butyl 2-diazo-2-phenylacetate (**2c**), 74 μL (0.877 g/mL, 0.6 mmol) of $PhSiH_3$, 100

μL (0.93 g/mL, 0.9 mmol) of 2-ethoxyethanol, 0.0028 g (0.015 mmol) of $Co(OAc)_2$, 0.0079 g (0.018 mmol) of **L5**, 1.2 mL (0.25 M) of EA, and 0.49 g (7.5 mmol) of Zn, 3 mL (0.1 M) of mixture AcOH-THF-H_2O (3/1/1, v/v/v), 85 μL (0.728 g/mL, 0.6 mmol) of Et_3N, 40 μL (0.45 mmol) of BzCl and 3 mL (0.1 M) of THF. The title compound (0.0836 g, 82% yield, 92.0/8.0 er) was obtained as a pale yellow solid after silica-gel column chromatography (PE/EA = 10/1, 1000 mL). Optical Rotation: $[\alpha]^{20}_D$ = -8.9 (c 0.985, $CHCl_3$).

The *er* was determined by HPLC, HPLC conditions: Chiralcel OD-H, *n*-hexane/*i*-PrOH = 90/10, flow rate = 1.0 mL/min, n = 254 nm, retention times (t_r) = 11.8 min (minor) and 14.1 min (major).

M.p.: 58.7-59.9 °C. IR (neat): 3311, 3063, 2928, 2855, 1635, 1541, 1458, 1303 cm^{-1}. ^1H NMR: (400 MHz, $CDCl_3$) δ 7.79-7.70 (m, 2H), 7.52-7.45 (m, 1H), 7.45-7.38 (m, 2H), 5.96 (d, J = 8.4 Hz, 1H), 4.26-4.10 (m, 1H), 3.39 (t, J = 6.8 Hz, 2H), 1.90-1.77 (m, 2H), 1.60-1.46 (m, 2H), 1.46-1.25 (m, 10H), 1.23 (d, J = 6.8 Hz, 3H); ^{13}C NMR: (100 MHz, $CDCl_3$) δ 167.8, 134.4, 131.6, 128.6, 126.8, 46.1, 36.9, 34.0, 32.7, 29.29, 29.26, 28.6, 28.1, 26.0, 20.9; HRMS (ESI) calculated for $C_{17}H_{26}BrNNaO$ (M+Na^+) requires m/z 362.1090, found m/z 362.1090.

Mechanistic Studies

Radical-trapping experiment

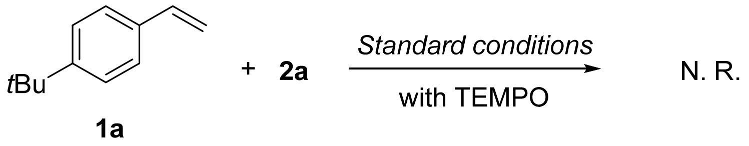

The experiment was runned according to the general procedure A using 0.0579 g (0.36 mmol) of 4-*tert*-butylstyrene (**1a**), 49 μL (1.165 g/ml, 0.3 mmol) of ethyl 2-diazo-2-phenylacetate (**2a**), 45 μL (0.877 g/mL, 0.36 mmol) of $PhSiH_3$, 0.0027 g (0.015 mmol) of $Co(OAc)_2$, 0.0058 g (0.018 mmol) of **L4**, and 1.2 mL (0.25 M) of THF, with additionally 0.0469 g (0.3 mmol) TEMPO as a radical scavenger. After 12 h, the

reaction was worked up. No desired amination was detected from ^{1}H NMR.

Radical-clock experiment

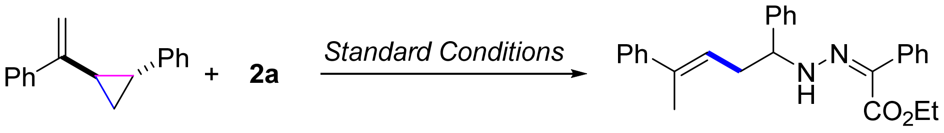

28 **29**

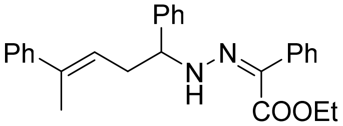

Ethyl (*Z*)-2-(2-((*E*)-1,4-diphenylpent-3-en-1-yl)hydrazono)-2-phenylacetate (29).

Prepared according to the general procedure A using 0.0801 g (0.36 mmol) of (1-(2-phenylcyclopropyl)vinyl)benzene (**28**), 49 µL (1.165 g/ml, 0.3 mmol) of ethyl 2-diazo-2-phenylacetate (**2a**), 45 µL (0.877 g/mL, 0.36 mmol) of $PhSiH_3$, 0.0028 g (0.015 mmol) of $Co(OAc)_2$, 0.0057 g (0.018 mmol) of **L4**, and 1.2 mL (0.25 M) of THF. After 12 h, the reaction was worked up. The crude mixture was purified by flash column chromatography using PE to PE/EA (100/1, 200 mL) as the eluent to give 0.0764 g (0.19 mmol, 62% yield, *E*/*Z* > 20:1) of the title compound as a colorless oil. IR (neat): 3247, 3029, 2980, 1669, 1599, 1514, 1448 cm^{-1}. ^{1}H NMR: (400 MHz, $CDCl_3$) δ 10.91 (d, J = 4.4 Hz, 1H), 7.51 (d, J = 7.6 Hz, 2H), 7.40-7.15 (m, 13H), 5.76 (t, J = 7.2 Hz, 1H), 4.84-4.72 (m, 1H), 4.68-4.58 (m, 0.04H), 4.25 (q, J = 7.2 Hz, 2H), 3.05-2.92 (m, 1H), 2.87-2.75 (m, 1H), 1.97 (s, 3H), 1.29 (t, 7.2 Hz, 3H); ^{13}C NMR: (100 MHz, $CDCl_3$) δ 163.6, 143.7, 141.9, 137.5, 137.1, 128.6, 128.3, 128.1, 127.7, 127.4, 126.9, 126.8, 126.7, 126.2, 125.7, 123.6, 65.0, 60.3, 35.6, 16.1, 14.2; HRMS (ESI) calculated for $C_{27}H_{29}N_2O_2$ (M+H$^+$) requires m/z 413.2229, found m/z 413.2227.

Deuterium labeling experiment

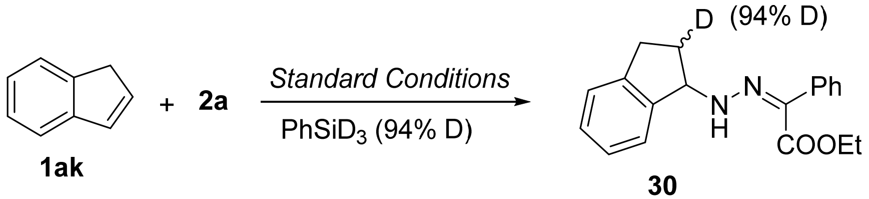

1ak 30

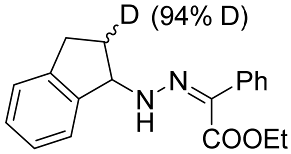

Ethyl (*Z*)-2-(2-((1*R*,2*S*)-2,3-dihydro-1*H*-inden-1-yl-2-d)hydrazono)-2-phenylacetate (30). Prepared according to the general procedure A using 0.0421 g (0.36 mmol) of 1*H*-indene (**1ak**), 49 μL (1.165 g/ml, 0.3 mmol) of ethyl 2-diazo-2-phenylacetate (**2a**), 45 μL (0.877 g/mL, 0.36 mmol, 94% D) of $PhSiD_3$, 0.0028 g (0.015 mmol) of $Co(OAc)_2$, 0.0058 g (0.018 mmol) of **L4**, and 1.2 mL (0.25 M) of THF. After 12 h, the reaction was worked up. The crude mixture was purified by flash column chromatography using PE to PE/EA (100/1, 200 mL) as the eluent to give 0.0825 g (0.27 mmol, 89% yield, 94% D, *dr* = 1/1) of the title compound as a light yellow oil. ^{1}H NMR: (400 MHz, $CDCl_3$) δ 10.73 (d, J = 5.6 Hz, 1H), 7.57 (d, J = 8.0 Hz, 2H), 7.40 (d, J = 6.8 Hz, 1H), 7.34 (t, J = 8.0 Hz, 2H), 7.30-7.18 (m, 4H), 5.21 (m, 1H), 4.25 (q, J = 7.2 Hz, 2H), 3.05 (dd, J = 16.0, 8.0 Hz, 1H), 2.88 (dd, J = 16.0, 8.0 Hz, 1H), 2.65-2.54 (m, 0.53H), 2.20-2.05 (m, 0.53H), 1.30 (t, J = 7.2 Hz, 3H); ^{2}D NMR: (77 MHz, $CHCl_3$) δ 7.26 (s, 15.48D), 2.62 (s, 1.00D), 2.14 (s, 1.01D);

Supplementary References

[1] Zheng, J.; Qi, J.; Cui, S. Fe-Catalyzed Olefin Hydroamination with Diazo Compounds for Hydrazone Synthesis. *Org. Lett.* **18**, 128–131 (2016).

[2.] Wen, H. A.; Wan, X. L.; Huang, Z. Asymmetric Synthesis of Silicon-Stereogenic Vinylhydrosilanes by Cobalt-Catalyzed Regio- and Enantioselective Alkyne Hydrosilylation with Dihydrosilanes. *Angew. Chem. Int. Ed.*, **57**, 6319–6323 (2018).

[3] Hou, S.; Yang, H.; Cheng, B.; Zhai, H.; Li, Yun. Cobaloxime-catalyzed hydration of terminal alkynes without acidic promoters. *Chem. Commoun*. **53**, 6926–6929 (2017).

[4] Raya, R.; Jing, S.; Balvsanthiran, V.; RajanBabu, T. V. Control of Selectivity through Synergy between Catalysts, Silanes, and Reaction Conditions in Cobalt-Catalyzed Hydrosilylation of Dienes and Terminal Alkenes. *ACS Catal*. **7**, 2275–2283 (2017).

5 Yeung, K.; To, W.; Sun, C.; Cheng, G.; Ma, C.; Tong, G. S. M. Yang, C.; Che, C. Luminescent Tungsten(VI) Complexes: Photophysics and Applicability to Organic Light-Emitting Diodes and Photocatalysis. *Angew. Chem. Int. Ed.* **56**, 133-137 (2017).

[6] Waser, J.; Nambu, H.; Carreira, E. M. Cobalt-Catalyzed Hydroazidation of Olefins: Convenient Access to Alkyl Azides. *J. Am. Chem. Soc*. **127**, 8294–8295 (2005).

[7] Chen, X.; Cheng, Z.; Lu, Z. Iron-Catalyzed, Markovnikov-Selective Hydroboration of Styrenes. *Org. Lett*. **19**, 969–971 (2017).

[8] Wolinska, E. Chiral oxazoline ligands containing a 1,2,4-triazine ring and their application in the Cu-catalyzed asymmetric Henry reaction *Tetrahedron* **69**, 7269–7278 (2013).

[9] Chen, X.; Cheng, Z.; Guo, J.; Lu, Z. Asymmetric remote C-H borylation of internal alkenes via alkene isomerization. *Nat. Commun*. **9**, 3939 (2018).

[10] Decken, A.; Gossage, R. A.; Yadav, P. N. Oxazoline chemistry. Part VIII. Synthesis and characterization of a new class of pincer ligands derived from the 2-(*o*-anilinyl)-2-oxazoline skeleton–Applications to the synthesis of group X transition metal catalysts. *Can. J. Chem*. **83**, 1185–1189 (2005).

[11] Chen, X.; Cheng, Z.; Lu, Z. Chiral quinolinamine compound, preparation method and application. Chinese patent CN 108707144, (2018).

[12] Cheng, B.; Liu, W.-B.; Lu, Z. Iron-Catalyzed Highly Enantioselective Hydrosilylation of

Unactivated Terminal Alkenes. *J. Am. Chem. Soc.* **140**, 5014−5017 (2018).

[13] San, H. H.; Wang, S.; Jiang, M.; Tang, X. Boron-Catalyzed O–H Bond Insertion of α-Aryl α-Diazoesters in Water. *Org. Lett.* **20**, 4672–4676 (2018).

[14] Cai, Y.; Yang, X.; Zhang, S.; Li, F.; Li, Y.; Ruan, L.; Hong, X.; Shi, S. Copper-Catalyzed Enantioselective Markovnikov Protoboration of α-Olefins Enabled by a Buttressed N-Heterocyclic Carbene Ligand. *Angew. Chem. Int. Ed.* **57**, 1376–1380 (2018).

[15] Cheng, Q.; Zhu, S.; Zhang, Y.; Xie, X.; Zhou, Q. Copper-Catalyzed B–H Bond Insertion Reaction: A Highly Efficient and Enantioselective C–B Bond-Forming Reaction with Amine–Borane and Phosphine–Borane Adducts. *J. Am. Chem. Soc.* **135**, 14094–14097 (2013).

[16] Parsons, A. T.; Buchwald, S. Copper-Catalyzed Trifluoromethylation of Unactivated Olefins. *Angew. Chem. Int. Ed.* **50**, 9120–9123 (2011).

[17] Atienza, B. J. P.; Truong, N.; Willians, F. J. Reliably Regioselective Dialkyl Ether Cleavage with Mixed Boron Trihalides. *Org. Lett.* **20**, 6332–6335 (2018).

[18] Thibon, J.; Latxague, L.; Déléris, G. Synthesis of Silicon Analogues of Acyclonucleotides Incorporable in Oligonucleotide Solid-Phase Synthesis. *J. Org. Chem.* **62**, 4635–4642 (1997).

[19] Lei, Z.; Banerjee, A.; Kusevska, E.; Rizzo, E.; Liu, P. β-Selective Aroylation of Activated Alkenes by Photoredox Catalysis. *Angew. Chem. Int. Ed.* **58**, 7318–7323 (2019).

[20.]Cheng, B.; Liu, W.; Lu, Z. Iron-Catalyzed Highly Enantioselective Hydrosilylation of Unactivated Terminal Alkenes. *J. Am. Chem. Soc.* **140**, 5014–5017 (2018).

[21] Panish, R.: Selvaraj, R.; Fox, J. M. Rh(II)-Catalyzed Reactions of Diazoesters with Organozinc Reagents. *Org. Lett.* **17**, 3978−3981 (2015).

[22] Johns, A. M.; Liu, Z.; Hartwig, J. F. Primary *tert*- and *sec*-Allylamines via Palladium-Catalyzed Hydroamination and Allylic Substitution with Hydrazine and Hydroxylamine Derivatives. *Angew. Chem. Int. Ed.* **46**, 7259–7261 (2007).

[23] Xie, Y.; Pan, H.; Xiao, X.; Li, S.; Shi, Y. Organocatalytic asymmetric biomimetic transamination of aromatic ketone to optically active amine. *Org. Biomol. Chem.* **10**, 8960–8962 (2012).

[24] Bornholdt, J.; Felding, J.; Clausen, R. P.; Kristensen, J. L. Ring Opening of Pymisyl-Protected Aziridines with Organocuprates. *Chem. Eur. J.* **16**, 12474–12480 (2010).

[25] Bakhoda, A. G.; Jiang, Q.; Badiei, Y. M.; Bertke, J. A.; Cundari, T. R. Warren, T. H. Copper-Catalyzed C(sp^3)−H Amidation: Sterically Driven Primary and Secondary C−H Site-Selectivity

Angew. Chem. Int. Ed. **58**, 3421–3425 (2019).

[26] Yu, W. Z.; Cheng, Y. A.; Wong, M. W.; Yeung, Y. Y. Atmosphere- and Temperature-Controlled Regioselective Aminobromination of Olefins. *Adv. Synth. Catal.* **359**, 234–239 (2017).

[27] Li, S.; Huang, K.; Zhang, J.; Wu, W.; Zhang, X. Cascade Synthesis of Fenpiprane and Related Pharmaceuticals via Rhodium-Catalyzed Hydroaminomethylation. *Org. Lett.* **15**, 1036–1039 (2013).

[28] Kerr, D. I. B.; Ong, J.; Perkins, M.; Prager, R. H.; Puspawati, N. M. Synthesis and Biological Activity of Allosteric Modulators of $GABA_B$ Receptors, Part 1. *N*-(Phenylpropyl)-1-arylethylamines. *Aust. J. Chem.* ***59***, 445–456 (2006).

[29] Karnik, A. V.; Kamath, S. S. *Tetrahedron: Asymmetry* **19**, 45–48 (2008).

[30] Mondal, D.; Bellucci, L.; Lepore, S. D. A Direct and Stereoretentive Synthesis of Amides from Cyclic Alcohols. *Eur. J. Org. Chem.* **2011**, 7057–7061 (2011).

[31] Sevov, C. S.; Zhou, J. S.; Hartwig, J. F. Iridium-Catalyzed Intermolecular Hydroamination of Unactivated Aliphatic Alkenes with Amides and Sulfonamides. *J. Am. Chem. Soc.* **134**, 11960–11963 (2012).

[32.] Das, S.; Majumdar, N.; De, C. K.; Kundu, D. S.; Döhring, A.; Garczynski, A.; List, B. Asymmetric Catalysis of the Carbonyl-Amine Condensation: Kinetic Resolution of Primary Amines. *J. Am. Chem. Soc.* **139**, 1357–1359 (2017).
